# Supplementary material for: Pyridine-based strategies towards nitrogen isotope exchange and multiple isotope incorporation
Source: Nat Commun. 2024 Jul 18;15:6063. doi: 10.1038/s41467-024-50139-w (PMC11258231; doi:10.1038/s41467-024-50139-w)
Supplement: Supplementary file 1 — Supplementary Information [file 41467_2024_50139_MOESM1_ESM.pdf]

Supplementary information

Pyridine-based Strategies towards Nitrogen Isotope Exchange and Multiple Isotope Incorporation

Minghao Feng,[1] Maylis Norlöff,[1] Benoit Guichard,[1] Steven Kealey,[2] Timothée D'Anfray,[1] Pierre Thuéry,[3] Frédéric Taran,[1] Antony Gee,[2] Sophie Feuillastre,[*,1] Davide Audisio[*,1]

Affiliations:

[1] Université Paris Saclay, CEA, INRAE, Département Médicaments et Technologies pour la Santé (DMTS), SCBM, 91191 Gif-sur-Yvette, France.

[2] King's College London, School of Biomedical Engineering and Imaging Sciences, Department of Imaging Chemistry and Biology, 4th Floor Lambeth Wing, St Thomas' Hospital, London, SE1 7EH, UK.

[3] Université Paris-Saclay, CEA, CNRS, NIMBE, 91191 Gif-sur-Yvette, France.

*Corresponding authors. Email: sophie.feuillastre@cea.fr; davide.audisio@cea.fr

Table of contents

1. **Materials and equipment** 3
2. **Investigations of ^{15}*N* labeling on Zincke pyridinium** 5
3. **Investigations of ^{15}*N* labeling of pyridines** 6
4. **Proposed Mechanism** 8
5. **Preparation of starting materials** 9
6. **General Procedures of ^{15}*N*-labeling of pyridines** 9

   5.1 General procedures of ^{15}*N*-labeling of pyridines 9

   5.2 General procedures of ^{15}*N*-labeling of pyridines with Zincke salts 11
7. **General procedures of nitrogen-carbon exchange of pyridines** 12
8. **Characterizations of the prepared compounds** 14

   7.1 Characterizations of starting materials 14

   7.2 Characterizations of ^{15}*N*-labeled pyridines 16

   7.3 Characterizations of ^{15}*N*-labeled pharmaceuticals and bioactive molecules 36

   7.4 Characterizations of nitrogen-carbon exchange products 43
9. **Unsuccessful substrates of ^{15}*N* labeling of pyridines** 51
10. **Reduction of [$^2H_5$, $^{13}C_1$]benzophenone** 52
11. **^{13}N Radioactive experiments** 53
12. **Crystallography** 59
13. **NMR spectra and isotopic enrichment** 60

    12.1 Zincke salts 60

    13.2 ^{15}*N*-labeled pyridines 64

    13.3 ^{15}*N*-labeled pharmaceutical molecules 164

    13.4 Nitrogen-carbon exchange products 199
14. **Supplementary References** 221

1. Materials and equipment

***Reactants and solvents**:*

Unless otherwise noted, all reactions were carried out in oven-dried glassware
Commercially available chemicals were purchased from ABCR, Acros Organics, Sigma-Aldrich, Alfa Aesar, Combi-Blocks, Carbolution, Fluorochem, and TCI Europe and used as received unless otherwise stated. The following solvents were dried by distillation over the drying agents indicated in parentheses: THF (Sodium), Dichloromethane (CaH_2). Additional anhydrous solvents were purchased from Acros Organics, SigmaAldrich, Alfa Aesar and stored over molecular sieves under an argon atmosphere.

***Purifications**:*

Flash chromatography were performed on silica gel (Merck Kieselgel 60, grading 40-63 µm) or using automate Puriflash XS 520 Plus with pre-packed column RediSep® Rf (grading 35-70 µm).

Analysis:

Reactions were monitored by TLC carried out on silica 0,25 mm (60 F254, Merck) using UV light as visualizing agent. For staining, the TLC plates were dipped into a solution basic aqueous permanganate (1 g $KMnO_4$, 6 g K_2CO_3 and 0.1 g KOH in 100 mL H_2O) and developed with a heat gun.

Nuclear Magnetic Resonance (NMR) Spectroscopy: ^{1}H NMR (400 MHz), ^{13}C NMR (100 MHz), ^{19}F NMR (376 MHz), ^{15}N NMR (41 MHz), ^{2}H NMR (61 MHz) spectra were acquired on a Brucker Avance 400 MHz spectrometer. All the ^{15}N NMR spectra presented in the supporting information are protons decoupled, unless otherwise noted. Chemical shifts are reported in parts per million (ppm) downfield from residual solvents peaks and coupling constants are reported as Hertz (Hz). Splitting patterns are designated as singlet (s), broad singlet (br. s), doublet (d), triplet (t), quartet (q), quintet (quint), multiplet (m). Splitting patterns that could not be interpreted or easily visualized are designated as multiplet (m).

Accurate mass measurement and isotopic enrichment calculation were performed on a Waters Xevo® G2-XS QTof High Resolution mass spectrometer (HRMS) using an ElectroSpray Ionization (ESI) source. For compound characterization, accurate mass of the unlabeled compounds were measured, unless a high isotopic enrichment value allowed the accurate mass measurement of the labeled product. Measured and calculated HRMS data are not corrected for the mass of the electron.

LC-MS spectra were recorded on a Waters Acquity UPLC® equipped PDA eλ Detector and SQ Detector 2, mobile phase A: H_2O + 0.1% formic acid, mobile phase B: acetonitrile + 0.1% formic acid.

Infrared spectra (IR) were obtained on a Perkin Elmer UATR TWO FTIR spectrophotometer and are reported as wavelength numbers (cm^{-1}).

Nitrogen-13 radiolabeling:

Aqueous [^{13}N]NH_3 was produced on a GE PETtrace 880 cyclotron by proton irradiation of a liquid target containing [^{16}O]H_2O + ethanol (0.03% v/v) via the $^{16}O(p,\alpha)^{13}N$ nuclear reaction at a beam current of 15 µA for 1 min. Radiolabeling reactions were performed using an Eckert and Zielgler Modular-Lab radiosynthesizer in a lead shielded hot-cell. Analytical HPLC was performed using an Agilent 1260 Infinity system with an in-line LabLogic Flow-RAM radiodetector and fitted with a Phenomenex™ Luna

C18(2) column (5 µm, 100 Å, 250 x 4.6 mm) under a gradient elution of water or ammonium formate (50 mM, pH 4) vs acetonitrile (1 mL/min, 20-90% gradient over 10 min).

2. Investigations of ^{15}N labeling on Zincke pyridinium

Supplementary Table 1: Preliminary screening of reaction solvents and equivalency of ^{15}N-ammonium chloride[a].

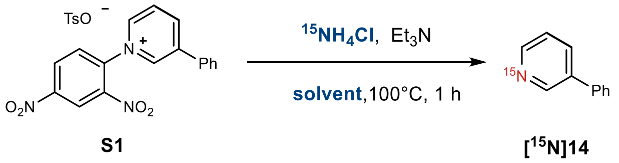

Entry	Equiv of $^{15}NH_4Cl$	Solvent	IE[b]	Yield (%)
1	3.0	acetonitrile	89	40
2	3.0	toluene	0	n.d.[c]
3	3.0	dioxane	0	n.d.
4	3.0	DMSO-d_6	84	44
5	3.0	*n*-propionitrile	85	79
6	1.5	*n*-propionitrile	85	75

Reaction conditions: [a] 3-Phenylpyridine Zincke salt (0.2 mmol, 1.0 equiv.), indicated equiv. of $^{15}NH_4Cl$, triethylamine (0.6 mmol, 3.0 equiv.), solvent (3.0 mL), 100 °C, 1 h. . [b] IE is the abbreviation of isotopic enrichment, measured by HRMS. [c]**:** n.d. is the abbreviation of not detected.

Supplementary Figure 1: ^{15}N-labeling scope of Zincke salts.[a,b]

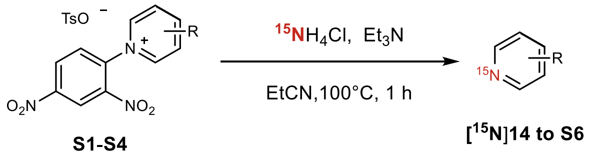

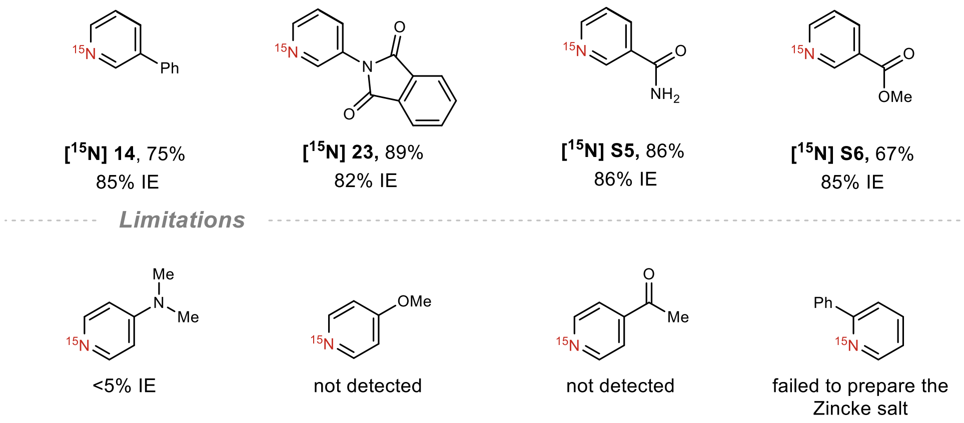

Reaction conditions: [a] Pyridine Zincke salt (0.2 mmol, 1.0 equiv.), $^{15}NH_4Cl$ (0.3 mmol, 1.5 equiv.), triethylamine (0.6 mmol, 3.0 equiv.), *n*-propionitrile (3.0 mL), 100 °C, 1 h.[b] IE is the abbreviation of isotopic enrichment, measured by HRMS.

3. Investigations of ^{15}N labeling of pyridines

Supplementary Table 2: Preliminary screening of reaction temperatures and equivalents of $^{15}NH_4Cl$.

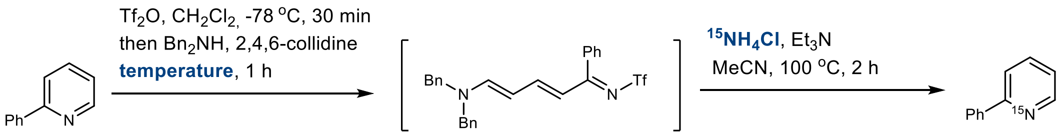

Entry	Equiv. of $^{15}NH_4Cl$	Temperature	IE[a]	NMR yield[b]
1	3.0	-78 to 25 °C	68%	99%
2	1.5	-78 to 25 °C	65%	81%
3	3.0	25 °C	77%	90%
4	3.0	40 °C	71%	88%
5[c]	3.0	60 °C	77%	87%

Reaction conditions: 2-Phenylpyridine (0.2 mmol, 1.0 equiv.), Tf_2O (0.2 mmol, 1.0 equiv.), dichloromethane (1.0 mL), -78 °C, 0.5 h, then dibenzylamine (0.24 mmol, 1.2 equiv.), 2,4,6-collidine (0.2 mmol, 1.0 equiv), indicated temperature, 1.0 h, then indicated equiv. of $^{15}NH_4Cl$, triethylamine (1.2 mmol, 6.0 equiv.), acetonitrile (2.0 mL), 100 °C, 1 h. **a:** IE is the abbreviation of isotopic enrichment, measured by LC-MS. **b:** ^{1}H-NMR yields using dibromomethane as an internal standard. **c:** Ethyl acetate was used instead of dichloromethane.

Supplementary Table 3: Screening of nucleophiles.

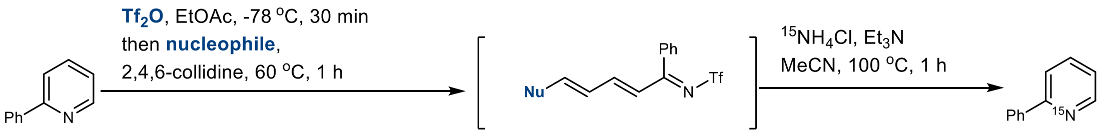

Entry	Equiv. of Tf_2O	Amine (equiv.)	IE[a]	NMR yield[b]
1	1.0	Bn_2NH (1.2)	77%	87%
2	1.2	Bn_2NH (3.0)	65%	95%
3	1.0	(-)-bis[(S)-1-phenyl-ethyl]amine (1.2)	<5%	80%
4	1.0	$MeNH_2$ (1.2)	<5%	65%
5	1.0	diethylamine (1.2)	<5%	51%
6	1.0	NH_2NH_2 (1.2)	<5%	99%
7	1.0	Indoline (1.2)	71%	99%
8	1.0	Tetrahydroquinoline (1.2)	42%	99%
9	1.0	*N*-methylaniline (1.2)	62%	97%
10	1.0	4-bromoaniline (1.2)	<5%	94%
11	1.0	2-methylindoline (1.2)	59%	95%
12	1.0	5-fluoroindoline (1.2)	32%	92%
13	1.0	5-bromoindoline (1.2)	73%	80%
14	1.0	5-cyanoindoline (1.2)	40%	76%
15	1.0	methyl indoline-5-carboxylate (1.2)	64%	80%
16	1.0	5-methoxyindoline (1.2)	70%	87%
17	1.0	7-methylindoline (1.2)	67%	98%
18	1.0	*N*-methylaniline (1.2)	62%	97%
19	1.0	*N*-benzylaniline (1.2)	62%	75%
20	1.0	4-bromoaniline (1.2)	<5%	94%

Reaction conditions: 2-Phenylpyridine (0.2 mmol, 1.0 equiv.), indicated equiv. of Tf_2O, ethyl acetate (1.0 mL), -78 °C, 0.5 h, then nucleophiles in the indicated equiv., 2,4,6-collidine (0.2 mmol, 1.0 equiv), 60 °C, 1.0 h, then $^{15}NH_4Cl$ (0.6 mmol, 3.0 equiv.), triethylamine (1.2 mmol, 6.0 equiv.), acetonitrile (2.0 mL), 100 °C, 1 h. **a:** IE is the abbreviation of isotopic enrichment, measured by LC-MS. **b:** ^{1}H-NMR yields using dibromomethane as an internal standard.

4. Proposed Mechanism

After activation of the pyridine with the triflic anhydride, dibenzylamine undergoes a nucleophilic addition to the pyridine at position 2. Subsequent electrocyclic ring opening (thermal, 6 π) allowed the obtention of the Zincke imine **Im1**, whose structure was confirmed by isolation and X-ray diffraction (see Section 11). Nitrogen isotopic exchange then took place through the insertion of the nitrogen-15 ammonia to the imine. After subsequent electrocyclic ring closure (thermal, 6 π) followed by rearomatization the nitrogen-labeled pyridines were afforded.

Supplementary Figure 2: proposed mechanism for the nitrogen isotope exchange.

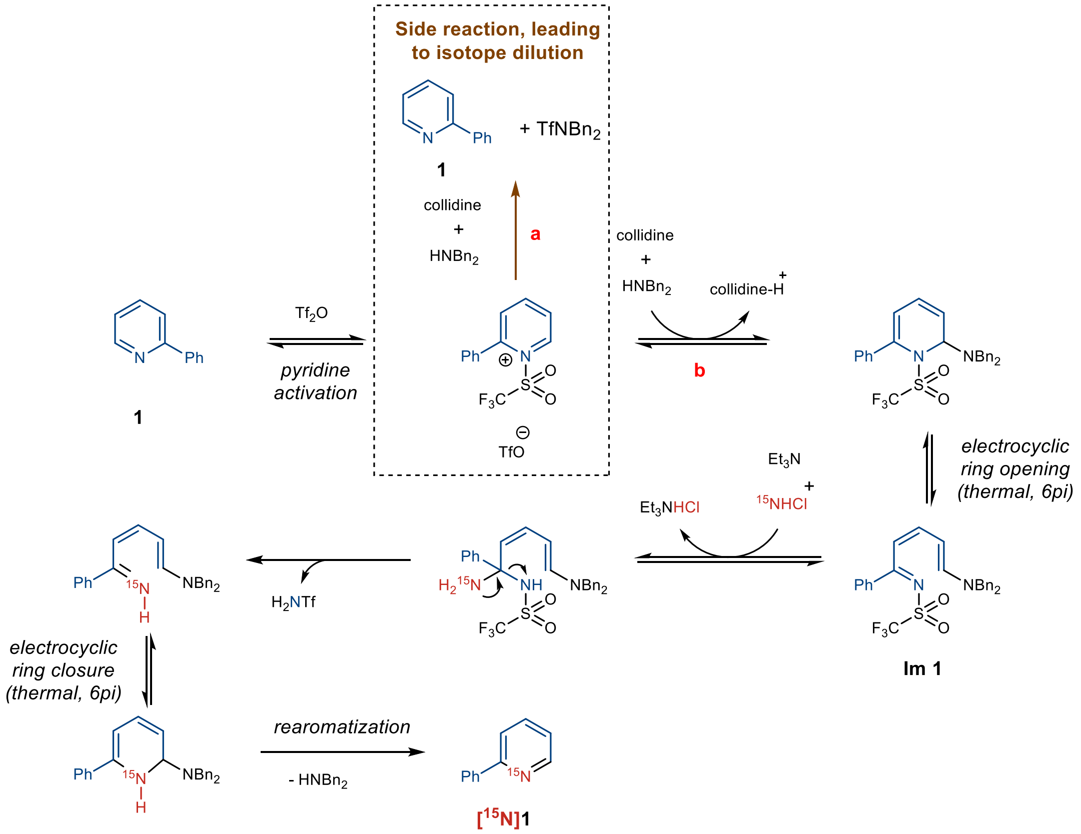

We believe that the exchange process is irreversible because the final rearomatization without an activating agent is unfavoured and because the $TfNH_2$ which is eliminated, will have a lower nucleophilicity and will not favour the opposite reaction. According to this arguments, we assumed that the maximal IE that can be achieved is the one of $[^{15}N]$ammonium chloride (i.e. $\geq$ 99%). However, isotopic dilution can be observed because, as described in the manuscript, the Bn_2NH may undergoes nucleophilic attack to the sulphur atom of the activated pyridinium to give the unlabeled SM is the key factor leading the drop of IE (Supplementary Figure 3).

Supplementary Figure 3: Regioselectivity of the nucleophilic addition.

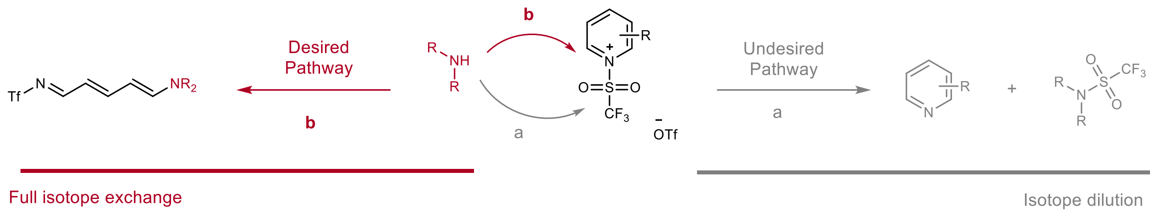

5. Preparation of starting materials

Preparation of Zincke pyridiniums

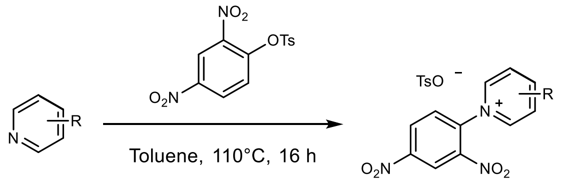

General procedure P: The procedure was adapted from the literature.[1] To a 25 mL round bottom flask equipped with a stirring bar, the heterocycle (1.00 mmol, 1.00 equiv.) and toluene (7.00 mL, 0.14M) were charged followed by the addition of 2,4-dinitrophenyl 4-methylbenzenesulfonate (372.1 mg, 1.10 mmol, 1.10 equiv.). The mixture was heated to reflux for 16 hours. After cooling to room temperature, the mixture was filtered on a büchner funnel, the precipitate was washed with dichloromethane (3 x 10.00 mL) and dried under reduced pressure. The obtained Zincke salts were used for the nitrogen isotopic exchange without further purification.

6. General Procedures of ^{15}*N*-labeling of pyridines

5.1 General procedures of ^{15}*N*-labeling of pyridines

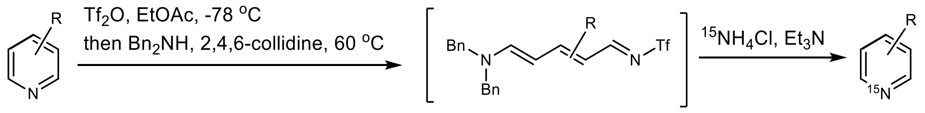

General Procedure A: To an oven-dried 8.00 mL pressure vial equipped with a stirring bar, the heterocycle (0.20 mmol, 1.00 equiv.) and ethyl acetate (1.00 mL) were charged under an argon atmosphere. The mixture was cooled to –78 °C and trifluoromethanesulfonic anhydride (33.6 µL, 0.20 mmol, 1.00 equiv.) was added dropwise. The reaction was stirred for 30 minutes at -78 °C before a solution of dibenzylamine (47.0 µL, 0.24 mmol, 1.20 equiv.) and 2,4,6-collidine (26.3 µL, 0.20 mmol, 1.00 equiv.) in ethyl acetate (0.25 mL) was added dropwise. The cooling bath was then removed and the reaction was warmed to 60 °C. After 1 hour, ^{15}*N*-ammonium chloride (32.7 mg, 0.60 mmol, 3.00 equiv.), triethylamine (167.3 µL, 1.20 mmol, 6.00 equiv.) and acetonitrile (2.50 mL) were added. The reaction was stirred at 100 °C for 1 hour. After cooling to room temperature, the reaction was diluted with dichloromethane (5.00 mL). The mixture was then washed with saturated aqueous ammonium chloride (5.00 mL). The aqueous phase was then extracted with dichloromethane (3 x 5.00 mL). The combined organic phases were dried over magnesium sulfate. The mixture was filtered and the filtrate was concentrated under vacuum to give the crude product. The crude product was purified with silica gel column chromatography. When the desired product presents the same retention factor as dibenzylamine on TLC, the crude product was dissolved in 1.00 mL of chloroform, di-*tert*-butyl dicarbonate (105 mg, 0.48 mmol, 2.40 equiv.) were added and the mixture was stirred at 25 °C until the complete conversion of dibenzylamine (approximately 4 hours). The solution was then concentrated and purified on silica gel column to give the desired product.

General Procedure B: To an oven-dried 8.00 mL pressure vial equipped with a stirring bar, the heterocycle (0.20 mmol, 1.00 equiv.) and dichloromethane (1.00 mL) were charged under argon atmosphere. The mixture was cooled to –78 °C and trifluoromethanesulfonic anhydride (33.6 µL, 0.20 mmol, 1.00 equiv.) was added dropwise. The reaction was stirred for 30 minutes at -78 °C before a solution of dibenzylamine (47.0 µL, 0.24 mmol, 1.20 equiv.) and 2,4,6-collidine (26.3 µL, 0.20 mmol,

1.00 equiv.) in dichloromethane (0.25 mL) was added dropwise. The cooling bath was then removed and the reaction was allowed to warm to 25 °C. After 1 hour, ^{15}N-ammonium chloride (32.7 mg, 0.60 mmol, 3.00 equiv.), triethylamine (167.3 µL, 1.20 mmol, 6.00 equiv.) and acetonitrile (2.50 mL) were added. The reaction was stirred at 100 °C for 1 hour. After cooling to room temperature, the reaction was diluted with dichloromethane (5.00 mL). The mixture was then washed with saturated aqueous ammonium chloride (5.00 mL). The aqueous phase was then extracted with dichloromethane (3 x 5.00 mL). The combined organic phases were dried over magnesium sulfate. The mixture was filtered and the filtrate was concentrated under vacuum to give the crude product. The crude product was purified with silica gel column chromatography. When the desired product presents the same retention factor as dibenzylamine on TLC, the crude product was dissolved in 1.00 mL of chloroform, di-*tert*-butyl dicarbonate (105 mg, 0.48 mmol, 2.40 equiv.) were added and the mixture was stirred at 25 °C until the complete conversion of dibenzylamine (approximately 4 hours). The solution was then concentrated and purified on silica gel column to give the desired product.

General Procedure C: To an oven-dried 8.00 mL pressure vial equipped with a stirring bar, the heterocycle (0.20 mmol, 1.00 equiv.) and dichloromethane (1.00 mL) were charged under argon atmosphere. The mixture was cooled to –78 °C and trifluoromethanesulfonic anhydride (67.2 µL, 0.40 mmol, 2.00 equiv.) was added dropwise. The reaction was stirred for 30 minutes at -78 °C before a solution of dibenzylamine (188.0 µL, 0.96 mmol, 4.80 equiv.) and 2,4,6-collidine (52.6 µL, 0.40 mmol, 2.00 equiv.) in dichloromethane (0.25 mL) was added dropwise. The cooling bath was then removed and the reaction was allowed to warm to 25 °C. After 1 hour, ^{15}N-ammonium chloride (32.7 mg, 0.60 mmol, 3.00 equiv.), triethylamine (167.3 µL, 1.20 mmol, 6.00 equiv.) and acetonitrile (2.50 mL) were added. The reaction was stirred at 100 °C for 1 hour. After cooling to room temperature, the reaction was diluted with dichloromethane (5.00 mL). The mixture was then washed with saturated aqueous ammonium chloride (5.00 mL). The aqueous phase was then extracted with dichloromethane (3 x 5.00 mL). The combined organic phases were dried over magnesium sulfate. The mixture was filtered and the filtrate was concentrated under vacuum to give the crude product. The crude product was purified with silica gel column chromatography. When the desired product presents the same retention factor as dibenzylamine on TLC, the crude product was dissolved in 1.00 mL of chloroform, di-*tert*-butyl dicarbonate (210 mg, 0.96 mmol, 4.80 equiv.) were added and the mixture was stirred at 25 °C until the complete conversion of dibenzylamine (approximately 4 hours). The solution was then concentrated and purified on silica gel column to give the desired product.

General Procedure D: To an oven-dried 10.0 mL flask equipped with a stirring bar, the heterocycle (0.20 mmol, 1.00 equiv.) and ethyl acetate (1.00 mL) were charged under argon atmosphere. The mixture was cooled to –78 °C and trifluoromethanesulfonic anhydride (33.6 µL, 0.20 mmol, 1.00 equiv.) was added dropwise. The reaction was stirred for 30 minutes at -78 °C before a solution of dibenzylamine (47.0 µL, 0.24 mmol, 1.20 equiv.) and 2,4,6-collidine (26.3 µL, 0.20 mmol, 1.00 equiv.) in ethyl acetate (0.25 mL) was added dropwise. The cooling bath was then removed and the reaction was allowed to warm to 25 °C. After 1 hour, the reaction was diluted with ethyl acetate (1.0 mL) then washed with water (3 x 1.0 mL). The organic extract was dried over magnesium sulfate and filtered. After filtration, the organic extract was added dropwise to *n*-hexane (approximately 20 mL) and the resulting oil was allowed to settle overnight in a –20 °C freezer. The *n*-hexane was decanted off and the residual oil was washed with *n*-hexane to give the Zincke intermediate. The obtained intermediate was dried under vacuum and then used in the next step without further purification.
The obtained intermediate was dissolved in acetonitrile (2.00 mL) and transferred to an 8.00 mL pressure vial. ^{15}N-ammonium chloride (32.7 mg, 0.60 mmol, 3.00 equiv.), triethylamine (167.3 µL, 1.20 mmol, 6.00 equiv.) were added to the solution. The reaction was stirred at 100 °C for 1 hour. After cooling to room temperature, the reaction was diluted with dichloromethane (5.00 mL). The mixture

was then washed with saturated aqueous ammonium chloride (5.00 mL). The aqueous phase was then extracted with dichloromethane (3 x 5.00 mL). The combined organic phases were dried over magnesium sulfate. The mixture was filtered and the filtrate was concentrated under vacuum to give the crude product. The crude product was purified with silica gel column chromatography. When the desired product presents the same retention factor as dibenzylamine on TLC, the crude product was dissolved in 1.0 mL of chloroform, di-*tert*-butyl dicarbonate (105 mg, 0.48 mmol, 2.40 equiv.) was added and the mixture was stirred at 25 °C until the complete conversion of dibenzylamine (approximately 4 hours). The solution was then concentrated and purified on silica gel column to give the desired product.

5.2 General procedures of ^{15}N-labeling of pyridines with Zincke salts

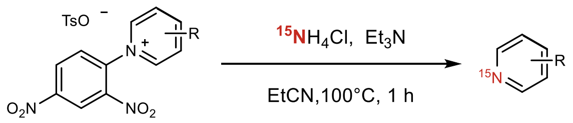

General procedure E: To a 8.00 mL pressure vial equipped with a stirring bar, triethylamine (83.6 µL, 0.60 mmol, 3.0 equiv.) was added to a suspension of ^{15}N-ammonium chloride (16.3 mg, 0.30 mmol, 1.50 equiv.) in proprionitrile (3.00 mL). The mixture was stirred 5 minutes at room temperature before the addition of the Zincke salt (0.20 mmol, 1.00 equiv.). The sealed vial was heated for 1 hour at 100 °C. After cooling to room temperature, the reaction was diluted with dichloromethane (5.00 mL). The mixture was then washed with saturated aqueous ammonium chloride (3 x 5.00 mL). The aqueous phase was then extracted with dichloromethane (3 x 5.00 mL). The combined organic phases were dried over magnesium sulfate. The mixture was filtered and the filtrate was concentrated under vacuum to give the crude product. The crude product was purified with silica gel column chromatography.

7. General procedures of nitrogen-carbon exchange of pyridines

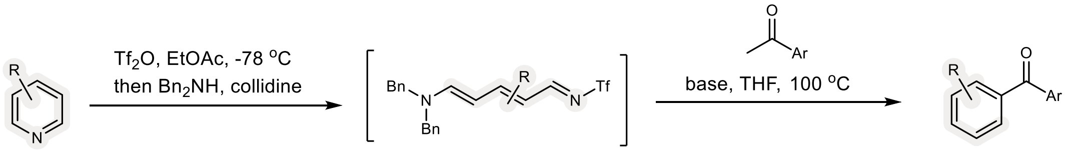

General Procedure F: To an oven-dried 10.0 mL flask equipped with a stirring bar, the heterocycle (0.20 mmol, 2.00 equiv.) and ethyl acetate (1.00 mL) were charged under argon atmosphere. The mixture was cooled to –78 °C and trifluoromethanesulfonic anhydride (33.6 µL, 0.20 mmol, 2.00 equiv.) was added dropwise. The reaction was stirred for 30 minutes at -78 °C before a solution of dibenzylamine (47.0 µL, 0.24 mmol, 2.40 equiv) and 2,4,6-collidine (26.3 µL, 0.20 mmol, 2.00 equiv) in ethyl acetate (0.25 mL) was added dropwise. The cooling bath was then removed and the reaction was allowed to warm to 25 °C. After 1 hour, the reaction was diluted with ethyl acetate (1.00 mL) then washed with water (3 x 1.00 mL). The organic extract was dried over magnesium sulfate and filtered. After filtration, the organic extract was added dropwise to *n*-hexane (approximately 20 mL) and the resulting oil was allowed to settle overnight in a –20 °C freezer. The *n*-hexane was decanted off and the residual oil was washed with *n*-hexane to give the Zincke intermediate. The obtained intermediate was dried under vacuum then used in the next step without further purification. The *N*Tf Zincke imine can be prepared in big scale and stored.

The obtained intermediate was dissolved in THF (1.00 mL) and transferred to an 8.00 mL pressure vial under argon. The ketone (0.10 mmol, 1.00 equiv.) was dissolved in 1.00 mL of THF under argon in a 5.00 mL flask and cooled to 0 °C. Sodium *tert*-butoxide (2.0 M in THF, 0.15 mL, 0.30 mmol, 1.50 equiv.) was added in dropwise. The solution was stirred at 0 °C for 10 minutes before adding to the Zincke imine solution in 1.0 mL THF in an 8.00 mL pressure vial. The reaction was stirred at 25 °C for 1 hour and then heated to 100 °C for 16 hours. After cooling to room temperature, the reaction was diluted with dichloromethane (5.00 mL). The mixture was then washed with saturated aqueous ammonium chloride (5.00 mL). The aqueous phase was then extracted with dichloromethane (3 x 5.00 mL). The combined organic phases were dried over magnesium sulfate. The mixture was filtered and the filtrate was concentrated under vacuum to give the crude product. The crude product was purified with silica gel column chromatography.

General Procedure G: To an oven-dried 10.0 mL flask equipped with a stirring bar, the heterocycle (0.20 mmol, 2.00 equiv.) and ethyl acetate (1.00 mL) were charged under argon atmosphere. The mixture was cooled to –78 °C and trifluoromethanesulfonic anhydride (33.6 µL, 0.20 mmol, 2.00 equiv.) was added dropwise. The reaction was stirred for 30 minutes at -78 °C before a solution of dibenzylamine (47.0 µL, 0.24 mmol, 2.40 equiv.) and 2,4,6-collidine (26.3 µL, 0.20 mmol, 2.00 equiv.) in ethyl acetate (0.25 mL) was added dropwise. The cooling bath was then removed and the reaction was allowed to warm to 25 °C. After 1 hour, the reaction was diluted with ethyl acetate (1.00 mL) then washed with water (3 x 1.00 mL). The organic extract was dried over magnesium sulfate and filtered. After filtration, the organic extract was added dropwise to *n*-hexane (approximately 20 mL) and the resulting oil was allowed to settle overnight in a –20 °C freezer. The *n*-hexane was decanted off and the residual oil was washed with *n*-hexane to give the Zincke intermediate. The obtained intermediate was dried under vacuum then used in the next step without further purification. The *N*Tf Zincke imine can be prepared in big scale and stored.

The obtained intermediate was dissolved in THF (1.00 mL) and transferred to an 8.00 mL pressure vial under argon. The ketone (0.10 mmol, 1.00 equiv.) was dissolved in 1.00 mL of THF under argon in a 5.00 mL flask and cooled to 0 °C. Sodium bis(trimethylsilyl)amide (1.0 M in THF, 0.30 mL, 0.30 mmol, 1.50 equiv.) was added in dropwise. The solution was stirred at 0 °C for 10 minutes before adding to

the Zincke imine solution in 1.00 mL THF in an 8.00 mL pressure vial under argon. The reaction was stirred at 25 °C for 1 hour and then heated to 100 ºC for 16 hours. After cooling to room temperature, the reaction was diluted with dichloromethane (5.00 mL). The mixture was then washed with saturated aqueous ammonium chloride (5.00 mL). The aqueous phase was then extracted with dichloromethane (3 x 5.00 mL). The combined organic phases were dried over magnesium sulfate. The mixture was filtered and the filtrate was concentrated under vacuum to give the crude product. The crude product was purified with silica gel column chromatography.

8. Characterizations of the prepared compounds

7.1 Characterizations of starting materials

1-(2,4-Dinitrophenyl)-3-phenylpyridin-1-ium 4-methylbenzenesulfonate (S1)

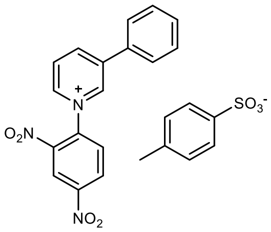

$C_{24}H_{19}N_3O_7S$
MW: 483.5 g.mol^{-1}
White powder

The title compound was obtained in 64% yield (309.4 mg, 0.64 mmol) using General Procedure P.

^{1}H NMR (400 MHz, DMSO-d$_6$) δ 9.82 (s, 1H), 9.36 (d, *J* = 5.9 Hz, 1H), 9.28 (d, *J* = 8.1 Hz, 1H), 9.14 (d, *J* = 2.1 Hz, 1H), 8.99 (dd, *J* = 8.6, 2.2 Hz, 1H), 8.50 (m, 2H), 7.96 (d, *J* = 6.6 Hz, 2H), 7.63 (d, *J* = 7.5 Hz, 3H), 7.44 (d, *J* = 7.9 Hz, 2H), 7.10 (d, *J* = 7.7 Hz, 2H), 2.28 (s, 3H).

The spectroscopic data are in accordance with literature.[1]

1-(2,4-Dinitrophenyl)-3-(1,3-dioxoisoindolin-2-yl)pyridin-1-ium 4-methylbenzenesulfonate (S2)

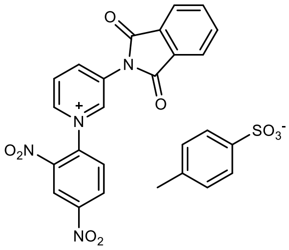

$C_{26}H_{18}N_4O_9S$
MW: 562.5 g.mol^{-1}
White powder

The title compound was obtained in 70% yield (393.8 mg, 0.70 mmol) using General Procedure P.

^{1}H NMR (400 MHz, DMSO-d$_6$) δ 9.74 (s, 1H), 9.47 (d, *J* = 6.1 Hz, 1H), 9.12 (d, *J* = 2.3 Hz, 1H), 9.10 (d, *J* = 8.7 Hz, 1H), 8.99 (dd, *J* = 8.7, 2.3 Hz, 1H), 8.64 (dd, *J* = 8.3, 6.3 Hz, 1H), 8.51 (d, *J* = 8.7 Hz, 1H), 8.11 (dd, *J* = 5.3, 3.0 Hz, 2H), 8.01 (dd, *J* = 5.2, 3.1 Hz, 2H), 7.45 (d, *J* = 7.9 Hz, 2H), 7.10 (d, *J* = 7.8 Hz, 2H), 2.28 (s, 3H).

^{13}C NMR (100 MHz, DMSO-d$_6$) δ 165.3 (2C), 149.2, 145.7, 145.1, 144.8, 143.1, 142.9, 138.4, 137.5, 135.6 (2C), 132.0, 131.7, 131.1 (2C), 130.3, 128.4, 128.0 (2C), 125.4 (2C), 124.2 (2C), 121.5, 20.7.

IR (cm^{-1}) 3126, 3049, 1732, 1606, 1557, 1503, 1367, 1349, 1192, 1034, 903, 810, 716, 674, 559.

HRMS (ESI-TOF) *m/z* [M-TsO$^-$] Calcd for $C_{19}H_{11}N_4O_6$ 391.0679; Found 391.0677.

3-Carbamoyl-1-(2,4-dinitrophenyl)pyridin-1-ium 4-methylbenzenesulfonate (S3)

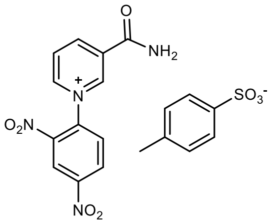

$C_{19}H_{16}N_4O_8S$
MW: 460.4 g.mol^{-1}
White powder

The title compound was obtained in 89% yield (409.8 mg, 0.89 mmol) using General Procedure P.

^{1}H NMR (400 MHz, DMSO-d$_6$) δ 9.79 (s, 1H), 9.50 (d, *J* = 6.1 Hz, 1H), 9.26 (d, *J* = 8.2 Hz, 1H), 9.10 (d, *J* = 2.4 Hz, 1H), 8.96 (dd, *J* = 8.7, 2.5 Hz, 1H), 8.66 (s, 1H), 8.54 (dd, *J* = 8.1, 6.3 Hz, 1H), 8.41 (d, *J* = 8.7 Hz, 1H), 8.28 (s, 1H), 7.44 (d, *J* = 8.0 Hz, 2H), 7.10 (d, *J* = 7.8 Hz, 2H), 2.28 (s, 3H).

^{13}C NMR (100 MHz, DMSO-d$_6$) δ 162.3, 149.2, 147.7, 146.4 (2C), 145.6, 142.9, 138.5, 137.6, 133.5, 131.9, 130.2, 128.0 (2C), 127.7, 125.4 (2C), 121.3, 20.7.

IR (cm^{-1}) 3425, 3170, 2942, 1716, 1617, 1542, 1345, 1212, 1187, 1033,819, 677, 566, 516.

HRMS (ESI-TOF) *m/z* [M-TsO$^-$] Calcd for $C_{12}H_9N_4O_5$ 289.0573; Found 289.0571.

1-(2,4-Dinitrophenyl)-3-(methoxycarbonyl)pyridin-1-ium 4-methylbenzenesulfonate (S4)

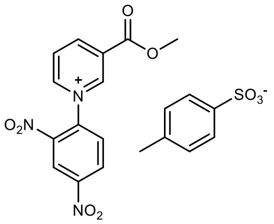

$C_{20}H_{17}N_3O_9S$
MW: 475.4 g.mol^{-1}
White powder

The title compound was obtained in 67% yield (318,5 mg, 0.67 mmol) using General Procedure P.

^{1}H NMR (400 MHz, DMSO-d$_6$) δ 10.00 (s, 1H), 9.60 (d, *J* = 6.1 Hz, 1H), 9.32 (d, *J* = 8.2 Hz, 1H), 9.10 (d, *J* = 2.3 Hz, 1H), 8.96 (dd, *J* = 8.7, 2.4 Hz, 1H), 8.68 – 8.51 (m, 1H), 8.40 (d, *J* = 8.7 Hz, 1H), 7.43 (d, *J* = 7.9 Hz, 2H), 7.09 (d, *J* = 7.8 Hz, 2H), 4.00 (s, 3H), 2.29 (s, 3H).

The spectroscopic data are in accordance with literature.[1]

7.2 Characterizations of ^{15}*N*-labeled pyridines

2-Phenylpyridine-1-^{15}N ([^{15}N]1)

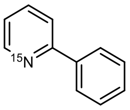

$C_{11}H_9{}^{15}N$
MW: 156.2 g.mol^{-1}
Colorless oil

The title compound was obtained in 82% (25.6 mg, 0.16 mmol) ± 3.5% yield using General Procedure A with 76) ± 0.2% IE (n=2). While General Procedure D was used, the title compound was obtained in 64% overall yield (85% for the Zincke imine preparation and 75% for the ^{15}*N*-labeling) (20.4 mg, 0.13 mmol) with 97% IE. The crude product was purified with silica gel column chromatography (heptane/ethyl acetate = 5:1).

^{1}H NMR (400 MHz, CDCl$_3$) δ 8.70 (dddd, $J_{N\text{-}H}$ = 11.2 Hz, J = 4.8, 1.8, 1.0 Hz, 1H), 8.05 – 7.96 (m, 2H), 7.75 (ddd, J = 8.0, 7.3, 1.0 Hz, 1H), 7.69 (ddd, J = 8.0, 1.3, 1.0 Hz, 1H), 7.54 – 7.45 (m, 2H), 7.45 – 7.38 (m, 1H), 7.23 (ddd, J = 7.3, 4.8, 1.3 Hz, 1H).

^{13}C NMR (100 MHz, CDCl$_3$) δ 149.6, 136.8, 136.8, 128.9 (2C), 128.7 (2C), 126.9, 126.9, 122.1, 120.6.

^{15}N NMR (41 MHz, CDCl$_3$) δ 306.0 (s, 1N).

IR (cm^{-1}) 2924, 1589, 1466, 1448, 1423, 1152, 745, 693.

HRMS (ESI-TOF) m/z calcd for $C_{11}H_{10}N$ $[M+H]^+$ 156.0813; found 156.0811. (unlabeled coimpound)

Isotopic enrichment (^{15}N): 75.8% measured by HRMS. (Procedure A)

Isotopic enrichment (^{15}N): 97.3% measured by HRMS. (Procedure D)

2-(4-Bromophenyl)pyridine-1-^{15}N ([^{15}N]2)

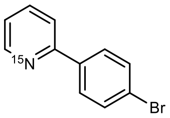

$C_{11}H_8Br^{15}N$
MW: 235.1 g.mol^{-1}
White solid

The title compound was obtained in 83% (39.0 mg, 0.17 mmol) yield using General Procedure A with 74% IE. The crude product was purified with silica gel column chromatography (heptane/ethyl acetate = 5:1).

^{1}H NMR (400 MHz, CDCl$_3$) δ 8.68 (dddd, $J_{N\text{-}H}$ = 11.2 Hz, J = 4.8, 1.8, 1.0 Hz, 1H), 7.89 (ddd, J = 8.2, 2.5, 0.3 Hz, 1H), 7.87 (ddd, J = 8.2, 2.5, 0.3 Hz, 1H), 7.75 (ddd, J = 8.0, 7.3, 1.0 Hz, 1H), 7.69 (ddd, J = 8.0, 1.3, 1.0 Hz, 1H), 7.61 (ddd, J = 8.2, 2.0, 0.3 Hz, 1H), 7.59 (ddd, J = 8.2, 2.0, 0.3 Hz, 1H), 7.25 (ddd, J = 7.3, 4.8, 1.3 Hz, 1H).

^{13}C NMR (100 MHz, CDCl$_3$) δ 156.2, 149.7, 138.2 (s+d, J = 7.8 Hz), 136.8 (s+d, J = 2.9 Hz), 131.9 (2C), 128.4 (2C, s+d, J = 2.9 Hz), 123.4, 122.4 (s+d, J = 2.3 Hz), 120.3 (s+d, J = 2.2 Hz).

^{15}N NMR (41 MHz, CDCl$_3$) δ 306.0 (s, 1N).

IR (cm^{-1}) 1591, 1461, 1430, 1393, 1153, 1095, 1070, 1007, 907, 839, 772, 761, 733.

HRMS (ESI-TOF) m/z calcd for $C_{11}H_9BrN$ [M+H]$^+$ 233.9918; found 233.9915. (unlabeled coimpound)

Isotopic enrichment (^{15}N): 73.5% measured by HRMS.

Isotopic enrichment (^{15}N): 73.5% measured by HRMS.

2-(4-Ttrifluoromethyl)phenyl)pyridine-1-^{15}N ([^{15}N]3)

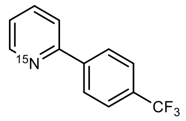

$C_{12}H_8F_3{}^{15}N$
MW: 224.2 g.mol^{-1}
Light yellow liquid

The title compound was obtained in 81% (36.3 mg, 0.16 mmol) yield using General Procedure A with 69% IE. The crude product was purified with silica gel column chromatography (heptane/ethyl acetate = 5:1).

^{1}H NMR (400 MHz, CDCl$_3$) δ 8.73 (dddd, $J_{N\text{-}H}$ = 11.2 Hz, J = 4.8, 1.8, 1.0 Hz, 1H), 8.15 – 8.08 (m, 2H), 7.80 (ddd, J = 8.0, 7.3, 1.0 Hz, 1H), 7.77 (ddd, J = 8.0, 1.3, 1.0 Hz, 1H), 7.76 – 7.70 (m, 2H), 7.30 (ddd, J = 7.3, 4.8, 1.3 Hz, 1H).

^{13}C NMR (100 MHz, CDCl$_3$) δ 156.0, 150.1, 142.8 (m), 137.1 (s+d, J = 2.2 Hz), 131.1 (q, J = 33.1 Hz), 127.3 (m, 2C), 125.8 (q, J = 3.8 Hz, 2C), 123.3, 123.0 (q, J = 271.9 Hz), 120.3.

^{15}N NMR (41 MHz, CDCl$_3$) δ 308.1 (s, 1N).

^{19}F NMR (376 MHz, CDCl$_3$) δ -62.6 (s).

IR (cm^{-1}) 3054, 2925, 1616, 1580, 1465, 1435, 1405, 1326, 1264, 1166, 1108, 1071, 782, 733, 704.

HRMS (ESI-TOF) m/z calcd for $C_{12}H_9F_3N$ [M+H]$^+$ 224.0687; found 224.0689. (unlabeled coimpound)

Isotopic enrichment (^{15}N): 68.8% measured by HRMS.

2-(2,4-Difluorophenyl)pyridine-1-^{15}N ([^{15}N]4)

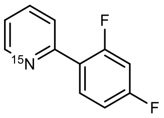

$C_{11}H_7F_2{}^{15}N$
MW: 192.2 g.mol^{-1}
Light yellow liquid

The title compound was obtained in 71% (27.3 mg, 0.14 mmol) yield using General Procedure A with 56% IE. The crude product was purified with silica gel column chromatography (heptane/ethyl acetate = 5:1).

^{1}H NMR (400 MHz, CDCl$_3$) δ 8.63 (dddd, $J_{N\text{-}H}$ = 11.2 Hz, J = 4.8, 1.8, 1.0 Hz, 1H), 7.92 (m, 1H), 7.74 (d, J = 3.8 Hz, 2H), 7.29 – 7.20 (m, 1H), 7.04 – 6.95 (m, 1H), 6.90 (m, 1H).

^{13}C NMR (100 MHz, CDCl$_3$) δ 165.4 – 158.8 (m, 2C), 152.7 (s+d, J = 2.5 Hz), 149.9 (s+d, J = 1.4 Hz), 136.6, 132.4 – 132.1 (m), 124.3 (d, J = 9.8 Hz), 124.0 – 123.8 (m), 122.6, 112.0 (dd, J = 21.1, 3.7 Hz), 104.5 (dd, J = 27.0, 25.4 Hz).

^{15}N NMR (41 MHz, CDCl$_3$) δ 310.3 (s, 1N).

^{19}F NMR (376 MHz, CDCl$_3$) δ -109.3 (d, J = 8.5 Hz), -113.0 (d, J = 8.5 Hz).

IR (cm^{-1}) 3056, 3010, 2925, 1598, 1504, 1462, 1438, 1416, 1261, 1138, 1104, 965, 848, 778, 742, 561.

HRMS (ESI-TOF) m/z calcd for $C_{11}H_8F_2N$ [M+H]$^+$ 192.0625; found 192.0626. (unlabeled compound)

Isotopic enrichment (^{15}N): 55.7% measured by HRMS.

2-(Thiophen-2-yl)pyridine-1-^{15}N ([^{15}N]5)

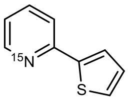

$C_9H_7{}^{15}NS$
MW: 162.2 g.mol^{-1}
Yellow foam

The title compound was obtained in 95% (30.8 mg, 0.19 mmol) yield using General Procedure A with 50% IE. The crude product was purified with silica gel column chromatography (heptane/ethyl acetate = 5:1).

^{1}H NMR (400 MHz, CDCl$_3$) δ 8.57 (dddd, $J_{N\text{-}H}$ = 11.2 Hz, J = 4.8, 1.8, 1.0 Hz, 1H), 7.68 (ddd, J = 8.0, 7.3, 1.0 Hz, 1H), 7.65 (ddd, J = 8.0, 1.3, 1.0 Hz, 1H), 7.58 (dd, J = 3.7, 1.1 Hz, 1H), 7.39 (dd, J = 5.1, 1.1 Hz, 1H), 7.13 (ddd, J = 7.3, 4.8, 1.3 Hz, 1H), 7.58 (dd, J = 5.1, 3.7 Hz, 1H).

^{13}C NMR (101 MHz, CDCl$_3$) δ 152.7, 149.5, 144.8 (s+d, J = 9.3 Hz), 136.6, 128.0, 127.5, 124.5, 121.8, 118.8.

^{15}N NMR (41 MHz, CDCl$_3$) δ 301.9 (s, 1N).

IR (cm^{-1}) 1581, 1560. 1533, 1463, 1435, 1420, 1290, 1221, 1152, 1057, 984, 852, 772, 736, 702, 612.

HRMS (ESI-TOF) m/z calcd for C_9H_8NS $[M+H]^+$ 162.0378; found 162.0377. (unlabeled compound)

Isotopic enrichment (^{15}N): 50.4% measured by HRMS.

2-(Pyridin-2-yl-^{15}N)benzo[d]oxazole ([^{15}N]6)

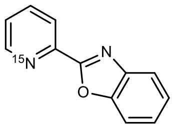

$C_{12}H_8N^{15}NO$
MW: 197.2 g.mol^{-1}
White solid

The title compound was obtained in 70% (27.6 mg, 0.14 mmol) yield using General Procedure A with 39% IE. The crude product was purified with silica gel column chromatography (heptane/ethyl acetate = 2:1).

^{1}H NMR (400 MHz, CDCl$_3$) δ 8.81 (dddd, $J_{N\text{-}H}$ = 11.2 Hz, J = 4.8, 1.8, 1.0 Hz, 1H), 8.35 (td, J = 7.9, 1.1c Hz, 1H), 7.88 (td, J = 7.8, 1.7 Hz, 1H), 7.84 – 7.79 (m, 1H), 7.69 – 7.62 (m, 1H), 7.44 (ddd, J = 7.3, 4.8, 1.3 Hz, 1H), 7.41 – 7.35 (m, 2H).

^{13}C NMR (100 MHz, CDCl$_3$) δ 161.6, 151.2, 150.4, 146.2, 141.9, 137.2, 126.2, 125.7, 125.0, 123.6, 120.7, 111.3.

^{15}N NMR (41 MHz, CDCl$_3$) δ 307.0 (s, 1N).

IR (cm^{-1}) 3408, 3091, 03059, 2924, 1584, 1552, 1451, 1437, 1348, 1242, 1077, 1090, 935, 810, 797, 739, 703, 528.

HRMS (ESI-TOF) m/z calcd for $C_{12}H_9N_2O$ $[M+H]^+$ 197.0715; found 197.0713. (unlabeled coimpound)

Isotopic enrichment (^{15}N): 39.0% measured by HRMS.

2-(3-Phenylpropyl)pyridine-1-^{15}N ([^{15}N]7)

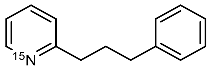

$C_{14}H_{15}{}^{15}N$
MW: 198.27 g.mol^{-1}
Yellow liquid

The title compound was obtained in 84% yield (33.4 mg, 0.17 mmol) using General Procedure A with 76% IE. The crude product was purified with silica gel column chromatography (heptane/ethyl acetate = 10:1).

^{1}**H NMR (400 MHz, CDCl$_3$)** δ 8.52 (dddd, J_{N-H} = 11.2 Hz, J = 4.8, 1.8, 1.0 Hz, 1H), 7.58 (td, J = 7.7, 1.8 Hz, 1H), 7.32 – 7.23 (m, 2H), 7.23 – 7.15 (m, 3H), 7.15 – 7.06 (m, 2H), 2.87 – 2.79 (m, 2H), 2.68 (t, J = 7.7 Hz, 2H), 2.12 – 2.02 (m, 2H).

^{13}**C NMR (100 MHz, CDCl$_3$)** δ 162.0 (s+d, J = 2.1 Hz), 149.3, 142.2, 136.4, 128.6 (2C), 128.4 (2C), 125.9, 122.9 (s+d, J = 2.3 Hz), 121.1 (s+d, J = 2.3 Hz), 37.9, 35.7, 31.6.

^{15}**N NMR (41 MHz, CDCl$_3$)** δ 309.4 (s, 1N).

IR (cm^{-1}) 3061, 3025, 2925, 2857, 1589, 1564, 1496, 1470, 1431, 1881, 1230, 1187, 1151, 1048, 1029, 979, 747, 699, 604, 494.

HRMS (ESI-TOF) *m/z* calcd for $C_{14}H_{16}N$ [M+H]+ 198,1283; found 198,1285.

Isotopic enrichment : 76,2% measured by HRMS.

Ethyl picolinate-^{15}N ([^{15}N]8)

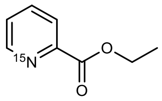

$C_8H_9{}^{15}NO_2$
MW: 152.2 g.mol^{-1}
Light yellow liquid

The title compound was obtained in 45% yield (13.7 mg, 0.09 mmol) using General Procedure A with 37% IE. The crude product was purified with silica gel column chromatography (heptane/ethyl acetate = 5:1).

^{1}**H NMR (400 MHz, CDCl$_3$)** δ 8.79 – 8.72 (dddd, J_{N-H} = 11.2 Hz, J = 4.8, 1.8, 1.0 Hz, 1H), 8.13 (ddd, J = 8.0, 1.3, 1.0 Hz, 1H), 7.84 (ddd, J = 8.0, 1.3, 1.0 Hz, 1H), 7.47 (ddd, J = 7.3, 4.8, 1.3 Hz, 1H), 4.48 (q, J = 7.1 Hz, 2H), 1.44 (t, J = 7.1 Hz, 3H).

^{1}**H NMR (400 MHz, CDCl$_3$)** δ 8.76 (m, 1H), 8.13 (d, J = 7.9 Hz, 1H), 7.84 (td, J = 7.7, 1.8 Hz, 1H), 7.50 – 7.43 (m, 1H), 4.48 (q, J = 7.1 Hz, 2H), 1.44 (t, J = 7.1 Hz, 3H).

^{13}**C NMR (100 MHz, CDCl$_3$)** δ 165.4, 150.0, 148.4, 137.1, 127.0, 125.2, 62.1, 14.5.

^{15}**N NMR (41 MHz, CDCl$_3$)** δ 311.7 (s, 1N).

IR (cm^{-1}) 3058, 2982, 2930, 1737, 1715, 1583, 1465, 1437, 1368, 1304, 1244, 1126, 1087, 1044, 1020, 994, 745, 705, 619.

HRMS (ESI-TOF) m/z calcd for $C_8H_{10}NO_2$ [M+H]$^+$ 152.0712; found 152.071.

Isotopic enrichment (^{15}N): 36.7% measured by HRMS.

4-Phenylpyridine-1-^{15}N ([^{15}N]9)

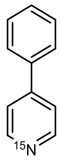

$C_{11}H_9{}^{15}N$
MW: 156.2 g.mol^{-1}
Yellow solid

The title compound was obtained in 85% (26.6 mg, 0.17 mmol) yield using General Procedure A with 70% IE. While General Procedure D was used, the title compound was obtained in 67% (20.9 mg, 0.13 mmol) (70% for the Zincke imine preparation and 95% for the ^{15}*N*-labeling) combined yield with >99% IE. The crude product was purified with silica gel column chromatography (dichloromethane/ethyl acetate = 2:1).

^{1}H NMR (400 MHz, CDCl$_3$) δ 8.66 (ddd, $J_{N\text{-}H}$ = 10.6 Hz, J = 4.5, 1.6 Hz, 2H), 7.70 – 7.60 (m, 2H), 7.54 – 7.39 (m, 5H).

^{13}C NMR (100 MHz, CDCl$_3$) δ 150.2, 148.3 (s+d, J = 3.6 Hz, 2C), 138.1, 129.1 (2C), 129.0, 127.0 (2C), 121.6 (2C).

^{15}N NMR (41 MHz, CDCl$_3$) δ 306.3 (s, 1N).

IR (cm^{-1}) 2923, 1591, 1479, 1402, 1043, 828, 755, 725, 687, 605.

HRMS (ESI-TOF) m/z calcd for $C_{11}H_{10}{}^{15}N$ [M+H]$^+$ 157.0784; found 157.0782. (labeled compound)

Isotopic enrichment (^{15}N): 70.0% measured by HRMS. (Procedure A)

Isotopic enrichment (^{15}N): 99.6% measured by HRMS. (Procedure D)

4-(4-Phenoxyphenyl)-^{15}N-pyridine ([^{15}N]10)

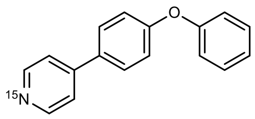

$C_{17}H_{13}{}^{15}NO$
MW: 248.3 g.mol^{-1}
White solid

The title compound was obtained in 60% yield (29.8 mg, 0.12 mmol) using General Procedure A with 74% IE. The crude product was purified with silica gel column chromatography (heptan/ethyl acetate = 2:1)

^{1}H NMR (400 MHz, CDCl$_3$) δ 8.64 (ddd, $J_{N\text{-}H}$ = 10.6 Hz, J = 4.5, 1.6 Hz, 2H), 7.69 – 7.54 (m, 2H), 7.48 (d, J = 5.0 Hz, 2H), 7.42 – 7.32 (m, 2H), 7.16 (dt, J = 8.5, 1.1 Hz, 1H), 7.11 – 7.02 (m, 4H).

^{13}C NMR (100 MHz, CDCl$_3$) δ 158.6, 156.5, 150.2 (2C), 147.6 (s+d, J = 2.1 Hz), 132.7, 129.9 (2C), 128.4 (2C), 123.9 (2C), 121.3, 119.5 (2C), 118.9 (2C).

^{15}**N NMR (41 MHz, CDCl$_3$)** δ 304.6 (s, 1N).

IR (cm^{-1}) 3036, 1629, 1589, 1512, 1484, 1402, 1330, 1276, 1241, 1224, 1200, 1171, 1071, 1023, 871, 815, 761, 710, 692,576, 491, 458, 442, 424, 405.

HRMS (ESI-TOF) m/z calcd for $C_{17}H_{14}NO$ [M+H]$^+$ 248.1075; found 248.1077. (unlabeled compound)

Isotopic enrichment (^{15}N): 74.2% measured by HRMS.

5-(Pyridin-4-yl-^{15}N)thiophene-2-carbaldehyde ([^{15}N]11)

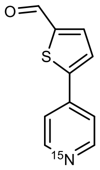

$C_{10}H_7{}^{15}NOS$
MW: 190.2 g.mol^{-1}
Yellow solid

The title compound was obtained in 50% yield (19.0 mg, 0.10 mmol) using General Procedure A with 74% IE. The crude product was purified with silica gel column chromatography (heptane/ethyl acetate = 2:1)

^{1}**H NMR (400 MHz, CDCl$_3$)** δ 9.93 (s, 1H), 8.67 (ddd, $J_{N\text{-}H}$ = 10.6 Hz, J = 4.5, 1.6 Hz, 2H), 7.78 (d, J = 4.0 Hz, 1H), 7.57 (d, J = 4.0 Hz, 1H), 7.52 (dd, J = 4.5, 1.6 Hz, 2H).

^{13}**C NMR (100 MHz, CDCl$_3$)** δ 182.8, 150.8, 150.2, 144.3, 140.1 (s+d, J = 3.7 Hz, 2C), 136.9, 126.1, 120.3 (s+d, J = 2.3 Hz, 2C).

^{15}**N NMR (41 MHz, CDCl$_3$)** δ 313.3 (s, 1N).

IR (cm^{-1}) 1662, 1596, 1454, 1413, 1221, 1050, 972, 802, 704, 460, 412.

HRMS (ESI-TOF) m/z calcd for $C_{10}H_8NOS$ [M+H]$^+$ 190.0327; found 190.0327. (unlabeled compound)

Isotopic enrichment (^{15}N): 74.3% measured by HRMS.

4-(Pentan-3-yl)pyridine-1-^{15}N ([^{15}N]12)

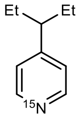

$C_{10}H_{15}{}^{15}N$
MW: 150.2 g.mol^{-1}
Colorless oil

The title compound was obtained in 55% yield (16.5 mg, 0.11 mmol) using General Procedure B with 80% IE. Due to the volatility of the title compound, the isolated yield is lower than the NMR yield (95%). The crude product was purified with silica gel column chromatography (heptane/ethyl acetate = 2:1).

^{1}H NMR (400 MHz, CDCl$_3$) δ 8.42 (ddd, $J_{N\text{-}H}$ = 10.6 Hz, J = 4.5, 1.6 Hz, 2H), 7.00 (dd, J = 4.5, 1.6 Hz, 2H), 2.31 – 2.20 (m, 1H), 1.68 – 1.44 (m, 4H), 0.70 (t, J = 7.4 Hz, 6H).

^{13}C NMR (100 MHz, CDCl$_3$) δ 155.0, 149.6 (2C), 123.4 (2C), 49.2, 28.6 (2C), 12.0 (2C).

^{15}N NMR (41 MHz, CDCl$_3$) δ 304.1 (s, 1N).

IR (cm^{-1}) 1459, 1415, 1377, 1274, 793, 731, 503, 432, 410.

HRMS (ESI-TOF) m/z calcd for $C_{10}H_{16}N$ [M+H]$^+$ 150.1283; found 150.1282. (unlabeled compound)

Isotopic enrichment (^{15}N): 79.8% measured by HRMS.

4-(Benzyloxy)pyridine-1-^{15}N ([^{15}N]13)

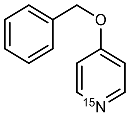

$C_{12}H_{11}{}^{15}NO$
MW: 186.2 g.mol^{-1}
White solid

The title compound was obtained in 66% yield (24.6 mg, 0.13 mmol) using General Procedure B with 59% IE. The crude product was purified with silica gel column chromatography (heptane/ethyl acetate = 2:1).

^{1}H NMR (400 MHz, CDCl$_3$) δ 8.44 (ddd, $J_{N\text{-}H}$ = 10.6 Hz, J = 4.5, 1.6 Hz, 2H), 7.44 – 7.32 (m, 5H), 6.91 – 6.83 (m, 2H), 5.11 (s, 2H).

^{13}C NMR (100 MHz, CDCl$_3$) δ 164.8, 151.3 (2C), 135.8, 128.9 (2C), 128.5 (2C), 127.7 (2C), 110.7, 69.9.

^{15}N NMR (41 MHz, CDCl$_3$) δ 289.6 (s, 1N).

IR (cm^{-1}) 3660, 3030, 2922, 1586, 1565, 1497, 1454, 1417, 1381, 1276, 1208, 1001, 861, 814, 736, 696, 534.

HRMS (ESI-TOF) *m/z* calcd for $C_{12}H_{12}NO$ [M+H]$^+$ 186,0919; found 186,0917. (unlabeled compound)

Isotopic enrichment (^{15}N): 59.1% measured by HRMS.

Tert-butyl 2-(pyridin-3-yl-^{15}N)-1H-pyrrole-1-carboxylate ([^{15}N]22)

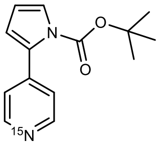

$C_{14}H_{16}{}^{15}NO_2$
MW: 245.29 g.mol^{-1}
Yellowish liquid

The title compound was obtained in 75% yield (37.0 mg, 0.15 mmol) using General Procedure A with 66% IE. The crude product was purified with silica gel column chromatography (heptane/ethyl acetate = 2:1).

^{1}H NMR (400 MHz, CDCl$_3$) δ 8.89 – 8.81 (ddd, $J_{N\text{-}H}$ = 10.6 Hz, J = 4.5, 1.6 Hz, 2H), 7.68 (dd, J = 3.3, 1.8 Hz, 1H), 7.56 – 7.55 (m, 1H), 7.55 – 7.53 (m, 1H), 6.60 (dd, J = 3.3, 1.8 Hz, 1H), 6.54 (t, J = 3.3 Hz, 1H), 1.69 (s, 9H).

^{13}C NMR (100 MHz, CDCl$_3$) δ 149.3, 149.2, 149.1, 142.0 (s+d, J = 2.4 Hz), 132.4, 124.4, 123.7, 120.6, 116.4, 111.2, 84.6, 27.8 (3C).

^{15}N NMR (41 MHz, CDCl$_3$) δ 306.0 (s, 1N).

IR (cm^{-1}) 2979, 2932, 1740, 1600, 1459, 1395, 1370, 1341, 1308, 1256, 1143, 1077, 1044, 982, 843, 813, 732.

LCMS (ESI) *m/z* [M+H]$^+$ 194.

HRMS (ESI-TOF) *m/z* calcd for $C_{14}H_{17}N_2O_2$ [M+H]$^+$ 245,129; found 245,1293. (unlabeled compound)

Isotopic enrichment : 66,4% measured by HRMS.

5-(Pyridin-4-yl-^{15}N)thiophene-2-carbaldehyde ([^{15}N]14)

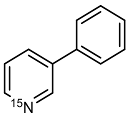

$C_{11}H_9{}^{15}N$
MW: 156.2 g.mol^{-1}
Colorless oil

The title compound was obtained in 94% yield (29.4 mg, 0.19 mmol) using General Procedure A with 73% IE. While general procedure E was used, the title compound was obtained in 75% yield (23.4 mg, 0.15 mmol) with 85% IE. The crude product was purified with silica gel column chromatography (heptane/ethyl acetate = 2:1).

^{1}H NMR (400 MHz, CDCl$_3$) δ 8.84 (ddd, $J_{N\text{-}H}$ = 10.4 Hz, J = 2.2, 0.9 Hz, 1H), 8.59 (ddd, $J_{N\text{-}H}$ = 10.8 Hz, J = 4.8, 1.8 Hz, 1H), 7.88 (ddd, J = 7.9, 2.3, 1.7 Hz, 1H), 7.61 – 7.55 (m, 2H), 7.52 – 7.45 (m, 2H), 7.44 – 7.34 (m, 2H).

^{13}C NMR (100 MHz, CDCl$_3$) δ 148.3 (d, J = 1.6 Hz), 148.2 (d, J = 1.6 Hz), 137.8, 136.7, 134.5 (s+d, J = 2.1 Hz), 129.1 (2C), 128.1, 127.2 (2C), 123.6.

^{15}N NMR (41 MHz, CDCl$_3$) δ 310.6 (s, 1N).

IR (cm^{-1}) 1472, 1450, 1308, 1231, 1187, 1005, 812, 753, 697, 638, 605, 550.

HRMS (ESI-TOF) m/z calcd for $C_{11}H_{10}N$ [M+H]$^+$ 156.0813; found 156.0818. (unlabeled compound)

Isotopic enrichment (^{15}N): 72.8% measured by HRMS. (Procedure A)

Isotopic enrichment (^{15}N): 85% measured by HRMS. (Procedure E)

Ethyl nicotinate-^{15}N ([^{15}N]15)

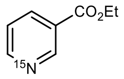

$C_8H_9{}^{15}NO_2$
MW: 152.2 g.mol^{-1}
Colorless oil

The title compound was obtained in 88% yield (26.8 mg, 0.18 mmol) using General Procedure A with 77% IE. The crude product was purified with silica gel column chromatography (heptane/ethyl acetate = 2:1).

^{1}H NMR (400 MHz, CDCl$_3$) δ 9.21 (ddd, $J_{N\text{-}H}$ = 10.7 Hz, J = 2.2, 0.9 Hz, 1H), 8.75 (ddd, $J_{N\text{-}H}$ = 10.8 Hz, J = 4.8, 1.8 Hz, 1H), 8.29 (ddd, J = 8.0, 2.4, 1.6 Hz, 1H), 7.38 (dddd, J = 8.0, 4.8, 1.6, 0.9, 1H), 4.40 (q, J = 7.1 Hz, 2H), 1.40 (t, J = 7.1 Hz, 3H).

^{13}C NMR (101 MHz, CDCl$_3$) δ 165.2, 153.2, 150.8, 137.0 (s+d, J = 3.2 Hz), 126.3 (s+d, J = 2.6 Hz), 123.2 (s+d, J = 2.4 Hz), 61.4, 14.2.

^{15}N NMR (41 MHz, CDCl$_3$) δ 311.3 (s, 1N).

IR (cm^{-1}) 1721, 1588, 1369, 1282, 1233, 1190, 1024, 741, 699, 611.

HRMS (ESI-TOF) m/z calcd for $C_8H_{10}NO_2$ [M+H]$^+$ 152.0712; found 152.0715. (unlabeled compound)

Isotopic enrichment (^{15}N): 77.4% measured by HRMS.

1-(Pyridin-3-yl-^{15}N)propan-1-one ([^{15}N]16)

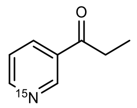

$C_8H_9{}^{15}NO$
MW: 136.2 g.mol^{-1}
Colorless liquid

The title compound was obtained in 68% yield (18.8 mg, 0.14 mmol) using General Procedure A with 49% IE. The crude product was purified with silica gel column chromatography (heptane/ethyl acetate = 2:1).

^{1}H NMR (400 MHz, $CDCl_3$) δ 9.18 (ddd, $J_{N\text{-}H}$ = 10.8 Hz, J = 2.2, 0.9 Hz, 1H), 8.77 (ddd, $J_{N\text{-}H}$ = 10.8 Hz, J = 4.8, 1.8 Hz, 1H), 8.24 (ddd, J = 8.0, 2.2, 1.8 Hz, 1H), 7.41 (ddd, J = 8.0, 4.8, 0.9, 1H), 3.03 (q, J = 7.2 Hz, 2H), 1.25 (t, J = 7.2 Hz, 3H).

^{13}C NMR (100 MHz, CD_2Cl_2) δ 200.1, 153.9, 150.0, 135.6, 128.9 (s+d, J = 2.3 Hz), 124.1, 32.7, 8.2.

IR (cm^{-1}) 2979, 2939, 1690, 1585, 1459, 1416, 1356, 1228, 1016, 949, 786, 702.

HRMS (ESI-TOF) m/z calcd for $C_8H_{10}NO$ [M+H]$^+$ 136.0762; found 136.0758. (unlabeled compound)

Isotopic enrichment (^{15}N): 48.9% measured by HRMS.

N,N-Dimethylpyridine-3-sulfonamide-1-^{15}N ([^{15}N]17)

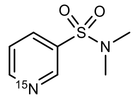

$C_7H_{10}N^{15}NO_2S$
MW: 187.2 g.mol^{-1}
Orange solid

The title compound was obtained in 81% yield (30.3 mg, 0.16 mmol) using General Procedure A with 87% IE. The crude product was purified with silica gel column chromatography (heptane/ethyl acetate = 2:1).

^{1}H NMR (400 MHz, CD_2Cl_2) δ 8.95 (ddd, $J_{N\text{-}H}$ = 10.8 Hz, J = 2.4, 0.9 Hz, 1H), 8.81 (ddd, $J_{N\text{-}H}$ = 10.8 Hz, J = 4.9, 1.6 Hz, 1H), 8.05 (ddd, J = 8.0, 2.4, 1.6 Hz, 1H), 7.51 (dddd, J = 8.0, 4.9, 1.6, 0.9 Hz, 1H), 2.72 (s, 6H).

^{13}C NMR (100 MHz, $CDCl_3$) δ 153.9, 148.9, 135.8 (s+d, J = 2.3 Hz), 132.8, 124.3, 38.2 (2C).

^{15}N NMR (41 MHz, $CDCl_3$) δ 316.0 (s, 1N).

IR (cm^{-1}) 3082, 3050, 2967, 2855, 2257, 1575, 1464, 1409, 1336, 1263, 1167, 1122, 1103, 944, 908, 729, 701, 613, 582, 535.

HRMS (ESI-TOF) m/z calcd for $C_7H_{11}N_2O_2S$ [M+H]$^+$ 187.0541; found 187.0545. (unlabeled compound)

Isotopic enrichment (^{15}N): 86.8% measured by HRMS.

3-Phenoxypyridine-1-^{15}N ([^{15}N]18)

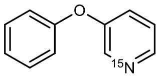

$C_{11}H_9{}^{15}NO$
MW: 172.2 g.mol^{-1}
Yellow liquid

The title compound was obtained in 75% (25.8 mg, 0.15 mmol) yield using General Procedure B with 80% IE. The crude product was purified with silica gel column chromatography (heptane/ethyl acetate = 2:1).

^{1}H NMR (400 MHz, CDCl$_3$) δ 8.45 – 8.39 (m, 1H), 8.38 – 8.32 (m, 1H), 7.40 – 7.33 (m, 2H), 7.31 – 7.23 (m, 2H), 7.19 – 7.12 (m, 1H), 7.05 – 7.00 (m, 2H).

^{13}C NMR (100 MHz, CDCl$_3$) δ 156.4, 151.9, 144.4, 141.5, 130.2 (2C), 125.5 (s+d, *J* = 2.9 Hz), 124.2, 124.2, 119.1 (2C).

^{15}N NMR (41 MHz, CDCl$_3$) δ 316.8 (s, 1N).

IR (cm^{-1}) 3671, 3058, 3036, 2987, 1591, 1569, 1489, 1471, 1413, 1243, 1015, 865, 791, 752, 691.

HRMS (ESI-TOF) *m/z* calcd for $C_{11}H_{10}NO$ [M+H]+ 172,0762; found 172,0762. (unlabeled compound)

Isotopic enrichment (^{15}N): 79.5% measured by HRMS.

***(E)*-3-Styrylpyridine-1-^{15}N ([^{15}N]19)**

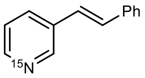

$C_{13}H_{11}{}^{15}N$
MW: 182.2 g.mol^{-1}
White solid

The title compound was obtained in 80% yield (29.2 mg, 0.16 mmol) using General Procedure B with 76% IE. The crude product was purified with silica gel column chromatography (heptane/ethyl acetate = 2:1).

^{1}H NMR (400 MHz, CDCl$_3$) δ 8.73 (ddd, $J_{N\text{-}H}$ = 10.5 Hz, *J* = 2.3, 0.9, 1H), 8.49 (ddd, $J_{N\text{-}H}$ = 10.8 Hz, *J* = 4.8, 1.8 Hz, 1H), 7.82 (ddd, *J* = 8.0, 2.3, 1.8 Hz, 1H), 7.57 – 7.49 (m, 2H), 7.41 – 7.34 (m, 2H), 7.30 (ddd, *J* = 6.7, 2.6, 1.3 Hz, 2H), 7.16 (d, *J* = 16.4 Hz, 1H), 7.07 (d, *J* = 16.4 Hz, 1H).

^{13}C NMR (100 MHz, CDCl$_3$) δ 148.5, 136.6, 132.9 (s+d, *J* = 2.5 Hz), 132.6 (s+s+d, *J* = 3.3 Hz, 2C), 130.8, 128.7 (2C), 128.2, 126.6 (2C), 124.8, 123.5 (s+d, *J* = 2.4 Hz).

^{15}N NMR (41 MHz, CDCl$_3$) δ 312.0 (s, 1N).

IR (cm^{-1}) 1562, 1493, 1449, 964, 800, 748, 702, 691, 530.

HRMS (ESI-TOF) m/z calcd for $C_{13}H_{12}N$ [M+H]$^+$ 182.0970; found 182.0972. (unlabeled compound)

Isotopic enrichment (^{15}N): 76.3% measured by HRMS.

3-(Phenylethynyl)pyridine-1-^{15}N ([^{15}N]20)

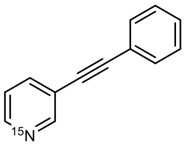

$C_{13}H_9{}^{15}N$
MW: 180.22 g.mol^{-1}
White solid

The title compound was obtained in 79% yield (28.5 mg, 0.16 mmol) using General Procedure A with 59% IE. The crude product was purified with silica gel column chromatography (heptane/ethyl acetate = 2:1).

^{1}H NMR (400 MHz, CD_2Cl_2) δ 8.75 (ddd, $J_{N\text{-}H}$ = 10.8 Hz, J = 2.2, 0.9 Hz, 1H), 8.53 (ddd, $J_{N\text{-}H}$ = 10.8 Hz, J = 4.8, 1.6 Hz, 1H), 7.83 (ddd, J = 7.9, 2.2, 1.6 Hz, 1H), 7.62 – 7.51 (m, 2H), 7.44 – 7.35 (m, 3H), 7.34 – 7.26 (m, 1H).

^{13}C NMR (100 MHz, CD_2Cl_2) δ 152.7 (s+d, J = 1.6 Hz), 149.2, 138.8, 132.2 (2C), 129.4, 129.1 (2C), 123.6, 123.1, 120.6, 92.8, 86.5.

^{15}N NMR (41 MHz, $CDCl_3$) δ 312.1 (s, 1N).

IR (cm^{-1}) 3054, 3032, 2925, 2220, 1558, 1491, 1472, 1442, 1397, 1185, 1018, 916, 802, 754, 688, 537.

HRMS (ESI-TOF) m/z calcd for $C_{13}H_{10}N$ $[M+H]^+$ 180.0813; found 180.0812. (unlabeled compound)

Isotopic enrichment (^{15}N): 59.2% measured by HRMS.

1-Methyl-5-(pyridin-3-yl -1-^{15}N)-1H-indole ([^{15}N]21)

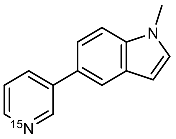

$C_{14}H_{12}{}^{15}NO$
MW: 209.3 g.mol^{-1}
White solid

The title compound was obtained in 71% yield (29.7 mg, 0.14 mmol) using General Procedure A with 82% IE. The crude product was purified with silica gel column chromatography (heptane/ethyl acetate = 2:1).

^{1}H NMR (400 MHz, $CDCl_3$) δ 8.91 (ddd, $J_{N\text{-}H}$ = 10.5 Hz, J = 2.4, 0.9, 1H), 8.55 (ddd, $J_{N\text{-}H}$ = 10.5 Hz, J = 4.8, 1.6 Hz, 1H), 7.97 – 7.89 (m, 1H), 7.87 – 7.80 (m, J = 0.8 Hz, 1H), 7.49 – 7.40 (m, 2H), 7.35 (dddd, J = 8.0, 4.8, 1.6, 0.9, 1H), 7.11 (d, J = 3.1 Hz, 1H), 6.60 – 6.49 (m, 1H), 3.84 (s, 3H).

^{13}C NMR (100 MHz, CDCl$_3$) δ 148.7, 147.6, 138.1 (s+d, *J* = 2.5 Hz), 136.7, 134.6 (s+d, *J* = 3.2 Hz), 130.0, 129.4, 129.2, 123.6 (s+d, *J* = 2.2 Hz), 121.2, 119.7, 110.0, 101.6, 33.1.

^{15}N NMR (41 MHz, CDCl$_3$) δ 311.2 (s, 1N).

IR (cm^{-1}) 3384, 3023, 2926, 2819, 1617, 1512, 1470, 1448, 1404, 1340, 1247, 1177, 1158, 1107, 1080, 1003, 883, 794, 709.

HRMS (ESI-TOF) *m/z* calcd for $C_{14}H_{13}N_2$ [M+H]+ 209,1079; found 209,1080. (unlabeled compound)

Isotopic enrichment (^{15}N): 81.7% measured by HRMS.

2-(Pyridin-3-yl-^{15}N)isoindoline-1,3-dione ([^{15}N]23)

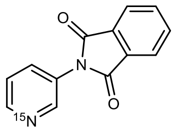

$C_{13}H_8N^{15}NO_2$
MW: 225.2 g.mol^{-1}
White solid

The title compound was obtained in 76% yield (34.2 mg, 0.15 mmol) using General Procedure A with 50% IE. While general procedure E was used, the title compound was obtained in 89% yield (40.1 mg, 0.18 mmol) using with 82% IE. The crude product was purified with silica gel column chromatography (heptane/ethyl acetate = 2:1).

^{1}H NMR (400 MHz, CDCl$_3$) δ 8.81 (m, 1H), 8.64 (m, 1H), 7.98 (dd, *J* = 5.5, 3.1 Hz, 2H), 7.86 (m, 1H), 7.83 (dd, *J* = 5.5, 3.1 Hz, 2H), 7.57 – 7.38 (m, 1H).

^{13}C NMR (100 MHz, CDCl$_3$) δ 166.8 (2C), 148.7, 147.3, 134.8 (2C), 133.6 (s+d, *J* = 3.1 Hz), 131.6 (2C), 128.8 (s+d, *J* = 2.6 Hz), 124.0 (2C), 123.7 (s+d, *J* = 2.6 Hz).

^{15}N NMR (41 MHz, CDCl$_3$) δ 314.9 (s, 1N).

IR (cm^{-1}) 3087, 3062, 3028, 2924, 1783, 1701, 1480, 1421, 1384, 1227, 1108, 1081, 879, 803, 709, 646, 531.

HRMS (ESI-TOF) m/z calcd for $C_{13}H_9N_2O_2$ [M+H]$^+$ 225.0664; found 225.0666. (unlabeled compound)

Isotopic enrichment (^{15}N): 49.5% measured by HRMS. (Procedure A)

Isotopic enrichment (^{15}N): 81.5% measured by HRMS. (Procedure E)

9-(4-(pyridin-3-yl-^{15}N)phenyl)-9H-carbazole ([^{15}N]24)

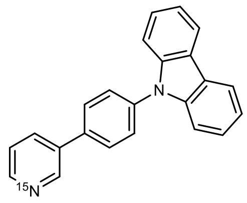

$C_{23}H_{16}N^{15}N$
MW: 321.4 g.mol^{-1}
Beige solid

The title compound was obtained in 53% yield (34.1 mg, 0.11 mmol) using General Procedure A with 40% IE. The crude product was purified with silica gel column chromatography (heptane/ethyl acetate = 2:1).

^{1}H NMR (400 MHz, CDCl$_3$) δ 8.97 (q, *J* = 5.0 Hz, 1H), 8.71 – 8.61 (m, 1H), 8.22 – 8.12 (m, 2H), 7.98 (ddd, *J* = 7.9, 2.3, 1.7 Hz, 1H), 7.86 – 7.78 (m, 2H), 7.75 – 7.66 (m, 2H), 7.46 (m, 4H), 7.40 – 7.35 (m, 1H), 7.35 – 7.28 (m, 2H).

^{13}C NMR (100 MHz, CDCl$_3$) δ 148.8 (s+d, *J* = 1.3 Hz), 148.3 (s+d, *J* = 1.5 Hz), 140.7 (2C), 137.8, 136.8, 135.8, 134.4, 128.6 (2C), 127.6 (2C), 126.1 (2C), 123.7, 123.5 (2C), 120.4 (2C), 120.2 (2C), 109.8 (2C).

^{15}N NMR (41 MHz, CDCl$_3$) δ 312.8 (s, 1N).

IR (cm^{-1}) 1600, 1518, 1477, 1450, 1363, 1335, 1318, 1227, 1170, 1148, 1019, 1000, 914, 840, 801, 746, 723, 701, 625, 565, 551, 443, 425, 407.

HRMS (ESI-TOF) m/z calcd for $C_{23}H_{17}N_2$ [M+H]$^+$ 321.1392; found 321.1393. (unlabeled compound)

Isotopic enrichment (^{15}N): 39.6% measured by HRMS.

4-(3-(Pyridin-3-yl-^{15}N)phenyl)morpholine ([^{15}N]25)

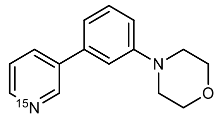

$C_{15}H_{16}{}^{15}NNO$
MW: 241.30 g.mol^{-1}
Light yellow liquid

The title compound was obtained in 76% yield (27.5 mg, 0.11 mmol) using General Procedure A with 54% IE, using 36.0 mg of 4-(3-(Pyridin-3-yl)phenyl)morpholine (0.15 mmol, 1.0 equiv.). The crude product was purified with silica gel column chromatography (heptane/ethyl acetate = 2:1).

^{1}H NMR (400 MHz, CDCl$_3$) δ 8.90 – 8.77 (m, 1H), 8.62 – 8.54 (m, 1H), 7.85 (ddd, *J* = 7.9, 2.3, 1.7 Hz, 1H), 7.41 – 7.32 (m, 2H), 7.10 – 7.06 (m, 2H), 6.96 (ddd, *J* = 8.3, 2.4, 0.8 Hz, 1H), 3.92 – 3.86 (m, 4H), 3.26 – 3.19 (m, 4H).

^{13}C NMR (100 MHz, CDCl$_3$) δ 152.0, 148.6 (s+d, *J* = 1.8 Hz), 148.5 (s+d, *J* = 1.8 Hz), 139.1, 137.3, 134.6 (s+d, *J* = 2.2 Hz), 130.0, 123.6, 119.1, 115.5, 114.6, 67.0 (2C), 49.4 (2C).

^{15}N NMR (41 MHz, CDCl$_3$) δ 311.9 (s, 1N).

IR (cm^{-1}) 3029, 2958, 2918, 2852, 2822, 1714, 1600, 1447, 1398, 1264, 1224, 1120, 941, 779, 710.

HRMS (ESI-TOF) *m/z* calcd for $C_{15}H_{17}N_2O$ [M+H]+ 241,1341; found 241,1346. (unlabeled compound)

Isotopic enrichment : 53,9% measured by HRMS.

Methyl 5-bromonicotinate-^{15}N ([^{15}N]26)

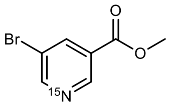

$C_7H_6Br^{15}NO_2$
MW: 217.03 g.mol^{-1}
White solid

The title compound was obtained in 73% yield (31.7 mg, 0.15 mmol) using General Procedure A with 43% IE. The crude product was purified with silica gel column chromatography (dichloromethane/ethyl acetate = 2:1).

^{1}H NMR (400 MHz, CDCl$_3$) δ 9.13 – 9.08 (m, 1H), 8.85 – 8.78 (m, 1H), 8.44 – 8.39 (m, 1H), 3.95 (s, 3H).

^{13}C NMR (100 MHz, CDCl$_3$) δ 164.6, 154.7, 149.0 , 139.6, 127.5, 120.7, 52.9.

^{15}N NMR (41 MHz, CDCl$_3$) δ 319.3 (s, 1N).

IR (cm^{-1}) 3046, 2964, 2923, 2848, 1719, 1576, 1440, 1419, 1410, 1310, 1274, 1195, 1103, 1014, 953, 901, 764, 742, 635, 648.

HRMS (ESI-TOF) m/z calcd for $C_7H_7BrNO_2$ $[M+H]^+$ 215.9660; found 215.9663. (unlabeled compound)

Isotopic enrichment (^{15}N): 43.1% measured by HRMS.

Isoquinoline-^{15}N ([^{15}N]27)

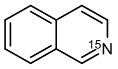

$C_9H_7{}^{15}N$
MW: 130.2 g.mol^{-1}
Yellow oil

The title compound was obtained in 66% yield (17.2 mg, 0.13 mmol) using General Procedure A with 62% IE. The crude product was purified with silica gel column chromatography (dichloromethane/ethyl acetate = 10:1). Due to the volatility of the title compound, the isolated yield is lower than the NMR yield (98%).

^{1}H NMR (400 MHz, CDCl$_3$) δ 9.31 – 9.20 (m, 1H), 8.54 – 8.50 (m, 1H), 7.96 (dd, *J* = 8.2, 0.8 Hz, 1H), 7.81 (dd, *J* = 8.2, 0.6 Hz, 1H), 7.72 – 7.56 (m, 3H).

^{13}C NMR (100 MHz, CDCl$_3$) δ 152.6, 143.0, 135.8, 130.3, 128.7, 127.6, 127.2, 126.5, 120.5.

^{15}N NMR (41 MHz, CDCl$_3$) δ 305.5 (s, 1N).

IR (cm^{-1}) 1626, 1573, 1496, 1381, 1273, 826, 742, 637, 481.

HRMS (ESI-TOF) m/z calcd for C_9H_8N [M+H]$^+$ 130.0657; found 130.0653. (unlabeled compound)

Isotopic enrichment (^{15}N): 62.2% measured by HRMS.

3-Methylisoquinoline-^{15}N ([^{15}N]28)

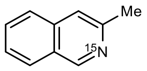

$C_{10}H_9{}^{15}N$
MW: 144.2 g.mol^{-1}
White solid

The title compound was obtained in 95% yield (27.4 mg, 0.19 mmol) using General Procedure A with 61% IE. The crude product was purified with silica gel column chromatography (dichloromethane/ethyl acetate = 10:1).

^{1}H NMR (400 MHz, CDCl$_3$) δ 9.21 – 9.13 (m, 1H), 7.91 (d, *J* = 8.2 Hz, 1H), 7.71 (d, *J* = 8.3 Hz, 1H), 7.63 (ddd, *J* = 8.2, 6.8, 1.2 Hz, 1H), 7.55 – 7.45 (m, 2H), 2.70 (d, *J* = 0.8 Hz, 3H).

^{13}C NMR (100 MHz, CDCl$_3$) δ 152.0 (s+d, *J* = 2.3 Hz), 151.6 (s+d, *J* = 1.4 Hz), 136.6, 130.3, 127.5, 126.9, 126.3, 125.9, 118.5, 24.2 (s+d, *J* = 9.1 Hz).

^{15}N NMR (41 MHz, CDCl$_3$) δ 308.9 (s, 1N).

IR (cm^{-1}) 1625, 1587, 1490, 1427, 1274, 888, 860, 755, 476.

HRMS (ESI-TOF) m/z calcd for $C_{10}H_{10}N$ [M+H]$^+$ 144.0813; found 144.0810. (unlabeled compound)

Isotopic enrichment (^{15}N): 61.0% measured by HRMS.

5-phenylpyrimidine-^{15}N ([^{15}N]29)

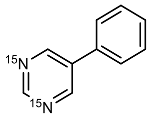

$C_{10}H_8{}^{15}N_2$
MW: 158.2 g.mol^{-1}
Yellow liquid

The title compound was obtained in 62% yield (19.5 mg, 0.12 mmol) using General Procedure B with 36% of mono-lableled **[^{15}N$_1$]29** and 40% of di-labeled **[^{15}N$_2$]29**. The crude product was purified with silica gel column chromatography (dichloromethane/ethyl acetate = 2:1).

^{1}H NMR (400 MHz, CDCl$_3$) δ 9.18 (ddd, $J_{N\text{-}H}$ = 10.5 Hz, *J* = 2.20, 0.9, 1H), 8.80 – 8.75 (ddd, $J_{N\text{-}H}$ = 10.5, *J* = 4.8, 1.8 Hz, 1H), 8.24 (ddd, *J* = 8.0, 2.2, 1.8 Hz, 1H), 7.45 – 7.39 (dddd, $J_{N\text{-}H}$ = 0.8 Hz, *J* = 8.0, 4.8, 0.9, 1H), 3.03 (q, *J* = 7.2 Hz, 2H), 1.25 (t, *J* = 7.2 Hz, 3H).

^{13}C NMR (100 MHz, CDCl$_3$) δ 157.6 (s+d, *J* = 3.4 Hz), 155.1 (s+d, *J* = 2.1 Hz, 2C), 134.5, 134.4, 129.6 (2C), 129.2, 127.1 (2C).

^{15}N NMR (41 MHz, CDCl$_3$) δ 292.3 (s, **[^{15}N$_1$]29** + **[^{15}N$_2$]29**).

IR (cm^{-1}) 3036, 1961, 1889, 1578, 1549, 1412, 1390, 1233, 1184, 1155, 1004, 904, 759, 721, 696, 629, 613, 546.

HRMS (ESI-TOF) *m/z* calcd for $C_{10}H_9N_2$ [M+H]+ 157,0766; found 157,0769. (unlabeled compound)

Isotopic enrichment : 36,4% for the mono-labeled **[^{15}N$_1$]29** and **40.4 %** for the di-labeled **[^{15}N$_2$]29** measured by HRMS.

4-Phenylpyrimidine-1-^{15}N ([^{15}N]30)

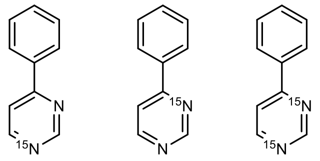

[^{15}N$_1$]$_{N1}$30 **[^{15}N$_1$]$_{N3}$30** **[^{15}N$_2$]30**

$C_{10}H_8N^{15}N$
MW: 157.2 g.mol^{-1}
Yellow solid

The title compound was obtained in 70% yield (22.0 mg, 0.14 mmol) using General Procedure A with a mixture of 48% of **30**, 44% of **[^{15}N$_1$]$_{N1}$30**, 2% of **[^{15}N$_1$]$_{N3}$30**, 6% of **[^{15}N$_2$]30**. The crude product was purified with silica gel column chromatography (dichloromethane/ethyl acetate = 2:1).

^{1}H NMR (400 MHz, CDCl$_3$) δ 9.27 (dd, *J* = 1.4, 0.4 Hz + ddd, *J* = 14.3, 1.4, 0.4 Hz + ddd, *J* = 15.1, 14.3, 1.4, 0.4 Hz, 1H, **H**-C^2), 8.76 (dd, *J* = 5.0, 0.4 Hz + ddd, *J* = 10.6, 5.0, 0.4 Hz, 1H, **H**-C^6), 8.12 – 8.06 (m, 2H, **H**Ar), 7.72 (dd, *J* = 5.0, 1.4 Hz, 1H, **H**-C^5), 7.54 – 7.49 (m, 3H, **H**Ar).

^{13}C NMR (100 MHz, CDCl$_3$) δ 163.9, 159.1 (d, *J* = 3.3 Hz), 157.4, 136.5, 131.1, 129.0 (2C), 127.1 (2C), 117.0.

^{15}N NMR coupled with ^{1}H (41 MHz, acetone-*d$_6$*) δ 336.08 (dd, *J* = 14.1, 11.0 Hz, N^1), 330.87 (d, *J* = 15.1 Hz, N^3).

IR (cm^{-1}) 1572, 1537, 1494, 1464, 1390, 1381, 1321, 851, 741, 688, 676, 621.

HRMS (ESI-TOF) m/z calcd for $C_{10}H_9N_2$ [M+H]$^+$ 157.0766; found 157.0771. (unlabeled compound)

Isotopic enrichment ([^{15}N$_1$]30): 46.36% measured by HRMS.
Isotopic enrichment ([^{15}N$_2$]30): 6.04% measured by HRMS.

Isotopic enrichment ([^{15}N$_1$]$_{N1}$30): 44% measured by NMR.
Isotopic enrichment ([^{15}N$_1$]$_{N2}$30): 2% measured by NMR.

*Details of the calculation for the respective IE of **[$^{15}N_1$]$_{N1}$30** and **[$^{15}N_1$]$_{N3}$30** are given in part dedicated to the characterizations of compound **30** (section 13. NMR spectra and isotopic enrichment).*

^{15}N-Nicotinamide (S5)

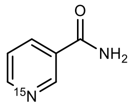

$C_6H_6N^{15}NO$
MW: 123.1 g.mol^{-1}
White solid

The title compound was obtained in 86% (21.2 mg, 0.17 mmol) yield using General Procedure E with 86% IE. The crude product was purified with silica gel column chromatography (dichloromethane/ethyl acetate = 2:1).

^{1}H NMR (400 MHz, CD_3OD) δ 9.03 (m, 1H), 8.69 (m, 1H), 8.29 (m, 1H), 7.55 (m, 1H). The amide protons are not visible on the spectrum.

^{13}C NMR (100 MHz, CD_3OD) δ 169.8, 152.8, 149.5, 137.3 (s+d, *J* = 3.3 Hz), 131.5 (m), 125.1.

^{15}N NMR (41 MHz, CD_3OD) δ 301.7 (s, 1N).

IR (cm^{-1}) 3360, 3151, 1674, 1615, 1592, 1574, 1458, 1421, 1392, 1339, 1201, 1153, 1123, 1028, 936, 828, 777, 722, 701, 644, 620, 599, 510, 464, 419, 411.

HRMS (ESI-TOF) *m/z* Calcd for $C_6H_7{}^{15}NNO$ $[M+H]^+$ 124.0529; Found 124.0526. (labeled compound)

Isotopic enrichment (^{15}N): 85.7% measured by HRMS.

Methyl nicotinate-^{15}N (S6)

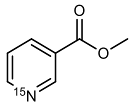

$C_7H_7{}^{15}NO_2$
MW: 138.1 g.mol^{-1}
Yellow oil

The title compound was obtained in 67% yield (18.5 mg, 0.13 mmol) using General Procedure E with 85% IE. The crude product was purified with silica gel column chromatography (dichloromethane/ethyl acetate = 5:1).

^{1}H NMR (400 MHz, $CDCl_3$) δ 9.23 (m, 1H), 8.77 (m, 1H), 8.30 (dt, *J* = 8.0, 2.0 Hz, 1H), 7.39 (dd, *J* = 7.9, 4.9 Hz, 1H), 3.96 (s, 3H).

^{13}C NMR (100 MHz, $CDCl_3$) δ 165.8, 153.4, 150.9 (s+d, *J* = 1.4 Hz), 137.1, 126.1 (s+d, *J* = 2.0 Hz), 123.3 (s+d, *J* = 0.6 Hz), 52.4.

IR (cm^{-1}) 3381, 2923, 2868, 1727, 1619, 1455, 1403, 1378, 1289, 1251, 1128, 1102, 1067, 1030, 874.

HRMS (ESI-TOF) ***m/z*** Calcd for $C_{11}H_{10}{}^{15}N$ $[M+H]^+$ 139.0525; Found 139.0523. (labeled compound)

Isotopic enrichment (^{15}N): 85.0% measured by HRMS.

7.3 Characterizations of ^{15}*N*-labeled pharmaceuticals and bioactive molecules

^{15}N-Etofibrate ([^{15}N]31)

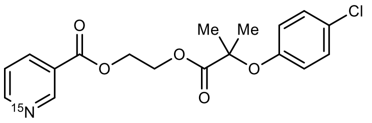

$C_{18}H_{18}{}^{15}NO_5SCl$
MW: 364.8 g.mol^{-1}
White solid

The title compound was obtained in 72% yield (52.5 mg, 0.14 mmol) using General Procedure B with 80% IE. The crude product was purified with silica gel column chromatography (heptane/ethyl acetate = 3:1).

^{1}H NMR (400 MHz, CDCl$_3$) δ 9.14 (ddd, $J_{N\text{-}H}$ = 10.5 Hz, J = 2.2, 0.9 Hz, 1H), 8.79 (ddd, $J_{N\text{-}H}$ = 10.8 Hz, J = 4.8, 1.8 Hz, 1H), 8.13 (ddd, *J* = 8.0, 2.2, 1.8 Hz, 1H), 7.37 (ddd, *J* = 8.0, 4.8, 0.9, 1H), 7.14 – 7.06 (m, 2H), 6.80 – 6.69 (m, 2H), 4.57 – 4.51 (m, 4H), 1.59 (s, 6H).

^{13}C NMR (100 MHz, CDCl$_3$) δ 173.9, 164.9, 153.9, 153.7, 150.9, 137.0 (s+d, *J* = 3.2 Hz), 129.1 (2C), 127.2, 125.4 (s+d, *J* = 2.6 Hz), 123.3 (s+d, *J* = 2.3 Hz), 120.1 (2C), 79.3, 63.0, 62.7, 25.3 (2C).

^{15}N NMR (41 MHz, CDCl$_3$) δ 312.9 (s, 1N).

IR (cm^{-1}) 1730, 1588, 1489, 1273, 1238, 1130, 828, 740.

HRMS (ESI-TOF) m/z calcd for $C_{18}H_{19}ClNO_5$ $[M+H]^+$ 364.0952; found 364.0952. (unlabeled compound)

Isotopic enrichment (^{15}N): 80.1% measured by HRMS.

^{15}N-Nicergoline ([^{15}N]32)

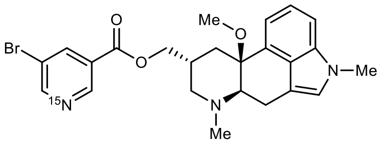

$C_{24}H_{26}N_2{}^{15}NO_3Br$
MW: 485.4 g.mol^{-1}
White solid

The title compound was obtained in 60% yield (58.2 mg, 0.12 mmol) using General Procedure B with 41% IE. The crude product was purified with silica gel column chromatography (heptane/ethyl acetate = 3:1).

^{1}H NMR (400 MHz, CDCl$_3$) δ 9.15 – 9.12 (m, 1H), 8.87 – 8.84 (m, 1H), 8.43 (t, *J* = 2.0 Hz, 1H), 7.24 – 7.18 (m, 2H), 7.05 (d, *J* = 6.5 Hz, 1H), 6.80 (d, *J* = 1.0 Hz, 1H), 4.43 – 4.27 (m, 2H), 3.78 (s, 3H), 3.23 – 3.21 (m, 2H), 3.07 – 2.96 (m, 5H), 2.66 – 2.60 (m, 1H), 2.48 (s, 3H), 2.37 (dd, *J* = 11.2, 4.5 Hz, 1H), 2.10 (t, *J* = 11.2 Hz, 1H), 1.39 (t, *J* = 13.3 Hz, 1H).

^{13}C NMR (100 MHz, CDCl$_3$) δ 164.0, 154.6, 148.7, 139.5, 135.1, 129.6, 127.4, 126.3, 123.3, 121.4, 120.7, 114.9, 110.2, 109.0, 73.5, 70.0, 68.5, 60.5, 49.5, 43.8, 32.8, 31.5, 30.1, 22.3.

^{15}N NMR (41 MHz, CDCl$_3$) δ 319.2 (s, 1N).

IR (cm^{-1}) 1726, 1579, 1467, 1367, 1305, 1269, 1072, 1021, 936, 747.

HRMS (ESI-TOF) m/z calcd for $C_{24}H_{27}BrN_3O_3$ [M+H]$^+$ 484.1236; found 484.1234. (unlabeled compound)

Isotopic enrichment (^{15}N): 40.7% measured by HRMS.

^{15}N-Abiraterone acetate ([^{15}N]33)

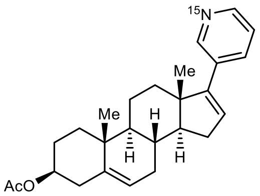

$C_{26}H_{33}{}^{15}NO_2$
MW: 392.5 g.mol^{-1}
White solid

The title compound was obtained in 90% yield (70.7 mg, 0.18 mmol) using General Procedure B with 54% IE. The crude product was purified with silica gel column chromatography (heptane/ethyl acetate = 3:1).

^{1}H NMR (400 MHz, CDCl$_3$) δ 8.60 (ddd, $J_{N\text{-}H}$ = 10.5 Hz, J = 2.2, 0.9 Hz, 1H), 8.44 (ddd, $J_{N\text{-}H}$ = 10.8 Hz, J = 4.8, 1.8 Hz, 1H), 7.63 (ddd, *J* = 8.0, 2.2, 1.8 Hz, 1H), 7.20 (ddd, *J* = 8.0, 4.8, 0.9, 1H), 5.98 (dd, *J* = 3.2, 1.8 Hz, 1H), 5.41 (d, *J* = 5.2 Hz, 1H), 4.65 – 4.57 (m, 1H), 2.40 – 2.30 (m, 2H), 2.29 – 2.23 (m, 1H), 2.11 – 1.98 (m, 6H), 1.91 – 1.82 (m, 2H), 1.82 – 1.53 (m, 6H), 1.48 (td, *J* = 12.0, 5.1 Hz, 1H), 1.21 – 1.06 (m, 5H), 1.04 (d, *J* = 7.9 Hz, 3H).

^{13}C NMR (100 MHz, CDCl$_3$) δ 170.5, 151.6, 147.9 (d, *J* = 1.5 Hz), 147.8 (d, *J* = 1.5 Hz), 140.0, 133.7, 133.6, 132.9, 129.2, 123.0, 122.2, 73.8, 57.4, 50.2, 47.3, 38.1, 36.9, 35.1, 31.7, 31.4, 30.4, 27.7, 21.4, 20.8, 19.2, 16.5.

^{15}N NMR (41 MHz, CDCl$_3$) δ 310.6 (s, 1N).

IR (cm^{-1}) 1730, 1372, 1238, 1031, 906, 795, 731, 709, 611.

HRMS (ESI-TOF) m/z calcd for $C_{26}H_{34}NO_2$ [M+H]$^+$ 392.2589; found 392.2592. (unlabeled compound)

Isotopic enrichment (^{15}N): 54.2% measured by HRMS.

^{15}N-Nicoboxil ([^{15}N]34)

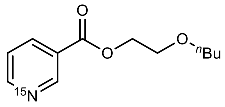

$C_{12}H_{17}{}^{15}NO_3$
MW: 224.3 g.mol^{-1}
Colorless oil

The title compound was obtained in 95% yield (42.6 mg, 0.19 mmol) using General Procedure A with 64% IE. The crude product was purified with silica gel column chromatography (heptane/ethyl acetate = 3:1).

^{1}H NMR (400 MHz, CDCl$_3$) δ 9.23 (ddd, J_{N-H} = 10.5 Hz, J = 2.2, 0.9 Hz, 1H), 8.76 (ddd, J_{N-H} = 10.8 Hz, J = 4.8, 1.8 Hz, 1H), 8.30 (ddd, *J* = 8.0, 2.2, 1.8 Hz, 1H), 7.38 (ddd, *J* = 8.0, 4.8, 0.9, 1H), 4.57 – 4.42 (m, 2H), 3.78 – 3.73 (m, 2H), 3.50 (t, *J* = 6.6 Hz, 2H), 1.57 (tt, *J* = 12.0, 6.5 Hz, 2H), 1.36 (tdd, *J* = 14.5, 8.4, 6.4 Hz, 2H), 0.90 (t, *J* = 7.4 Hz, 3H).

^{13}C NMR (101 MHz, CDCl$_3$) δ 165.2, 153.4, 150.9, 137.1 (s+d, *J* = 2.1 Hz), 126.0 (s+d, *J* = 2.6 Hz), 123.2 (s+d, *J* = 1.1 Hz), 71.2, 68.4, 64.6, 31.6, 19.2, 13.8.

^{15}N NMR (41 MHz, CDCl$_3$) δ 312.6 (s, 1N).

IR (cm^{-1}) 2958, 1724, 1588, 1279, 1110, 1024, 740, 700.

HRMS (ESI-TOF) m/z calcd for $C_{12}H_{18}NO_3$ [M+H]$^+$ 224.1287; found 224.1291. (unlabeled compound)

Isotopic enrichment (^{15}N): 63.7% measured by HRMS.

^{15}N-Nicotine ([^{15}N]35)

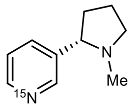

$C_{10}H_{14}N^{15}N$
MW: 163.2 g.mol^{-1}
Colorless oil

The title compound was obtained in 45% yield (14.7 mg, 0.09 mmol) using General Procedure C with 98% IE. The crude product was purified with silica gel column chromatography (heptane/ethyl acetate = 3:1). Due to the volatility of the title compound, the isolated yield is lower than the NMR yield (65%).

^{1}H NMR (400 MHz, CDCl$_3$) δ 8.51 (ddd, J_{N-H} = 10.5 Hz, J = 2.2, 0.9 Hz, 1H), 8.47 (ddd, J_{N-H} = 10.8 Hz, J = 4.8, 1.8 Hz, 1H), 7.69 (ddd, *J* = 8.0, 2.2, 1.8 Hz, 1H), 7.24 (ddd, *J* = 8.0, 4.8, 0.9, 1H), 3.27 – 3.18 (m, 1H), 3.07 (t, *J* = 8.3 Hz, 1H), 2.31 – 2.26 (m, 1H), 2.25 – 2.10 (m, 4H), 2.02 – 1.88 (m, 1H), 1.87 – 1.77 (m, 1H), 1.76 – 1.65 (m, 1H).

^{13}C NMR (100 MHz, CDCl$_3$) δ 149.4, 148.5, 138.7 (d, *J* = 2.6 Hz), 134.9 (d, *J* = 3.5 Hz), 123.6 (d, *J* = 2.6 Hz), 68.8, 57.0, 40.3, 35.1, 22.5.

^{15}N NMR (41 MHz, CDCl$_3$) δ 310.2 (s, 1N).
IR (cm^{-1}) 1574, 1423, 1314, 1188, 1045, 1019, 901, 805, 712, 609.

HRMS (ESI-TOF) m/z calcd for $C_{10}H_{15}N_2$ [M+H]$^+$ 163.1235; found 163.1232. (unlabeled compound)

Isotopic enrichment (^{15}N): 98.2% measured by HRMS.

^{15}N-Nifenazone ([^{15}N]36)

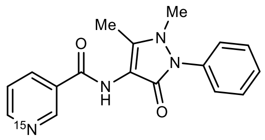

$C_{17}H_{16}N_3{}^{15}NO_2$
MW: 309.3 g.mol^{-1}
Yellow solid

The title compound was obtained in 64% yield (39.6 mg, 0.13 mmol) using General Procedure C with 38% IE. The crude product was purified with silica gel column chromatography (dichloromethane/ethyl acetate = 3:1).

^{1}H NMR (400 MHz, CDCl$_3$) δ 9.68 (s, 1H), 9.02 (ddd, $J_{N\text{-}H}$ = 10.5 Hz, J = 2.2, 0.9 Hz, 1H), 8.63 (ddd, $J_{N\text{-}H}$ = 10.8 Hz, J = 4.8, 1.8 Hz, 1H), 8.12 (ddd, *J* = 8.0, 2.2, 1.8 Hz, 1H), 7.51 – 7.42 (m, 2H), 7.40 – 7.29 (m, 3H), 7.24 (ddd, *J* = 8.0, 4.8, 0.9, 1H), 3.11 (s, 3H), 2.20 (s, 3H).

^{13}C NMR (100 MHz, CDCl$_3$) δ 164.6, 161.9, 152.1, 150.2, 149.1, 135.2, 134.1, 129.4 (2C), 128.9, 127.5, 125.1 (2C), 123.1, 107.6, 35.5, 12.0.

^{15}N NMR (41 MHz, CDCl$_3$) δ 311.1 (s, 1N).

IR (cm^{-1}) 1650, 1590, 1496, 1301, 1188, 1137, 1058, 697, 593.

HRMS (ESI-TOF) m/z calcd for $C_{17}H_{17}N_4O_2$ [M+H]$^+$ 309.1352; found 309.1351. (unlabeled compound)

Isotopic enrichment (^{15}N): 37.7% measured by HRMS.

^{15}N-Bisacodyl ([^{15}N]37)

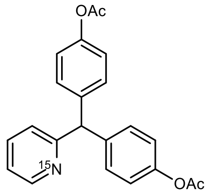

$C_{22}H_{19}{}^{15}NO_4$
MW: 362.4 g.mol^{-1}
White solid

The title compound was obtained in 80% yield (58.0 mg, 0.16 mmol) using General Procedure A with 68% IE. The crude product was purified with silica gel column chromatography (heptane/ethyl acetate = 5:1).

^{1}H NMR (400 MHz, CDCl$_3$) δ 8.59 (dddd, $J_{N\text{-}H}$ = 11.2 Hz, J = 4.8, 1.8, 1.0 Hz, 1H), 7.62 (ddd, J = 8.0, 7.3, 1.0 Hz, 1H), 7.20 – 7.15 (m, 4H), 7.15 – 7.12 (m, 1H) 7.12 – 7.09 (m, 1H), 7.04 – 7.00 (m, 4H), 5.66 (s, 1H), 2.27 (s, 6H).
^{13}C NMR (100 MHz, CDCl$_3$) δ 169.4 (2C), 162.5, 149.6, 149.2 (2C), 139.9 (2C), 136.6 (d, J = 2.0 Hz), 130.2 (4C), 123.7, 121.6, 121.4 (4C), 58.1 (d, J = 8.4 Hz), 21.1 (2C).

^{15}N NMR (41 MHz, CDCl$_3$) δ 313.8 (s, 1N).

IR (cm^{-1}) 1752, 1584, 1502, 1431, 1368, 1189, 1163, 1016, 911, 839, 730, 660, 610, 594, 564, 516.

HRMS (ESI-TOF) m/z calcd for $C_{22}H_{20}NO_4$ $[M+H]^+$ 362.1392; found 362.139. (unlabeled compound)

Isotopic enrichment (^{15}N): 68.4% measured by HRMS.

^{15}N-Vismodegib ([^{15}N]38)

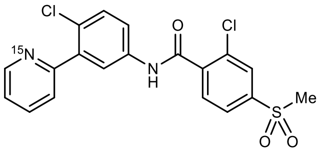

$C_{19}H_{14}{}^{15}NNO_3SCl_2$
MW: 422.3 g.mol^{-1}
White solid

The title compound was obtained in 59% yield (49.8 mg, 0.12 mmol) using General Procedure C with 27% IE. The crude product was purified with silica gel column chromatography (heptane/ethyl acetate = 5:1).

^{1}H NMR (400 MHz, CDCl$_3$) δ 9.74 (s, 1H), 8.38 (dddd, $J_{N\text{-}H}$ = 11.2 Hz, J = 4.8, 1.8, 1.0 Hz, 1H), 8.03 (dd, J = 8.8, 2.7 Hz, 1H), 7.85 (d, J = 1.6 Hz, 1H), 7.72 (ddd, J = 8.0, 7.3, 1.0 Hz, 1H), 7.67 (ddd, J = 8.0, 1.3, 1.0 Hz, 1H), 7.19 (ddd, J = 7.3, 4.8, 1.3 Hz, 1H), 2.99 (s, 3H).

^{13}C NMR (100 MHz, CDCl$_3$) δ 163.7, 155.8, 148.8, 142.6, 140.6, 138.6, 137.0, 136.4, 132.2, 131.0, 130.0, 128.8, 127.4, 125.8, 125.4, 122.8, 122.7, 121.7, 44.3.

^{15}N NMR (41 MHz, CDCl$_3$) δ 306.6 (s, 1N).

IR (cm^{-1}) 1681, 1587, 1542, 1461, 1400, 1315, 1154, 1099, 907, 809, 731, 566.

HRMS (ESI-TOF) m/z calcd for $C_{19}H_{15}Cl_2N_2O_2S$ $[M+H]^+$ 421.018; found 421.0178. (unlabeled compound)

Isotopic enrichment (^{15}N): 26.8% measured by HRMS.

^{15}*N*-Loratadine ([^{15}N]39)

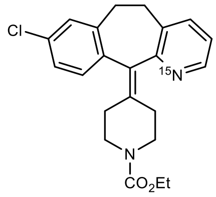

$C_{22}H_{23}N^{15}NO_2Cl$
MW: 383.9 g.mol^{-1}
Yellow solid

The title compound was obtained in overall 28% yield (50% for the Zincke imine preparation and 56% for the ^{15}*N*-labeling) (21.5 mg, 0.015 mmol) using a modified General Procedure D with 57% IE. The reaction was stirred at 100 °C for 24 hours. The crude product was purified with silica gel column chromatography (heptane/ethyl acetate = 1:1).

^{1}H NMR (400 MHz, $CDCl_3$) δ 8.40 (ddd, $J_{N\text{-}H}$ = 11.0 Hz, *J* = 4.8, 1.8 Hz, 1H), 7.43 (dd, *J* = 7.7, 1.8 Hz, 1H), 7.20 – 7.11 (m, 3H), 7.09 (dd, *J* = 7.3, 4.8 Hz, 1H), 4.18 – 4.09 (m, 2H), 3.80 (br, 2H), 3.43 – 3.30 (m, 2H), 3.22 – 3.05 (m, 2H), 2.90 – 2.73 (m, 2H), 2.51 – 2.47 (m, 1H), 2.41 – 2.25 (m, 3H), 1.24 (t, *J* = 7.1 Hz, 3H).

^{13}C NMR (100 MHz, $CDCl_3$) δ 157.0, 155.5, 146.7, 139.5, 137.6, 137.4 (2C), 134.2, 133.3, 132.9, 130.5, 129.0, 126.1, 122.2, 61.3, 44.8 (2C), 44.7, 31.7 (2C), 31.4, 14.7.

^{15}N NMR (41 MHz, $CDCl_3$) δ 311.3 (s, 1N).

IR (cm^{-1}) 1692, 1432, 1277, 1222, 1172, 1113, 996, 924, 829, 767, 730.

HRMS (ESI-TOF) m/z calcd for $C_{22}H_{24}ClN_2O_2$ $[M+H]^+$ 383.1526; found 383.1527. (unlabeled compound)

Isotopic enrichment (^{15}N): 57.0% measured by HRMS.

^{15}*N*-Etoricoxib ([^{15}N]40)

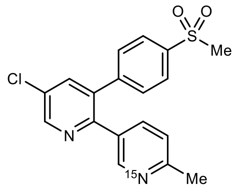

$C_{18}H_{15}N^{15}NO_2SCl$
MW: 359.8 g.mol^{-1}
White solid

The title compound was obtained in 89% yield (64.0 mg, 0.18 mmol) using General Procedure C with 83% IE. The crude product was purified with silica gel column chromatography (heptane/ethyl acetate = 3:1). According to the NMR spectra, only the nitrogen of disubstituted pyridine was lableled.

^{1}H NMR (400 MHz, $CDCl_3$) δ 8.70 (d, *J* = 2.4 Hz, 1H), 8.68 (ddd, $J_{N\text{-}H}$ = 11.2 Hz, *J* = 4.8, 2.4, 0.9, 0.3 Hz, 1H), 7.95 – 7.83 (m, 2H), 7.72 (d, *J* = 2.4 Hz, 1H), 7.55 (dd, *J* = 8.0, 2.4 Hz, 1H), 7.44 – 7.35 (m2H), 7.08 (d, *J* = 8.0 Hz, 1H), 3.08 (s, 3H), 2.57 – 2.47 (m, 3H).

^{13}C NMR (100 MHz, CDCl$_3$) δ 158.5, 152.3, 149.8, 148.4, 143.8, 140.2, 137.9, 137.2, 135.2, 131.2 (s+d, *J* = 2.7 Hz), 131.1, 130.3 (2C), 127.9 (2C), 122.8, 44.5, 24.2 (s+d, *J* = 9.2 Hz).

^{15}N NMR (41 MHz, CDCl$_3$) δ 310.4 (s, 1N).

IR (cm^{-1}) 1597, 1430, 1311, 1149, 1090, 1005, 957, 907, 840, 770, 731, 586, 544.

HRMS (ESI-TOF) m/z calcd for $C_{18}H_{16}ClN_2O_2S$ [M+H]$^+$ 359.0621; found 359.0622.

Isotopic enrichment (^{15}N): 83.1% measured by HRMS.

^{15}N-Metyrapone ([^{15}N]41)

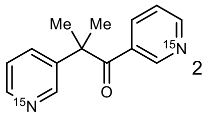

1 2

$C_{14}H_{14}{}^{15}NNO$
MW: 228.3 g.mol^{-1}
White solid

The title compound was obtained in 50% yield (22.8 mg, 0.10 mmol) using General Procedure C with 77% IE. The crude product was purified with silica gel column chromatography (dichloromethane/ethyl acetate = 3:1). According to the NMR spectra and MS-MS analysis, the enrichment of N1 and N2 are 16% and 61%, respectively.

^{1}H NMR (400 MHz, CDCl$_3$) δ 8.72 – 8.51 (m, 4H), 7.79 (ddd, *J* = 8.1, 2.2, 1.8 Hz, 1H), 7.60 (ddd, *J* = 8.0, 2.5, 1.6 Hz, 1H), 7.30 (dd, *J* = 7.7, 5.1 Hz, 1H), 7.22 (ddd, *J* = 8.0, 4.8, 0.7 Hz, 1H), 1.66 (s, 6H).

^{13}C NMR (100 MHz, CDCl$_3$) δ 201.2, 152.4, 150.8, 148.5, 147.3, 139.9, 136.9, 133.6, 131.0, 124.0, 123.2, 50.3, 27.3 (2C).

^{15}N NMR (41 MHz, CDCl$_3$) δ 314.4 (s, 1N), 312.1 (s, 1N).

IR (cm^{-1}) 1681, 1581, 1468, 1415, 1258, 1024, 976, 814, 741, 715.

HRMS (ESI-TOF) m/z calcd for $C_{14}H_{15}N_2O$ [M+H]$^+$ 227.1184; found 227.1188. (unlabeled compound)

Isotopic enrichment (^{15}N): 76.8% measured by HRMS.

7.4 Characterizations of nitrogen-carbon exchange products

[1,1'-Biphenyl]-4-yl(phenyl)methanone (42)

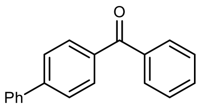

$C_{19}H_{14}O$
MW: 258.3 g.mol^{-1}
Yellow solid

The title compound was obtained in 90% yield (23.2 mg, 0.09 mmol) using General Procedure F. While the Zincke imine was used without isolation, the title compound was obtained in 45% yield (23.2 mg, 0.09 mmol). The crude product was purified with silica gel column chromatography (heptane/ethyl acetate = 50:1).

^{1}H NMR (400 MHz, $CDCl_3$) δ 7.94 – 7.81 (m, 4H), 7.74 – 7.69 (m, 2H), 7.67 – 7.64 (m, 2H), 7.63 – 7.58 (m, 1H), 7.54 – 7.45 (m, 4H), 7.45 – 7.38 (m, 1H).

^{13}C NMR (100 MHz, $CDCl_3$) δ 196.4, 145.2, 140.0, 137.8, 136.2, 132.4, 130.7 (2C), 130.0 (2C), 129.0 (2C), 128.3 (2C), 128.2, 127.3 (2C), 127.0 (2C).

All analytical data were in good accordance with data reported in the literature[2].

[1,1'-Biphenyl]-4-yl(3,4-dimethoxyphenyl)methanone (43)

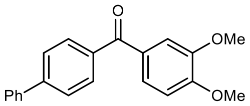

$C_{21}H_{18}O_3$
MW: 318.4 g.mol^{-1}
Yellow solid

The title compound was obtained in 85% (27.1 mg, 0.085 mmol) yield using General Procedure F. The crude product was purified with silica gel column chromatography (heptane/ethyl acetate = 50:1).

^{1}H NMR (400 MHz, $CDCl_3$) δ 7.92 – 7.83 (m, 2H), 7.73 – 7.68 (m, 2H), 7.68 – 7.63 (m, 2H), 7.55 – 7.37 (m, 5H), 6.92 (d, J = 8.4 Hz, 1H), 3.97 (s, 3H), 3.96 (s, 3H).

^{13}C NMR (100 MHz, $CDCl_3$) δ 195.2, 153.0, 149.0, 144.7, 140.0, 136.9, 130.4 (2C), 130.3, 128.9 (2C), 128.1, 127.2 (2C), 126.8 (2C), 125.4, 112.1, 109.8, 56.1, 56.0.

IR (cm^{-1}) 1647, 1595, 1510, 1271, 1130, 1023, 748.

HRMS (ESI-TOF) m/z calcd for $C_{21}H_{19}O_3$ $[M+H]^+$ 319.1334; found 319.1334.

[1,1'-Biphenyl]-4-yl(3-bromophenyl)methanone (44)

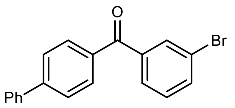

$C_{19}H_{13}OBr$
MW: 337.2 g.mol^{-1}
Yellow solid

The title compound was obtained in 53% yield (17.6 mg, 0.053 mmol) using General Procedure F. The crude product was purified with silica gel column chromatography (heptane/ethyl acetate = 50:1).

^{1}H NMR (400 MHz, CDCl$_3$) δ 7.97 (t, *J* = 1.8 Hz, 1H), 7.91 – 7.85 (m, 2H), 7.79 – 7.70 (m, 4H), 7.68 – 7.63 (m, 2H), 7.51 – 7.47 (m, 2H), 7.44 – 7.36 (m, 2H).

^{13}C NMR (101 MHz, CDCl$_3$) δ 194.7, 145.7, 139.8, 139.6, 135.5, 135.2, 132.3, 130.7 (2C), 129.9, 129.0 (2C), 128.5, 128.3, 127.3 (2C), 127.1 (2C), 122.6.

IR (cm^{-1}) 1656, 1602, 1560, 1404, 1312, 1277, 1007, 941, 836, 769, 745, 715, 697.

HRMS (ESI-TOF) m/z calcd for $C_{19}H_{14}BrO$ $[M+H]^+$ 337.0228; found 337.0225.

[1,1'-Biphenyl]-4-yl(thiophen-3-yl)methanone (45)

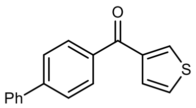

$C_{17}H_{12}OS$
MW: 264.3 g.mol^{-1}
Yellow solid

The title compound was obtained in 55% yield (14.6 mg, 0.055 mmol) using General Procedure F. The crude product was purified with silica gel column chromatography (heptane/ethyl acetate = 50:1).

^{1}H NMR (400 MHz, CDCl$_3$) δ 7.98 (dd, *J* = 2.9, 1.2 Hz, 1H), 7.97 – 7.92 (m, 2H), 7.74 – 7.70 (m, 2H), 7.69 – 7.61 (m, 3H), 7.52 – 7.46 (m, 2H), 7.45 – 7.38 (m, 2H).

^{13}C NMR (100 MHz, CDCl$_3$) δ 189.6, 145.1, 141.4, 140.0, 137.2, 133.7, 130.0 (2C), 129.0 (2C), 128.6, 128.2, 127.3 (2C), 127.0 (2C), 126.2.

IR (cm^{-1}) 1635, 1601, 1512, 1410, 1272, 1139, 971, 852, 775, 740, 699.

HRMS (ESI-TOF) m/z calcd for $C_{17}H_{13}OS$ $[M+H]^+$ 265.0687; found 265.069.

[1,1'-Biphenyl]-4-yl(5-methylthiophen-2-yl)methanone (46)

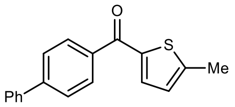

$C_{18}H_{14}OS$
MW: 278.4 g.mol^{-1}
Yellow solid

The title compound was obtained in 50% yield (14.0 mg, 0.050 mmol) using General Procedure F. The crude product was purified with silica gel column chromatography (heptane/ethyl acetate = 50:1).

^{1}H NMR (400 MHz, CDCl$_3$) δ 7.97 – 7.88 (m, 2H), 7.73 – 7.68 (m, 2H), 7.64 (dq, *J* = 2.7, 1.8 Hz, 2H), 7.55 – 7.45 (m, 3H), 7.44 – 7.37 (m, 1H), 6.86 (dd, *J* = 3.8, 1.0 Hz, 1H), 2.59 (d, *J* = 0.6 Hz, 3H).

^{13}C NMR (100 MHz, CDCl$_3$) δ 187.5, 150.4, 144.8, 141.5, 140.0, 137.0, 135.5, 129.7 (2C), 128.9 (2C), 128.1, 127.3 (2C), 127.0 (2C), 126.7, 16.1.

IR (cm^{-1}) 1627, 1603, 1448, 1298, 870, 743, 698.

HRMS (ESI-TOF) m/z calcd for $C_{18}H_{15}OS$ $[M+H]^+$ 279.0844; found 279.0847.

[1,1'-Biphenyl]-3-yl(phenyl)methanone (47)

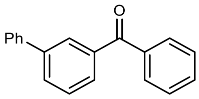

$C_{19}H_{14}O$
MW: 258.3 g.mol^{-1}
Yellow solid

The title compound was obtained in 72% yield (18.6 mg, 0.07 mmol) using General Procedure E. The crude product was purified with silica gel column chromatography (heptane/ethyl acetate = 50:1).

^{1}H NMR (400 MHz, CDCl$_3$) δ 8.03 (dd, *J* = 2.5, 1.1 Hz, 1H), 7.89 – 7.75 (m, 4H), 7.64 – 7.43 (m, 8H), 7.41 – 7.35 (m, 1H).

^{13}C NMR (100 MHz, CDCl$_3$) δ 196.7, 141.4, 140.1, 138.2, 137.6, 132.5, 131.0, 130.1 (2C), 128.9 (3C), 128.7, 128.6, 128.3 (2C), 127.8, 127.2 (2C).

All analytical data were in good accordance with data reported in the literature[3].

(3,4-Dimethoxyphenyl)(phenyl-d$_5$)methanone ([$^2H_5$]48)

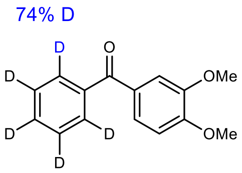

$C_{15}H_9D_5O_3$
MW: 247.3 g.mol^{-1}
Yellow solid

The title compound was obtained in 80% (19.6 mg, 0.08 mmol) yield using General Procedure G. The crude product was purified with silica gel column chromatography (heptane/ethyl acetate = 50:1).

^{1}H NMR (400 MHz, CDCl$_3$) δ 7.77 (s, 0.24 H), 7.50 (d, *J* = 2.0 Hz, 1H), 7.38 (dd, *J* = 8.3, 2.0 Hz, 1H), 6.89 (d, *J* = 8.4 Hz, 1H), 3.96 (s, 3H), 3.95 (s, 3H).

^{13}C NMR (100 MHz, CDCl$_3$) δ 195.6, 154.0, 149.0, 130.2, 125.5, 112.1, 109.7, 56.1, 56.0. Due to the deuterium isotope effects, signals of the carbons on the deuterated benzene are too small to be characterized.

^{2}H NMR (61 MHz, CHCl$_3$) δ 7.60 (s, 1.69D), 7.48 (s, 1D), 7.43 (s, 2D).

IR (cm^{-1}) 1680, 1632, 1555, 1495, 1305, 1228, 1111, 1058, 1065, 776.

HRMS (ESI-TOF) m/z calcd for $C_{15}H_{10}O_3D_5$ $[M+H]^+$ 248.1335; found 248.1340 (labeled compound).

Isotopic purity (5D): 73.6% measured by HRMS.

(Phenyl-d$_5$)(thiophen-3-yl)methanone ([$^2H_5$]49)

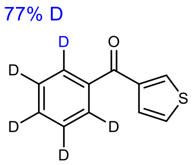

$C_{11}H_3D_5OS$
MW: 194.1 g.mol^{-1}
Yellow oil

The title compound was obtained in 50% (9.5 mg, 0.05 mmol) yield using General Procedure G. The crude product was purified with silica gel column chromatography (heptane/ethyl acetate = 50:1).

^{1}H NMR (400 MHz, CDCl$_3$) δ 7.94 (dd, *J* = 2.9, 1.2 Hz, 1H), 7.85 (s, 0.28H), 7.61 (dd, *J* = 5.1, 1.2 Hz, 1H), 7.39 (dd, *J* = 5.1, 2.9 Hz, 1H). The deuterium losing position was determined by the comparison with the undeuterated compound and the ^{1}H-^{1}H COSY of the undeuterated compound.

^{13}C NMR (100 MHz, CDCl$_3$) δ 190.0, 141.3, 133.9, 128.6, 126.2. Due to the deuterium isotope effects, signals of the carbons on the deuterated benzene are too tiny to be characterized.

^{2}H NMR (61 MHz, CHCl$_3$) δ 7.66 (s, 1.79D), 7.50 (s, 1D), 7.44 (s, 2D).

IR (cm^{-1}) 1658, 1531, 1455, 1222, 1188, 955, 867, 731, 722, 668.

HRMS (ESI-TOF) m/z calcd for $C_{11}H_3OD_5S$ $[M+H]^+$ 194.0688; found 194.0691 (labeled compound).

Isotopic purity (5D): 76.9% measured by HRMS.

Phenyl(4-phenylphenyl-1-^{13}C)methanone ([$^{13}C_1$]42)

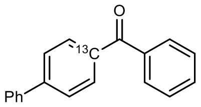

$C_{18}{}^{13}CH_{14}O$
MW: 259.3 g.mol^{-1}
Yellow solid

The title compound was obtained in 85% yield (22.0 mg, 0.085 mmol) using General Procedure G. The crude product was purified with silica gel column chromatography (heptane/ethyl acetate = 50:1).

^{1}H NMR (400 MHz, CDCl$_3$) δ 7.92 – 7.83 (m, 2H), 7.73 – 7.68 (m, 2H), 7.68 – 7.63 (m, 2H), 7.55 – 7.37 (m, 5H), 6.92 (d, *J* = 8.4 Hz, 1H), 3.97 (s, 3H), 3.96 (s, 3H).

^{13}C NMR (100 MHz, CDCl$_3$) δ 196.4 (d, *J* = 55.4 Hz), 145.3 (d, *J* = 8.3 Hz), 140.0, 137.8 (d, *J* = 13.5 Hz), 136.2, 132.4, 131.0 (2C), 130.0 (2C), 129.0 (2C), 128.3 (2C), 128.2, 127.3 (2C), 127.0 (d, *J* = 1.7 Hz, 2C).

IR (cm^{-1}) 1655, 1598, 1446, 1275, 1177, 923, 850, 759, 732, 698, 630.

HRMS (ESI-TOF) m/z calcd for $C_{18}{}^{13}CH_{15}O$ [M+H]$^+$ 260.1157; found 260.1159 (labeled compound).

Phenyl(3-phenylphenyl-1-^{13}C)methanone ([$^{13}C_1$]47)

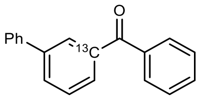

$C_{18}{}^{13}CH_{14}O$
MW: 259.3 g.mol^{-1}
Yellow solid

The title compound was obtained in 70% yield (18.2 mg, 0.07 mmol) using General Procedure G. The crude product was purified with silica gel column chromatography (heptane/ethyl acetate = 50:1).

^{1}H NMR (400 MHz, CDCl$_3$) δ 8.03 (t, *J* = 1.6 Hz, 1H), 7.88 – 7.80 (m, 3H), 7.78 (dt, *J* = 7.7, 1.2 Hz, 1H), 7.65 – 7.54 (m, 4H), 7.54 – 7.44 (m, 4H), 7.41 – 7.36 (m, 1H).

^{13}C NMR (100 MHz, CDCl$_3$) δ 196.7 (d, *J* = 54.7 Hz), 141.4 (d, *J* = 1.0 Hz), 140.1 (d, *J* = 3.7 Hz), 138.2, 137.5 (d, *J* = 13.5 Hz), 132.6, 131.0 (d, *J* = 8.1 Hz), 130.1 (2C), 129.0, 128.9 (d, *J* = 57.6 Hz), 128.7 (d, *J* = 1.3 Hz), 128.6 (d, *J* = 58.8 Hz), 128.3 (2C), 127.8 (2C), 127.2 (2C).

IR (cm^{-1}) 1657, 1594, 1448, 1294, 1252, 953, 758, 716, 698.

HRMS (ESI-TOF) m/z calcd for $C_{18}{}^{13}CH_{15}O$ [M+H]$^+$ 260.1157; found 260.1158 (labeled compound).

d_5-Benzophenone-1-^{13}C ([$^2H_5$, $^{13}C_1$]50)

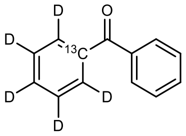

$C_{12}{}^{13}CH_5D_5O$
MW: 188.2 g.mol^{-1}
Yellow solid

The title compound was obtained in 75% yield (14.0 mg, 0.075 mmol) using General Procedure G. The crude product was purified with silica gel column chromatography (heptane/ethyl acetate = 50:1).

^{1}H NMR (400 MHz, $CDCl_3$) δ 7.84 – 7.78 (m, 2H), 7.64 – 7.55 (m, 1H), 7.53 – 7.45 (m, 2H).

^{13}C NMR (100 MHz, $CDCl_3$) δ 196.0 (d, *J* = 54.8 Hz), 137.5, 137.4, 132.4, 130.0 (2C), 128.3 (2C). Due to the D-^{13}C coupling, the signals of carbons binding to D are to low to be recognized.

^{2}H NMR (61 MHz, $CHCl_3$) δ 7.64 (s, 2D), 7.50 (s, 1D), 7.43 (s, 2D).

IR (cm^{-1}) 1656, 1598, 1447, 1361, 1319, 1247, 915, 764, 702, 610, 531.

HRMS (ESI-TOF) m/z calcd for $C_{12}{}^{13}CH_6D_5O$ [M+H]$^+$ 189.1158; found 189.1157 (labeled compound).

Benzophenone-1-^{13}C ([$^{13}C_1$]50)

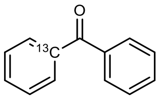

$C_{12}{}^{13}CH_{10}O$
MW: 183.2 g.mol^{-1}
Yellow solid

The title compound was obtained in 74% yield (12.9 mg, 0.074 mmol) using General Procedure G. The crude product was purified with silica gel column chromatography (heptane/ethyl acetate = 50:1).

^{1}H NMR (400 MHz, $CDCl_3$) δ 7.86 – 7.75 (m, 4H), 7.59 (ddd, *J* = 6.8, 2.1, 1.0 Hz, 2H), 7.49 (tdd, *J* = 7.5, 3.7, 2.3 Hz, 4H).

^{13}C NMR (100 MHz, $CDCl_3$) δ 196.8 (d, *J* = 54.9 Hz), 137.6, 132.4 (s+d, *J* = 9.1 Hz, 2C), 130.0 (s+d, *J* = 57.7 Hz, 4C), 128.3 (4C).

IR (cm^{-1}) 1657, 1593, 1439, 1265, 939, 918, 762, 700, 636.

HRMS (ESI-TOF) m/z calcd for $C_{12}{}^{13}CH_{11}O$ [M+H]$^+$ 184.0844; found 184.0846 (labeled compound).

Benzophenone (50)

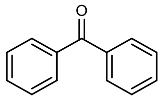

$C_{13}H_{10}O$
MW: 182.2 g.mol^{-1}
Yellow solid

The title compound was obtained in 82% yield (15.0 mg, 0.082 mmol) using General Procedure G.

^{1}H NMR (400 MHz, $CDCl_3$) δ 7.85 – 7.77 (m, 4H), 7.63 – 7.56 (m, 2H), 7.49 (tt, *J* = 6.6, 1.2 Hz, 4H).

^{13}C NMR (100 MHz, $CDCl_3$) δ 196.8, 137.6 (2C), 132.4 (2C), 130.0 (4C), 128.3 (4C).

All analytical data were in good accordance with data reported in the literature.[4]

(S)-(3-(1-Methylpyrrolidin-2-yl)phenyl)(phenyl)methanone (51)

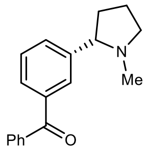

$C_{18}H_{19}ON$
MW: 265.4 g.mol^{-1}
Brown oil

The title compound was obtained in 64% yield (16.9 mg, 0.064 mmol) using General Procedure G. The crude product was purified with silica gel column chromatography (heptane/ethyl acetate = 10:1).

^{1}H NMR (400 MHz, $CDCl_3$) δ 7.82 – 7.77 (m, 3H), 7.69 – 7.63 (m, 1H), 7.61 – 7.59 (m, 2H), 7.52 – 7.40 (m, 3H), 3.27 – 3.20 (m, 1H), 3.13 (t, *J* = 8.2 Hz, 1H), 2.30 (dd, *J* = 17.6, 9.3 Hz, 1H), 2.26 – 2.16 (m, 4H), 2.00 – 1.90 (m, 1H), 1.86 – 1.70 (m, 2H).

^{13}C NMR (100 MHz, $CDCl_3$) δ 196.9, 143.9, 137.8, 137.7, 132.3, 131.5, 130.1 (2C), 129.2, 129.0, 128.4, 128.2 (2C), 71.2, 57.0, 40.5, 35.3, 22.5.

IR (cm^{-1}) 1658, 1598, 1446, 1355, 1315, 1279, 1217, 1179, 1044, 902, 785, 716, 646, 504.

HRMS (ESI-TOF) m/z calcd for $C_{18}H_{20}NO$ $[M+H]^+$ 266.1545; found 266.1547.

(S)-(3-(1-Methylpyrrolidin-2-yl)phenyl-1-^{13}C)(phenyl)methanone ([$^{13}C_1$]51)

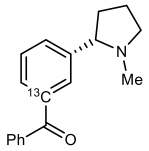

$C_{17}{}^{13}CH_{19}ON$
MW: 266.4 g.mol^{-1}
Brown oil

The title compound was obtained in 65% yield (17.3 mg, 0.065 mmol) using General Procedure G. The crude product was purified with silica gel column chromatography (heptane/ethyl acetate = 10:1).

^{1}H NMR (400 MHz, $CDCl_3$) δ 7.82 – 7.77 (m, 3H), 7.67 – 7.64 (m, 1H), 7.63 – 7.55 (m, 2H), 7.51 – 7.39 (m, 3H), 3.27 – 3.20 (m, 1H), 3.13 (t, *J* = 8.2 Hz, 1H), 2.30 (dd, *J* = 17.6, 9.3 Hz, 1H), 2.25 – 2.16 (m, 4H), 2.02 – 1.88 (m, 1H), 1.83 – 1.72 (m, 2H).

^{13}C NMR (100 MHz, $CDCl_3$) δ 196.8 (d, *J* = 54.7 Hz), 143.2, 137.7, 137.4, 132.4, 131.6 (d, *J* = 8.2 Hz), 130.1 (2C), 129.3 (d, *J* = 58.3 Hz), 129.2 (d, *J* = 57.8 Hz), 128.5 (d, *J* = 1.0 Hz), 128.3 (2C), 71.3 (d, *J* = 3.5 Hz), 56.9, 40.3, 35.1, 22.5.

IR (cm^{-1}) 1655, 1596, 1447, 1314, 1267, 1179, 1045, 901, 814, 785, 712, 643.

HRMS (ESI-TOF) m/z calcd for $C_{17}{}^{13}CH_{20}ON$ $[M+H]^+$ 267.1579; found 267.158. (labeled compound)

9. Unsuccessful substrates of ^{15}N labeling of pyridines

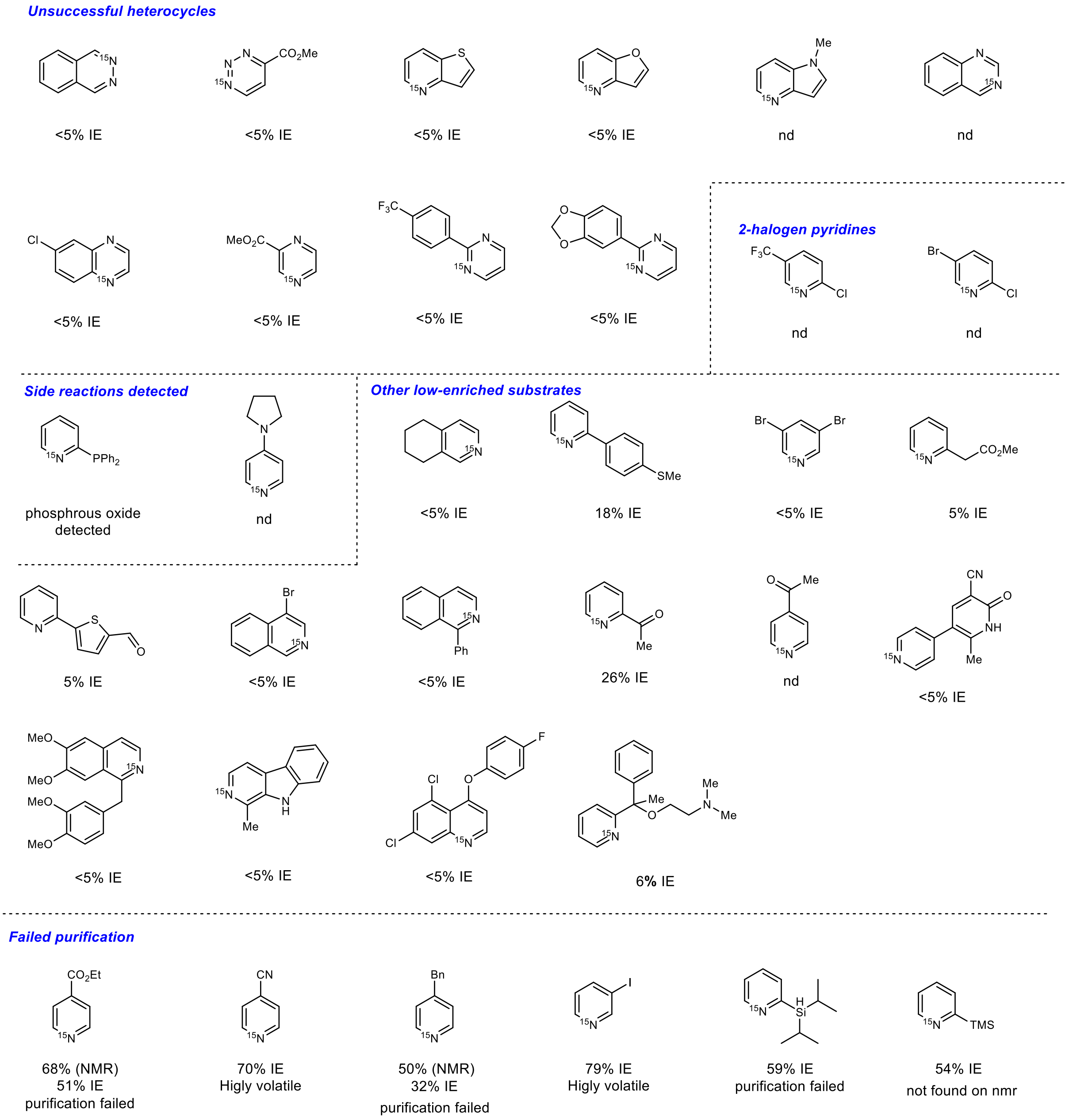

10. Reduction of [$^{2}H_5$, $^{13}C_1$]benzophenone

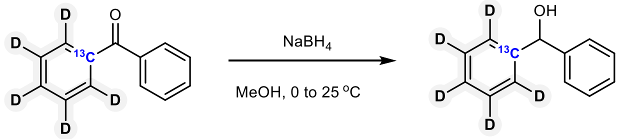

[$^{2}H_5$, $^{13}C_1$]50 **[$^{2}H_5$, $^{13}C_1$]52**, 95%

To a solution of [$^{2}H_5$, $^{13}C_1$]benzophenone **[$^{2}H_5$, $^{13}C_1$]50** (7.00 mg, 0.037 mmol, 1.00 equiv.) in methanol (0.50 mL), sodium borohydride (2.80 mg, 0.074 mmol, 2.00 equiv.) was added at 0 °C. The reaction was then stirred at 25 °C for 2 h. After the full conversion of the starting materiel (monitored by TLC), the reaction was quenched with saturated aqueous ammonium chloride solution (2.00 mL). The mixture was then extracted with dichloromethane (3 x 3.00 mL). The combined organic phase was then dried over magnesium sulphate. After concentration under vacuum, the crude product was afforded. Purification by a short column chromatography (heptane/ethyl acetate = 3:1) gave the desired product (6.70 mg, 0.035 mmol, 95%) as a white solid.

Characterizations of **[$^{2}H_5$, $^{13}C_1$]52**:

^{1}H NMR (400 MHz, $CDCl_3$) δ 7.41 – 7.32 (m, 4H), 7.30 – 7.25 (m, 1H), 5.86 (d, *J* = 3.8 Hz, 1H), 2.23 (s, 1H).

^{13}C NMR (100 MHz, $CDCl_3$) δ 143.6, 128.5, 127.6, 126.5, 29.7. Due to the D-^{13}C coupling, the signals of carbons binding to D are to low to be recognized.

HRMS (ESI-TOF) m/z calcd for $C_{12}{}^{13}CH_6D_5$ [M +H-H_2O]$^+$ 173.1208; found 173.1207 (labeled compound).

11. ^{13}N Radioactive experiments

Supplementary Figure 4: proof-of-concept radioactive ^{13}N-labeling of pyridines.

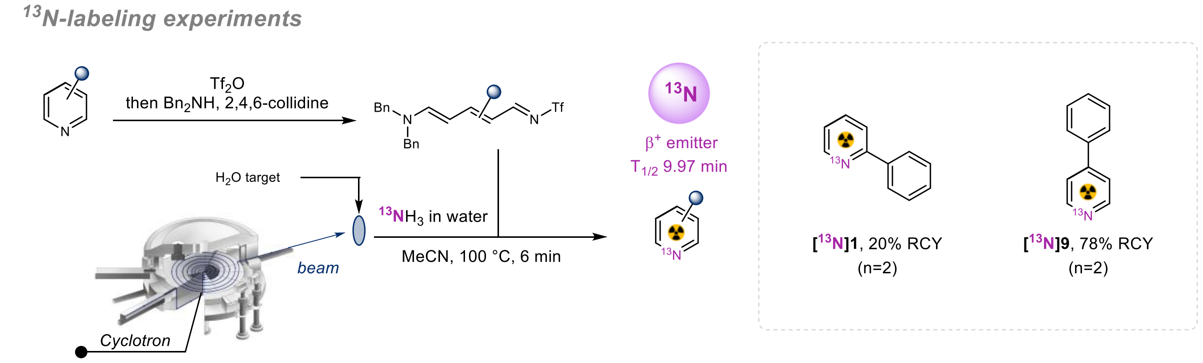

Aqueous [^{13}N]NH_3 was produced on a GE PETtrace 880 cyclotron by proton irradiation of a liquid target containing [^{16}O]H_2O + ethanol (0.03% v/v) via the $^{16}O(p,\alpha)^{13}N$ nuclear reaction at a beam current of 15 µA for 1 min. Radiolabelling reactions were performed using an Eckert and Zielgler Modular-Lab radiosynthesizer in a lead shielded hot-cell. Analytical HPLC was performed using an Agilent 1260 Infinity system with an in-line LabLogic Flow-RAM radiodetector and fitted with a Phenomenex™ Luna C18(2) column (5 µm, 100 Å, 250 x 4.6 mm) under a gradient elution of water or ammonium formate (50 mM, pH 4) vs acetonitrile (1 mL/min, 20-90% gradient over 10 min).

Aqueous [^{13}N]NH_3 (1.6 mL, <1 GBq) was delivered to the hotcell under a positive pressure of N_2 gas and trapped on a solid-phase cation exchange cartridge (Waters™ Sep-Pak Accell Plus CM Plus Light Cartridge, 130 mg, WAT023531), washed with water (2.0 mL) and eluted with saline solution (0.9% w/v, 0.5 mL) into a 2 mL glass vial containing a stirrer and a solution of precursor (**Im1** or **Im8**, 5 µmol), triethylamine (5 µL, 30 µmol) in acetonitrile (0.5 mL), and fitted with rubber septum crimp cap. The reaction mixture was heated with stirring at 100°C for 6 min then the solution was cooled, removed from the hotcell, its activity measured using a Capintec™ CRC-55tR dose calibrator and an aliquot removed for analysis by radio-analytical HPLC. Product identity was confirmed by coinjection with the non-radiolabelled product, whereby coelution of the product UV and radio peaks was observed. Non-isolated product radiochemical yield (RCY) was calculated based on radio-HPLC as the fraction of the area of the product peak vs. the total area of all radioactive peaks.

<u>Synthesis of [^{13}N]2-phenylpyridine **[^{13}N]1**</u>

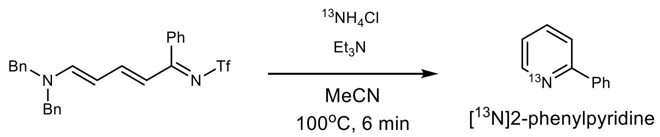

Product activity = 644 MBq (decay corrected). Total synthesis time from end of bombardment = 22 min. Non-isolated RCY = 20% (n=2)

*Note: The decay corrected dose of the crude product is 644 MBq and non-isolated RCY is 20%, thus 129 MBq of **[^{13}N]1** is present.*

Supplementary Figure 5: HPLC chromatogram of crude product. Red line: gamma chromatogram. Blue line: UV chromatogram recorded at 254 nm.

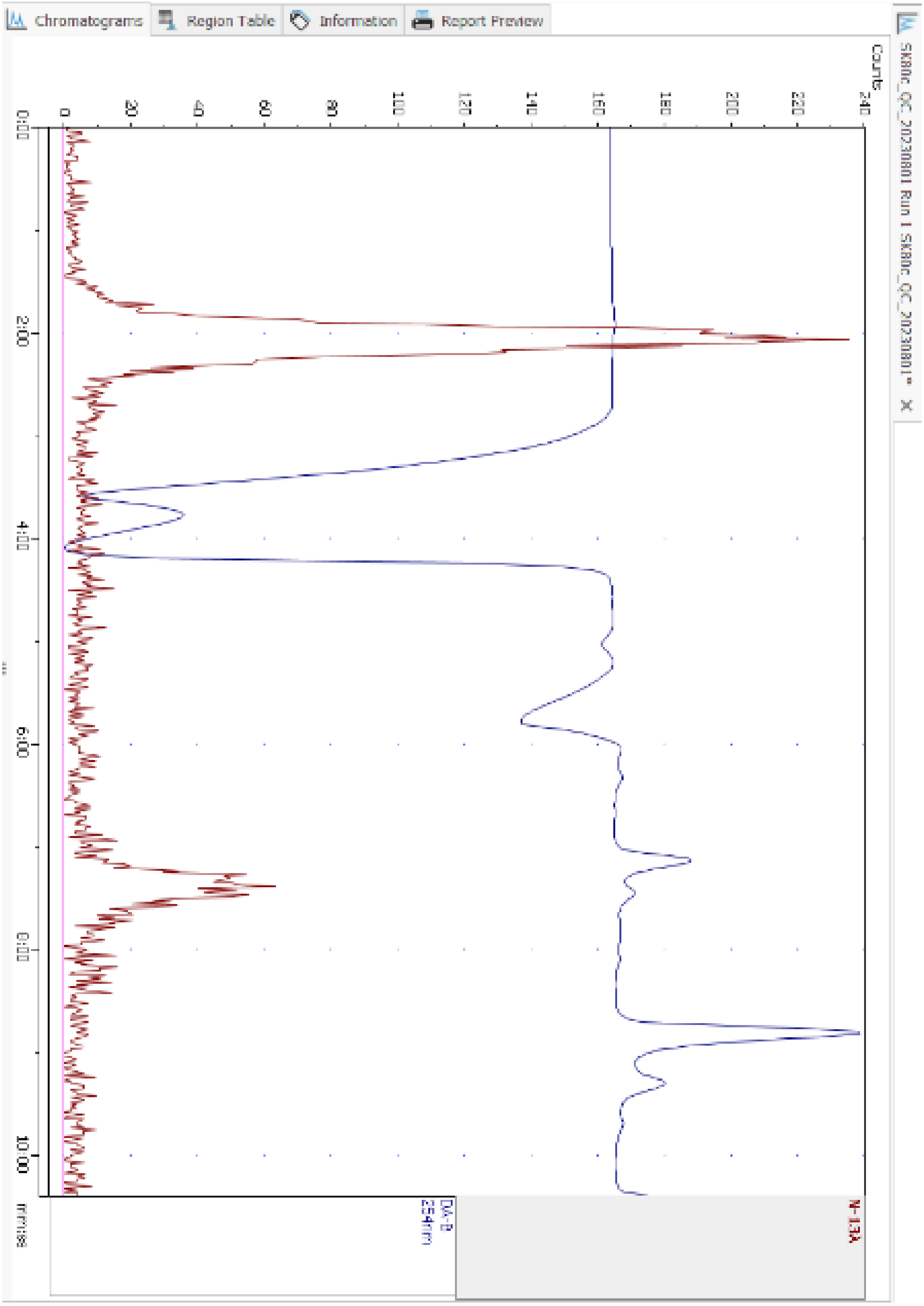

Supplementary Figure 6: HPLC chromatogram of crude product + unlabeled sample of **1**. Red line: gamma chromatogram. Blue line: UV chromatogram recorded at 254 nm.

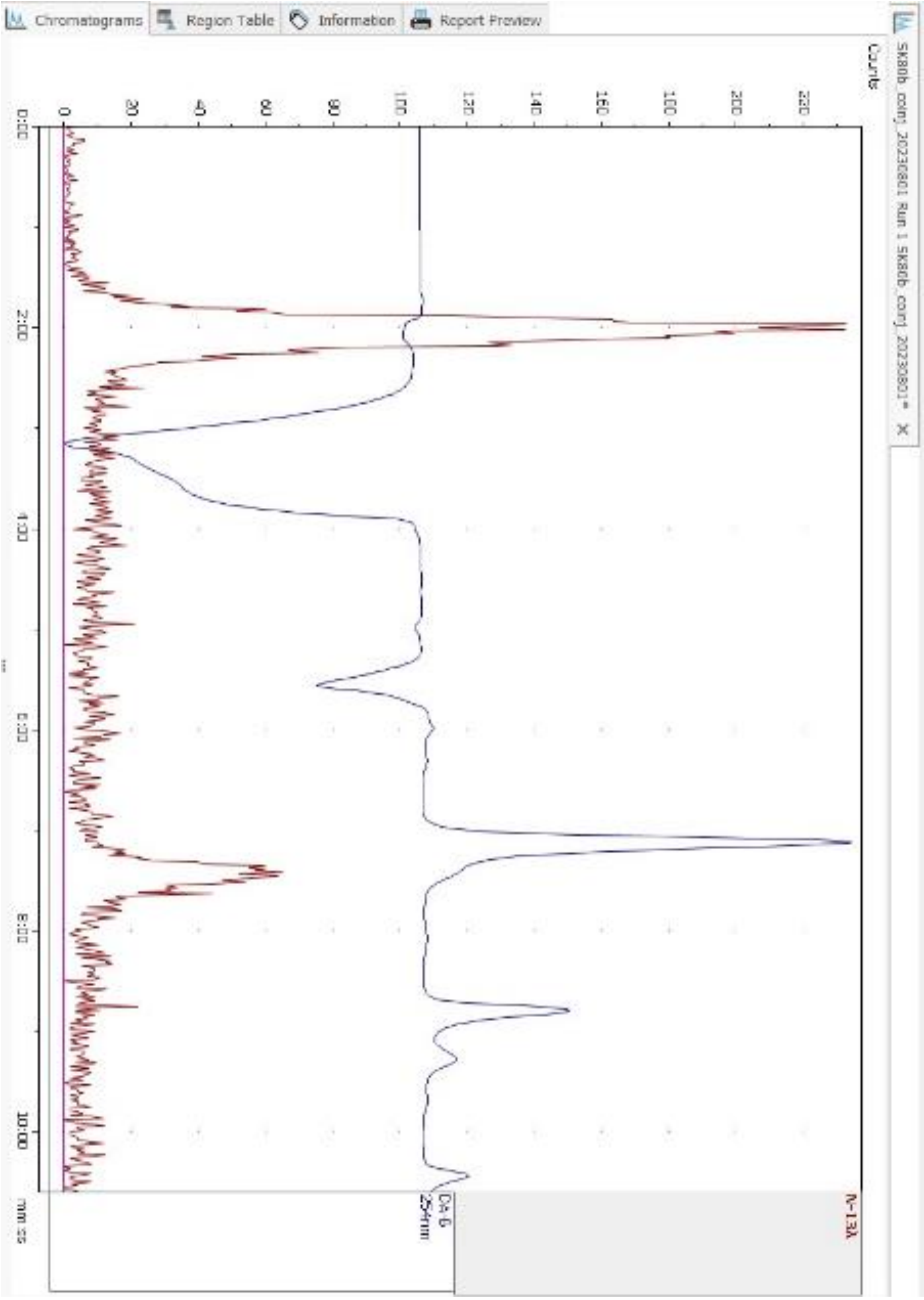

Synthesis of [^{13}N]4-phenylpyridine **[^{13}N]8**

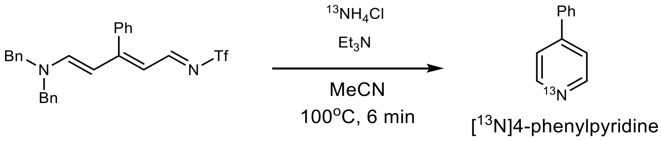

Product activity = 576 MBq (decay corrected). Total synthesis time from end of bombardment = 22 min. Non-isolated RCY = 78% (n=2)

Note: The decay corrected dose of the crude product is 576 MBq and non-isolated RCY is 78%, thus 449 MBq of ***[^{13}N]8*** *is present.*

Supplementary Figure 7: HPLC chromatogram of crude product. Red line: gamma chromatogram. Blue line: UV chromatogram recorded at 254 nm.

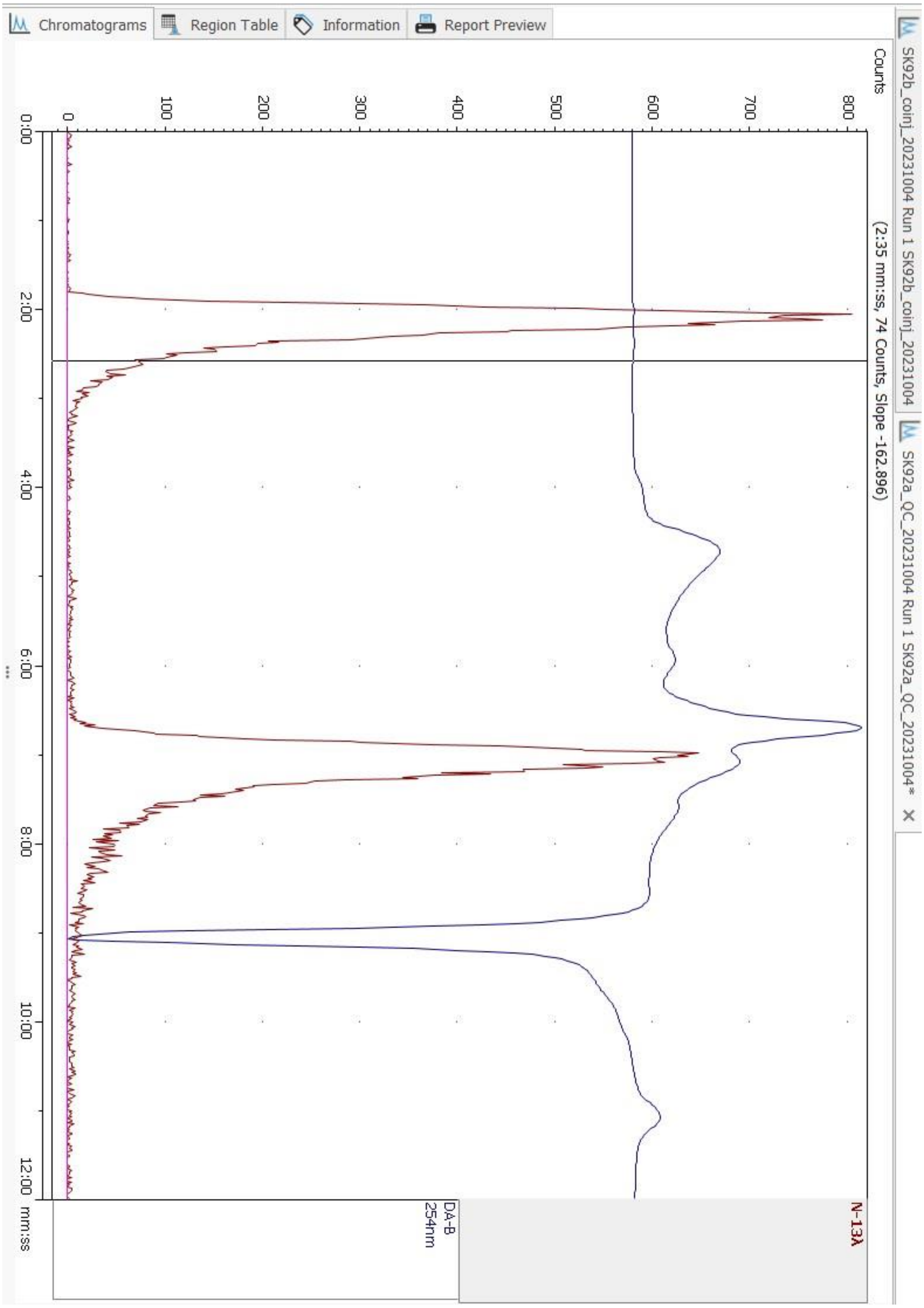

Supplementary Figure 8: HPLC chromatogram of crude product + unlabelled sample of **8**. Red line: gamma chromatogram. Blue line: UV chromatogram recorded at 254 nm.

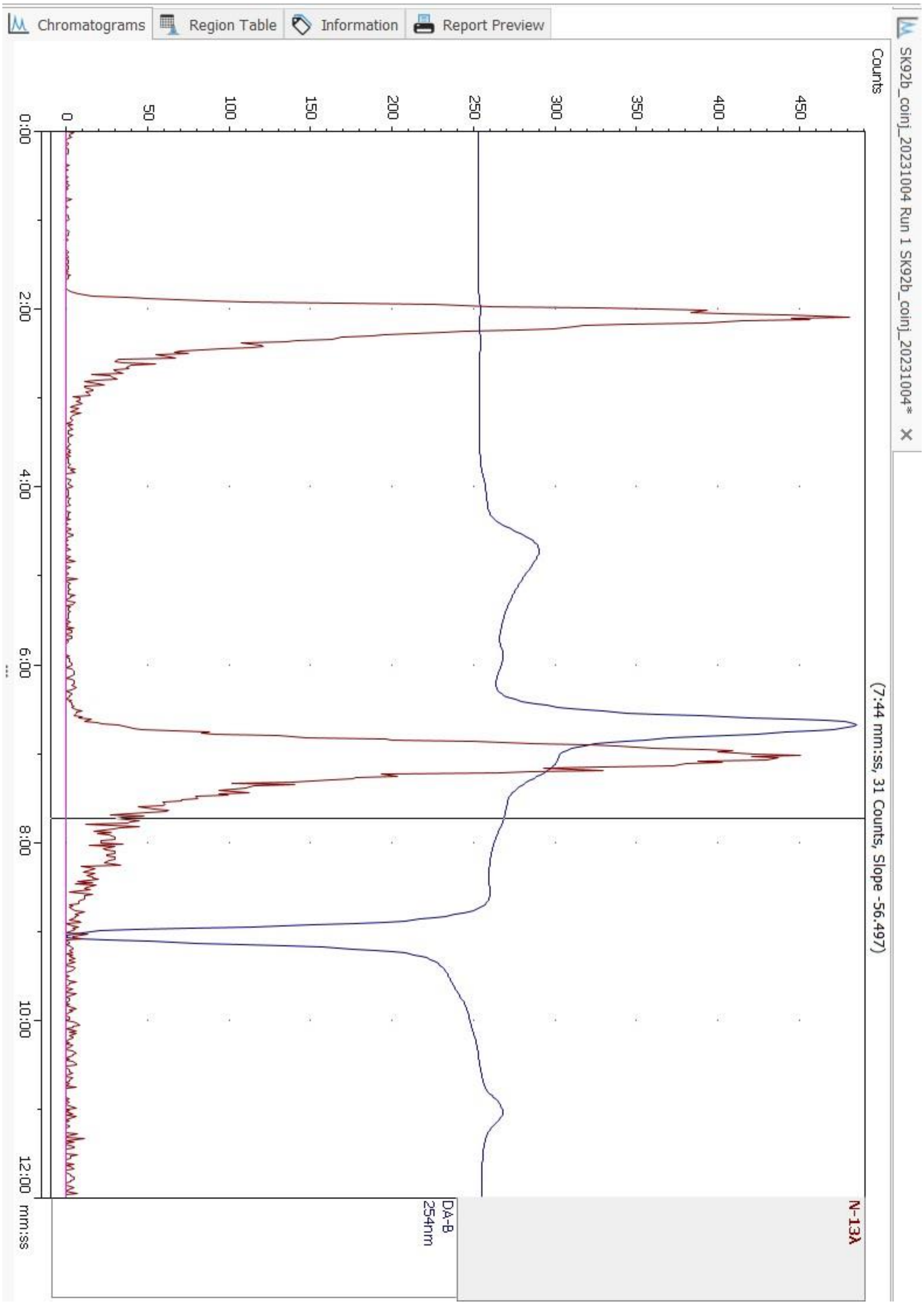

12. Crystallography

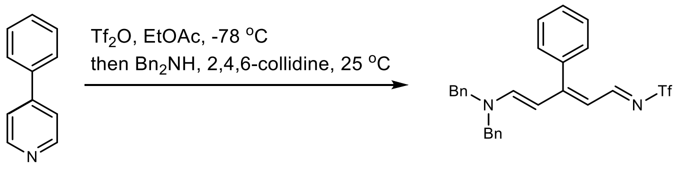

9 **Im9**, 70%

Compound **Im9** was prepared according to the procedure reported by McNally *et al*.[5] The crystal was obtained by vapour diffusion of a *n*-hexane/ethyl acetate system.

The data were collected at 100(2) K on a Bruker D8 Quest diffractometer equipped with an Incoatec microfocus source (IµS 3.0 Mo, λ = 0.71073 Å) and a PHOTON III area detector, and operated through the APEX3 software.[6] The crystal was mounted on a Mitegen micromount with a protective coating of Paratone-N oil (Hampton Research). The data were processed with SAINT,[7] and the structure was solved by intrinsic phasing with SHELXT,[8] and refined by full-matrix least-squares on F^2 with SHELXL,[9] using the ShelXle interface.[10] All non-hydrogen atoms were refined with anisotropic displacement parameters. The hydrogen atoms were introduced at calculated positions and were treated as riding atoms with an isotropic displacement parameter equal to 1.2 times that of the parent atom. The molecular plot was drawn with ORTEP-3.[11]

Crystal data for **Im9**: $C_{26}H_{23}F_3N_2O_2S$, M = 484.52, triclinic, space group $P\bar{1}$, a = 8.8569(5), b = 12.4246(7), c = 12.6822(8) Å, α = 105.641(2), β = 102.682(2), γ = 108.089(2)°, V = 1205.35(12) Å^3, Z = 2. Refinement of 307 parameters on 4576 independent reflections out of 46097 measured reflections (R_{int} = 0.073) led to $R1$ = 0.049, $wR2$ = 0.131, S = 1.043, $\Delta\rho_{min}$ = –0.41, $\Delta\rho_{max}$ = 0.30 e Å^{-3}.

Supplementary Figure 9. ORTEP view of **Im9** with displacement ellipsoids shown at the 50% probability level and hydrogen atoms omitted.

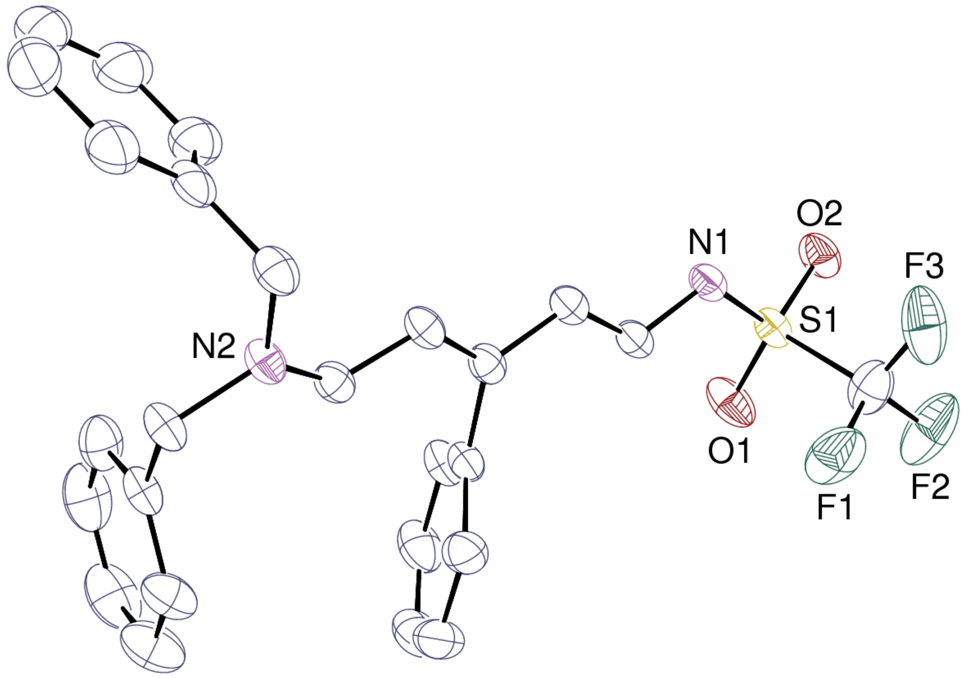

13. NMR spectra and isotopic enrichment

12.1 Zincke salts

1-(2,4-Dinitrophenyl)-3-phenylpyridin-1-ium 4-methylbenzenesulfonate (S1)

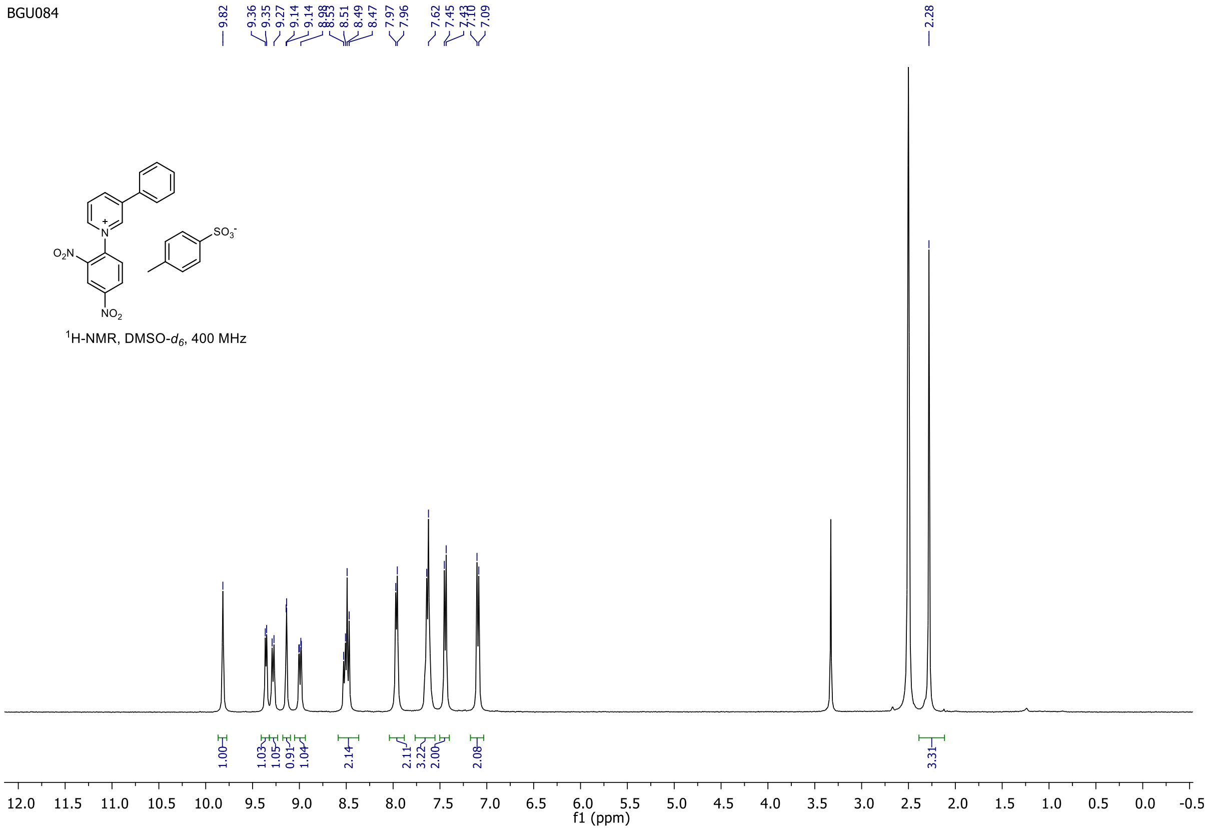

1-(2,4-Dinitrophenyl)-3-(1,3-dioxoisoindolin-2-yl)pyridin-1-ium 4-methylbenzenesulfonate (S2)

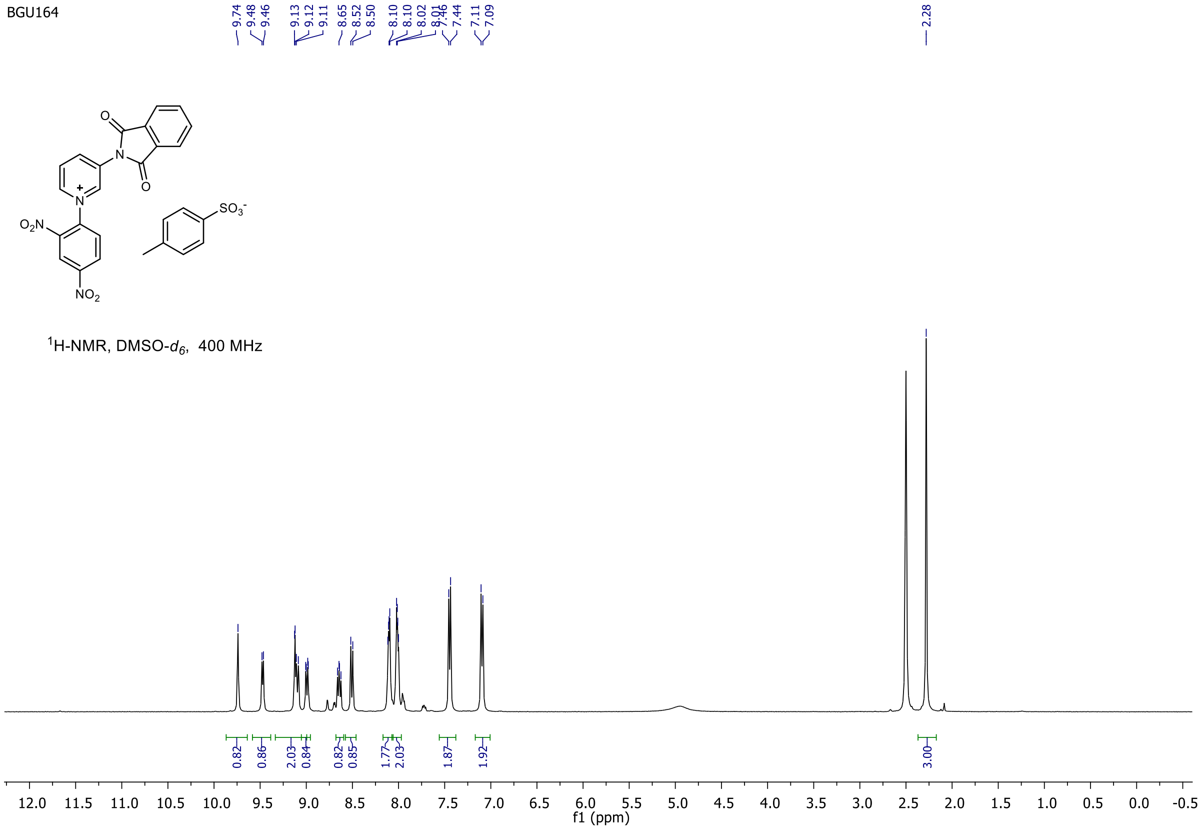

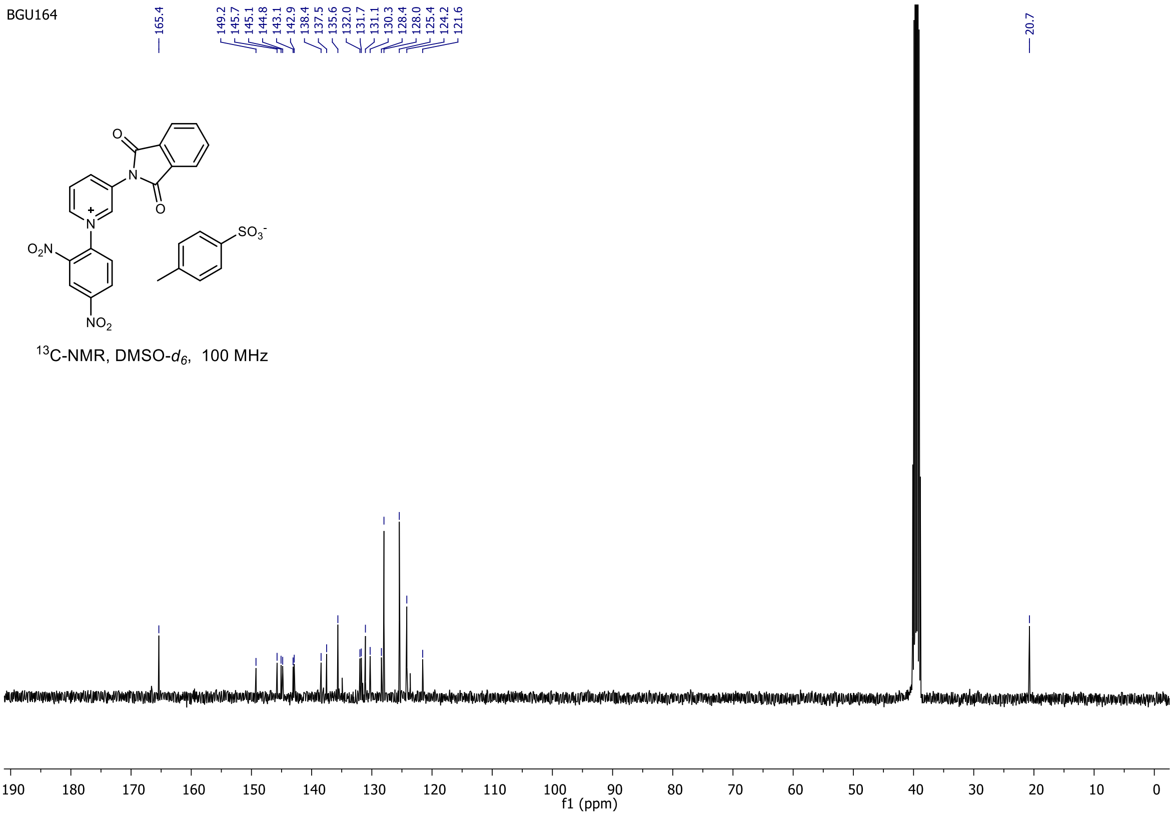

3-Carbamoyl-1-(2,4-dinitrophenyl)pyridin-1-ium 4-methylbenzenesulfonate (S3)

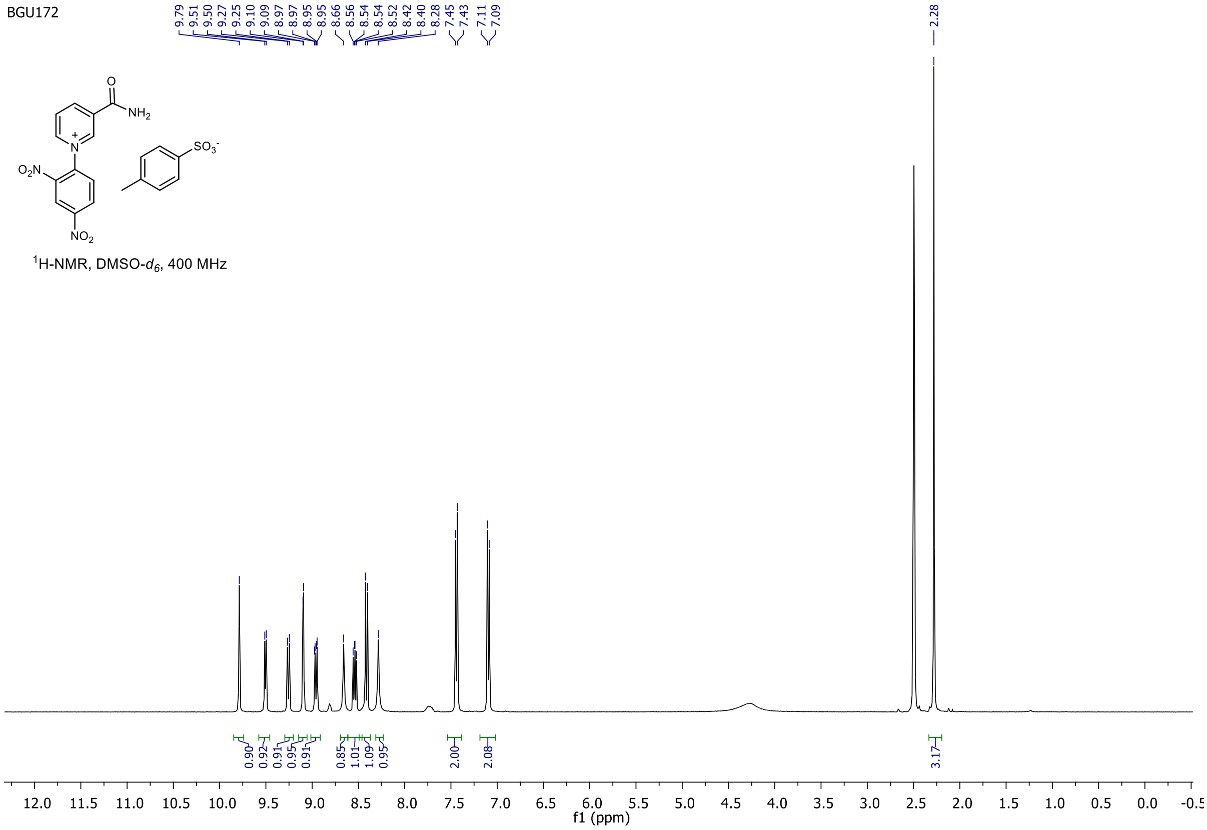

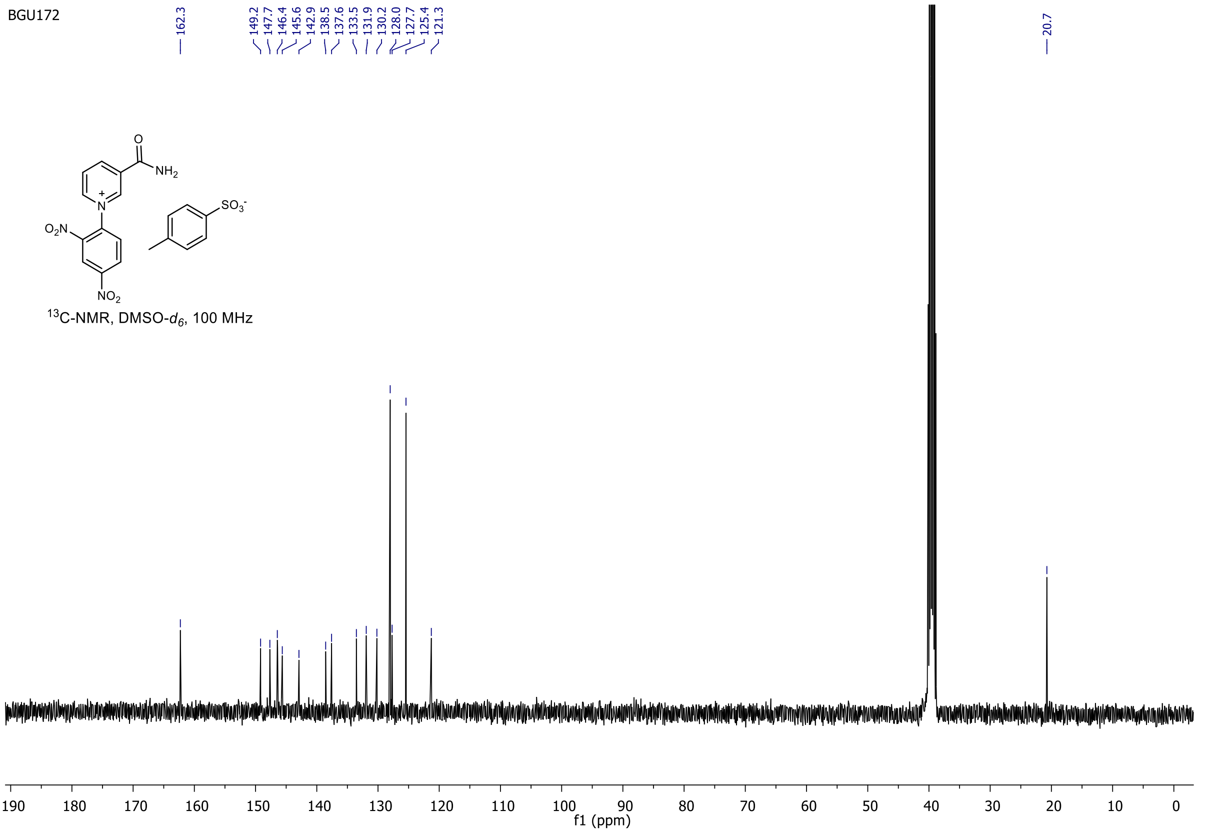

1-(2,4-Dinitrophenyl)-3-(methoxycarbonyl)pyridin-1-ium 4-methylbenzenesulfonate (S4)

BGU146

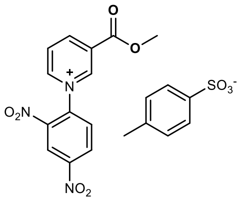

^{1}H-NMR, DMSO-d_6, 400 MHz

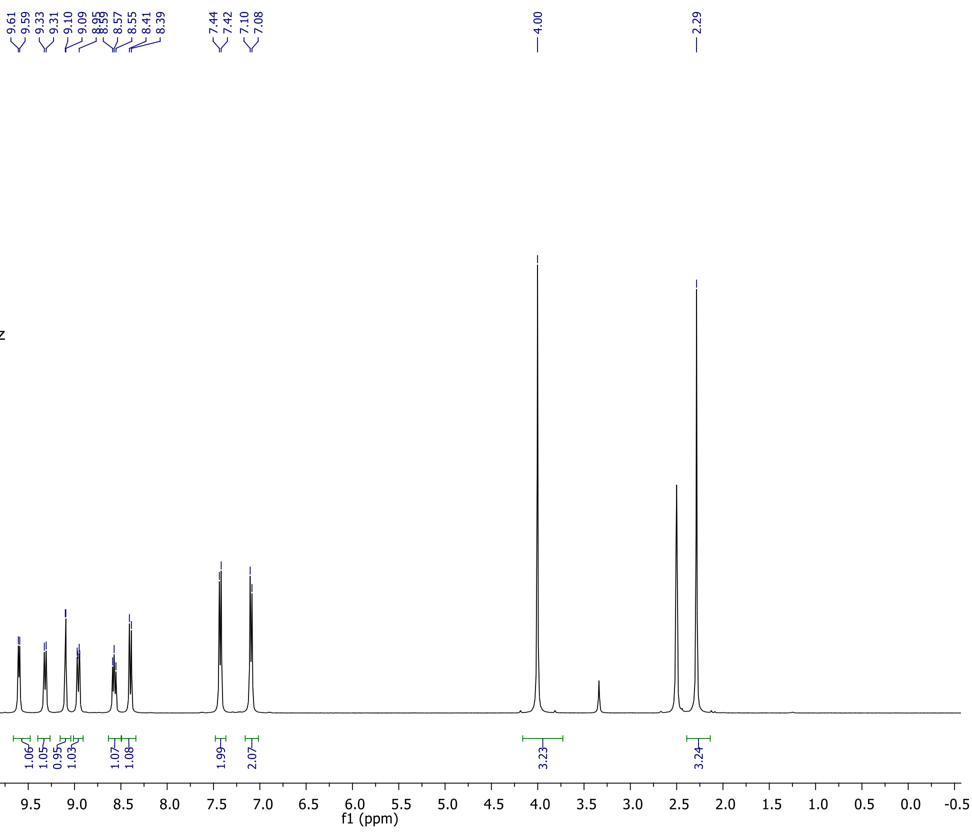

13.2 ^{15}*N*-labeled pyridines

2-Phenylpyridine-1-^{15}N ([^{15}N]1)

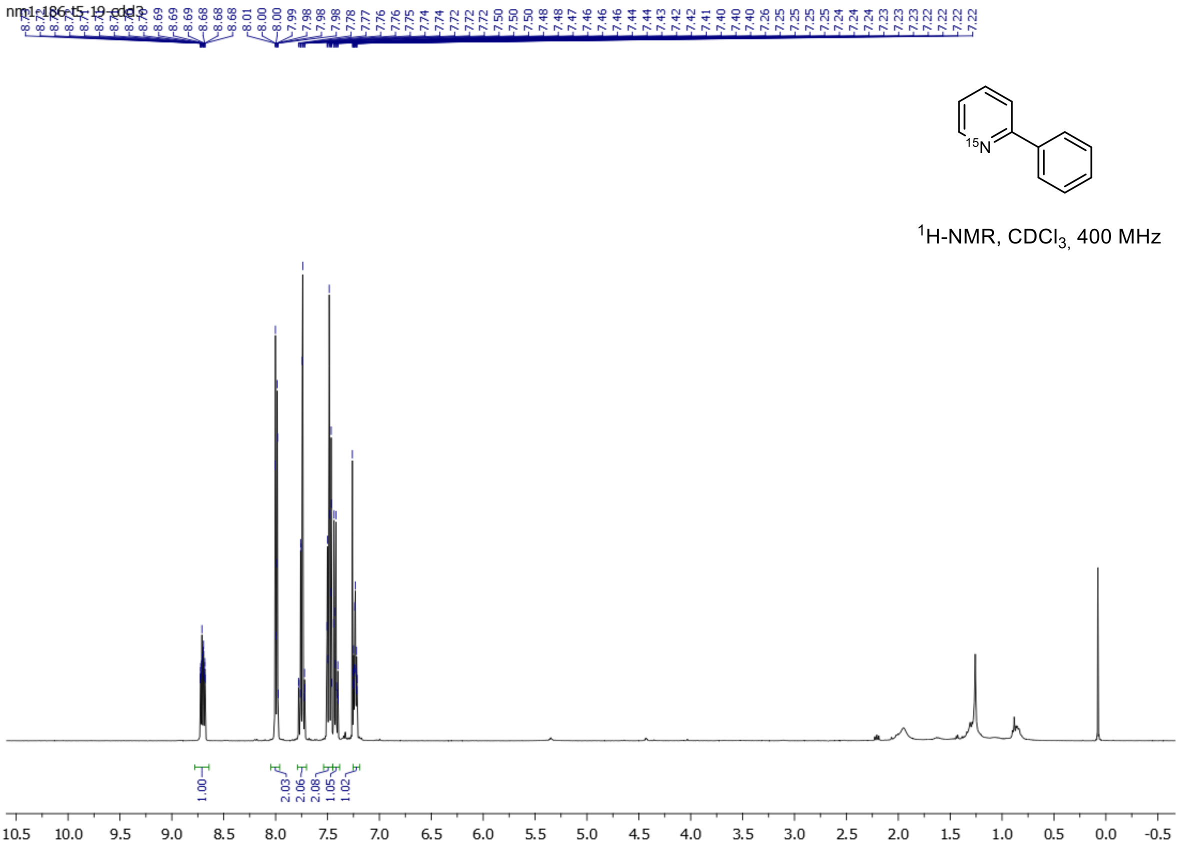

^{1}H-NMR, $CDCl_3$, 400 MHz

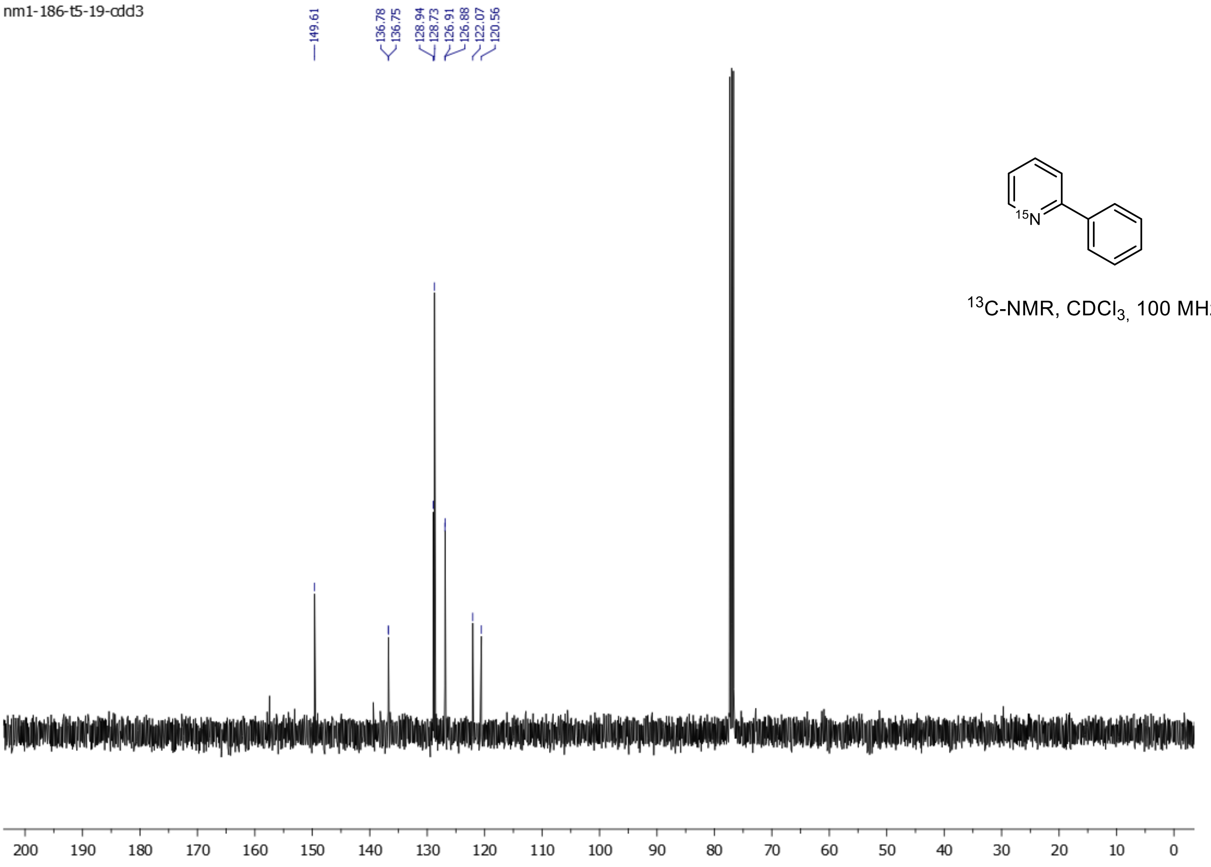

^{13}C-NMR, $CDCl_3$, 100 MHz

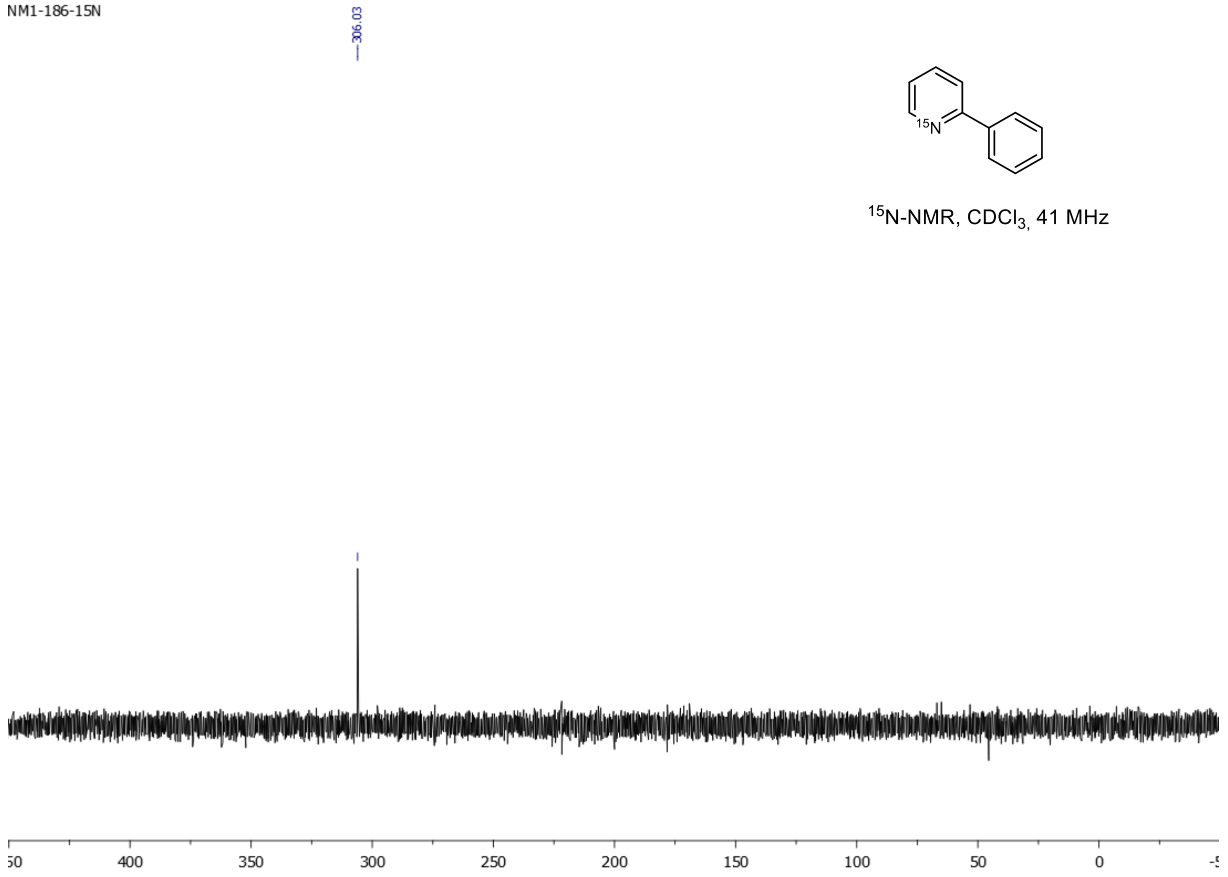
NM1-186-15N
306.03
15N
15N-NMR, CDCl3, 41 MHz
50
400
350
300
250
200
150
100
50
0

Isotopic Enrichment

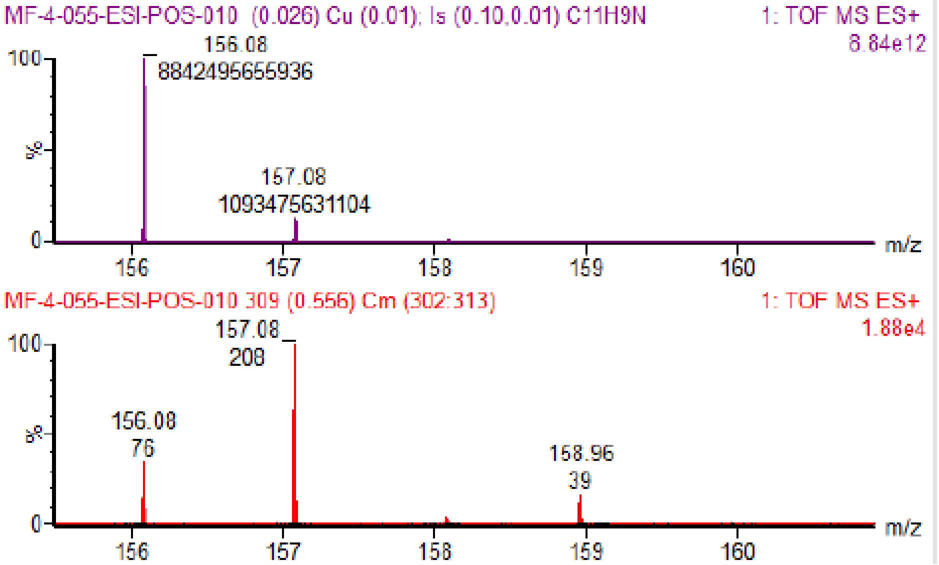

theorical isotopic distribution

	M	M+1	M+2	M+3	M+4	M+5	M+6	M+7
m/z	156	157	158	159	160	161	162	163
%	100	12,4	0,6	0	0	0	0	0

Enrichment calculation

Isotopomer	m/z	Area	natural isotope correction	Corrected area	Isotopic purity (%)
0	156	76	0,00	76,00	29,01
1	157	208	9,42	198,58	75,81
2	158	11	0,46	-14,08	-5,37
3	159	1	0,00	1,55	0,59
4	160	0	0,00	-0,11	-0,04
5	161	0	0,00	0,00	0,00
6	162	0	0,00	0,00	0,00
7	163	0	0,00	0,00	0,00
8	164	0	0,00	0,00	0,00
9	165	0	0,00	0,00	0,00
10	166	0	0,00	0,00	0,00
11	167	0	0,00	0,00	0,00
Total		296		261,95	100,00

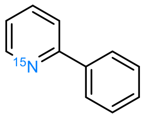

IE obtained following procedure A

% Isotopic enrichment : 75,8

Isotopic Enrichment

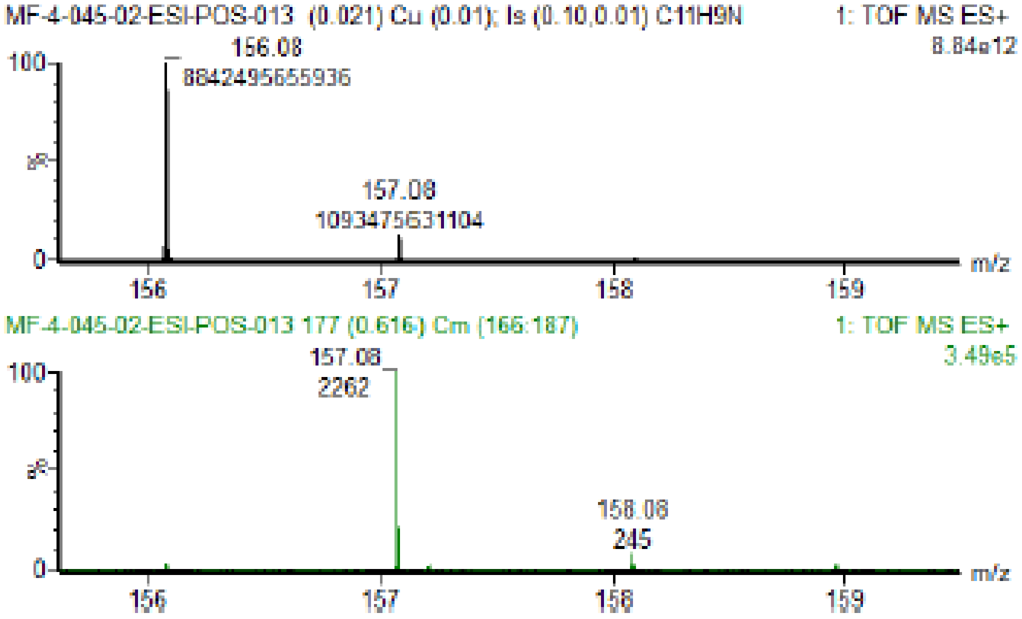

theorical isotopic distribution

	M	M+1	M+2	M+3	M+4	M+5	M+6	M+7
m/z	157	158	159	160	161	162	163	164
%	100,0	12,4	0,6	0,0	0,0	0,0	0,0	0,0

Enrichment calculation

Isotopomer	m/z	Area	natural isotope correction	Corrected area	Isotopic purity (%)
0	157	101	0,00	101,00	4,37
1	158	2262	12,52	2249,48	97,32
2	159	245	0,61	-34,54	-1,49
3	160	4	0,00	-5,21	-0,23
4	161	0	0,00	0,85	0,04
5	162	0	0,00	-0,07	0,00
6	163	0	0,00	0,00	0,00
7	164	0	0,00	0,00	0,00
8	165	0	0,00	0,00	0,00
9	166	0	0,00	0,00	0,00
10	167	0	0,00	0,00	0,00
11	168	0	0,00	0,00	0,00
Total		2612		2311,50	100,00

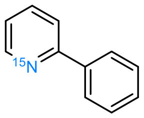

IE obtained following procedure D

% Isotopic enrichment : 97,3

2-(4-Bromophenyl)pyridine-1-^{15}N ([^{15}N]2)

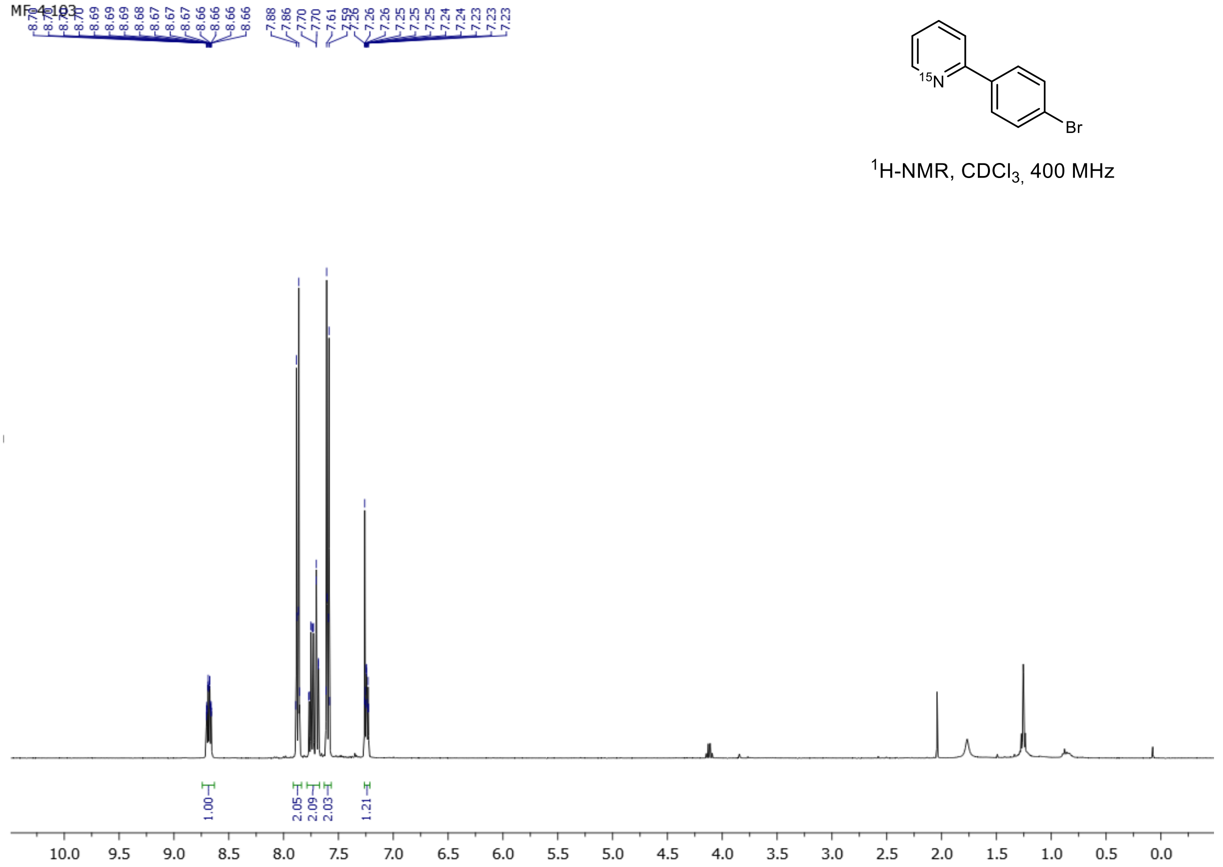

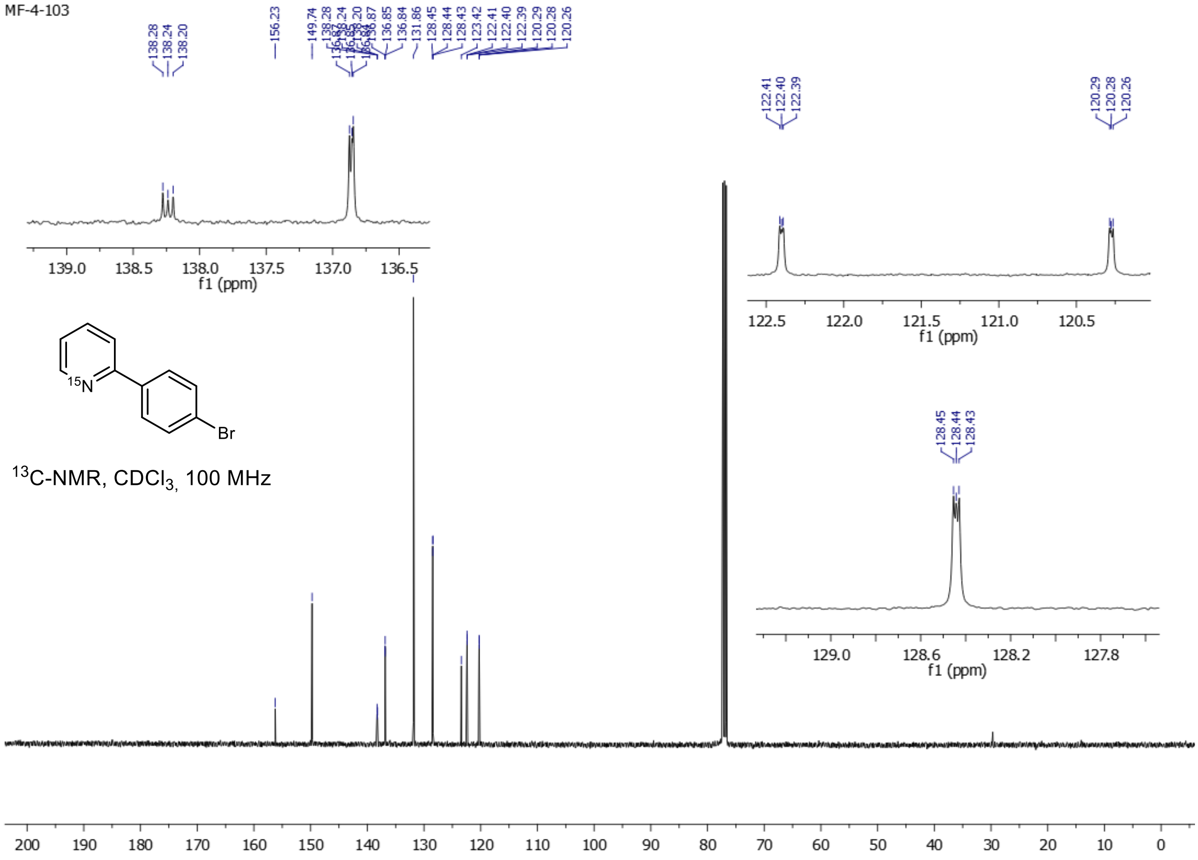

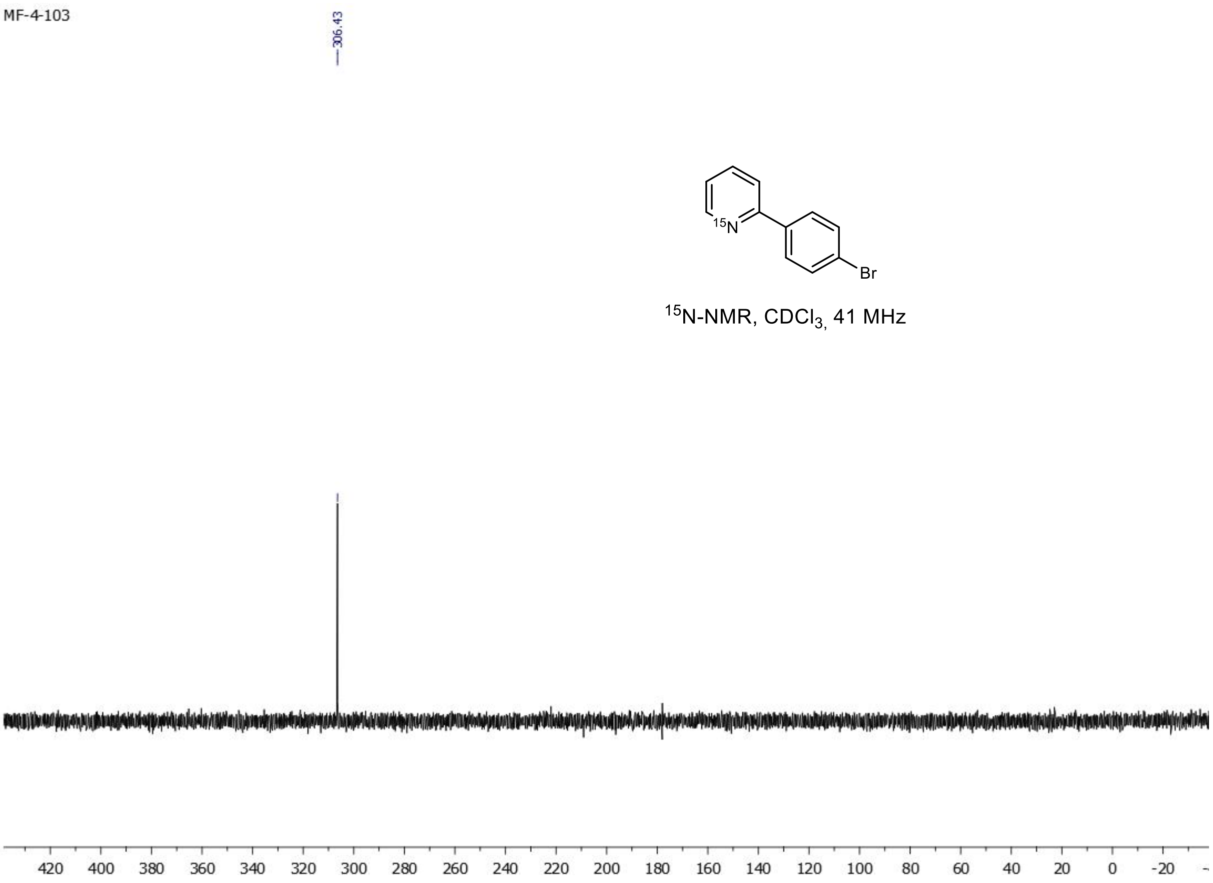
MF-4-103
306.43
15N
Br
15N-NMR, CDCl3, 41 MHz
420 400 380 360 340 320 300 280 260 240 220 200 180 160 140 120 100 80 60 40 20 0 -20

Isotopic Enrichment

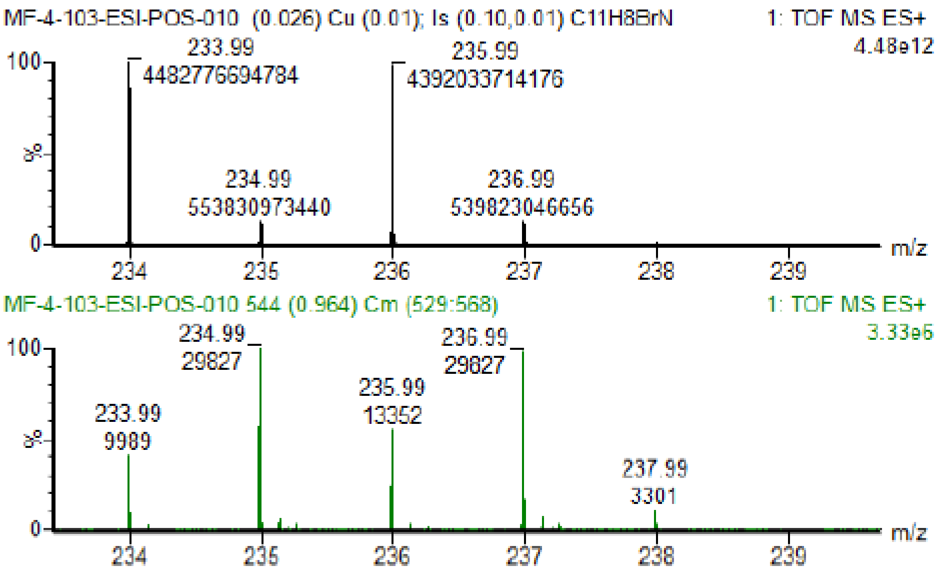

theorical isotopic distribution

	M	M+1	M+2	M+3	M+4	M+5	M+6	M+7
m/z	234	235	236	237	238	239	240	241
%	100	12,4	98,2	12,1	0,6	0	0	0

Enrichment calculation

Isotopomer	m/z	Area	natural isotope correction	Corrected area	Isotopic purity (%)
0	234	9989	0,00	9983,69	25,71
1	235	29827	1240,93	28631,52	73,50
2	236	13352	9827,39	3,14	0,01
3	237	29827	1210,91	458,88	1,18
4	238	3301	60,05	-288,87	-0,74
5	239	157	0,00	-385,34	-0,99
6	240	4	0,00	285,62	0,74
7	241	0	0,00	329,45	0,85
8	242	0	0,00	-278,82	-0,72
9	243	0	0,00	-274,33	-0,71
10	244	0	0,00	272,24	0,70
11	245	0	0,00	219,37	0,56
Total		86457		38956,55	100,08

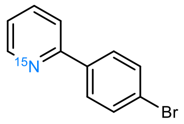

% Isotopic enrichment : 73,5

2-(4-Ttrifluoromethyl)phenyl)pyridine-1-^{15}N ([^{15}N]3)

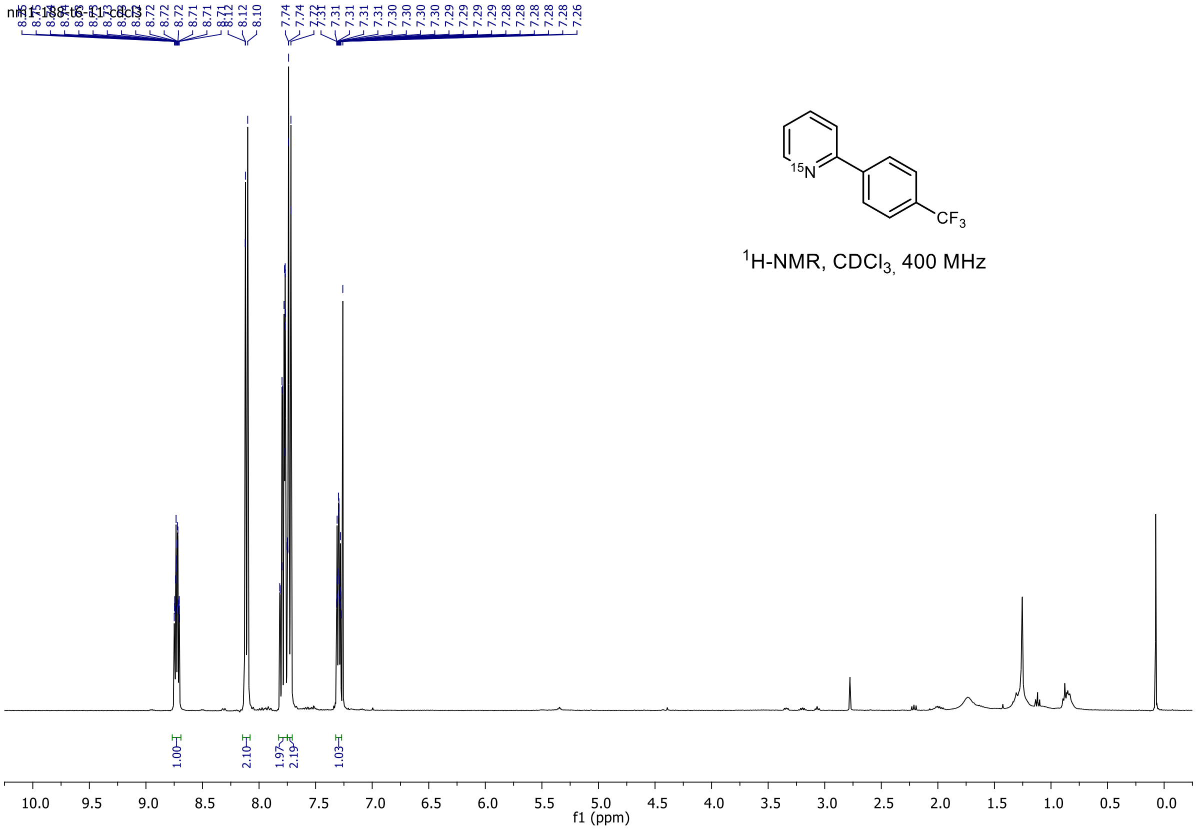

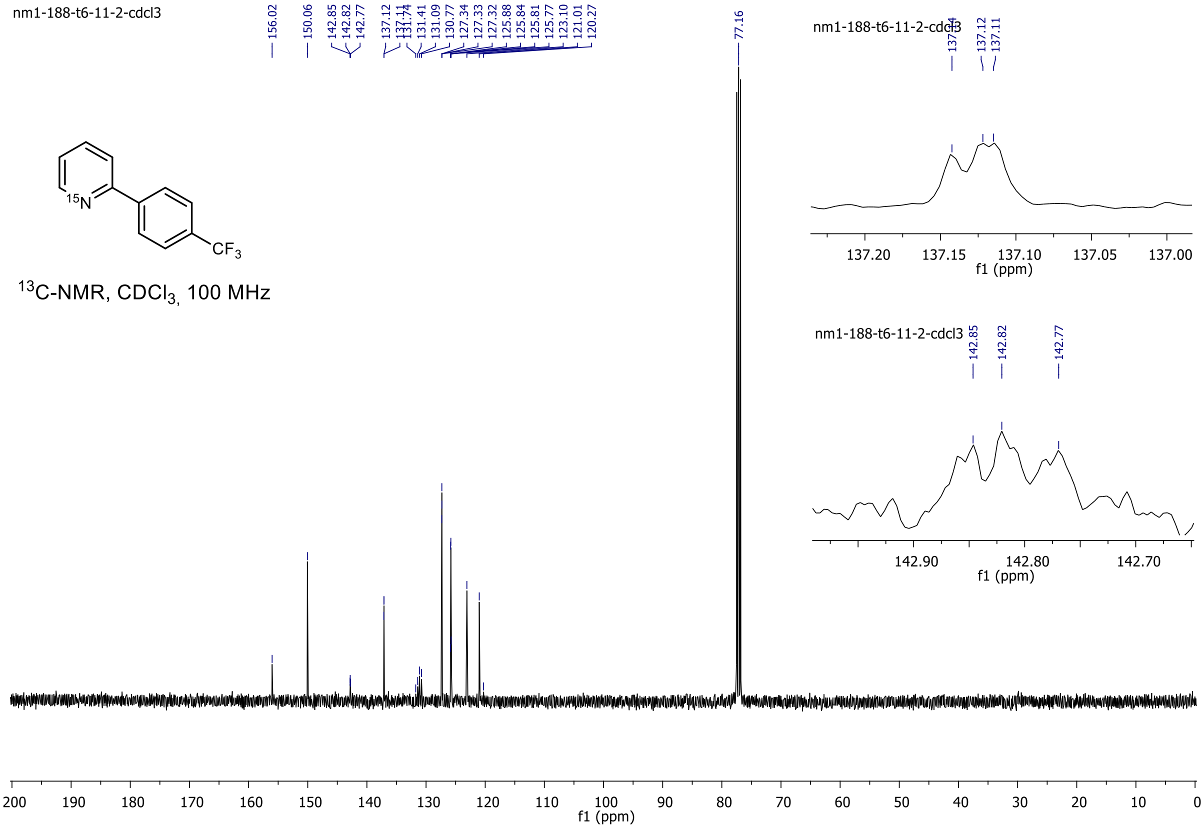

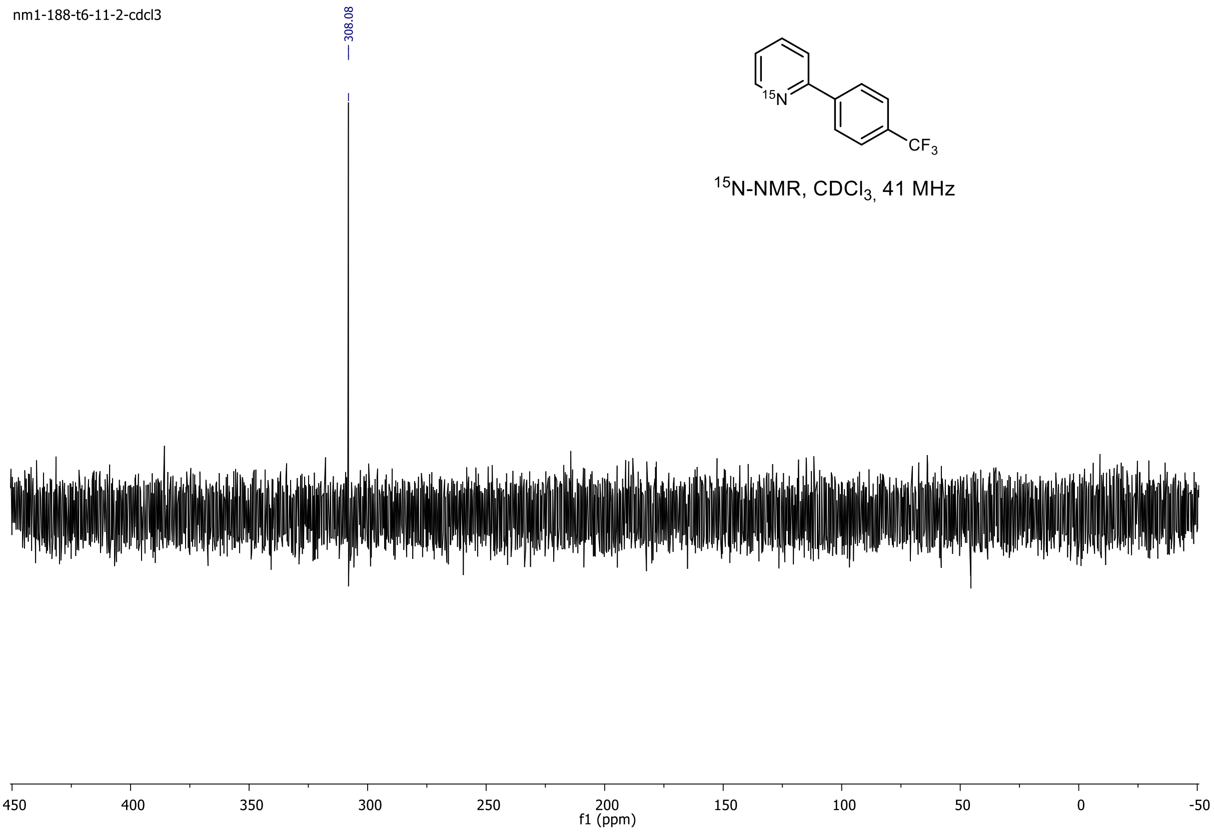
nm1-188-t6-11-2-cdcl3
308.08
15N
CF3
15N-NMR, CDCl3, 41 MHz
450
400
350
300
250
200
150
100
50
0
-50
f1 (ppm)

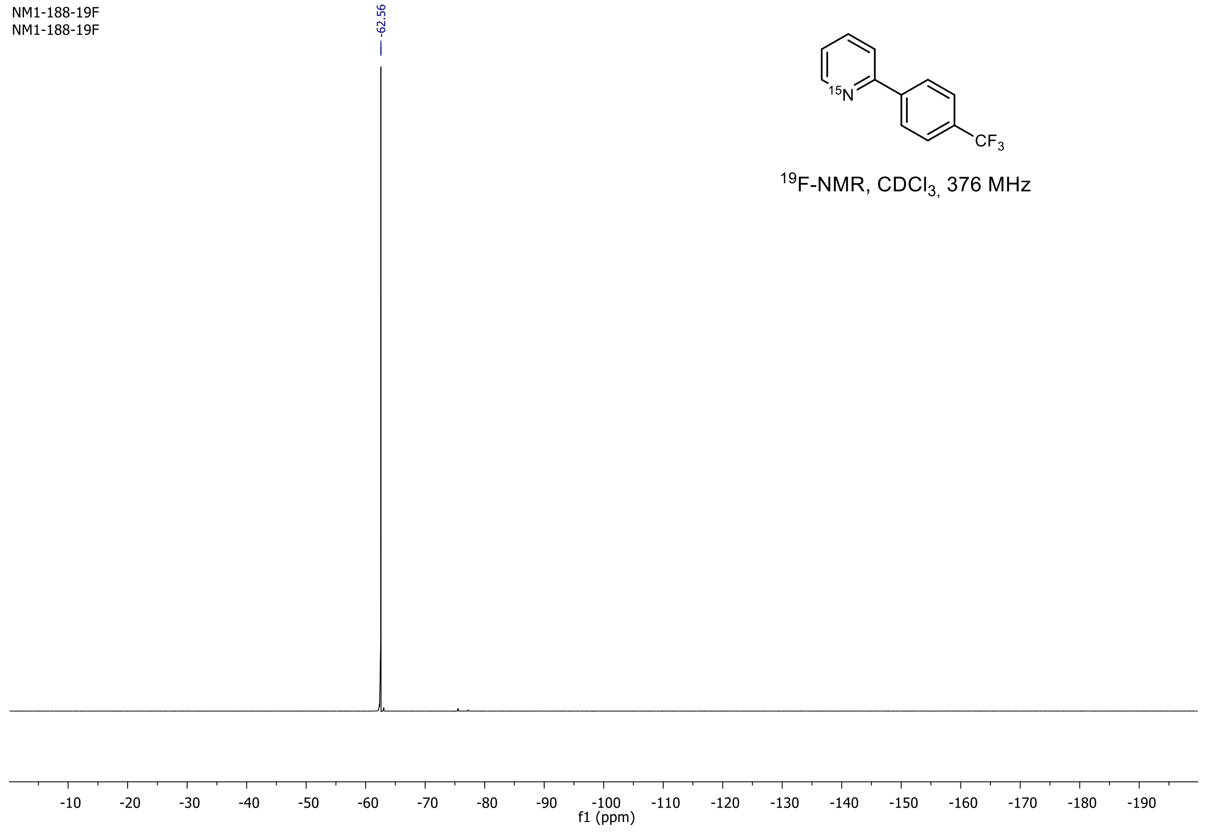
NM1-188-19F
NM1-188-19F
-62.56
15N
CF3
19F-NMR, CDCl3, 376 MHz
-10
-20
-30
-40
-50
-60
-70
-80
-90
-100
-110
-120
-130
-140
-150
-160
-170
-180
-190
f1 (ppm)

Isotopic Enrichment

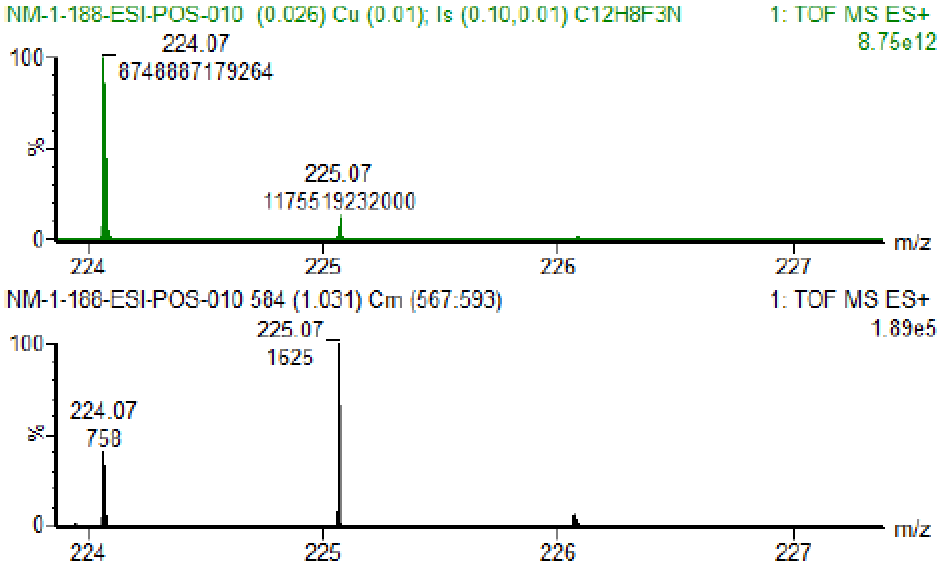

theorical isotopic distribution

	M	M+1	M+2	M+3	M+4	M+5	M+6	M+7
m/z	224	225	226	227	228	229	230	231
%	100,0	13,5	0,8	0,0	0,0	0,0	0,0	0,0

Enrichment calculation

Isotopomer	m/z	Area	natural isotope correction	Corrected area	Isotopic purity (%)
0	224	758	0,00	758,00	34,27
1	225	1625	102,33	1522,67	68,85
2	226	139	6,06	-72,62	-3,28
3	227	6	0,00	3,62	0,16
4	228	0	0,00	0,09	0,00
5	229	0	0,00	-0,04	0,00
6	230	0	0,00	0,00	0,00
7	231	0	0,00	0,00	0,00
8	232	0	0,00	0,00	0,00
9	233	0	0,00	0,00	0,00
10	234	0	0,00	0,00	0,00
11	235	0	0,00	0,00	0,00
Total		2528		2211,72	100,00

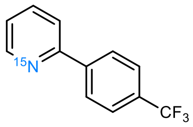

% Isotopic enrichment : 68,8

2-(2,4-Difluorophenyl)pyridine-1-^{15}N ([^{15}N]4)

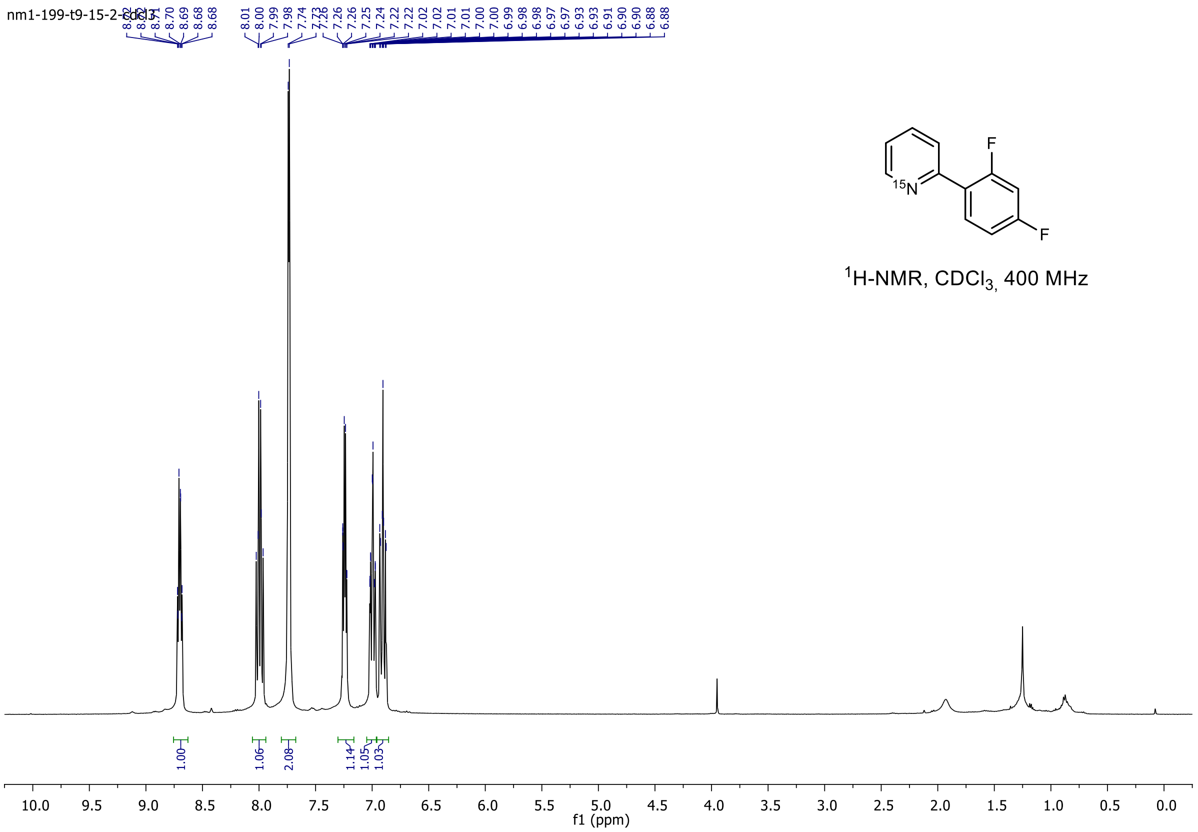

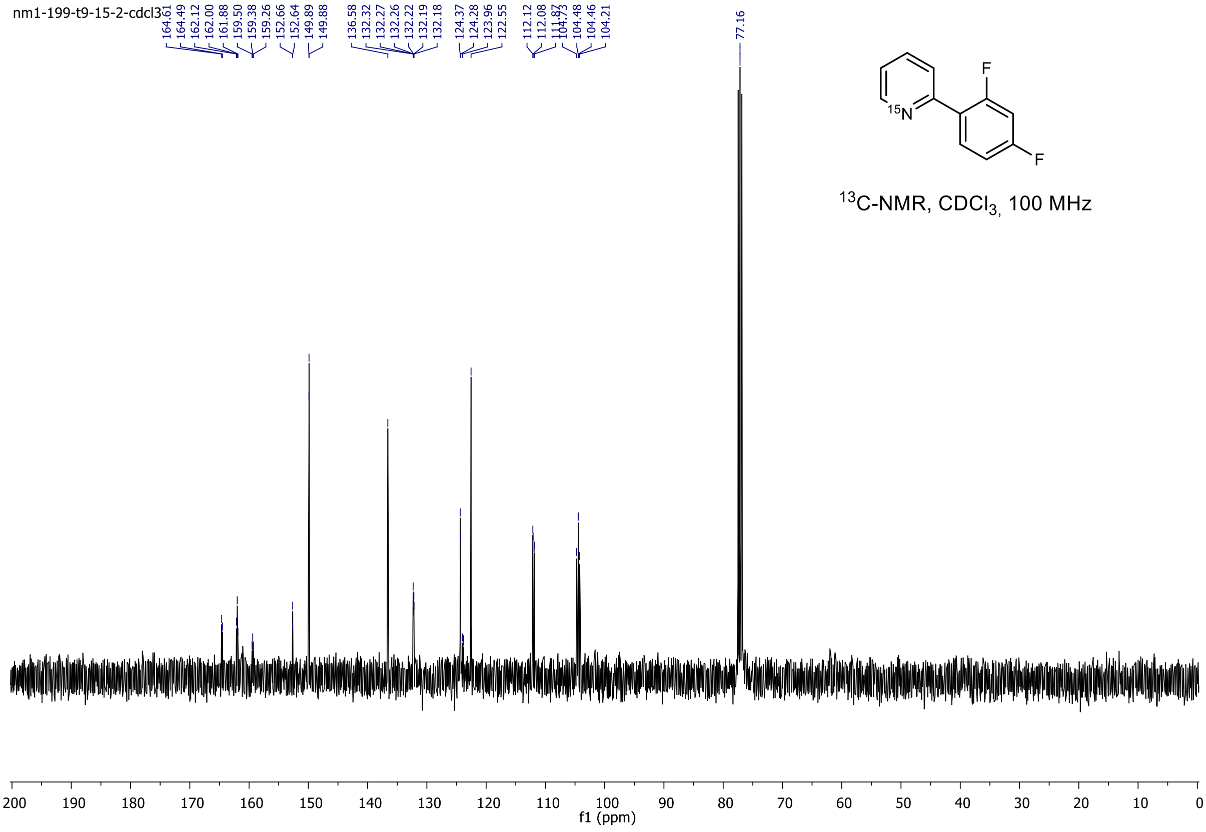

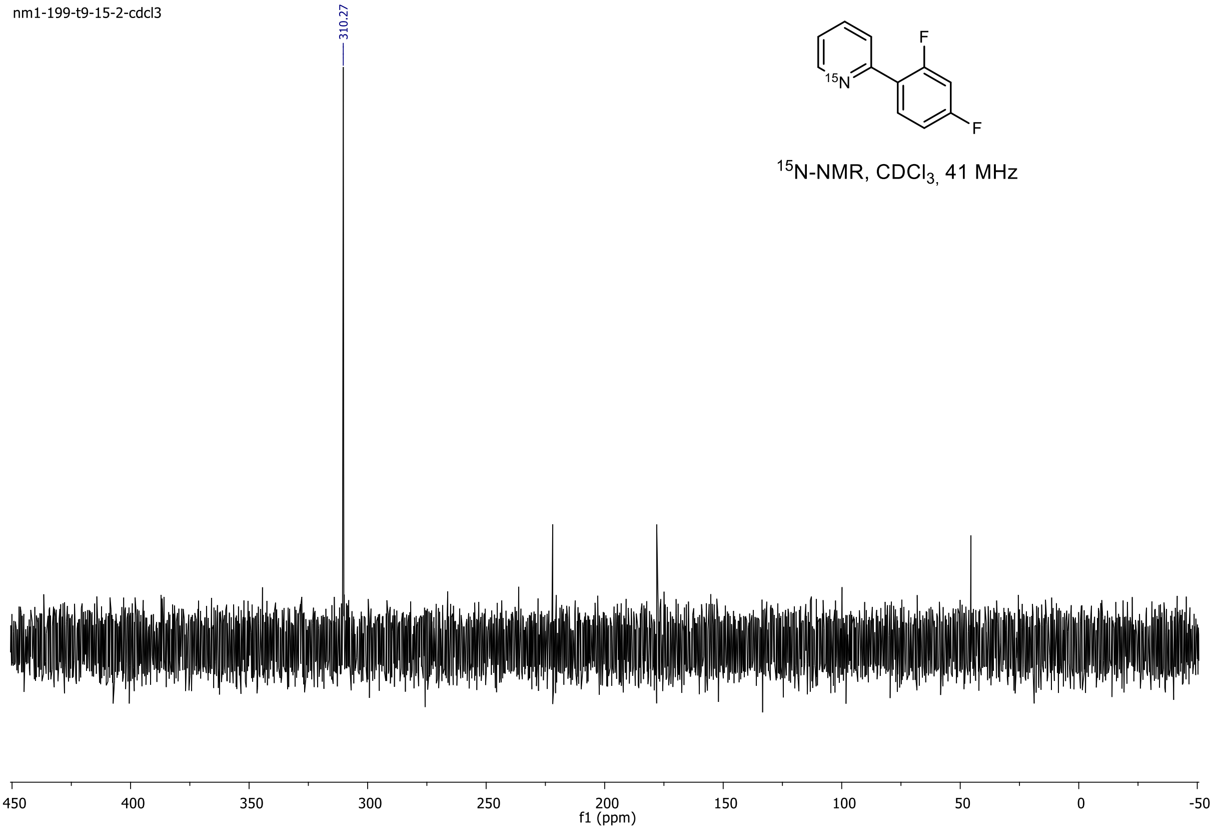

^{15}N-NMR, $CDCl_3$, 41 MHz

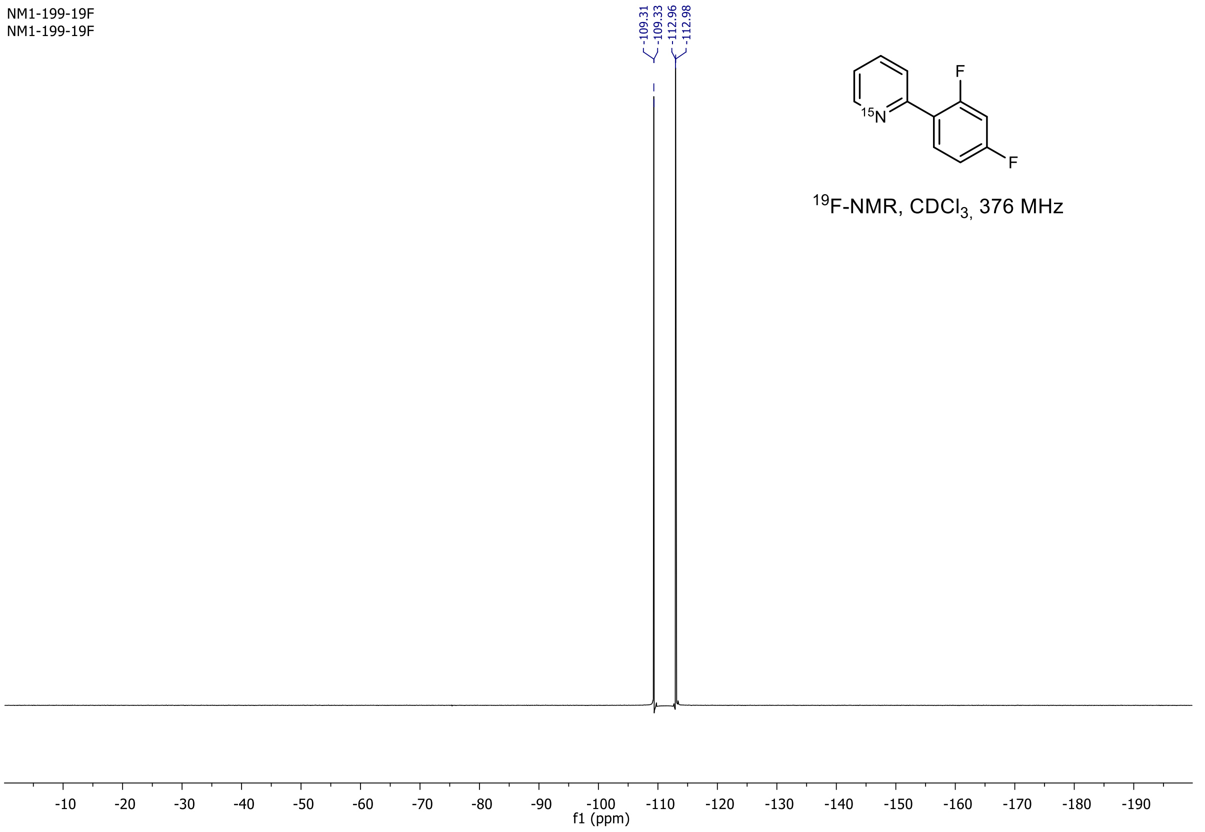

^{19}F-NMR, $CDCl_3$, 376 MHz

Isotopic Enrichment

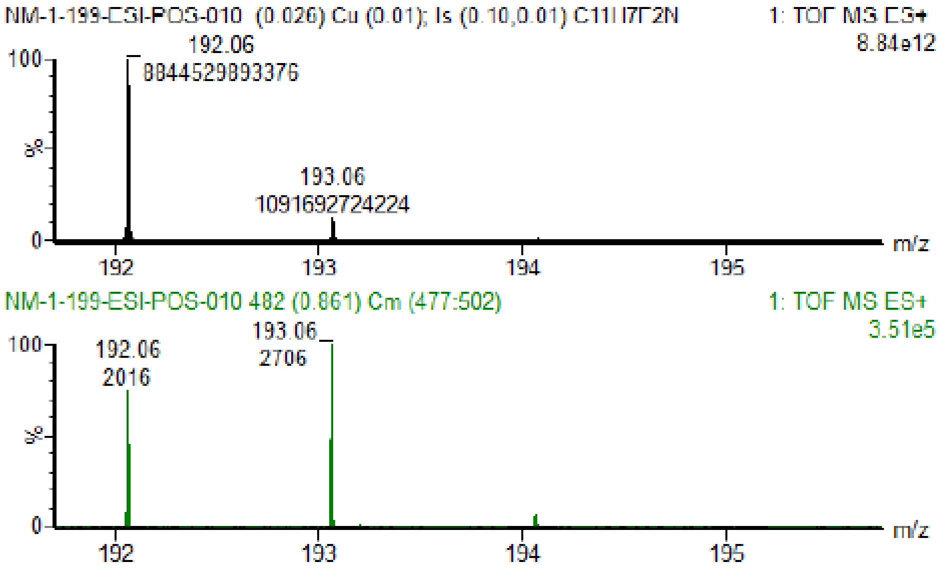

theorical isotopic distribution

	M	M+1	M+2	M+3	M+4	M+5	M+6	M+7
**m/z**	192	193	194	195	196	197	198	199
**%**	100,0	12,4	0,6	0,0	0,0	0,0	0,0	0,0

Enrichment calculation

Isotopomer	m/z	Area	natural isotope correction	Corrected area	Isotopic purity (%)
0	192	2016	0,00	2016,00	45,74
1	193	2706	249,98	2456,02	55,73
2	194	254	12,10	-62,64	-1,42
3	195	4	0,00	-2,97	-0,07
4	196	0	0,00	0,74	0,02
5	197	0	0,00	-0,07	0,00
6	198	0	0,00	0,00	0,00
7	199	0	0,00	0,00	0,00
8	200	0	0,00	0,00	0,00
9	201	0	0,00	0,00	0,00
10	202	0	0,00	0,00	0,00
11	203	0	0,00	0,00	0,00
Total		4980		4407,08	100,00

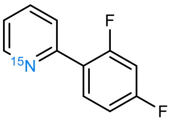

% Isotopic enrichment : 55,7

2-(Thiophen-2-yl)pyridine-1-^{15}N ([^{15}N]5)

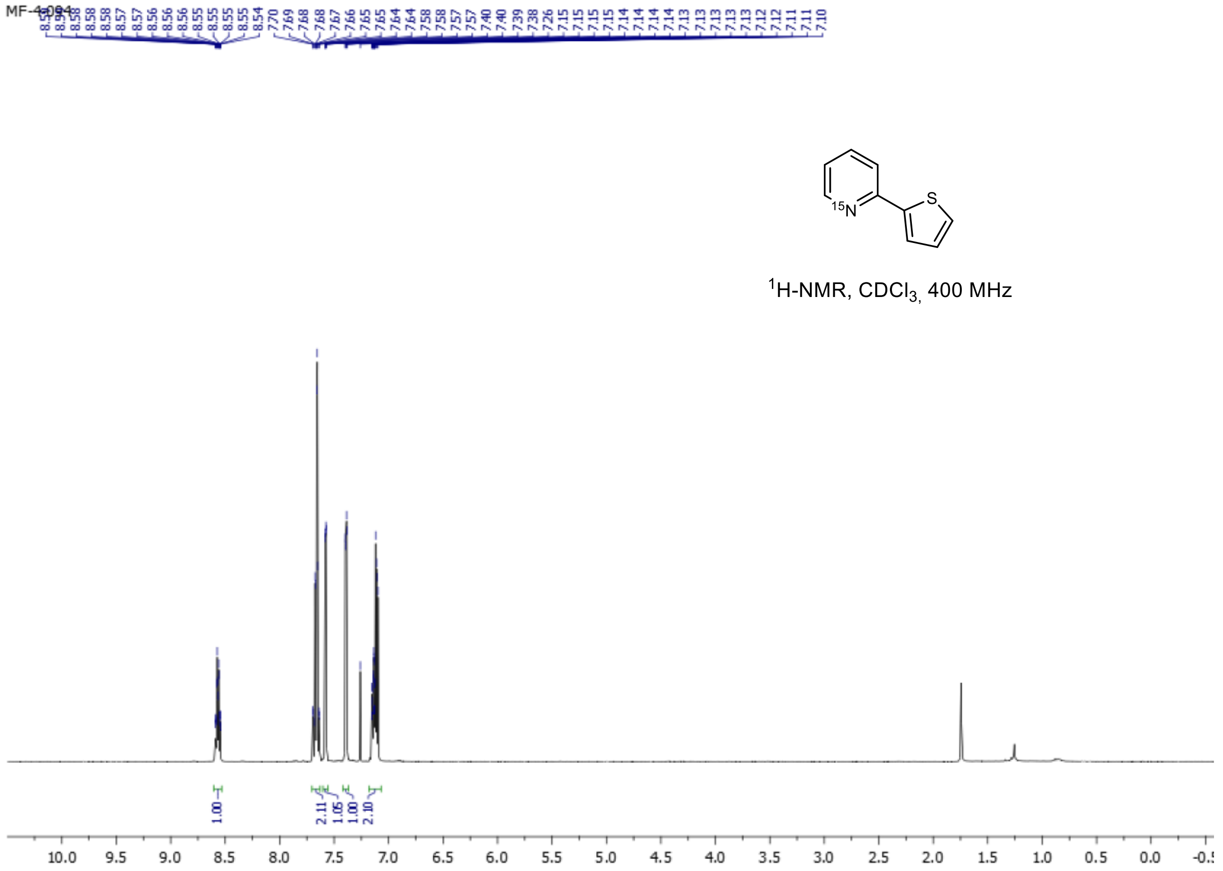

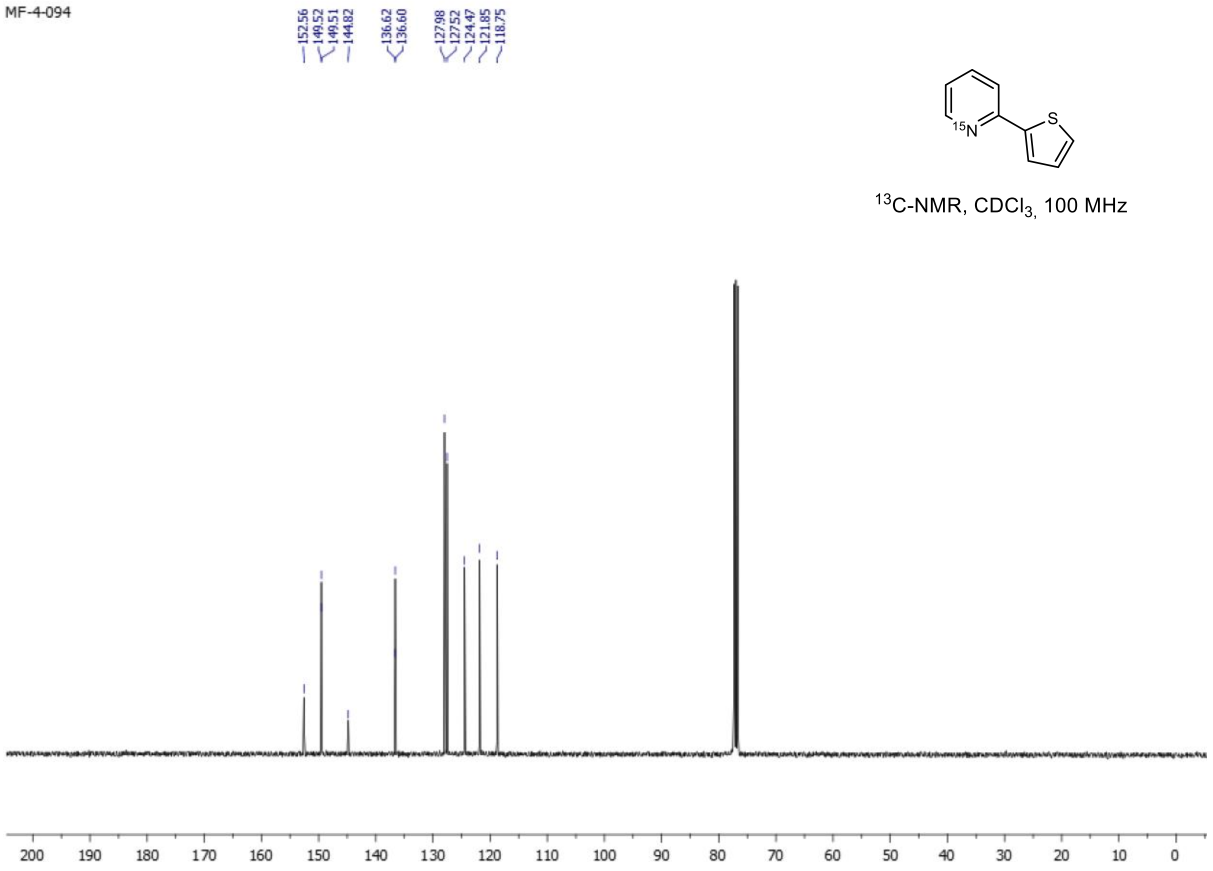

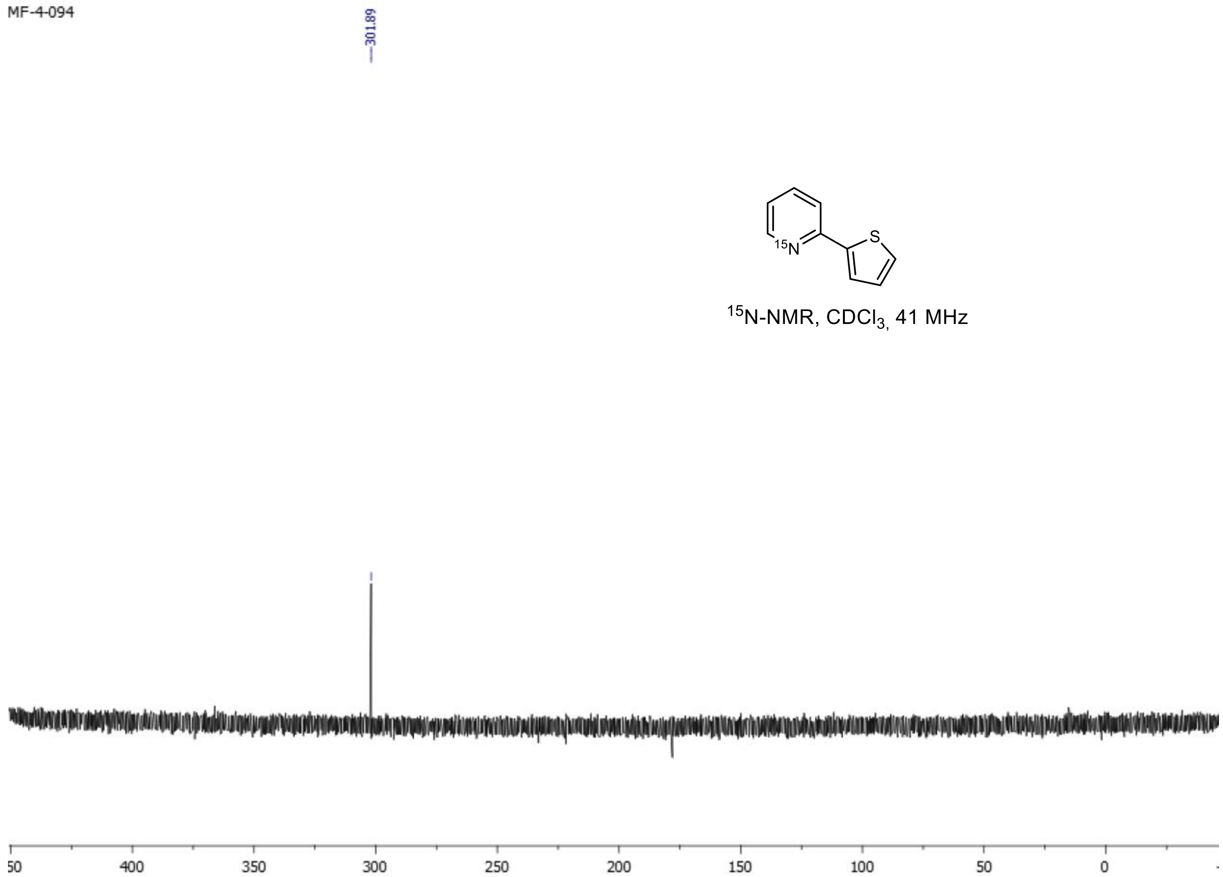
MF-4-094
301.89
15N
S
15N-NMR, CDCl3, 41 MHz
50
400
350
300
250
200
150
100
50
0

Isotopic Enrichment

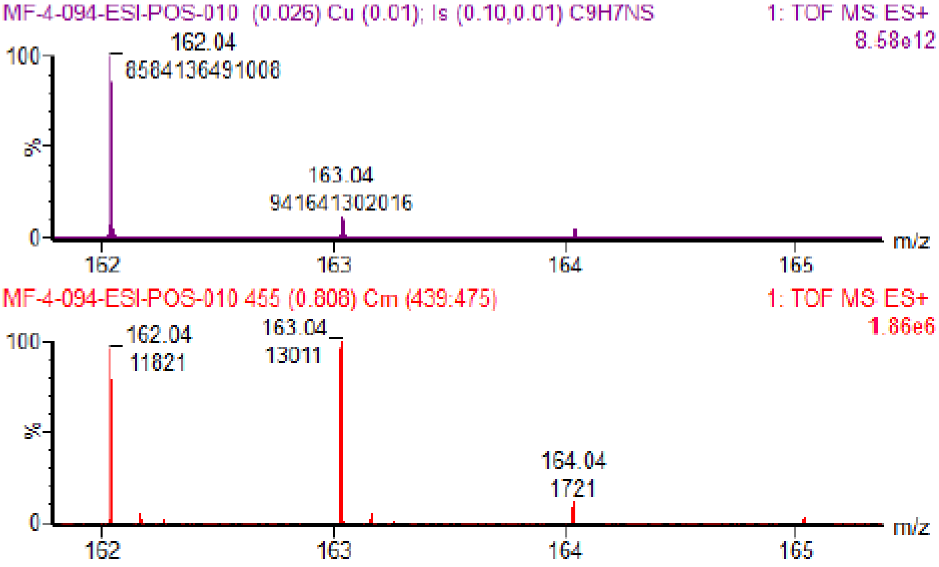

theorical isotopic distribution

	M	M+1	M+2	M+3	M+4	M+5	M+6	M+7
m/z	162	163	164	165	166	167	168	169
%	100,0	11,0	4,9	0,4	0,0	0,0	0,0	0,0

Enrichment calculation

Isotopomer	m/z	Area	natural isotope correction	Corrected area	Isotopic purity (%)
0	162	11821	0,00	11821,00	50,84
1	163	13011	1300,31	11710,69	50,36
2	164	1721	579,23	-146,40	-0,63
3	165	475	47,28	-130,00	-0,56
4	166	15	0,00	-10,37	-0,04
5	167	0	0,00	8,10	0,03
6	168	0	0,00	0,14	0,00
7	169	0	0,00	-0,37	0,00
8	170	0	0,00	0,00	0,00
9	171	0	0,00	0,02	0,00
10	172	0	0,00	0,00	0,00
11	173	0	0,00	0,00	0,00
Total		27043		23252,79	100,00

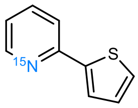

% Isotopic enrichment : 50,4

2-(Pyridin-2-yl-^{15}N)benzo[d]oxazole ([^{15}N]6)

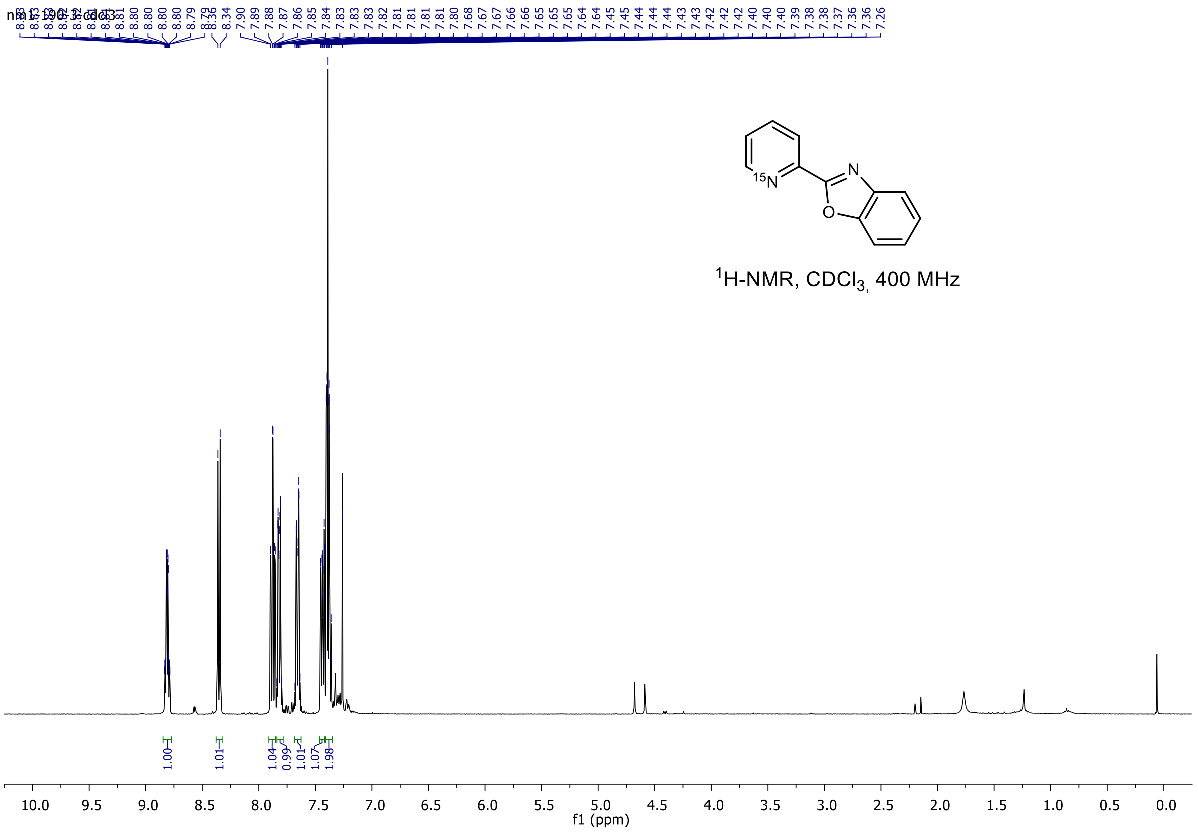

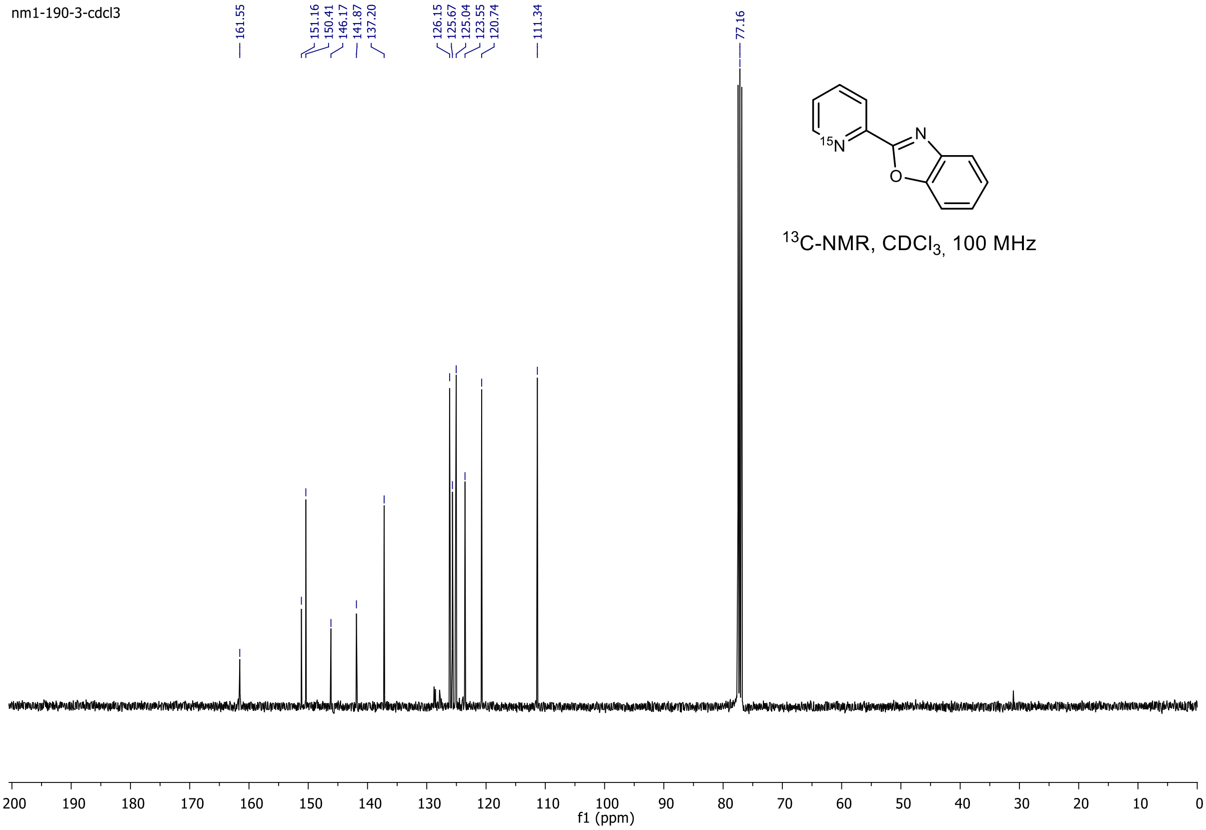

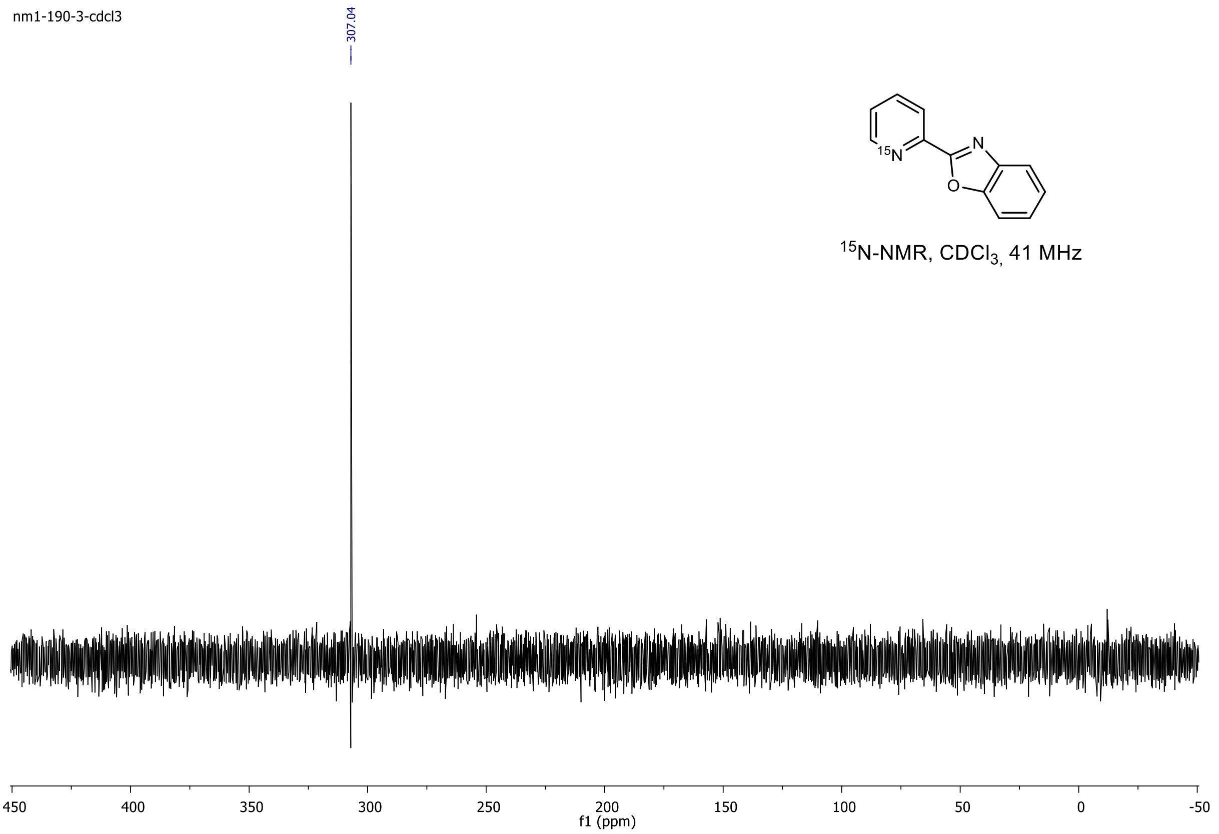
nm1-190-3-cdcl3
307.04
15N
N
O
15N-NMR, CDCl3, 41 MHz
450
400
350
300
250
200
150
100
50
0
-50
f1 (ppm)

Isotopic Enrichment

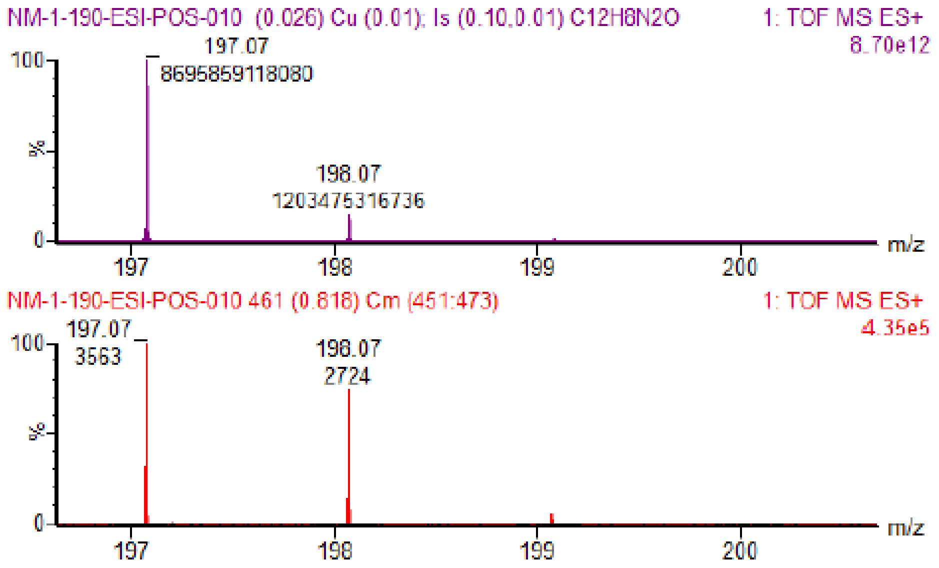

theorical isotopic distribution

	M	M+1	M+2	M+3	M+4	M+5	M+6	M+7
m/z	197	198	199	200	201	202	203	204
%	100,0	13,8	1,1	0,0	0,0	0,0	0,0	0,0

Enrichment calculation

Isotopomer	m/z	Area	natural isotope correction	Corrected area	Isotopic purity (%)
0	197	3563	0,00	3563,00	62,28
1	198	2724	491,69	2232,31	39,02
2	199	278	39,19	-69,25	-1,21
3	200	8	0,00	-7,00	-0,12
4	201	0	0,00	1,73	0,03
5	202	0	0,00	-0,16	0,00
6	203	0	0,00	0,00	0,00
7	204	0	0,00	0,00	0,00
8	205	0	0,00	0,00	0,00
9	206	0	0,00	0,00	0,00
10	207	0	0,00	0,00	0,00
11	208	0	0,00	0,00	0,00
Total		6573		5720,63	100,00

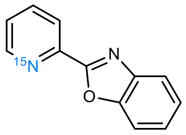

% Isotopic enrichment : 39,0

2-(3-Phenylpropyl)pyridine-1-^{15}N ([^{15}N]7)

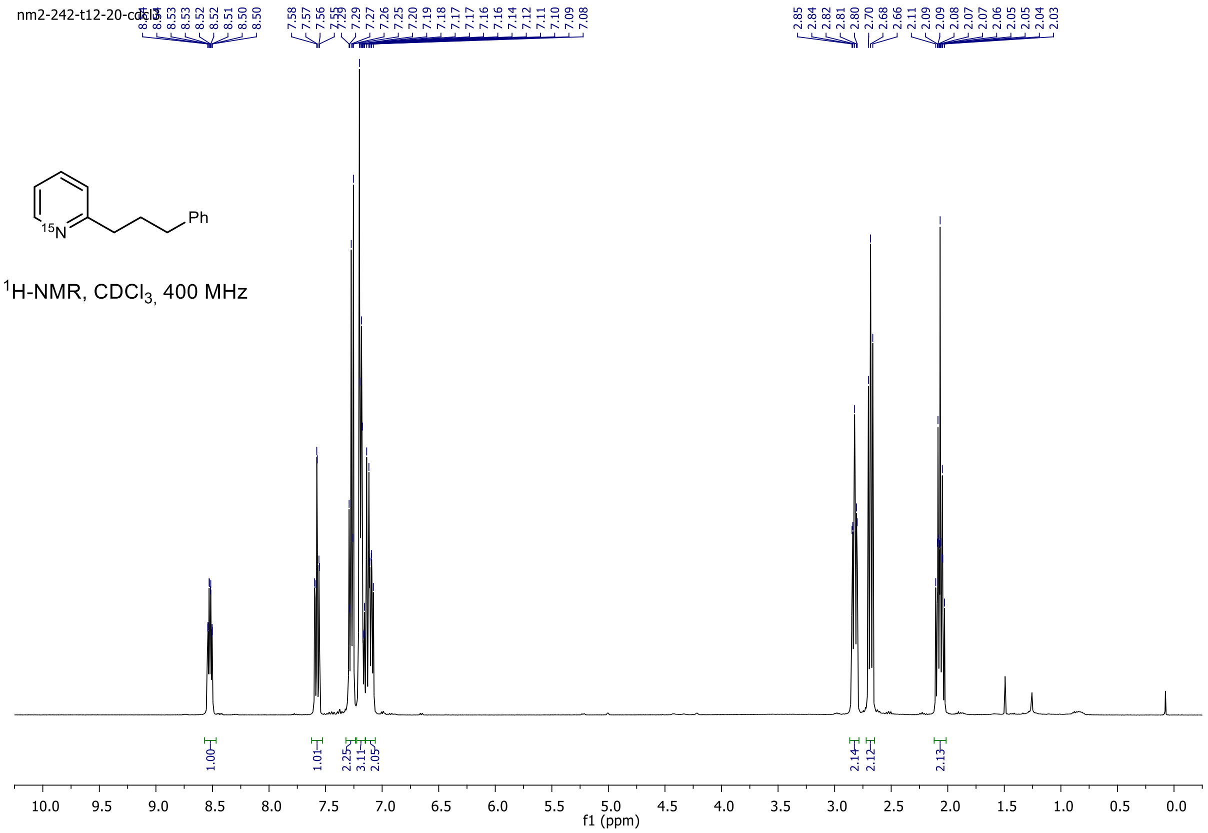

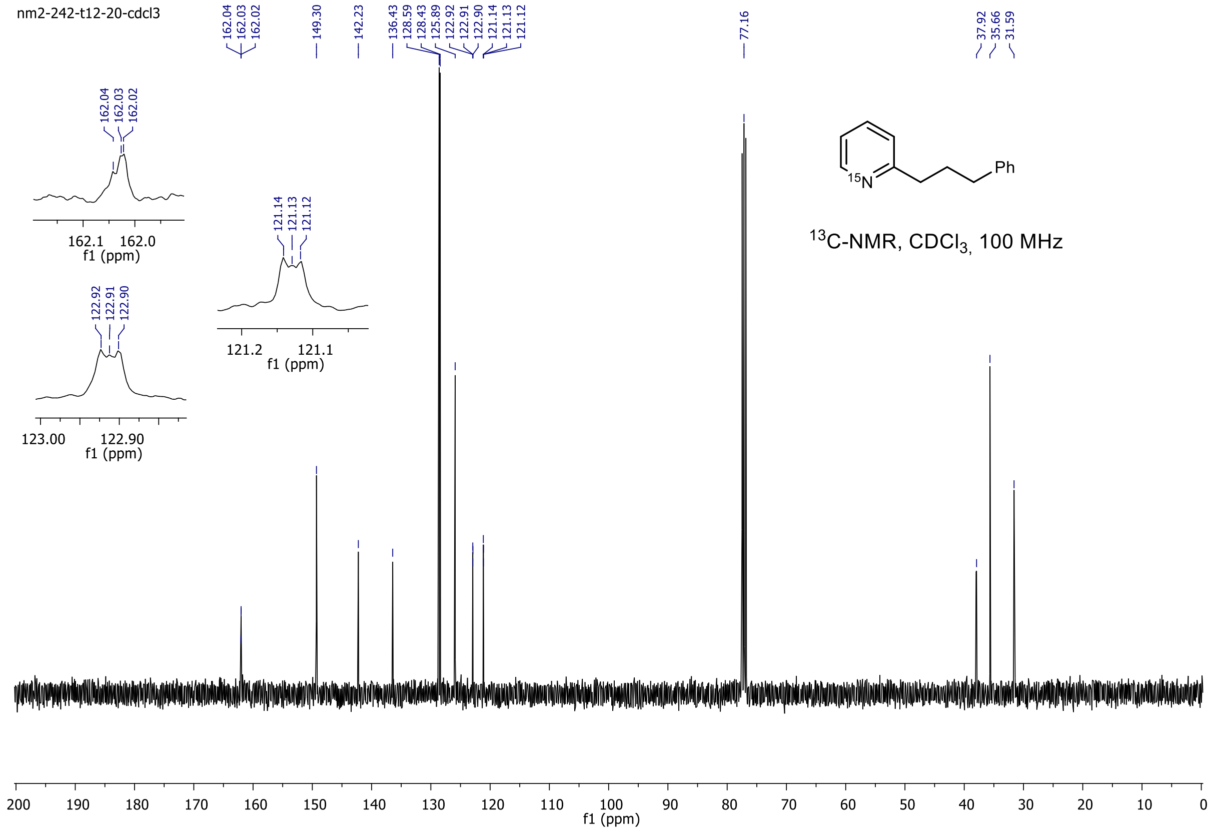

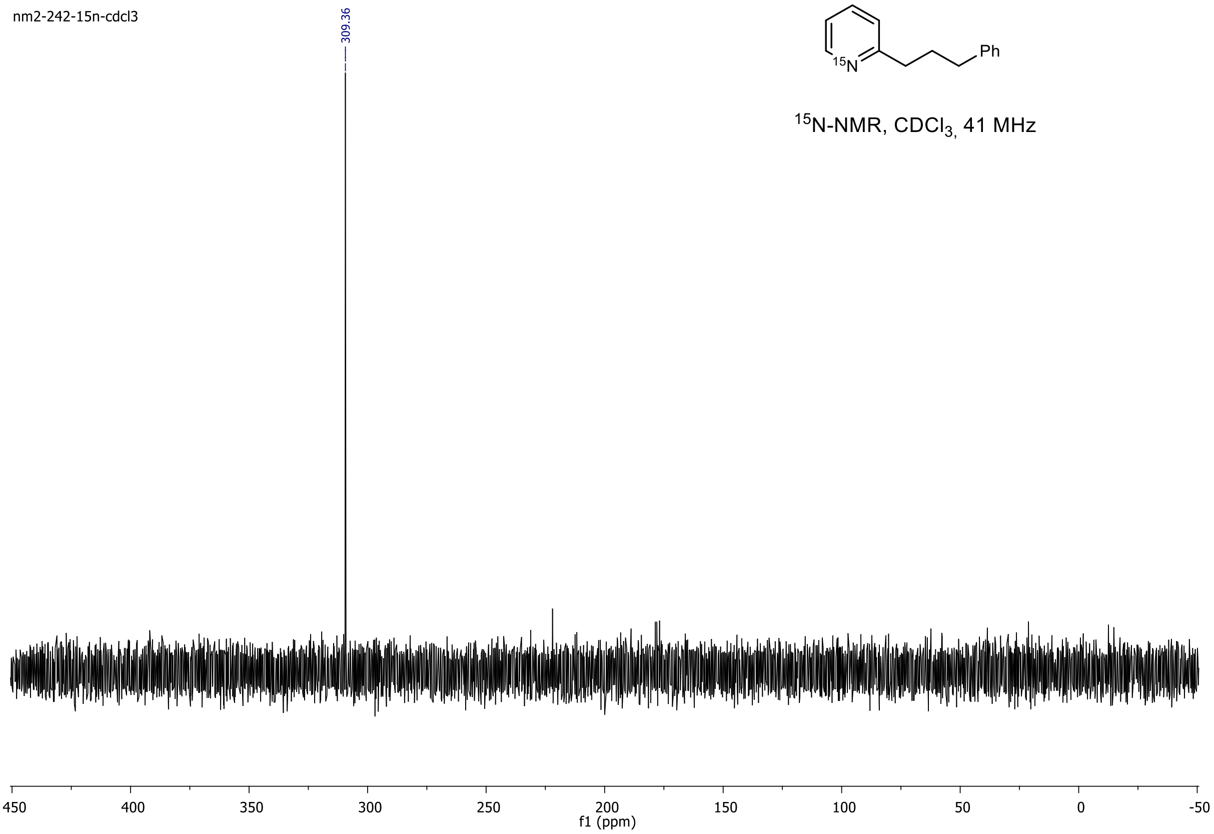

^{15}N-NMR, $CDCl_3$, 41 MHz

Isotopic Enrichment

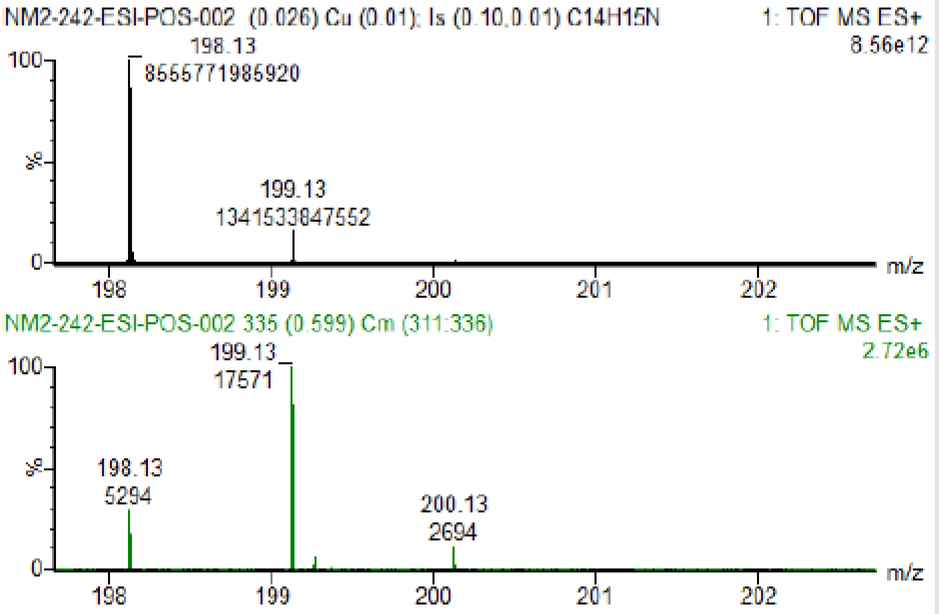

theorical isotopic distribution

	M	M+1	M+2	M+3	M+4	M+5	M+6	M+7
m/z	198	199	200	201	202	203	204	205
%	100,0	15,8	1,1	0,0	0,0	0,0	0,0	0,0

Enrichment calculation

Isotopomer	m/z	Area	natural isotope correction	Corrected area	Isotopic purity (%)
0	198	5294	0,00	5294,00	24,12
1	199	17571	836,45	16734,55	76,23
2	200	2694	58,23	-8,29	-0,04
3	201	101	0,00	-81,77	-0,37
4	202	3	0,00	16,01	0,07
5	203	0	0,00	-1,63	-0,01
6	204	0	0,00	0,08	0,00
7	205	0	0,00	0,01	0,00
8	206	0	0,00	0,00	0,00
9	207	0	0,00	0,00	0,00
10	208	0	0,00	0,00	0,00
11	209	0	0,00	0,00	0,00
Total		25663		21952,95	100,00

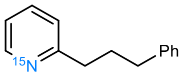

% Isotopic enrichment : 76,2

Ethyl picolinate-^{15}N ([^{15}N]8)

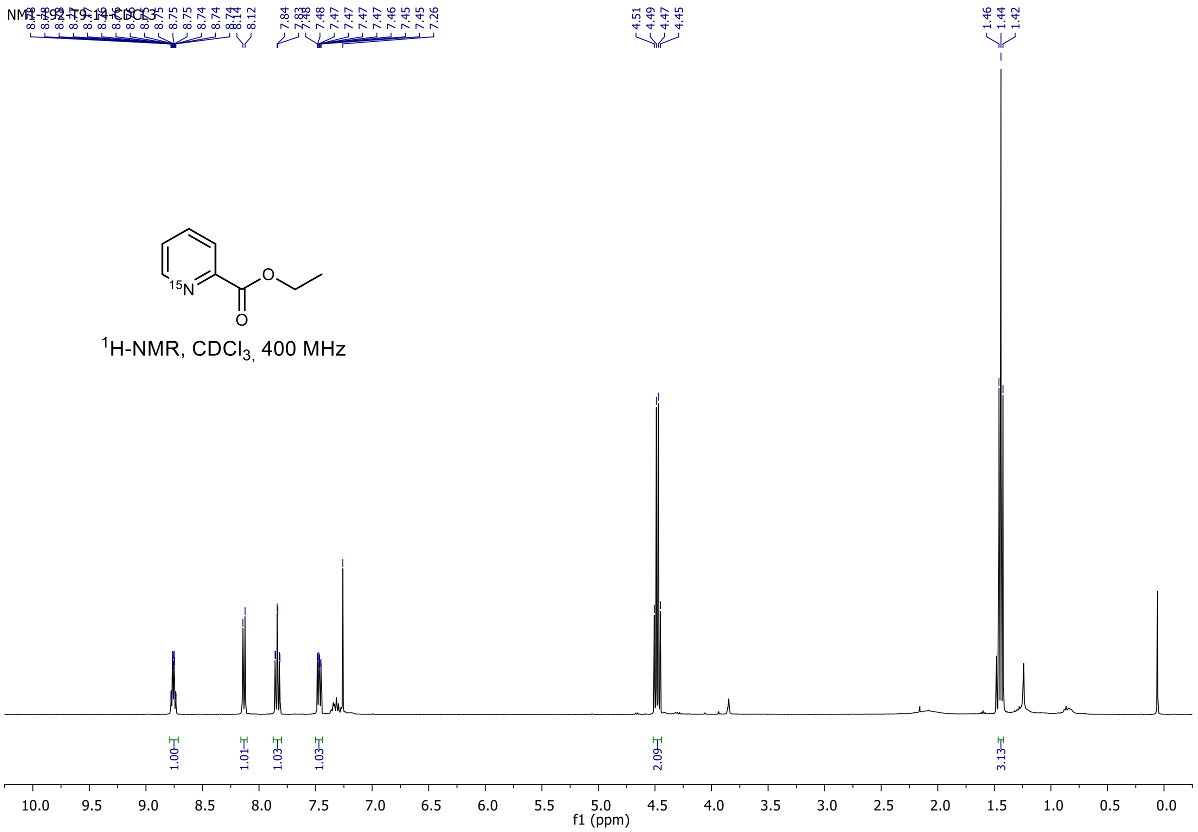

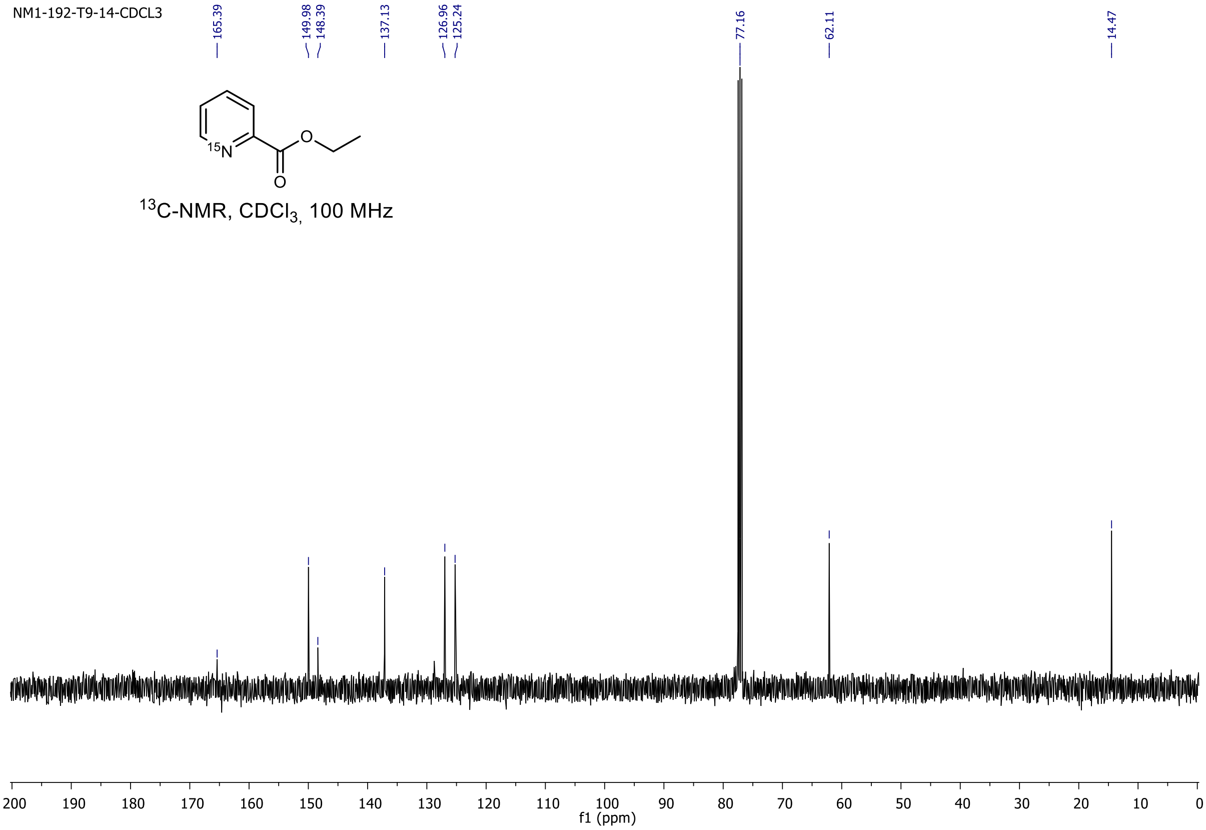

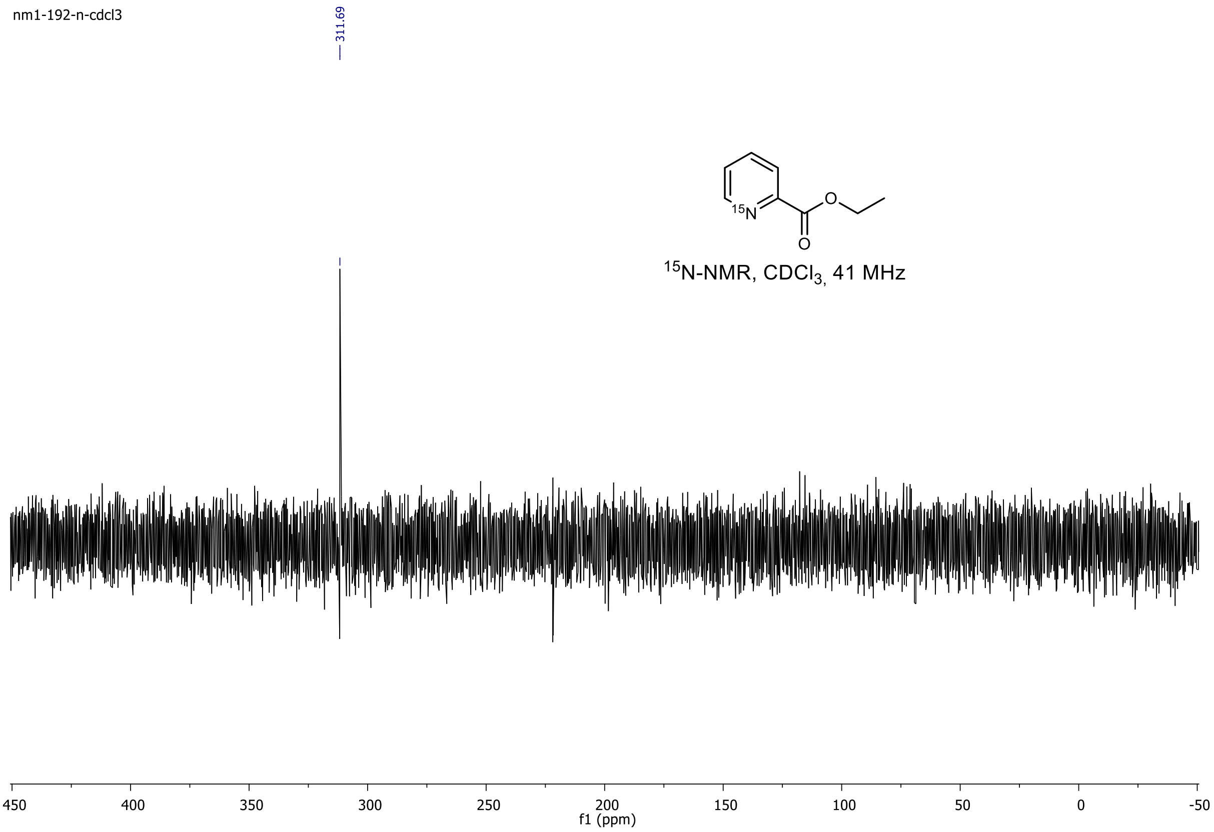
nm1-192-n-cdcl3
311.69
15N
O
O
15N-NMR, CDCl3, 41 MHz
450
400
350
300
250
200
150
100
50
0
-50
f1 (ppm)

Isotopic Enrichment

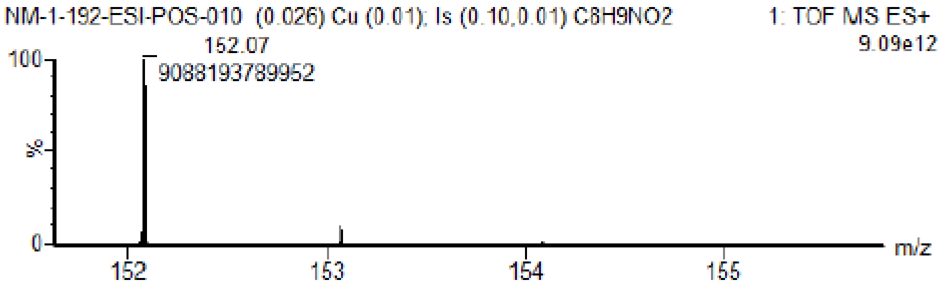

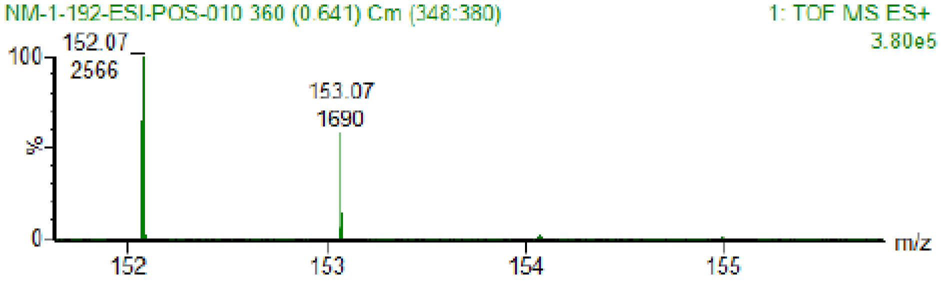

theorical isotopic distribution

	M	M+1	M+2	M+3	M+4	M+5	M+6	M+7
**m/z**	152	153	154	155	156	157	158	159
**%**	100,0	9,3	0,7	0,0	0,0	0,0	0,0	0,0

Enrichment calculation

Isotopomer	m/z	Area	natural isotope correction	Corrected area	Isotopic purity (%)
0	152	2566	0,00	2566,00	64,84
1	153	1690	238,64	1451,36	36,68
2	154	92	17,96	-60,94	-1,54
3	155	5	0,00	0,51	0,01
4	156	0	0,00	0,38	0,01
5	157	0	0,00	-0,04	0,00
6	158	0	0,00	0,00	0,00
7	159	0	0,00	0,00	0,00
8	160	0	0,00	0,00	0,00
9	161	0	0,00	0,00	0,00
10	162	0	0,00	0,00	0,00
11	163	0	0,00	0,00	0,00
Total		4353		3957,27	100,00

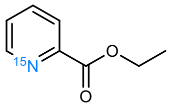

% Isotopic enrichment : 36,7

4-Phenylpyridine-1-^{15}N ([^{15}N]9)

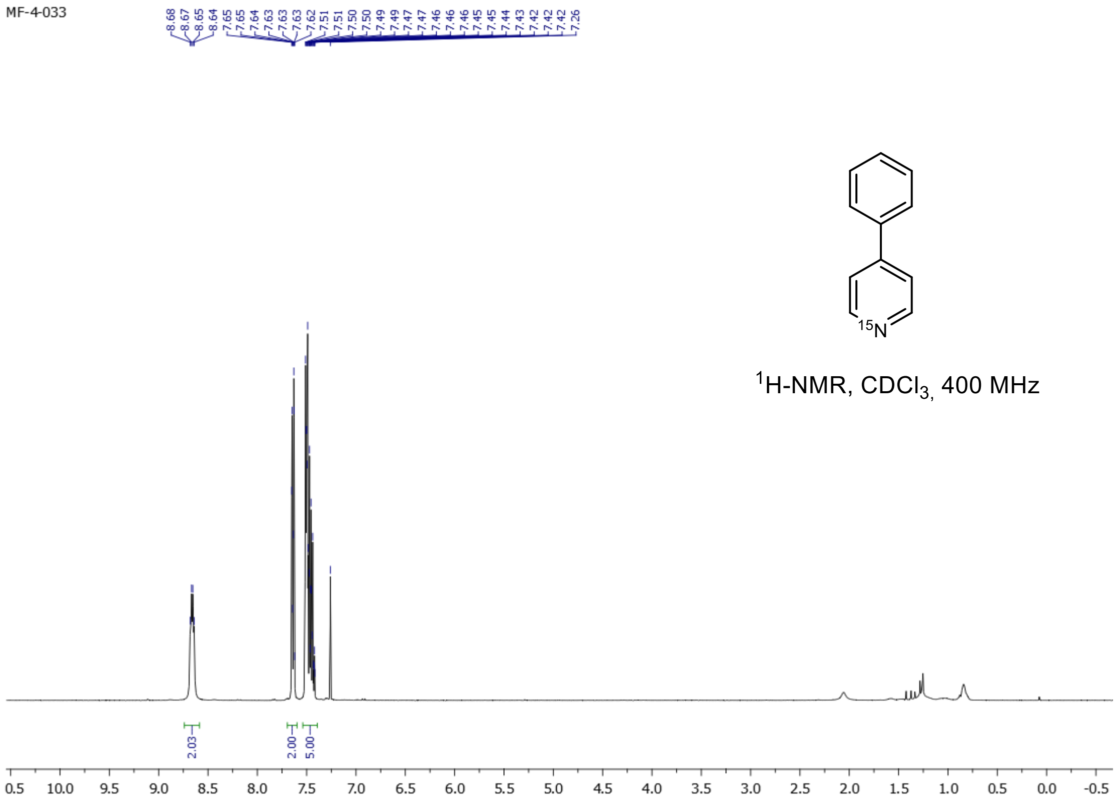

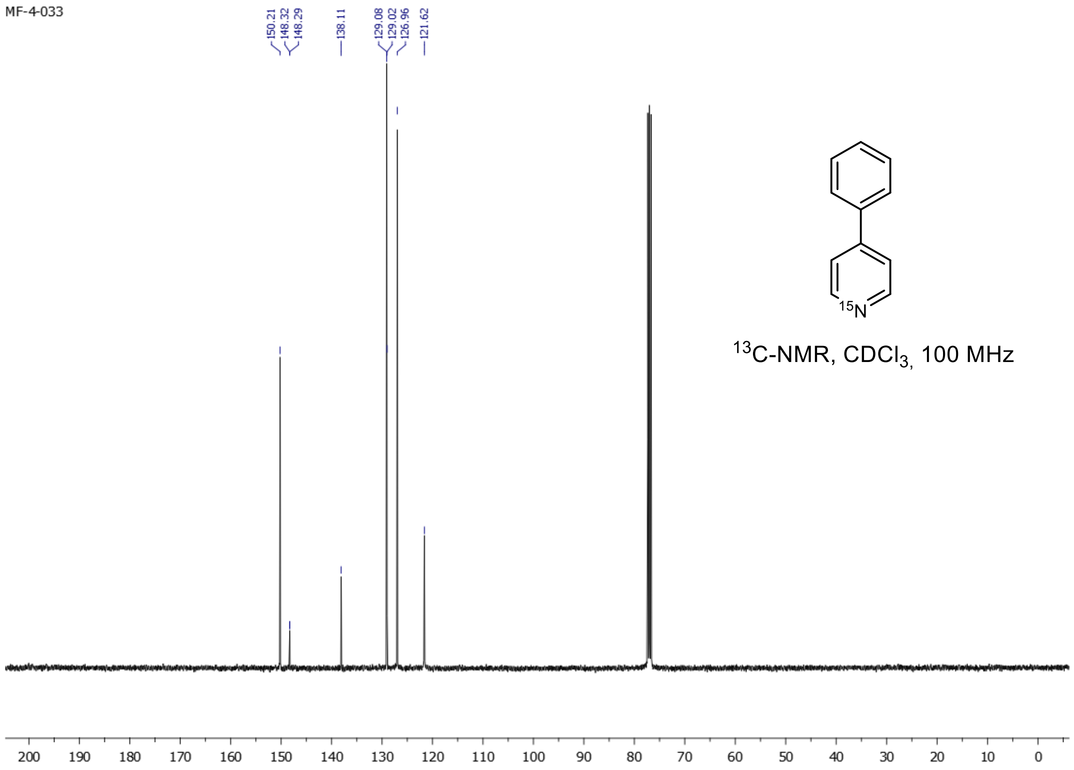

MF-4-080-15N
MF-4-080-15N

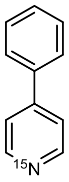

^{15}N-NMR, $CDCl_3$, 41 MHz

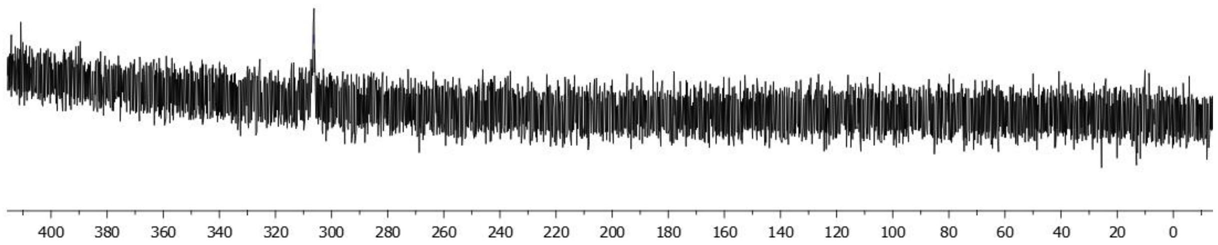

Isotopic Enrichment

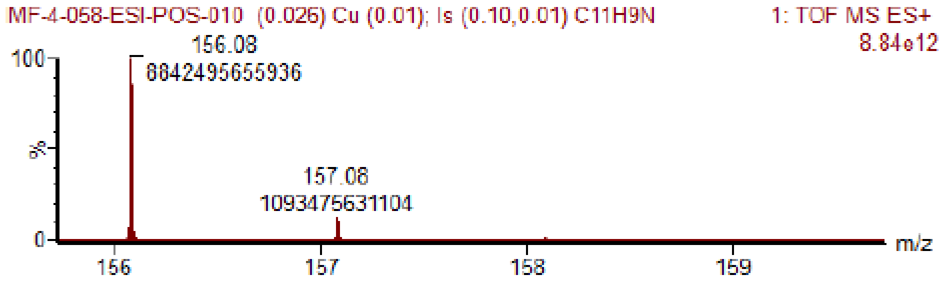

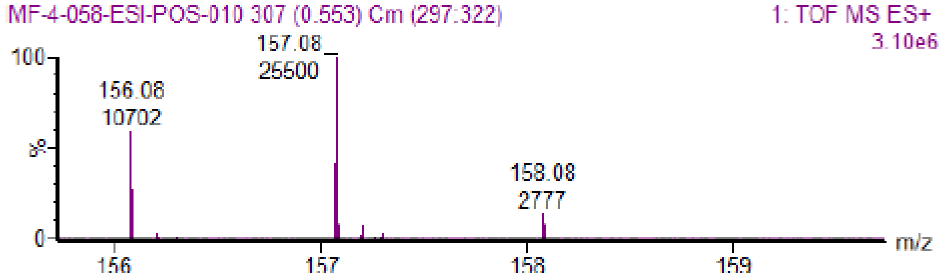

theorical isotopic distribution

	M	M+1	M+2	M+3	M+4	M+5	M+6	M+7
m/z	156	157	158	159	160	161	162	163
%	100,0	12,4	0,6	0,0	0,0	0,0	0,0	0,0

Enrichment calculation

Isotopomer	m/z	Area	natural isotope correction	Corrected area	Isotopic purity (%)
0	156	10702	0,00	10702,00	30,99
1	157	25500	1327,05	24172,95	69,99
2	158	2777	64,21	-284,66	-0,82
3	159	50	0,00	-59,74	-0,17
4	160	0	0,00	9,12	0,03
5	161	0	0,00	-0,77	0,00
6	162	0	0,00	0,04	0,00
7	163	0	0,00	0,00	0,00
8	164	0	0,00	0,00	0,00
9	165	0	0,00	0,00	0,00
10	166	0	0,00	0,00	0,00
11	167	0	0,00	0,00	0,00
Total		39029		34538,94	100,00

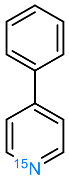

IE obtained following procedure A

% Isotopic enrichment : 70,0

Isotopic Enrichment

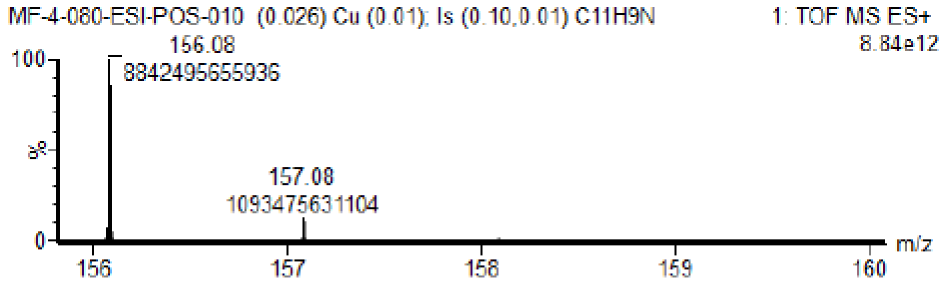

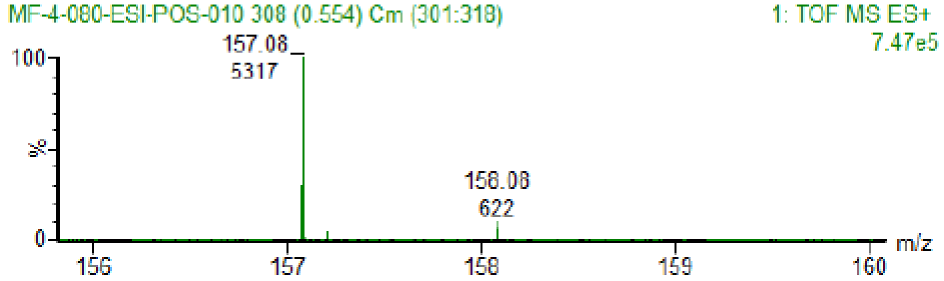

theorical isotopic distribution

	M	M+1	M+2	M+3	M+4	M+5	M+6	M+7
m/z	157	158	159	160	161	162	163	164
%	100,0	12,4	0,6	0,0	0,0	0,0	0,0	0,0

Enrichment calculation

Isotopomer	m/z	Area	natural isotope correction	Corrected area	Isotopic purity (%)
0	157	24	0,00	24,00	0,45
1	158	5317	3,00	5314,02	99,55
2	159	622	0,15	-37,08	-0,70
3	160	12	0,00	-15,29	-0,29
4	161	0	0,00	2,12	0,04
5	162	0	0,00	-0,17	0,00
6	163	0	0,00	0,01	0,00
7	164	0	0,00	0,00	0,00
8	165	0	0,00	0,00	0,00
9	166	0	0,00	0,00	0,00
10	167	0	0,00	0,00	0,00
11	168	0	0,00	0,00	0,00
Total		5975		5287,61	99,05

% Isotopic enrichment : 99,6

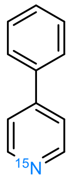

IE obtained following procedure D

4-(4-Phenoxyphenyl)-^{15}N-pyridine ([^{15}N]10)

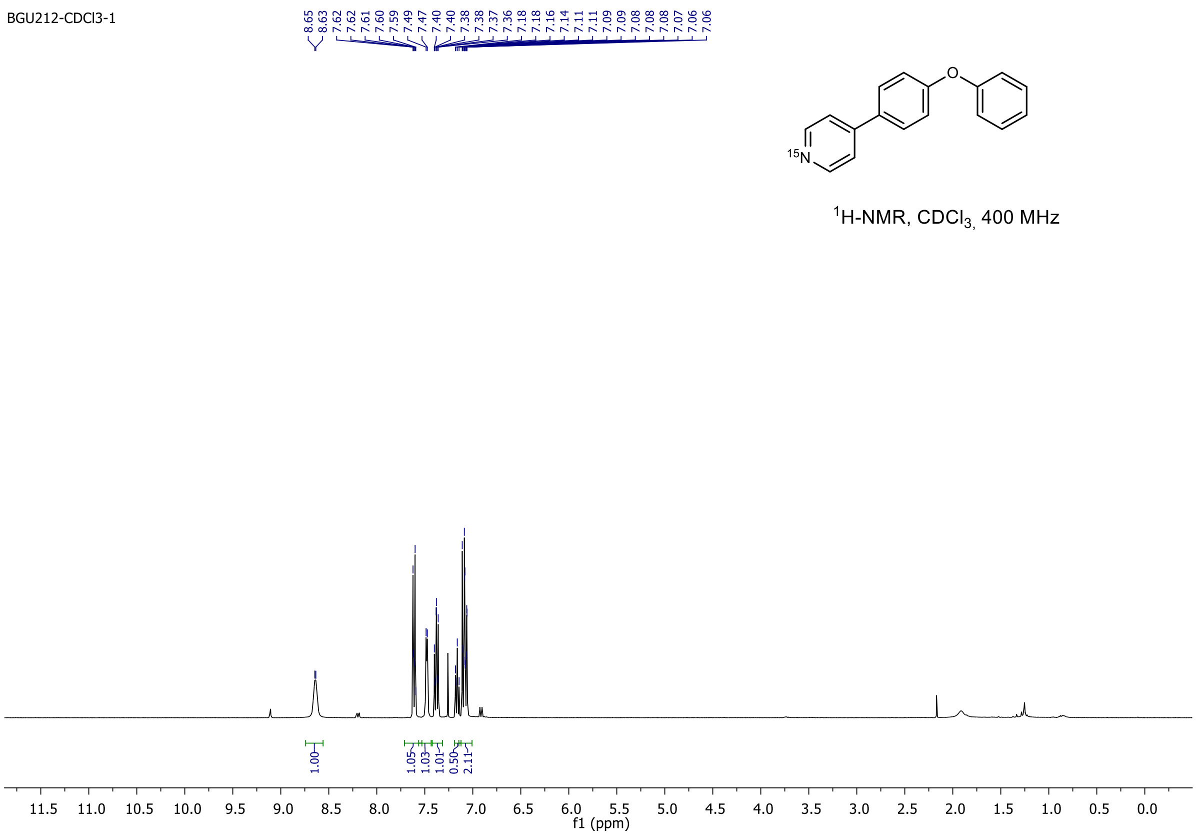

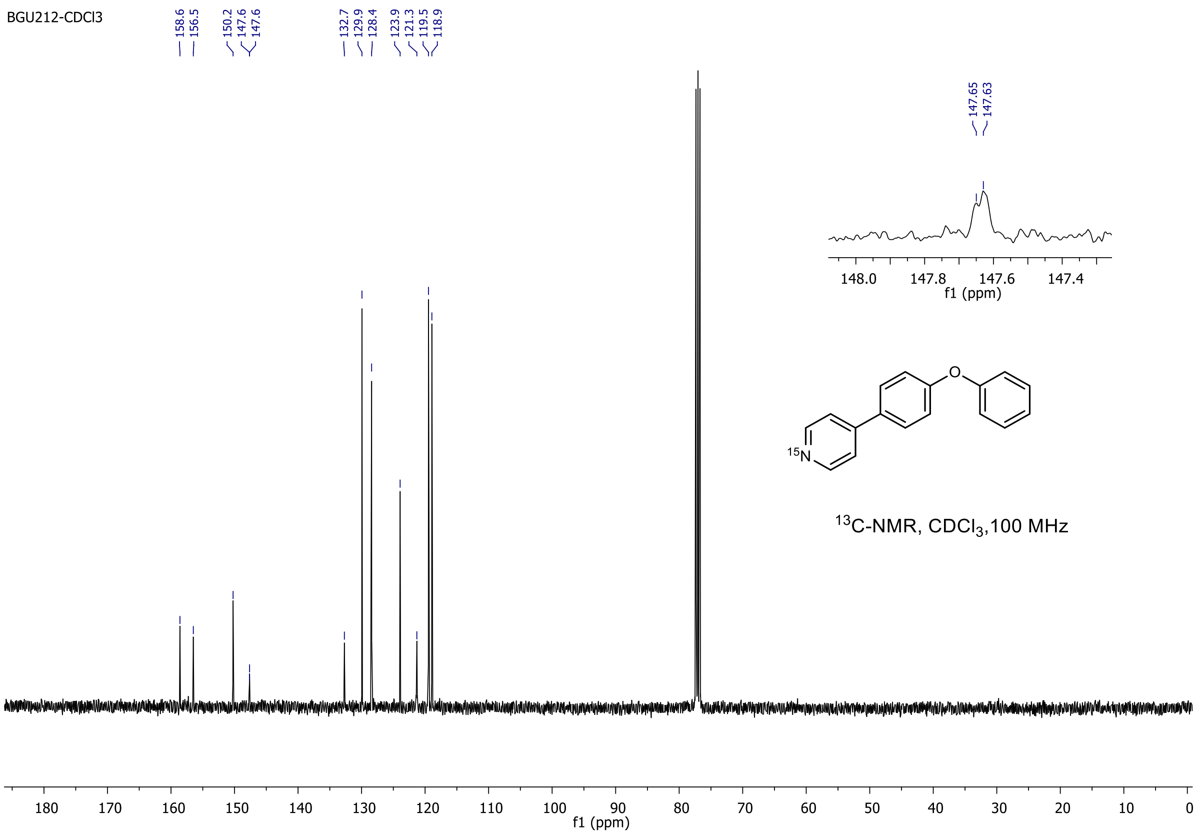

BGU212-15N

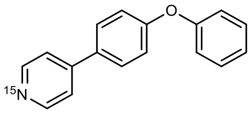

^{15}N-NMR, $CDCl_3$, 41 MHz

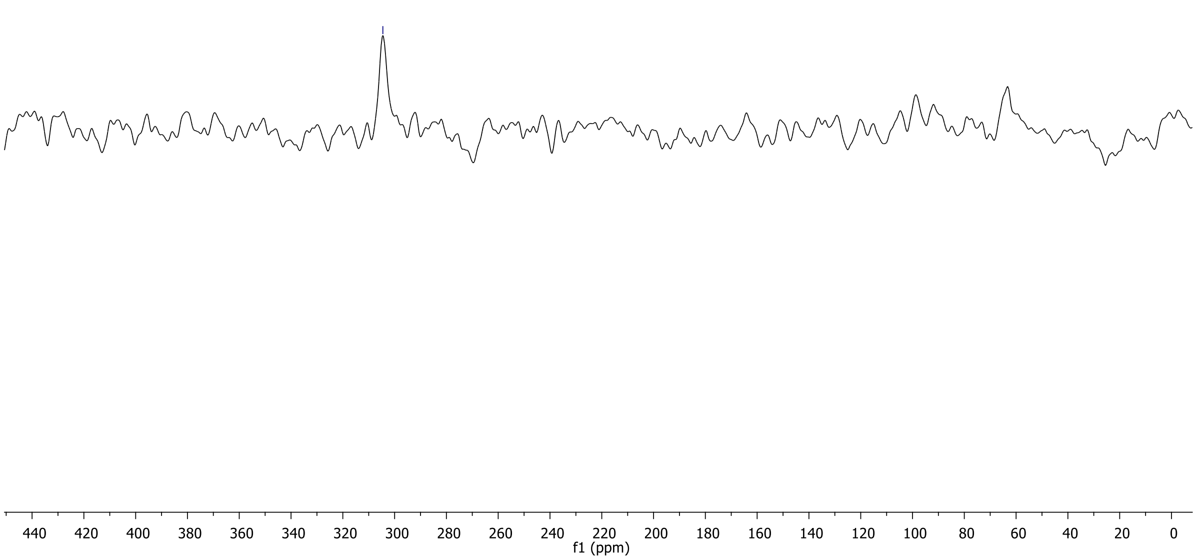

Isotopic Enrichment

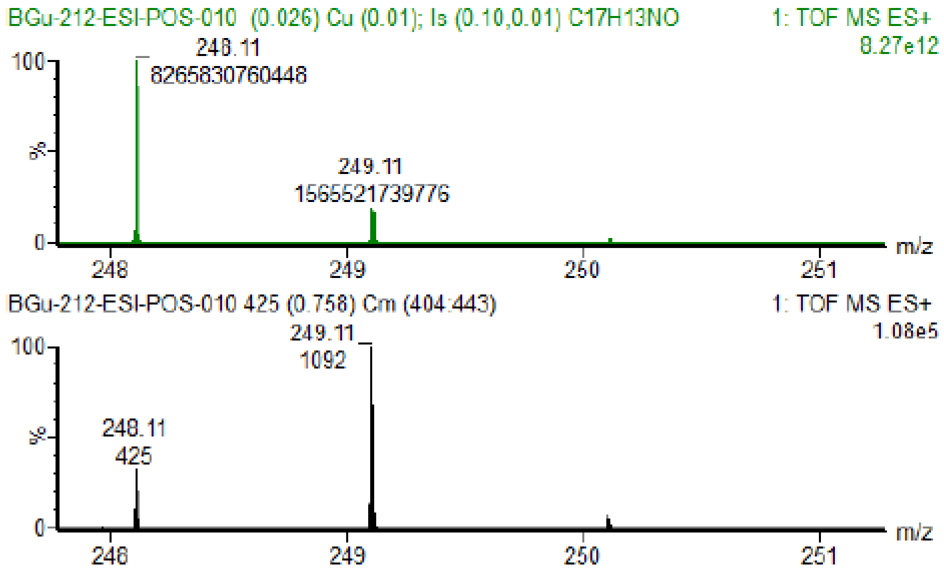

theorical isotopic distribution

	M	M+1	M+2	M+3	M+4	M+5	M+6	M+7
**m/z**	248	249	250	251	252	253	254	255
**%**	100,0	19,0	1,9	0,1	0,0	0,0	0,0	0,0

Enrichment calculation

Isotopomer	m/z	Area	natural isotope correction	Corrected area	Isotopic purity (%)
0	248	425	0,00	425,00	31,19
1	249	1092	80,75	1011,25	74,20
2	250	121	8,08	-79,21	-5,81
3	251	11	0,43	6,41	0,47
4	252	0	0,00	-0,72	-0,05
5	253	0	0,00	0,10	0,01
6	254	0	0,00	-0,01	0,00
7	255	0	0,00	0,00	0,00
8	256	0	0,00	0,00	0,00
9	257	0	0,00	0,00	0,00
10	258	0	0,00	0,00	0,00
11	259	0	0,00	0,00	0,00
Total		1649		1362,81	100,00

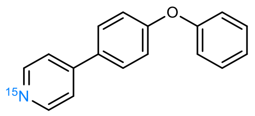

% Isotopic enrichment : 74,2

5-(Pyridin-4-yl-^{15}N)thiophene-2-carbaldehyde ([^{15}N]11)

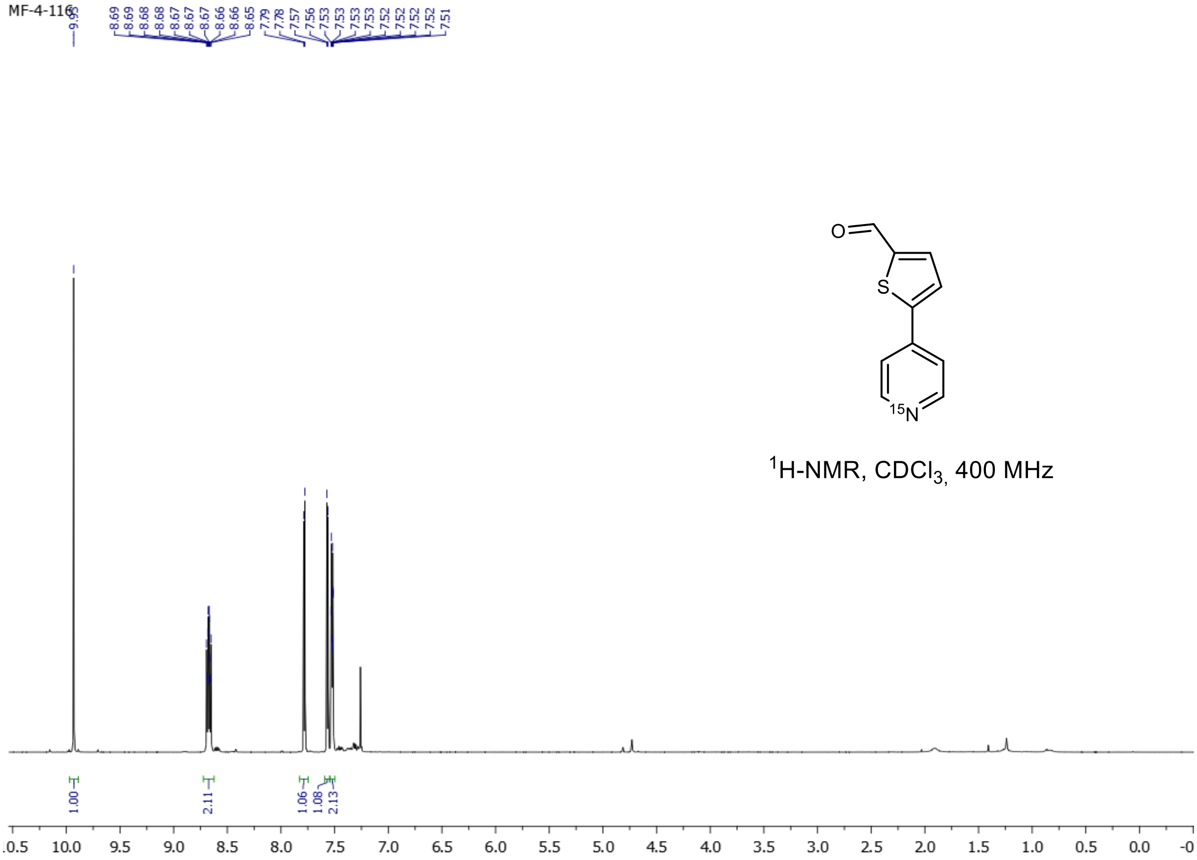

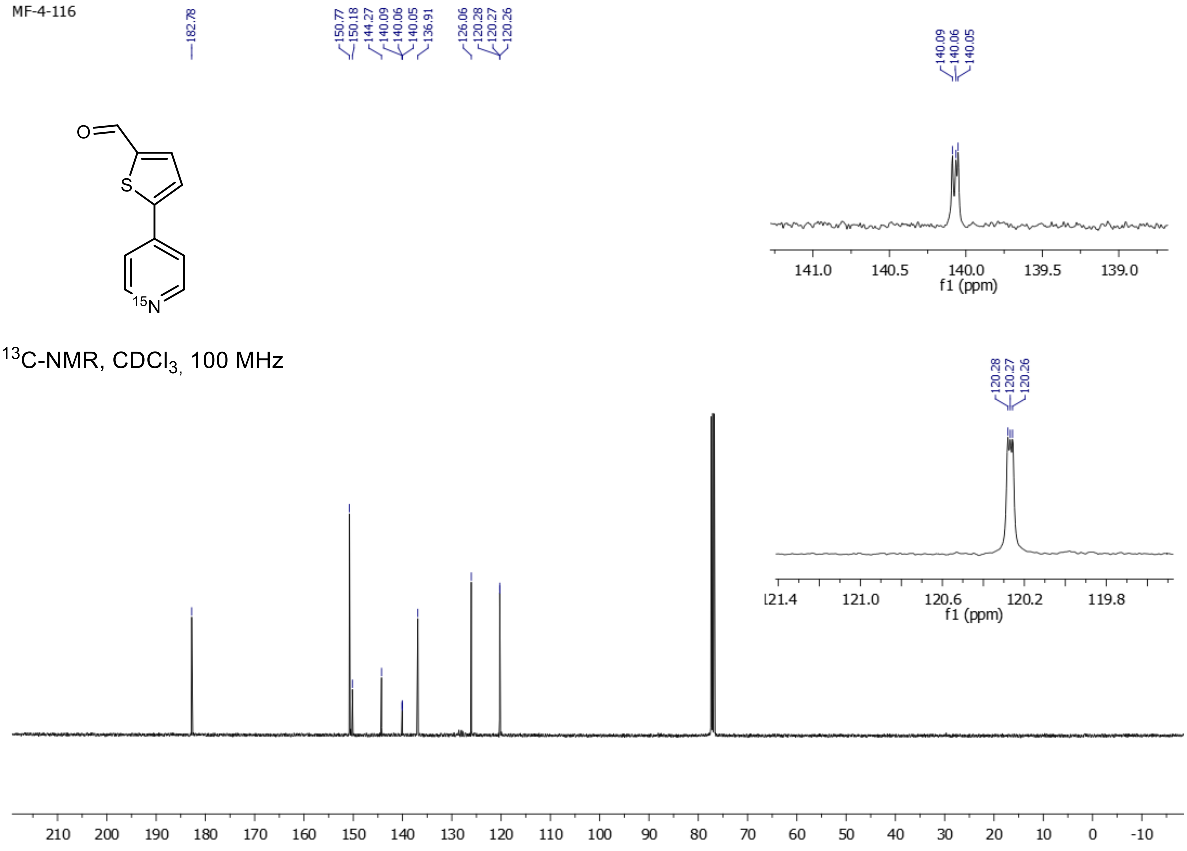

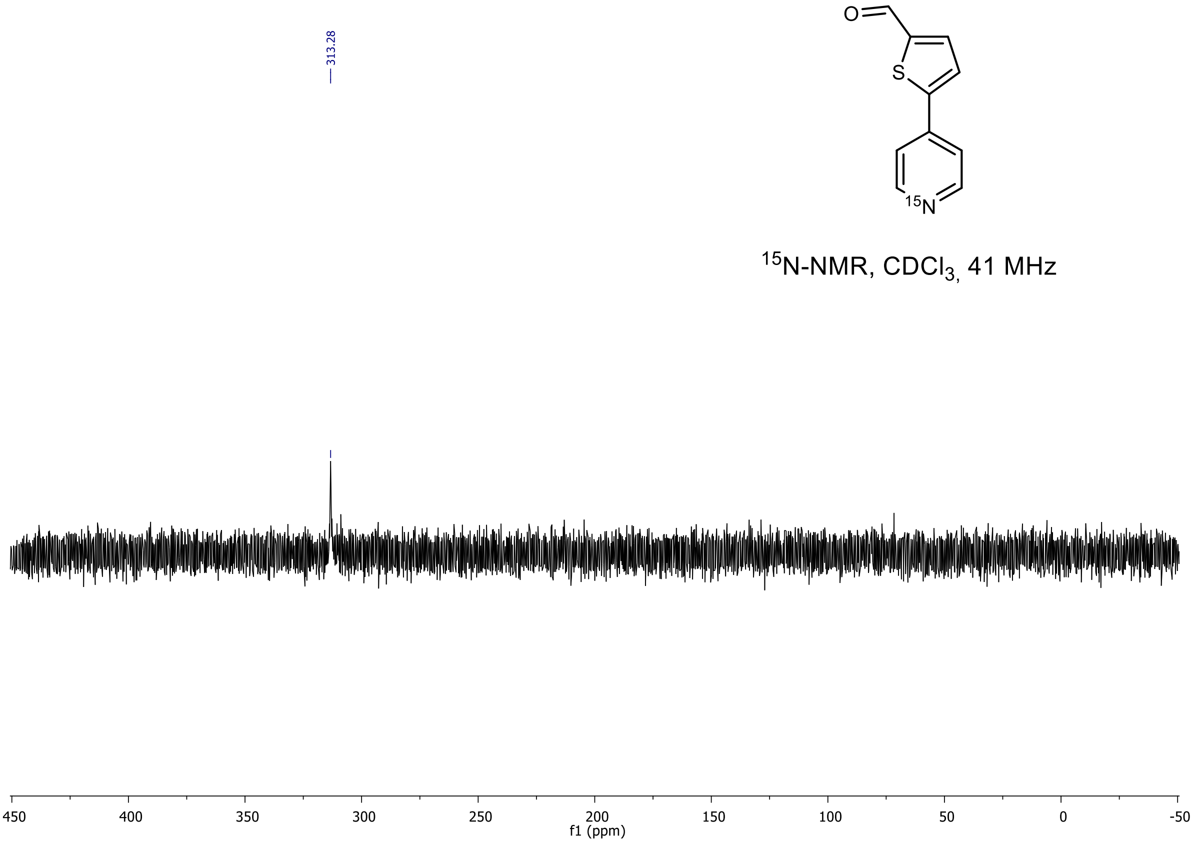

313.28
O
S
^{15}N
^{15}N-NMR, $CDCl_3$, 41 MHz
450 400 350 300 250 200 150 100 50 0 -50
f1 (ppm)

Isotopic Enrichment

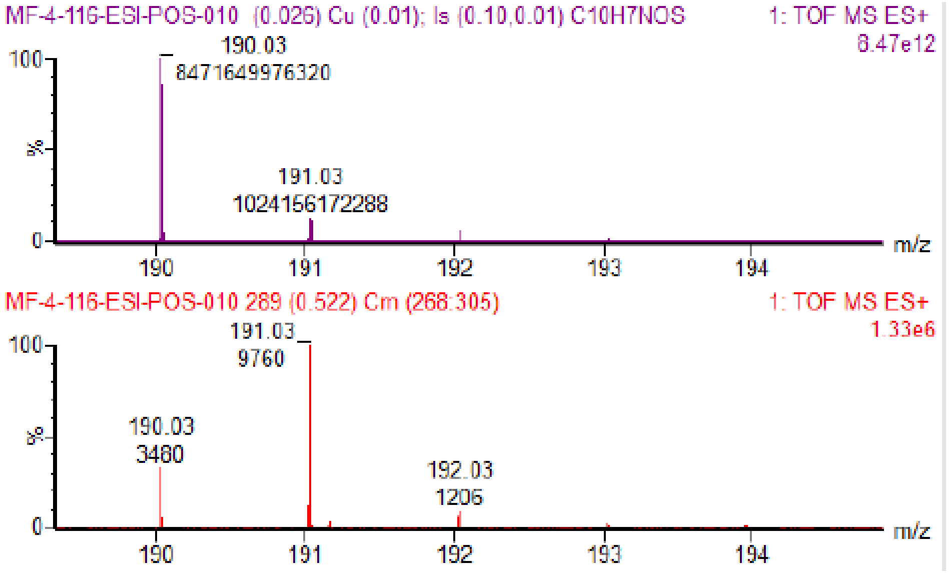

theorical isotopic distribution

	M	M+1	M+2	M+3	M+4	M+5	M+6	M+7
m/z	190	191	192	193	194	195	196	197
%	100,0	12,1	5,2	0,5	0,0	0,0	0,0	0,0

Enrichment calculation

Isotopomer	m/z	Area	natural isotope correction	Corrected area	Isotopic purity (%)
0	190	3480	0,00	3480,00	27,70
1	191	9760	421,08	9338,92	74,32
2	192	1206	180,96	-104,97	-0,84
3	193	344	17,40	-146,32	-1,16
4	194	12	0,00	-11,53	-0,09
5	195	0	0,00	9,53	0,08
6	196	0	0,00	0,18	0,00
7	197	0	0,00	-0,46	0,00
8	198	0	0,00	0,00	0,00
9	199	0	0,00	0,02	0,00
10	200	0	0,00	0,00	0,00
11	201	0	0,00	0,00	0,00
Total		14802		12565,36	100,00

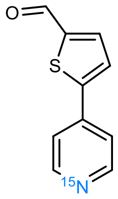

% Isotopic enrichment : 74,3

4-(Pentan-3-yl)pyridine-1-^{15}N ([^{15}N]12)

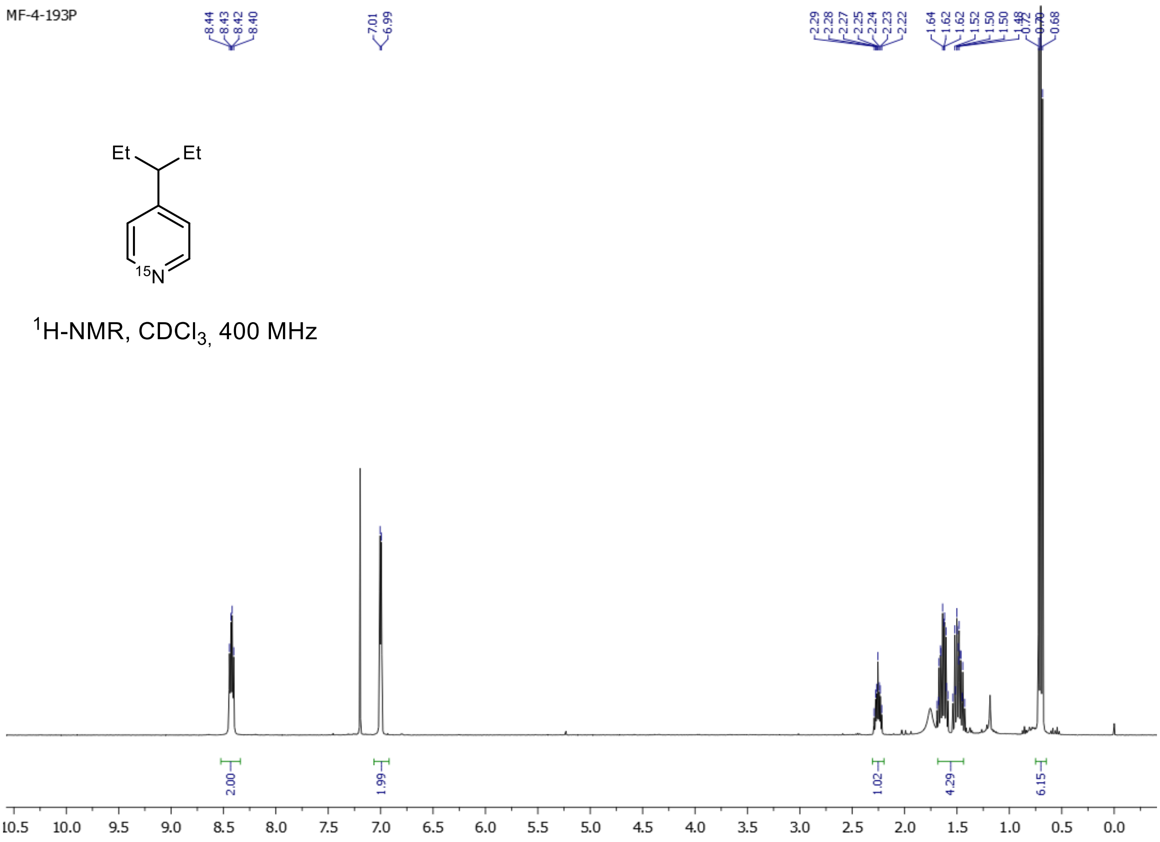

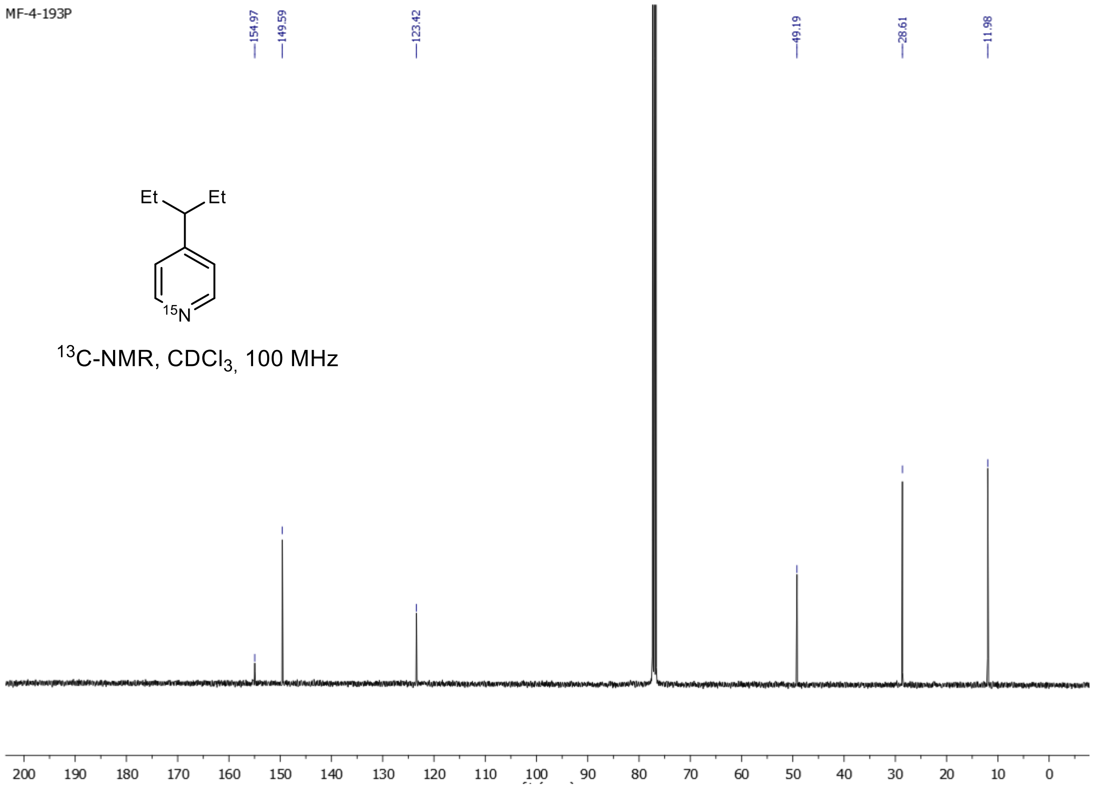

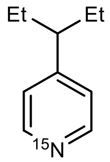

^{15}N-NMR, $CDCl_3$, 41 MHz

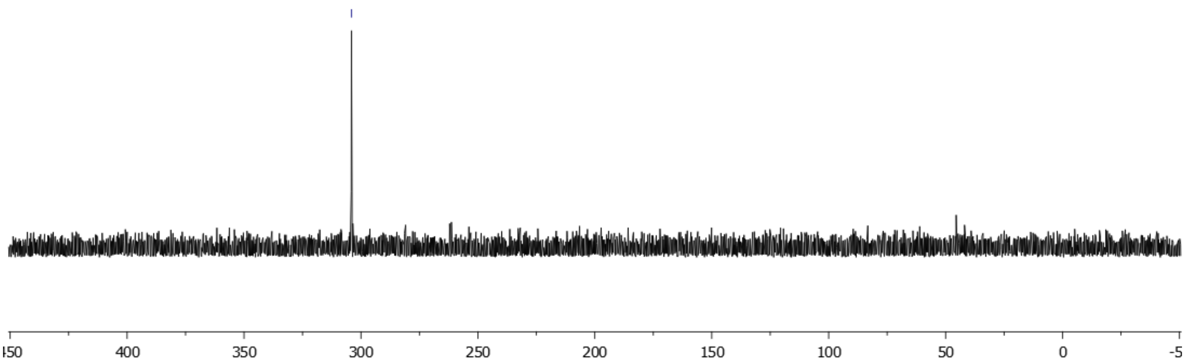

Isotopic Enrichment

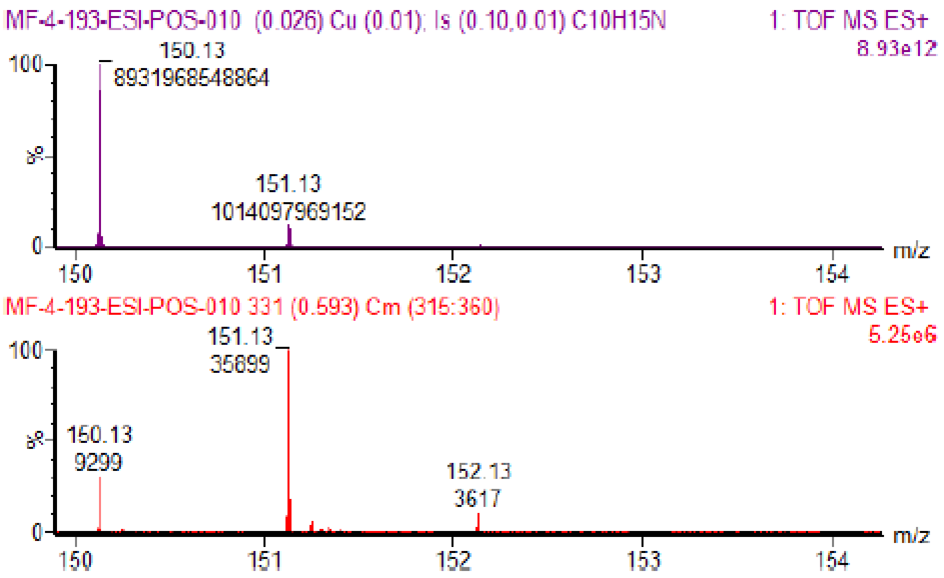

theorical isotopic distribution

	M	M+1	M+2	M+3	M+4	M+5	M+6	M+7
m/z	150	151	152	153	154	155	156	157
%	100,0	11,4	0,5	0,0	0,0	0,0	0,0	0,0

Enrichment calculation

Isotopomer	m/z	Area	natural isotope correction	Corrected area	Isotopic purity (%)
0	150	9299	0,00	9299,00	21,29
1	151	35899	1060,09	34838,91	79,76
2	152	3617	46,50	-401,13	-0,92
3	153	59	0,00	-69,47	-0,16
4	154	2	0,00	11,92	0,03
5	155	0	0,00	-1,01	0,00
6	156	0	0,00	0,06	0,00
7	157	0	0,00	0,00	0,00
8	158	0	0,00	0,00	0,00
9	159	0	0,00	0,00	0,00
10	160	0	0,00	0,00	0,00
11	161	0	0,00	0,00	0,00
Total		48876		43678,28	100,00

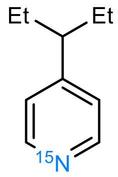

% Isotopic enrichment : 79,8

4-(Benzyloxy)pyridine-1-^{15}N ([^{15}N]13)

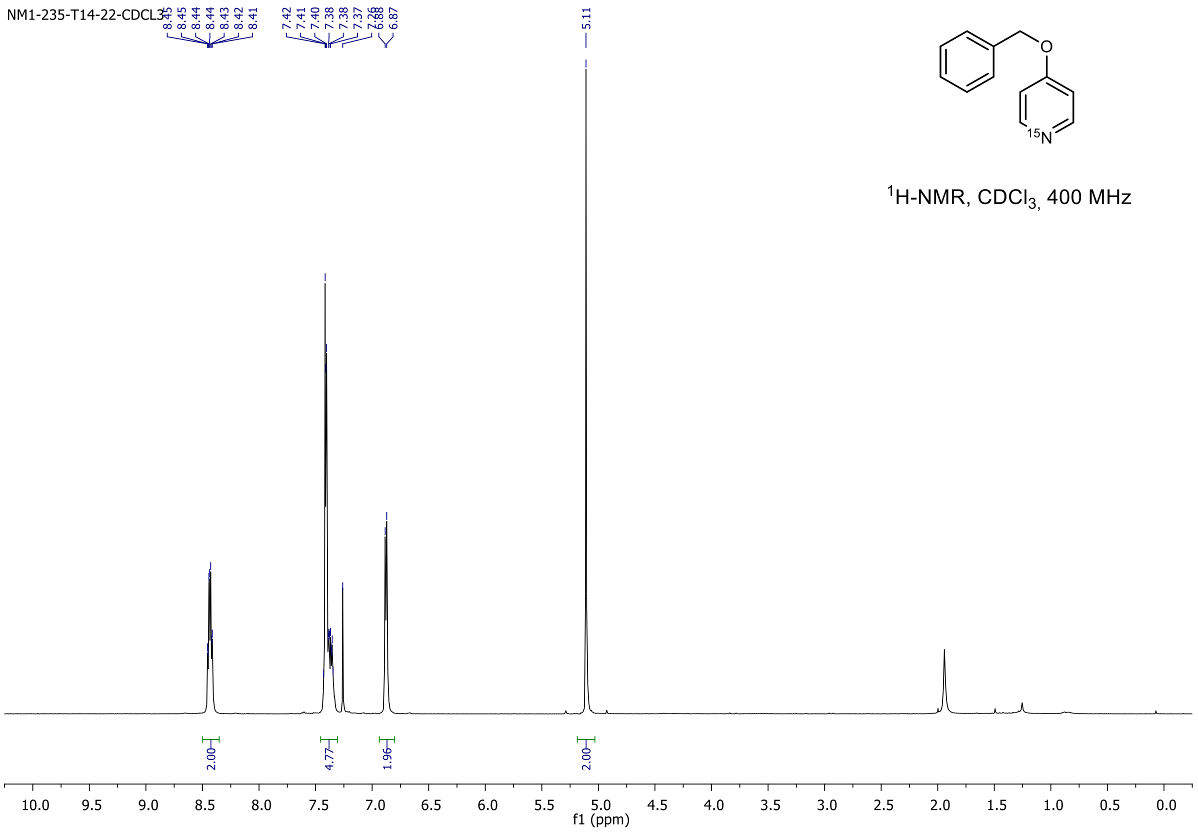

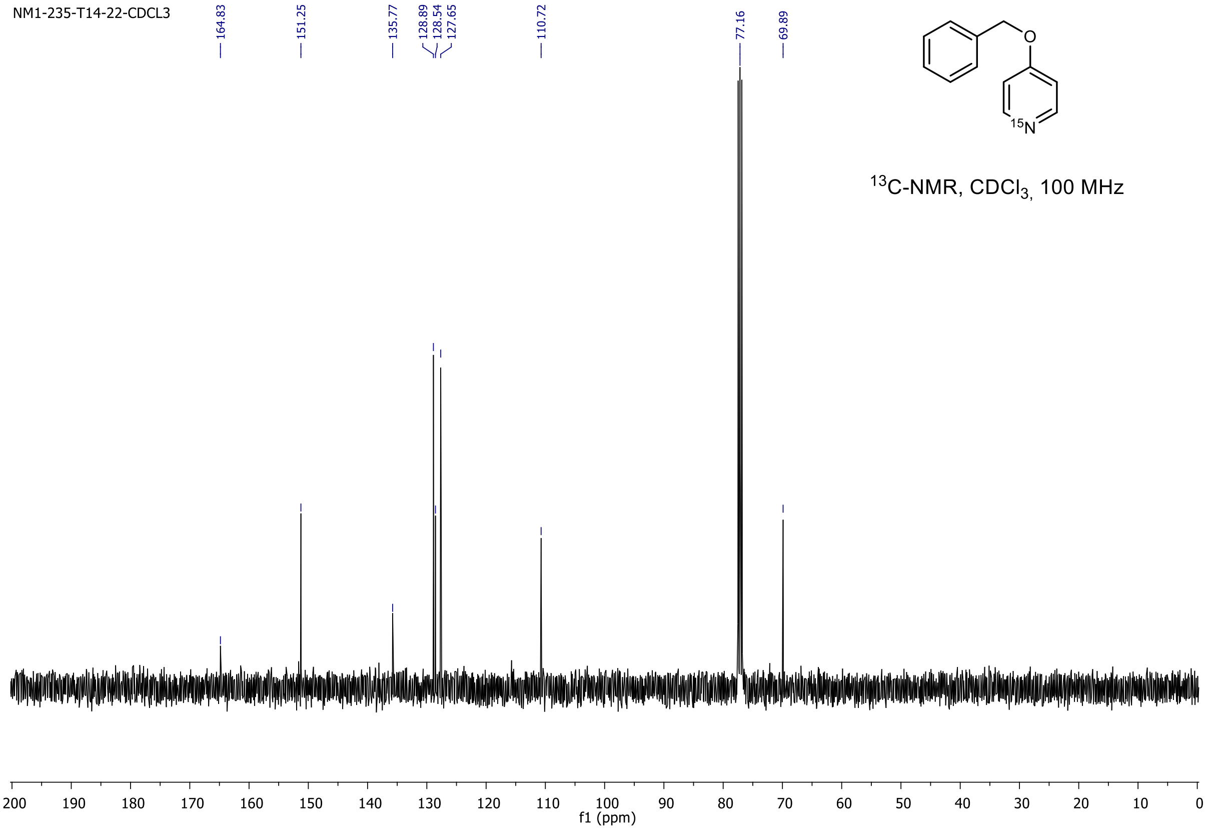

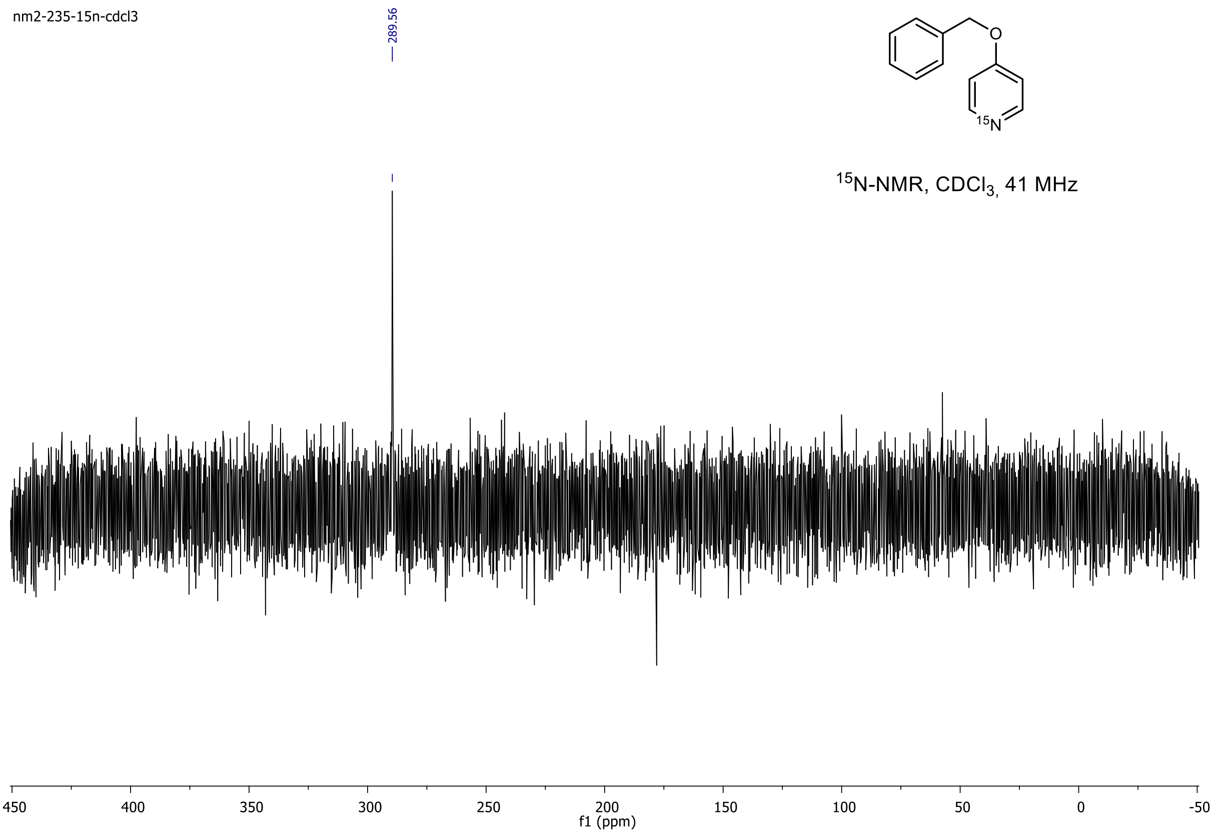
nm2-235-15n-cdcl3
289.56
15N
15N-NMR, CDCl3, 41 MHz
450
400
350
300
250
200
150
100
50
0
-50
f1 (ppm)

Isotopic Enrichment

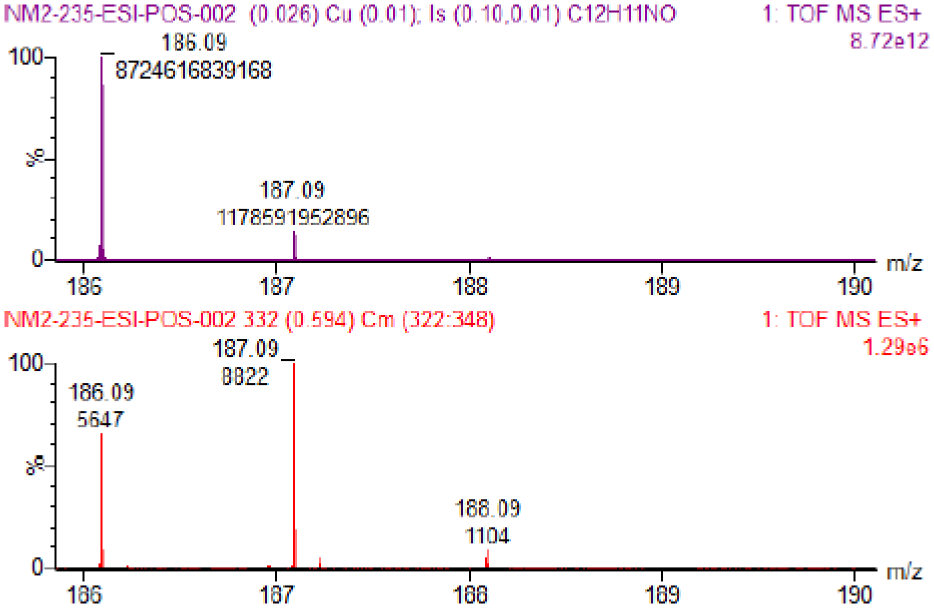

theorical isotopic distribution

	M	M+1	M+2	M+3	M+4	M+5	M+6	M+7
**m/z**	186	187	188	189	190	191	192	193
**%**	100,0	13,5	1,0	0,0	0,0	0,0	0,0	0,0

Enrichment calculation

Isotopomer	m/z	Area	natural isotope correction	Corrected area	Isotopic purity (%)
0	186	5647	0,00	5647,00	41,43
1	187	8822	762,35	8059,66	59,13
2	188	1104	56,47	-40,52	-0,30
3	189	32	0,00	-43,13	-0,32
4	190	1	0,00	7,23	0,05
5	191	0	0,00	-0,54	0,00
6	192	0	0,00	0,00	0,00
7	193	0	0,00	0,01	0,00
8	194	0	0,00	0,00	0,00
9	195	0	0,00	0,00	0,00
10	196	0	0,00	0,00	0,00
11	197	0	0,00	0,00	0,00
Total		15606		13629,69	100,00

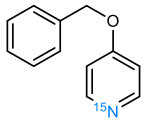

% Isotopic enrichment : 59,1

Tert-butyl 2-(pyridin-3-yl-^{15}N)-1H-pyrrole-1-carboxylate ([^{15}N]22)

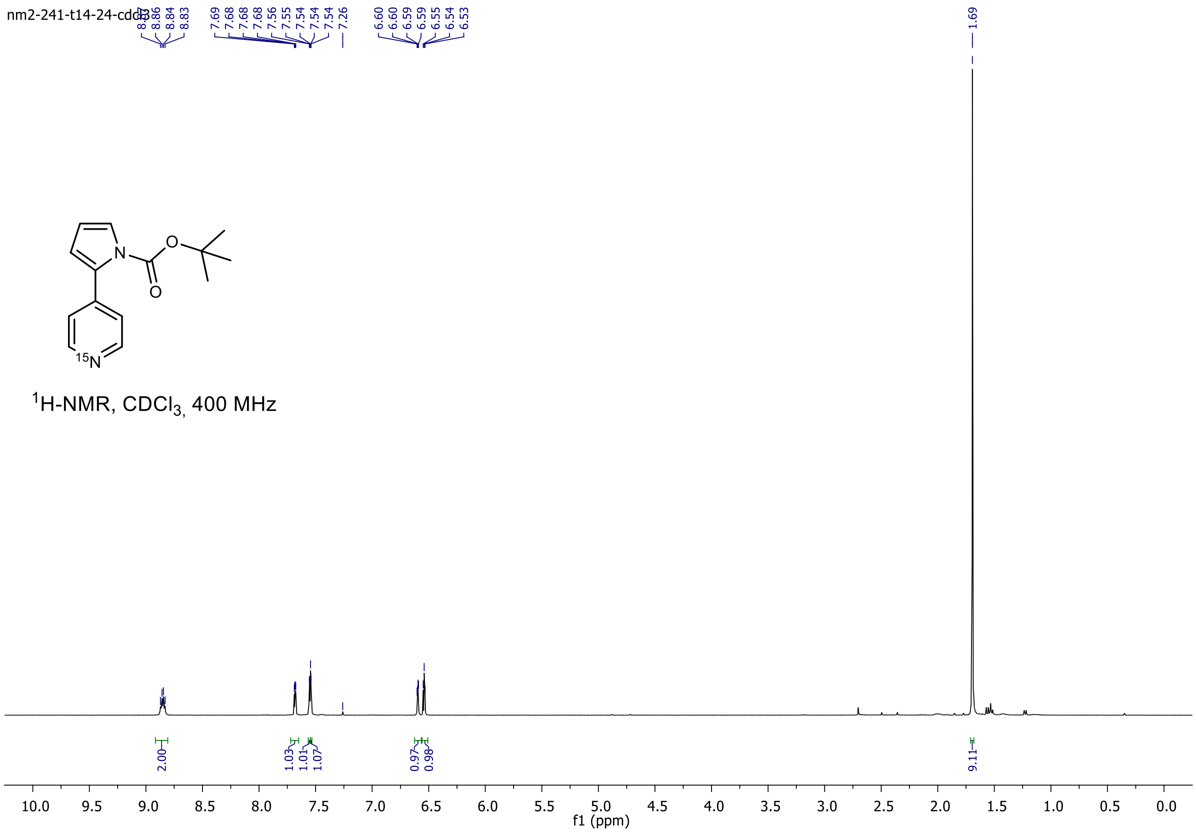

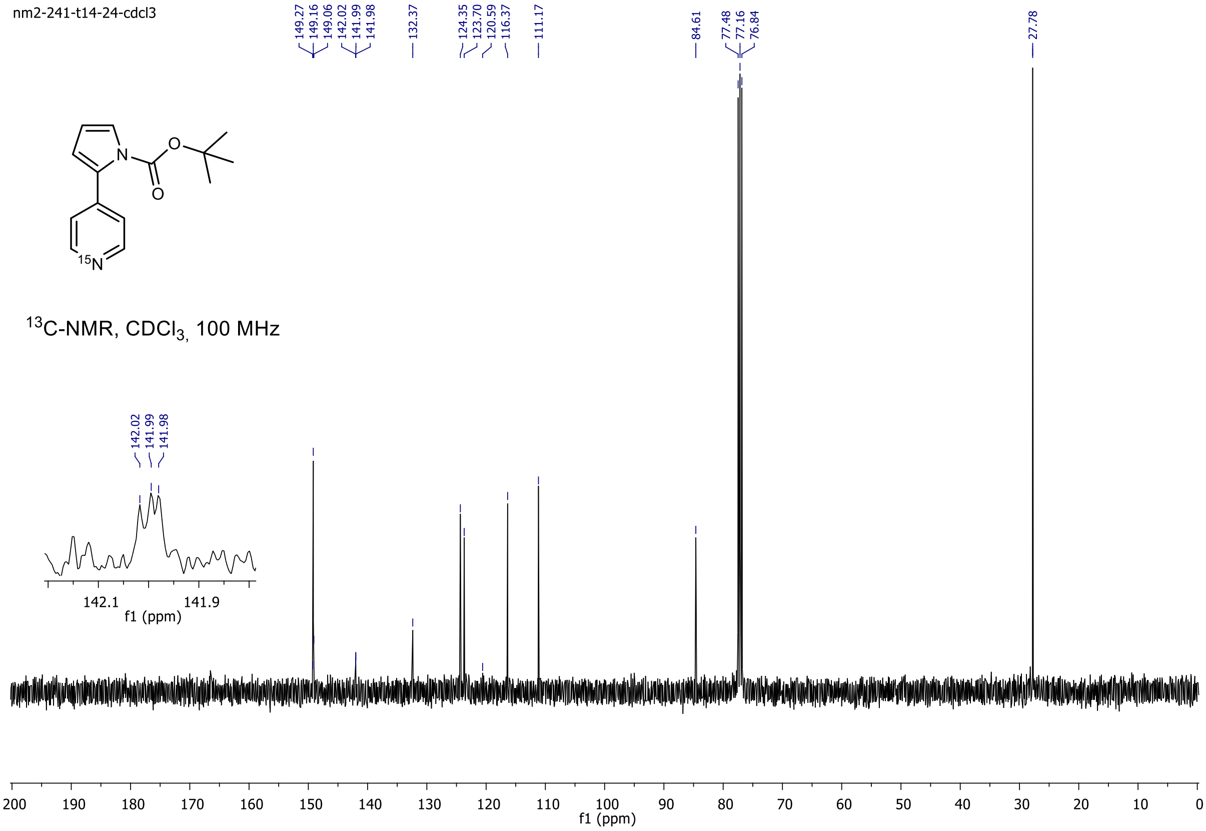

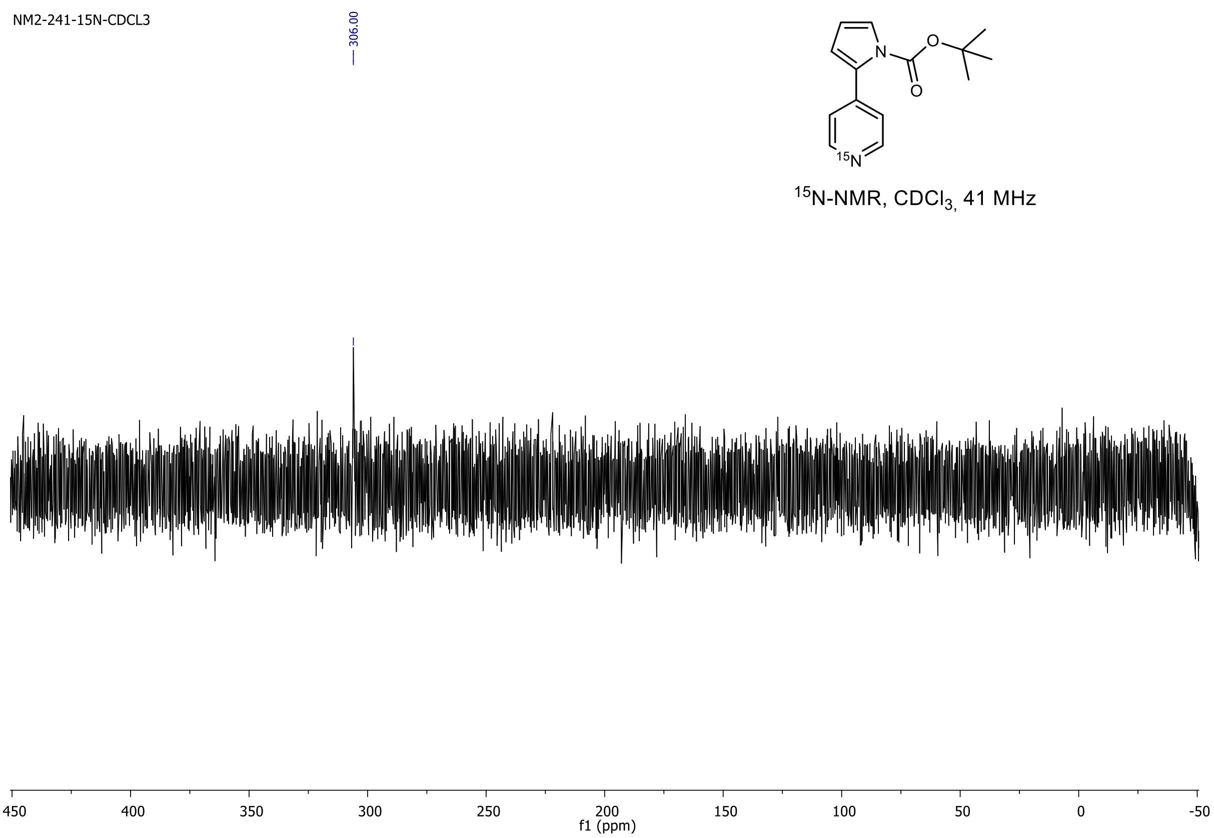
NM2-241-15N-CDCL3
306.00
15N
O
N
O
15N-NMR, CDCl3, 41 MHz
450
400
350
300
250
200
150
100
50
0
-50
f1 (ppm)

Isotopic Enrichment

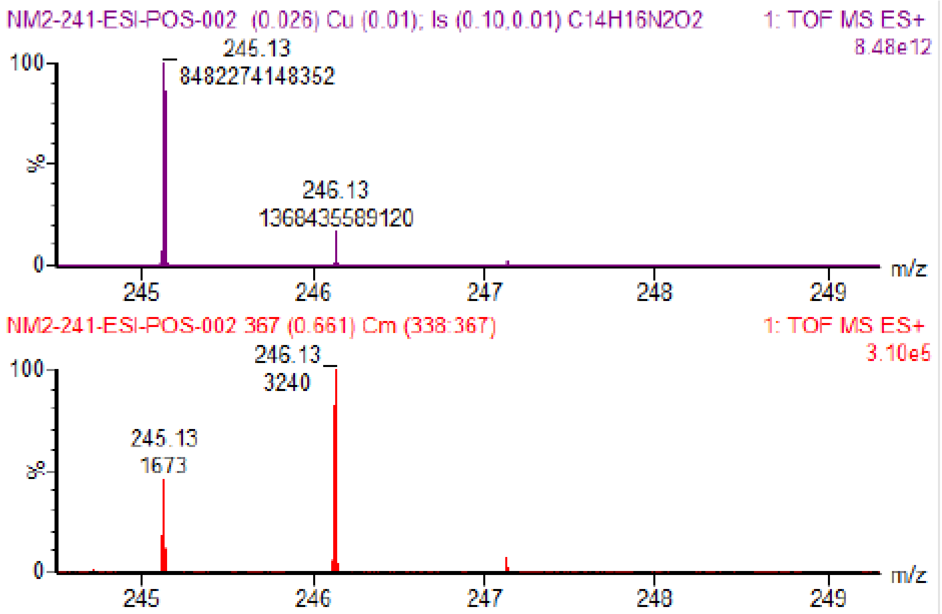

theorical isotopic distribution

	M	M+1	M+2	M+3	M+4	M+5	M+6	M+7
**m/z**	245	246	247	248	249	250	251	252
**%**	100,0	16,2	1,6	0,1	0,0	0,0	0,0	0,0

Enrichment calculation

Isotopomer	m/z	Area	natural isotope correction	Corrected area	Isotopic purity (%)
0	245	1673	0,00	1673,00	37,39
1	246	3240	271,03	2968,97	66,36
2	247	344	26,77	-163,74	-3,66
3	248	17	1,67	-5,65	-0,13
4	249	1	0,00	1,57	0,04
5	250	0	0,00	0,00	0,00
6	251	0	0,00	-0,02	0,00
7	252	0	0,00	0,00	0,00
8	253	0	0,00	0,00	0,00
9	254	0	0,00	0,00	0,00
10	255	0	0,00	0,00	0,00
11	256	0	0,00	0,00	0,00
Total		5275		4474,13	100,00

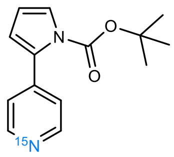

% Isotopic enrichment : 66,4

5-(Pyridin-4-yl-^{15}N)thiophene-2-carbaldehyde ([^{15}N]14)

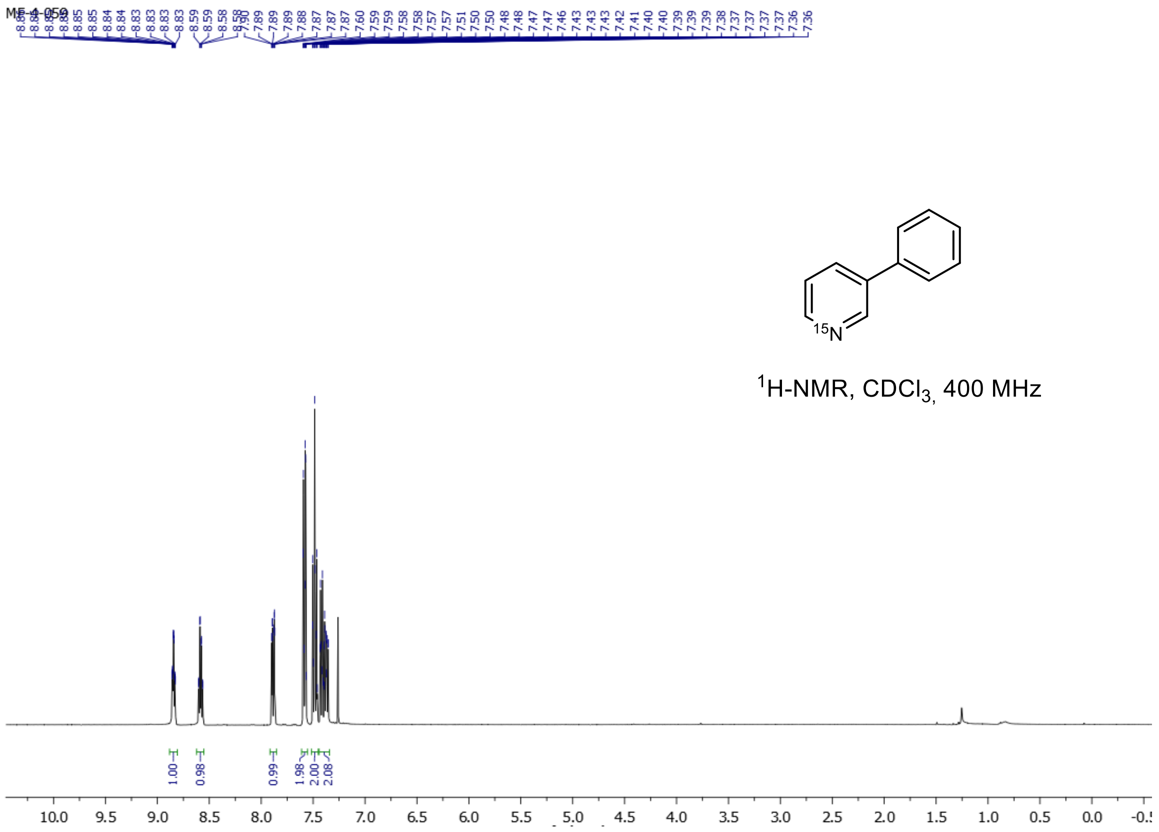

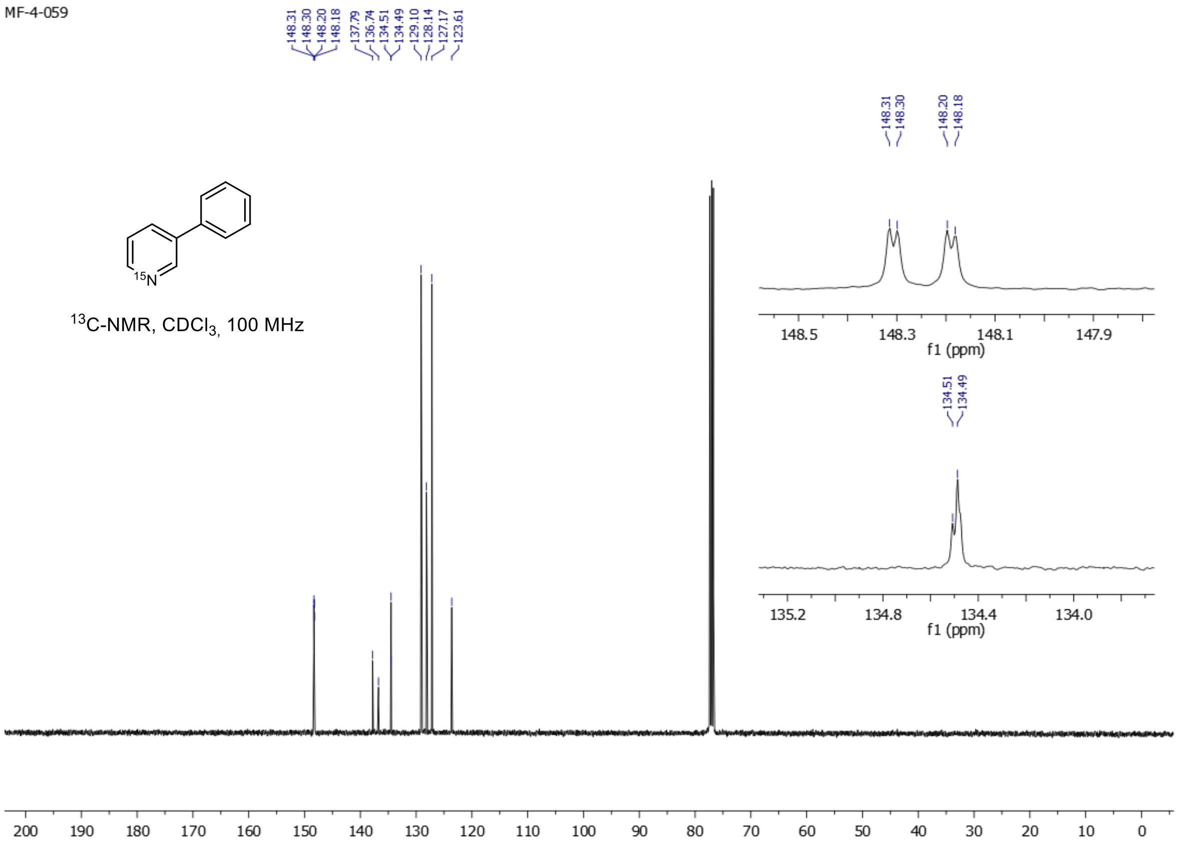

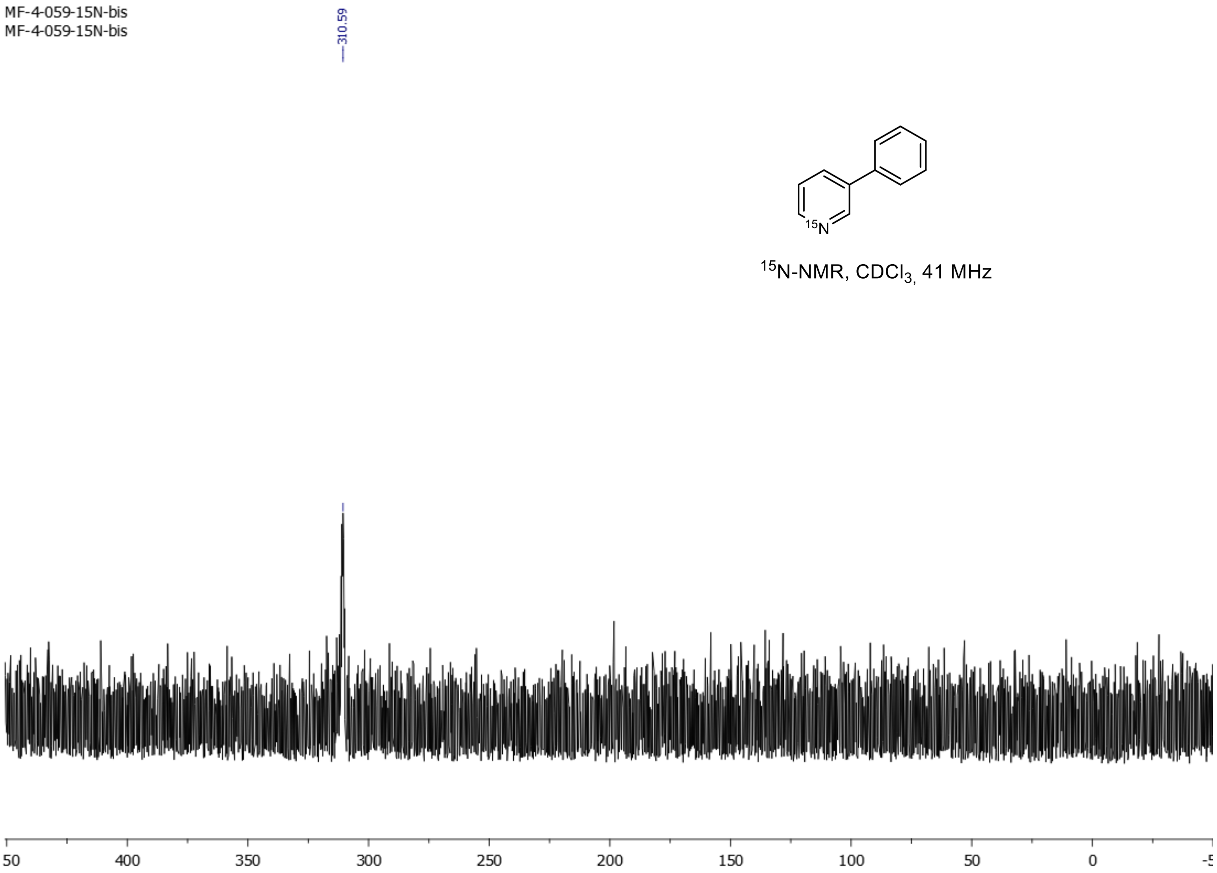
MF-4-059-15N-bis
MF-4-059-15N-bis
310.59
15N
15N-NMR, CDCl3, 41 MHz
50
400
350
300
250
200
150
100
50
0

Isotopic Enrichment

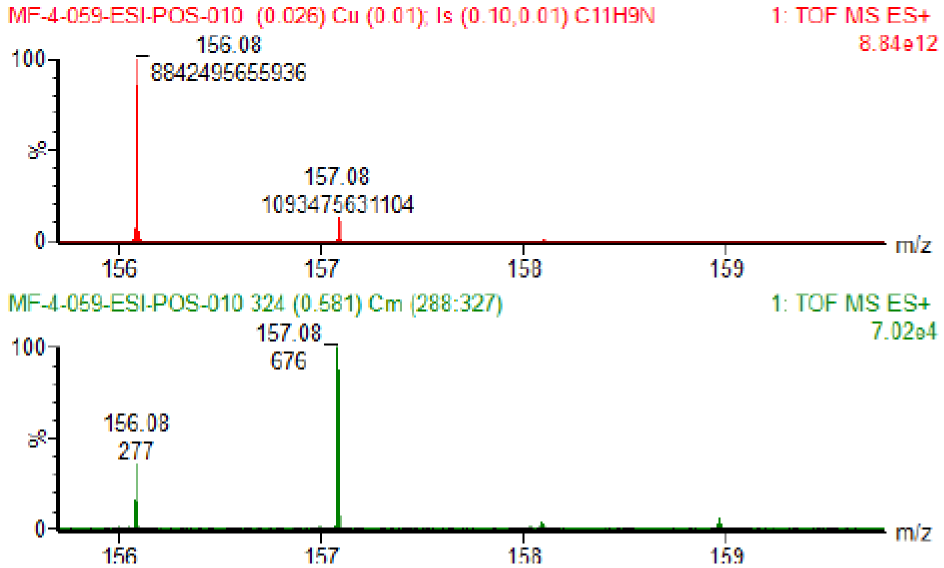

theorical isotopic distribution

	M	M+1	M+2	M+3	M+4	M+5	M+6	M+7
m/z	156	157	158	159	160	161	162	163
%	100,0	12,4	0,6	0,0	0,0	0,0	0,0	0,0

Enrichment calculation

Isotopomer	m/z	Area	natural isotope correction	Corrected area	Isotopic purity (%)
0	156	277	0,00	277,00	31,43
1	157	676	34,35	641,65	72,80
2	158	41	1,66	-40,23	-4,56
3	159	2	0,00	3,14	0,36
4	160	0	0,00	-0,15	-0,02
5	161	0	0,00	0,00	0,00
6	162	0	0,00	0,00	0,00
7	163	0	0,00	0,00	0,00
8	164	0	0,00	0,00	0,00
9	165	0	0,00	0,00	0,00
10	166	0	0,00	0,00	0,00
11	167	0	0,00	0,00	0,00
Total		996		881,42	100,00

% Isotopic enrichment : 72,8

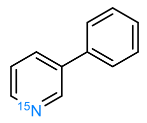

IE obtained following procedure A

Isotopic Enrichment

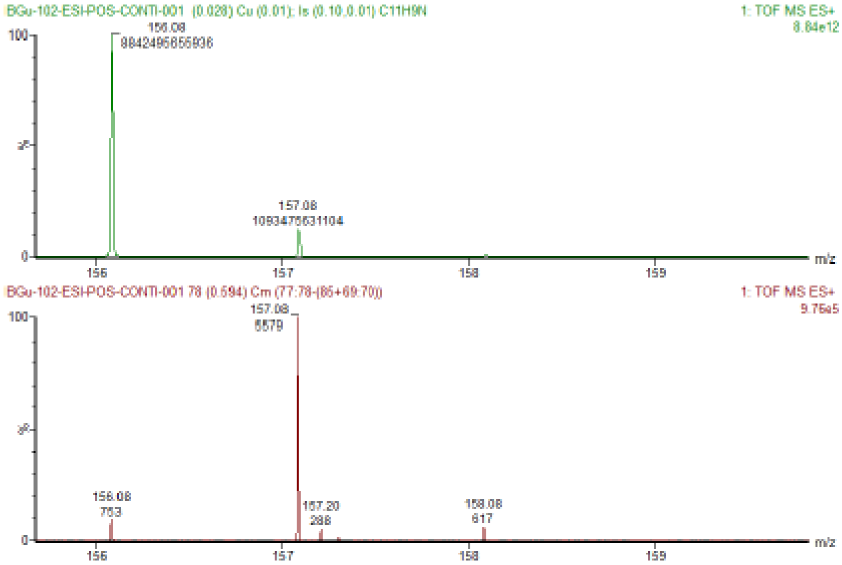

theorical isotopic distribution

	M	M+1	M+2	M+3	M+4	M+5	M+6	M+7
m/z	156	157	158	159	160	161	162	163
%	100,0	12,4	0,6	0,0	0,0	0,0	0,0	0,0

Enrichment calculation

Isotopomer	m/z	Area	natural isotope correction	Corrected area	Isotopic purity (%)
0	156	1332	0,00	1332,00	15,50
1	157	7495	165,17	7329,83	85,30
2	158	871	7,99	-45,89	-0,53
3	159	12	0,00	-26,29	-0,31
4	160	0	0,00	3,54	0,04
5	161	0	0,00	-0,28	0,00
6	162	0	0,00	0,01	0,00
7	163	0	0,00	0,00	0,00
8	164	0	0,00	0,00	0,00
9	165	0	0,00	0,00	0,00
10	166	0	0,00	0,00	0,00
11	167	0	0,00	0,00	0,00
Total		9710		8592,92	100,00

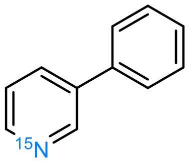

IE obtained following procedure E

% Isotopic enrichment : 85,3

Ethyl nicotinate-^{15}N ([^{15}N]15)

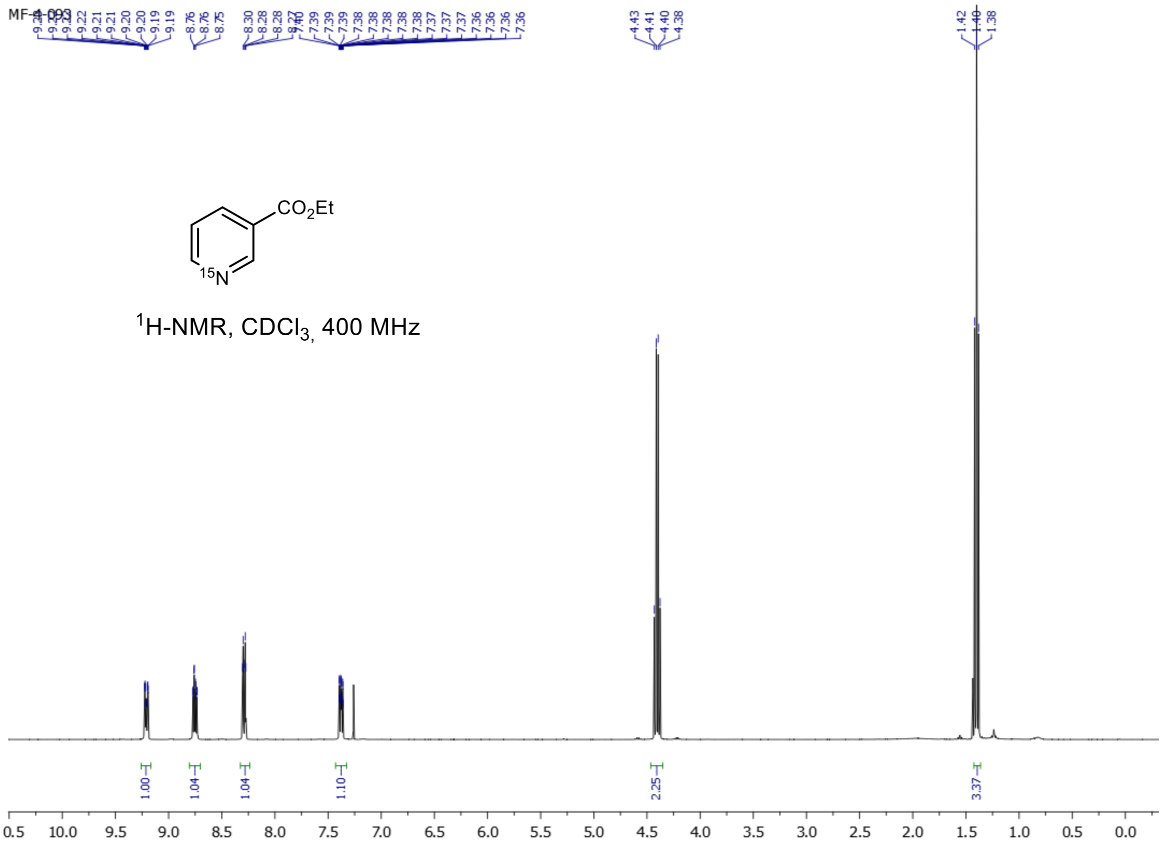

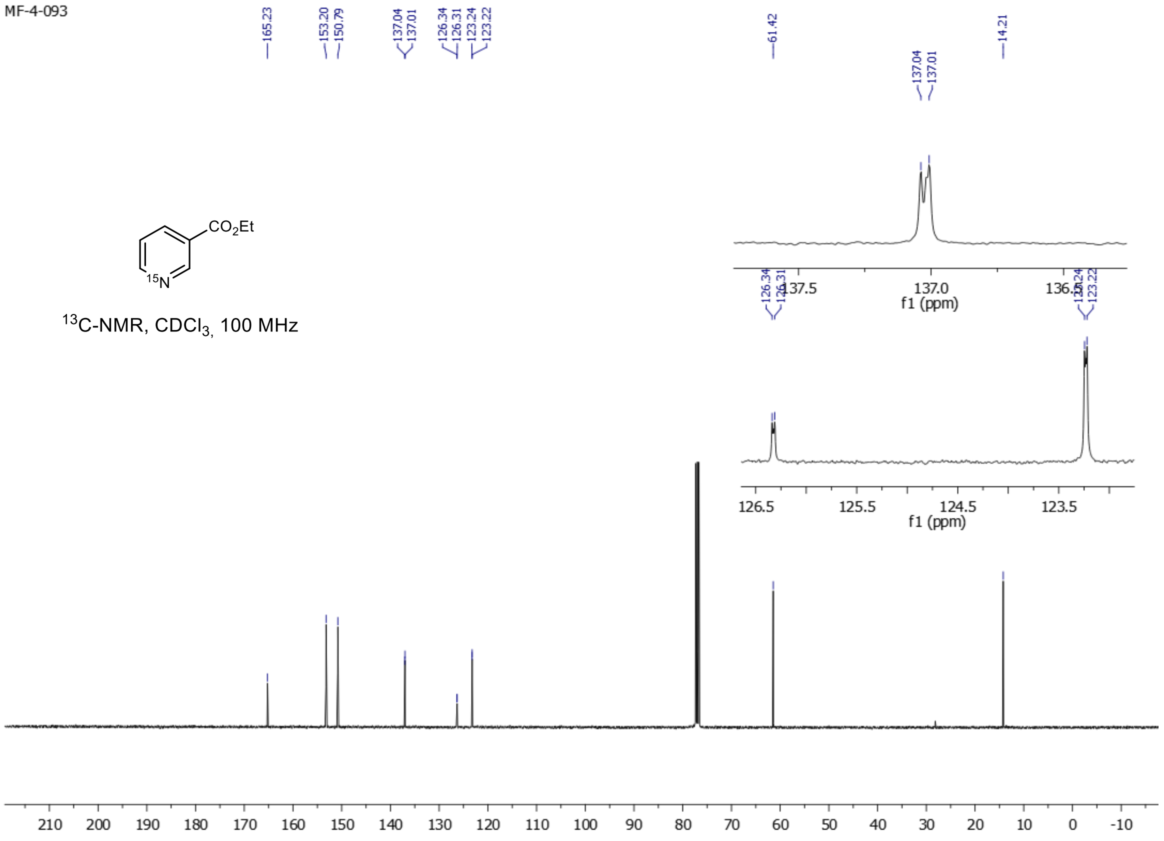

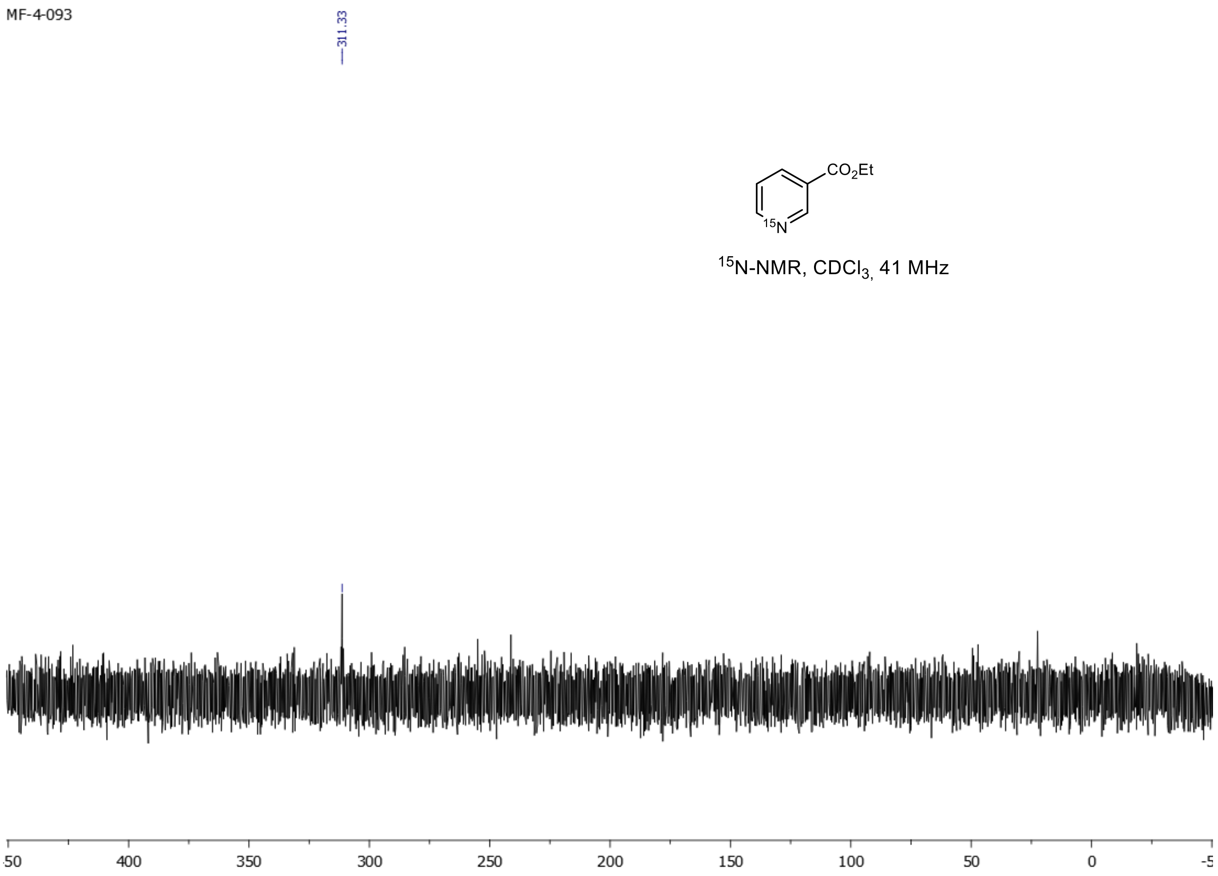

MF-4-093
311.33
CO_2Et
^{15}N
^{15}N-NMR, $CDCl_3$, 41 MHz
50
400
350
300
250
200
150
100
50
0

Isotopic Enrichment

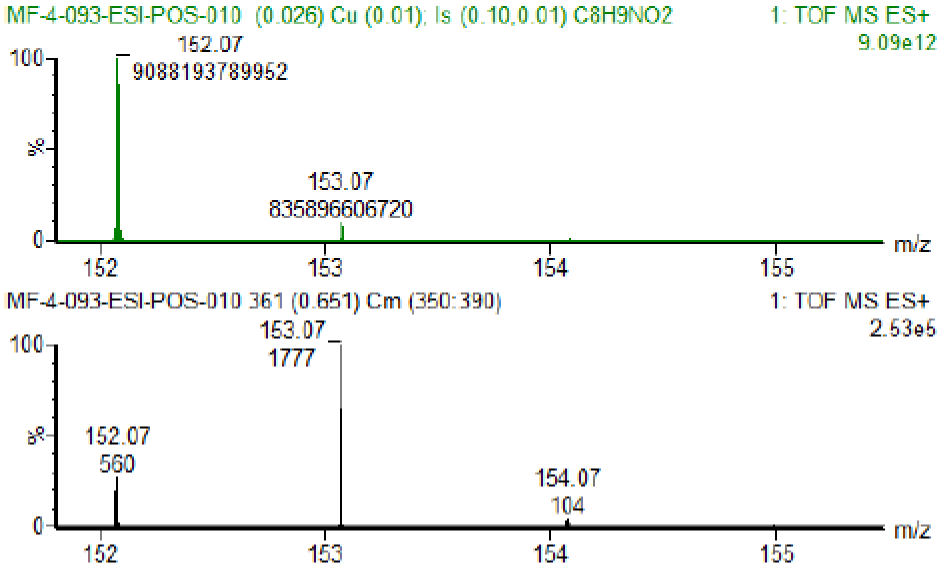

theorical isotopic distribution

	M	M+1	M+2	M+3	M+4	M+5	M+6	M+7
**m/z**	152	153	154	155	156	157	158	159
**%**	100,0	9,3	0,7	0,0	0,0	0,0	0,0	0,0

Enrichment calculation

Isotopomer	m/z	Area	natural isotope correction	Corrected area	Isotopic purity (%)
0	152	560	0,00	560,00	25,14
1	153	1777	52,08	1724,92	77,45
2	154	104	3,92	-60,34	-2,71
3	155	9	0,00	2,54	0,11
4	156	0	0,00	0,19	0,01
5	157	0	0,00	-0,04	0,00
6	158	0	0,00	0,00	0,00
7	159	0	0,00	0,00	0,00
8	160	0	0,00	0,00	0,00
9	161	0	0,00	0,00	0,00
10	162	0	0,00	0,00	0,00
11	163	0	0,00	0,00	0,00
Total		2450		2227,27	100,00

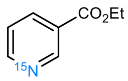

% Isotopic enrichment : 77,4

1-(Pyridin-3-yl-^{15}N)propan-1-one ([^{15}N]16)

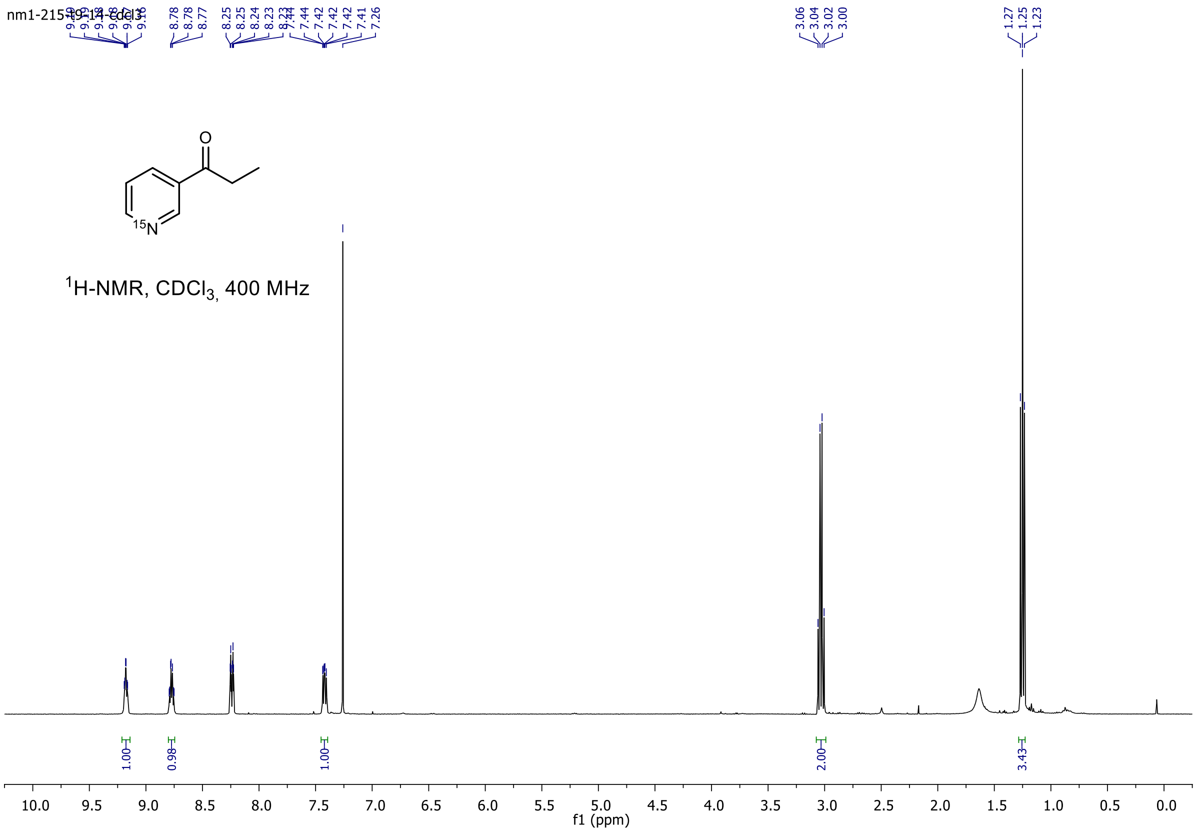

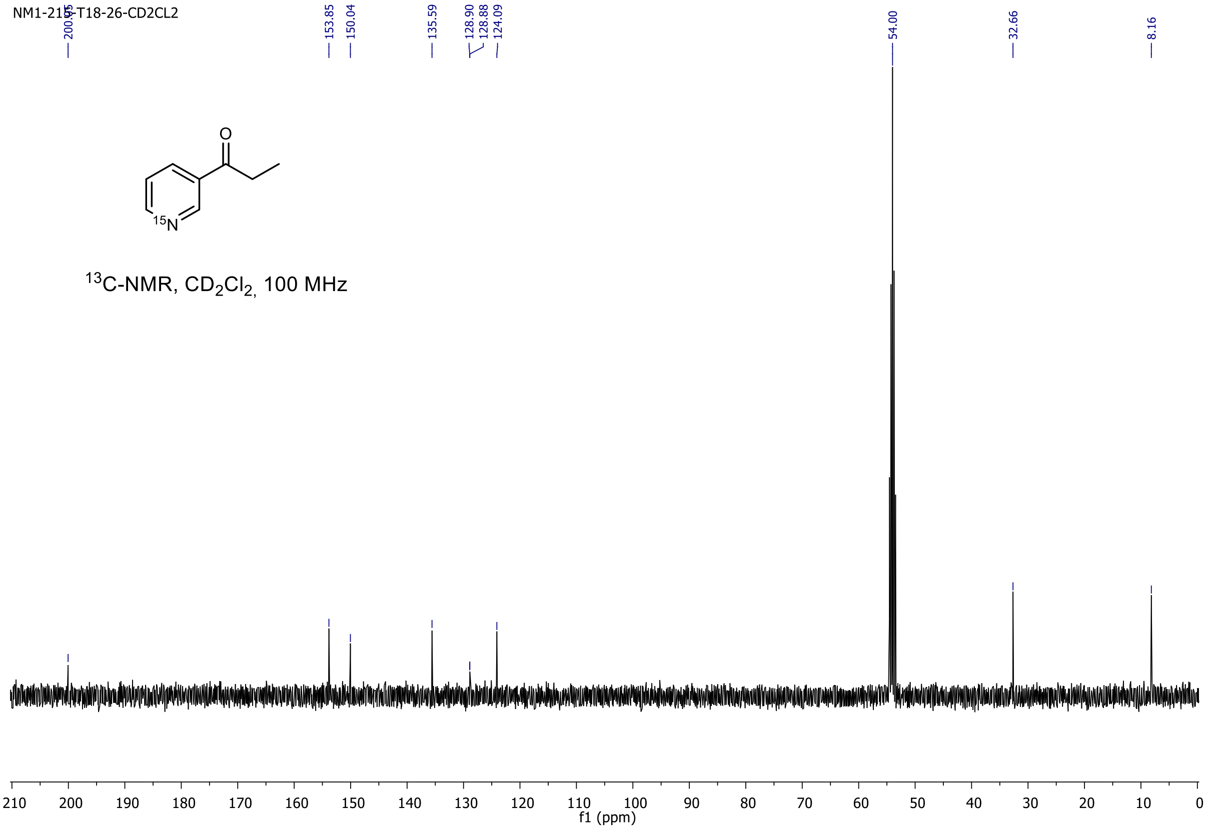

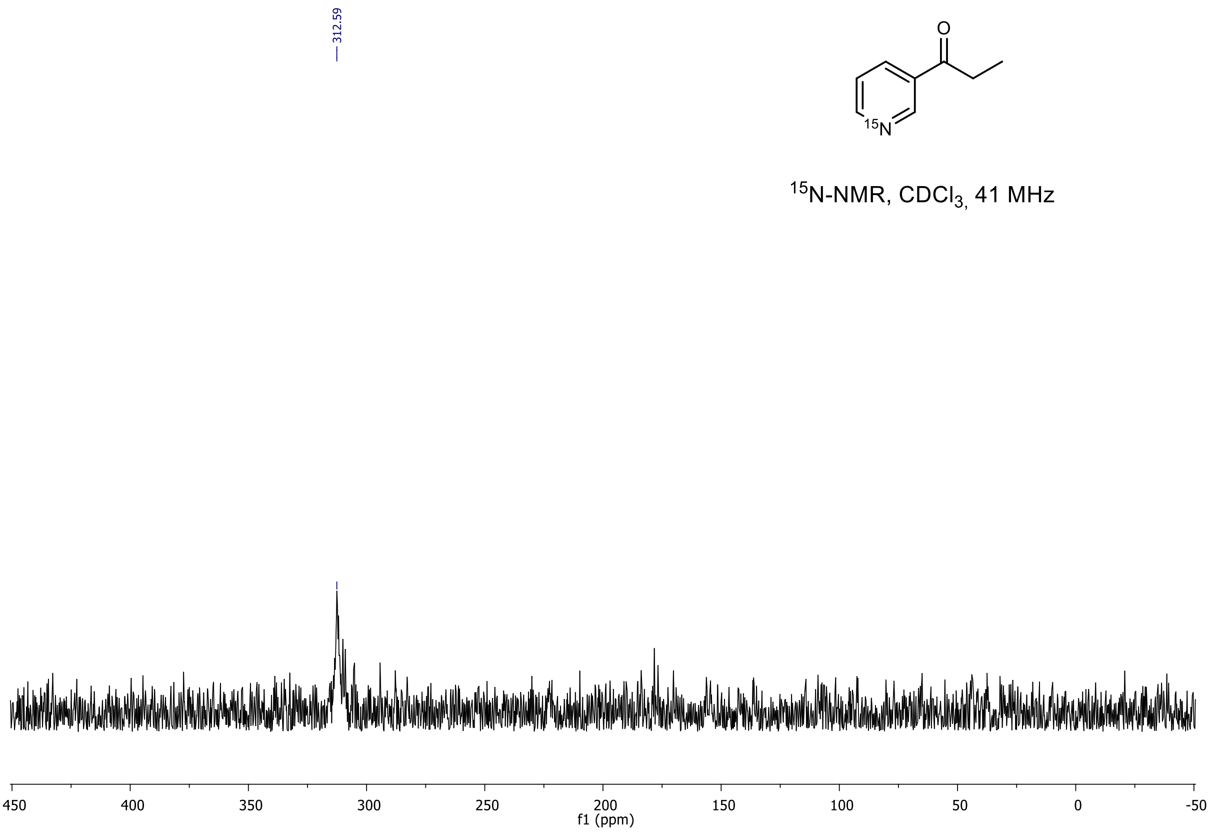
312.59
O
15N
15N-NMR, CDCl3, 41 MHz
450
400
350
300
250
200
150
100
50
0
-50
f1 (ppm)

Isotopic Enrichment

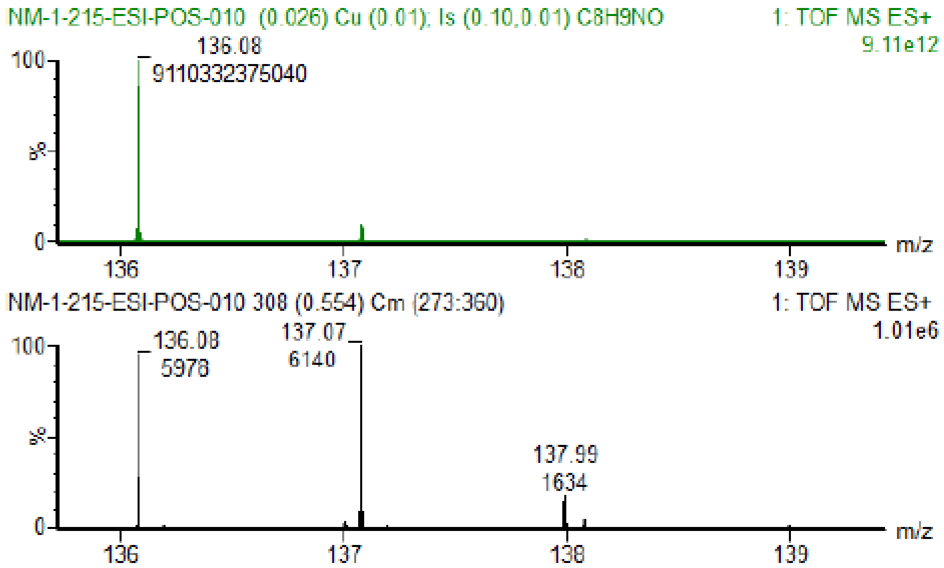

theorical isotopic distribution

	M	M+1	M+2	M+3	M+4	M+5	M+6	M+7
**m/z**	136	137	138	139	140	141	142	143
**%**	100,0	9,2	0,5	0,0	0,0	0,0	0,0	0,0

Enrichment calculation

Isotopomer	m/z	Area	natural isotope correction	Corrected area	Isotopic purity (%)
0	136	5978	0,00	5978,00	52,27
1	137	6140	549,98	5590,02	48,87
2	138	412	29,89	-132,17	-1,16
3	139	17	0,00	1,21	0,01
4	140	0	0,00	0,55	0,00
5	141	0	0,00	-0,06	0,00
6	142	0	0,00	0,00	0,00
7	143	0	0,00	0,00	0,00
8	144	0	0,00	0,00	0,00
9	145	0	0,00	0,00	0,00
10	146	0	0,00	0,00	0,00
11	147	0	0,00	0,00	0,00
Total		12547		11437,56	100,00

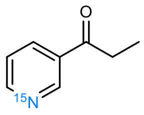

% Isotopic enrichment : 48,9

N,N-Dimethylpyridine-3-sulfonamide-1-^{15}N ([^{15}N]17)

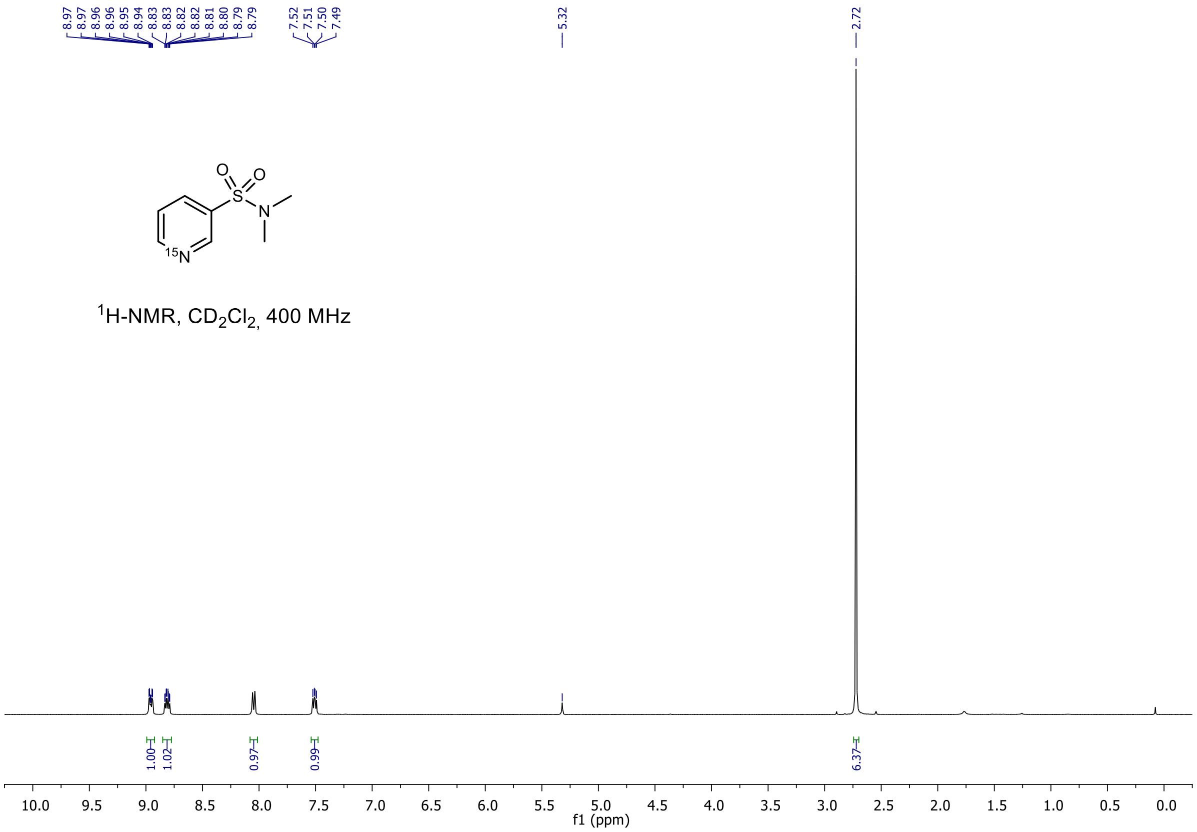

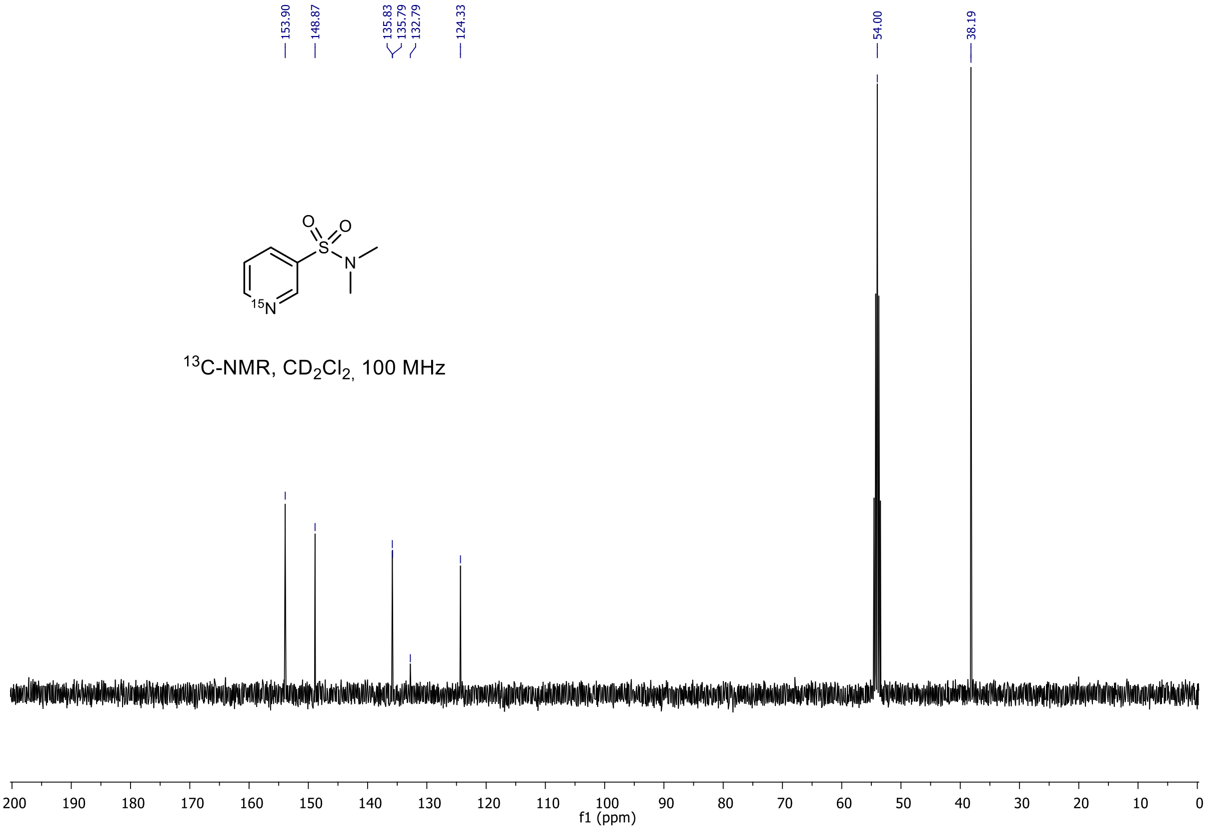

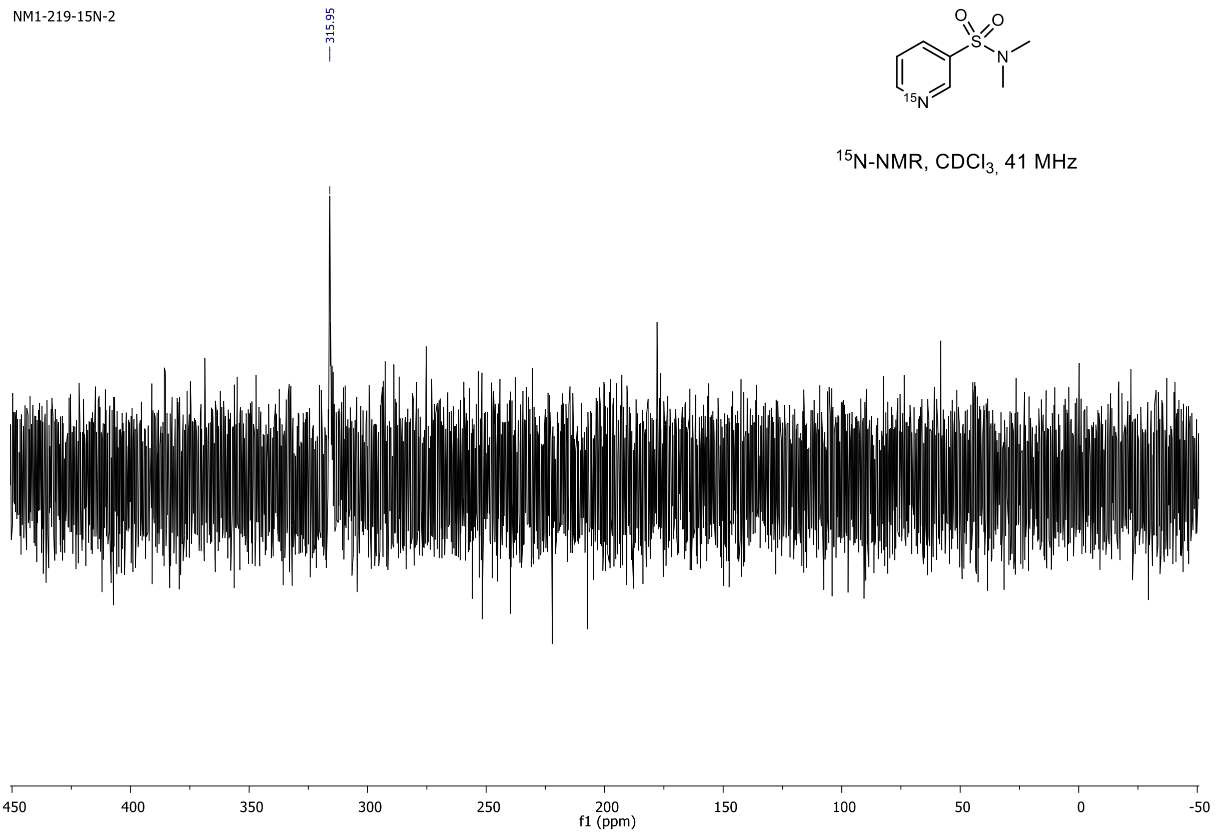
NM1-219-15N-2
315.95
O
O
S
N
15N
^{15}N-NMR, $CDCl_3$, 41 MHz
450
400
350
300
250
200
150
100
50
0
-50
f1 (ppm)

Isotopic Enrichment

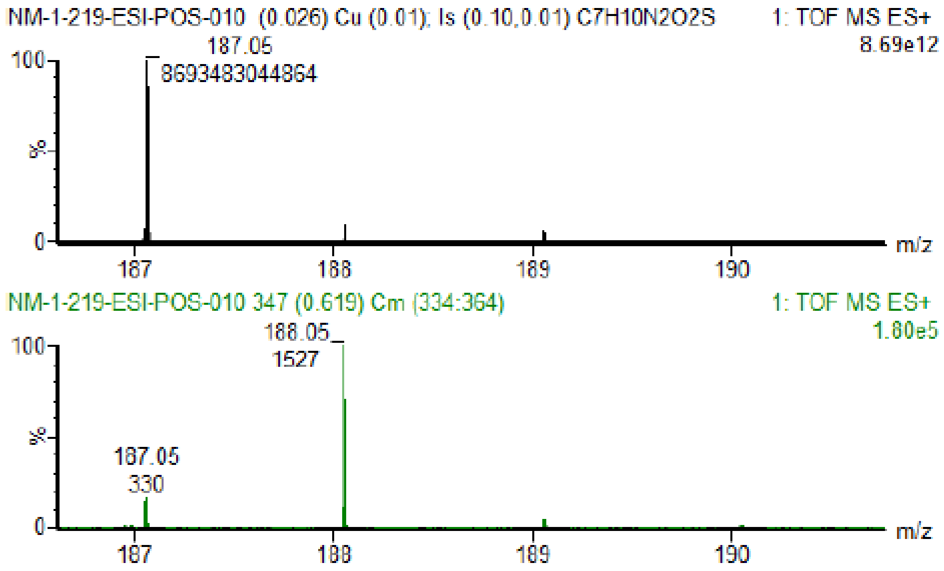

theorical isotopic distribution

	M	M+1	M+2	M+3	M+4	M+5	M+6	M+7
**m/z**	187	188	189	190	191	192	193	194
**%**	100,0	9,3	5,2	0,3	0,0	0,0	0,0	0,0

Enrichment calculation

Isotopomer	m/z	Area	natural isotope correction	Corrected area	Isotopic purity (%)
0	187	330	0,00	330,00	19,13
1	188	1527	30,69	1496,31	86,76
2	189	88	17,16	-68,32	-3,96
3	190	34	0,99	-38,44	-2,23
4	191	1	0,00	3,64	0,21
5	192	0	0,00	1,87	0,11
6	193	0	0,00	-0,25	-0,01
7	194	0	0,00	-0,08	0,00
8	195	0	0,00	0,02	0,00
9	196	0	0,00	0,00	0,00
10	197	0	0,00	0,00	0,00
11	198	0	0,00	0,00	0,00
Total		1980		1724,74	100,00

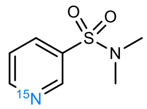

% Isotopic enrichment : 86,8

3-Phenoxypyridine-1-^{15}N ([^{15}N]18)

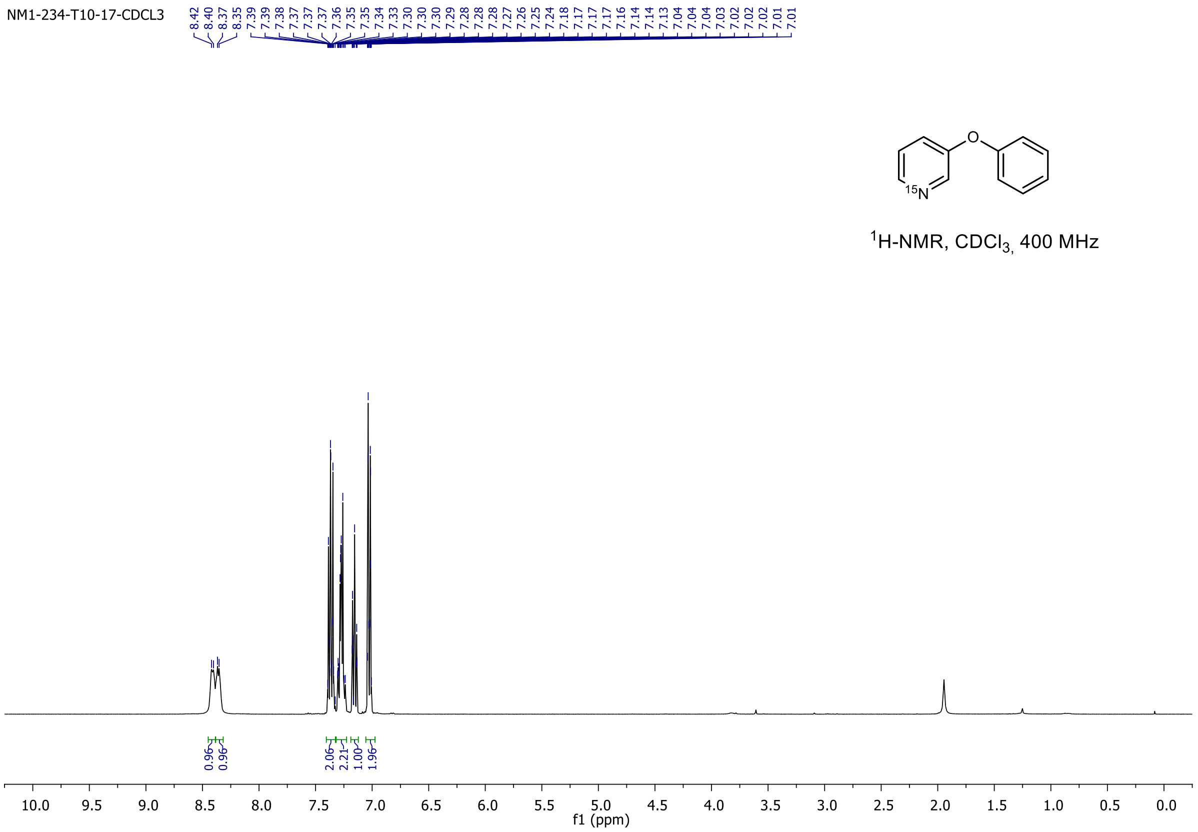

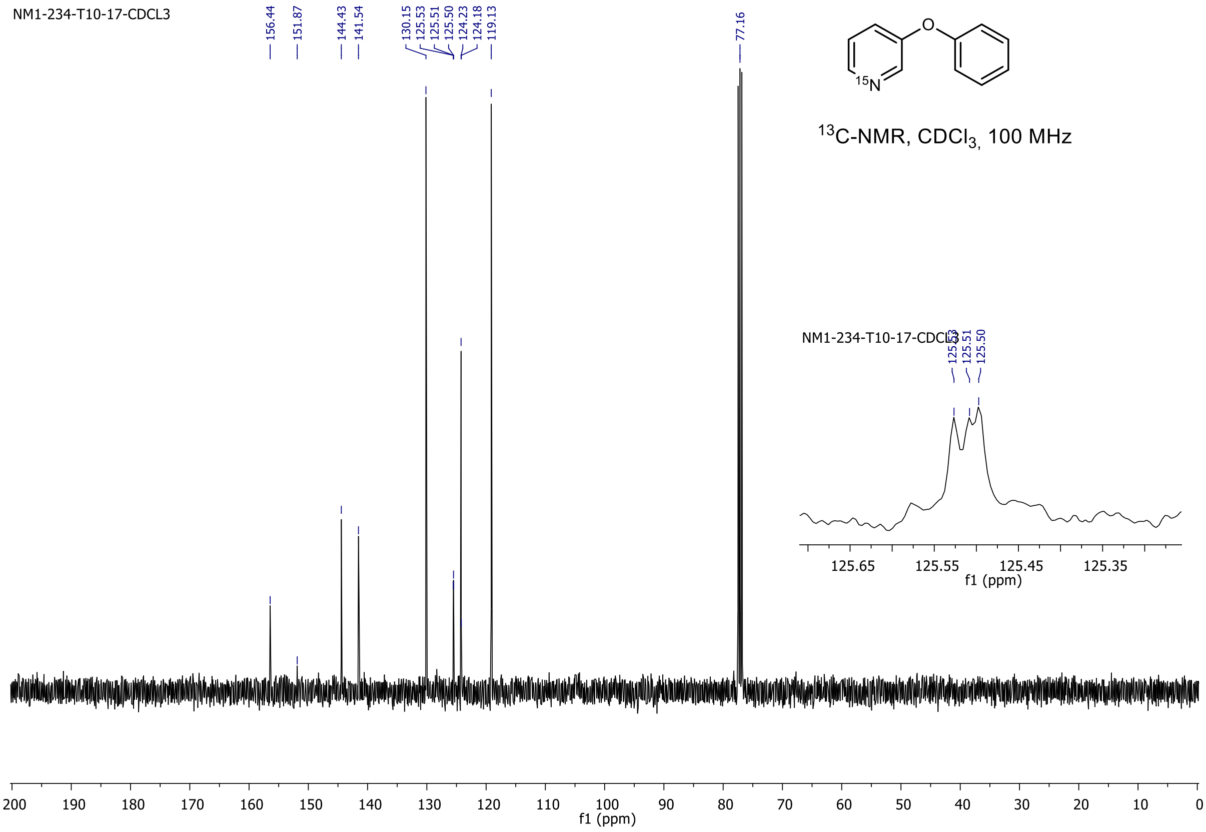

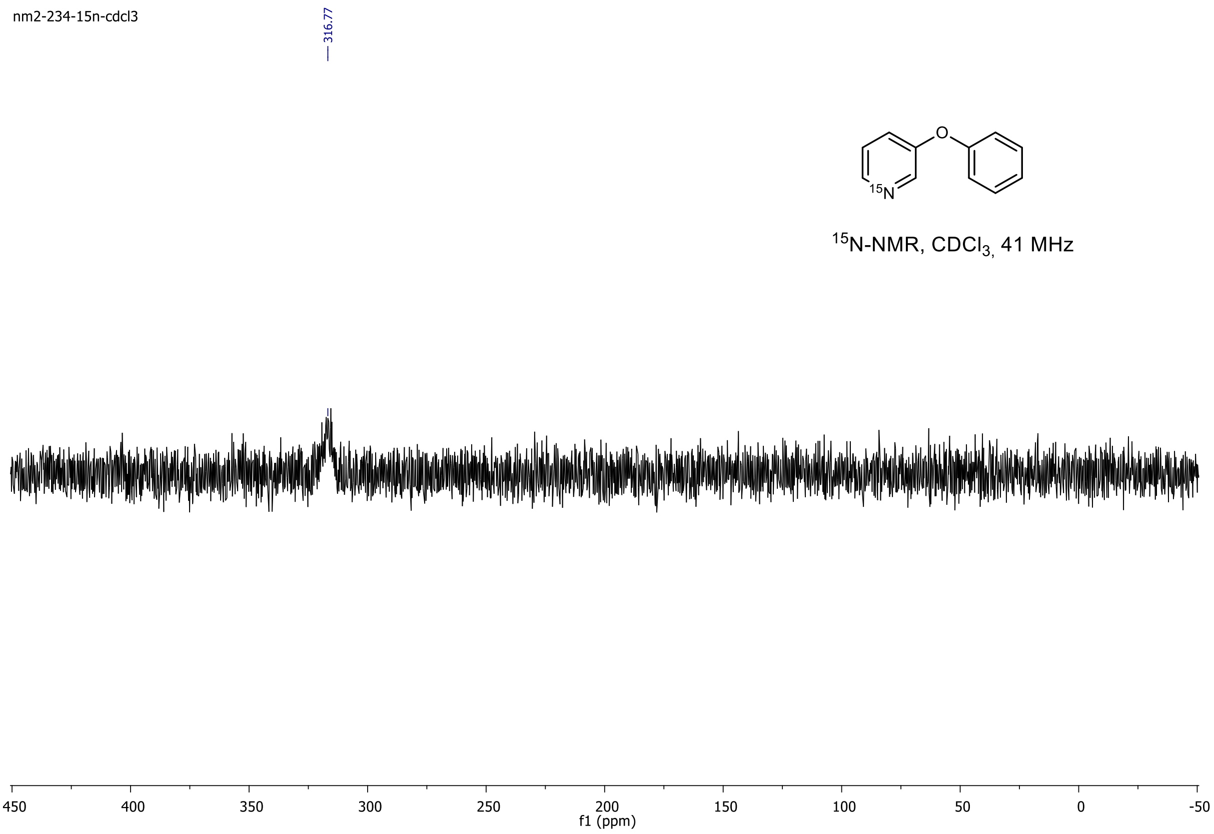

nm2-234-15n-cdcl3
316.77
^{15}N
O
^{15}N-NMR, $CDCl_3$, 41 MHz
450
400
350
300
250
200
150
100
50
0
-50
f1 (ppm)

Isotopic Enrichment

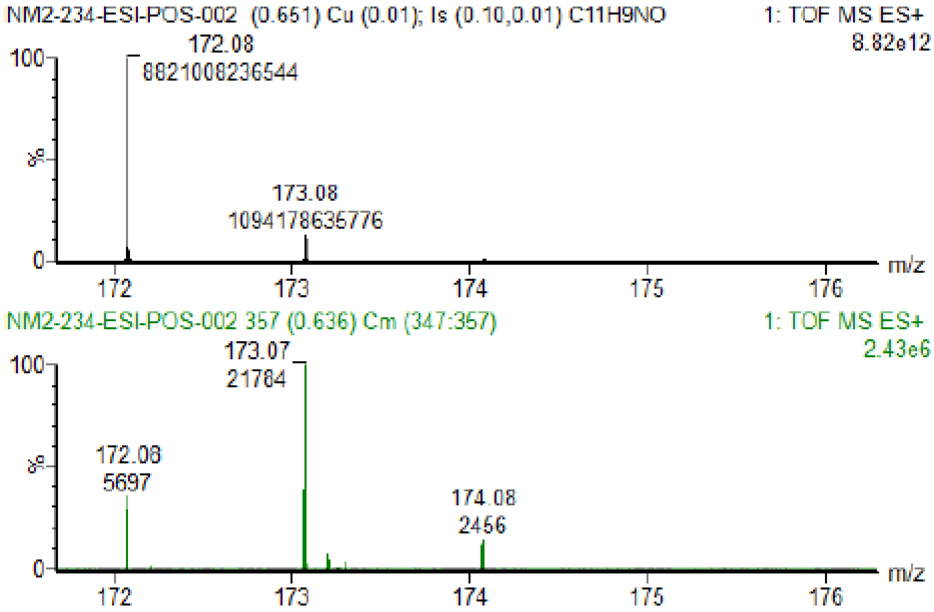

theorical isotopic distribution

	M	M+1	M+2	M+3	M+4	M+5	M+6	M+7
m/z	172	173	174	175	176	177	178	179
%	100,0	12,4	0,8	0,0	0,0	0,0	0,0	0,0

Enrichment calculation

Isotopomer	m/z	Area	natural isotope correction	Corrected area	Isotopic purity (%)
0	172	5697	0,00	5697,00	21,48
1	173	21784	706,43	21077,57	79,48
2	174	2456	45,58	-203,19	-0,77
3	175	82	0,00	-61,42	-0,23
4	176	1	0,00	10,24	0,04
5	177	0	0,00	-0,78	0,00
6	178	0	0,00	0,01	0,00
7	179	0	0,00	0,00	0,00
8	180	0	0,00	0,00	0,00
9	181	0	0,00	0,00	0,00
10	182	0	0,00	0,00	0,00
11	183	0	0,00	0,00	0,00
Total		30020		26519,43	100,00

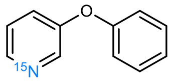

% Isotopic enrichment : 79,5

(E)-3-Styrylpyridine-1-^{15}N ([^{15}N]19)

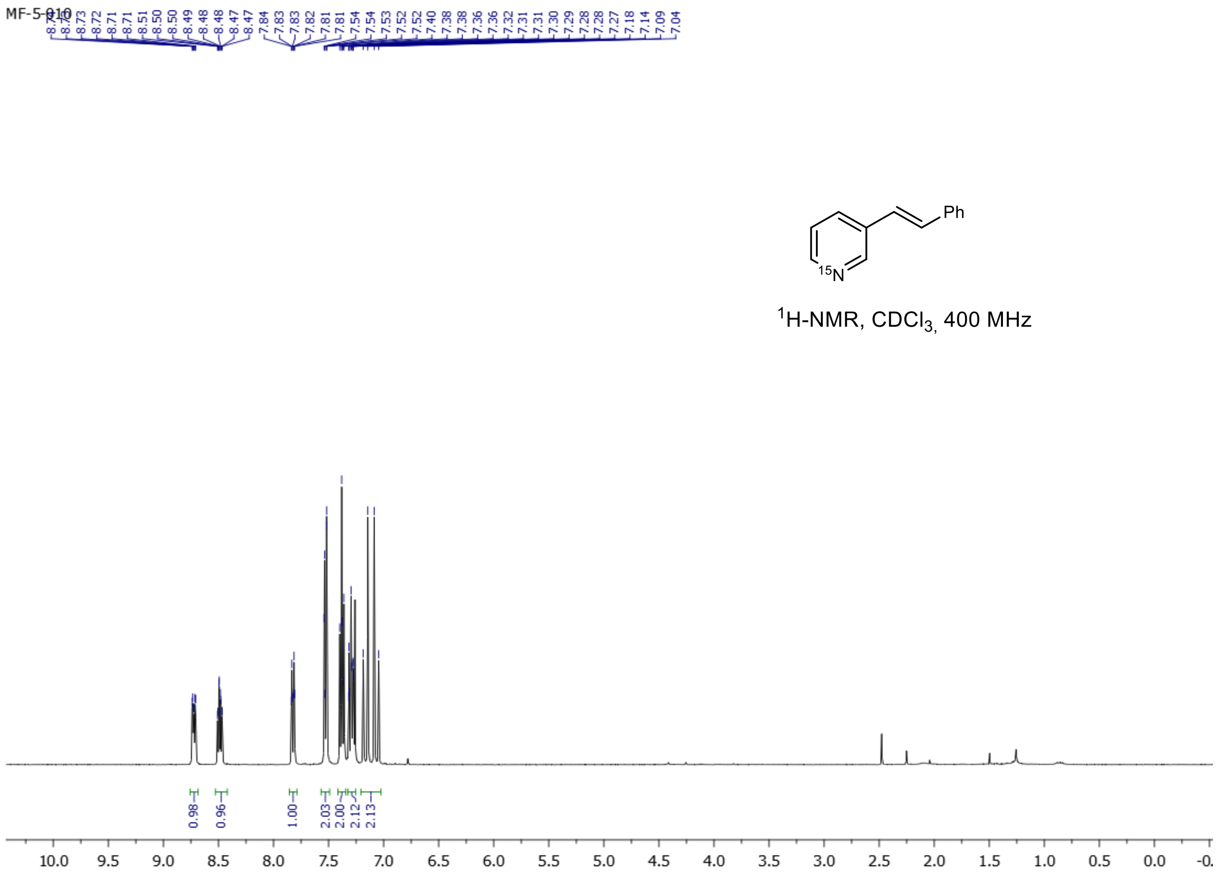

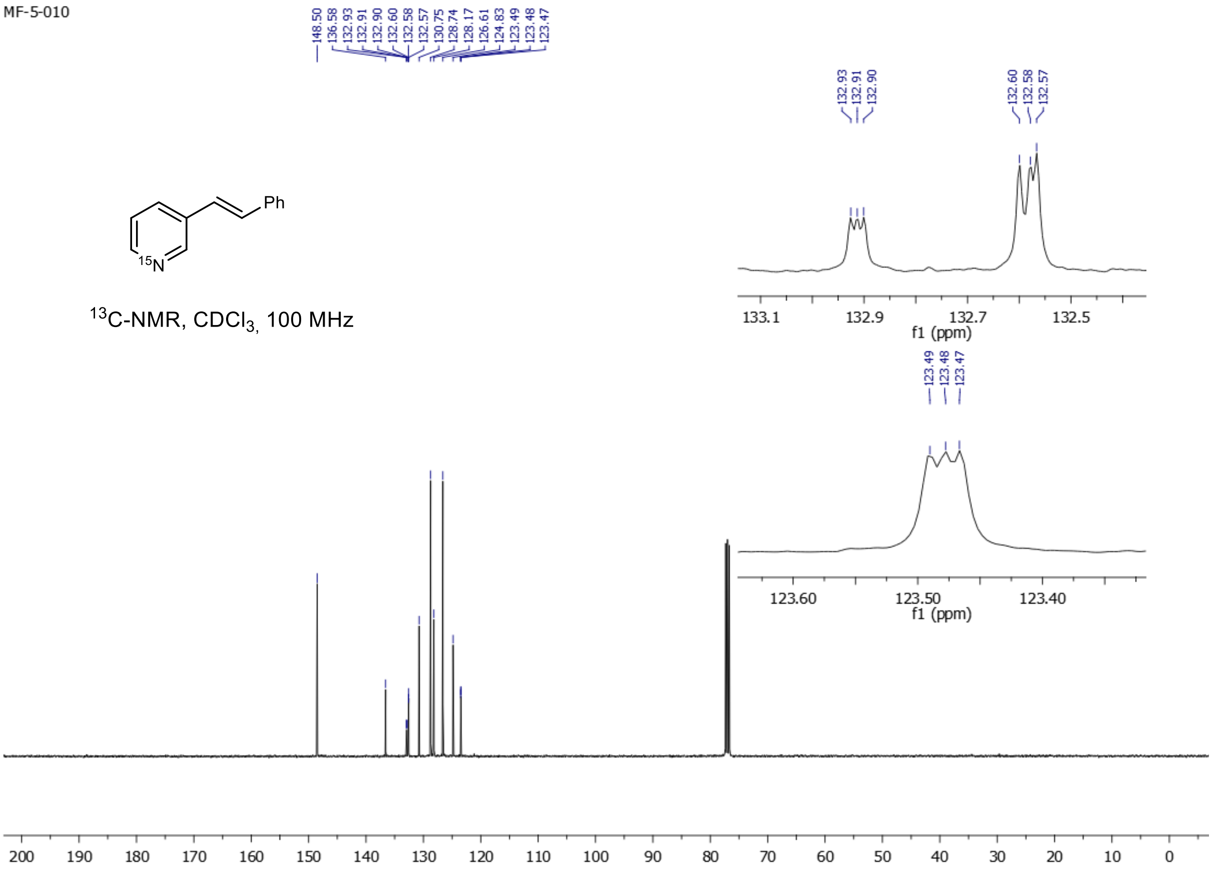

MF-5-010

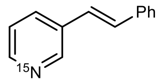

^{15}N-NMR, $CDCl_3$, 41 MHz

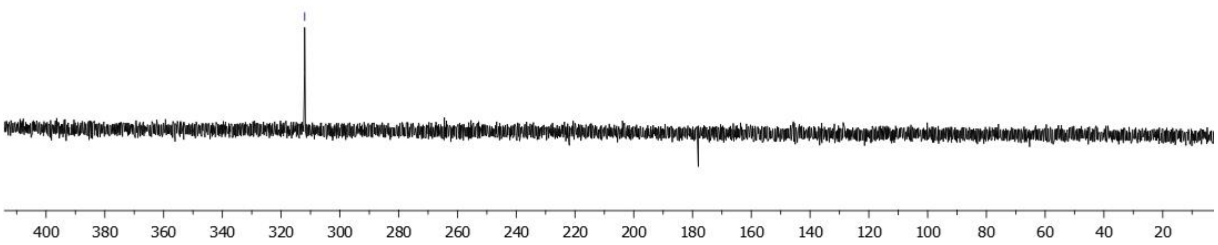

Isotopic Enrichment

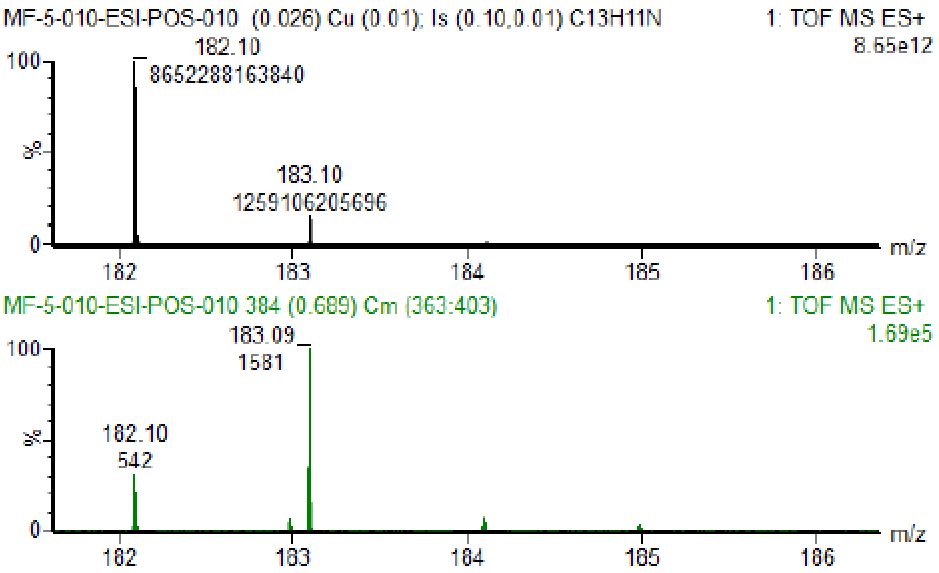

theorical isotopic distribution

	M	M+1	M+2	M+3	M+4	M+5	M+6	M+7
**m/z**	182	183	184	185	186	187	188	189
**%**	100,0	14,6	1,0	0,0	0,0	0,0	0,0	0,0

Enrichment calculation

Isotopomer	m/z	Area	natural isotope correction	Corrected area	Isotopic purity (%)
0	182	542	0,00	542,00	27,54
1	183	1581	79,13	1501,87	76,31
2	184	137	5,42	-87,69	-4,46
3	185	15	0,00	12,78	0,65
4	186	0	0,00	-0,99	-0,05
5	187	0	0,00	0,02	0,00
6	188	0	0,00	0,01	0,00
7	189	0	0,00	0,00	0,00
8	190	0	0,00	0,00	0,00
9	191	0	0,00	0,00	0,00
10	192	0	0,00	0,00	0,00
11	193	0	0,00	0,00	0,00
Total		2275		1967,99	100,00

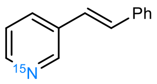

% Isotopic enrichment : 76,3

3-(Phenylethynyl)pyridine-1-^{15}N ([^{15}N]20)

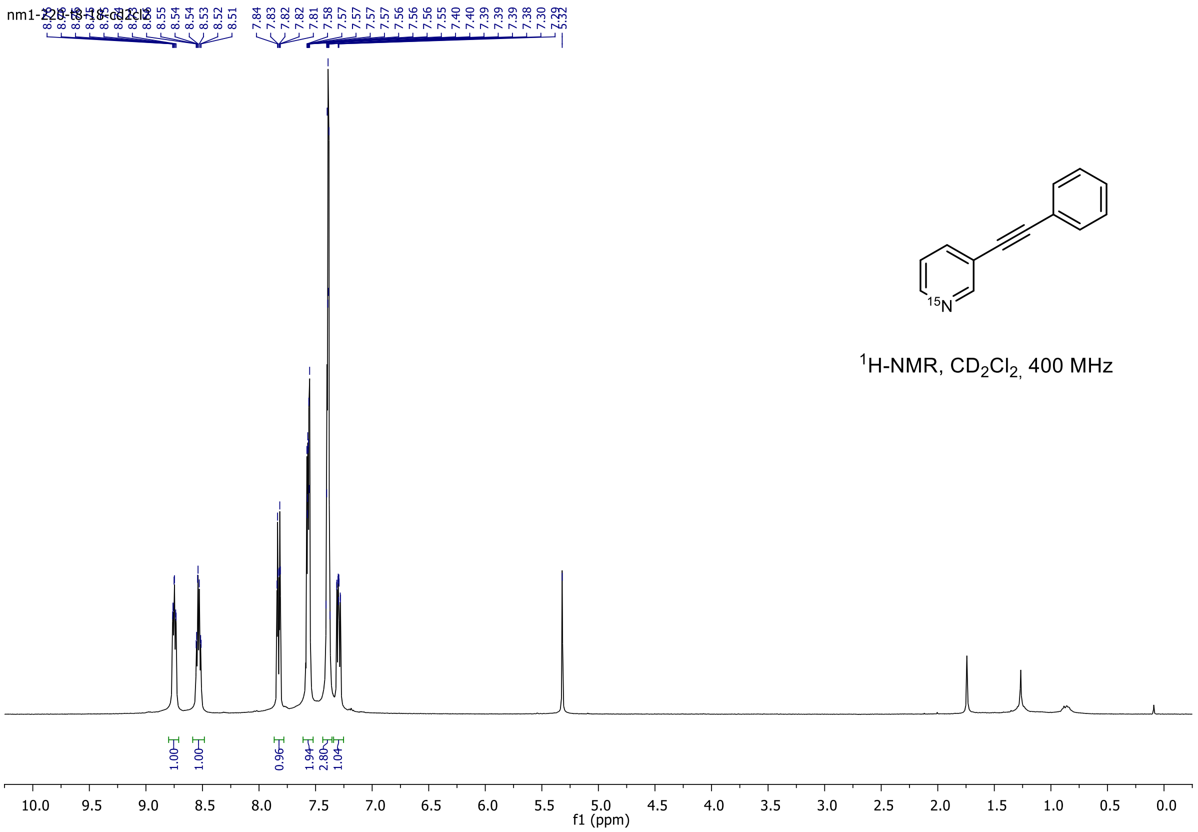

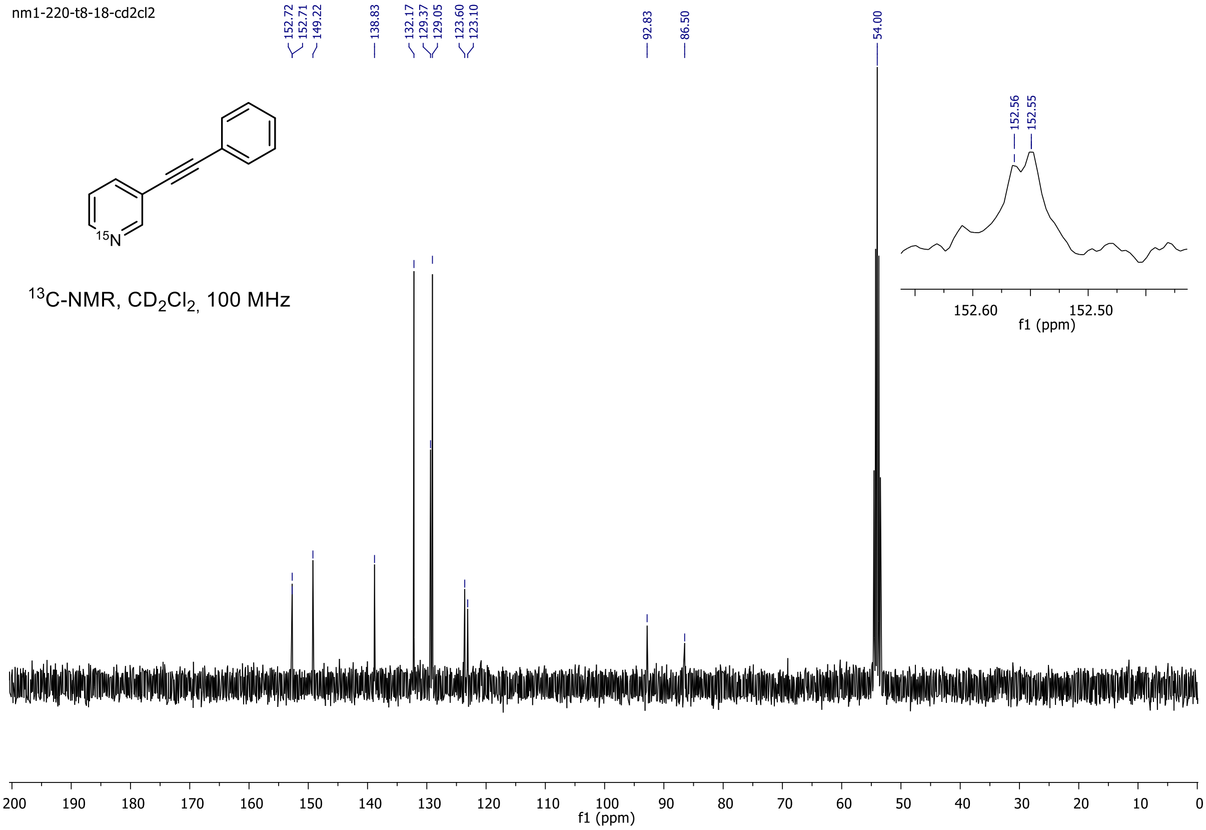

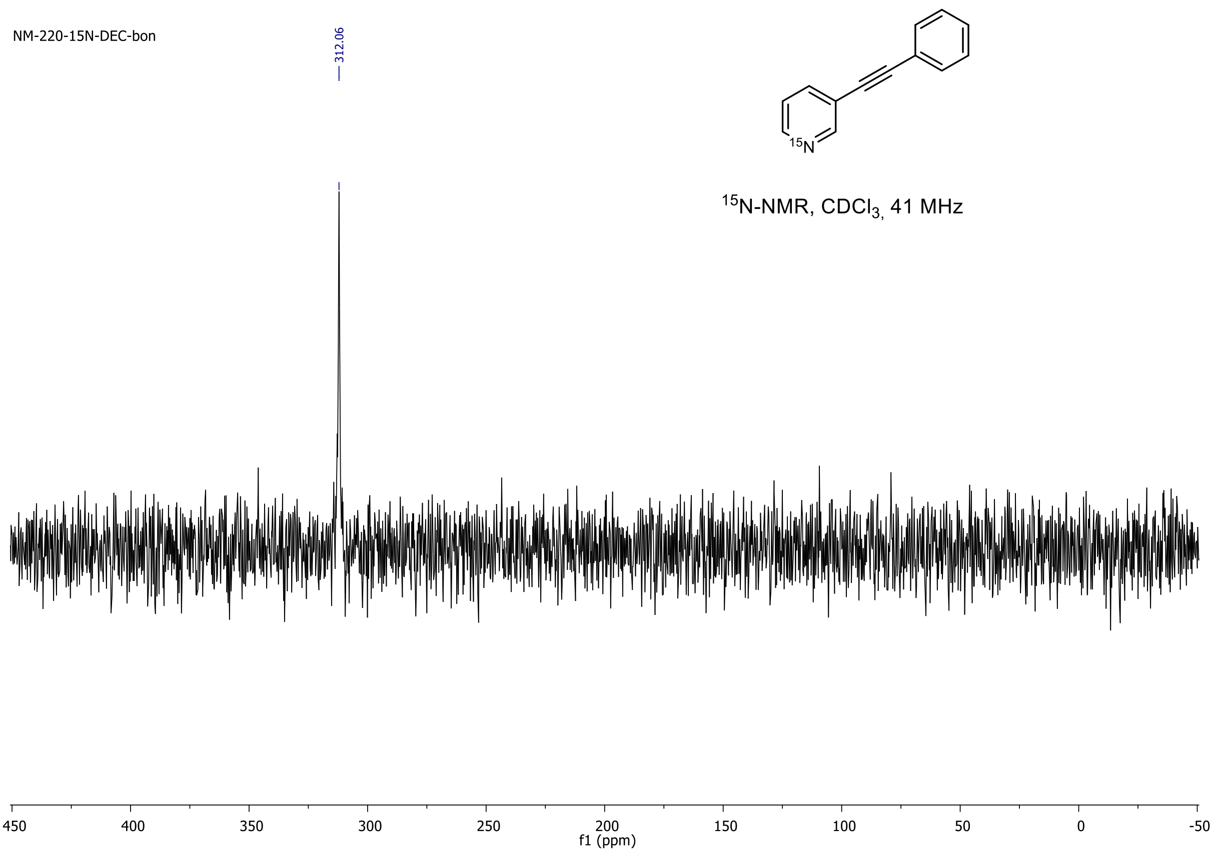
NM-220-15N-DEC-bon
312.06
15N
15N-NMR, CDCl3, 41 MHz
450 400 350 300 250 200 150 100 50 0 -50
f1 (ppm)

Isotopic Enrichment

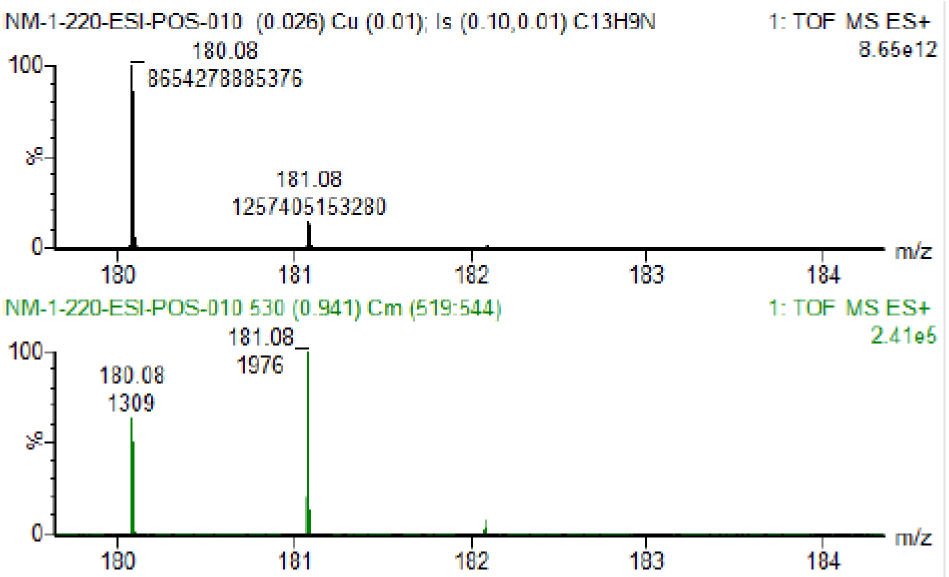

theorical isotopic distribution

	M	M+1	M+2	M+3	M+4	M+5	M+6	M+7
m/z	180	181	182	183	184	185	186	187
%	100,0	14,6	1,0	0,0	0,0	0,0	0,0	0,0

Enrichment calculation

Isotopomer	m/z	Area	natural isotope correction	Corrected area	Isotopic purity (%)
0	180	1309	0,00	1309,00	43,38
1	181	1976	191,11	1784,89	59,16
2	182	197	13,09	-76,68	-2,54
3	183	6	0,00	-0,65	-0,02
4	184	0	0,00	0,86	0,03
5	185	0	0,00	-0,12	0,00
6	186	0	0,00	0,01	0,00
7	187	0	0,00	0,00	0,00
8	188	0	0,00	0,00	0,00
9	189	0	0,00	0,00	0,00
10	190	0	0,00	0,00	0,00
11	191	0	0,00	0,00	0,00
Total		3488		3017,30	100,00

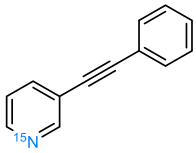

% Isotopic enrichment : 59,2

1-Methyl-5-(pyridin-3-yl -1-^{15}N)-1H-indole ([^{15}N]21)

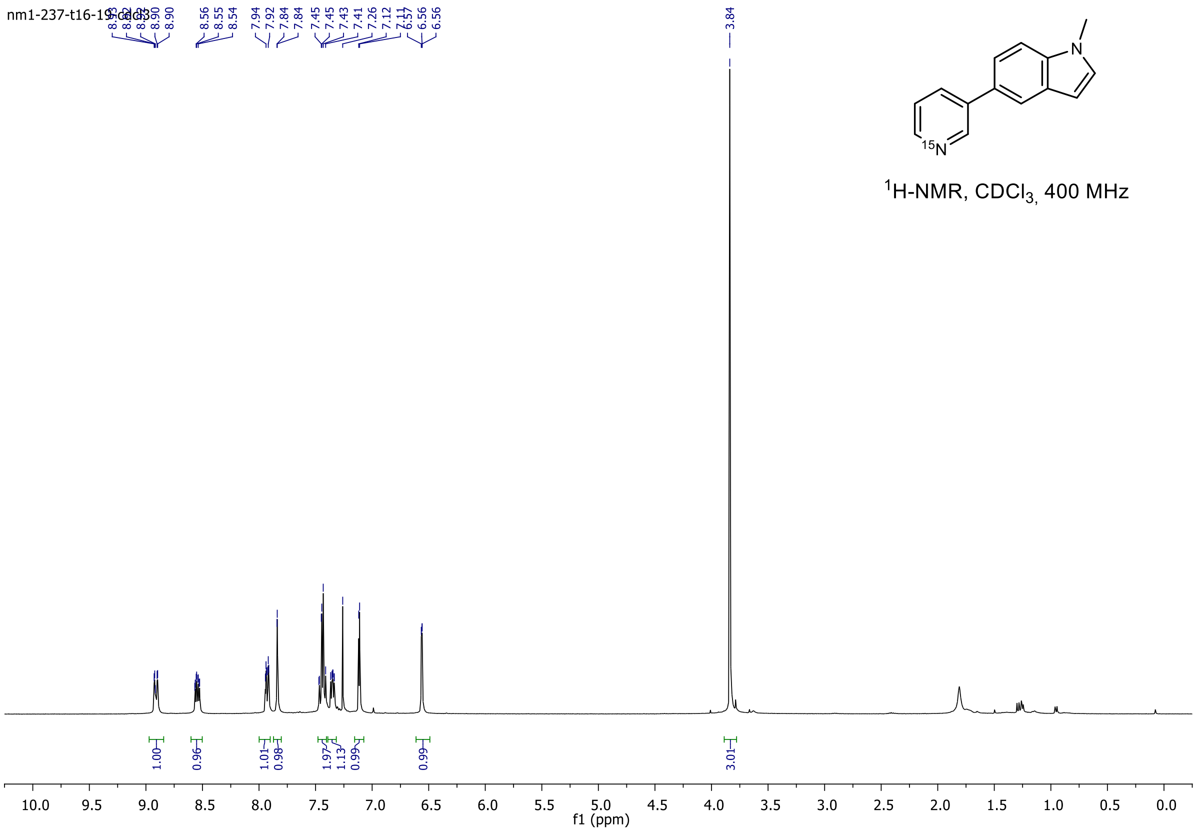

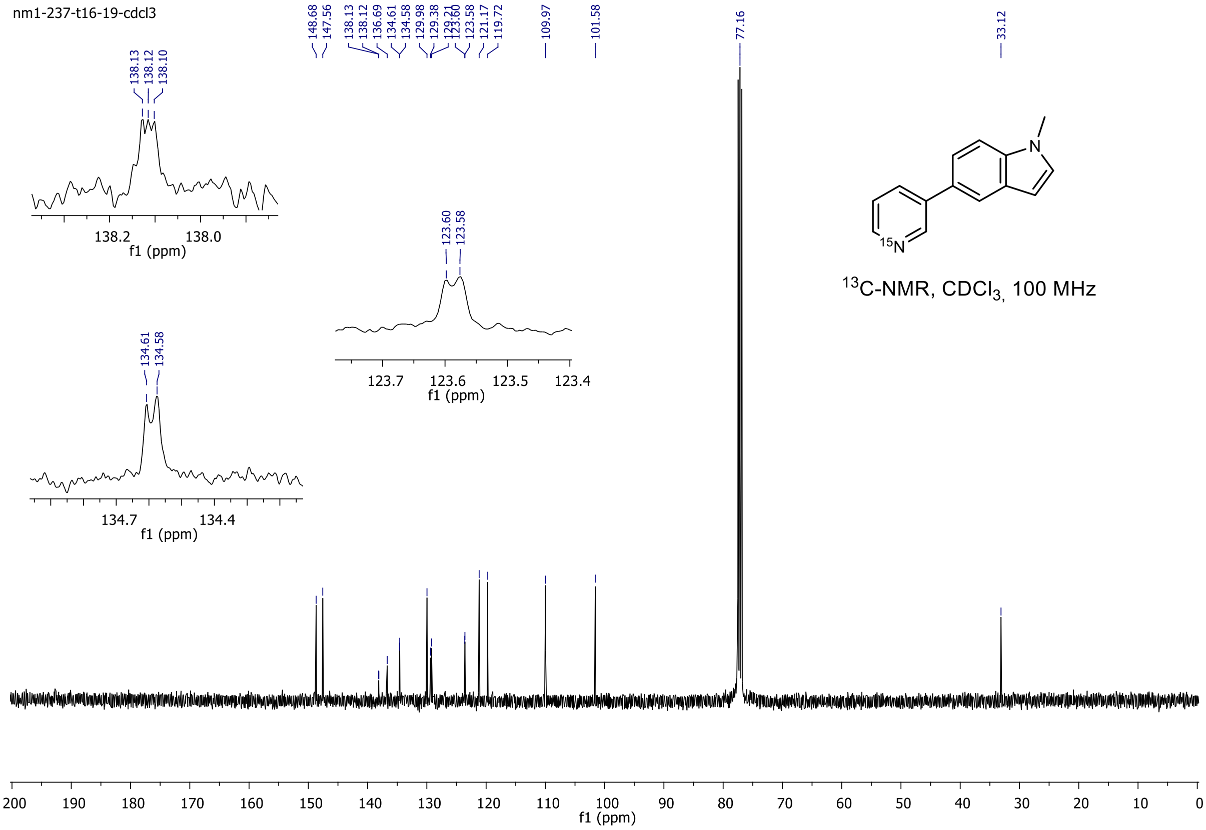

nm1-237-t16-19-cdcl3

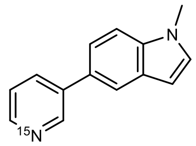

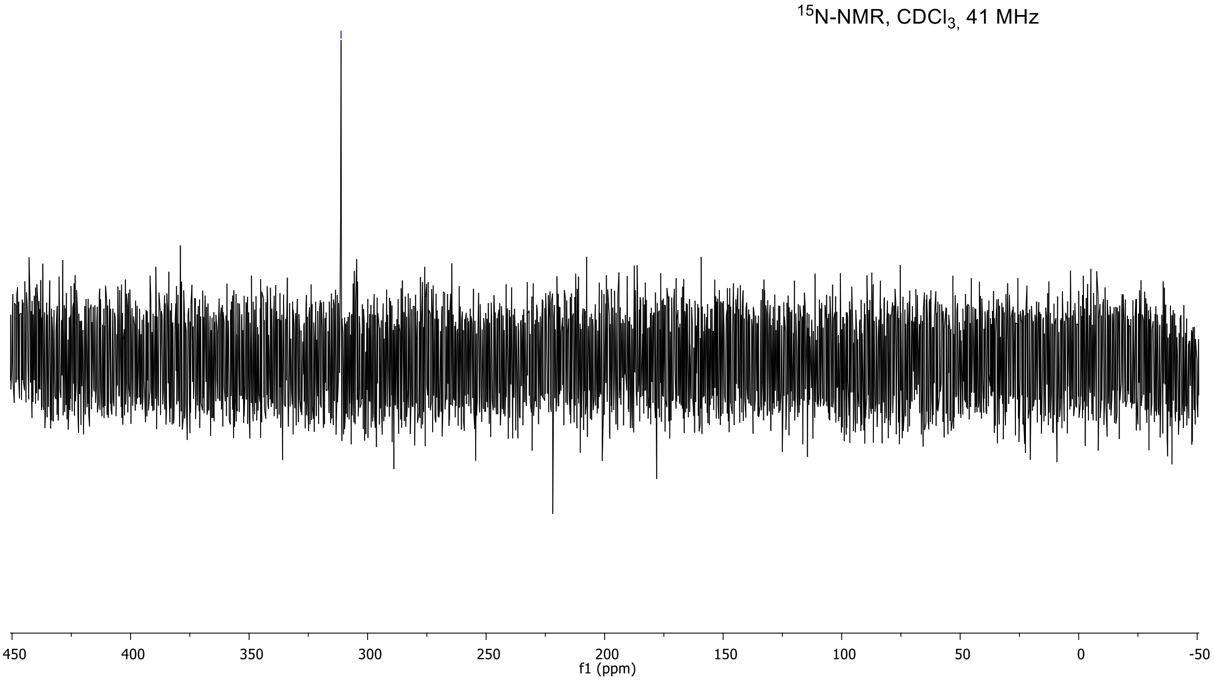

^{15}N-NMR, $CDCl_3$, 41 MHz

Isotopic Enrichment

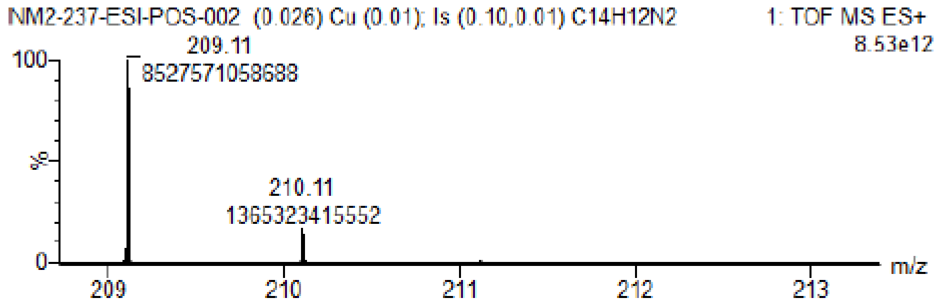

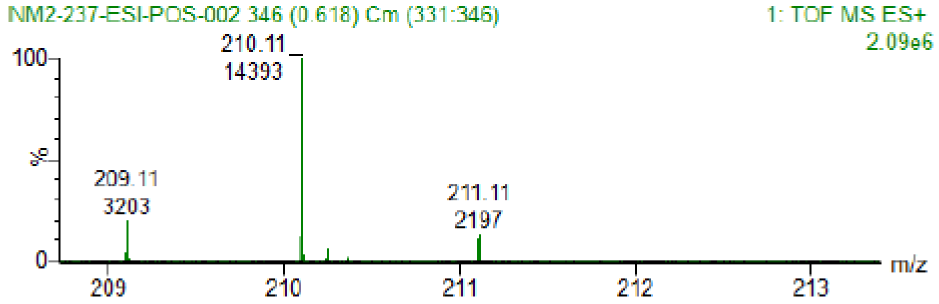

theorical isotopic distribution

	M	M+1	M+2	M+3	M+4	M+5	M+6	M+7
m/z	209	210	211	212	213	214	215	216
%	100,0	16,1	1,2	0,0	0,0	0,0	0,0	0,0

Enrichment calculation

Isotopomer	m/z	Area	natural isotope correction	Corrected area	Isotopic purity (%)
0	209	3203	0,00	3203,00	18,87
1	210	14393	515,68	13877,32	81,75
2	211	2197	38,44	-75,68	-0,45
3	212	111	0,00	-43,34	-0,26
4	213	9	0,00	16,89	0,10
5	214	0	0,00	-2,20	-0,01
6	215	0	0,00	0,15	0,00
7	216	0	0,00	0,00	0,00
8	217	0	0,00	0,00	0,00
9	218	0	0,00	0,00	0,00
10	219	0	0,00	0,00	0,00
11	220	0	0,00	0,00	0,00
Total		19913		16976,13	100,00

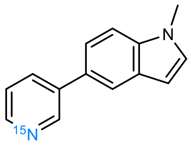

% Isotopic enrichment : 81,7

2-(Pyridin-3-yl-^{15}N)isoindoline-1,3-dione ([^{15}N]23)

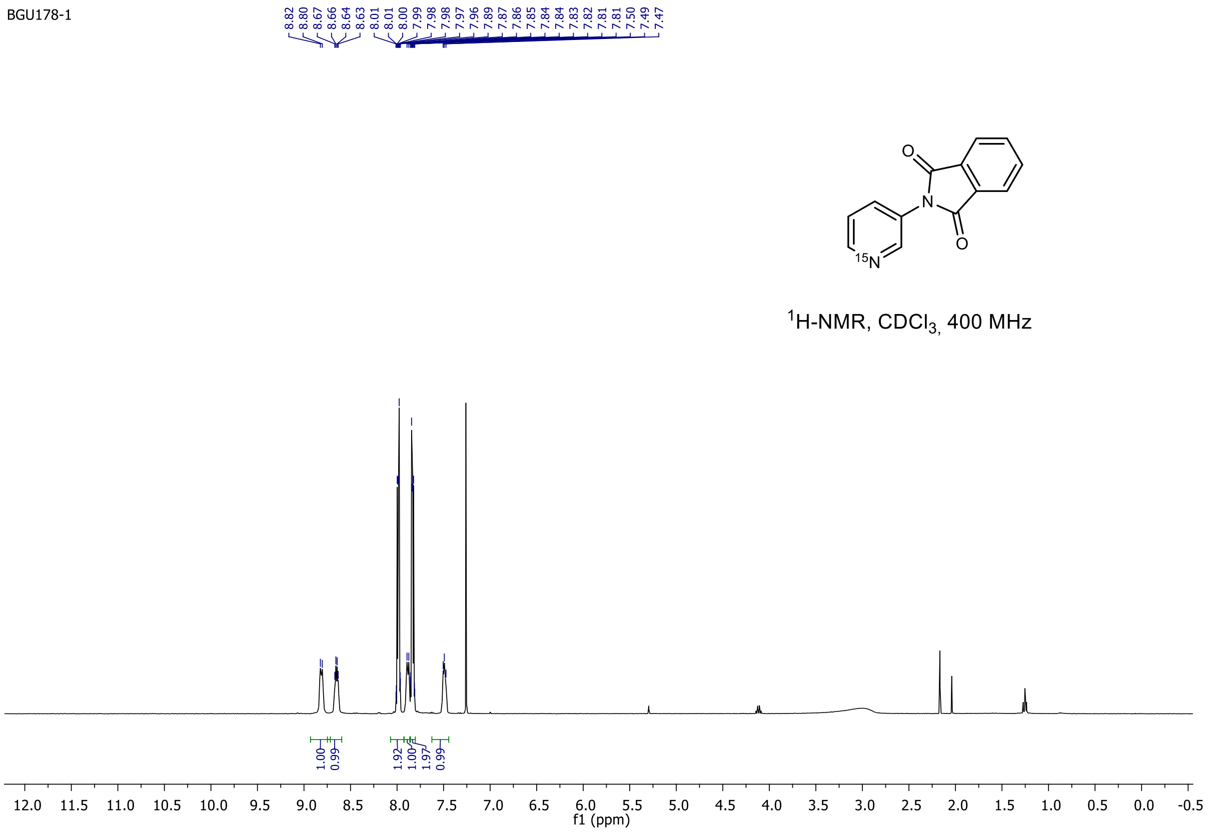

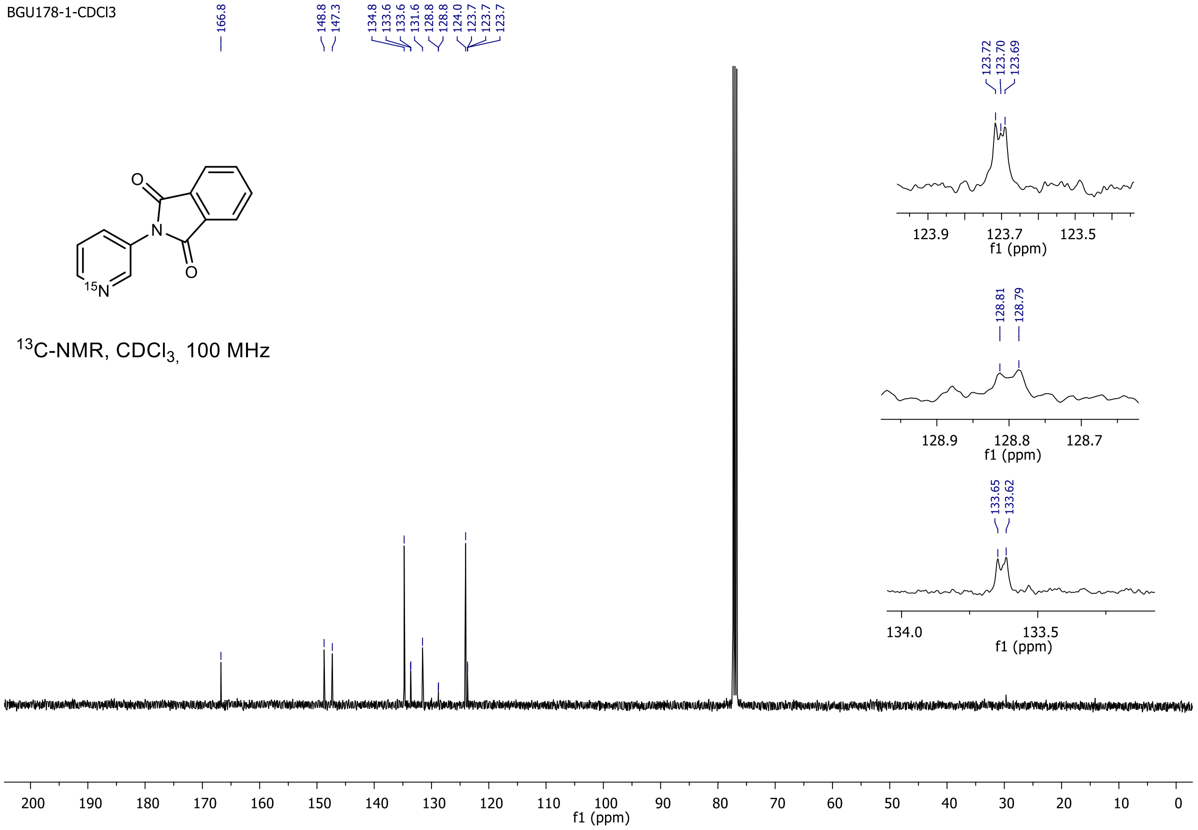

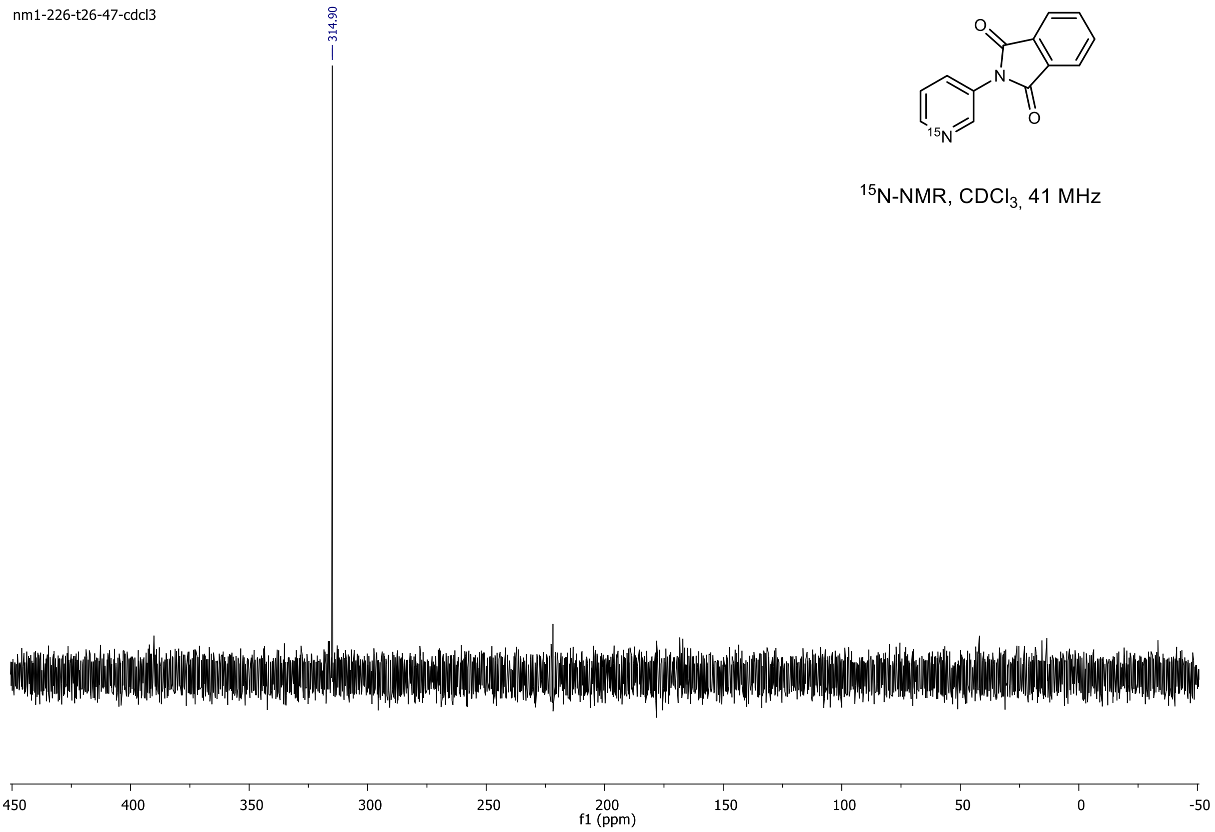

^{15}N-NMR, $CDCl_3$, 41 MHz

Isotopic Enrichment

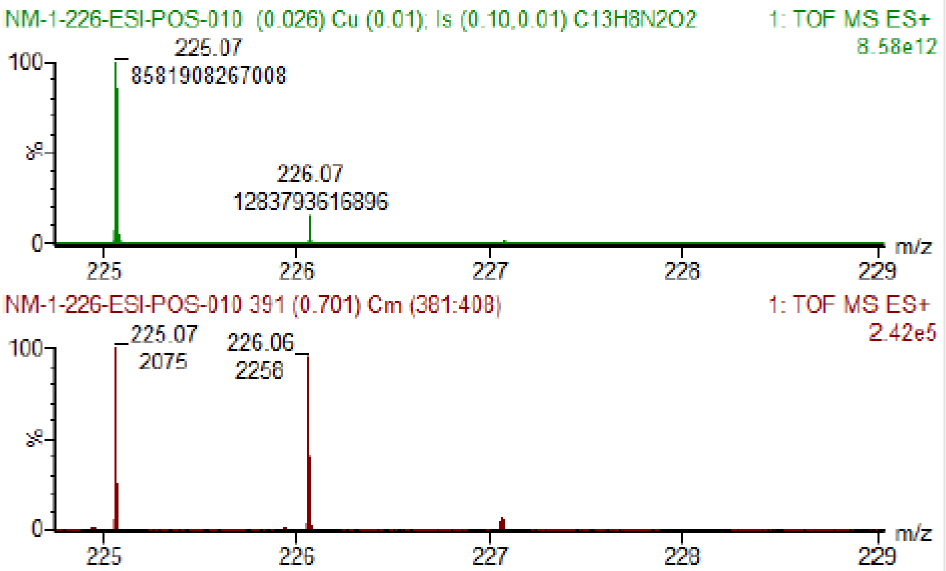

theorical isotopic distribution

	M	M+1	M+2	M+3	M+4	M+5	M+6	M+7
**m/z**	225	226	227	228	229	230	231	232
**%**	100,0	15,0	1,4	0,1	0,0	0,0	0,0	0,0

Enrichment calculation

Isotopomer	m/z	Area	natural isotope correction	Corrected area	Isotopic purity (%)
0	225	2075	0,00	2075,00	52,80
1	226	2258	311,25	1946,75	49,54
2	227	236	29,05	-85,06	-2,16
3	228	9	2,08	-7,57	-0,19
4	229	0	0,00	0,38	0,01
5	230	0	0,00	0,13	0,00
6	231	0	0,00	-0,02	0,00
7	232	0	0,00	0,00	0,00
8	233	0	0,00	0,00	0,00
9	234	0	0,00	0,00	0,00
10	235	0	0,00	0,00	0,00
11	236	0	0,00	0,00	0,00
Total		4578		3929,61	100,00

% Isotopic enrichment : 49,5

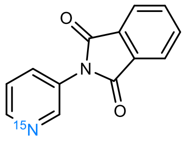

IE obtained following procedure A

Isotopic Enrichment

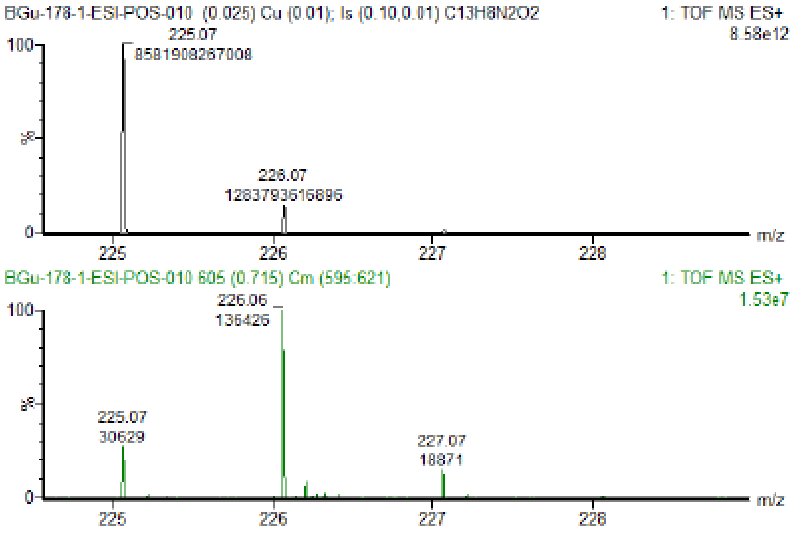

theorical isotopic distribution

	M	M+1	M+2	M+3	M+4	M+5	M+6	M+7
m/z	225	226	227	228	229	230	231	232
%	100,0	15,0	1,4	0,1	0,0	0,0	0,0	0,0

Enrichment calculation

Isotopomer	m/z	Area	natural isotope correction	Corrected area	Isotopic purity (%)
0	225	30629	0,00	30629,00	18,93
1	226	136426	4594,35	131831,65	81,49
2	227	18871	428,81	-1332,55	-0,82
3	228	2056	30,63	379,61	0,23
4	229	495	0,00	324,88	0,20
5	230	0	0,00	-52,71	-0,03
6	231	0	0,00	2,98	0,00
7	232	0	0,00	-0,03	0,00
8	233	0	0,00	0,02	0,00
9	234	0	0,00	0,00	0,00
10	235	0	0,00	0,00	0,00
11	236	0	0,00	0,00	0,00
Total		188477		161782,83	100,00

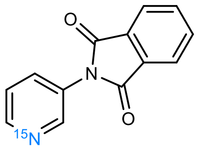

IE obtained following procedure E

% Isotopic enrichment : 81,5

9-(4-(pyridin-3-yl-^{15}N)phenyl)-9H-carbazole ([^{15}N]24)

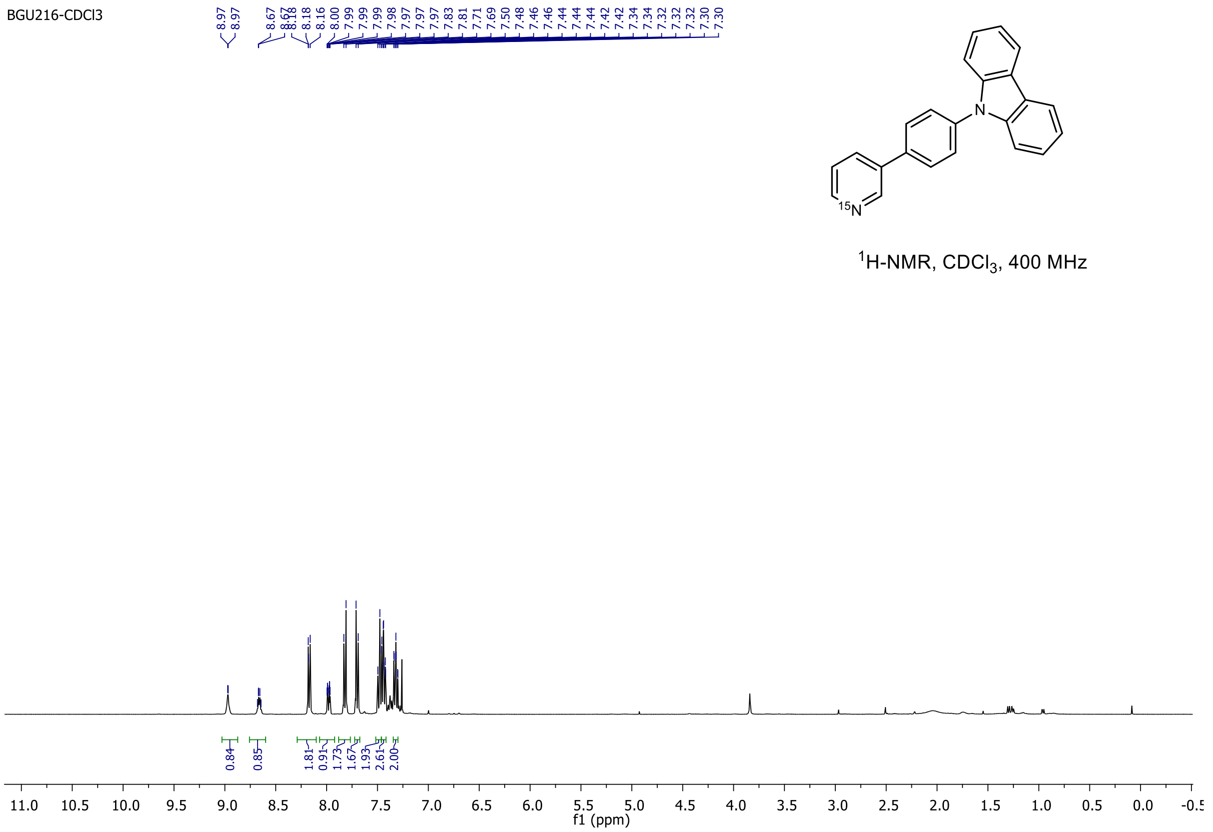

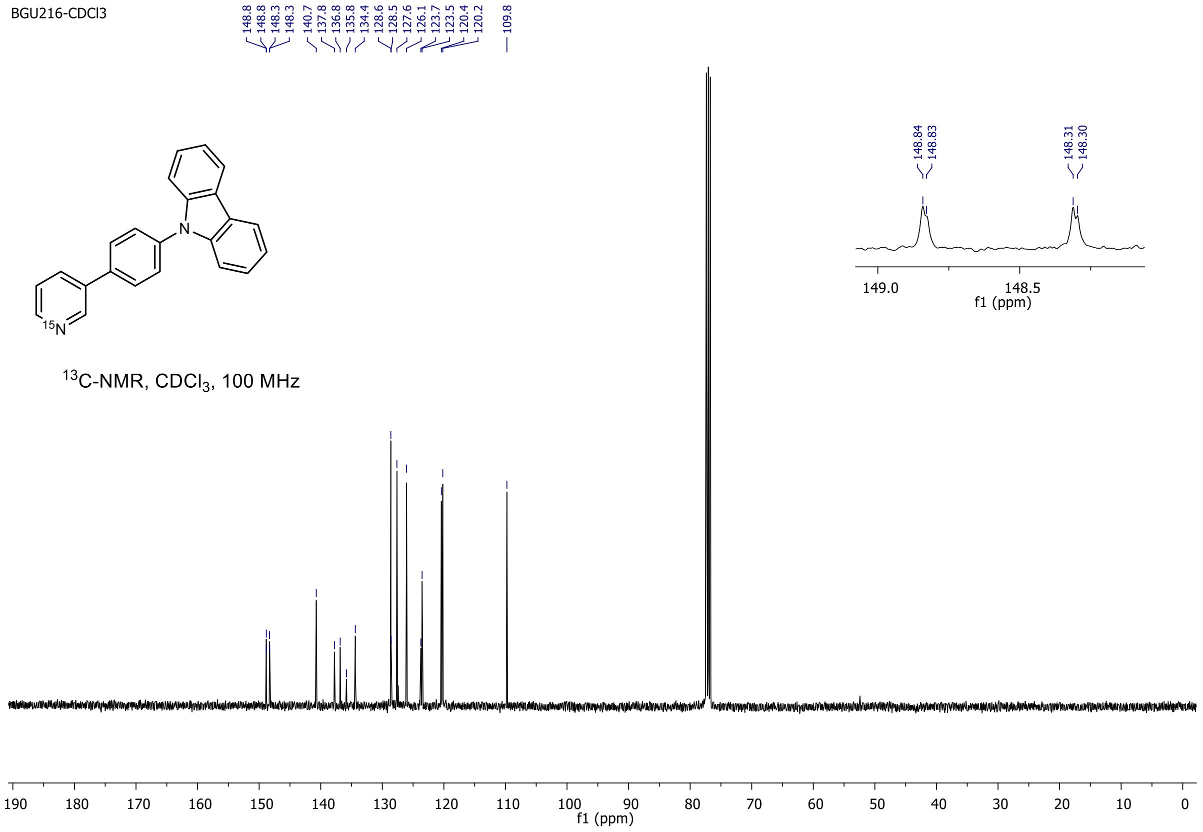

bgu_216_15N_dec

312.7

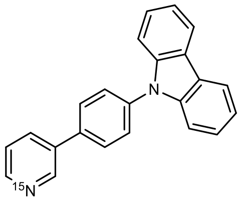

^{15}N-NMR, $CDCl_3$, 41 MHz

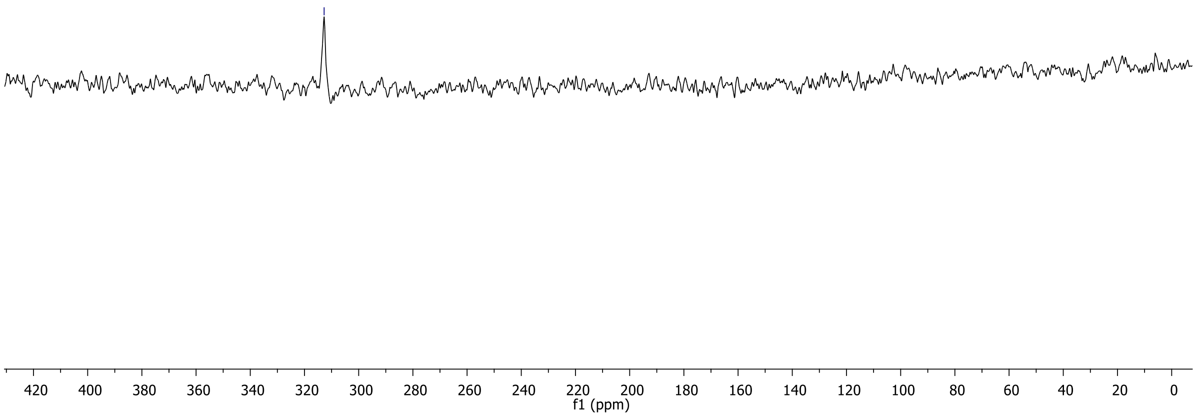

Isotopic Enrichment

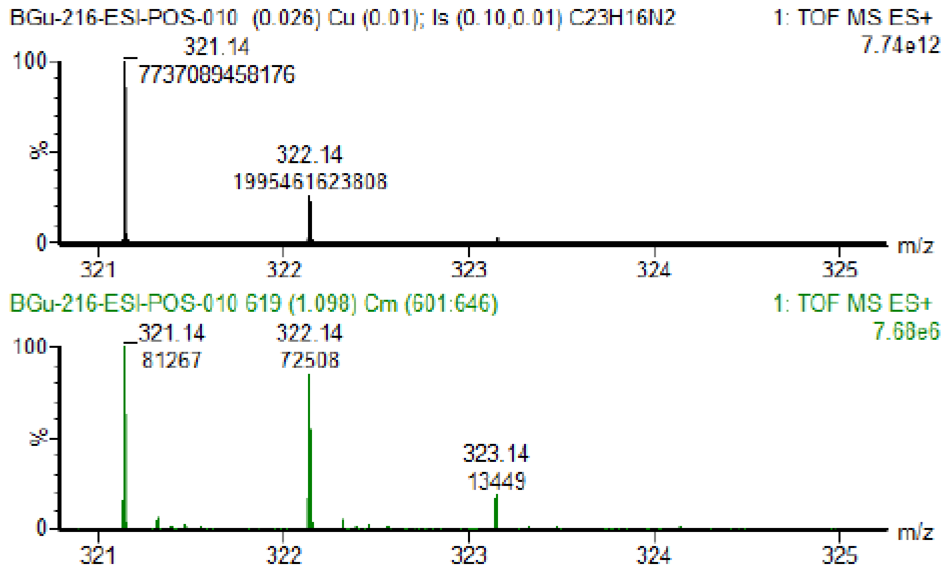

theorical isotopic distribution

	M	M+1	M+2	M+3	M+4	M+5	M+6	M+7
m/z	321	322	323	324	325	326	327	328
%	100,0	25,9	3,2	0,2	0,0	0,0	0,0	0,0

Enrichment calculation

Isotopomer	m/z	Area	natural isotope correction	Corrected area	Isotopic purity (%)
0	321	81267	0,00	81267,00	62,55
1	322	72508	21048,15	51459,85	39,61
2	323	13449	2600,54	-2479,64	-1,91
3	324	765	162,53	-402,02	-0,31
4	325	11	0,00	91,55	0,07
5	326	0	0,00	-5,89	0,00
6	327	0	0,00	-0,60	0,00
7	328	0	0,00	0,16	0,00
8	329	0	0,00	-0,01	0,00
9	330	0	0,00	0,00	0,00
10	331	0	0,00	0,00	0,00
11	332	0	0,00	0,00	0,00
Total		168000		129930,39	100,00

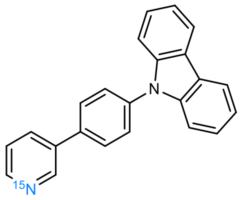

% Isotopic enrichment : 39,6

4-(3-(Pyridin-3-yl-^{15}N)phenyl)morpholine ([^{15}N]25)

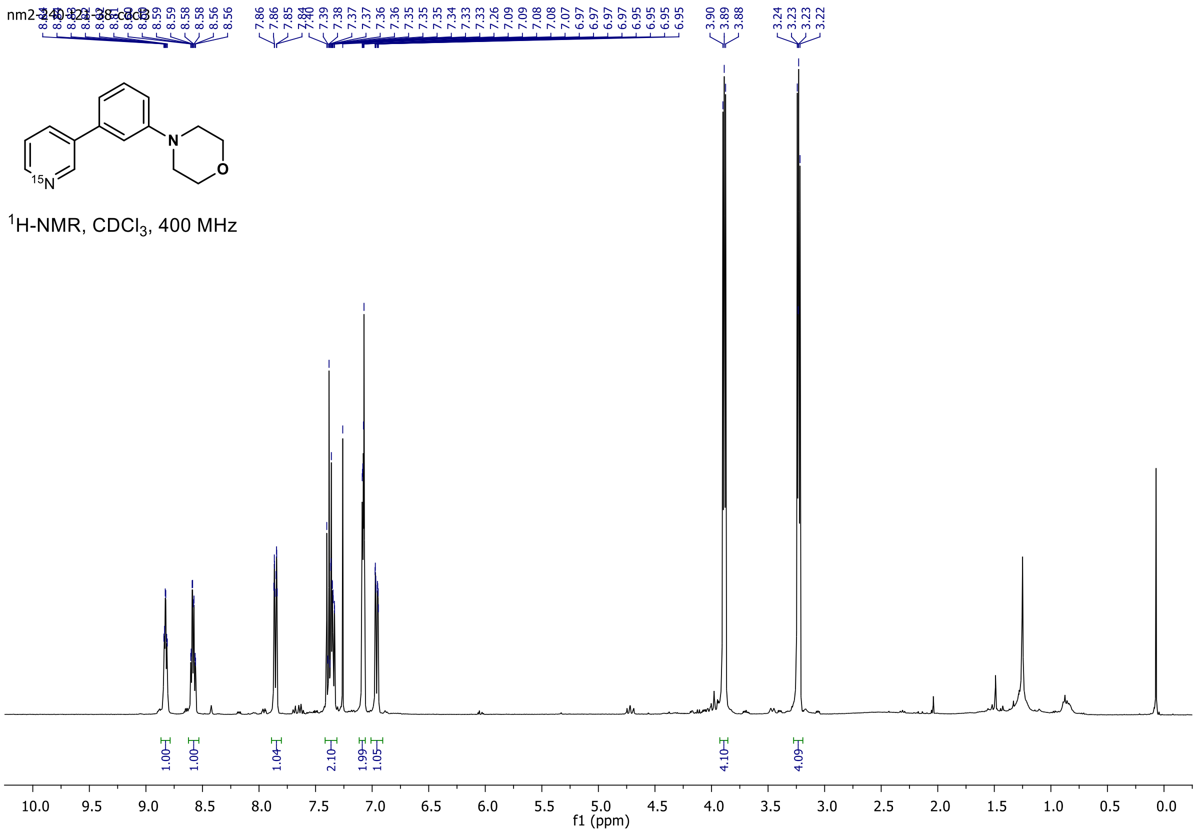

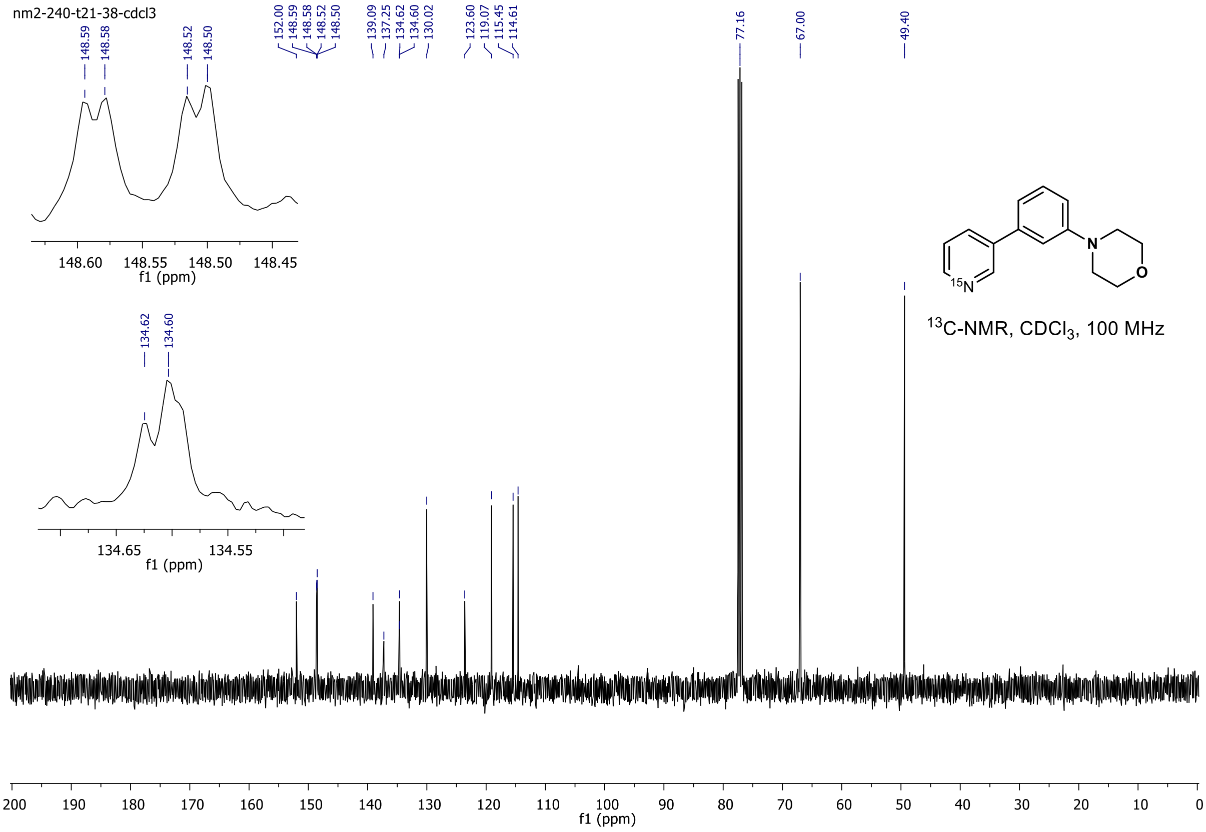

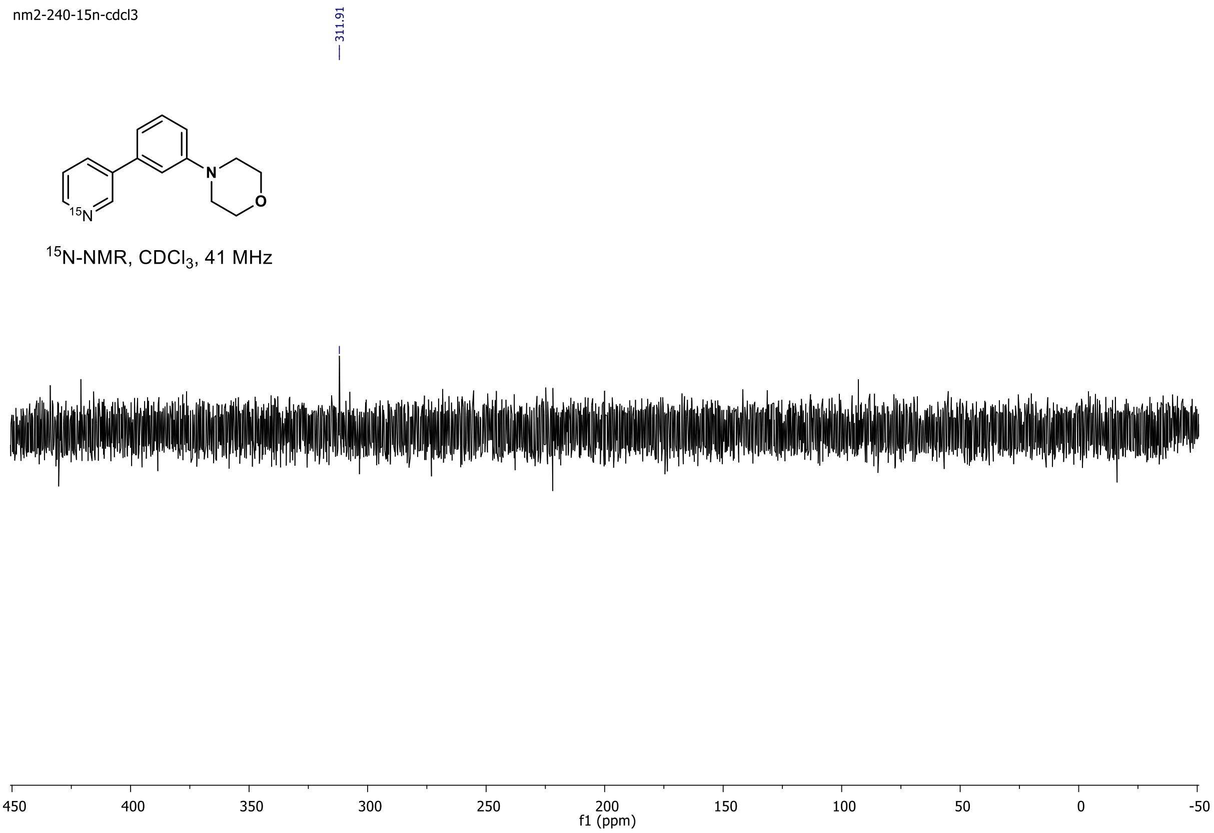
nm2-240-15n-cdcl3
311.91
15N
N
O
^{15}N-NMR, $CDCl_3$, 41 MHz
450
400
350
300
250
200
150
100
50
0
-50
f1 (ppm)

Isotopic Enrichment

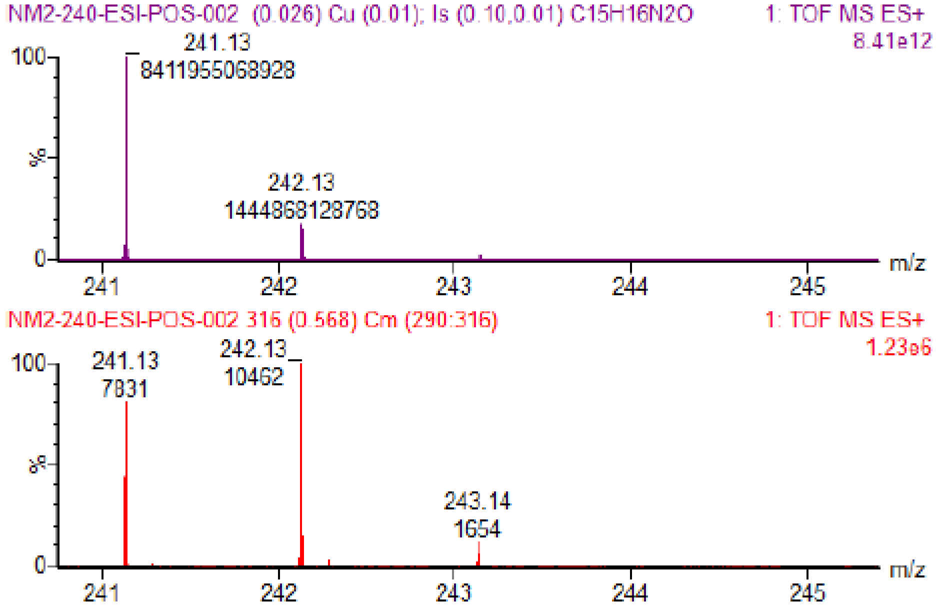

theorical isotopic distribution

	M	M+1	M+2	M+3	M+4	M+5	M+6	M+7
m/z	241	242	243	244	245	246	247	248
%	100,0	17,2	1,5	0,1	0,0	0,0	0,0	0,0

Enrichment calculation

Isotopomer	m/z	Area	natural isotope correction	Corrected area	Isotopic purity (%)
0	241	7831	0,00	7831,00	46,31
1	242	10462	1346,93	9115,07	53,91
2	243	1654	117,47	-31,26	-0,18
3	244	73	7,83	-66,18	-0,39
4	245	68	0,00	70,74	0,42
5	246	0	0,00	-11,14	-0,07
6	247	0	0,00	0,92	0,01
7	248	0	0,00	-0,06	0,00
8	249	0	0,00	0,01	0,00
9	250	0	0,00	0,00	0,00
10	251	0	0,00	0,00	0,00
11	252	0	0,00	0,00	0,00
Total		20088		16909,09	100,00

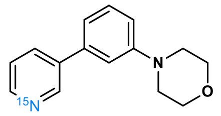

% Isotopic enrichment : 53,9

Methyl 5-bromonicotinate-^{15}N ([^{15}N]26)

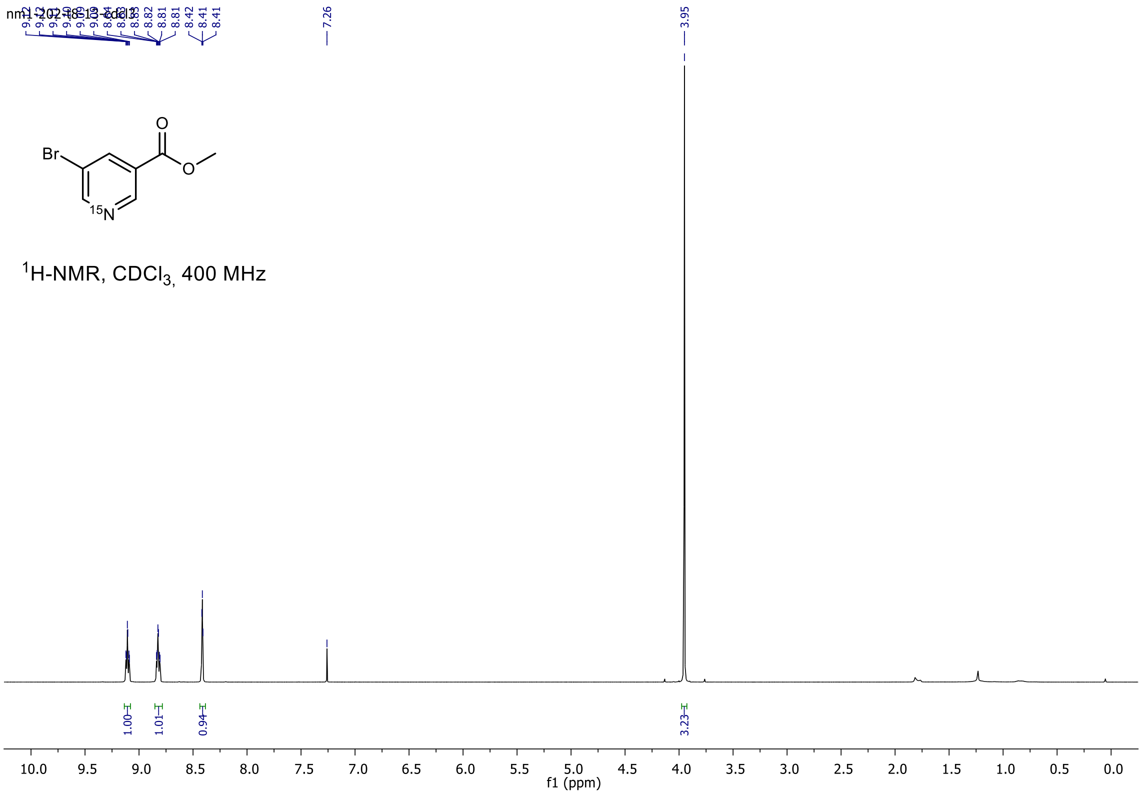

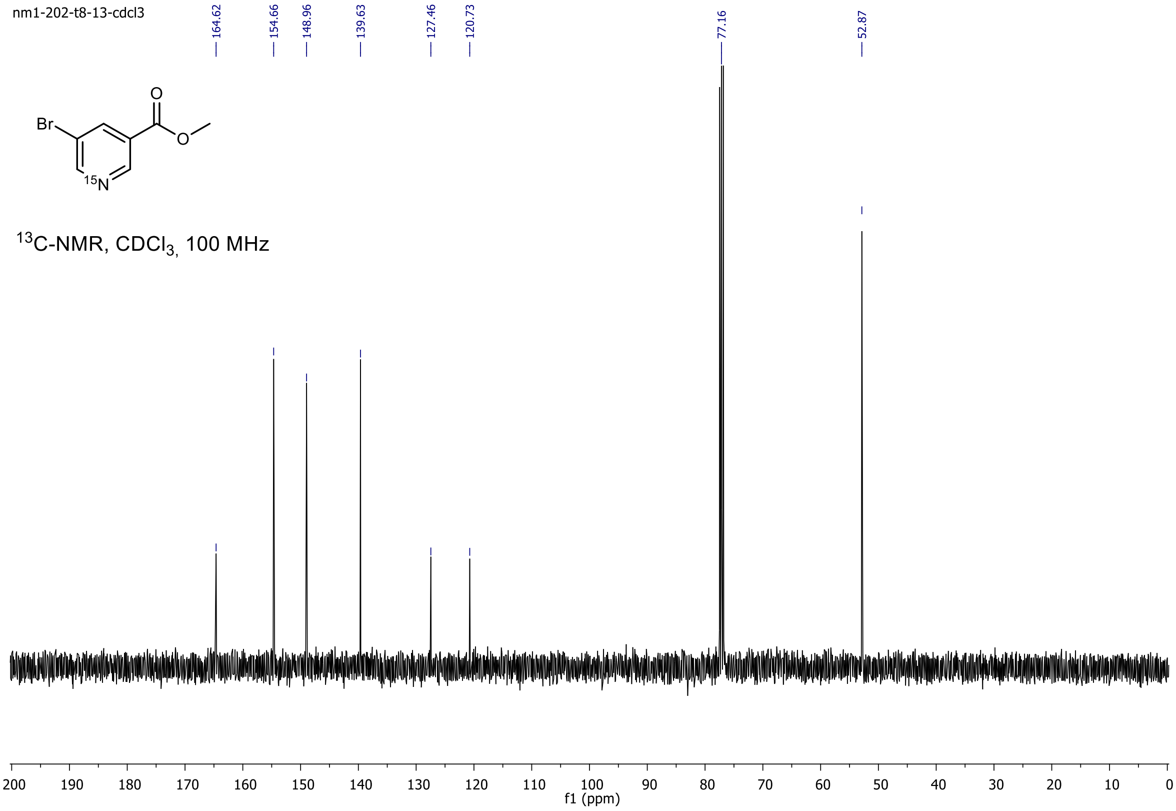

NM1-202-15N-CDCL3

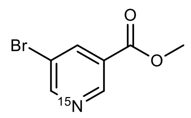

^{15}N-NMR, $CDCl_3$, 41 MHz

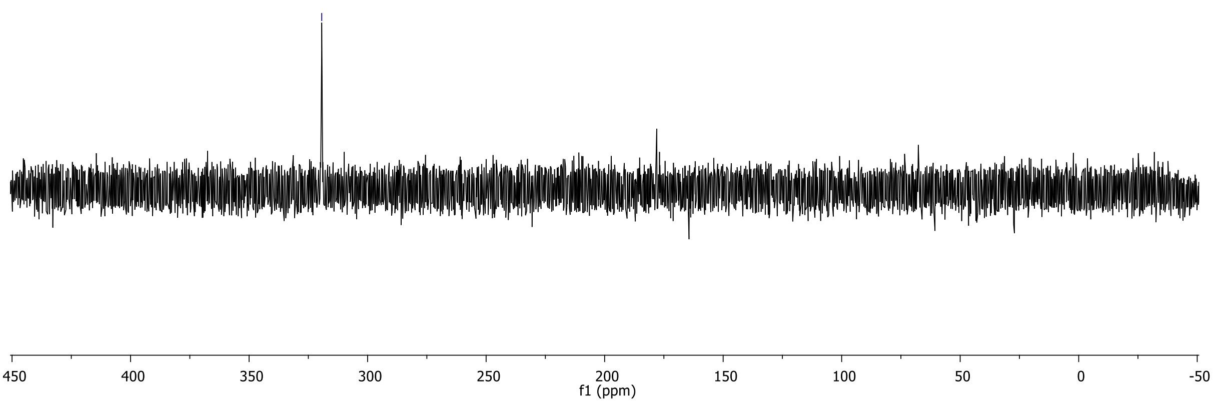

Isotopic Enrichment

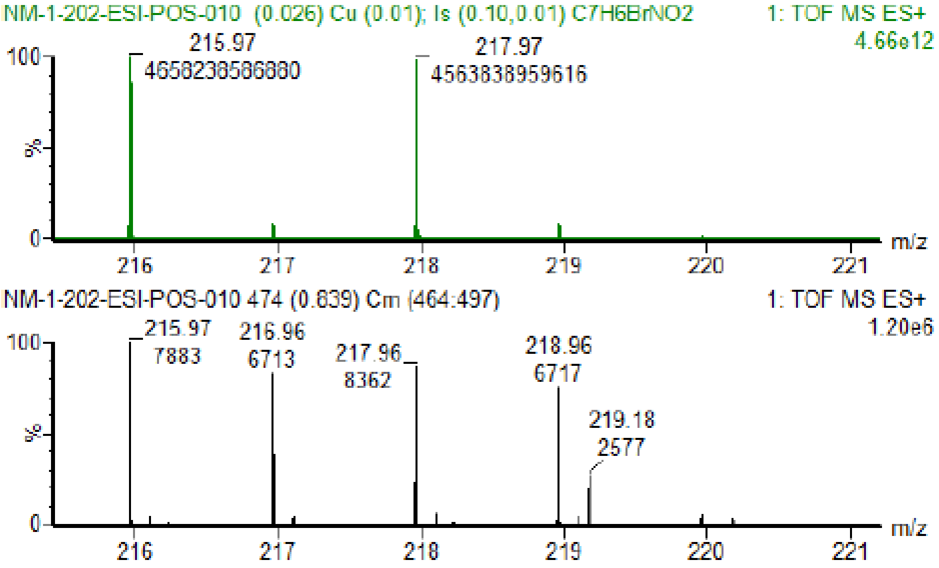

theorical isotopic distribution

	M	M+1	M+2	M+3	M+4	M+5	M+6	M+7
**m/z**	216	217	218	219	220	221	222	223
**%**	100,0	8,1	98,2	7,9	0,6	0,0	0,0	0,0

Enrichment calculation

Isotopomer	m/z	Area	natural isotope correction	Corrected area	Isotopic purity (%)
0	216	7883	0,00	7882,09	55,80
1	217	6713	638,48	6085,61	43,08
2	218	8362	7740,58	129,78	0,92
3	219	6717	622,71	96,40	0,68
4	220	519	47,29	-145,26	-1,03
5	221	18	0,00	-100,04	-0,71
6	222	0	0,00	143,32	1,01
7	223	0	0,00	85,63	0,61
8	224	0	0,00	-139,90	-0,99
9	225	0	0,00	-71,28	-0,50
10	226	0	0,00	136,55	0,97
11	227	0	0,00	56,97	0,40
Total		30212		14159,88	100,25

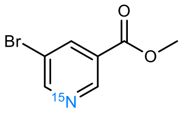

% Isotopic enrichment : 43,1

Isoquinoline-^{15}N ([^{15}N]27)

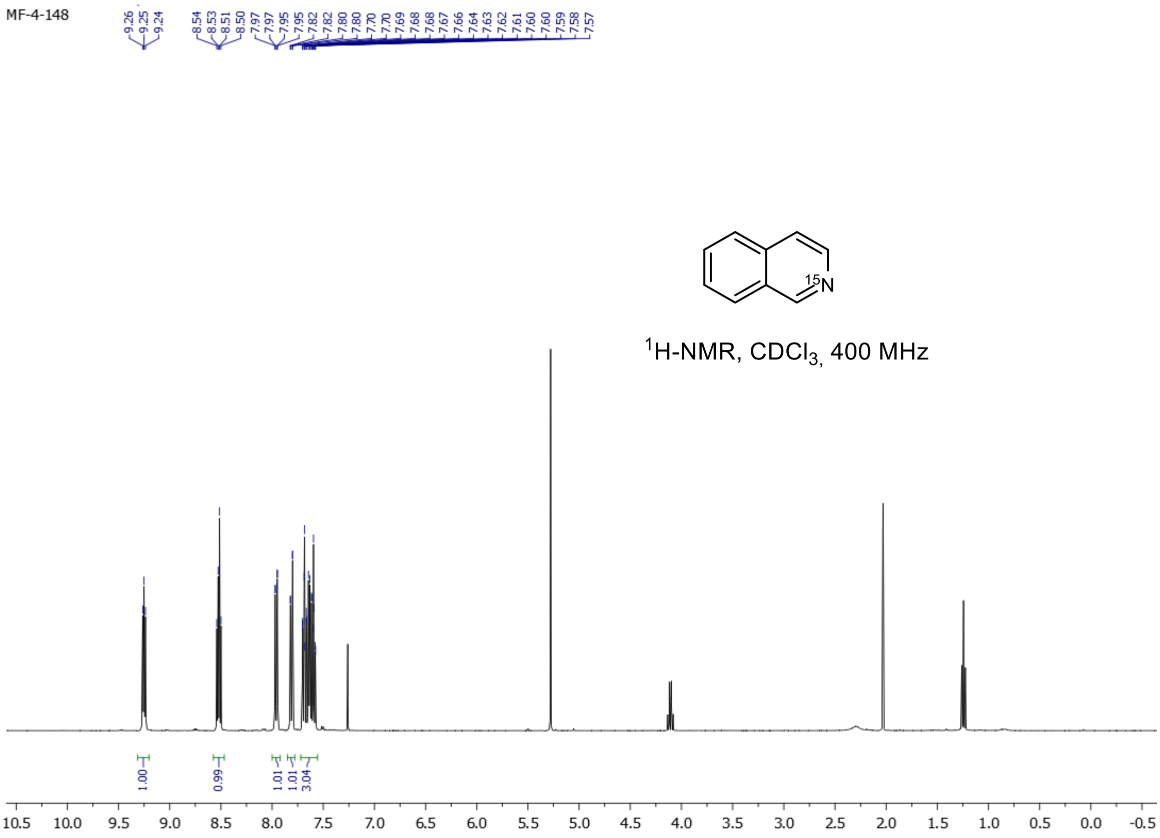

*Due to the volatility of the tilte compound, the NMR spectra contains solvent peaks.

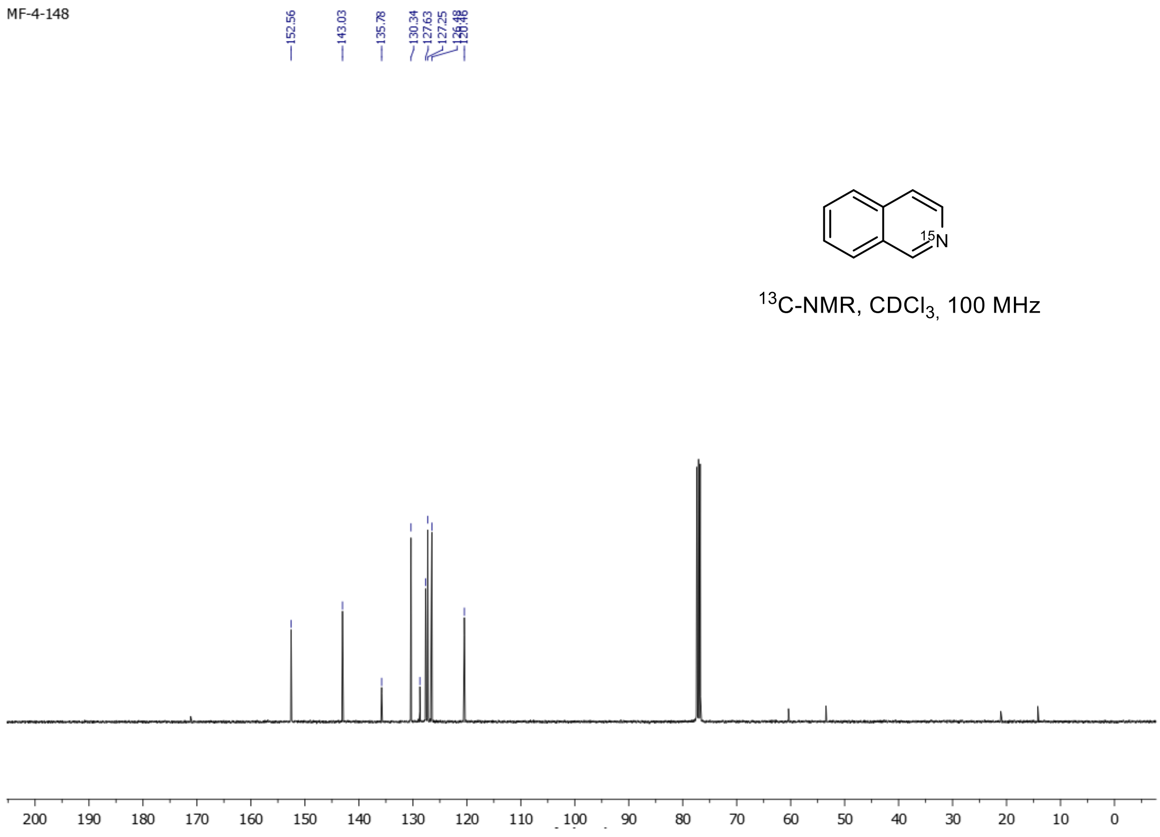

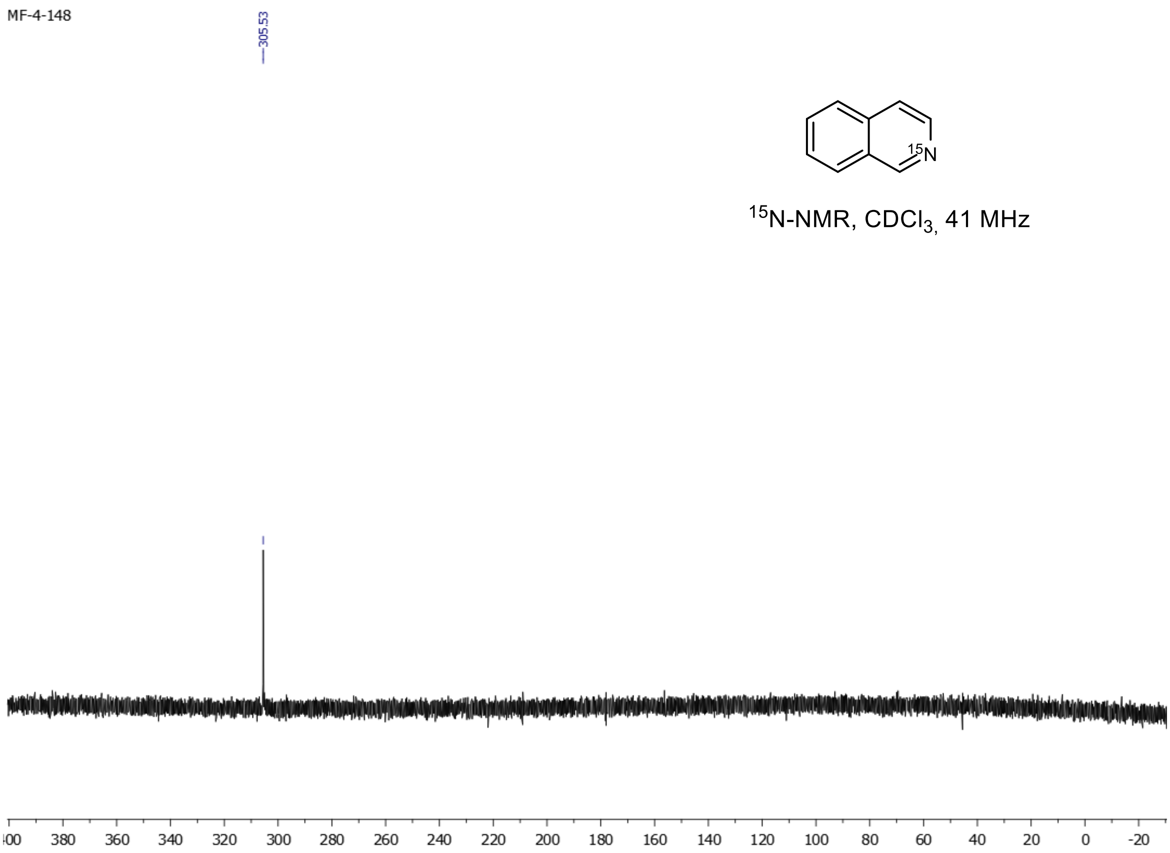

^{15}N-NMR, $CDCl_3$, 41 MHz

Isotopic Enrichment

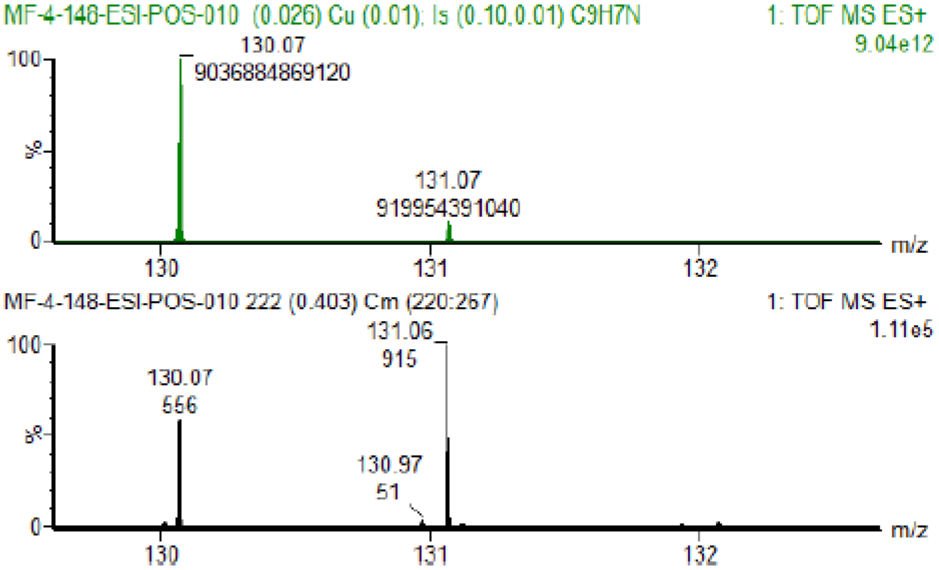

theorical isotopic distribution

	M	M+1	M+2	M+3	M+4	M+5	M+6	M+7
m/z	130	131	132	133	134	135	136	137
%	100,0	10,2	0,4	0,0	0,0	0,0	0,0	0,0

Enrichment calculation

Isotopomer	m/z	Area	natural isotope correction	Corrected area	Isotopic purity (%)
0	130	556	0,00	556,00	40,30
1	131	915	56,71	858,29	62,21
2	132	38	2,22	-51,77	-3,75
3	133	17	0,00	18,85	1,37
4	134	0	0,00	-1,72	-0,12
5	135	0	0,00	0,10	0,01
6	136	0	0,00	0,00	0,00
7	137	0	0,00	0,00	0,00
8	138	0	0,00	0,00	0,00
9	139	0	0,00	0,00	0,00
10	140	0	0,00	0,00	0,00
11	141	0	0,00	0,00	0,00
Total		1526		1379,75	100,00

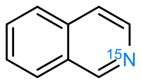

% Isotopic enrichment : 62,2

3-Methylisoquinoline-^{15}N ([^{15}N]28)

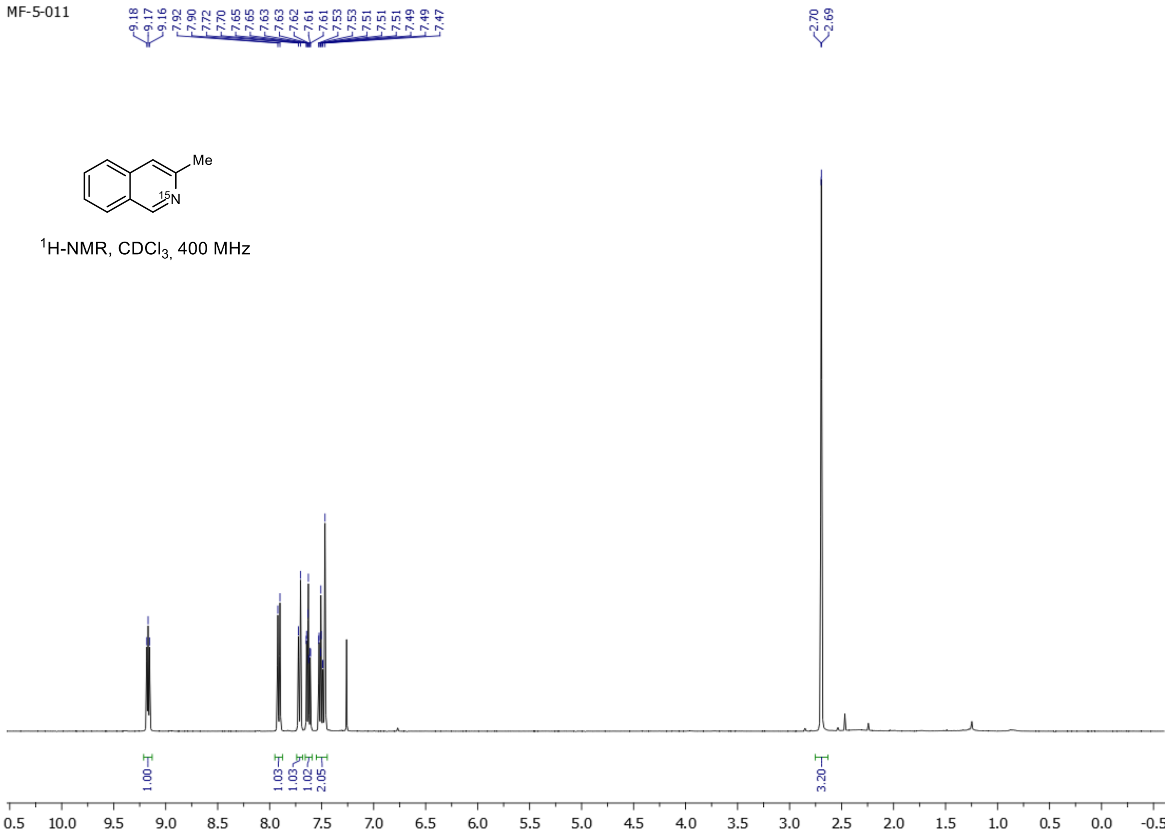

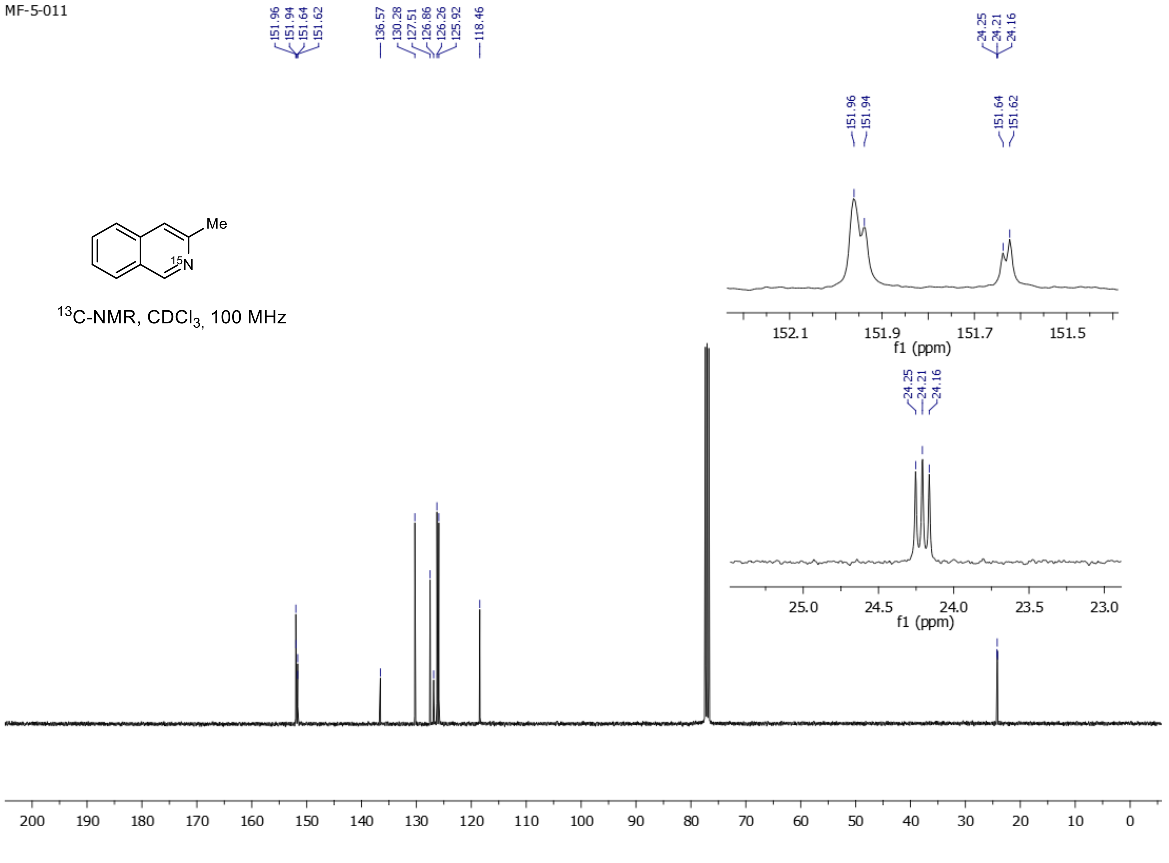

MF-5-011

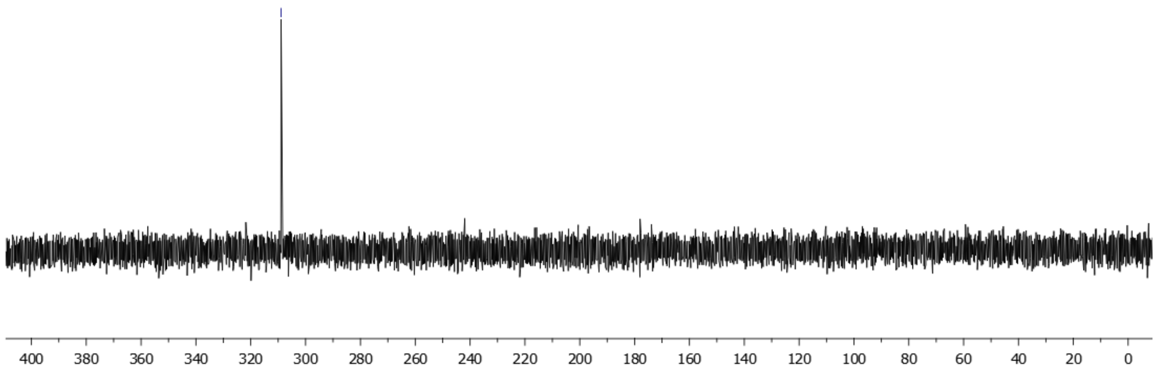

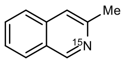

^{15}N-NMR, $CDCl_3$, 41 MHz

Isotopic Enrichment

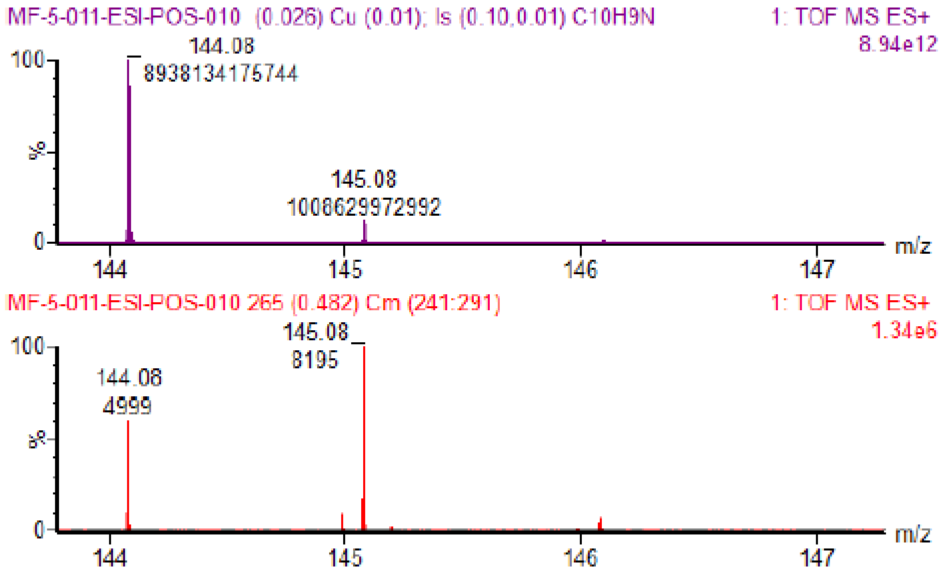

theorical isotopic distribution

	M	M+1	M+2	M+3	M+4	M+5	M+6	M+7
m/z	144	145	146	147	148	149	150	151
%	100,0	11,3	0,5	0,0	0,0	0,0	0,0	0,0

Enrichment calculation

Isotopomer	m/z	Area	natural isotope correction	Corrected area	Isotopic purity (%)
0	144	4999	0,00	4999,00	39,98
1	145	8195	564,89	7630,11	61,03
2	146	770	25,00	-117,20	-0,94
3	147	14	0,00	-10,91	-0,09
4	148	0	0,00	1,82	0,01
5	149	0	0,00	-0,15	0,00
6	150	0	0,00	0,01	0,00
7	151	0	0,00	0,00	0,00
8	152	0	0,00	0,00	0,00
9	153	0	0,00	0,00	0,00
10	154	0	0,00	0,00	0,00
11	155	0	0,00	0,00	0,00
Total		13978		12502,68	100,00

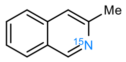

% Isotopic enrichment : 61,0

5-phenylpyrimidine-^{15}N ([^{15}N]29)

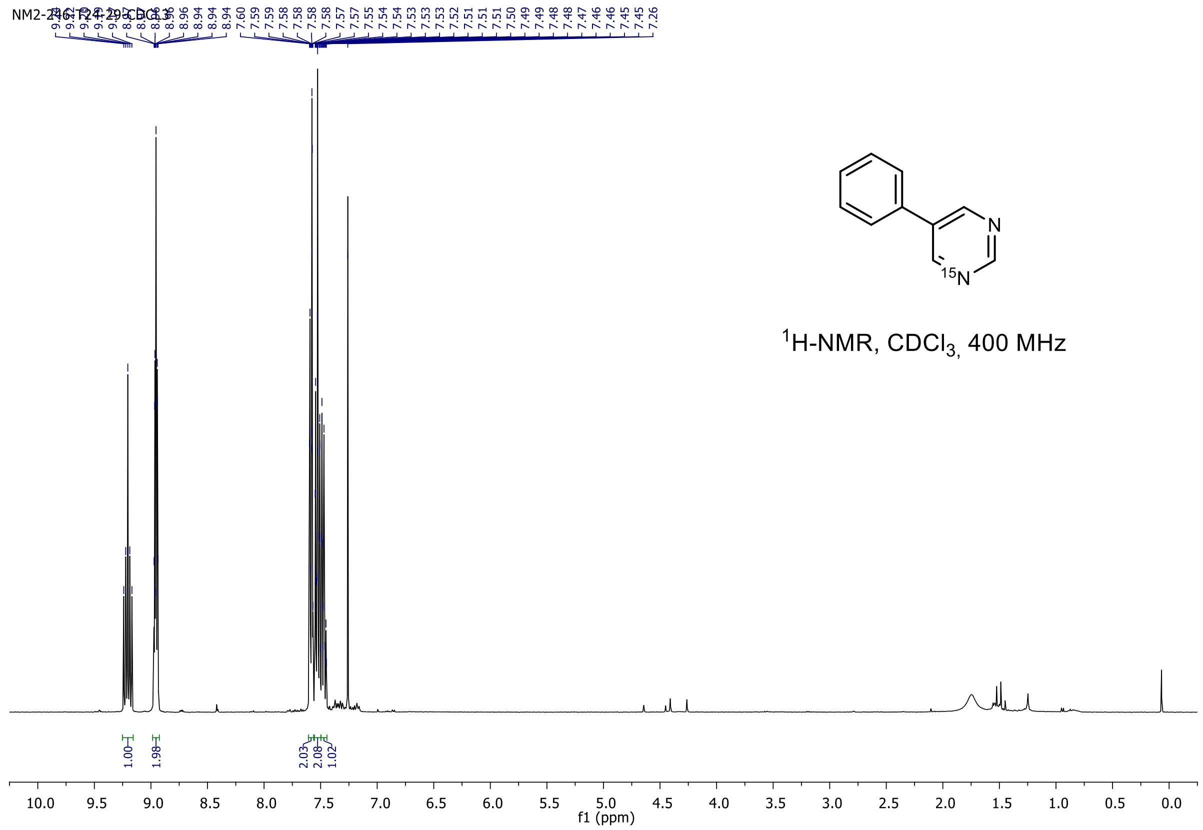

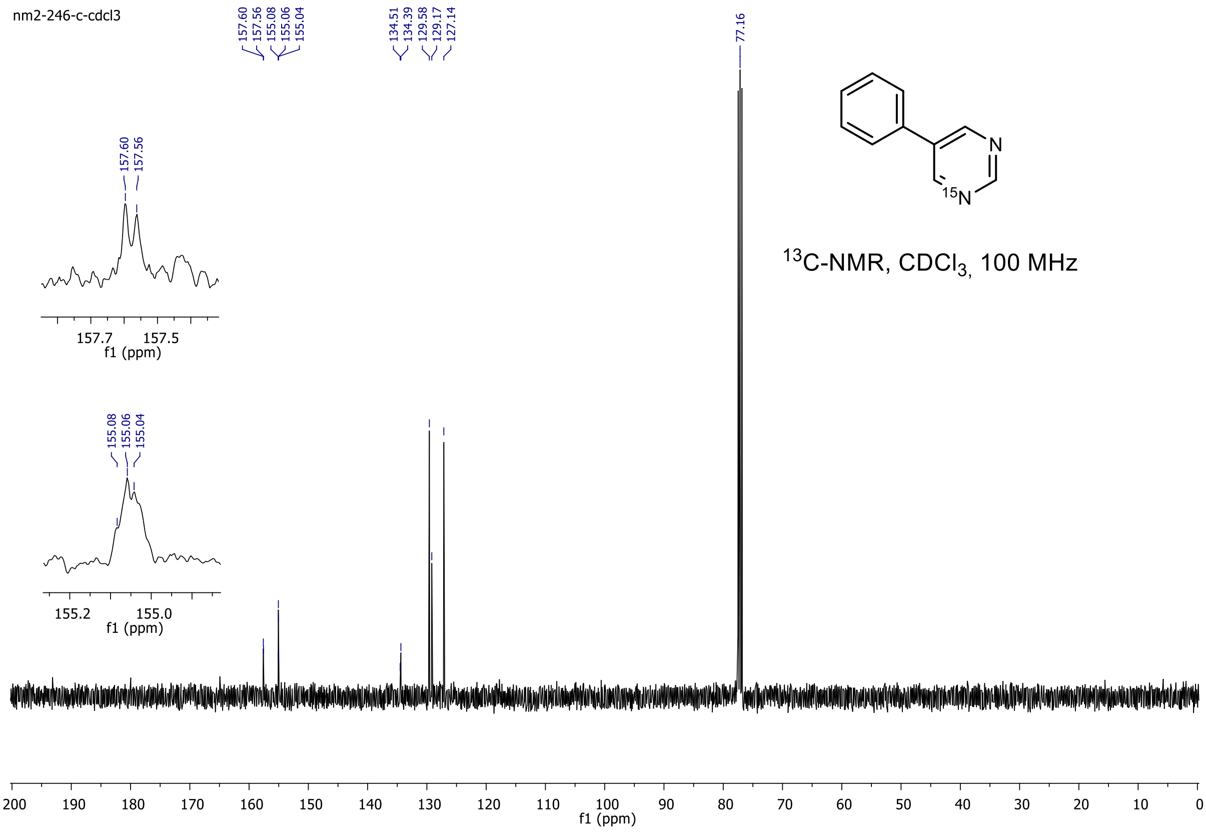

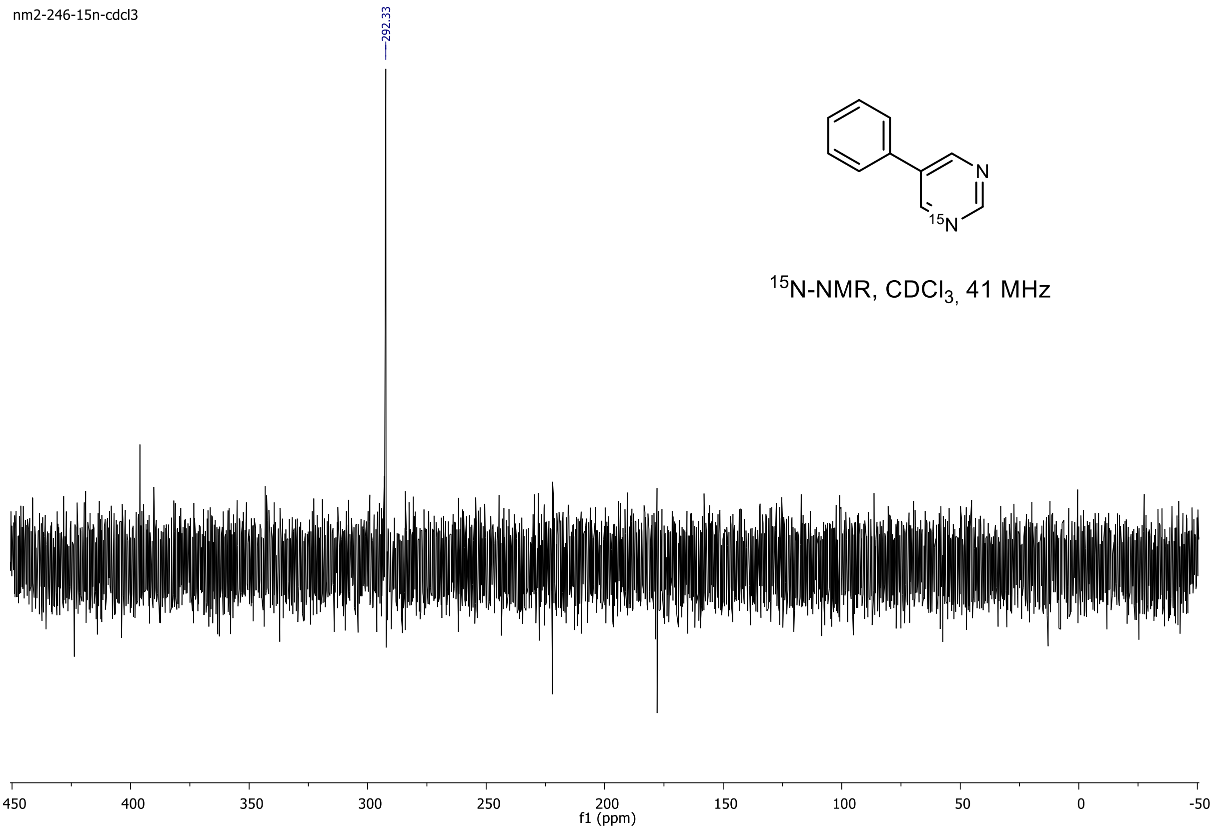
nm2-246-15n-cdcl3
292.33
N
15N
15N-NMR, CDCl3, 41 MHz
450
400
350
300
250
200
150
100
50
0
-50
f1 (ppm)

Isotopic Enrichment

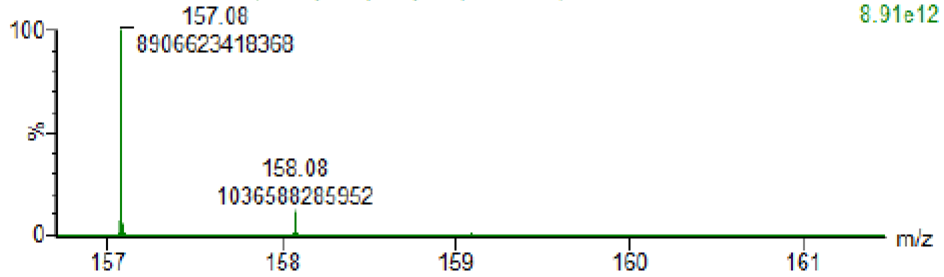

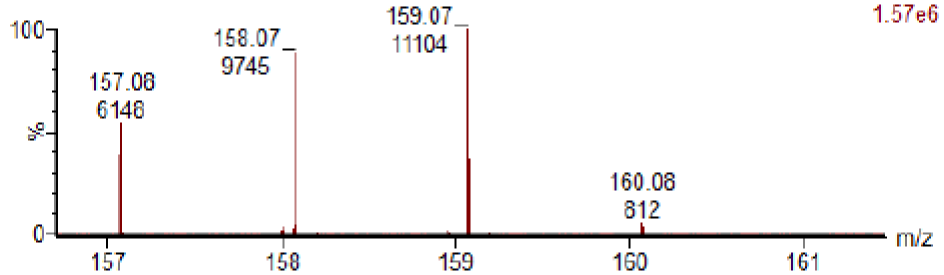

theorical isotopic distribution

	M	M+1	M+2	M+3	M+4	M+5	M+6	M+7
m/z	157	158	159	160	161	162	163	164
%	100,0	11,7	0,6	0,0	0,0	0,0	0,0	0,0

Enrichment calculation

Isotopomer	m/z	Area	natural isotope correction	Corrected area	Isotopic purity (%)
0	157	6148	0,00	6148,00	24,81
1	158	9745	719,32	9025,68	36,43
2	159	11104	36,89	10011,11	40,40
3	160	812	0,00	-413,45	-1,67
4	161	16	0,00	4,31	0,02
5	162	0	0,00	1,98	0,01
6	163	0	0,00	-0,26	0,00
7	164	0	0,00	0,02	0,00
8	165	0	0,00	0,00	0,00
9	166	0	0,00	0,00	0,00
10	167	0	0,00	0,00	0,00
11	168	0	0,00	0,00	0,00
Total		27825		24777,38	100,00

36.4%

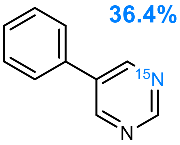

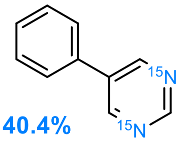

40.4%

% Isotopic enrichment : **36,4** (1 x ^{15}N)

40,4 (2 x ^{15}N)

4-Phenylpyrimidine-1-^{15}N ([^{15}N]30)

^{1}H-NMR, $CDCl_3$, 400 MHz

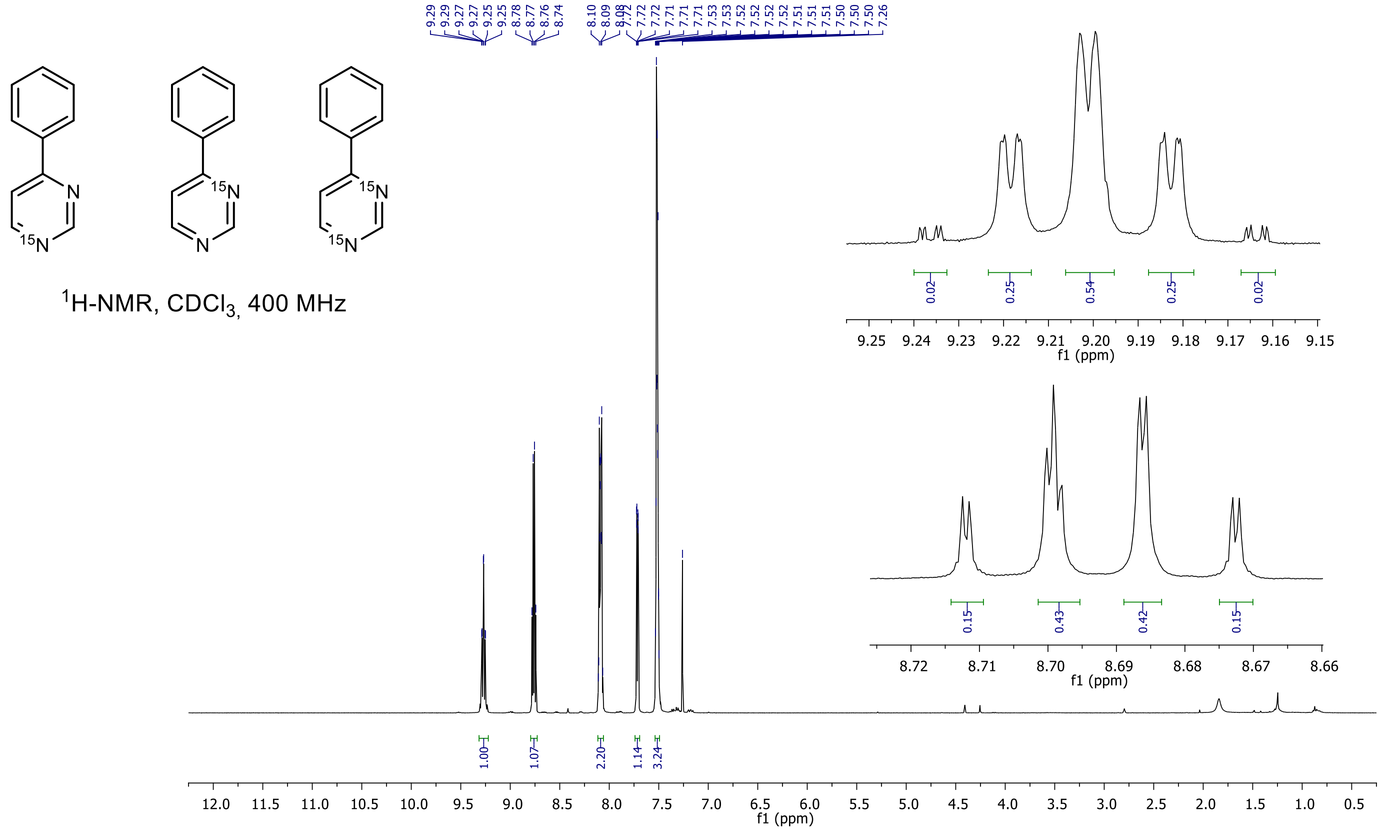

^{13}C-NMR, $CDCl_3$, 100 MHz

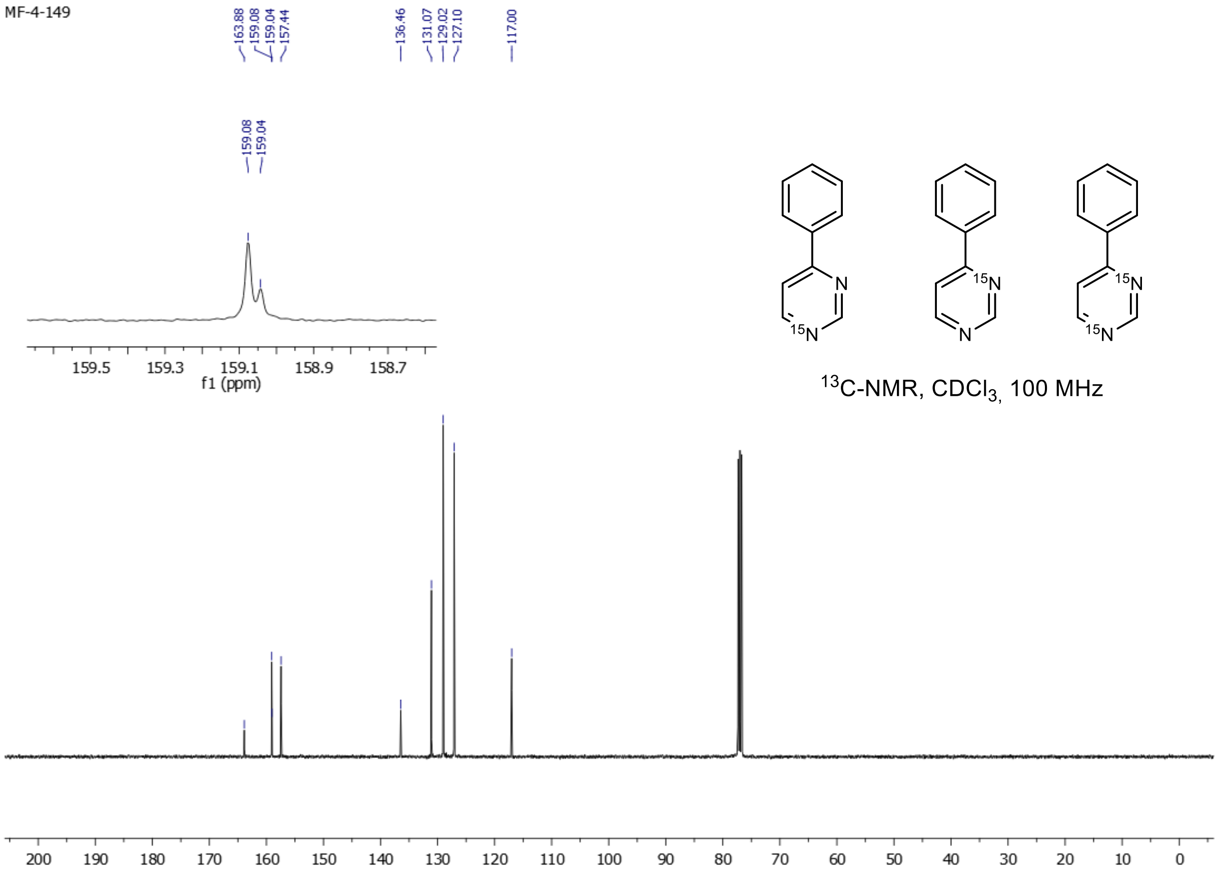

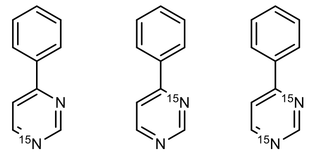

^{15}N-NMR coupled with ^{1}H, acetone-d_6, 41 MHz

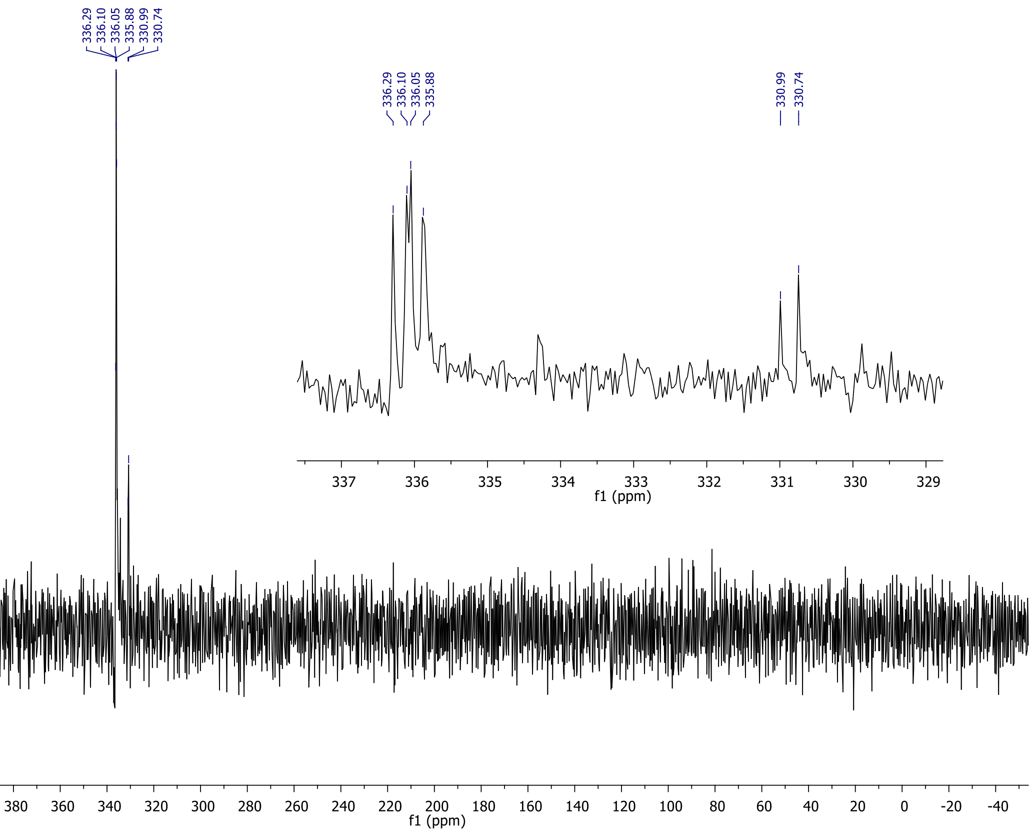

Isotopic Enrichment

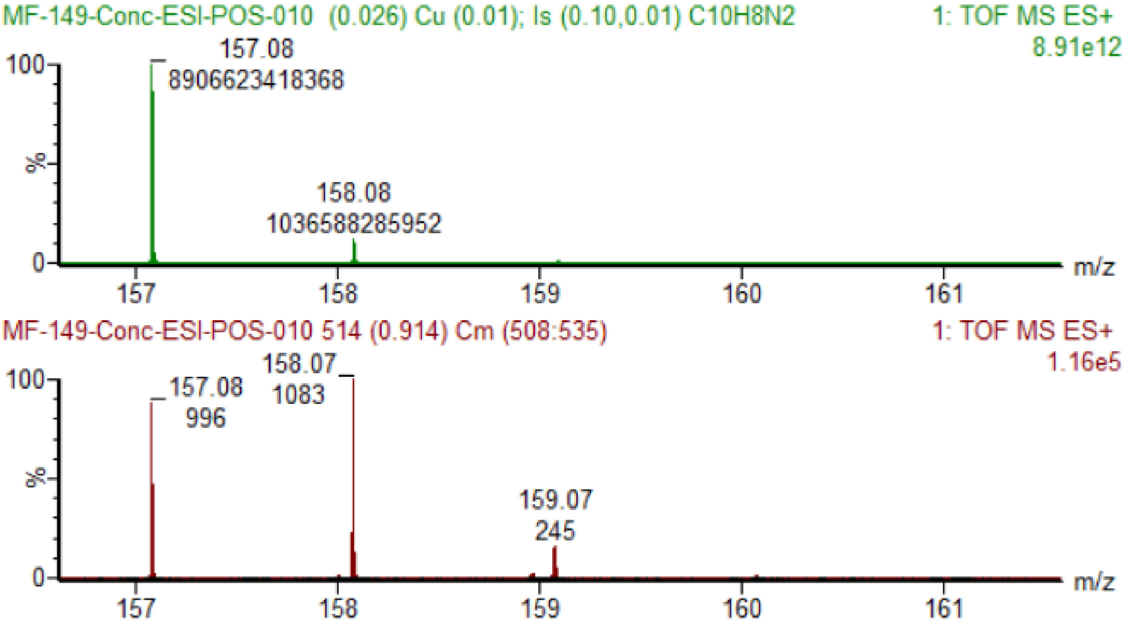

__theorical isotopic distribution__

	M	M+1	M+2	M+3	M+4	M+5	M+6	M+7
m/z	157	158	159	160	161	162	163	164
%	100,0	11,7	0,6	0,0	0,0	0,0	0,0	0,0

__Enrichment calculation__

Isotopomer	m/z	Area	natural isotope correction	Corrected area	Isotopic purity (%)	
0	157	996	0,00	996,00	47,78	
1	158	1083	116,53	966,47	46,36	$[^{15}N_1]$30
2	159	245	5,98	125,95	6,04	$[^{15}N_2]$30
3	160	17	0,00	-3,53	-0,17	
4	161	0	0,00	-0,34	-0,02	
5	162	0	0,00	0,06	0,00	
6	163	0	0,00	-0,01	0,00	
7	164	0	0,00	0,00	0,00	
8	165	0	0,00	0,00	0,00	
9	166	0	0,00	0,00	0,00	
10	167	0	0,00	0,00	0,00	
11	168	0	0,00	0,00	0,00	
Total		2341		2084,59	100,00	

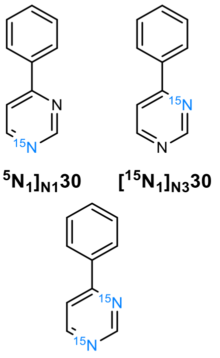

$[^{15}N_1]_{N1}$30 $[^{15}N_1]_{N3}$30 $[^{15}N_2]$30

% Isotopic enrichment : 46,4

*Details of the calculation for NMR IE determination of **$[^{15}N_1]_{N1}$30** and **$[^{15}N_1]_{N3}$30** :*

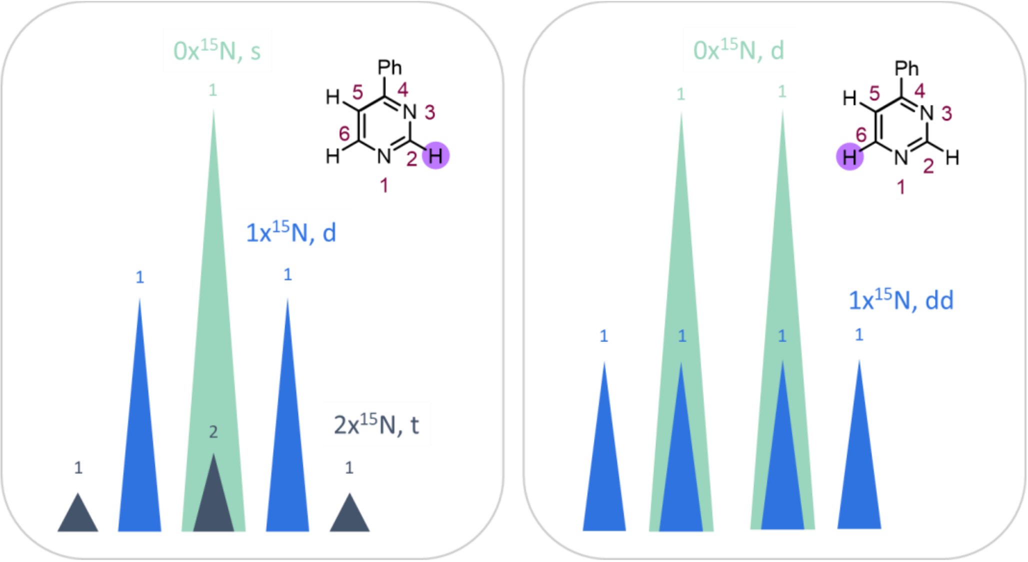

Theoretical multiplicity of H-C^2 (left) and H-C^6 (right) in ^{1}H NMR.

Protons H-C^2 and H-C^6 exhibit a different multiplicity when coupling occurs with the ^{15}N nucleus as shown is the above figure. Thanks to the large $^2J_{N\text{-}H}$ coupling constant value (J = 10-15 Hz), signals from labeled compounds are easily distinguishable from signals of unlabeled ones. Relative integration of splitted and unsplitted NMR signals of H-C^2 and H-C^6 (displayed on the ^{1}H NMR spectrum) allows the determination of the isotopic enrichment of each N position with good accuracy. Relative integration of the signals of H-C^2 confirmed HRMS results, with 46% of mono-labeled (**$[^{15}N_1]_{N1}$30** and **$[^{15}N_1]_{N3}$30**) and 8% of double-labeled product (**$[^{15}N_2]$30**).

Relative integration of the signals of H-C^6 allows the determination of the proportion of **$[^{15}N_1]_{N1}$30**. %**$[^{15}N_1]_{N1}$30** = 0.13*4 - %**$[^{15}N_2]$30** = **44%.**

The proportion of **$[^{15}N_1]_{N3}$30** can be determined from the above presented results, subtracting the proportion of **$[^{15}N_1]_{N1}$30** from the total proportion of mono-labeled product. %**$[^{15}N_1]_{N3}$30** = 46 – 44 = 2%.

^{15}N-Nicotinamide (S5)

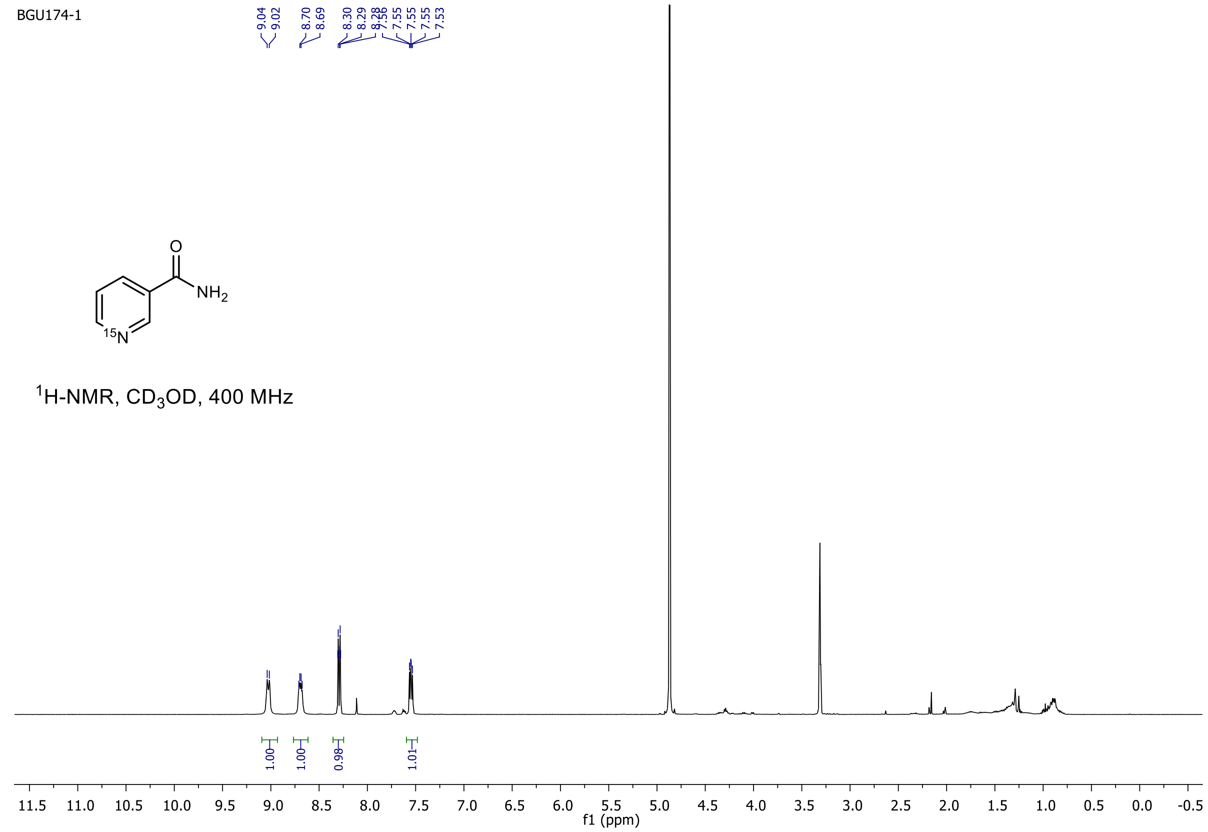

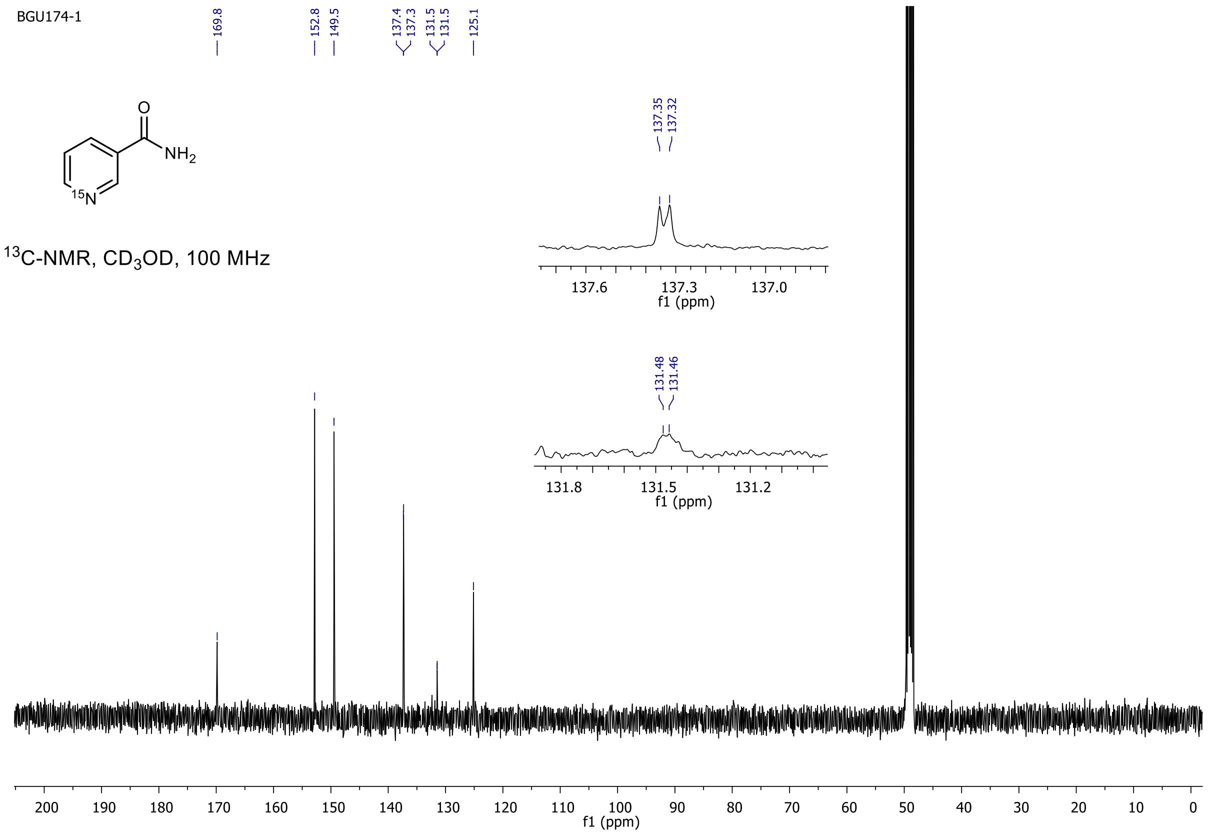

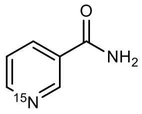

^{15}N-NMR, CD_3OD, 41 MHz

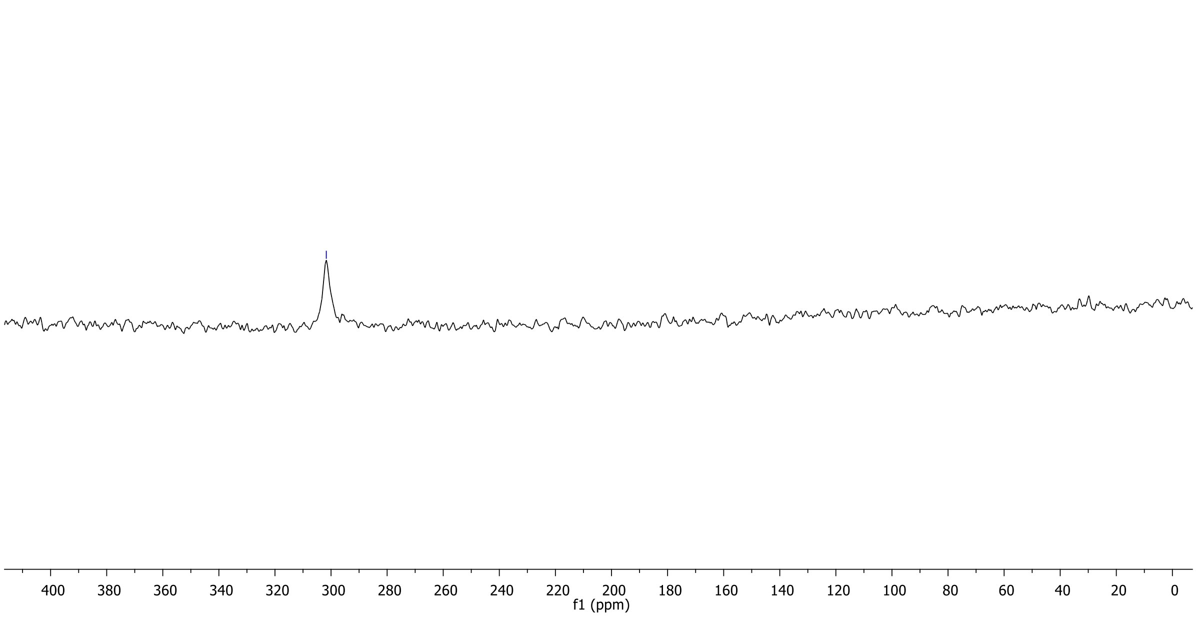

Isotopic Enrichment

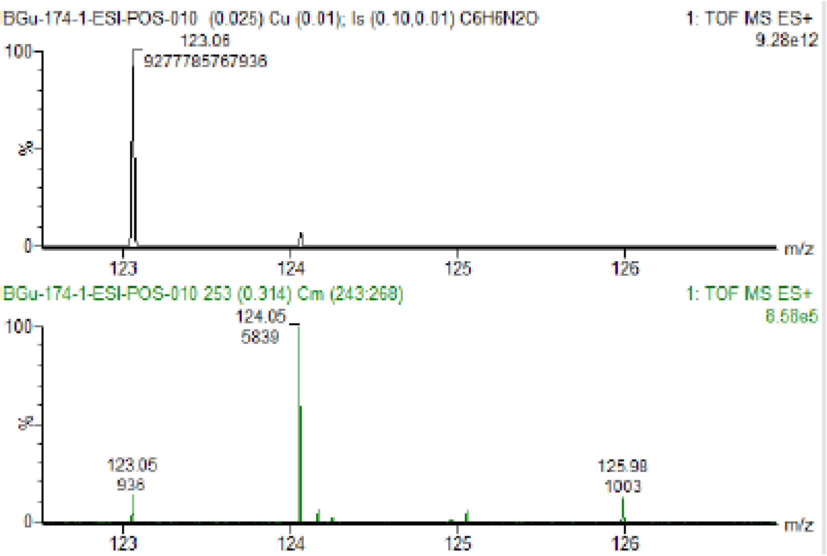

theorical isotopic distribution

	M	M+1	M+2	M+3	M+4	M+5	M+6	M+7
m/z	123	124	125	126	127	128	129	130
%	100,0	7,3	0,4	0,0	0,0	0,0	0,0	0,0

Enrichment calculation

Isotopomer	m/z	Area	natural isotope correction	Corrected area	Isotopic purity (%)
0	123	936	0,00	936,00	13,90
1	124	5839	68,33	5770,67	85,69
2	125	459	3,74	34,00	0,50
3	126	19	0,00	-6,56	-0,10
4	127	0	0,00	0,34	0,01
5	128	0	0,00	0,00	0,00
6	129	0	0,00	0,00	0,00
7	130	0	0,00	0,00	0,00
8	131	0	0,00	0,00	0,00
9	132	0	0,00	0,00	0,00
10	133	0	0,00	0,00	0,00
11	134	0	0,00	0,00	0,00
Total		7253		6734,45	100,00

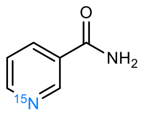

% Isotopic enrichment : 85,7

Methyl nicotinate-^{15}N (S6)

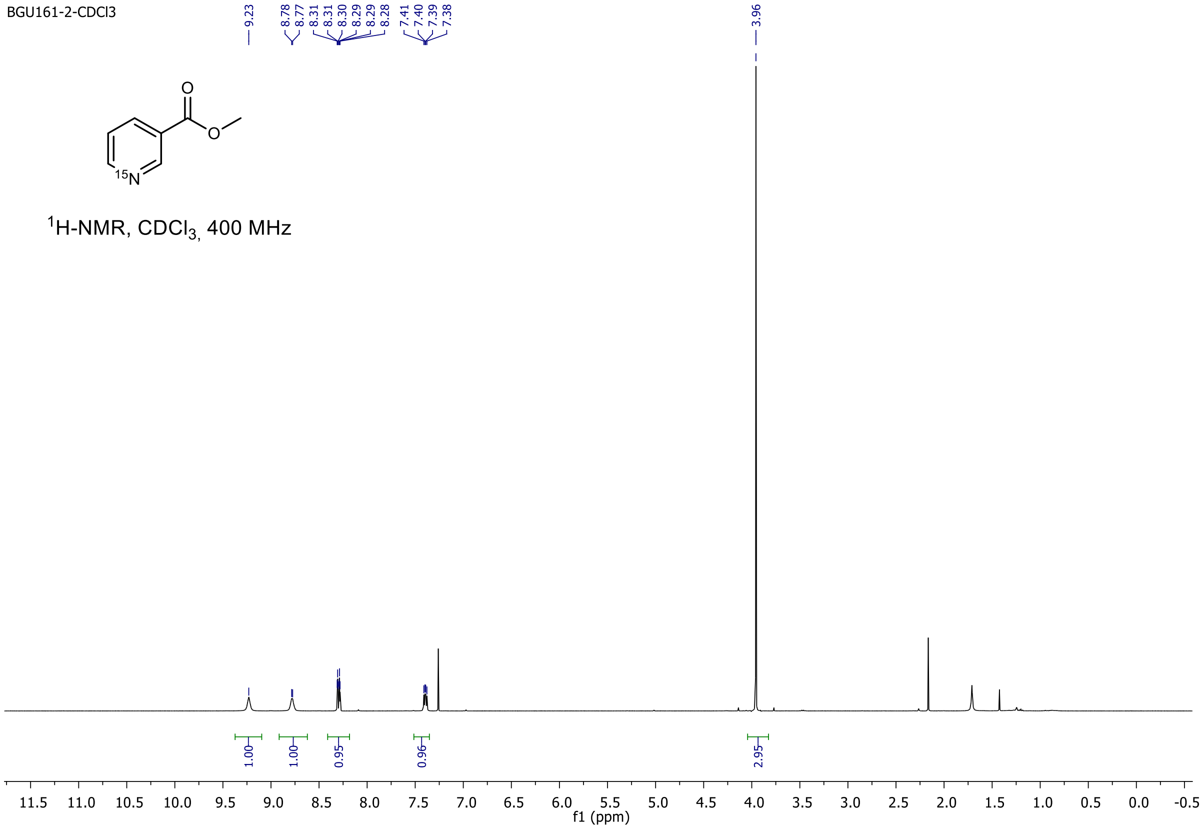

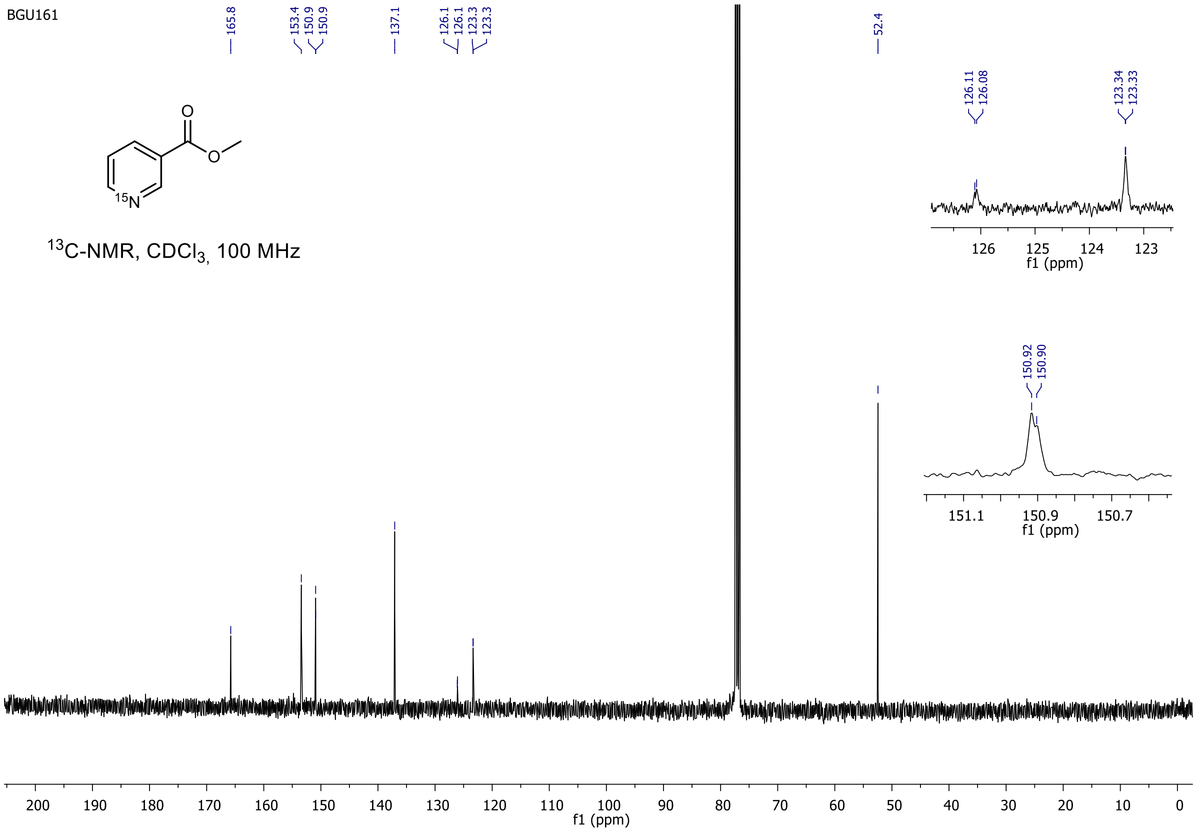

Isotopic Enrichment

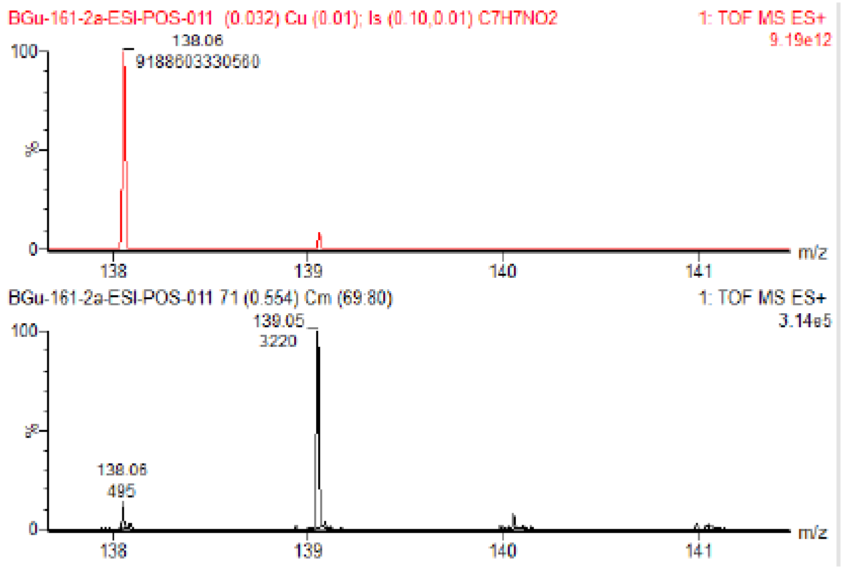

theorical isotopic distribution

	M	M+1	M+2	M+3	M+4	M+5	M+6	M+7
**m/z**	138	139	140	141	142	143	144	145
**%**	100,0	8,1	0,6	0,0	0,0	0,0	0,0	0,0

Enrichment calculation

Isotopomer	m/z	Area	natural isotope correction	Corrected area	Isotopic purity (%)
0	138	495	0,00	495,00	13,23
1	139	3220	40,10	3179,91	84,99
2	140	292	2,97	31,46	0,84
3	141	60	0,00	38,37	1,03
4	142	0	0,00	-3,30	-0,09
5	143	0	0,00	0,04	0,00
6	144	0	0,00	0,02	0,00
7	145	0	0,00	0,00	0,00
8	146	0	0,00	0,00	0,00
9	147	0	0,00	0,00	0,00
10	148	0	0,00	0,00	0,00
11	149	0	0,00	0,00	0,00
Total		4067		3741,49	100,00

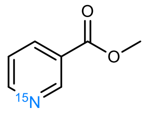

% Isotopic enrichment : 85,0

13.3 ^{15}N-labeled pharmaceutical molecules

^{15}N-Etofibrate ([^{15}N]31)

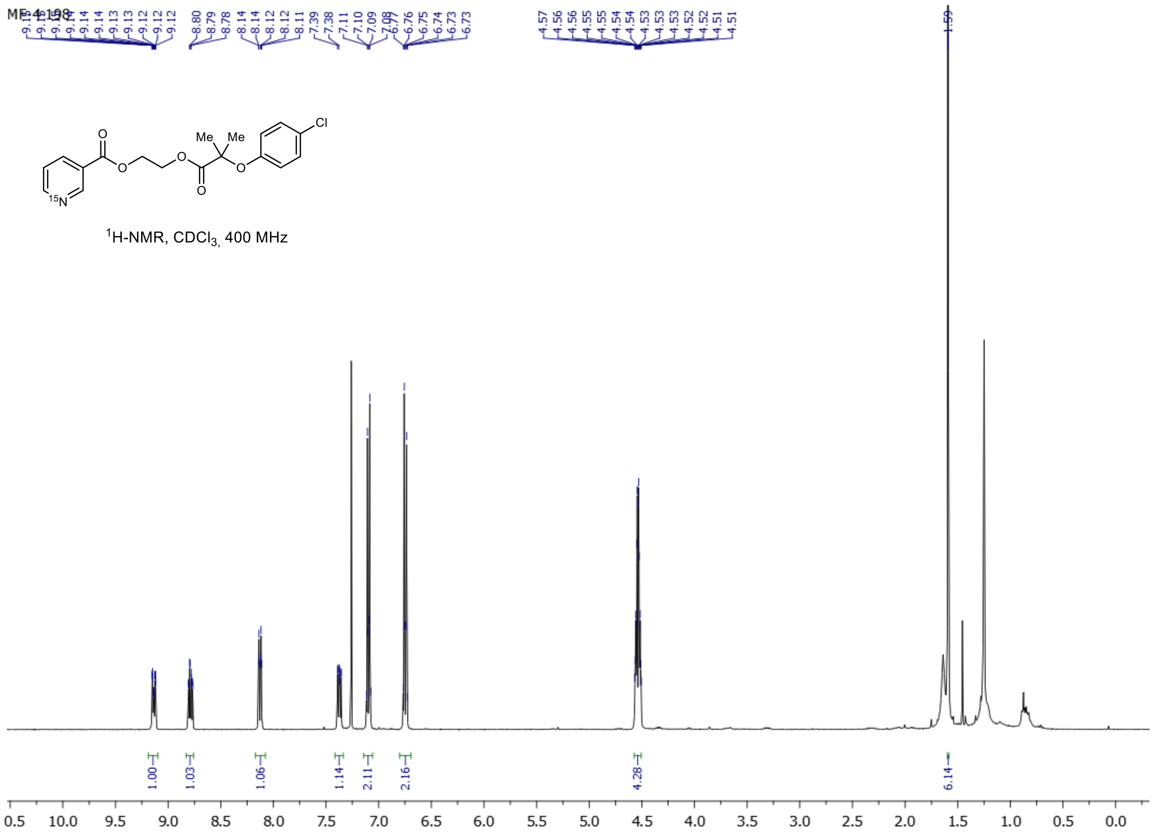

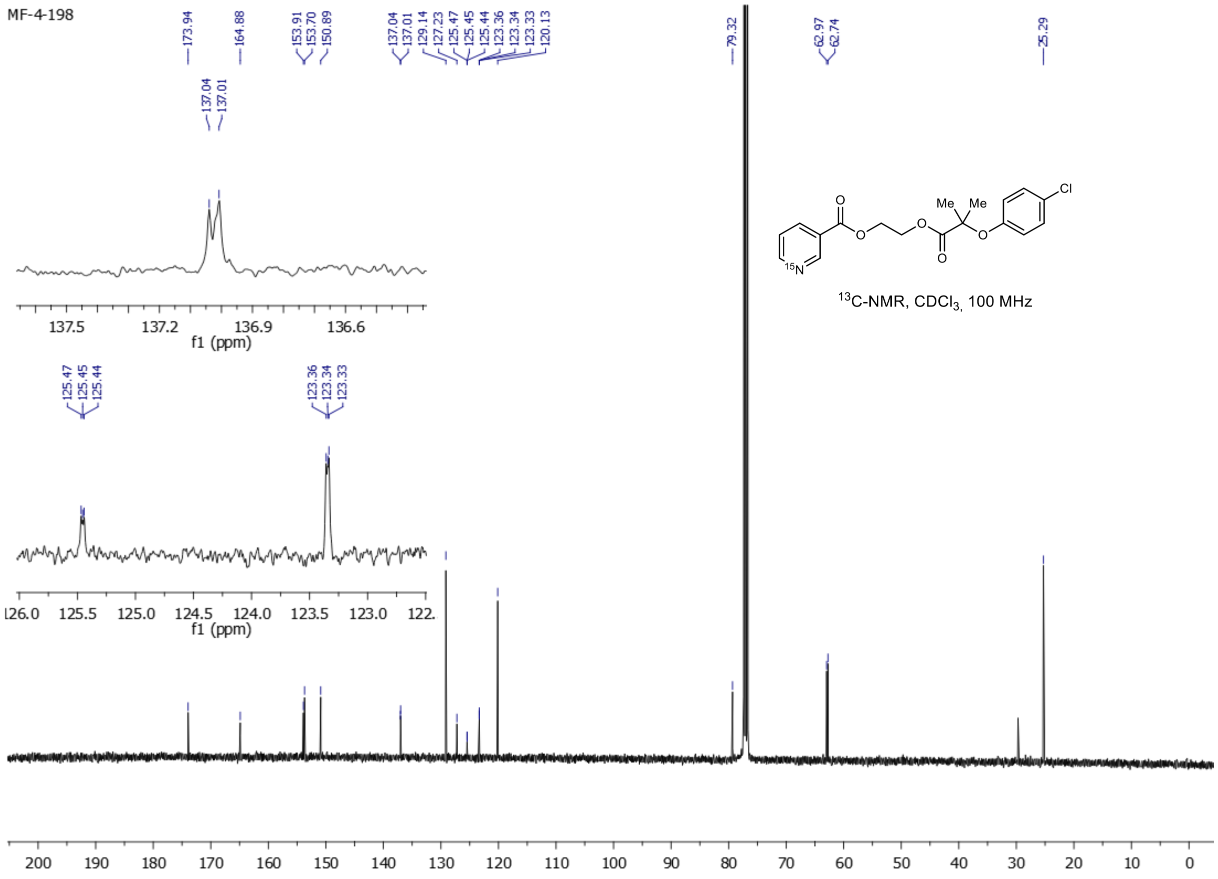

MF-4-198

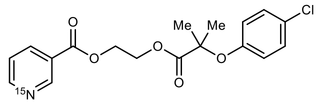

^{15}N-NMR, $CDCl_3$, 41 MHz

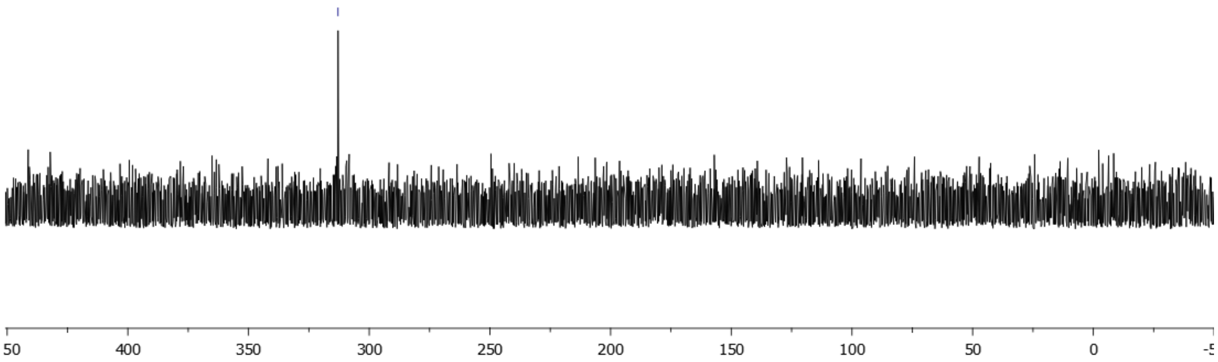

Isotopic Enrichment

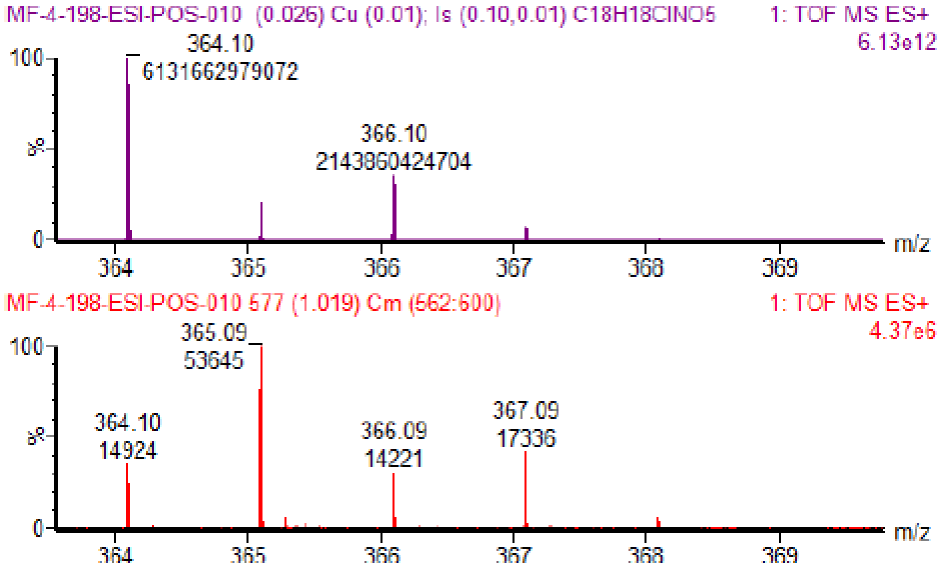

theorical isotopic distribution

	M	M+1	M+2	M+3	M+4	M+5	M+6	M+7
m/z	364	365	366	367	368	369	370	371
%	100,0	20,3	35,3	6,9	0,9	0,1	0,0	0,0

Enrichment calculation

Isotopomer	m/z	Area	natural isotope correction	Corrected area	Isotopic purity (%)
0	364	14924	0,00	14924,00	23,63
1	365	53645	3029,57	50615,43	80,14
2	366	14221	5268,17	-1322,10	-2,09
3	367	17336	1029,76	-1292,61	-2,05
4	368	2837	134,32	-60,68	-0,10
5	369	279	14,92	368,37	0,58
6	370	27	0,00	24,11	0,04
7	371	0	0,00	-117,79	-0,19
8	372	0	0,00	-8,18	-0,01
9	373	0	0,00	38,32	0,06
10	374	0	0,00	2,65	0,00
11	375	0	0,00	-12,46	-0,02
Total		103269		63159,06	100,00

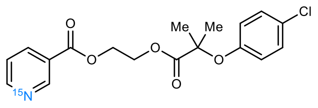

% Isotopic enrichment : 80,1

^{15}N-Nicergoline ([^{15}N]32)

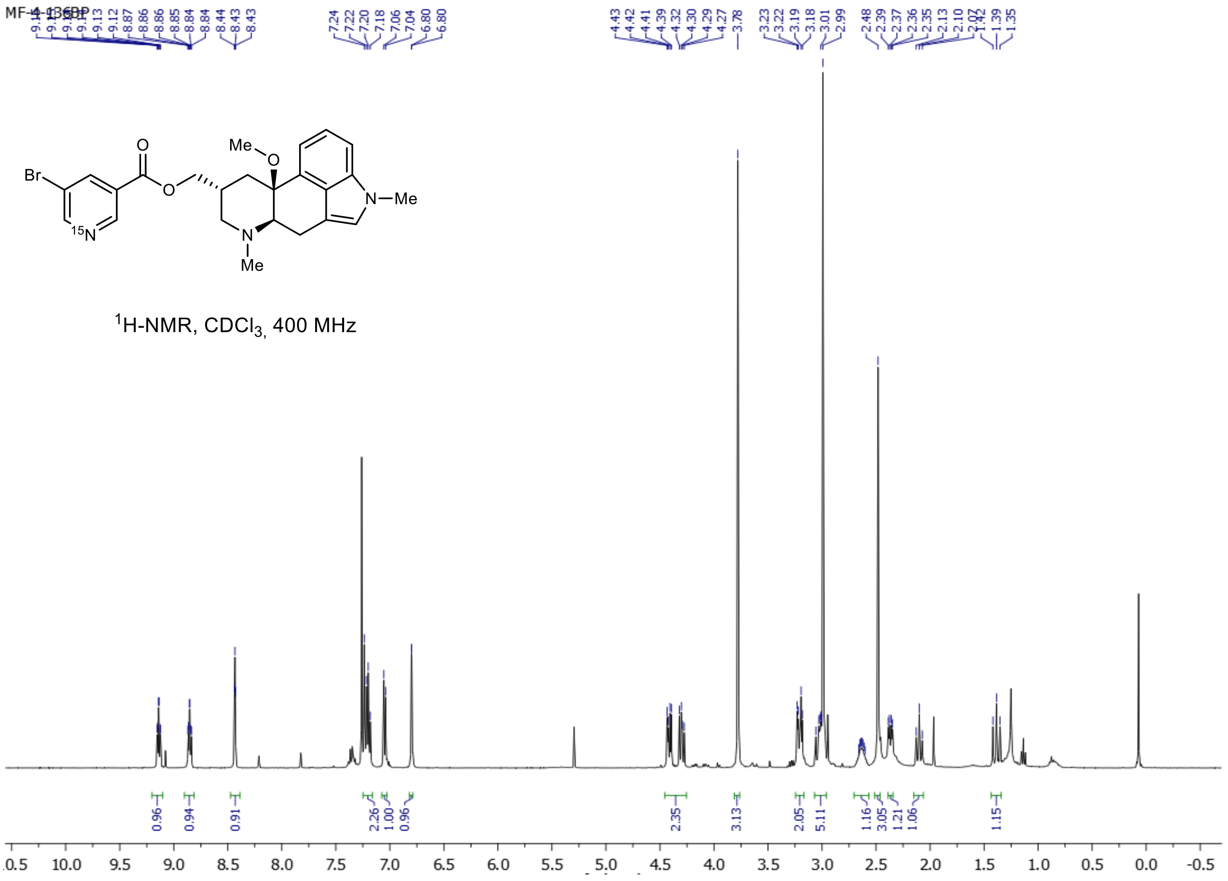

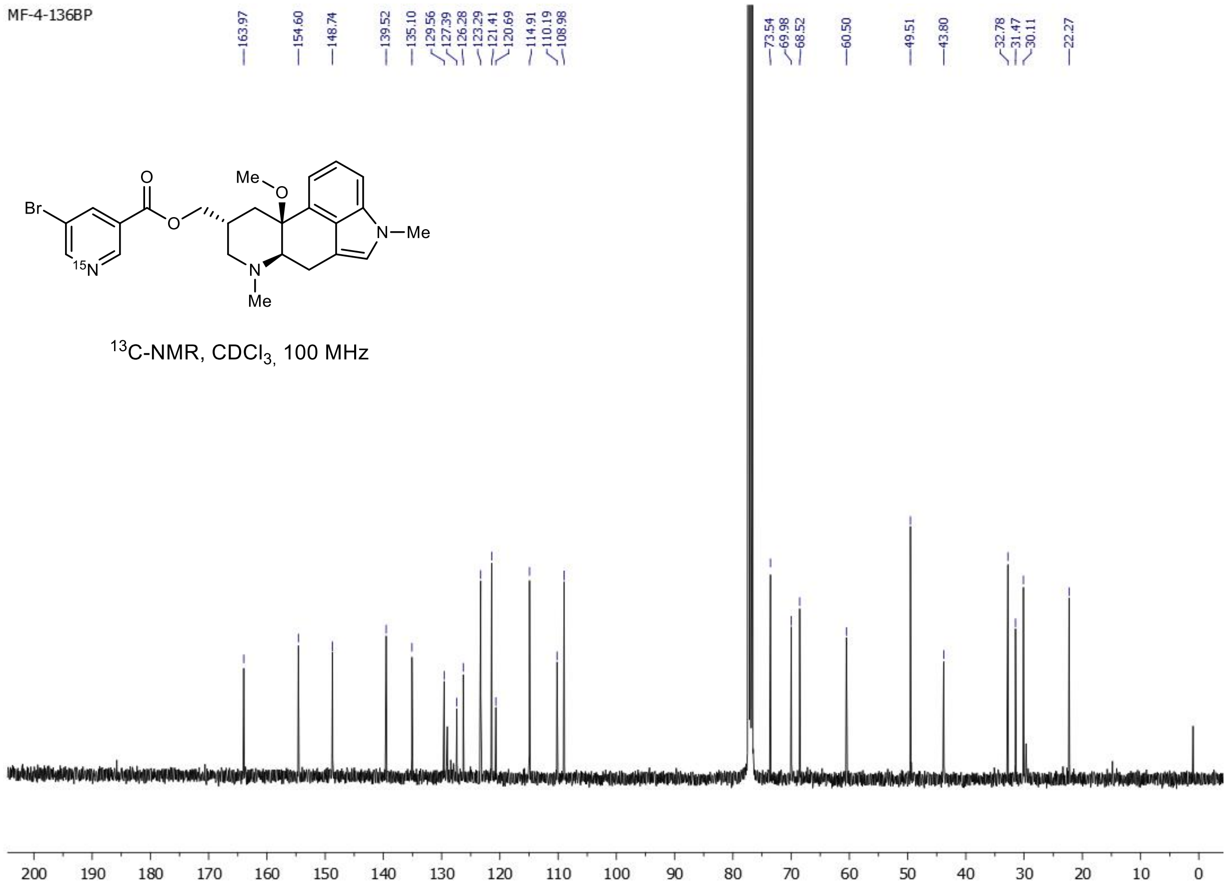

MF-4-136

319.25

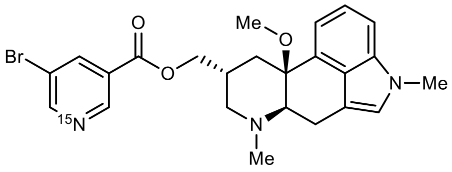

^{15}N-NMR, $CDCl_3$, 41 MHz

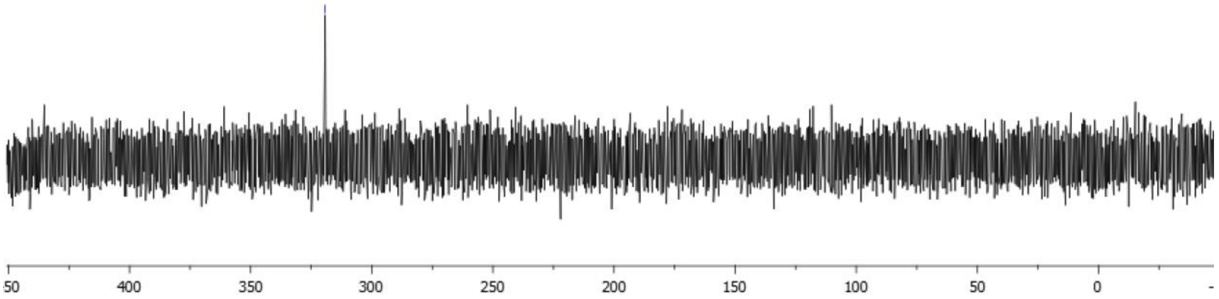

Enrichissement isotopique

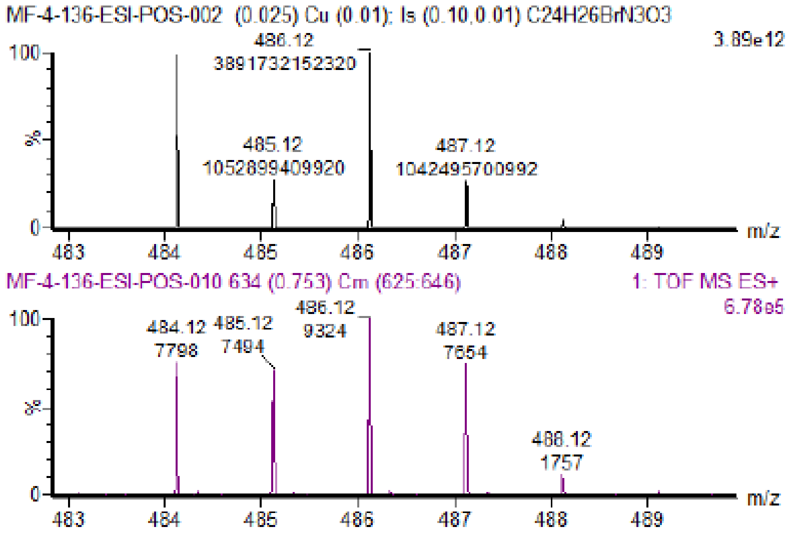

Profil isotopique théorique

	M	M+1	M+2	M+3	M+4	M+5	M+6	M+7
m/z	484	485	486	487	488	489	490	491
%	98,3	27,1	100	26,9	4	0,4	0	0

Calcul de l'enrichissement

Isotopomère	m/z	Aires	Correction isotopes naturels	Aires corrigées	Pureté isotopique (%)
0	484	7798	0,00	7931,47	59,31
1	485	7494	2150,31	5441,99	40,66
2	486	9324	7934,71	-82,24	-0,62
3	487	7654	2134,44	97,36	0,73
4	488	1757	317,39	30,79	0,23
5	489	276	31,74	-52,73	-0,39
6	490	45	0,00	-14,94	-0,11
7	491	0	0,00	40,34	0,30
8	492	0	0,00	15,31	0,11
9	493	0	0,00	-33,65	-0,25
10	494	0	0,00	-14,93	-0,11
11	495	0	0,00	26,94	0,20
Total		34348		13385,70	100,00

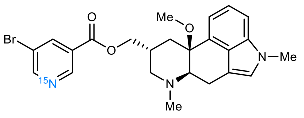

% Enrichissement isotopique : 40,7

^{15}N-Abiraterone acetate ([^{15}N]33)

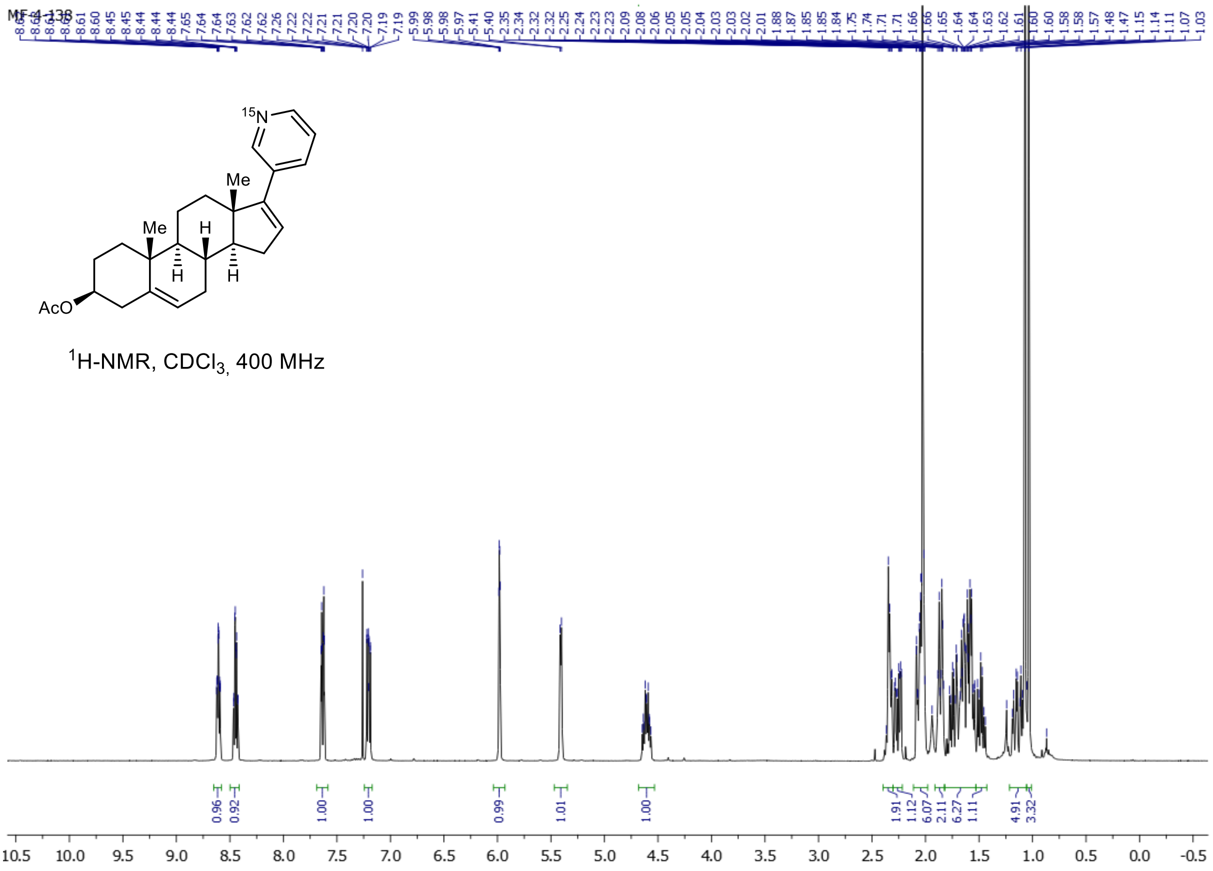

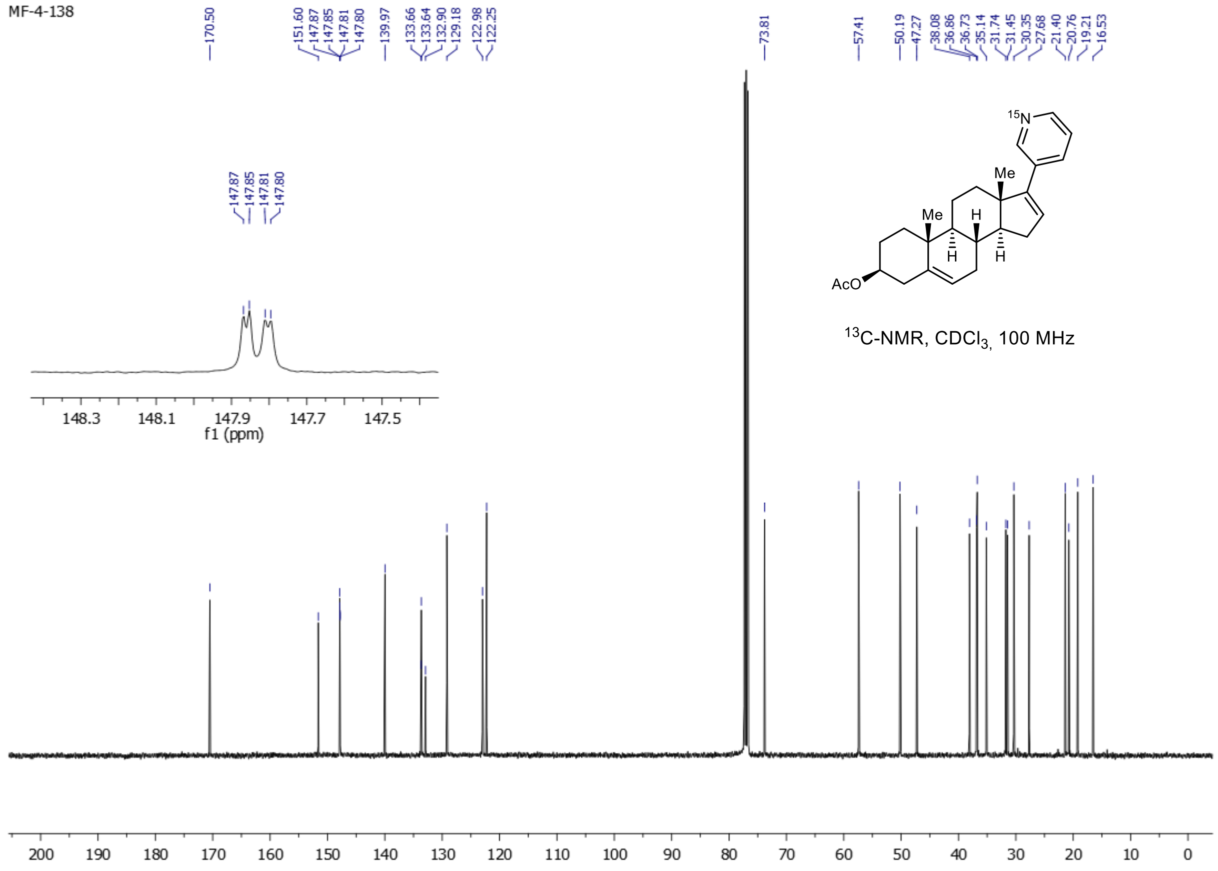

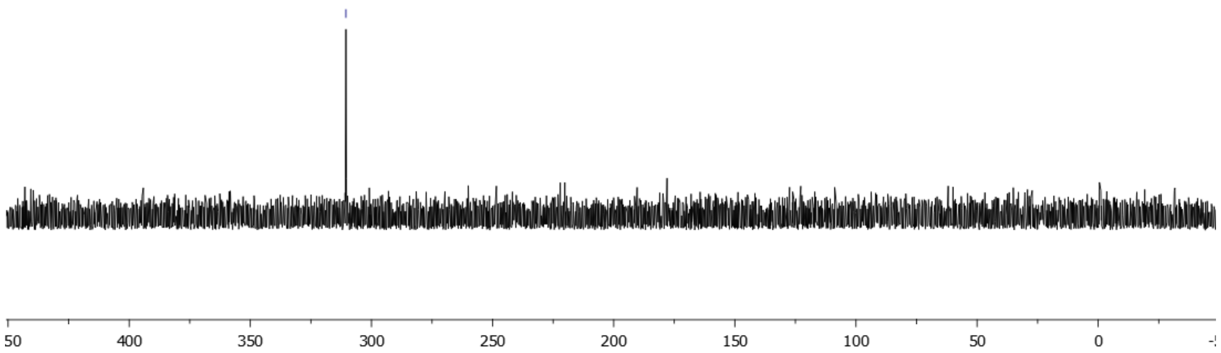

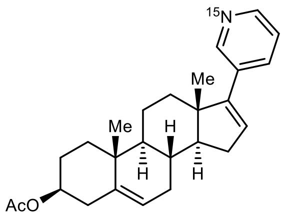

^{15}N-NMR, $CDCl_3$, 41 MHz

Isotopic Enrichment

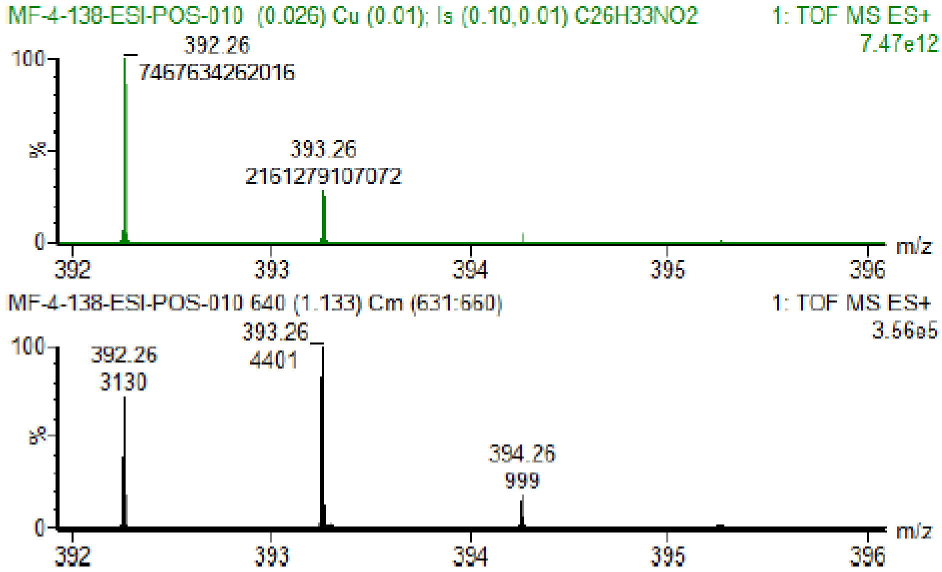

theorical isotopic distribution

	M	M+1	M+2	M+3	M+4	M+5	M+6	M+7
**m/z**	392	393	394	395	396	397	398	399
**%**	100,0	29,1	4,5	0,4	0,0	0,0	0,0	0,0

Enrichment calculation

Isotopomer	m/z	Area	natural isotope correction	Corrected area	Isotopic purity (%)
0	392	3130	0,00	3130,00	48,57
1	393	4401	910,83	3490,17	54,16
2	394	999	140,85	-157,49	-2,44
3	395	100	12,52	-23,75	-0,37
4	396	6	0,00	6,04	0,09
5	397	0	0,00	-0,06	0,00
6	398	0	0,00	-0,16	0,00
7	399	0	0,00	0,02	0,00
8	400	0	0,00	0,00	0,00
9	401	0	0,00	0,00	0,00
10	402	0	0,00	0,00	0,00
11	403	0	0,00	0,00	0,00
Total		8636		6444,78	100,00

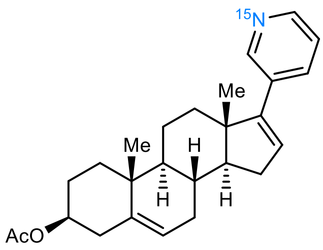

% Isotopic enrichment : 54,2

[15]N-Nicoboxil ([^{15}N]34)

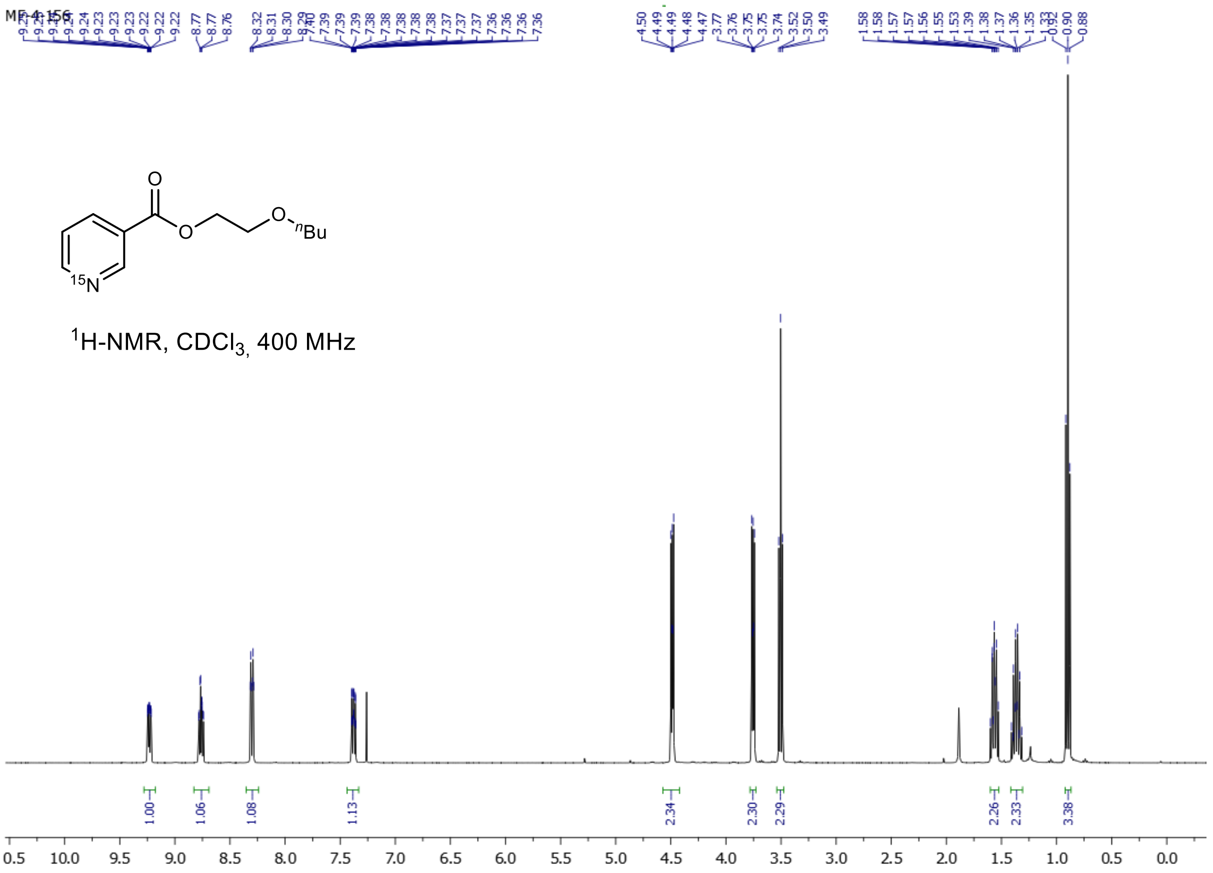

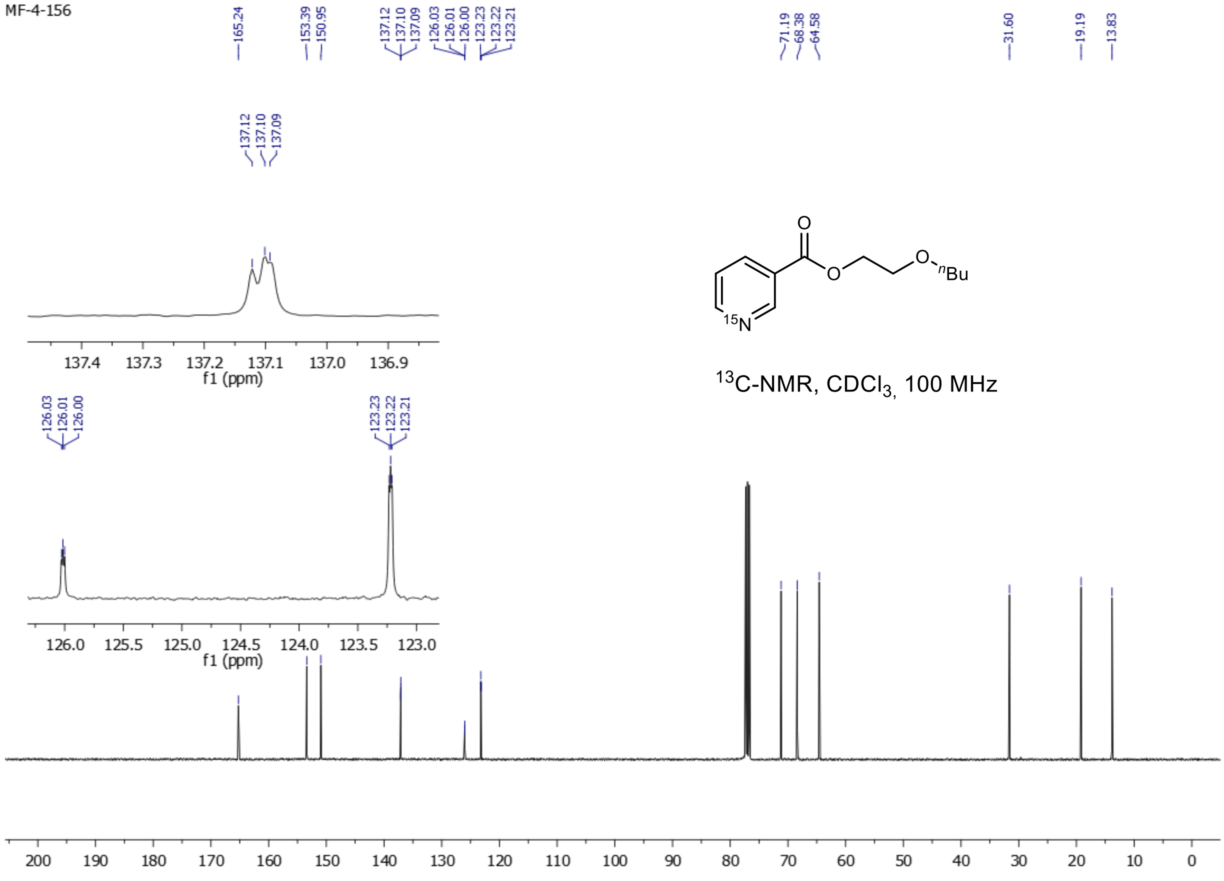

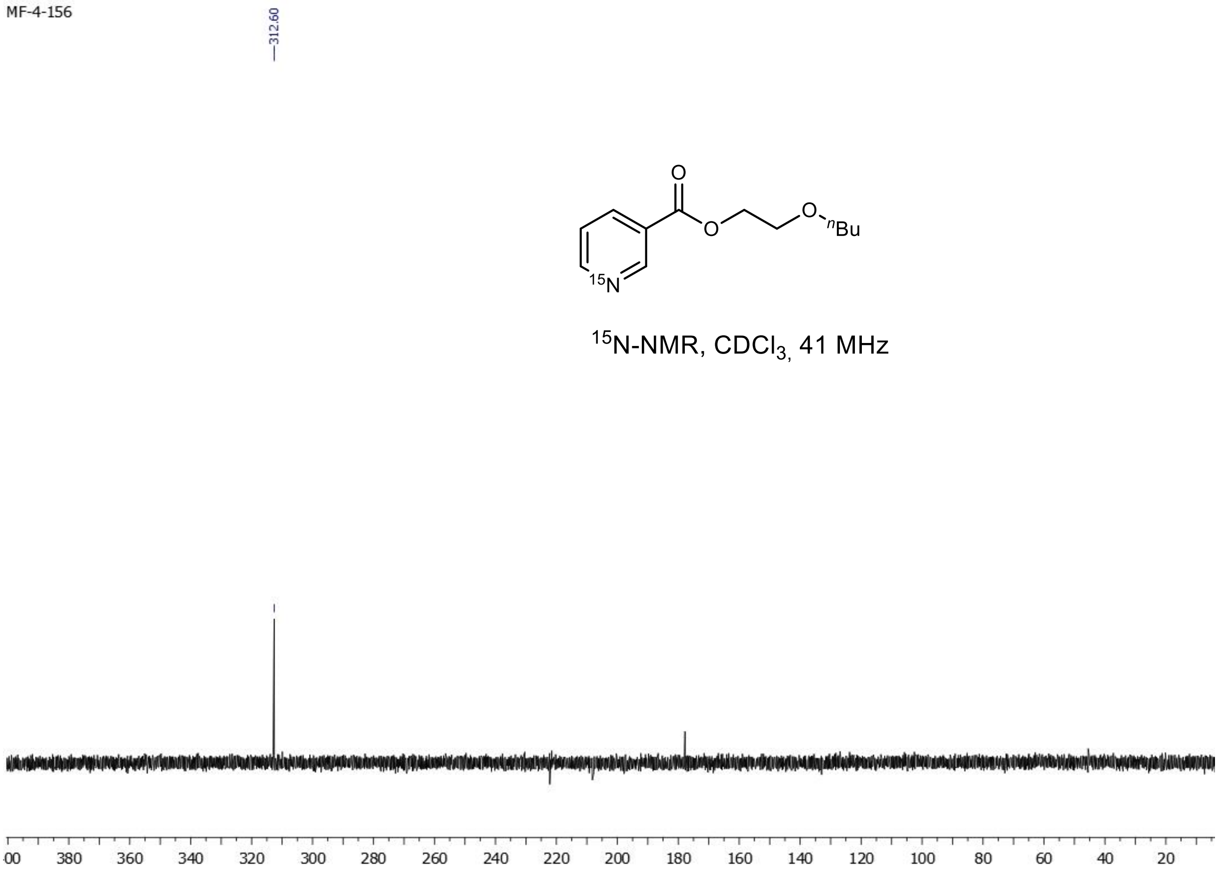
MF-4-156
312.60
O
O
O
nBu
15N
15N-NMR, CDCl3, 41 MHz
00 380 360 340 320 300 280 260 240 220 200 180 160 140 120 100 80 60 40 20

Isotopic Enrichment

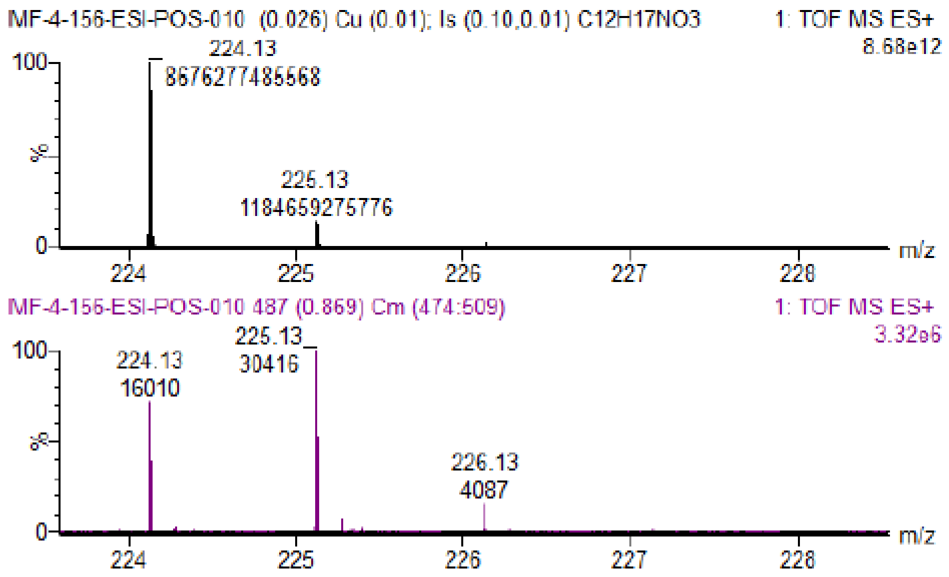

theorical isotopic distribution

	M	M+1	M+2	M+3	M+4	M+5	M+6	M+7
**m/z**	224	225	226	227	228	229	230	231
**%**	100,0	13,7	1,4	0,1	0,0	0,0	0,0	0,0

Enrichment calculation

Isotopomer	m/z	Area	natural isotope correction	Corrected area	Isotopic purity (%)
0	224	16010	0,00	16010,00	36,16
1	225	30416	2193,37	28222,63	63,74
2	226	4087	224,14	-3,64	-0,01
3	227	469	16,01	58,37	0,13
4	228	24	0,00	-12,17	-0,03
5	229	0	0,00	0,85	0,00
6	230	0	0,00	0,00	0,00
7	231	0	0,00	0,00	0,00
8	232	0	0,00	0,00	0,00
9	233	0	0,00	0,00	0,00
10	234	0	0,00	0,00	0,00
11	235	0	0,00	0,00	0,00
Total		51006		44276,04	100,00

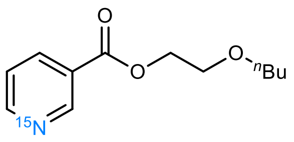

% Isotopic enrichment : 63,7

^{15}N-Nicotine ([^{15}N]35)

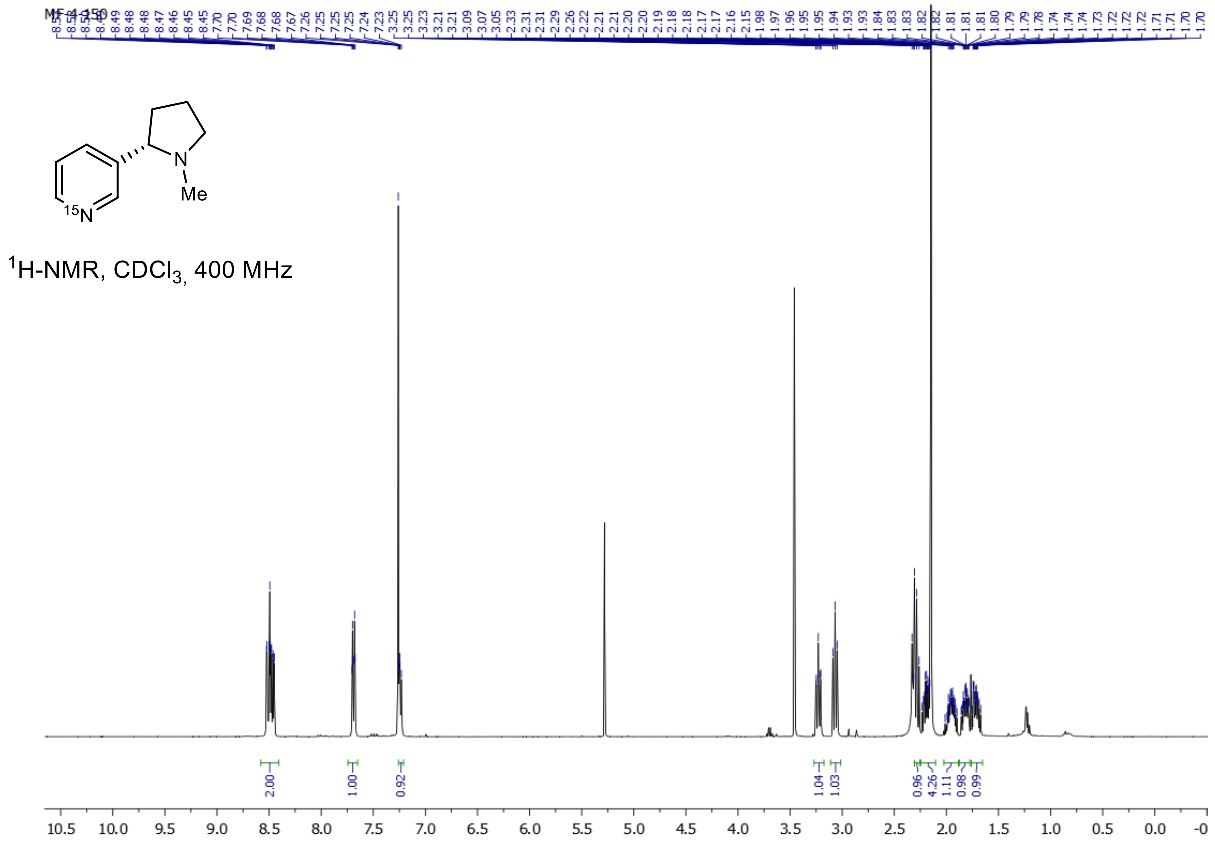

*Due to the volatility of the tiltel compound, the NMR spectra contains solvent peaks.

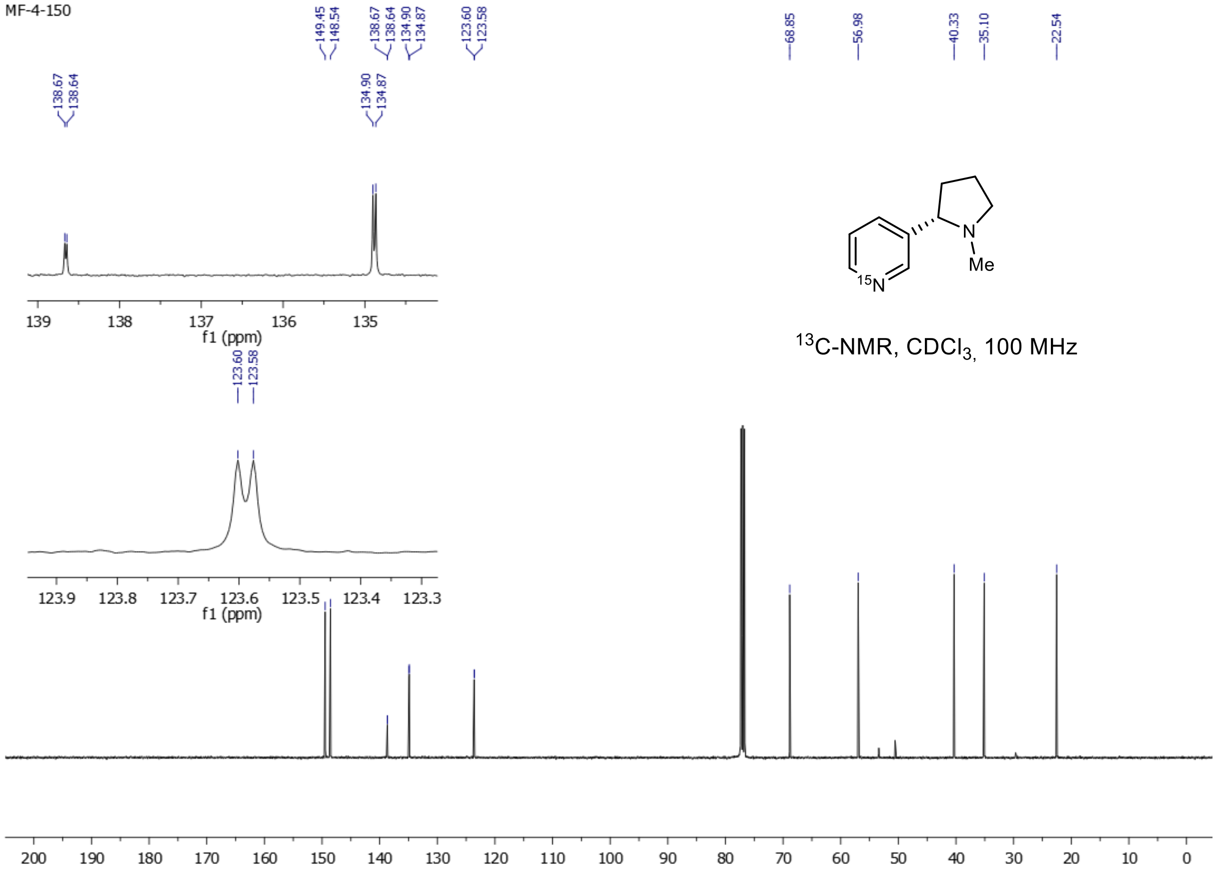

MF-4-150

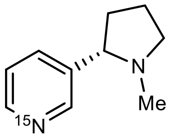

^{15}N-NMR, CDCl$_3$, 41 MHz

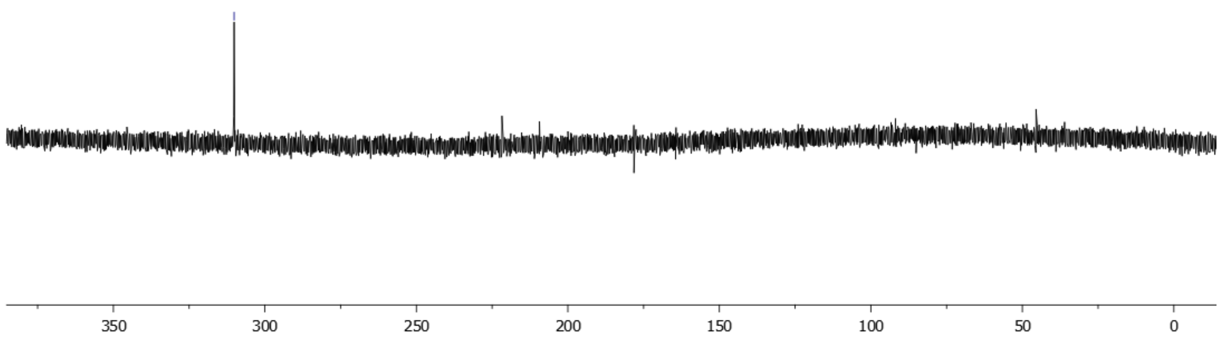

Isotopic Enrichment

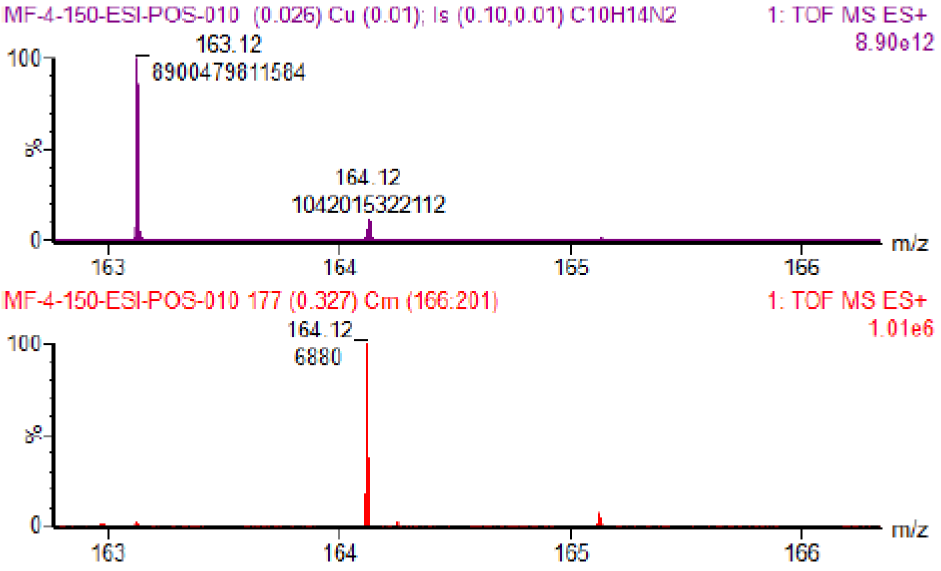

theorical isotopic distribution

	M	M+1	M+2	M+3	M+4	M+5	M+6	M+7
**m/z**	163	164	165	166	167	168	169	170
**%**	100,0	11,8	0,6	0,0	0,0	0,0	0,0	0,0

Enrichment calculation

Isotopomer	m/z	Area	natural isotope correction	Corrected area	Isotopic purity (%)
0	163	244	0,00	244,00	3,50
1	164	6880	28,79	6851,21	98,20
2	165	703	1,46	-106,91	-1,53
3	166	15	0,00	-13,49	-0,19
4	167	0	0,00	2,23	0,03
5	168	0	0,00	-0,18	0,00
6	169	0	0,00	0,01	0,00
7	170	0	0,00	0,00	0,00
8	171	0	0,00	0,00	0,00
9	172	0	0,00	0,00	0,00
10	173	0	0,00	0,00	0,00
11	174	0	0,00	0,00	0,00
Total		7842		6976,87	100,00

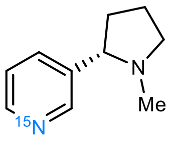

% Isotopic enrichment : 98,2

^{15}N-Nifenazone ([^{15}N]36)

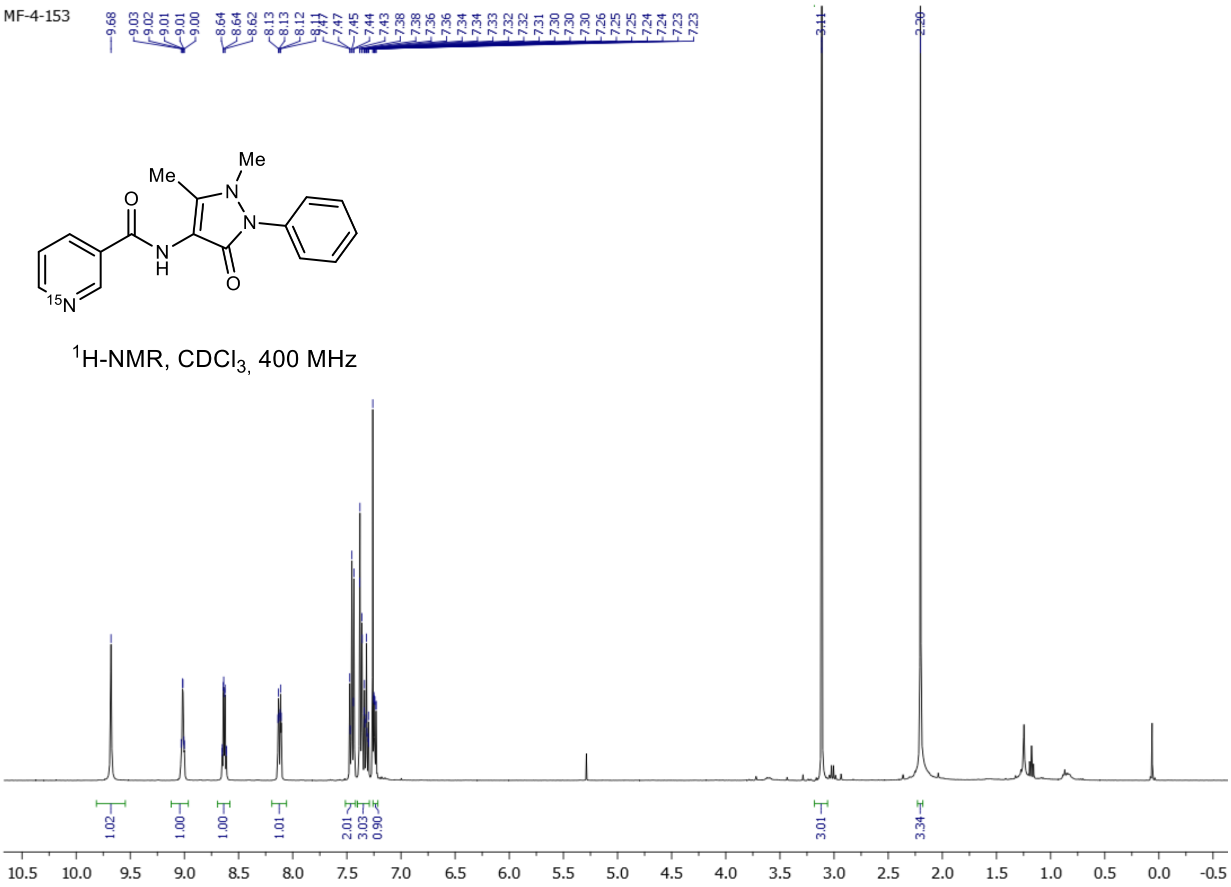

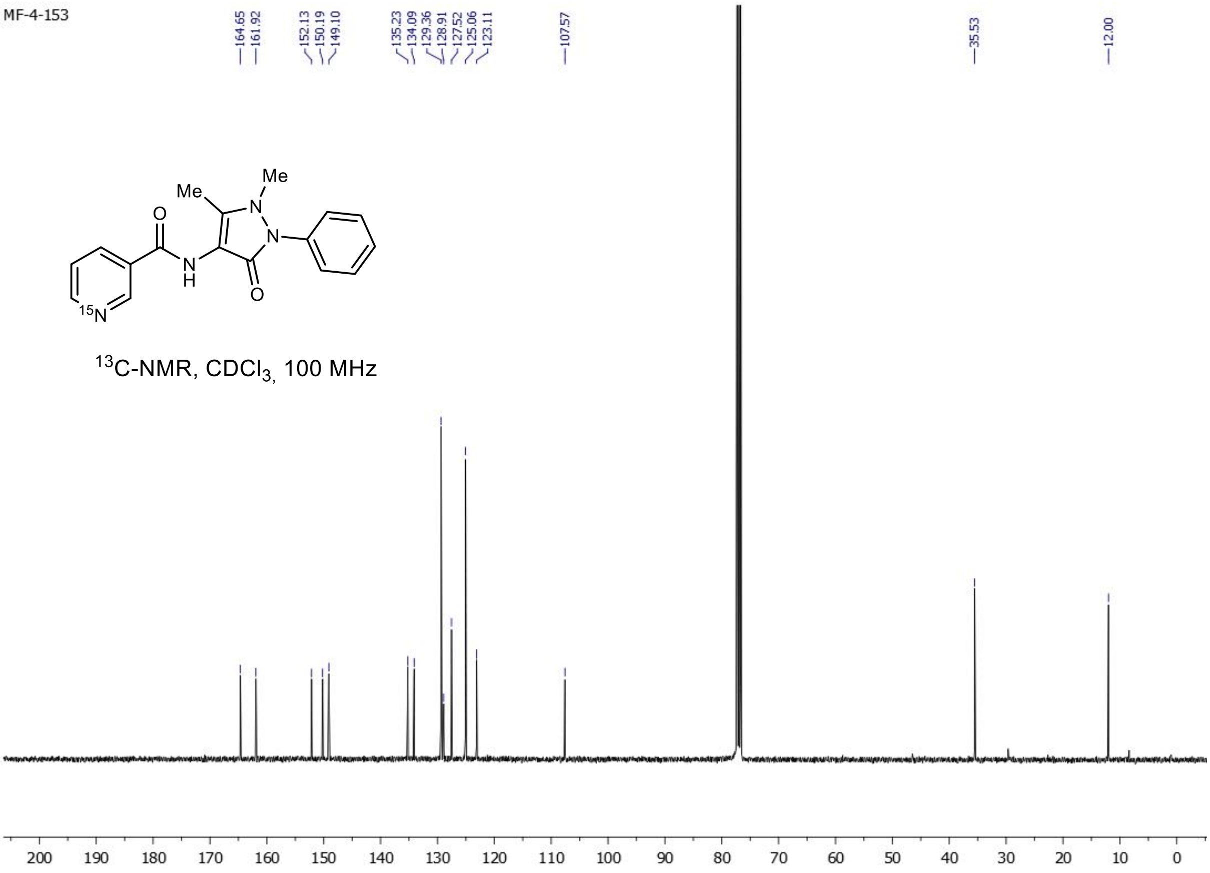

MF-4-153

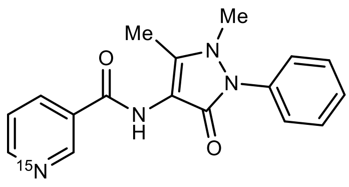

^{15}N-NMR, $CDCl_3$, 41 MHz

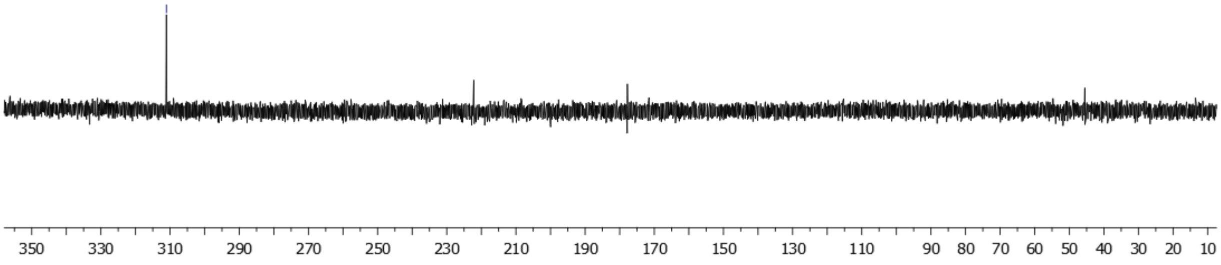

Isotopic Enrichment

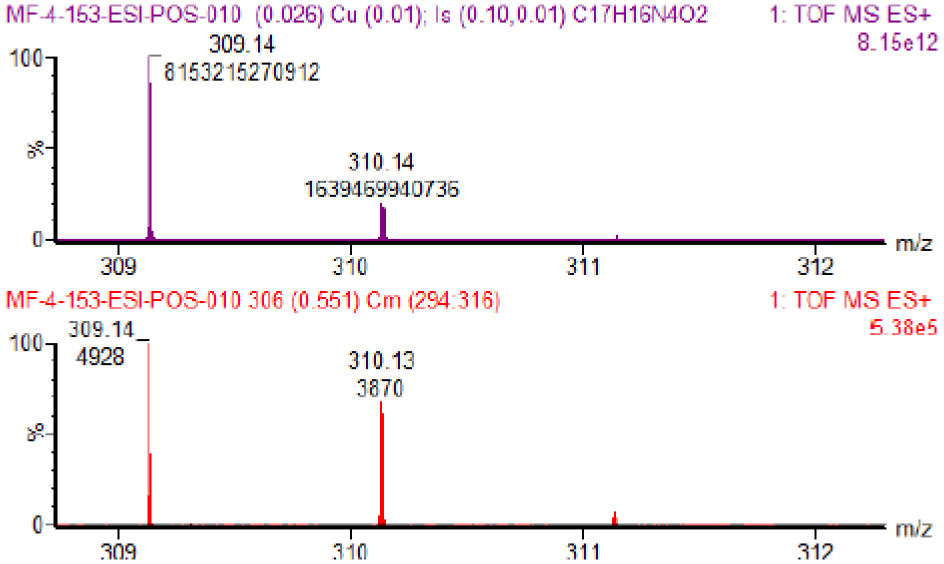

theorical isotopic distribution

	M	M+1	M+2	M+3	M+4	M+5	M+6	M+7
m/z	309	310	311	312	313	314	315	316
%	100,0	20,2	2,3	0,2	0,0	0,0	0,0	0,0

Enrichment calculation

Isotopomer	m/z	Area	natural isotope correction	Corrected area	Isotopic purity (%)
0	309	4928	0,00	4928,00	64,59
1	310	3870	995,46	2874,54	37,68
2	311	541	113,34	-153,00	-2,01
3	312	21	9,86	-24,06	-0,32
4	313	1	0,00	3,63	0,05
5	314	0	0,00	0,13	0,00
6	315	0	0,00	-0,06	0,00
7	316	0	0,00	0,00	0,00
8	317	0	0,00	0,00	0,00
9	318	0	0,00	0,00	0,00
10	319	0	0,00	0,00	0,00
11	320	0	0,00	0,00	0,00
Total		9361		7629,18	100,00

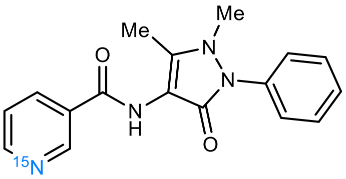

% Isotopic enrichment : 37,7

^{15}N-Bisacodyl ([^{15}N]37)

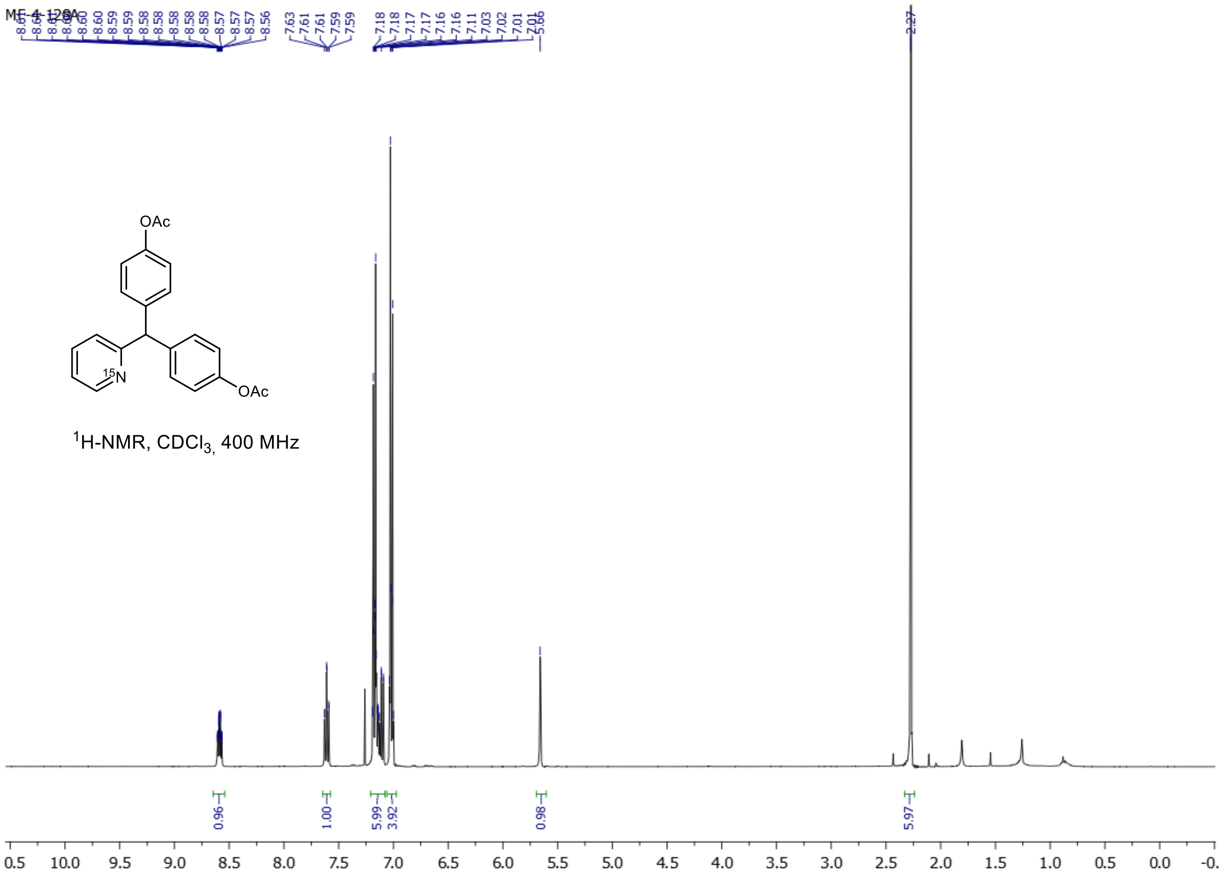

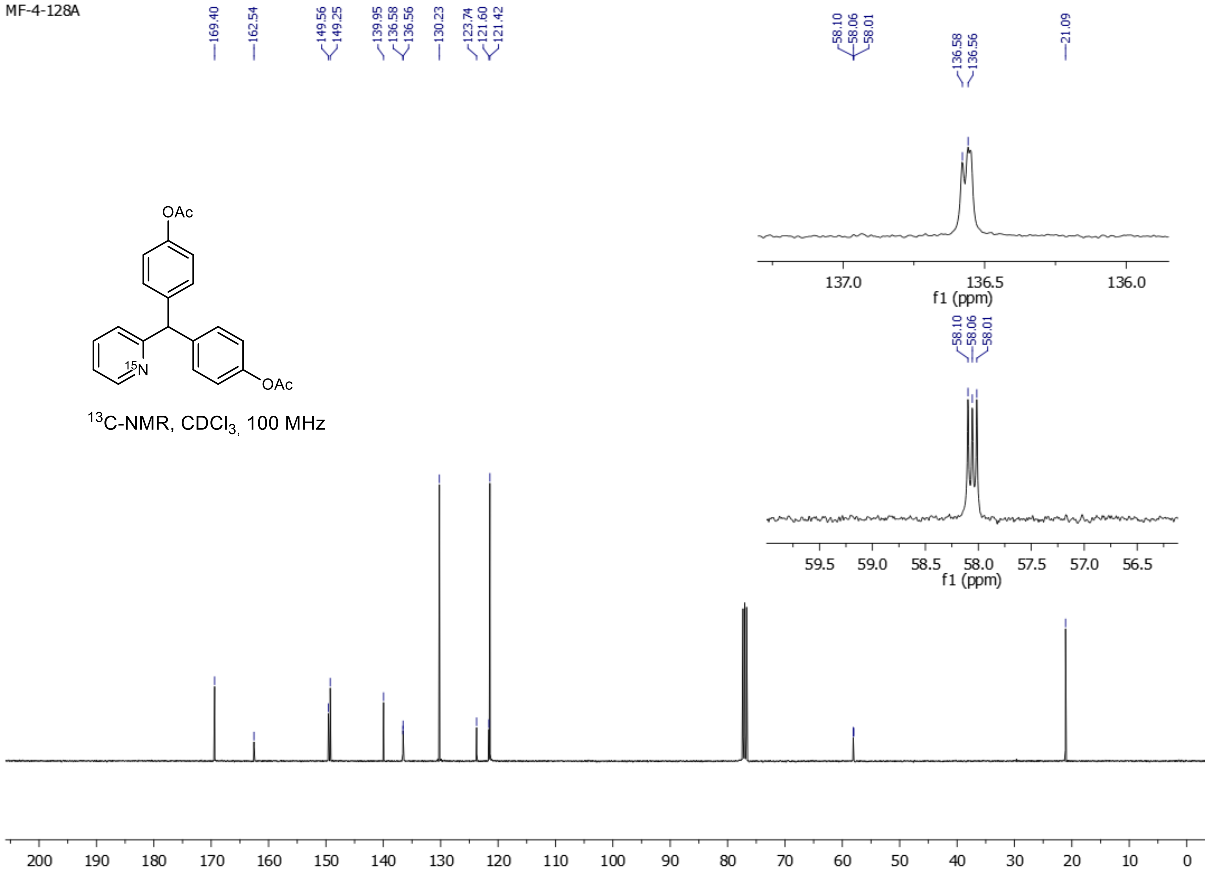

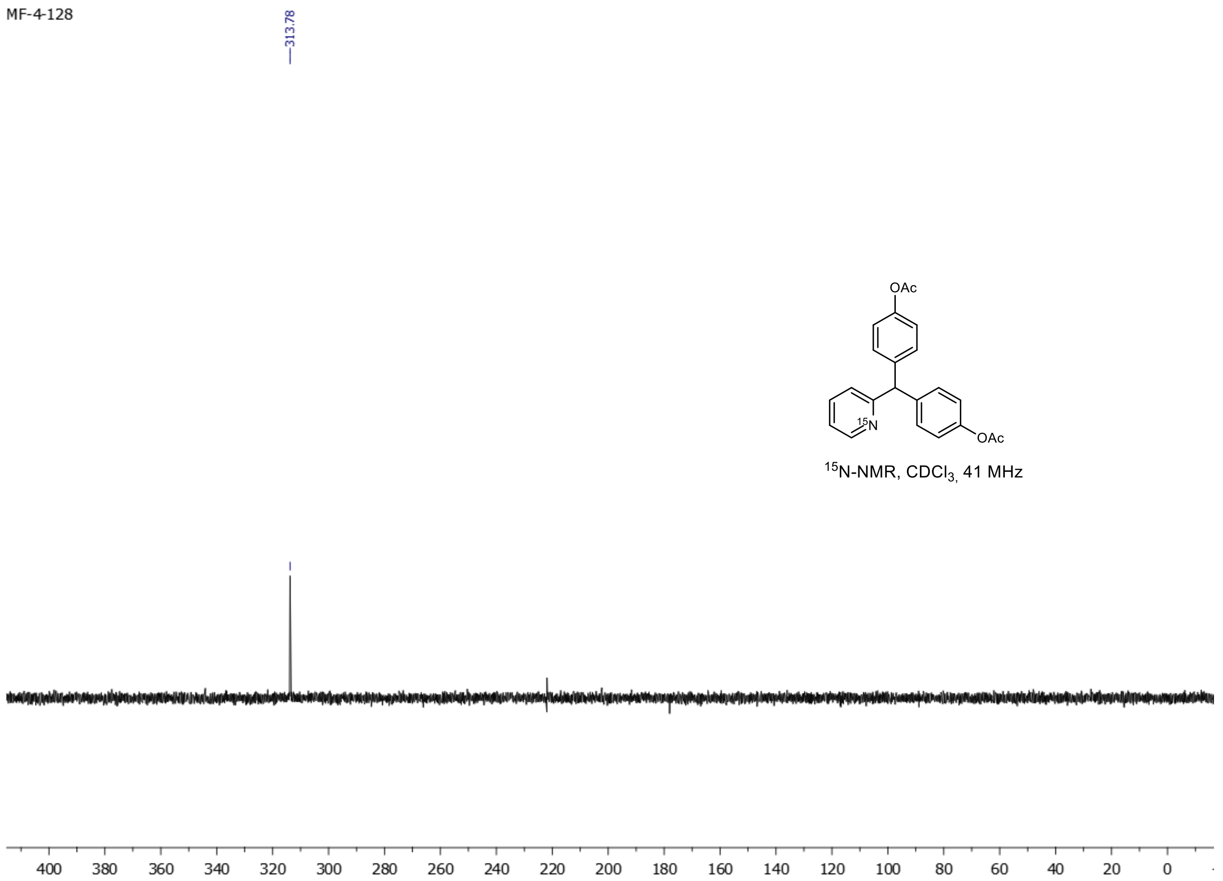
MF-4-128
313.78
OAc
15N
OAc
^{15}N-NMR, $CDCl_3$, 41 MHz
400 380 360 340 320 300 280 260 240 220 200 180 160 140 120 100 80 60 40 20 0

Isotopic Enrichment

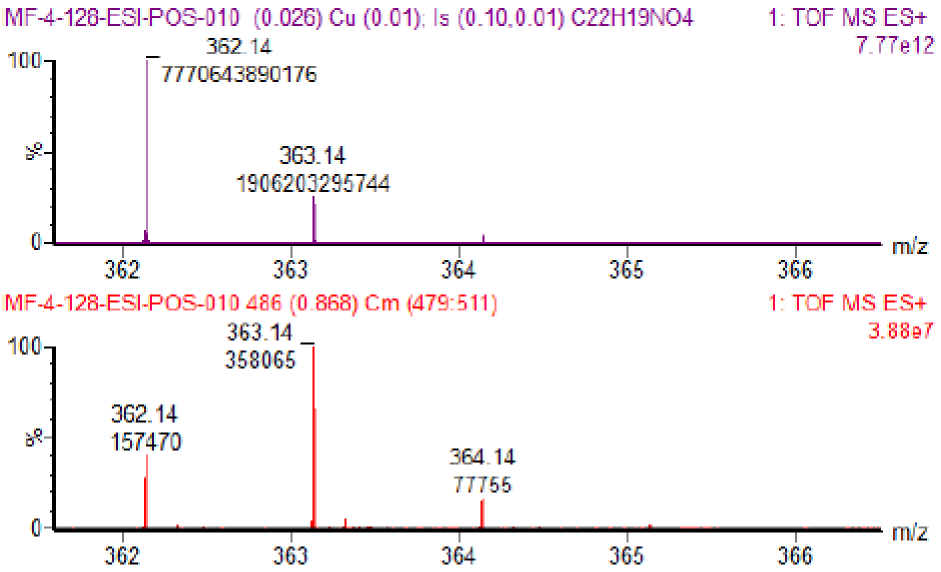

theorical isotopic distribution

	M	M+1	M+2	M+3	M+4	M+5	M+6	M+7
**m/z**	362	363	364	365	366	367	368	369
**%**	100,0	24,5	3,6	0,4	0,0	0,0	0,0	0,0

Enrichment calculation

Isotopomer	m/z	Area	natural isotope correction	Corrected area	Isotopic purity (%)
0	362	157470	0,00	157470,00	33,71
1	363	358065	38580,15	319484,85	68,40
2	364	77755	5668,92	-6187,71	-1,32
3	365	6723	629,88	-3892,35	-0,83
4	366	208	0,00	106,44	0,02
5	367	0	0,00	138,80	0,03
6	368	0	0,00	-22,27	0,00
7	369	0	0,00	0,03	0,00
8	370	0	0,00	0,24	0,00
9	371	0	0,00	0,03	0,00
10	372	0	0,00	-0,02	0,00
11	373	0	0,00	0,00	0,00
Total		600221		467098,05	100,00

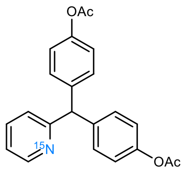

% Isotopic enrichment : 68,4

^{15}*N-Vismodegib ([^{15}N]38)*

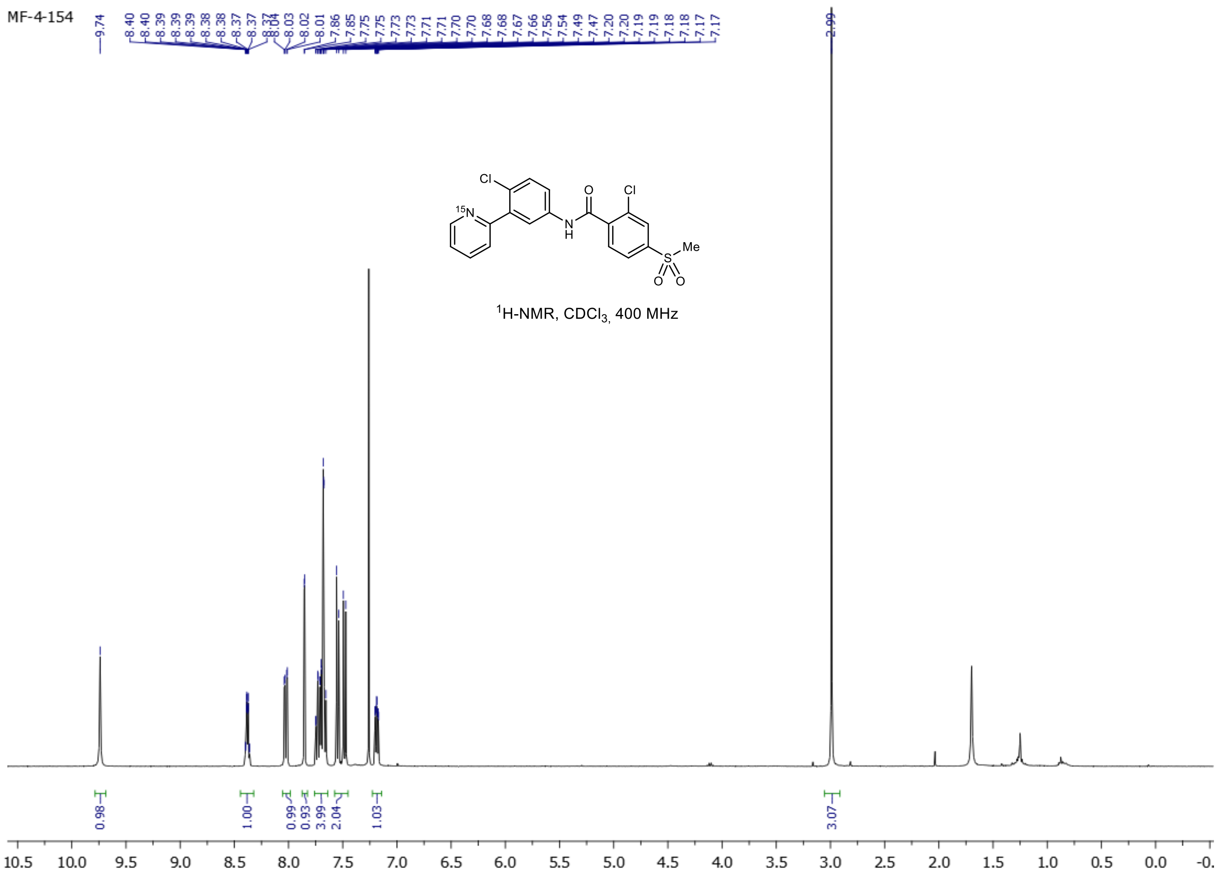

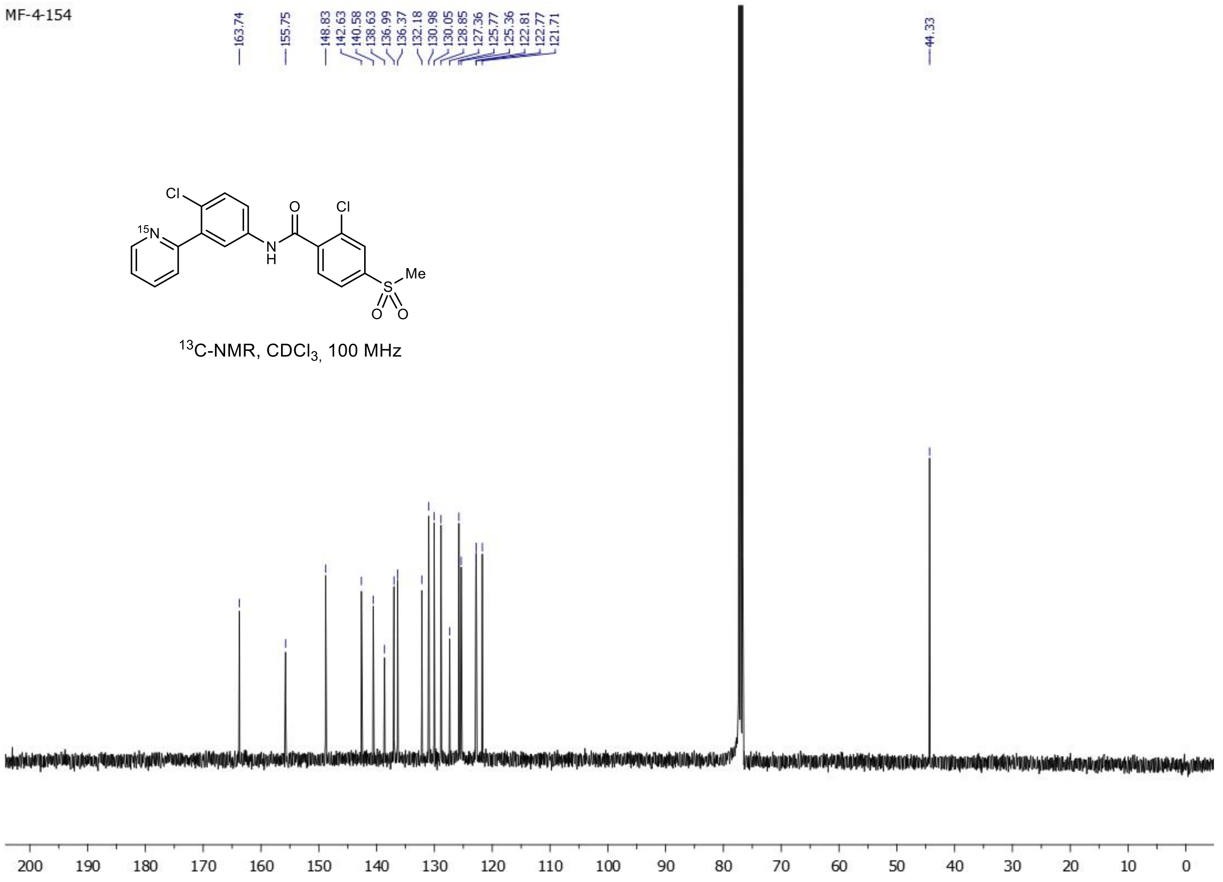

MF-4-154

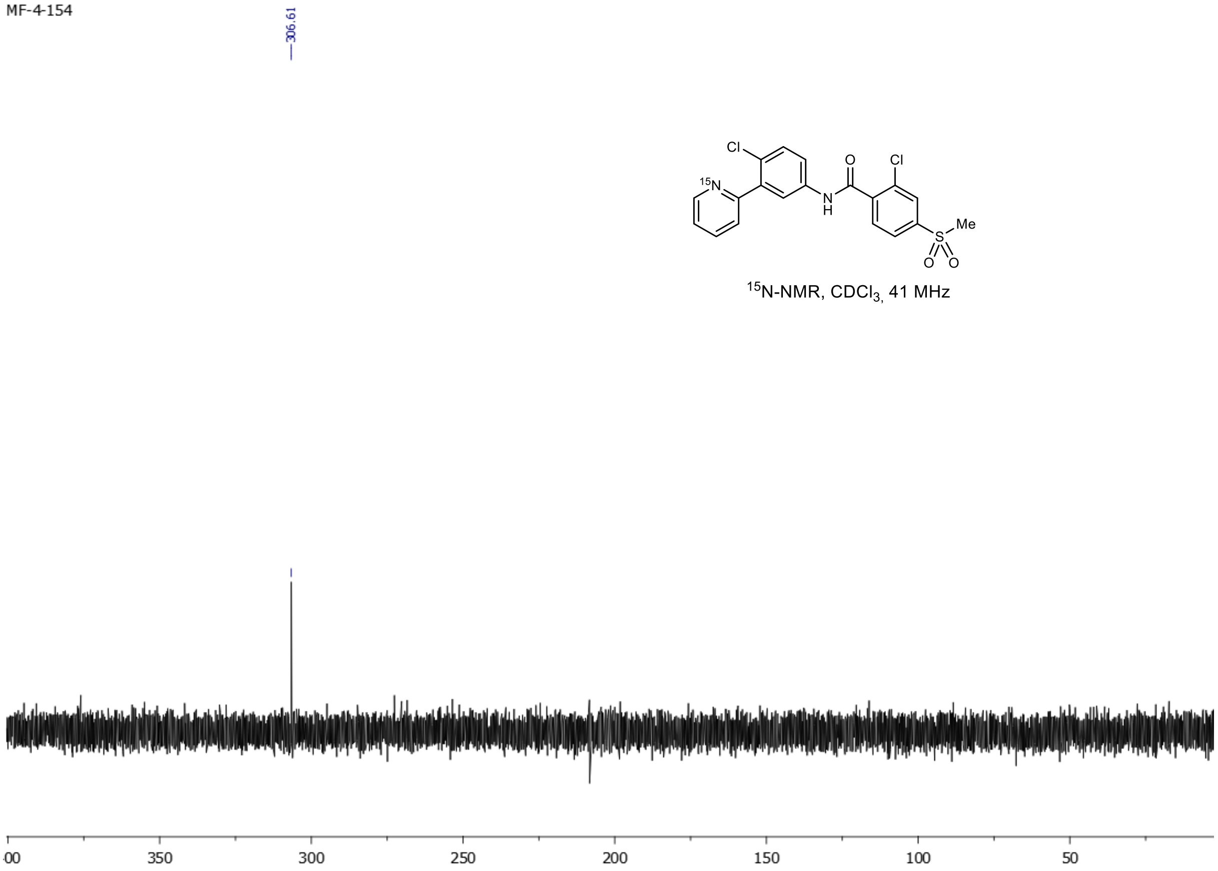

306.61
Cl
15N
N
H
O
Cl
S
Me
O
O
15N-NMR, CDCl3, 41 MHz
00
350
300
250
200
150
100
50

Isotopic Enrichment

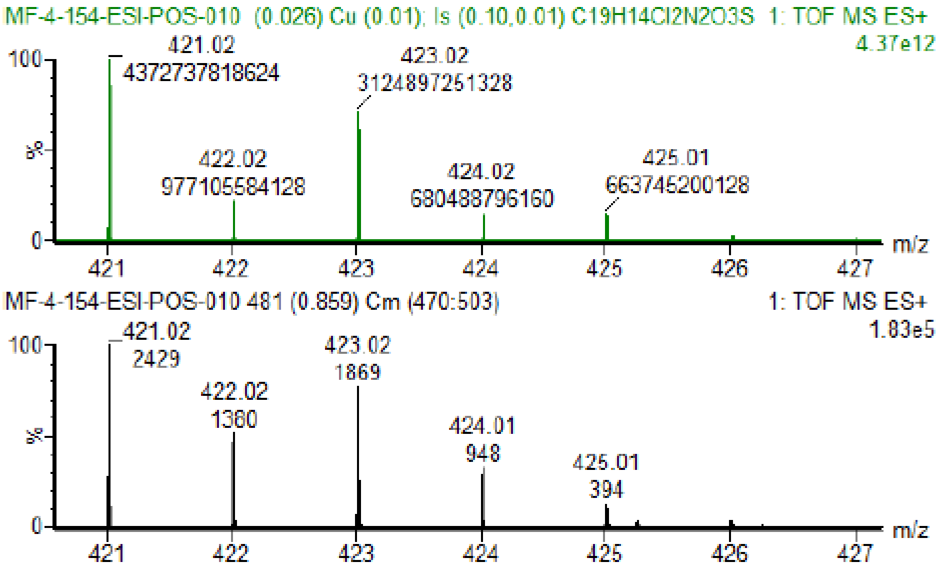

theorical isotopic distribution

	M	M+1	M+2	M+3	M+4	M+5	M+6	M+7
**m/z**	421	422	423	424	425	426	427	428
**%**	100,0	22,4	71,9	15,6	15,2	3,0	0,7	0,1

Enrichment calculation

Isotopomer	m/z	Area	natural isotope correction	Corrected area	Isotopic purity (%)
0	421	2429	0,00	2429,00	77,77
1	422	1380	544,10	835,90	26,76
2	423	1869	1746,46	-64,69	-2,07
3	424	948	378,93	-17,45	-0,56
4	425	394	369,21	-55,19	-1,77
5	426	114	72,87	-50,93	-1,63
6	427	15	17,00	36,56	1,17
7	428	0	2,43	33,35	1,07
8	429	0	0,00	-17,29	-0,55
9	430	0	0,00	-16,22	-0,52
10	431	0	0,00	7,24	0,23
11	432	0	0,00	6,98	0,22
Total		7149		3127,27	100,13

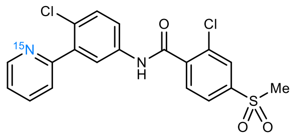

% Isotopic enrichment : 26,8

^{15}N-Loratadine ([^{15}N]39)

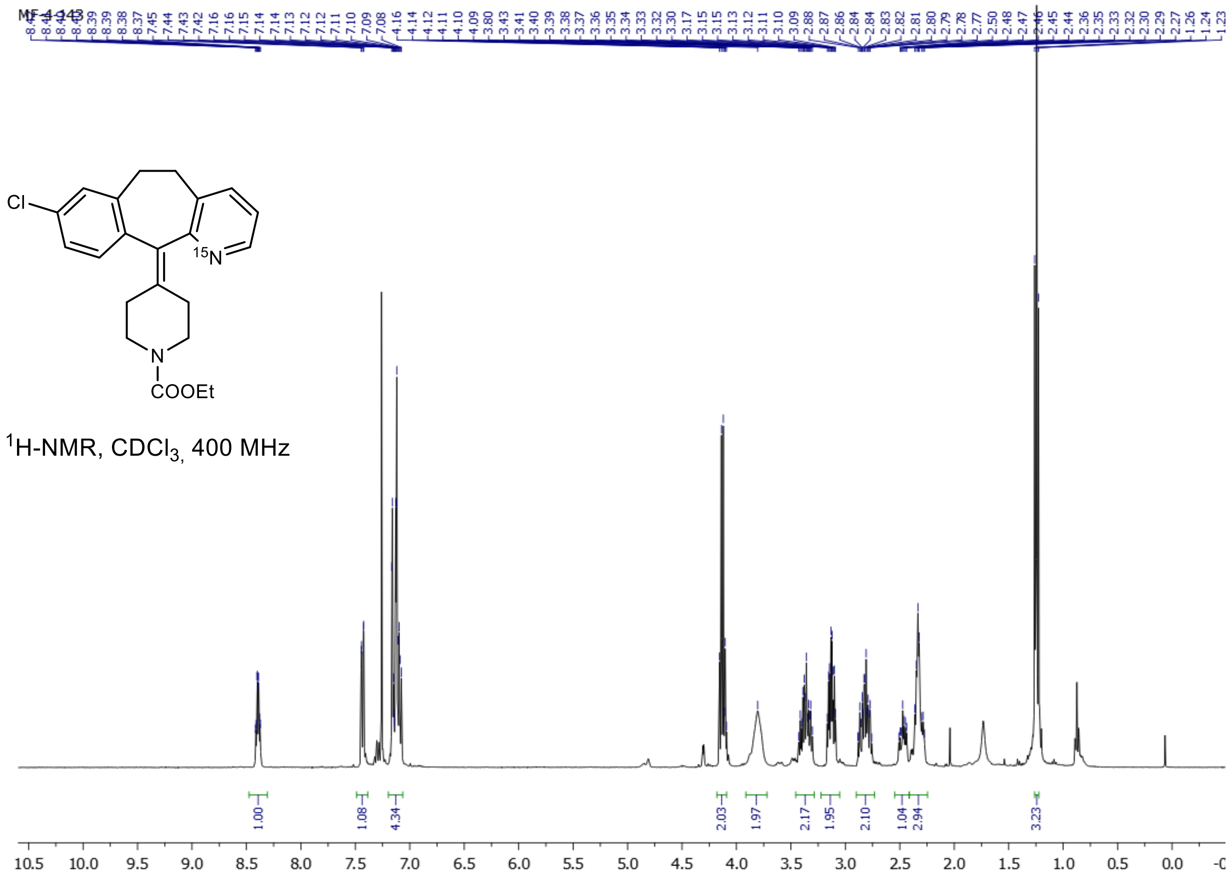

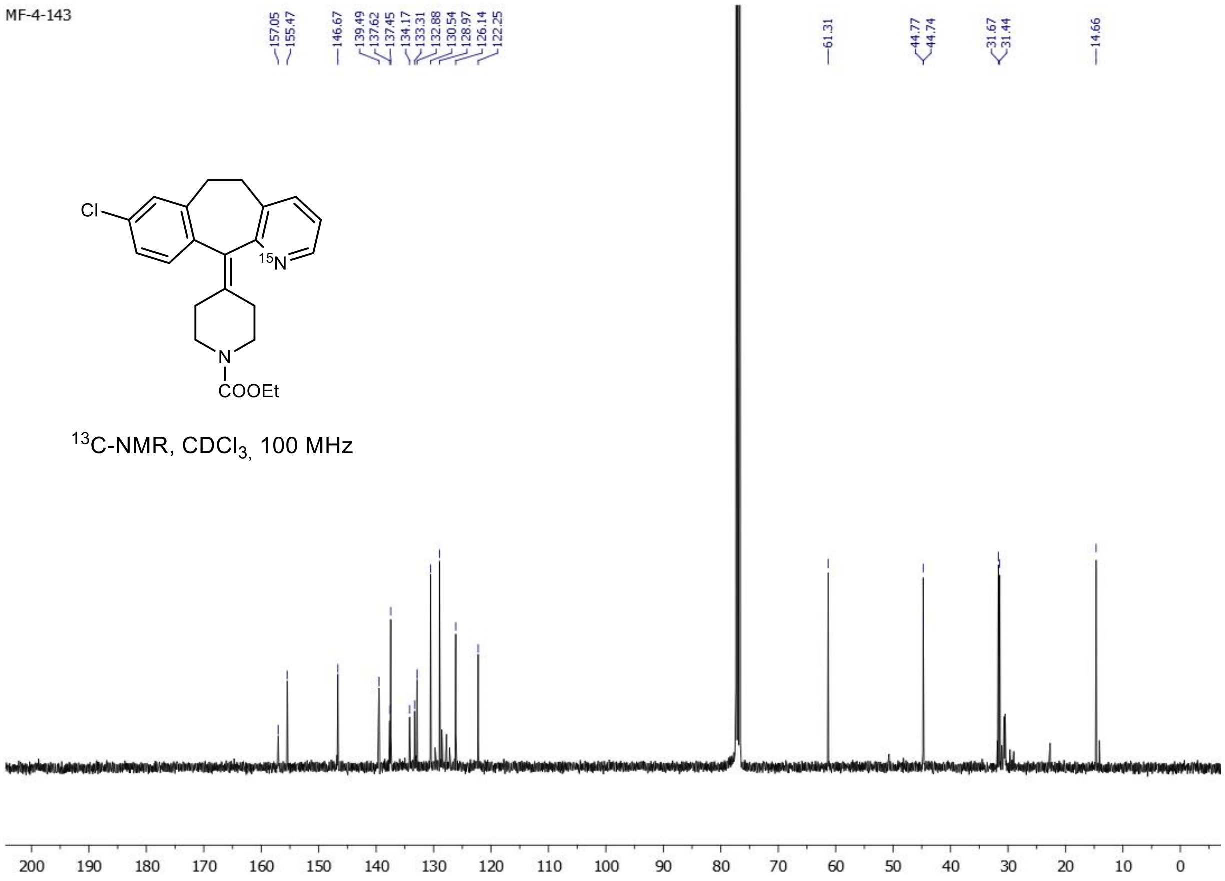

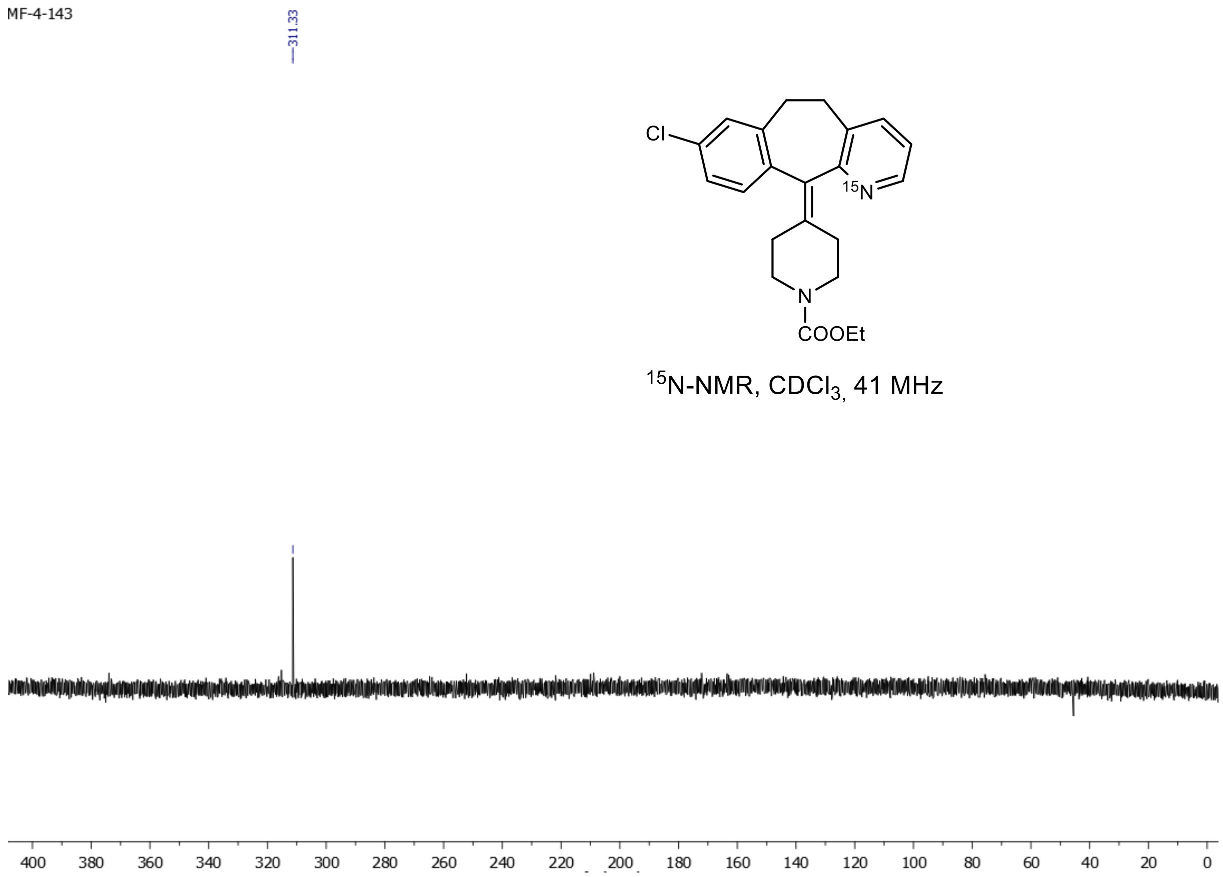

^{15}N-NMR, $CDCl_3$, 41 MHz

Isotopic Enrichment

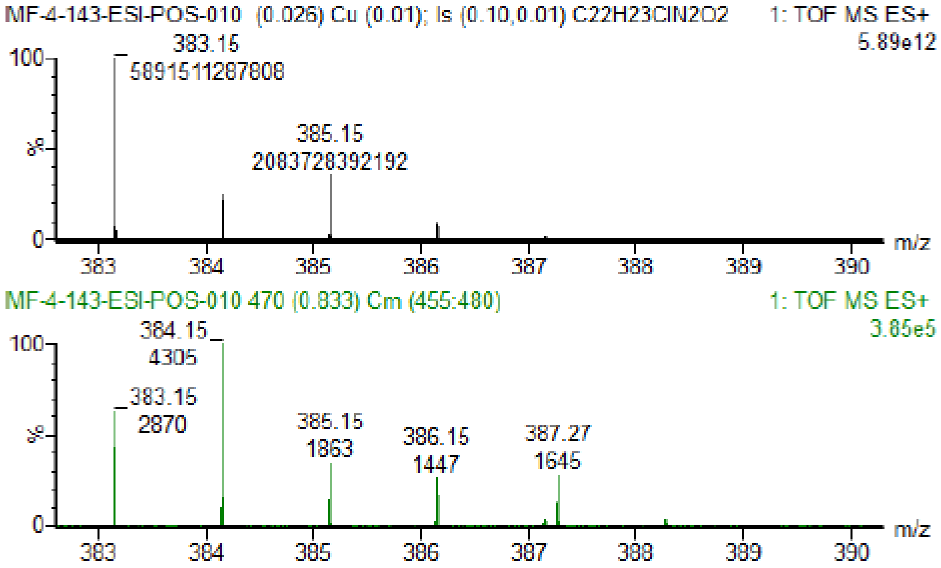

theorical isotopic distribution

	M	M+1	M+2	M+3	M+4	M+5	M+6	M+7
m/z	383	384	385	386	387	388	389	390
%	100,0	25,0	35,8	8,4	1,1	0,1	0,0	0,0

Enrichment calculation

Isotopomer	m/z	Area	natural isotope correction	Corrected area	Isotopic purity (%)
0	383	2870	0,00	2870,00	45,57
1	384	4305	717,50	3587,50	56,96
2	385	1863	1027,46	-61,34	-0,97
3	386	1447	241,08	-63,07	-1,00
4	387	231	31,57	-64,19	-1,02
5	388	16	2,87	17,45	0,28
6	389	0	0,00	21,00	0,33
7	390	0	0,00	-5,35	-0,08
8	391	0	0,00	-6,88	-0,11
9	392	0	0,00	1,74	0,03
10	393	0	0,00	2,23	0,04
11	394	0	0,00	-0,57	-0,01
Total		10732		6298,53	100,01

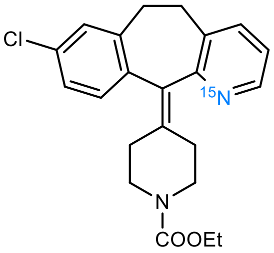

% Isotopic enrichment : 57,0

^{15}N-Etoricoxib ([^{15}N]40)

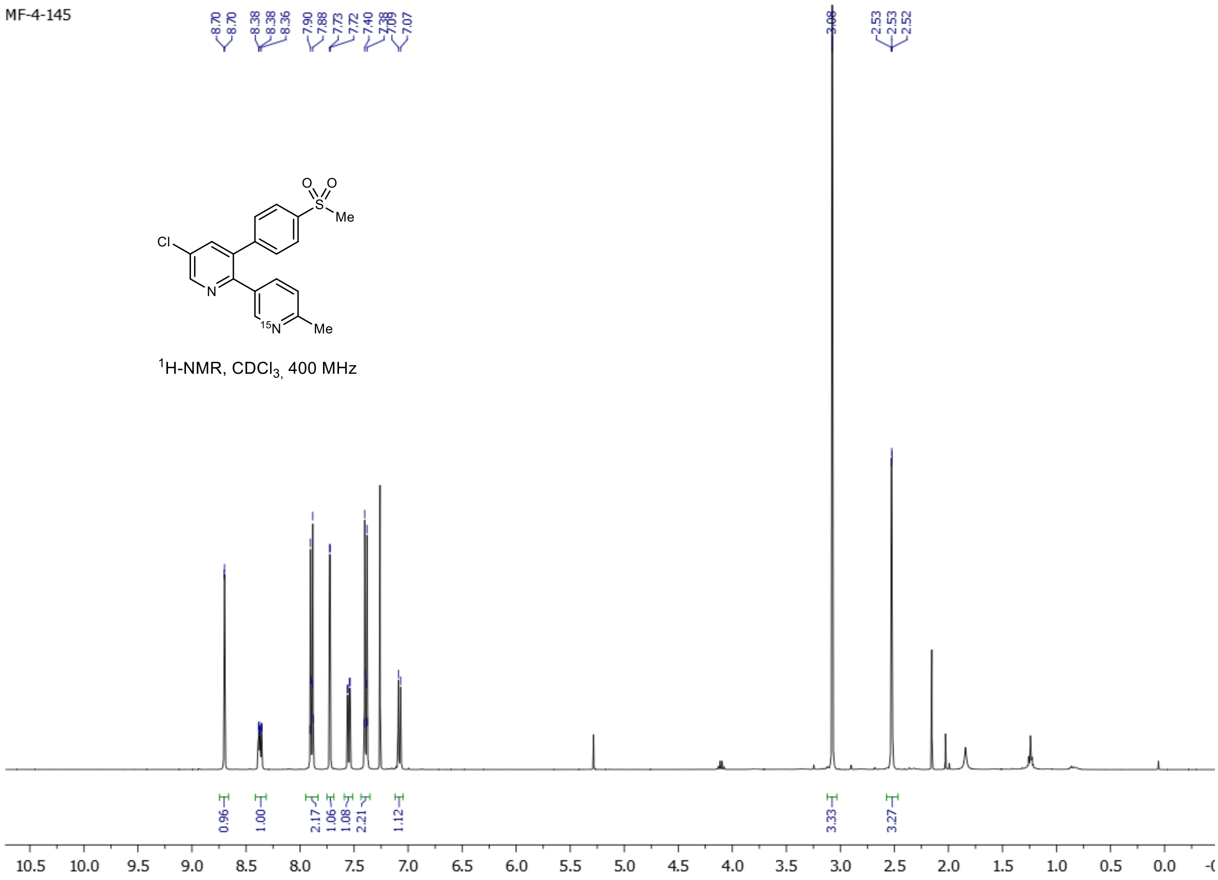

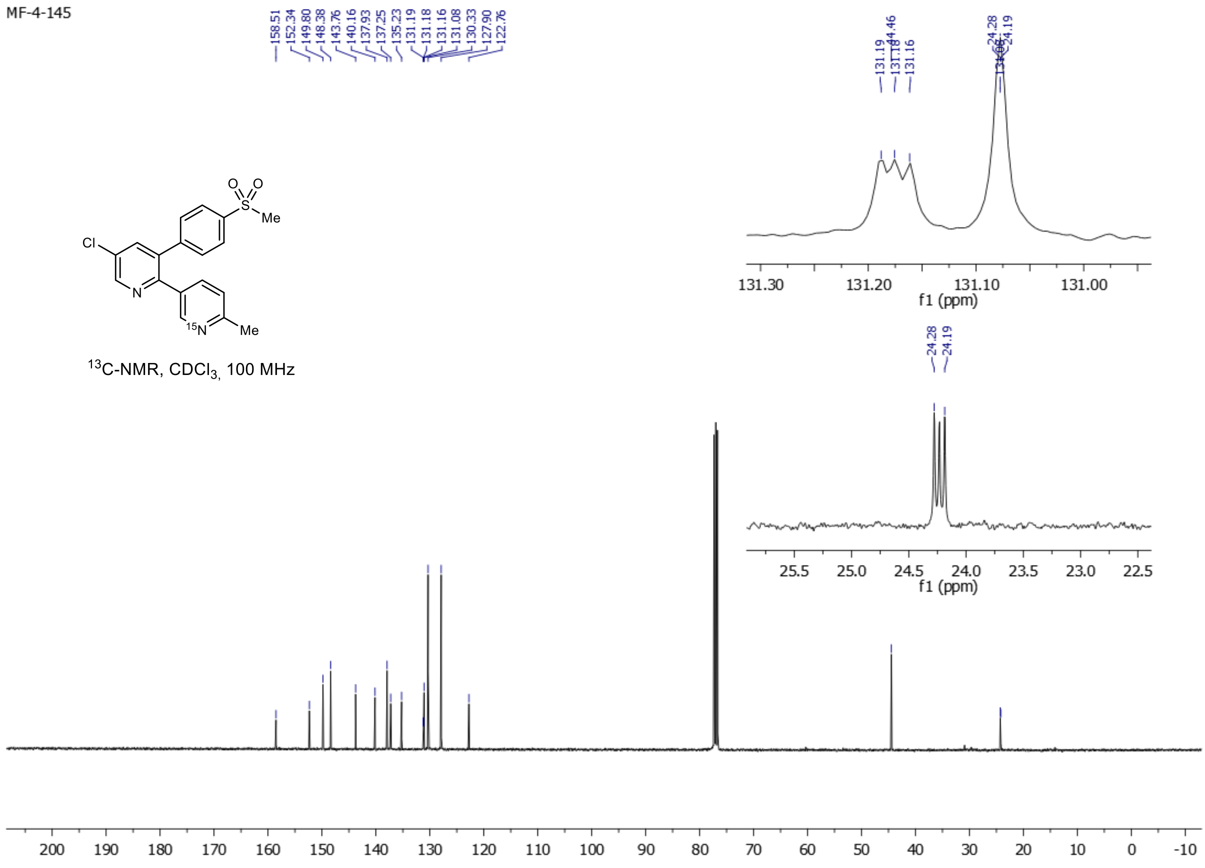

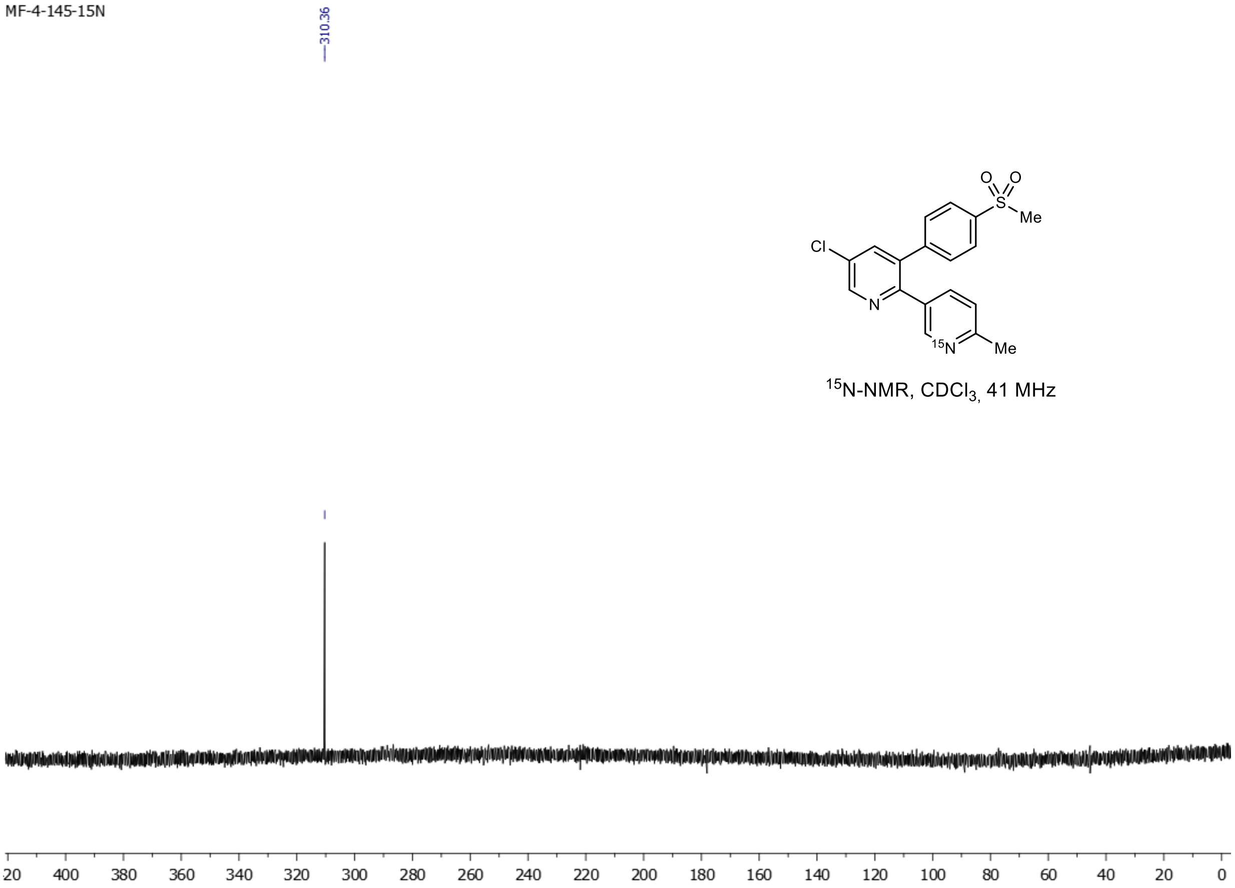
MF-4-145-15N
310.36
Cl
N
^{15}N
Me
O
O
S
Me
^{15}N-NMR, CDCl$_3$, 41 MHz
20
400
380
360
340
320
300
280
260
240
220
200
180
160
140
120
100
80
60
40
20
0

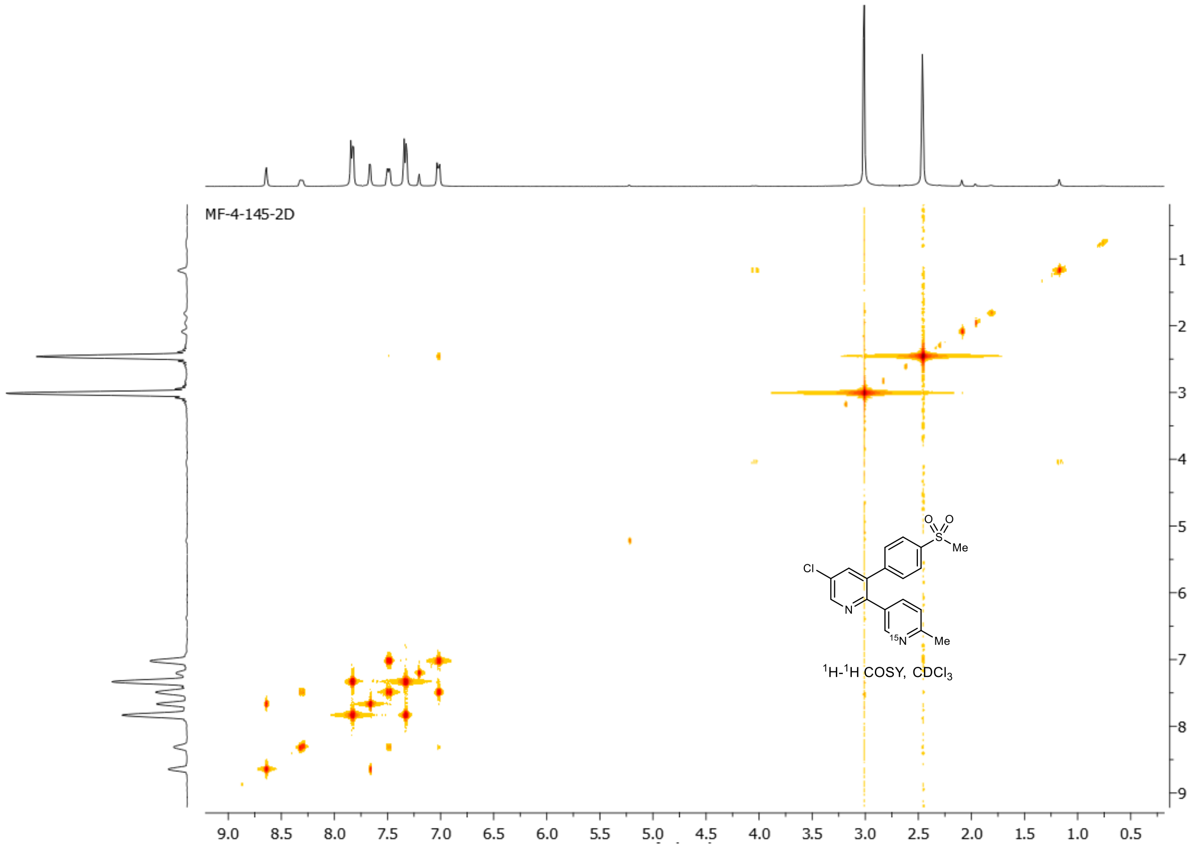
MF-4-145-2D
Cl
N
^{15}N
Me
O
O
S
Me
^{1}H-^{1}H COSY, CDCl$_3$
9.0
8.5
8.0
7.5
7.0
6.5
6.0
5.5
5.0
4.5
4.0
3.5
3.0
2.5
2.0
1.5
1.0
0.5

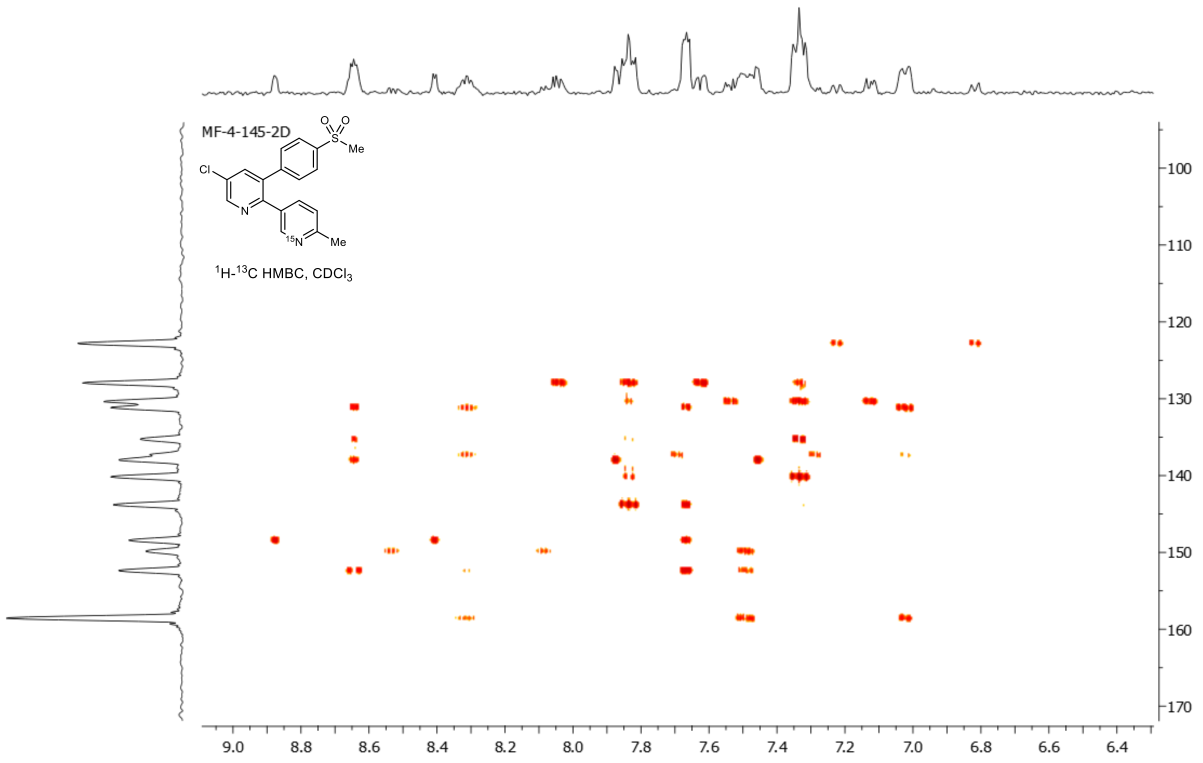
MF-4-145-2D
Cl
N
15N
Me
O
O
S
Me
1H-13C HMBC, CDCl3
9.0 8.8 8.6 8.4 8.2 8.0 7.8 7.6 7.4 7.2 7.0 6.8 6.6 6.4
100 110 120 130 140 150 160 170

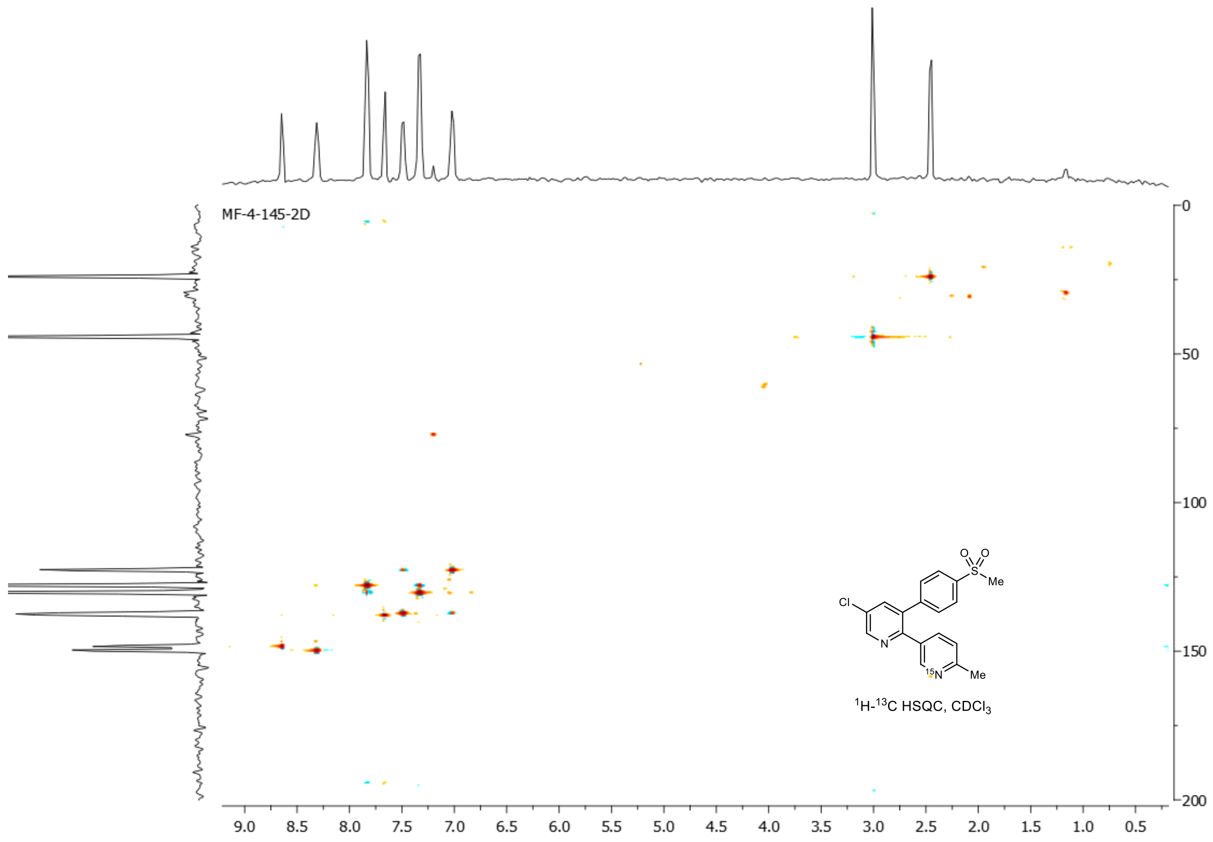
MF-4-145-2D
Cl
N
15N
Me
O
O
S
Me
1H-13C HSQC, CDCl3
9.0 8.5 8.0 7.5 7.0 6.5 6.0 5.5 5.0 4.5 4.0 3.5 3.0 2.5 2.0 1.5 1.0 0.5
0 50 100 150 200

Isotopic Enrichment

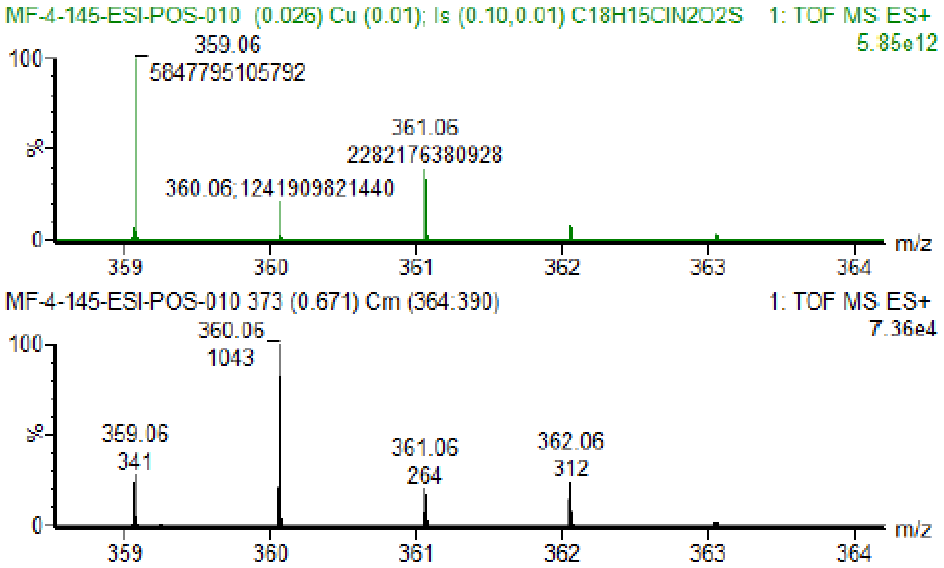

theorical isotopic distribution

	M	M+1	M+2	M+3	M+4	M+5	M+6	M+7
m/z	359	360	361	362	363	364	365	366
%	100,0	21,3	39,3	7,9	2,2	0,3	0,0	0,0

Enrichment calculation

Isotopomer	m/z	Area	natural isotope correction	Corrected area	Isotopic purity (%)
0	359	341	0,00	341,00	29,21
1	360	1043	72,63	970,37	83,13
2	361	264	134,01	-76,70	-6,57
3	362	312	26,94	-79,96	-6,85
4	363	32	7,50	-4,99	-0,43
5	364	4	1,02	20,17	1,73
6	365	0	0,00	2,76	0,24
7	366	0	0,00	-6,13	-0,53
8	367	0	0,00	-1,02	-0,09
9	368	0	0,00	1,98	0,17
10	369	0	0,00	0,34	0,03
11	370	0	0,00	-0,64	-0,06
Total		1996		1167,18	99,99

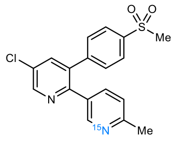

% Isotopic enrichment : 83,1

[15]N-Metyrapone ([^{15}N]41)

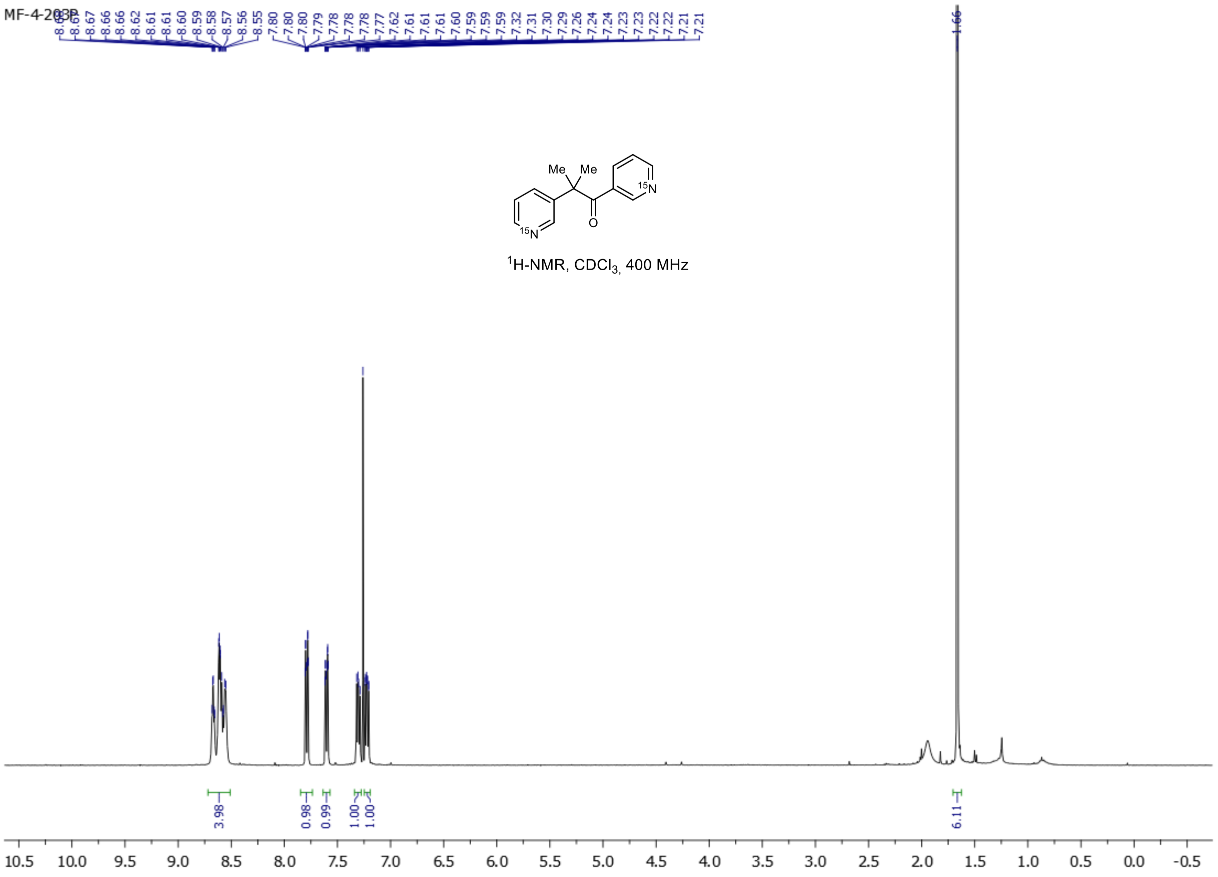

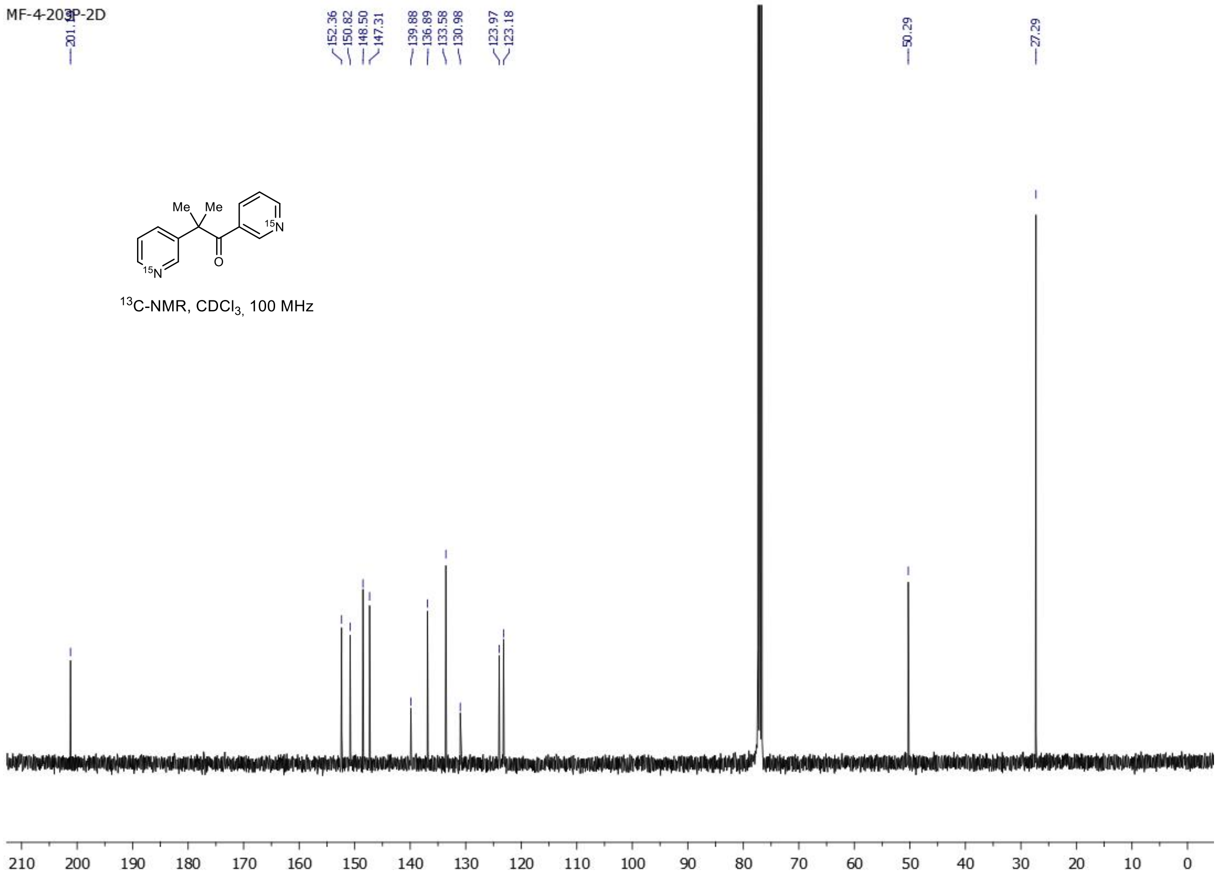

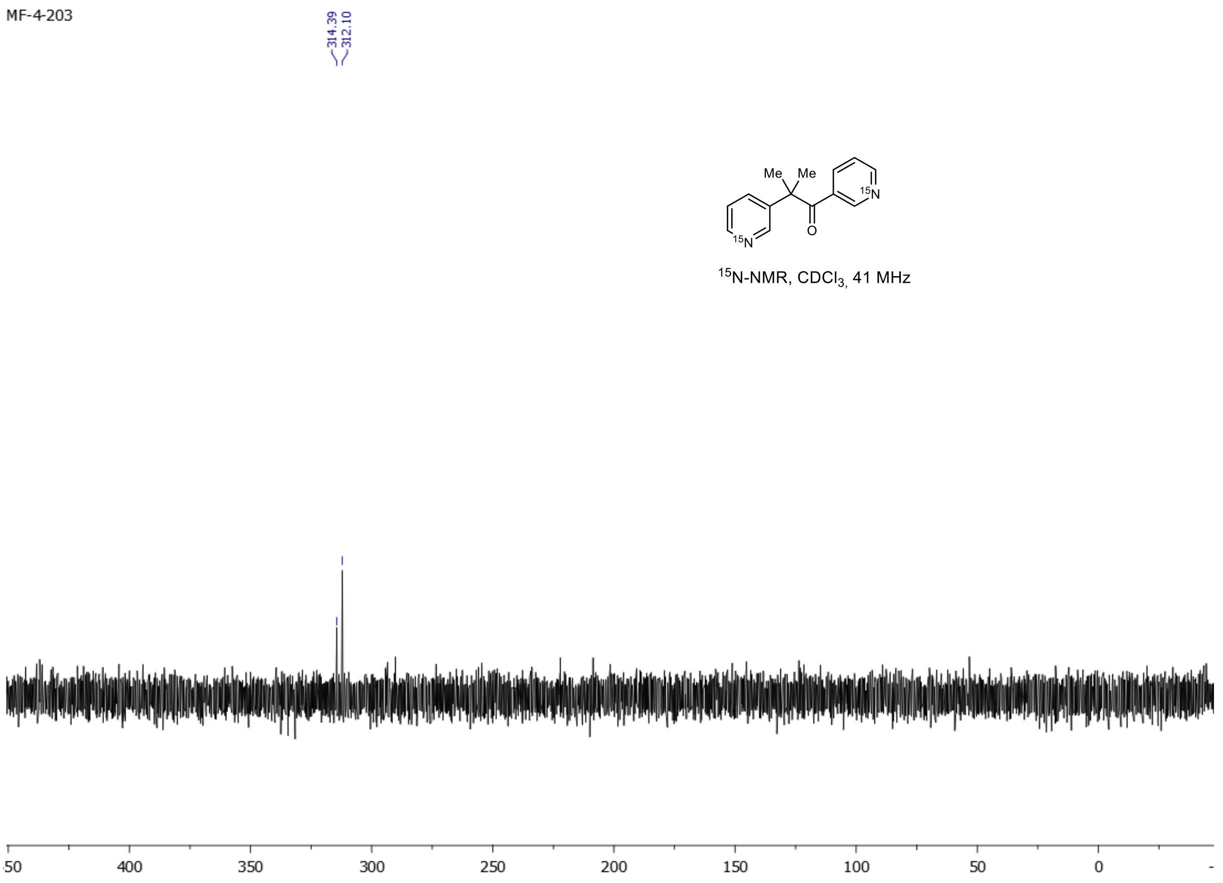
MF-4-203
314.39
312.10
Me
Me
15N
O
15N
15N-NMR, CDCl3, 41 MHz
50
400
350
300
250
200
150
100
50
0

Isotopic Enrichment

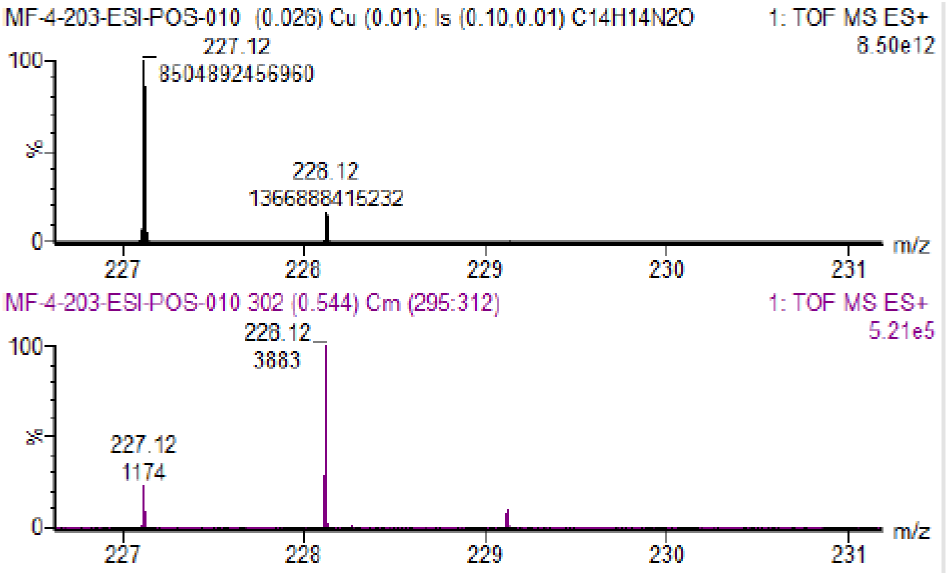

theorical isotopic distribution

	M	M+1	M+2	M+3	M+4	M+5	M+6	M+7
**m/z**	227	228	229	230	231	232	233	234
**%**	100,0	16,1	1,4	0,0	0,0	0,0	0,0	0,0

Enrichment calculation

Isotopomer	m/z	Area	natural isotope correction	Corrected area	Isotopic purity (%)
0	227	1174	0,00	1174,00	24,41
1	228	3883	189,01	3693,99	76,81
2	229	572	16,44	-39,17	-0,81
3	230	18	0,00	-27,41	-0,57
4	231	4	0,00	8,96	0,19
5	232	0	0,00	-1,06	-0,02
6	233	0	0,00	0,05	0,00
7	234	0	0,00	0,01	0,00
8	235	0	0,00	0,00	0,00
9	236	0	0,00	0,00	0,00
10	237	0	0,00	0,00	0,00
11	238	0	0,00	0,00	0,00
Total		5651		4809,36	100,00

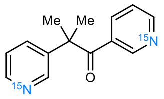

% Isotopic enrichment : 76,8

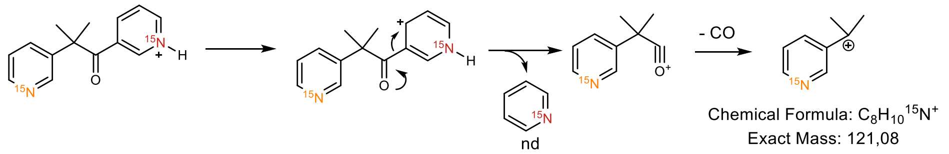

isotopic Enrichment (fragment 1)

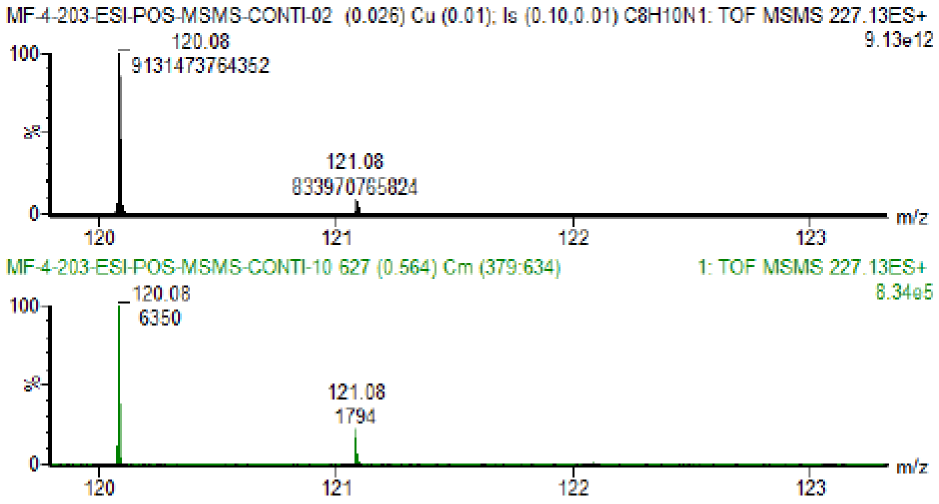

theorical isotopic distribution

	M	M+1	M+2	M+3	M+4	M+5	M+6	M+7
m/z	120	121	122	123	124	125	126	127
%	100	9,2	0,3	0	0	0	0	0

Enrichment calculation

Isotopomer	m/z	Area	natural isotope correction	Corrected area	Isotopic purity (%)
0	120	6350	0,00	6350,00	84,82
1	121	1794	584,20	1209,80	16,16
2	122	53	19,05	-77,35	-1,03
3	123	1	0,00	4,49	0,06
4	124	0	0,00	-0,18	0,00
5	125	0	0,00	0,00	0,00
6	126	0	0,00	0,00	0,00
7	127	0	0,00	0,00	0,00
8	128	0	0,00	0,00	0,00
9	129	0	0,00	0,00	0,00
10	130	0	0,00	0,00	0,00
11	131	0	0,00	0,00	0,00
Total		8198			100,00

% Isotopic enrichment (frag1) : 16,2

13.4 Nitrogen-carbon exchange products

[1,1'-Biphenyl]-4-yl(phenyl)methanone (42)

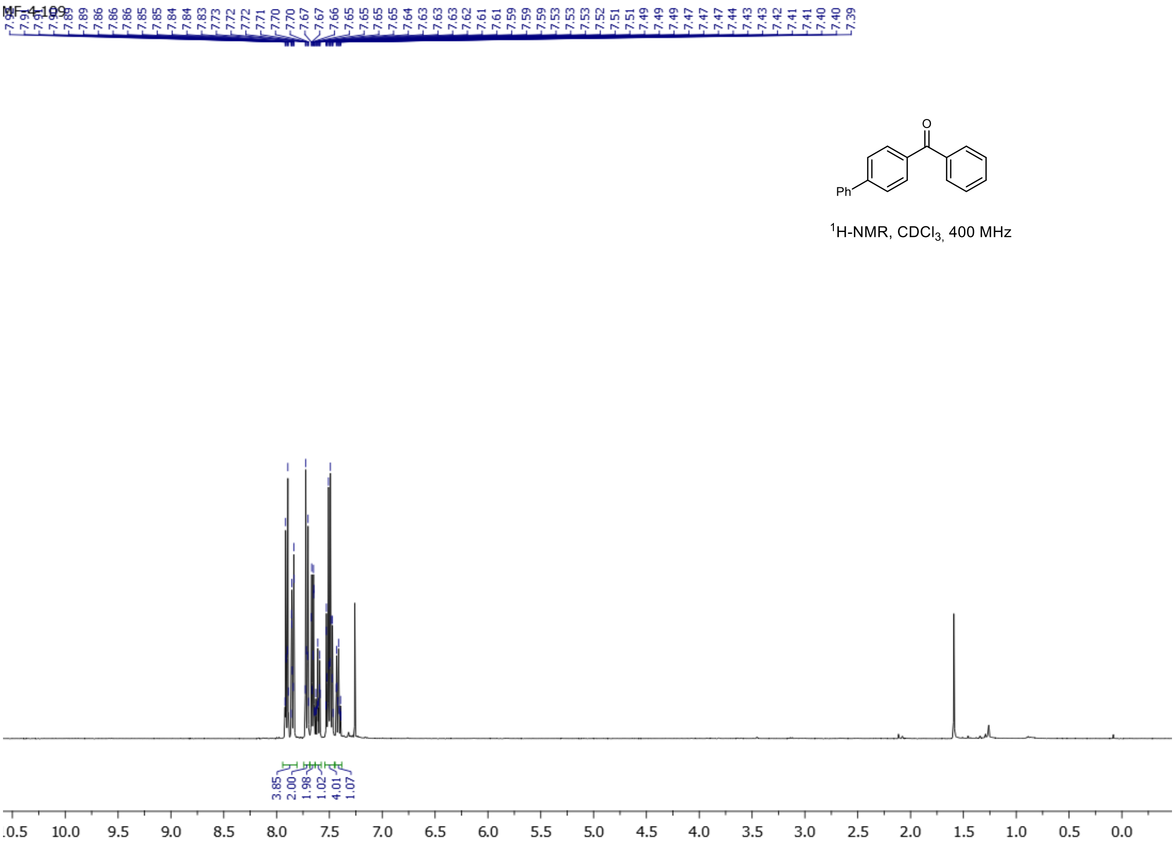

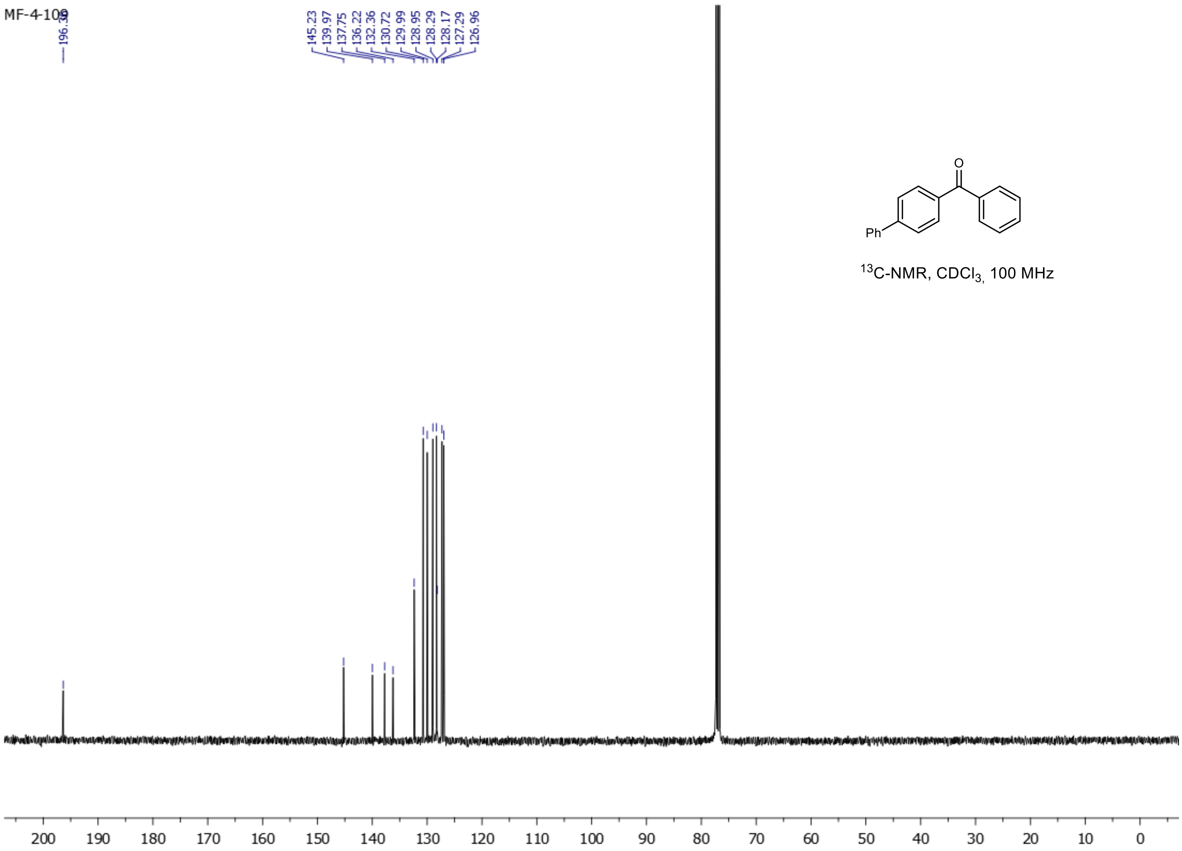

[1,1'-Biphenyl]-4-yl(3,4-dimethoxyphenyl)methanone (43)

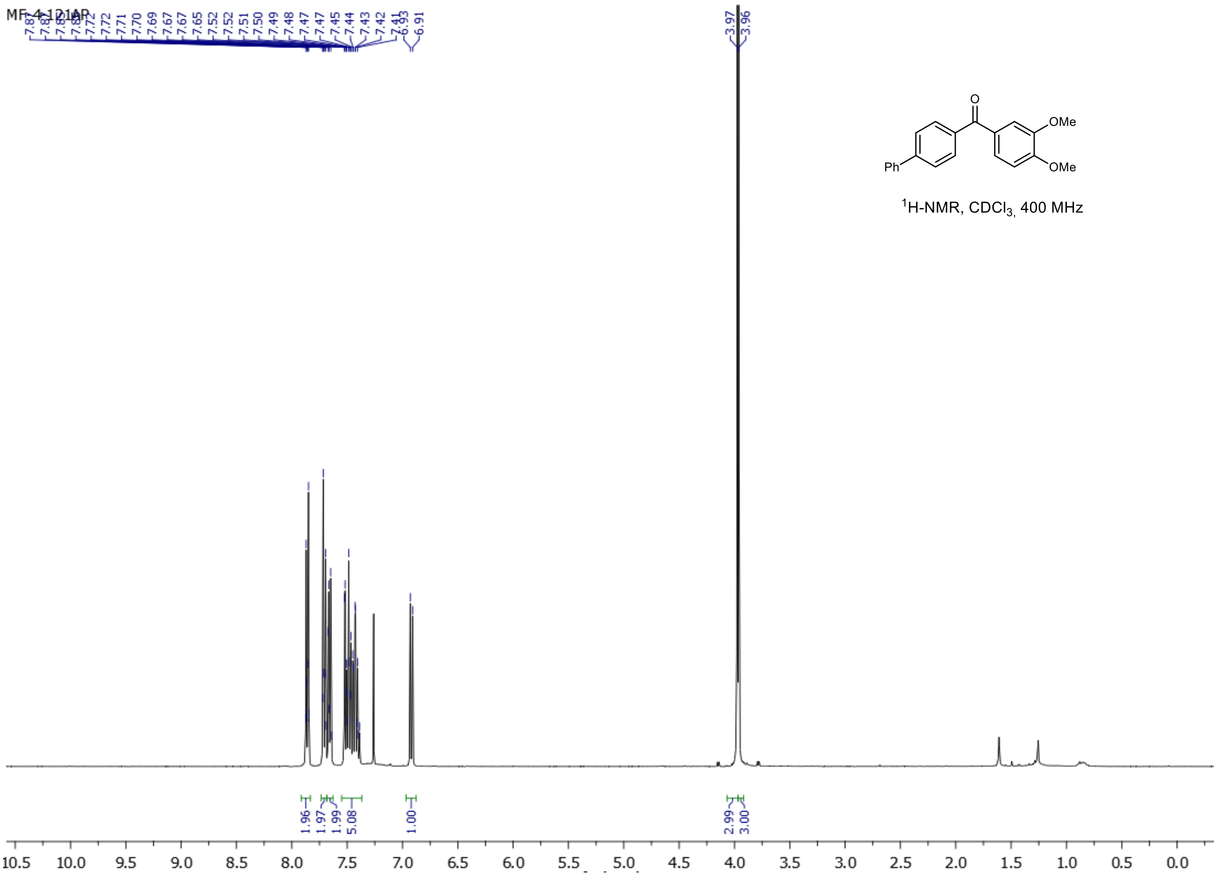

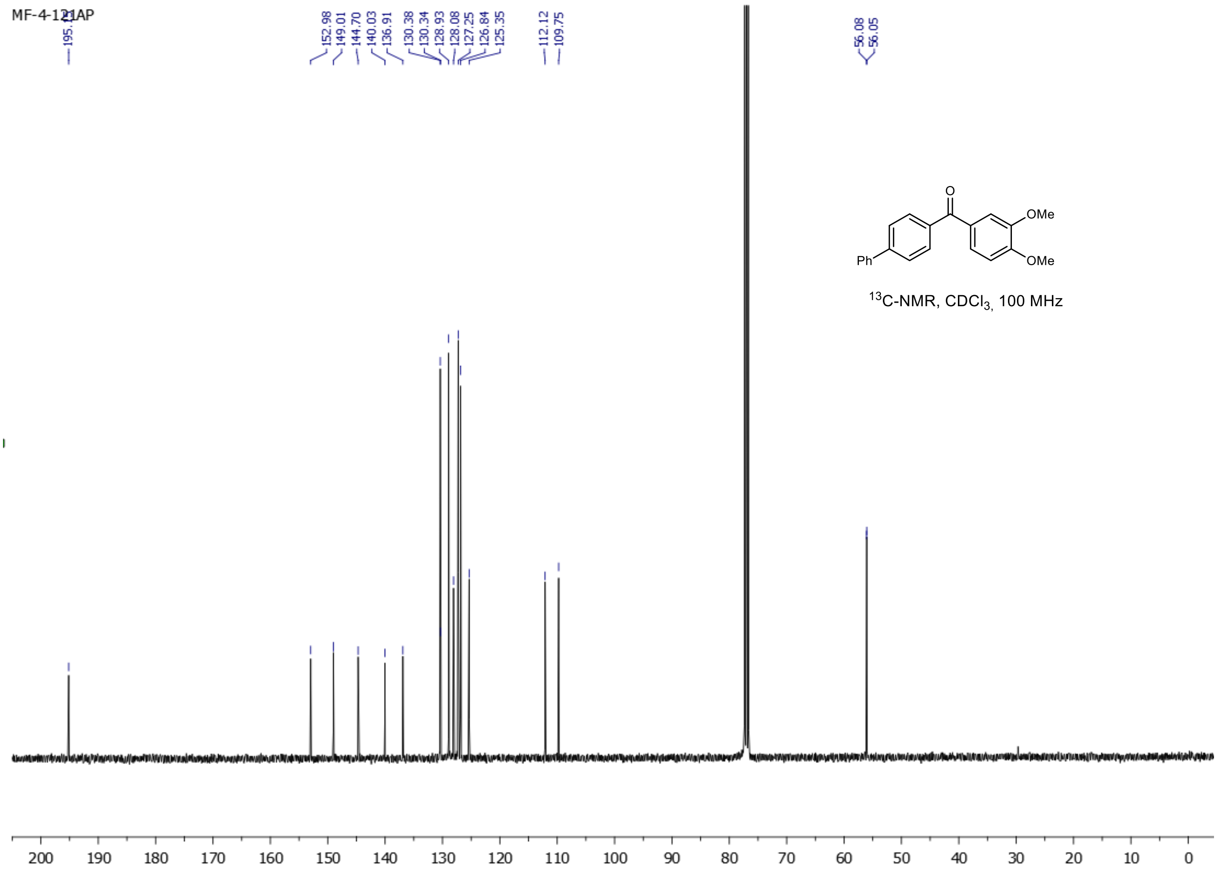

[1,1'-Biphenyl]-4-yl(3-bromophenyl)methanone (44)

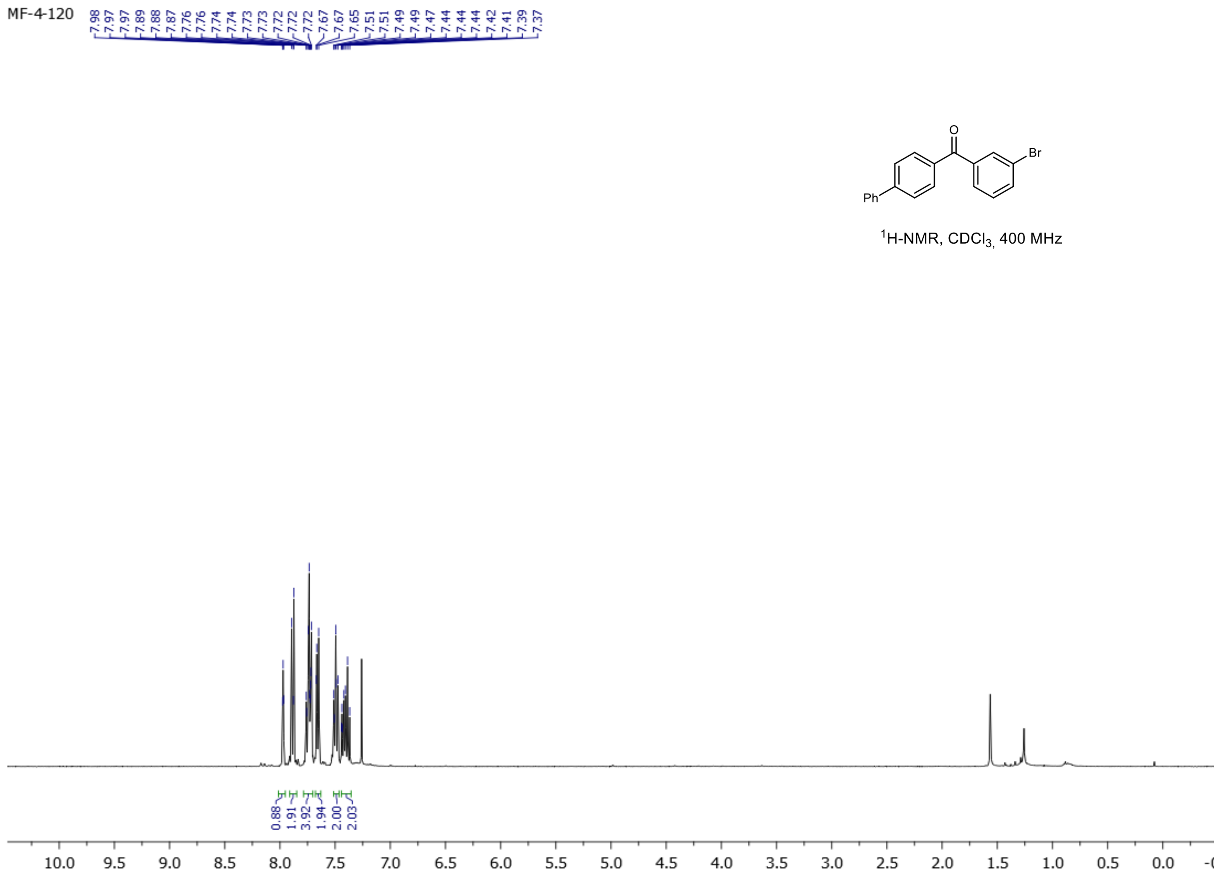

^{1}H-NMR, $CDCl_3$, 400 MHz

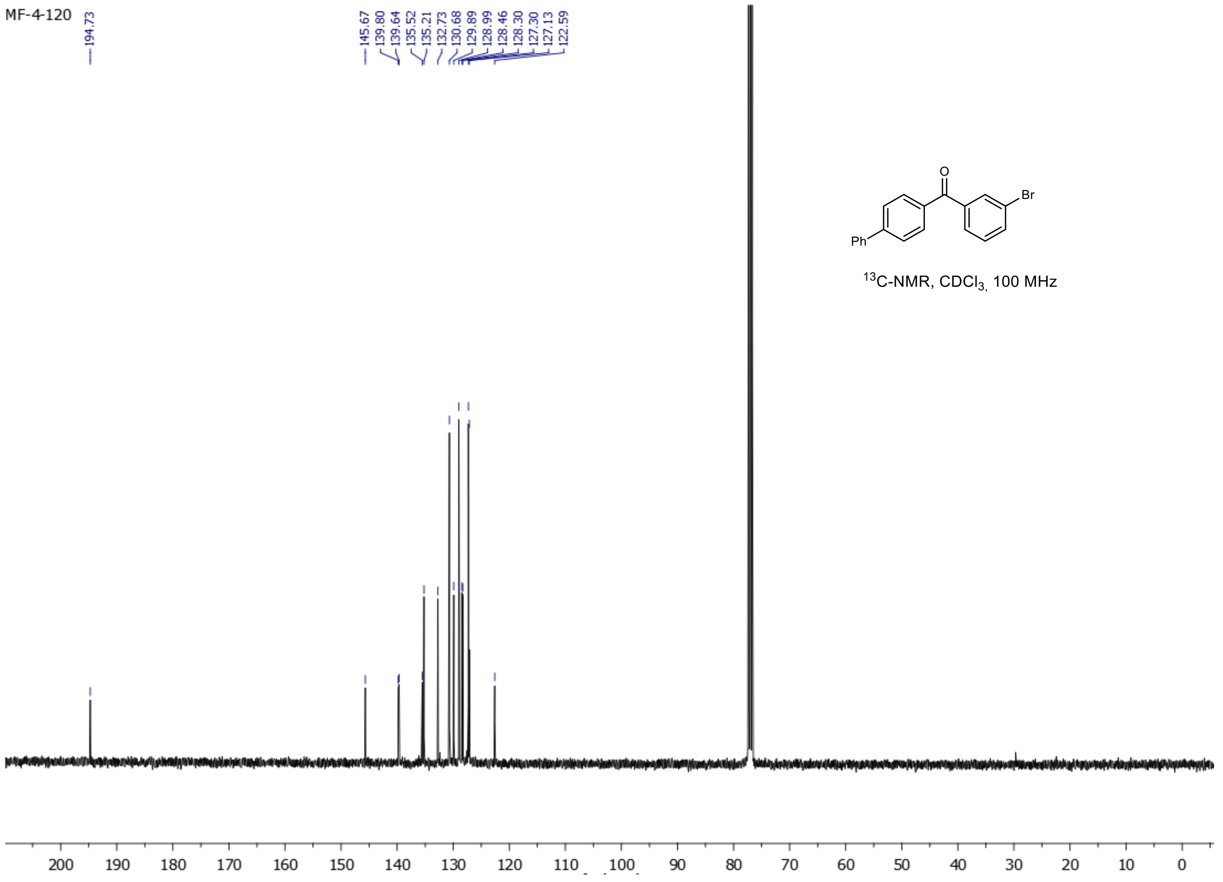

^{13}C-NMR, $CDCl_3$, 100 MHz

[1,1'-Biphenyl]-4-yl(thiophen-3-yl)methanone (45)

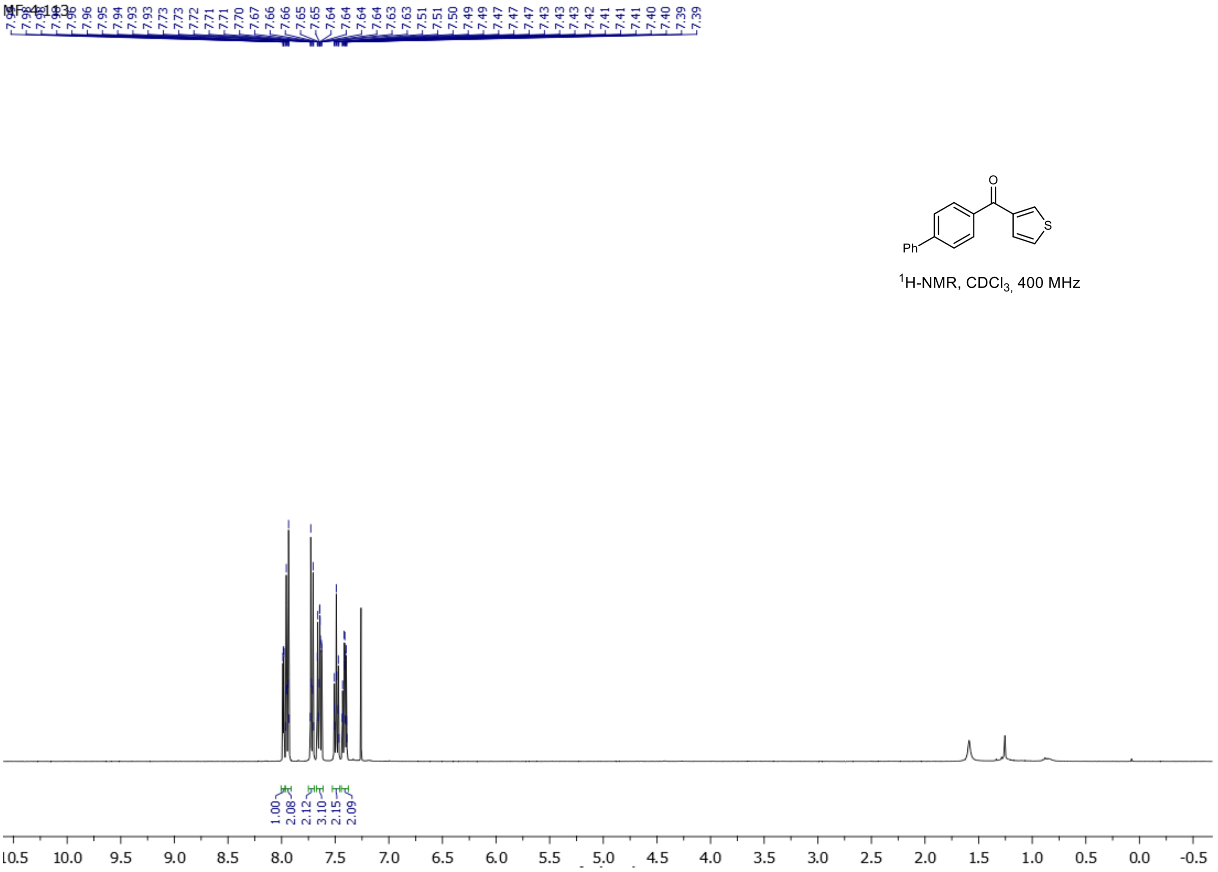

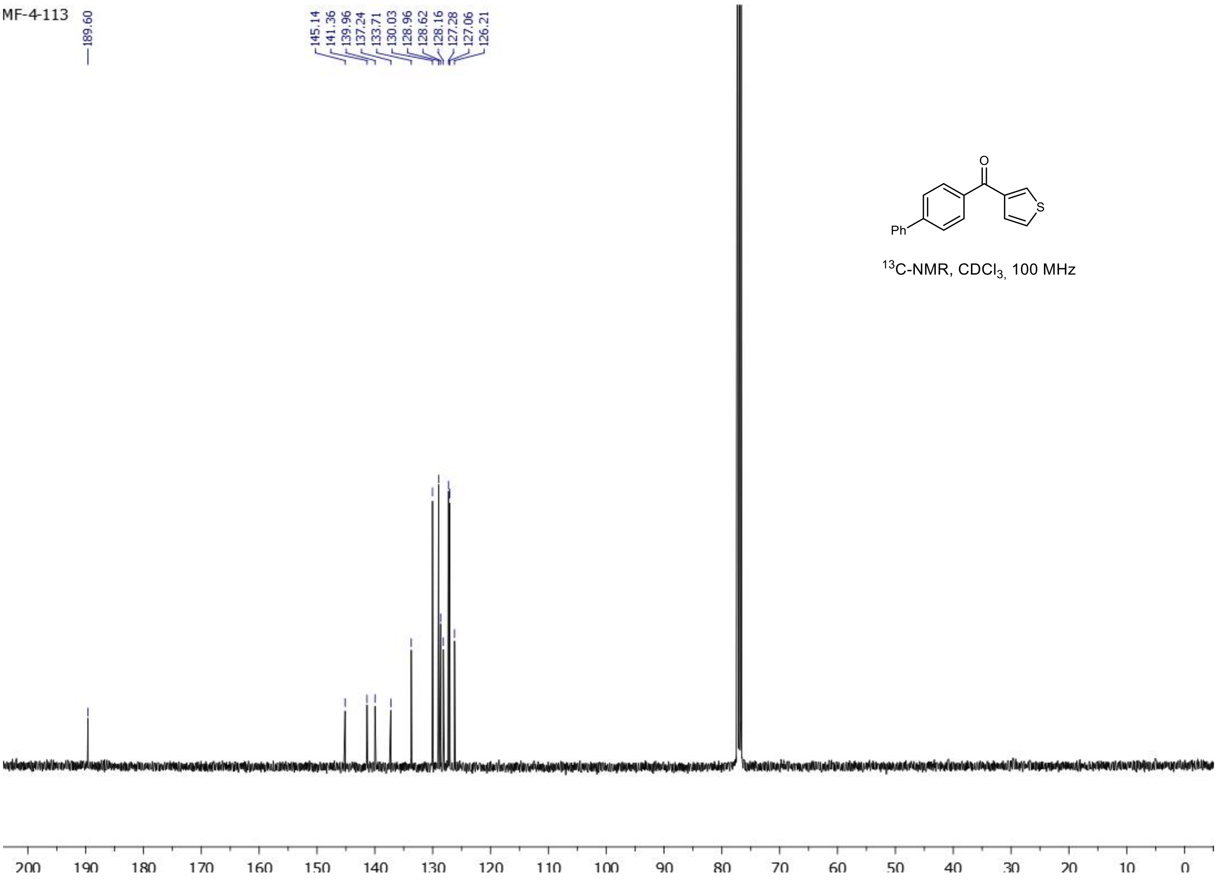

[1,1'-Biphenyl]-4-yl(5-methylthiophen-2-yl)methanone (46)

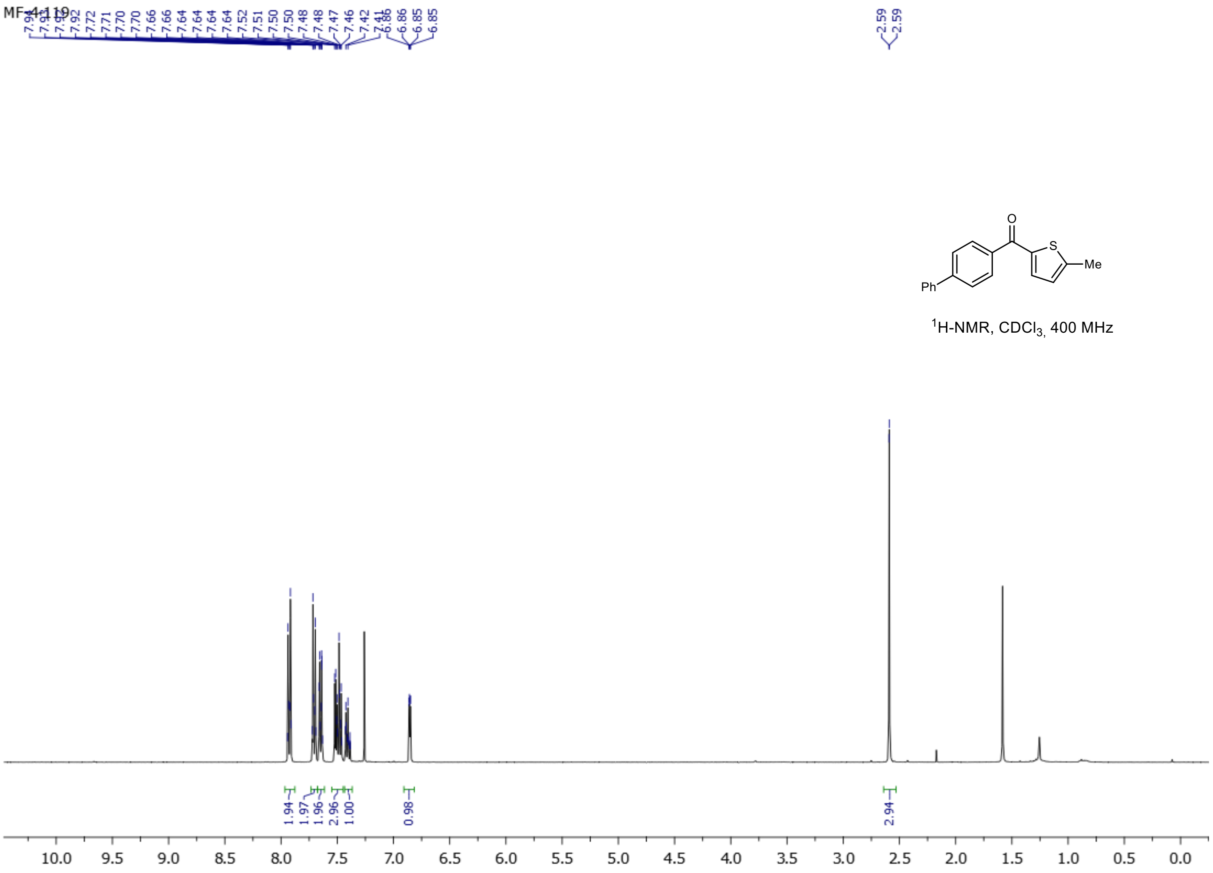

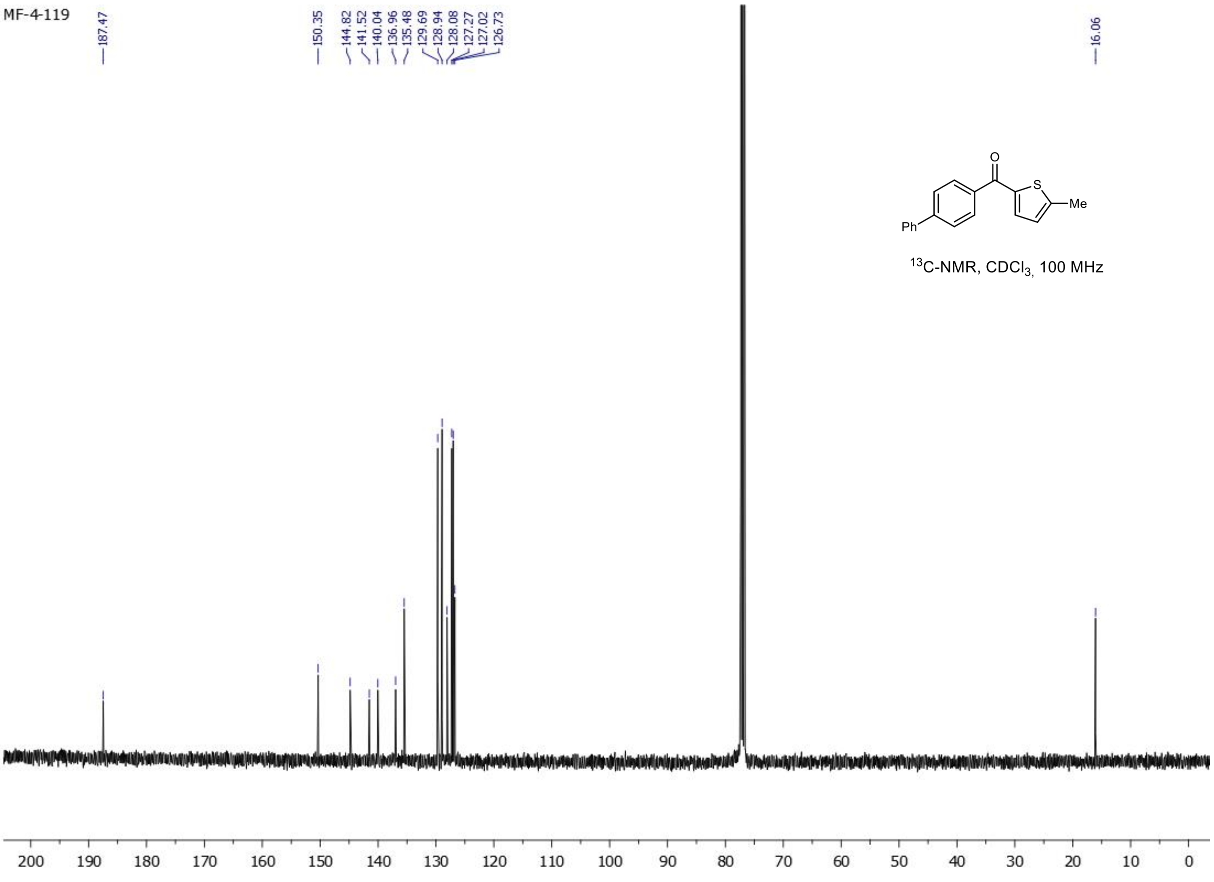

[1,1'-Biphenyl]-3-yl(phenyl)methanone (47)

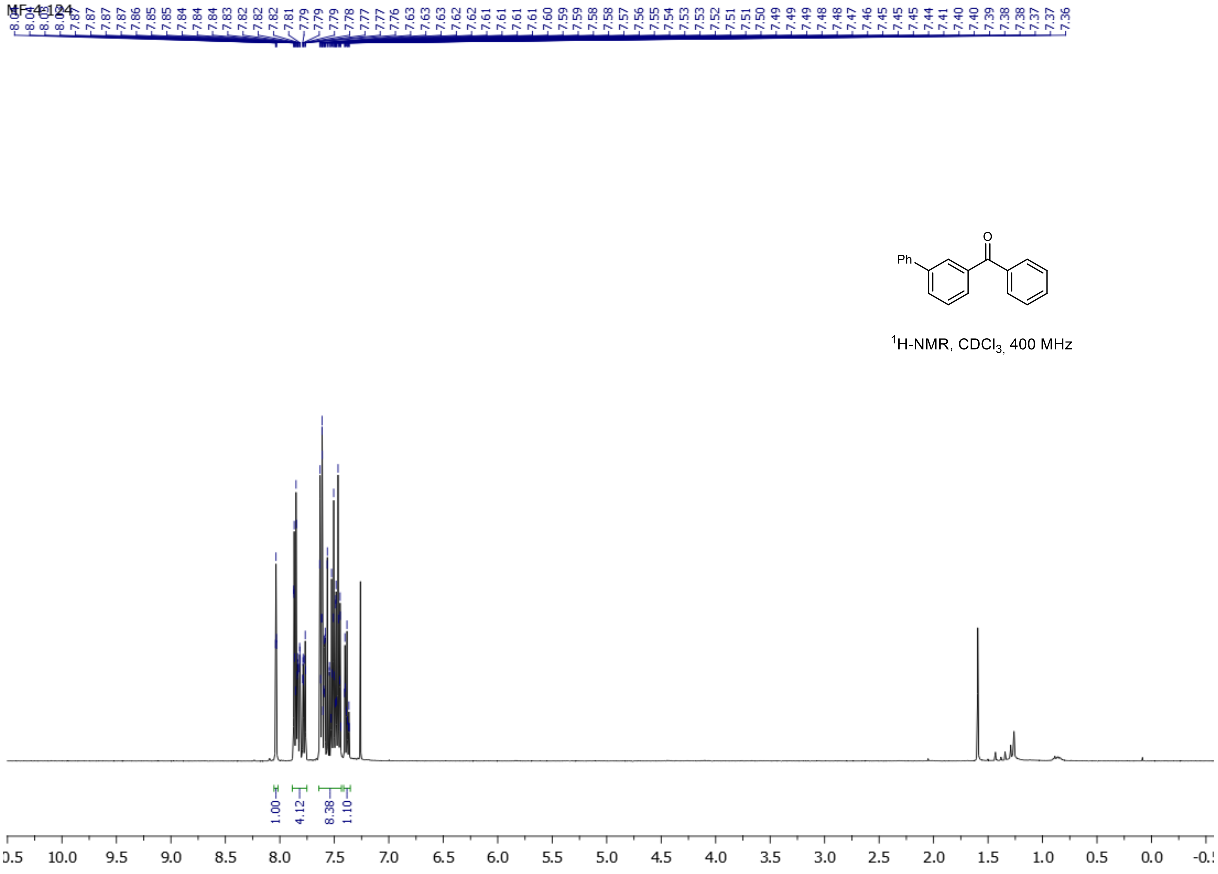

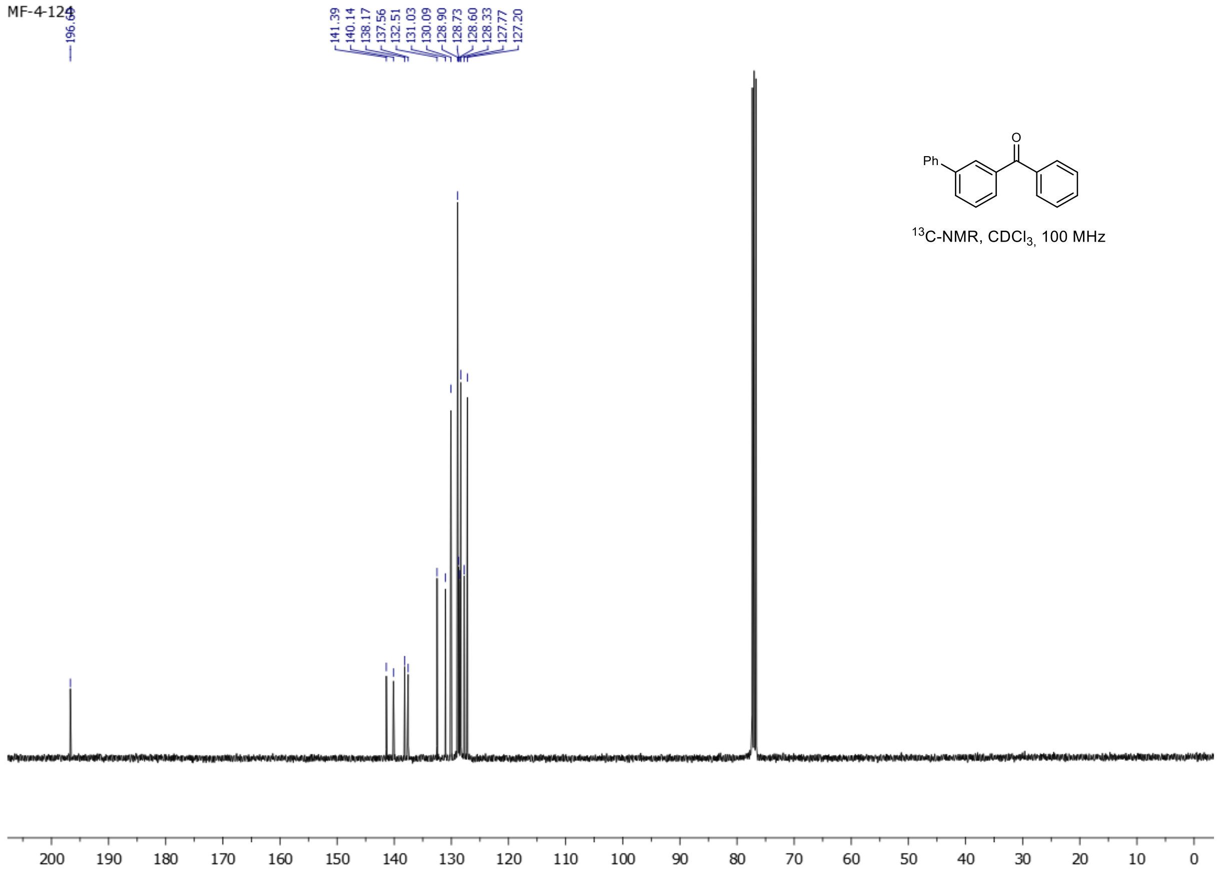

(3,4-Dimethoxyphenyl)(phenyl-d$_5$)methanone ([$^2H_5$]48)

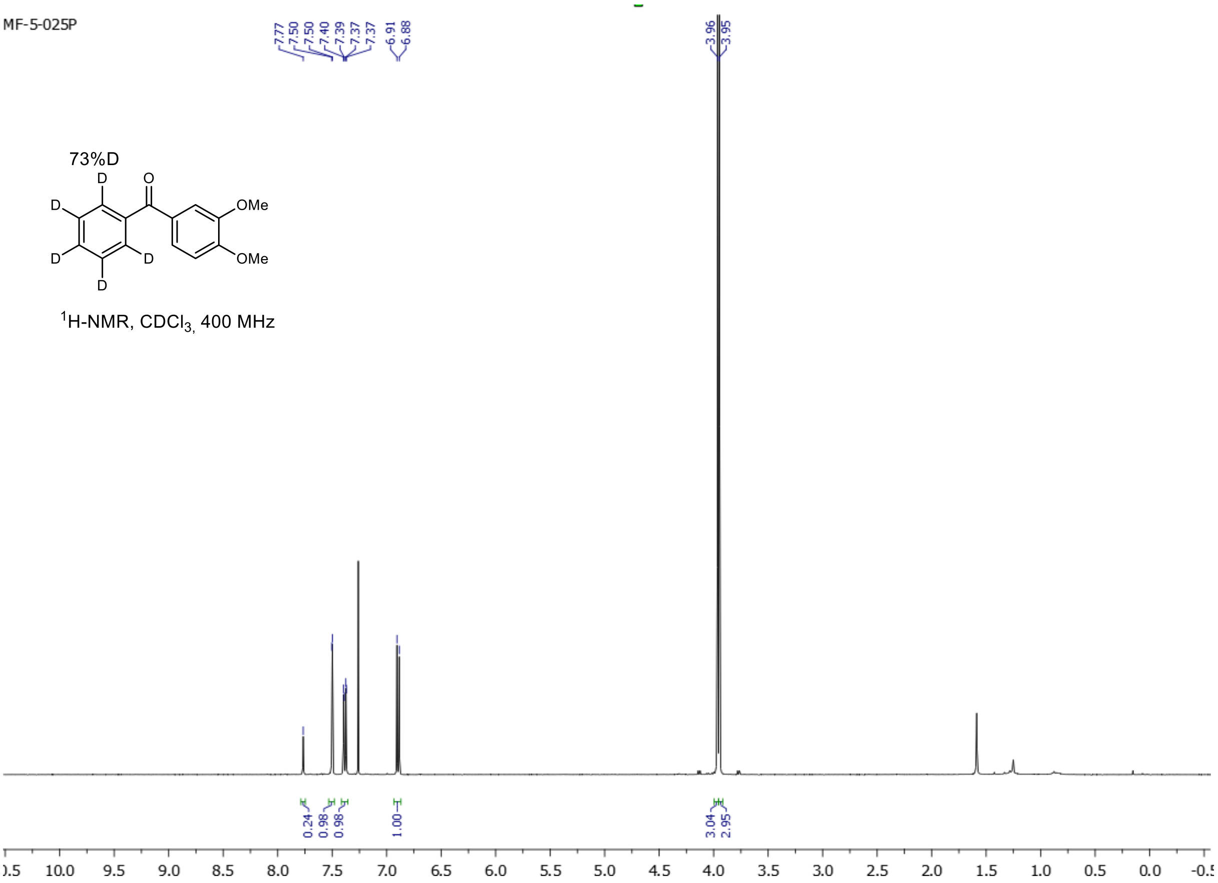

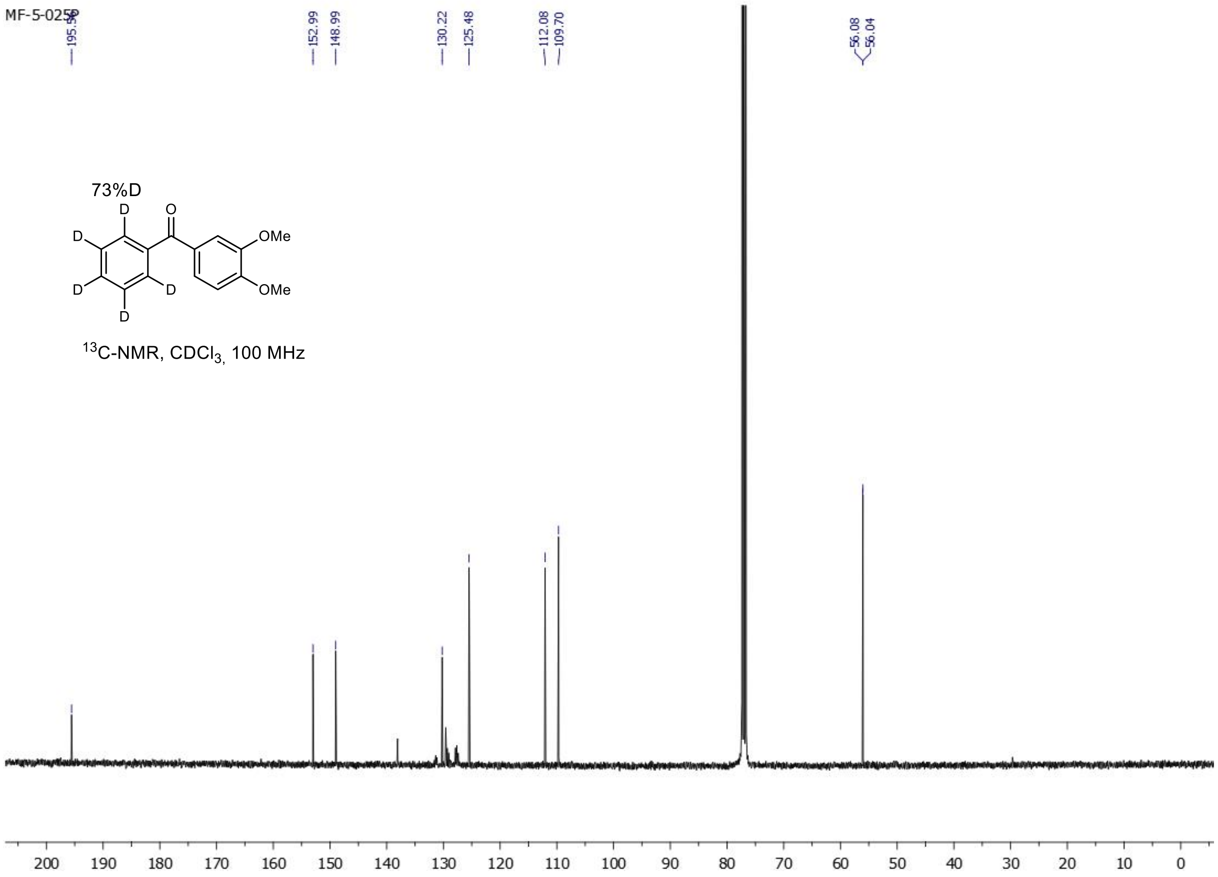

MF-5-025-D
MF-5-025-D

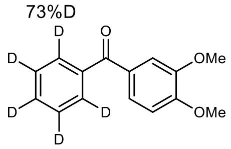

^{2}H-NMR, $CHCl_3$, 61 MHz

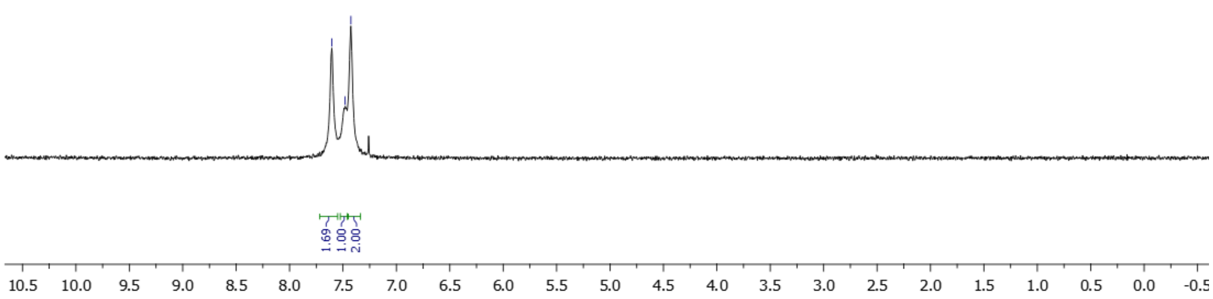

Isotopic Enrichment

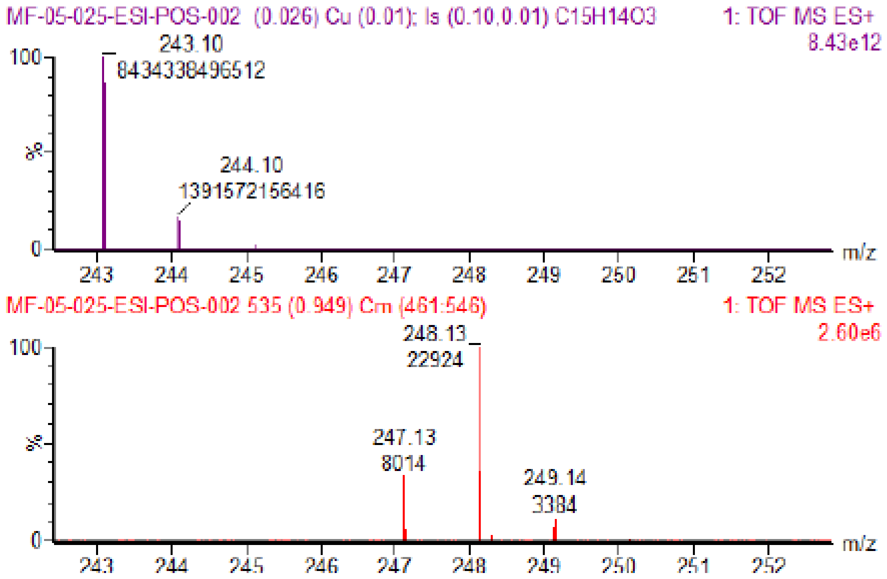

theorical isotopic distribution

	M	M+1	M+2	M+3	M+4	M+5	M+6	M+7
m/z	243	244	245	246	247	248	249	250
%	100,0	16,6	1,8	0,2	0,0	0,0	0,0	0,0

Enrichment calculation

Isotopomer	m/z	Area	natural isotope correction	Corrected area	Isotopic purity (%)
0	243	0	0,00	0,00	0,00
1	244	0	0,00	0,00	0,00
2	245	0	0,00	0,00	0,00
3	246	167	0,00	167,00	0,59
4	247	7740	0,00	7712,28	27,41
5	248	21993	0,00	20709,76	73,59
6	249	3295	0,00	-281,97	-1,00
7	250	180	0,00	-161,39	-0,57
8	251	0	0,00	-9,55	-0,03
9	252	0	0,00	5,05	0,02
10	253	0	0,00	-0,34	0,00
11	254	0	0,00	-0,01	0,00
Total		33375		28140,81	100,00

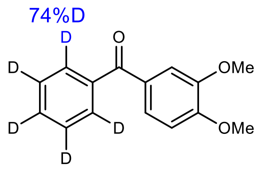

% Isotopic purity (5xD) : 73,6

(Phenyl-d$_5$)(thiophen-3-yl)methanone ([$^2H_5$]49)

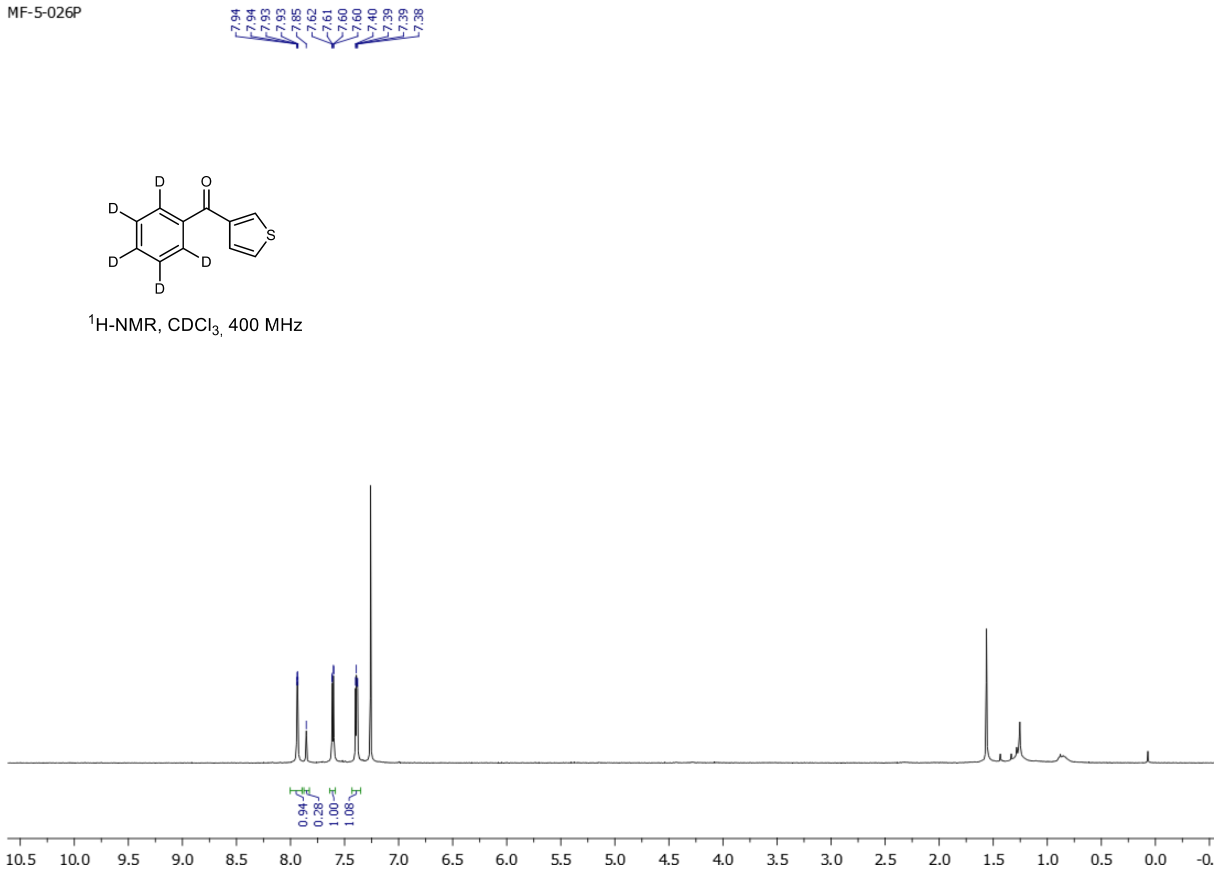

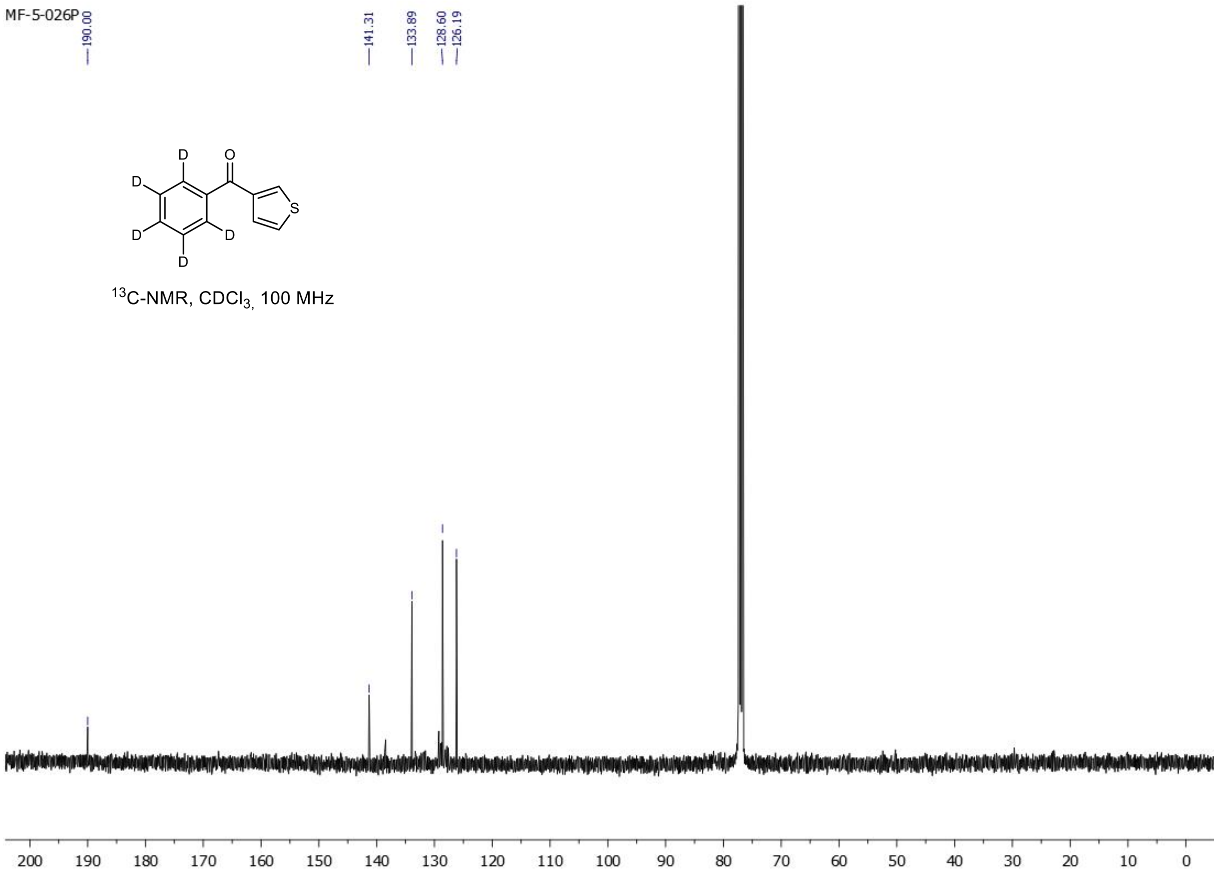

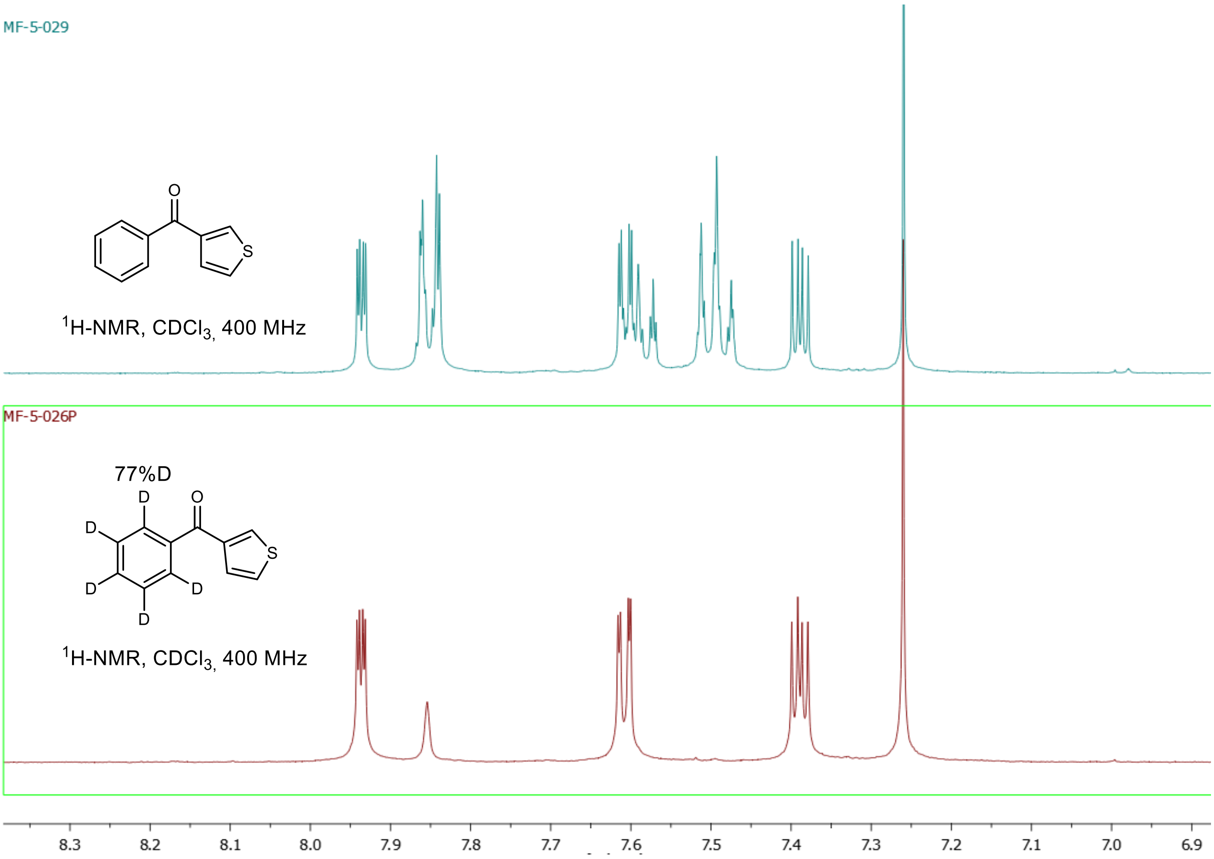
MF-5-029
O
S
^{1}H-NMR, CDCl$_3$, 400 MHz
MF-5-026P
77%D
D
O
D
S
D
D
D
^{1}H-NMR, CDCl$_3$, 400 MHz
8.3
8.2
8.1
8.0
7.9
7.8
7.7
7.6
7.5
7.4
7.3
7.2
7.1
7.0
6.9

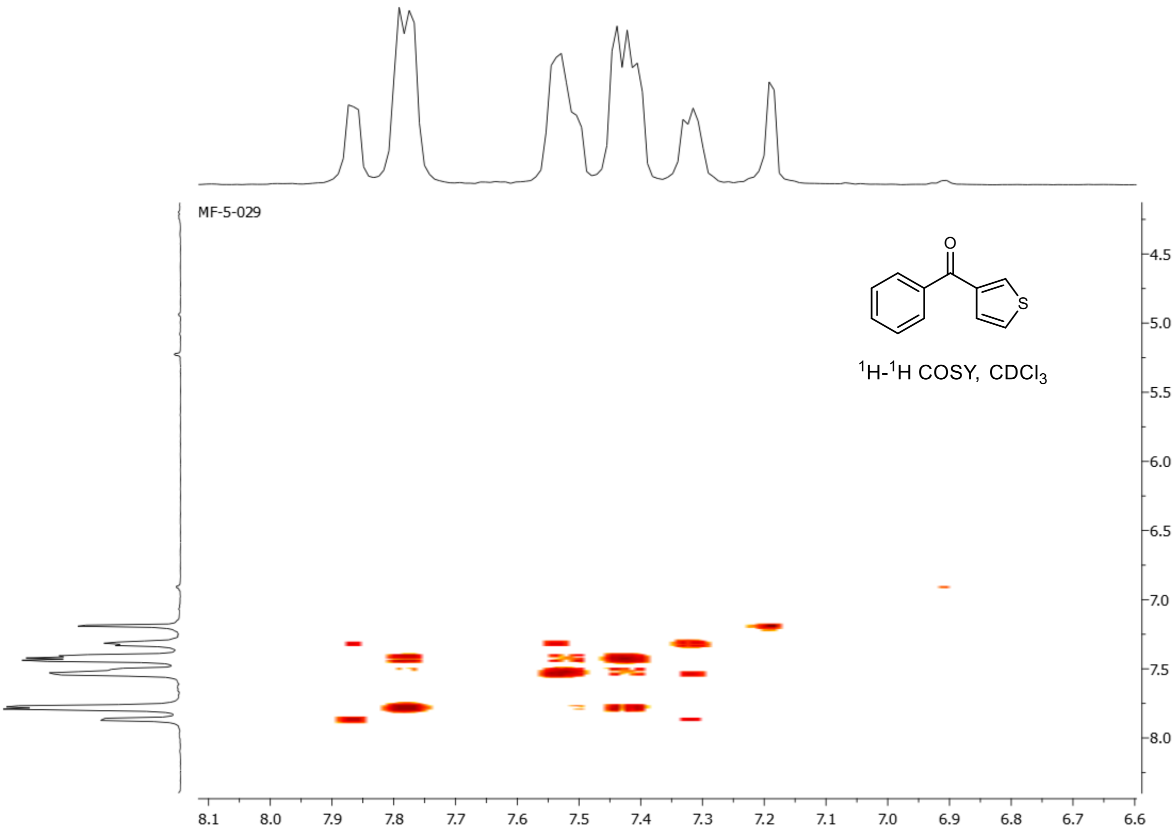
MF-5-029
O
S
^{1}H-^{1}H COSY, CDCl$_3$
4.5
5.0
5.5
6.0
6.5
7.0
7.5
8.0
8.1
8.0
7.9
7.8
7.7
7.6
7.5
7.4
7.3
7.2
7.1
7.0
6.9
6.8
6.7
6.6

MF-5-026-D
MF-5-026-D

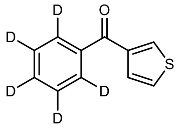

^{2}H-NMR, $CHCl_3$, 61 MHz

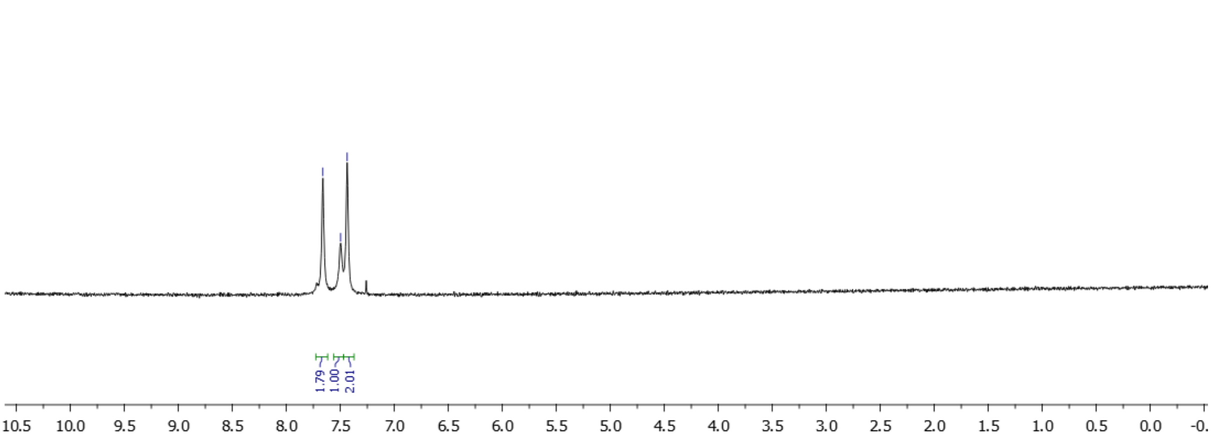

Isotopic Enrichment

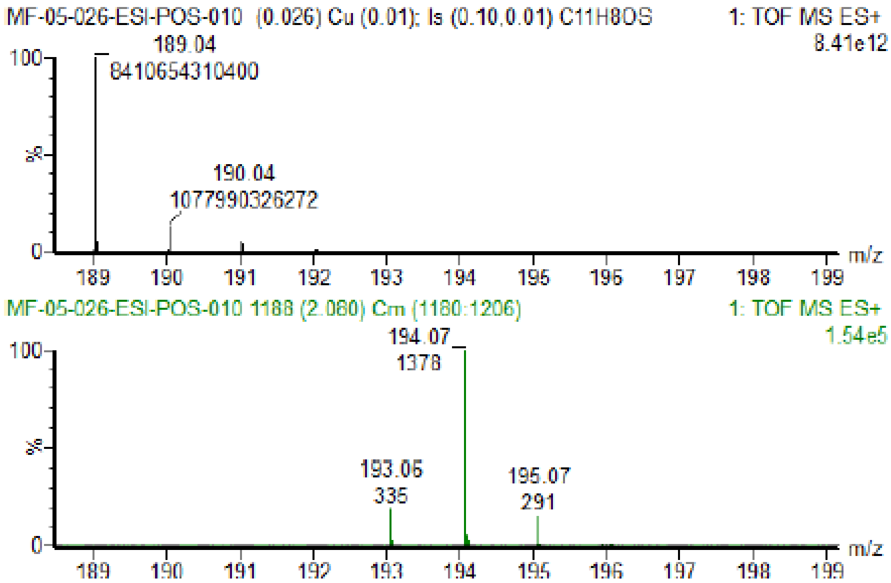

theorical isotopic distribution

	M	M+1	M+2	M+3	M+4	M+5	M+6	M+7
m/z	189	190	191	192	193	194	195	196
%	100,0	12,8	5,4	0,5	0,0	0,0	0,0	0,0

Enrichment calculation

Isotopomer	m/z	Area	natural isotope correction	Corrected area	Isotopic purity (%)
0	189	0	0,00	0,00	0,00
1	190	0	0,00	0,00	0,00
2	191	0	0,00	0,00	0,00
3	192	7	0,00	7,00	0,40
4	193	335	0,00	334,10	19,25
5	194	1378	0,00	1334,86	76,92
6	195	291	0,00	102,06	5,88
7	196	41	0,00	-45,82	-2,64
8	197	8	0,00	1,68	0,10
9	198	0	0,00	1,75	0,10
10	199	0	0,00	-0,09	0,00
11	200	0	0,00	-0,09	-0,01
Total		2060		1735,46	100,00

77%D

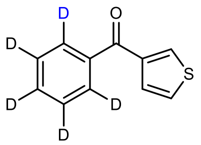

% Isotopic purity (5xD): 76,9

Phenyl(4-phenylphenyl-1-^{13}C)methanone ([$^{13}C_1$]42)

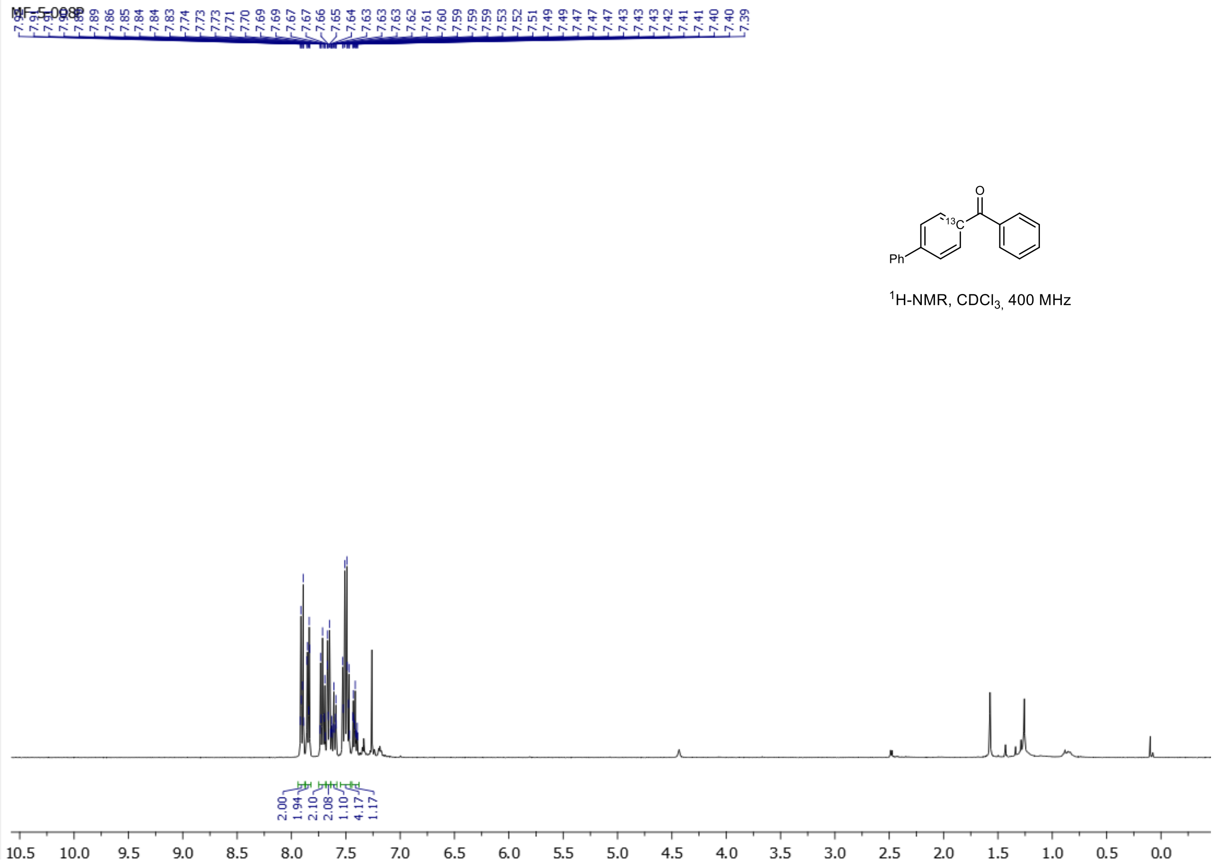

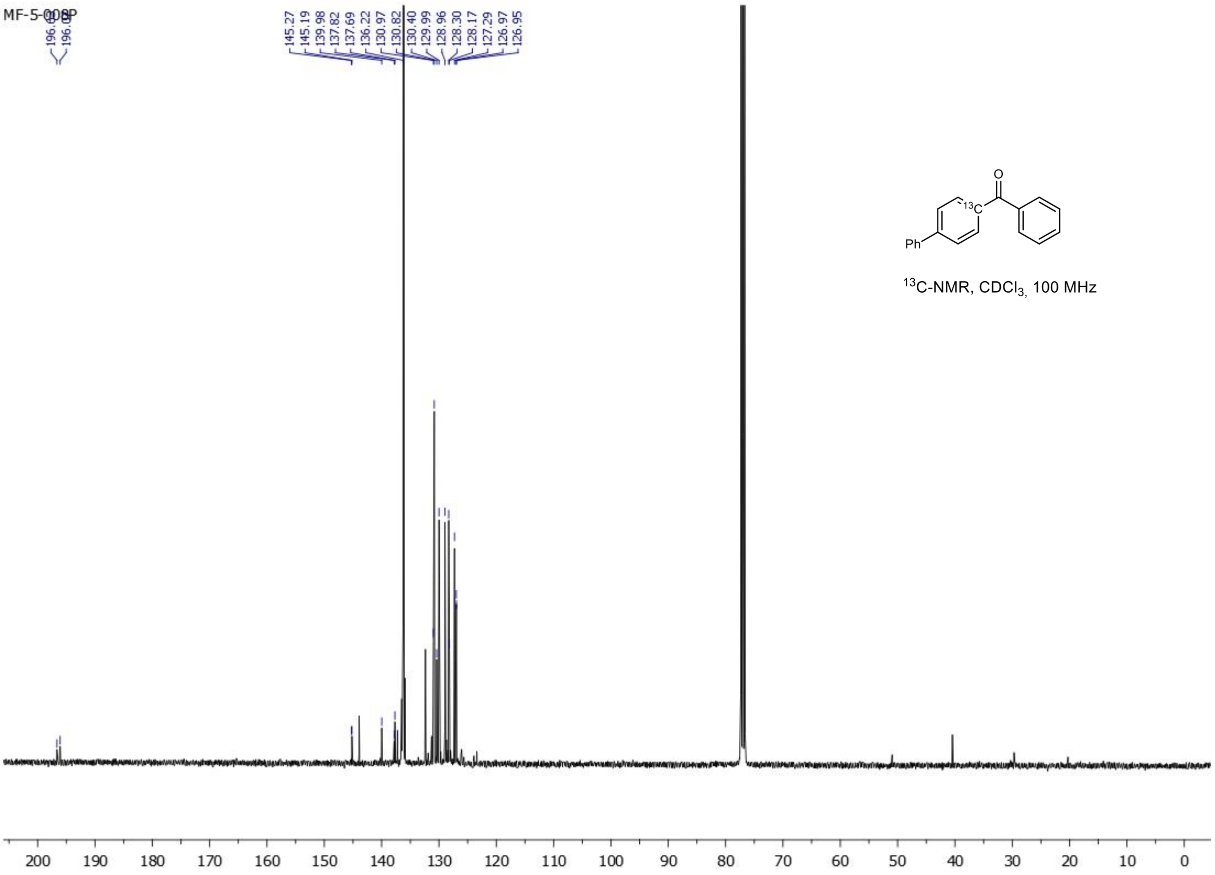

Phenyl(3-phenylphenyl-1-^{13}C)methanone ([$^{13}C_1$]47)

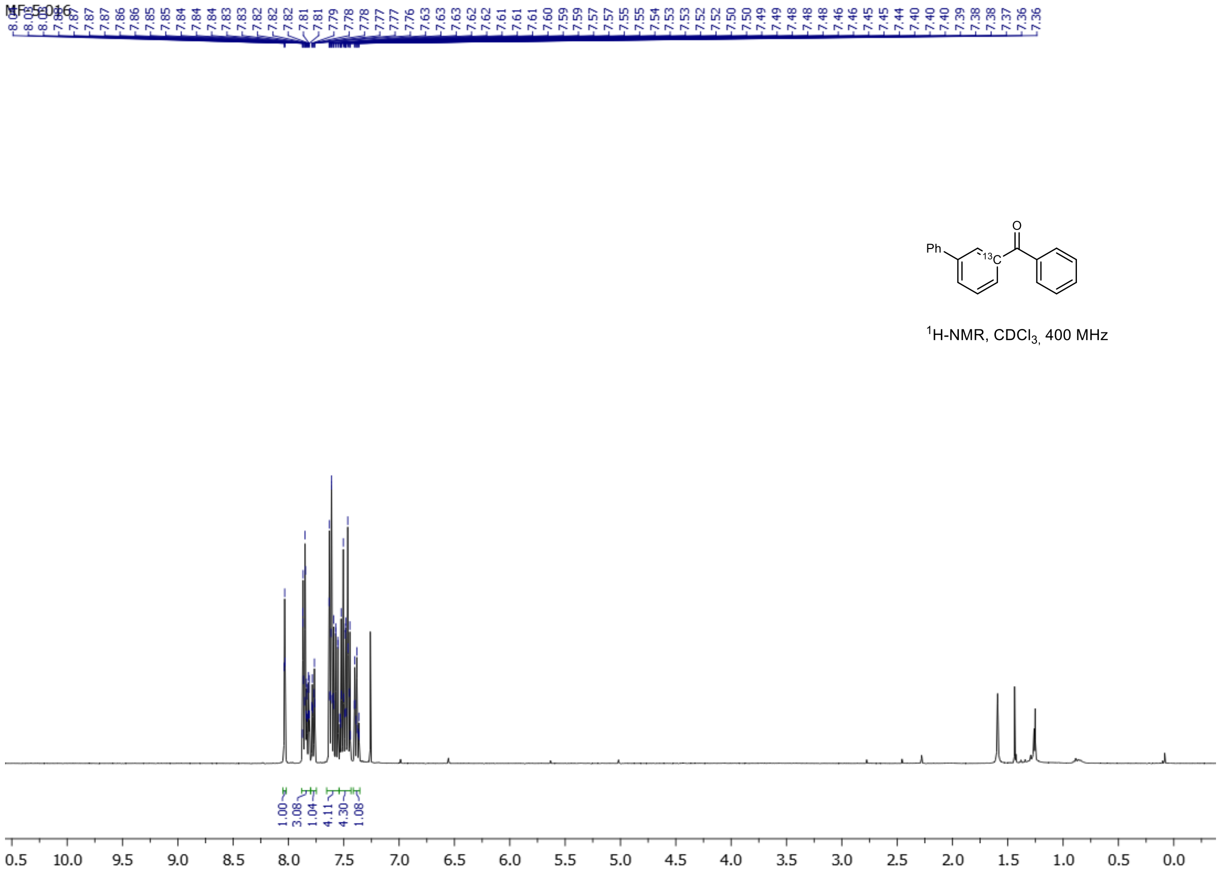

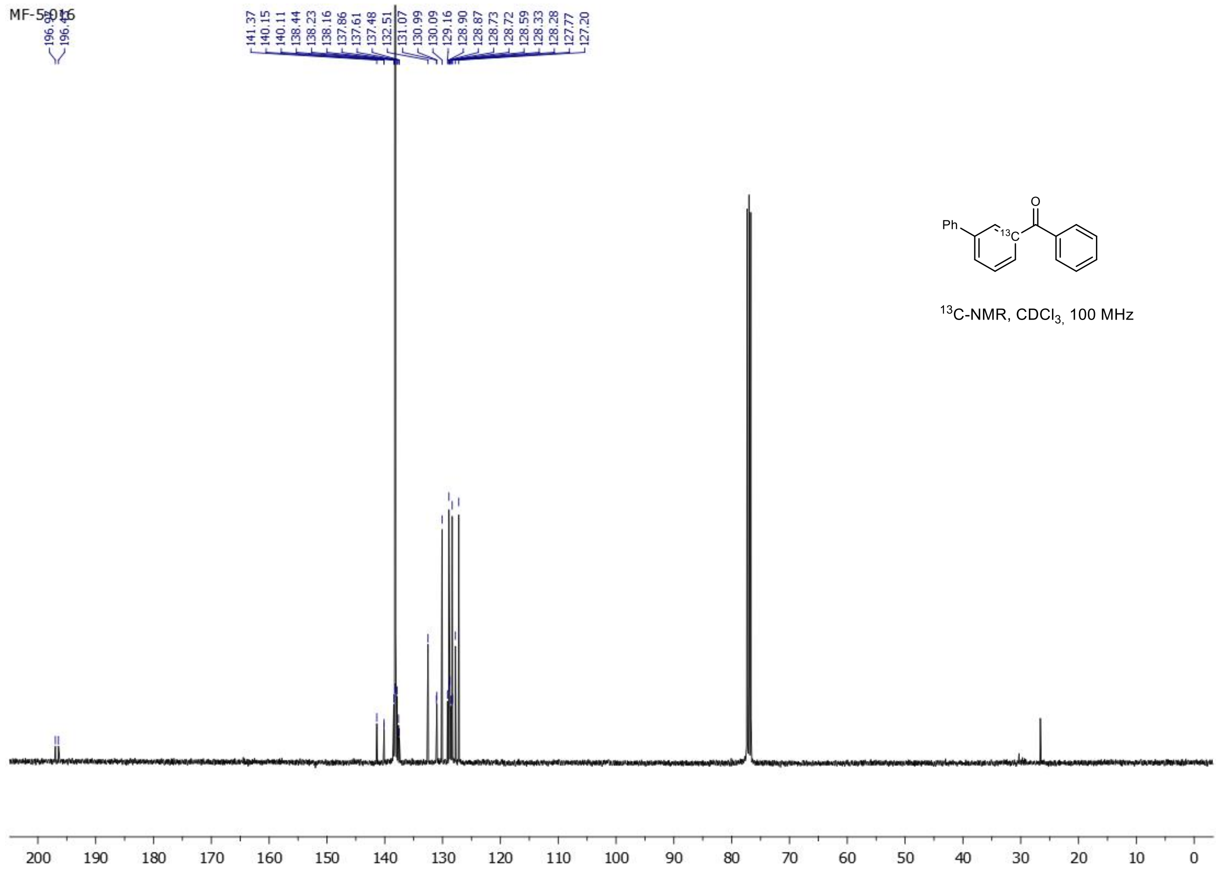

d_5-Benzophenone-1-^{13}C ([$^2H_5$, $^{13}C_1$]50)

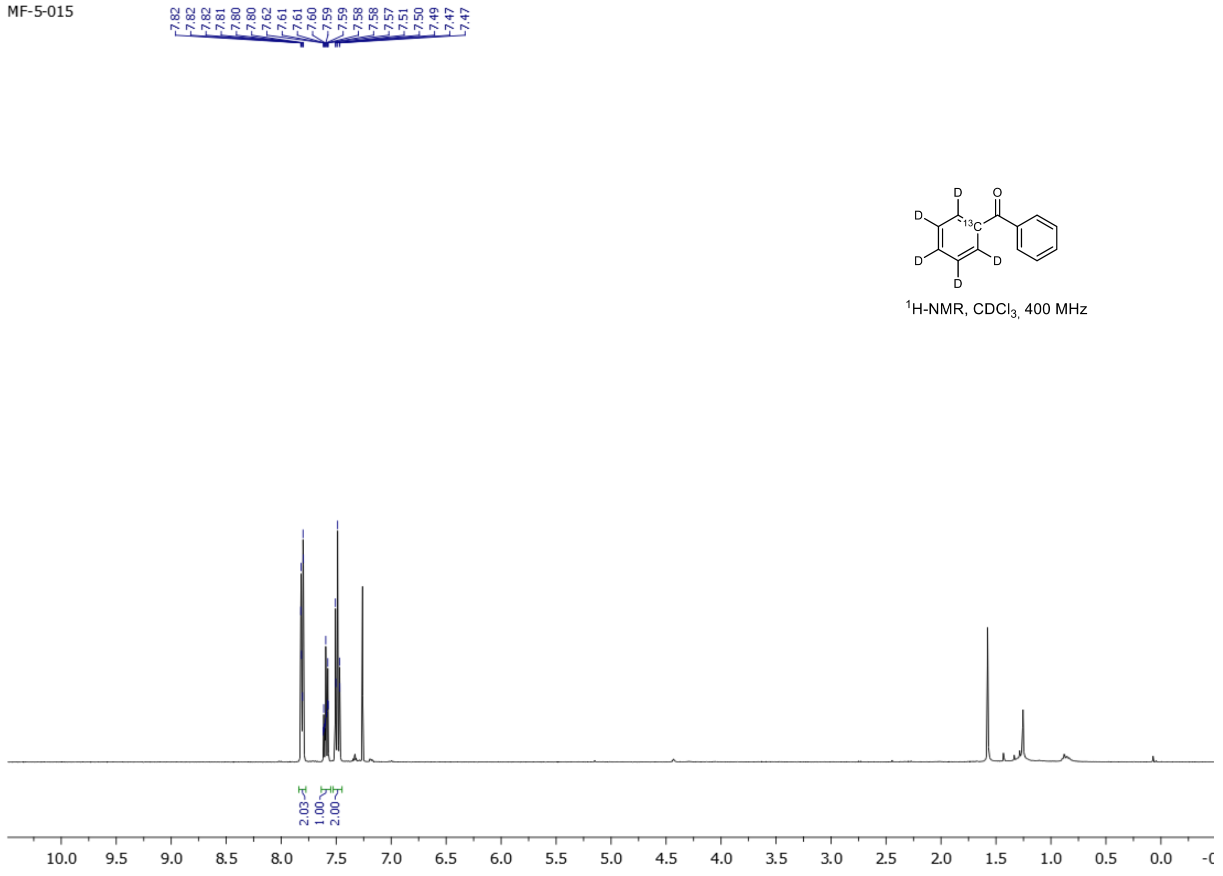

^{1}H-NMR, $CDCl_3$, 400 MHz

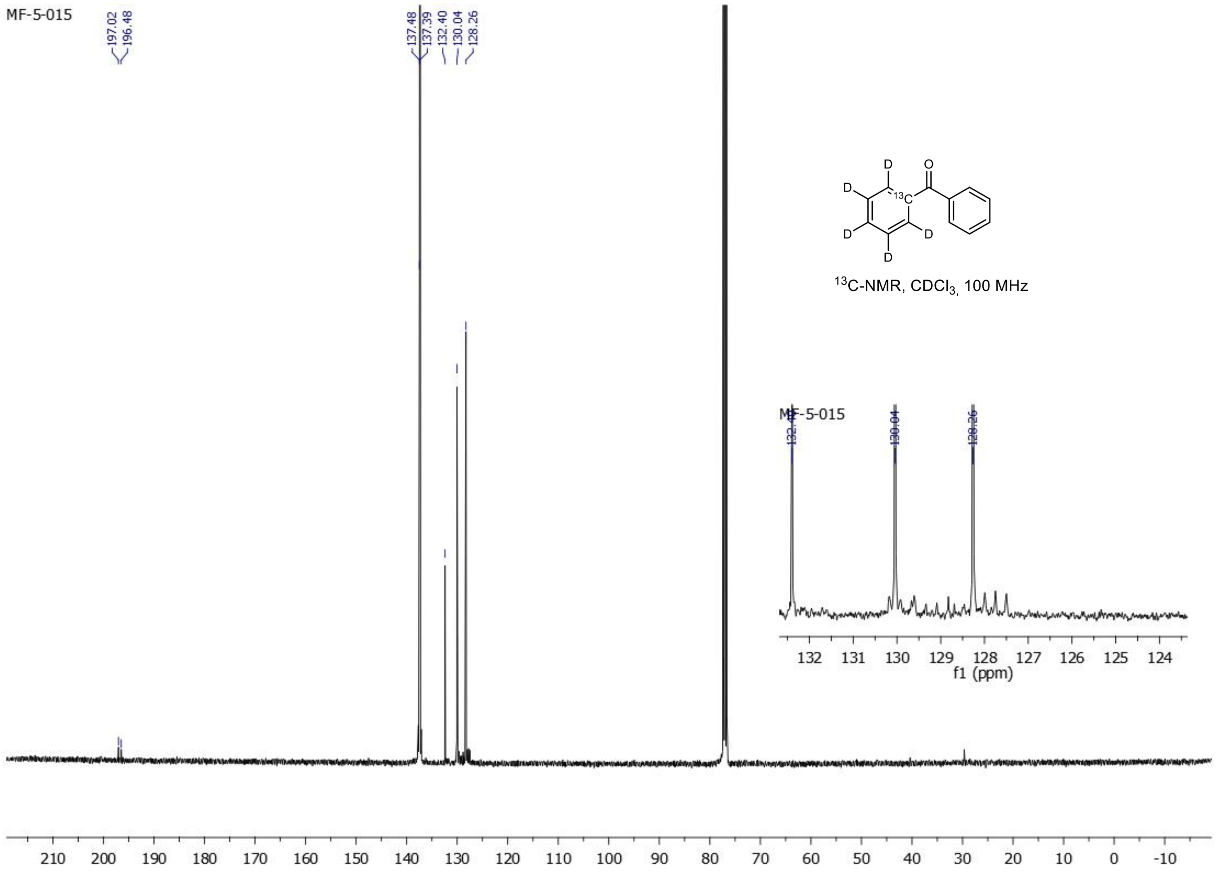

^{13}C-NMR, $CDCl_3$, 100 MHz

MF-4-015-D
MF-4-015-D

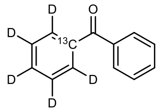

^{2}H-NMR, $CHCl_3$, 61 MHz

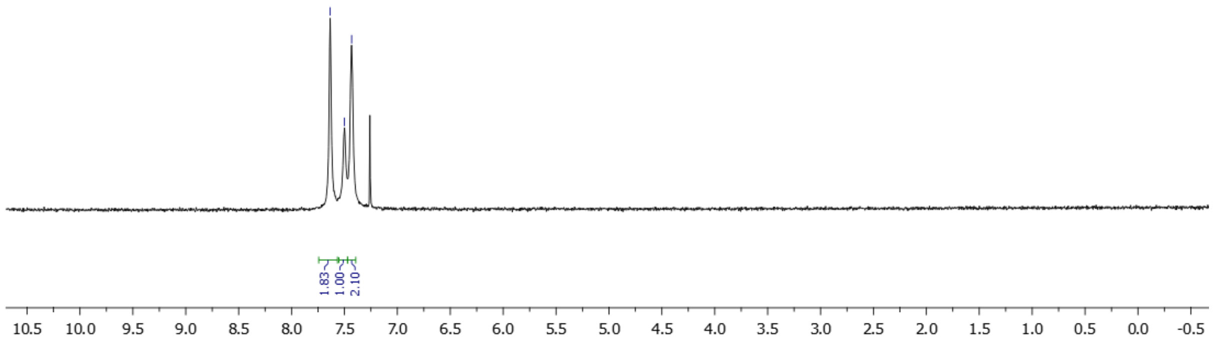

Benzophenone-1-^{13}C ([$^{13}C_1$]50)

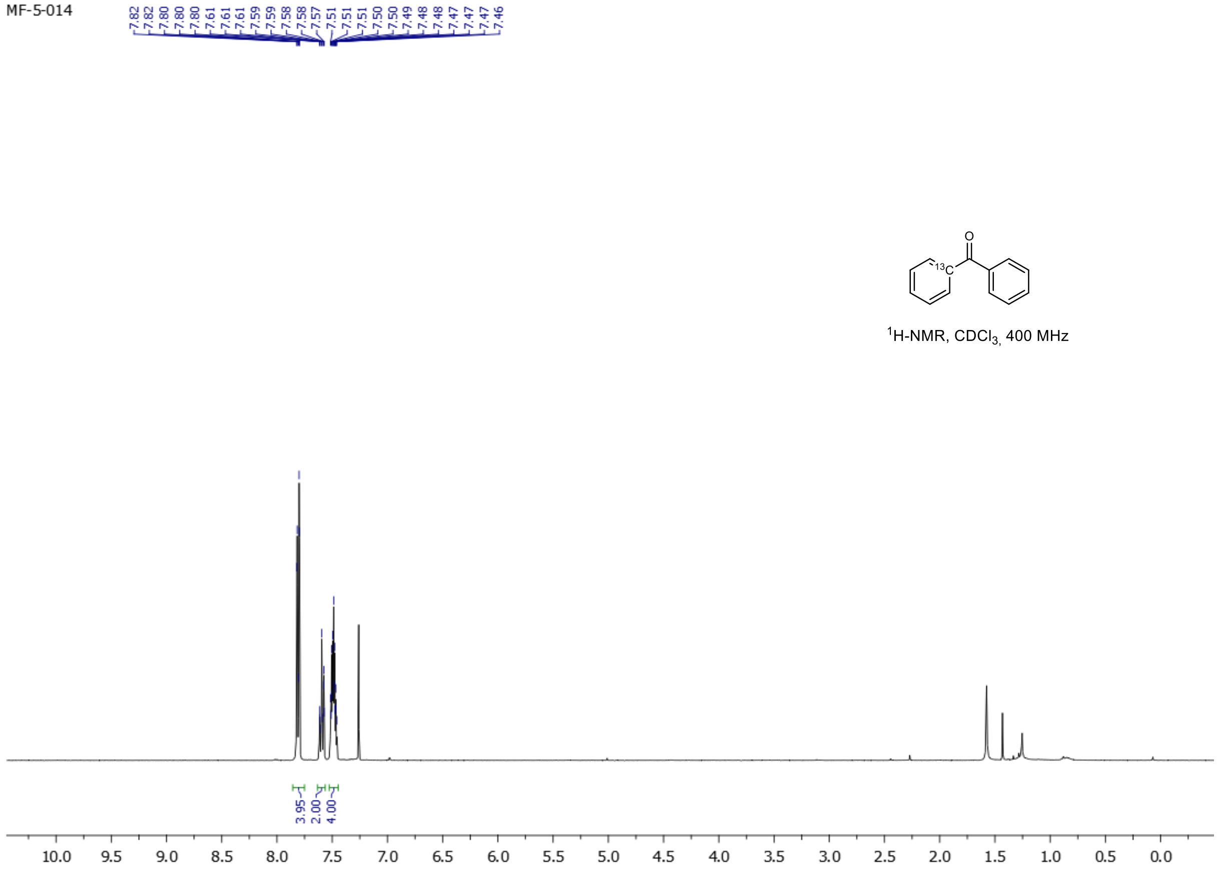

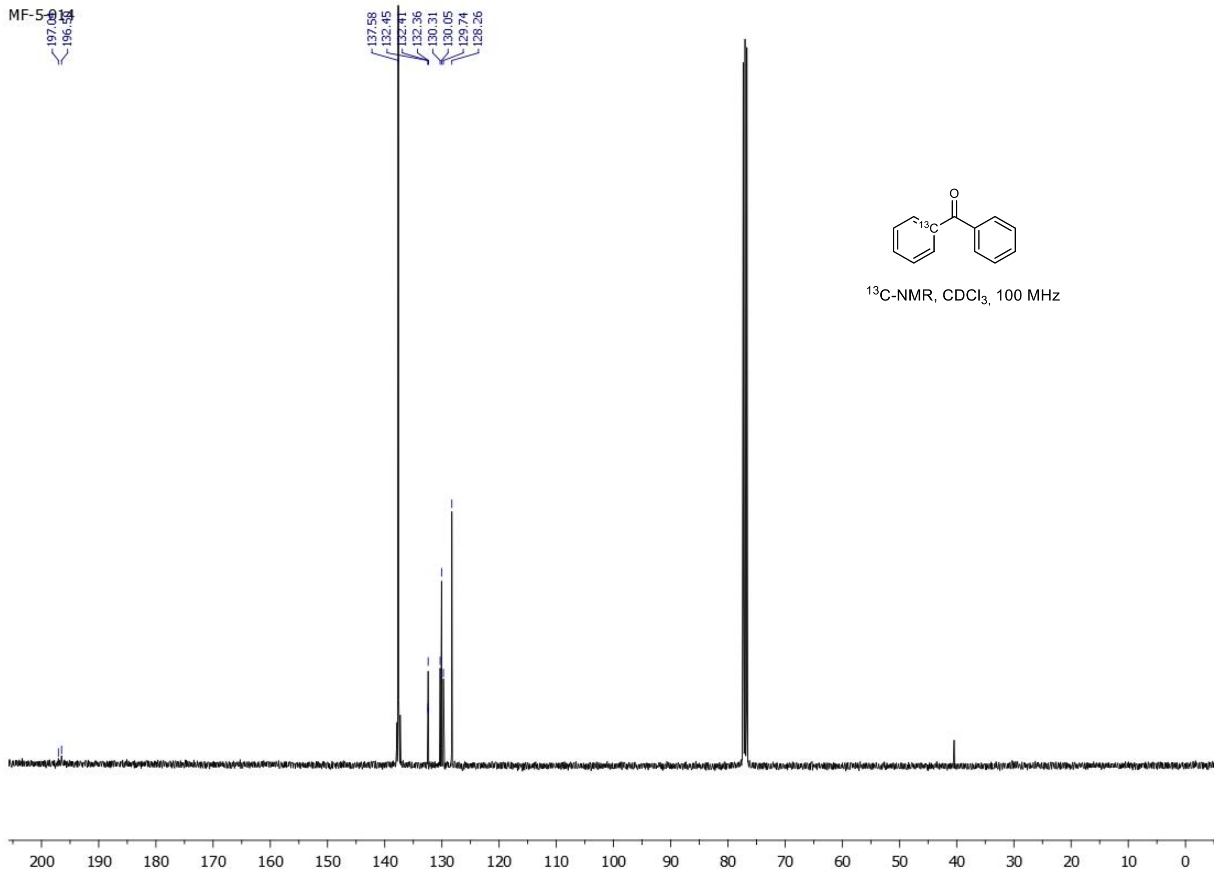

Benzophenone (50)

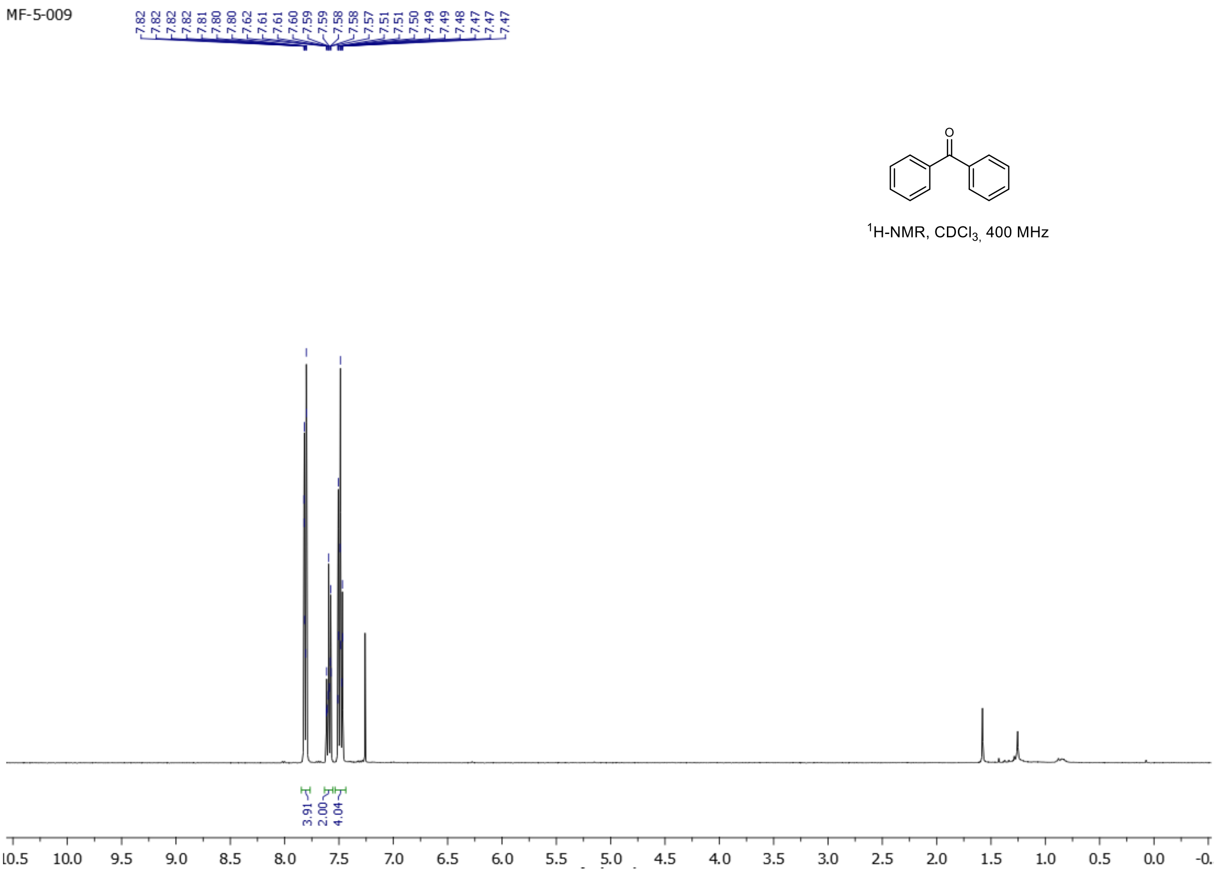

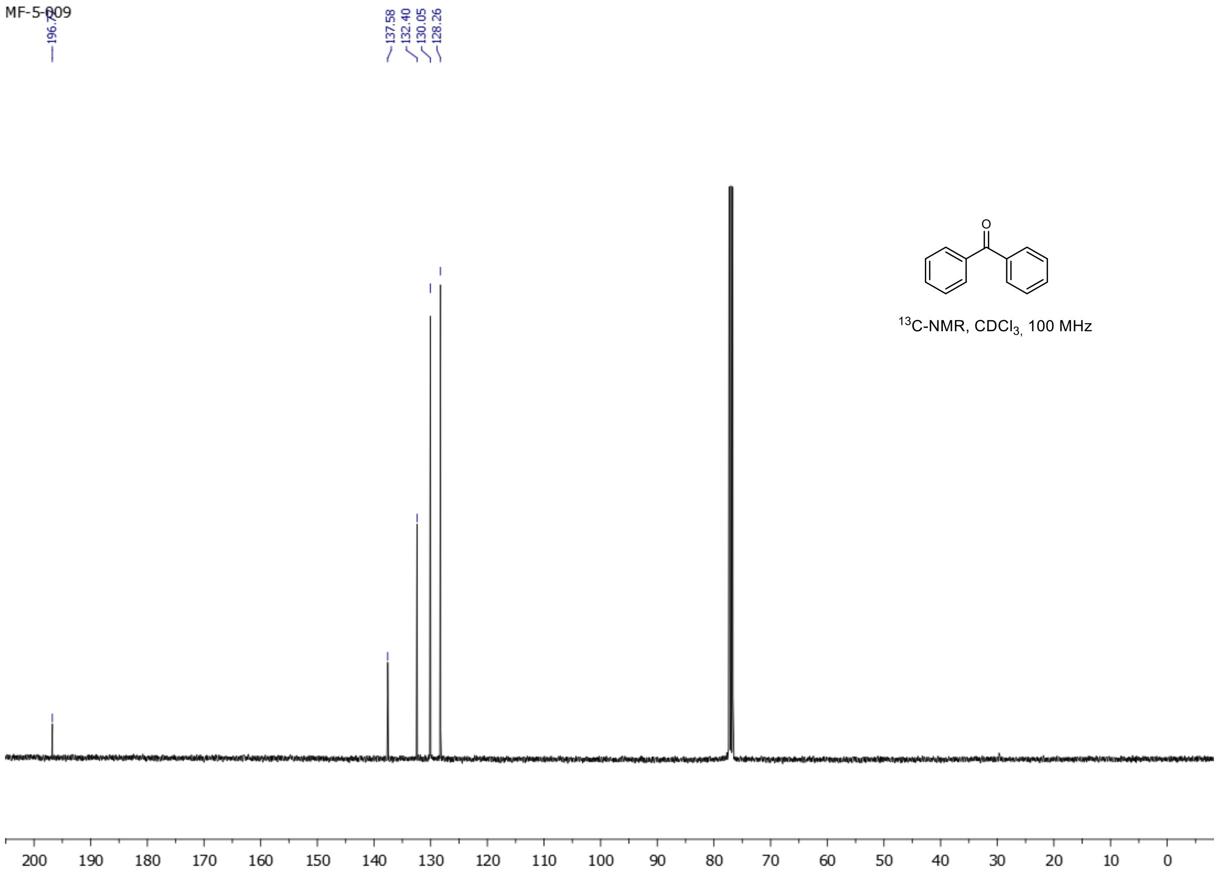

(S)-(3-(1-Methylpyrrolidin-2-yl)phenyl)(phenyl)methanone (51)

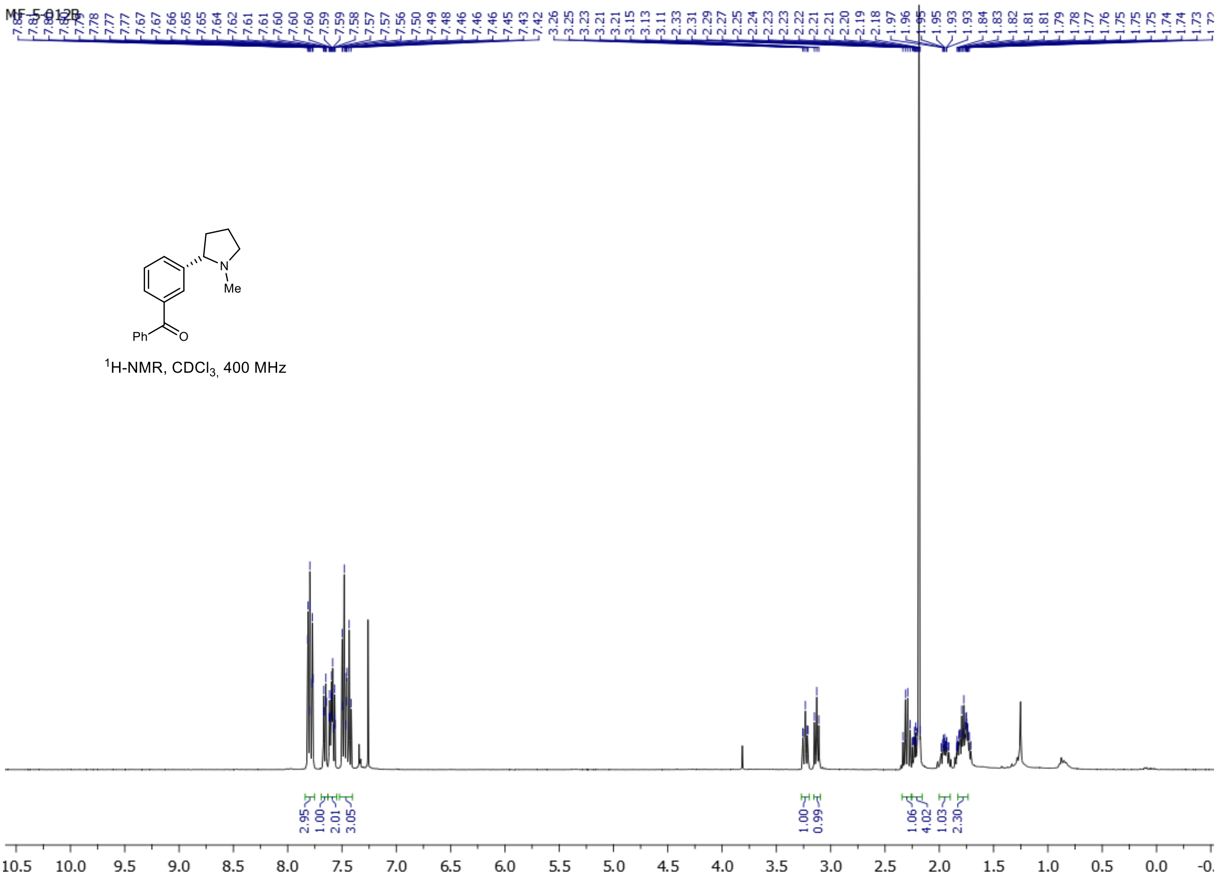

^{1}H-NMR, $CDCl_3$, 400 MHz

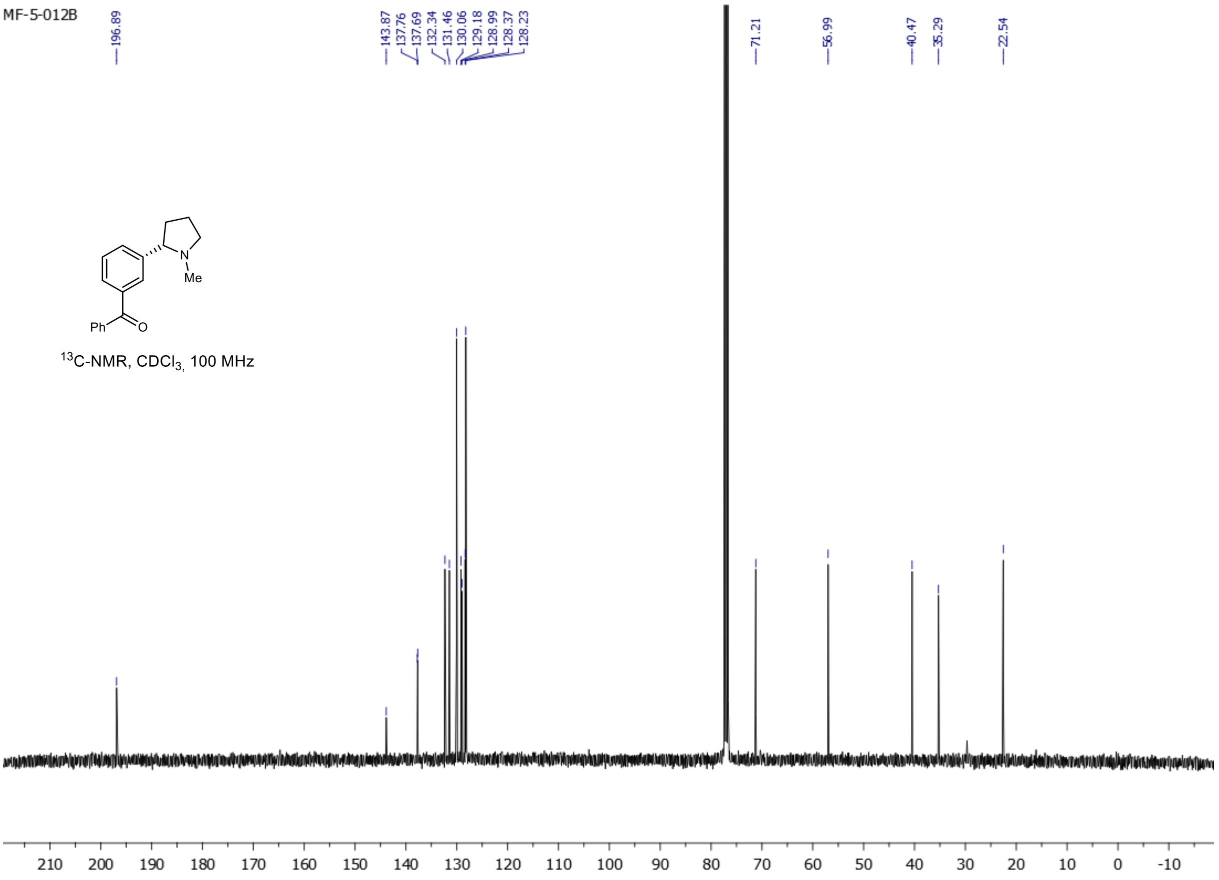

^{13}C-NMR, $CDCl_3$, 100 MHz

(S)-(3-(1-Methylpyrrolidin-2-yl)phenyl-1-^{13}C)(phenyl)methanone ([$^{13}C_1$]51)

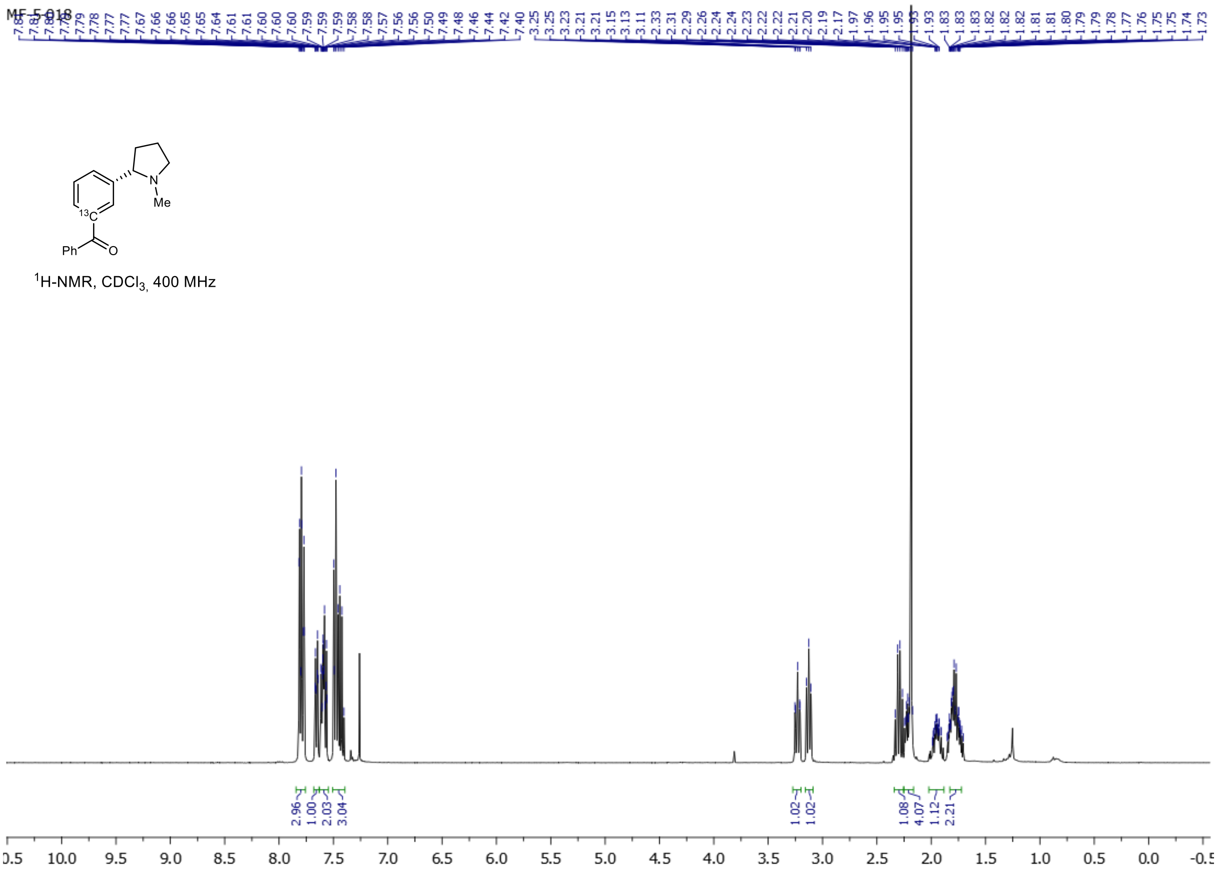

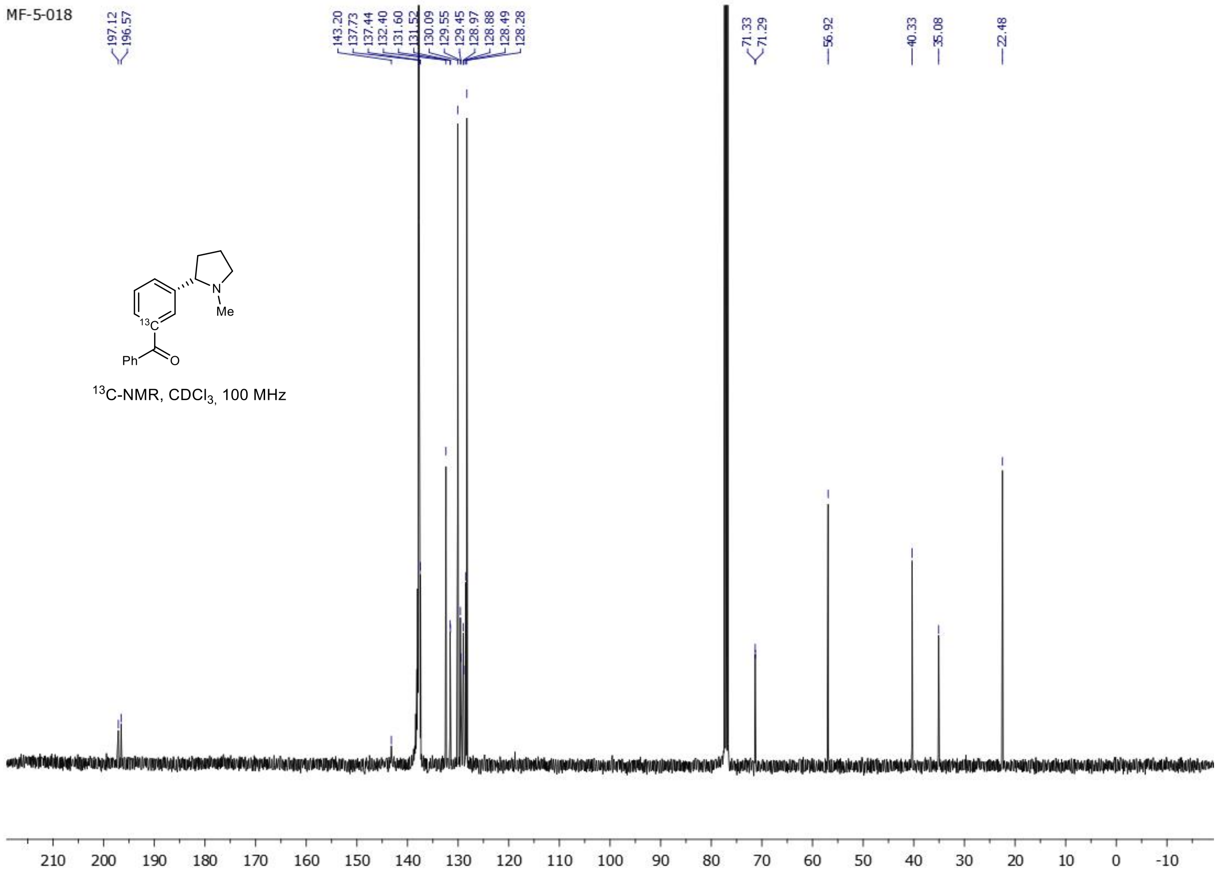

Phenyl(d_5-phenyl)methanol-^{13}C ([$^2H_5$, $^{13}C_1$]52)

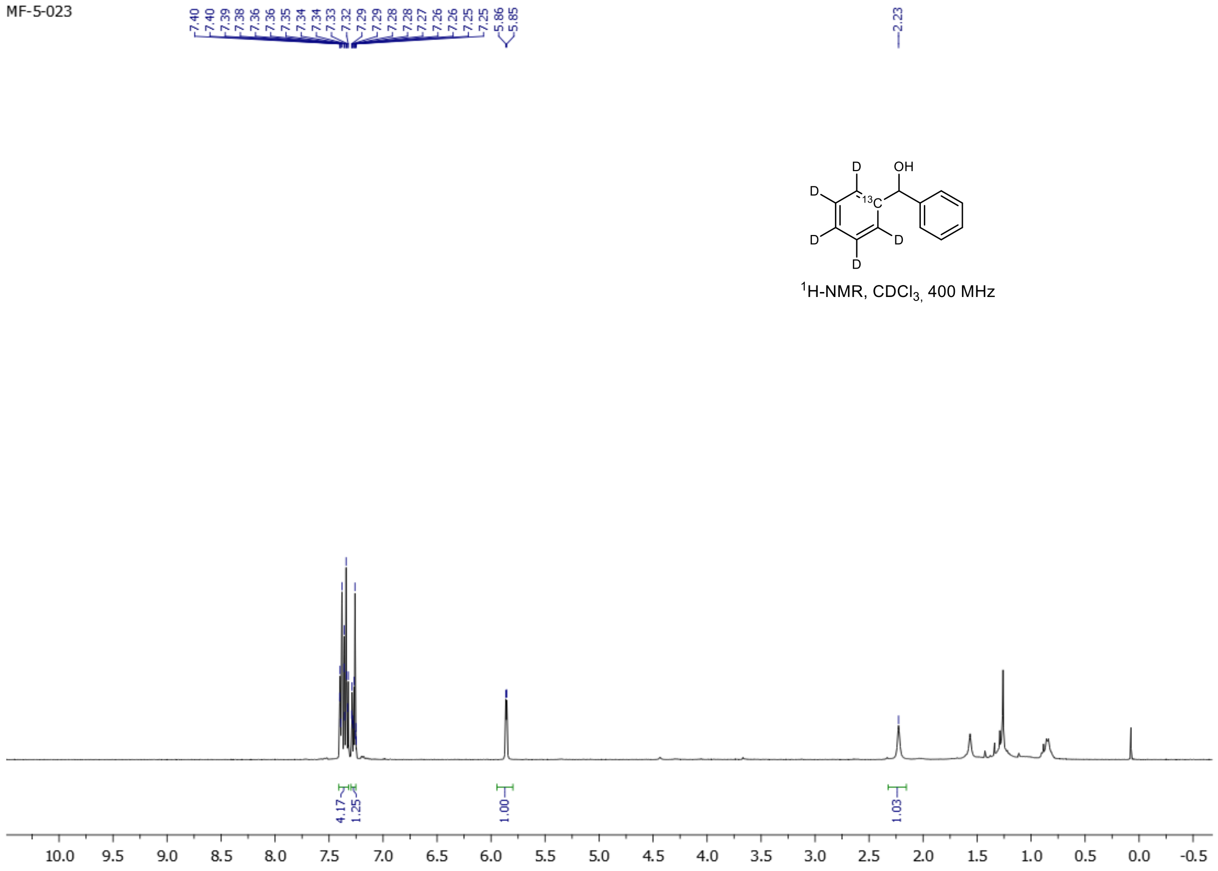

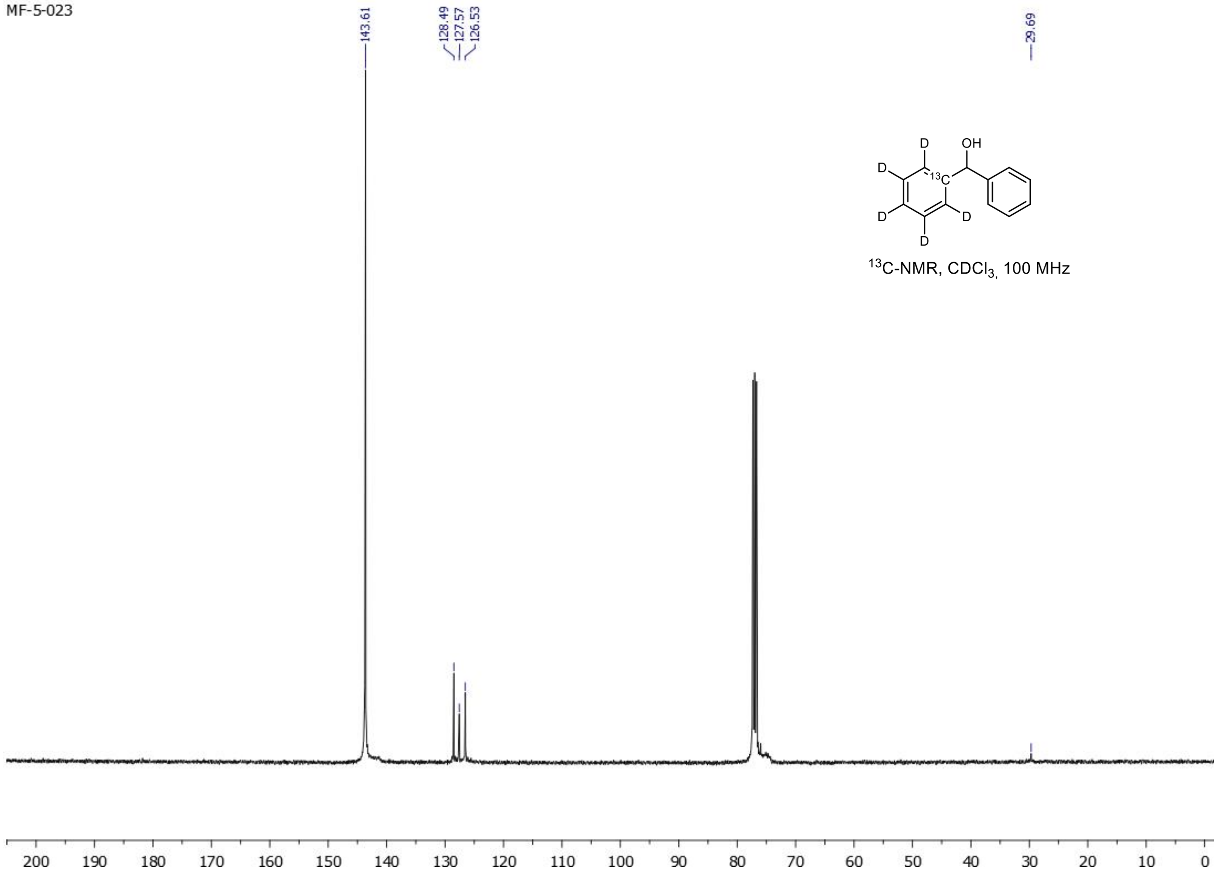

14. Supplementary References

[1] Štacková, L.; Štacko, P.; Klán, P. *J. Am. Chem. Soc.* **2019**, 141, 7155.

[2] Wang, L.; Wang, Z.-X. *Org. Lett.* **2007**, *9*, 4335.

[3] Niakan, M.; Asadi, Z.; Masteri-Farahani, M.; *ChemistrySelect*. **2019**, *4*, 1766.

[4] Yu, C.; Huang, R.; Patureau, F. W. *Angew. Chem. Int. Ed*. **2022**, *61*, e202201142

[5] Boyle, B. T.; Levy, J. N.; d Lescure, L.; Paton, R. S.; McNally, A. *Science*, **2022**, *378*, 773.

[6] *APEX3 Crystallography Software Suite,* Ver. 2019.1-0; Bruker AXS: Madison, WI, **2019**.

[7] *SAINT,* Ver. 8.40A; Bruker Nano: Madison, WI, **2019**.

[8] Sheldrick, G. M. *Acta Crystallogr., Sect. A* **2015**, *71*, 3–8.

[9] Sheldrick, G. M. *Acta Crystallogr., Sect. C* **2015**, *71*, 3–8.

[10] Hübschle, C. B.; Sheldrick, G. M.; Dittrich, B.; *J. Appl. Crystallogr.* **2011**, *44*, 1281–1284.

[11] a) Burnett, M. N.; Johnson, C. K.; *ORTEPIII*, Report ORNL-6895; Oak Ridge National Laboratory: TN, **1996**. b) Farrugia, L. J. *J. Appl. Crystallogr.* **2012**, *45,* 849–854.
